# Supplementary material for: Exploration of Novel Urolithin C Derivatives as Non-Competitive Inhibitors of Liver Pyruvate Kinase
Source: Pharmaceuticals (Basel). 2023 Apr 28;16(5):668. doi: 10.3390/ph16050668 (PMC10224133; doi:10.3390/ph16050668)

Supporting Information

Exploration of Novel Urolithin C Derivatives as Non-Competitive Inhibitors of Liver Pyruvate Kinase

Umberto Maria Battisti[1,2,†], Leticia Monjas[1,2,†], Fady Akladios[1,2], Josipa Matic[1,2], Eric Andresen[1], Carolin H. Nagel[1], Malin Hagkvist[1], Liliana Håversen[3,4], Woonghee Kim[2], Mathias Uhlen[2], Jan Borén[3,4], Adil Mardinoğlu[2,5] and Morten Grøtli[1,*]

[1]Department of Chemistry and Molecular Biology, University of Gothenburg, SE-412 96 Gothenburg, Sweden

[2]Science for Life Laboratory, KTH - Royal Institute of Technology, SE-171 65 Stockholm, Sweden

[3]Department of Molecular and Clinical Medicine, University of Gothenburg, SE-413 45, Gothenburg, Sweden

[4]Sahlgrenska University Hospital, SE-413 45, Gothenburg, Sweden

[5]Centre for Host-Microbiome Interactions, Faculty of Dentistry, Oral & Craniofacial Sciences, King's College London, London SE1 9RT, UK

[†]These authors contributed equally to this work.

^{1}H and ^{13}C NMR spectra of selected compounds

3-Hydroxy-9-methoxy-6H-benzo[c]chromen-6-one (**5**)

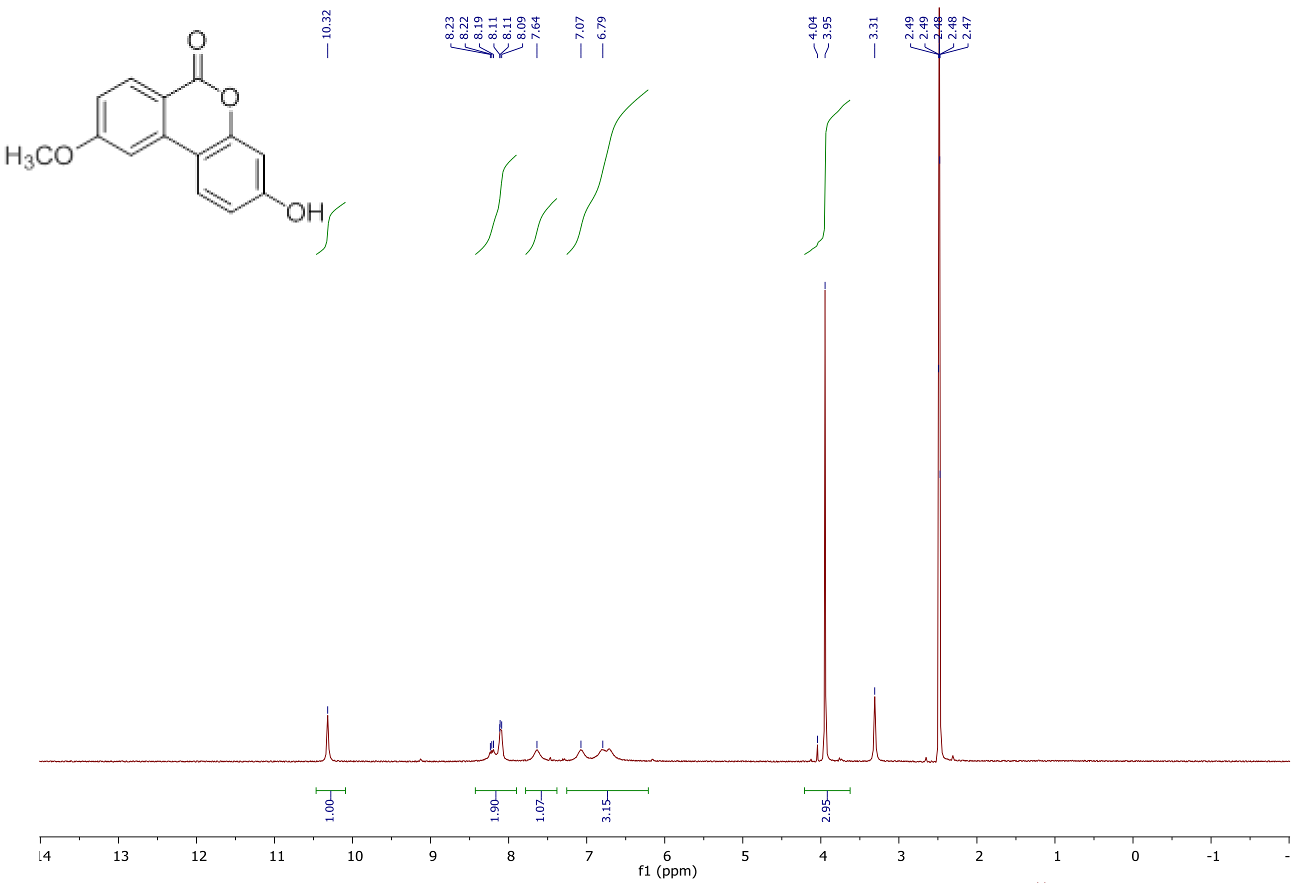

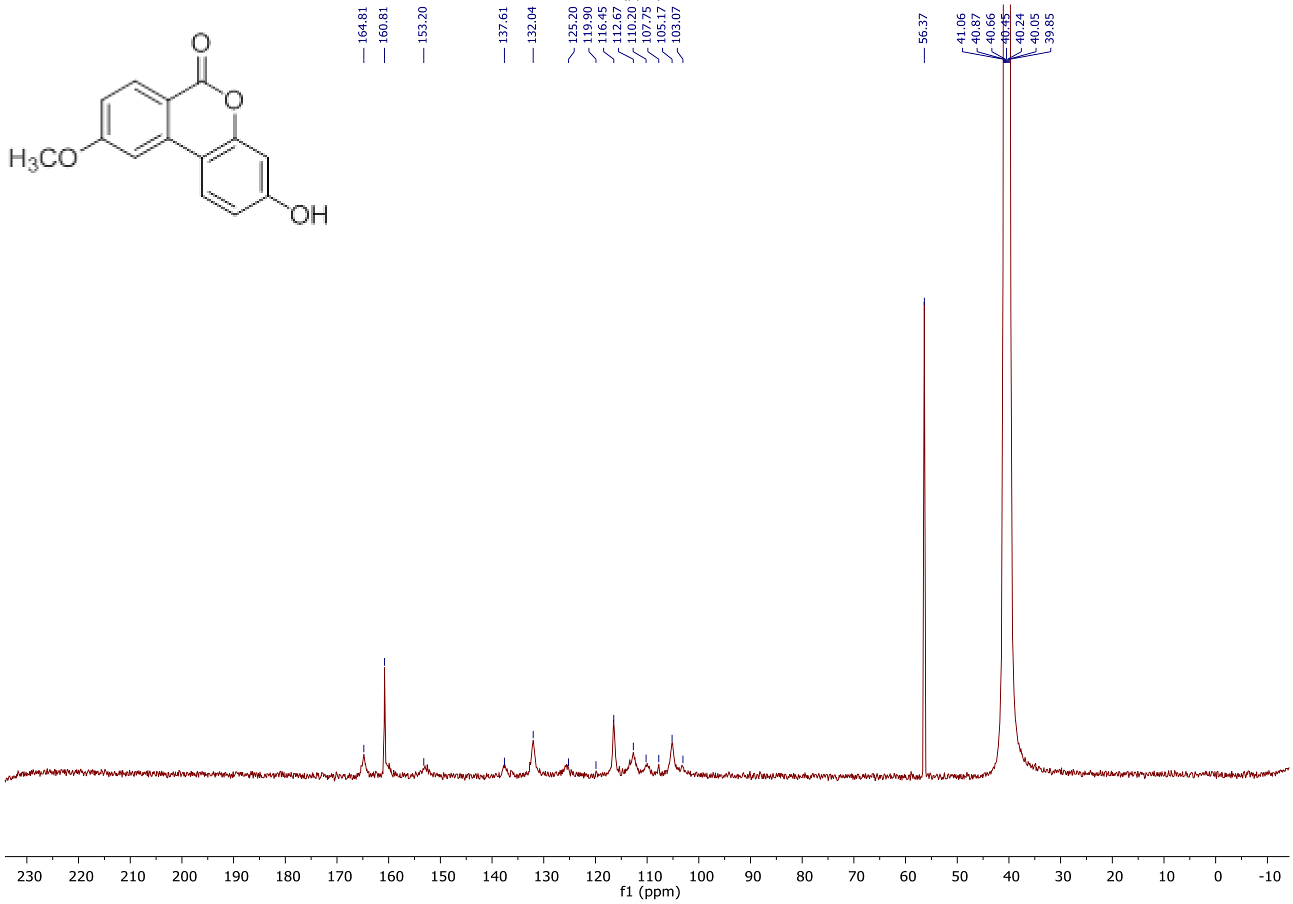

3,9-dihydroxy-6H-benzo[c]chromen-6-one (**6**)

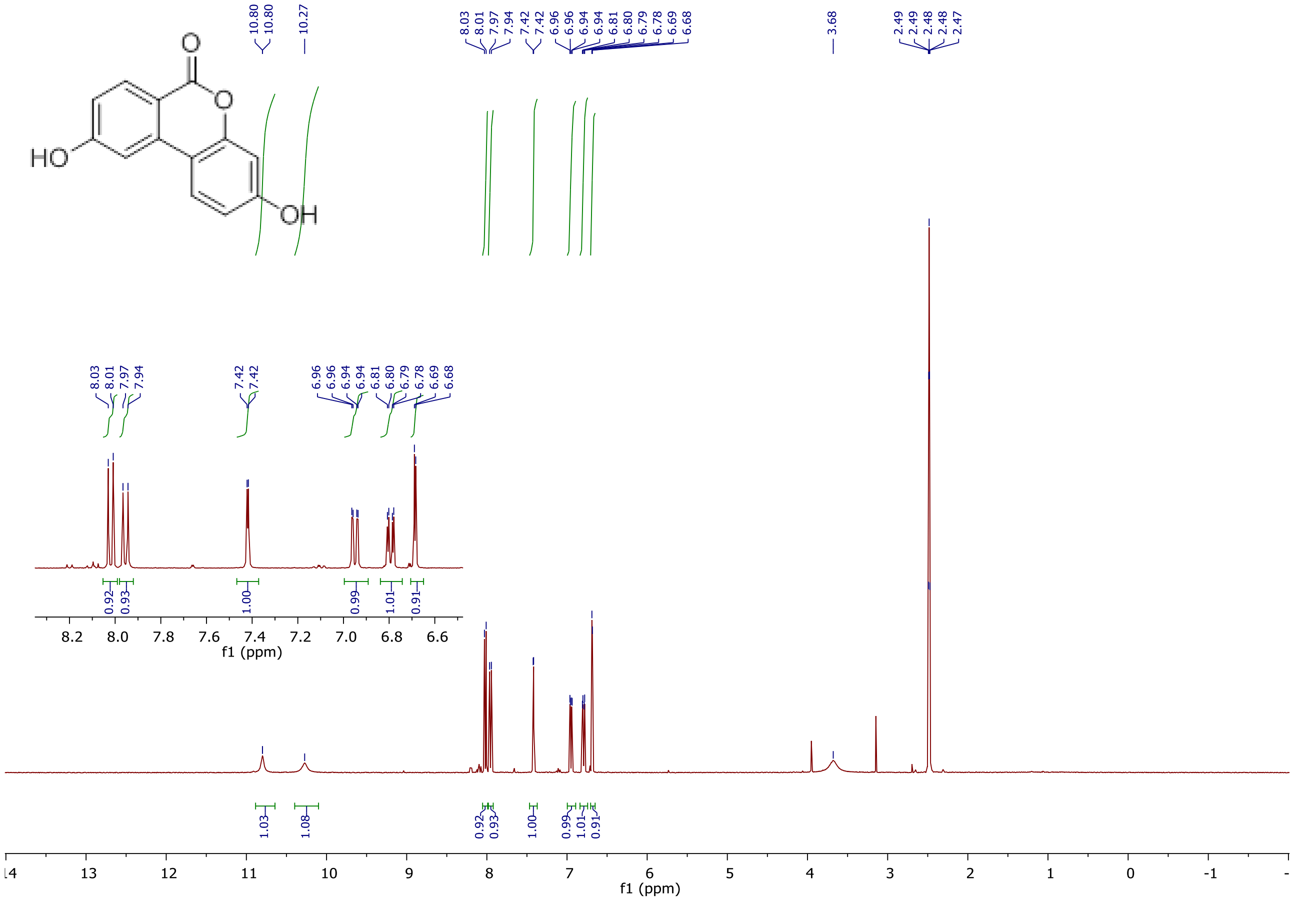

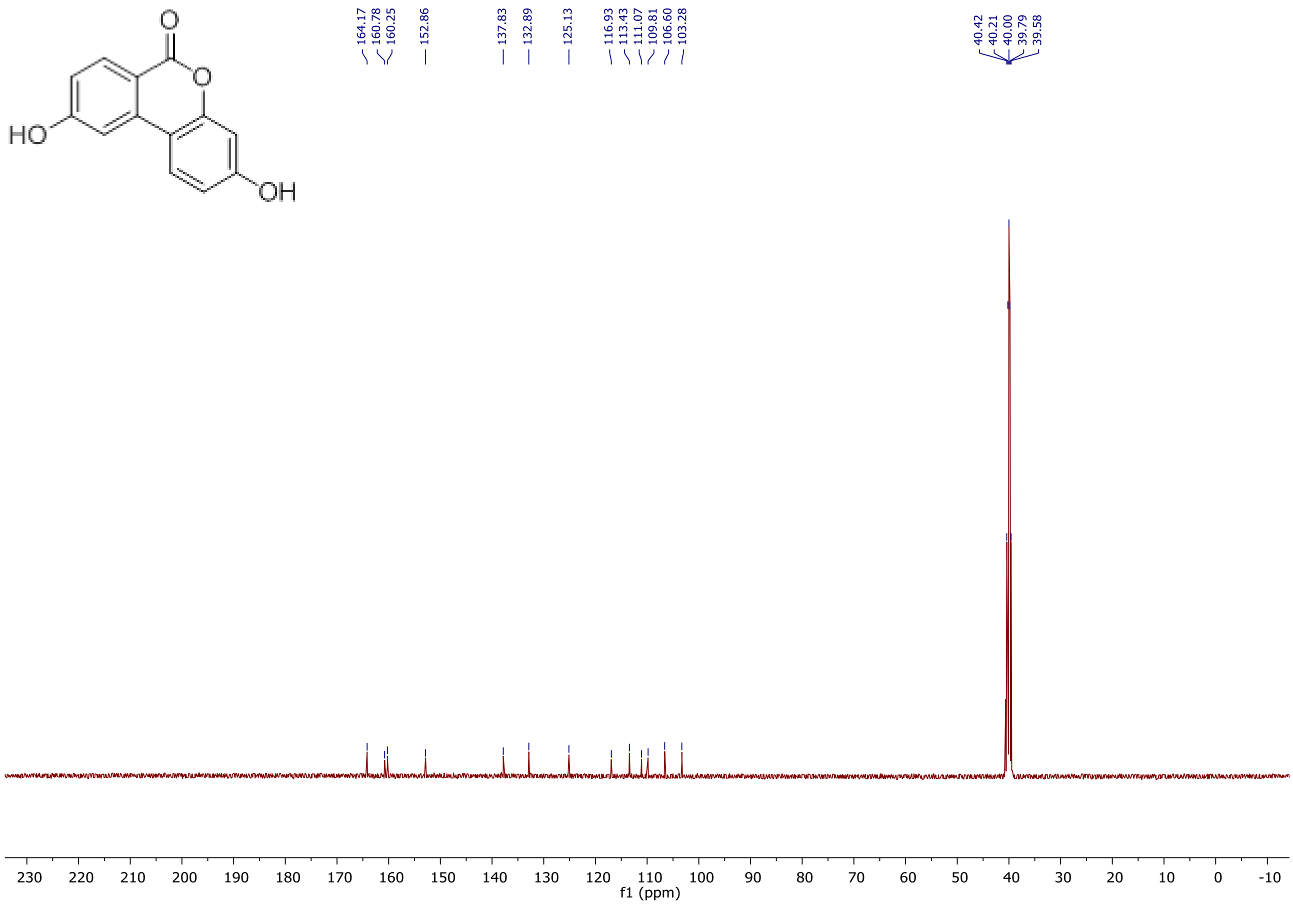

2-Bromo-4,5-dimethoxybenzoic acid (**8**)

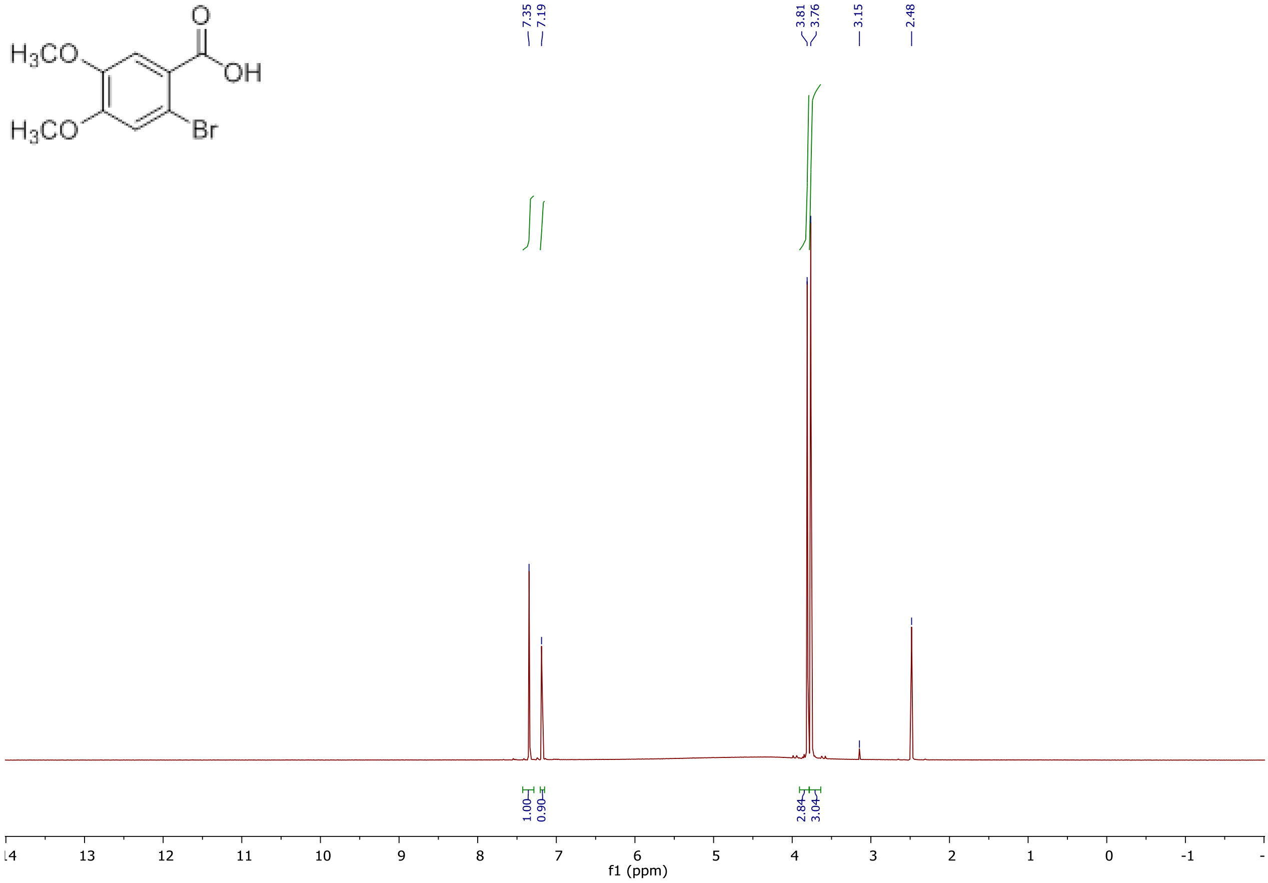

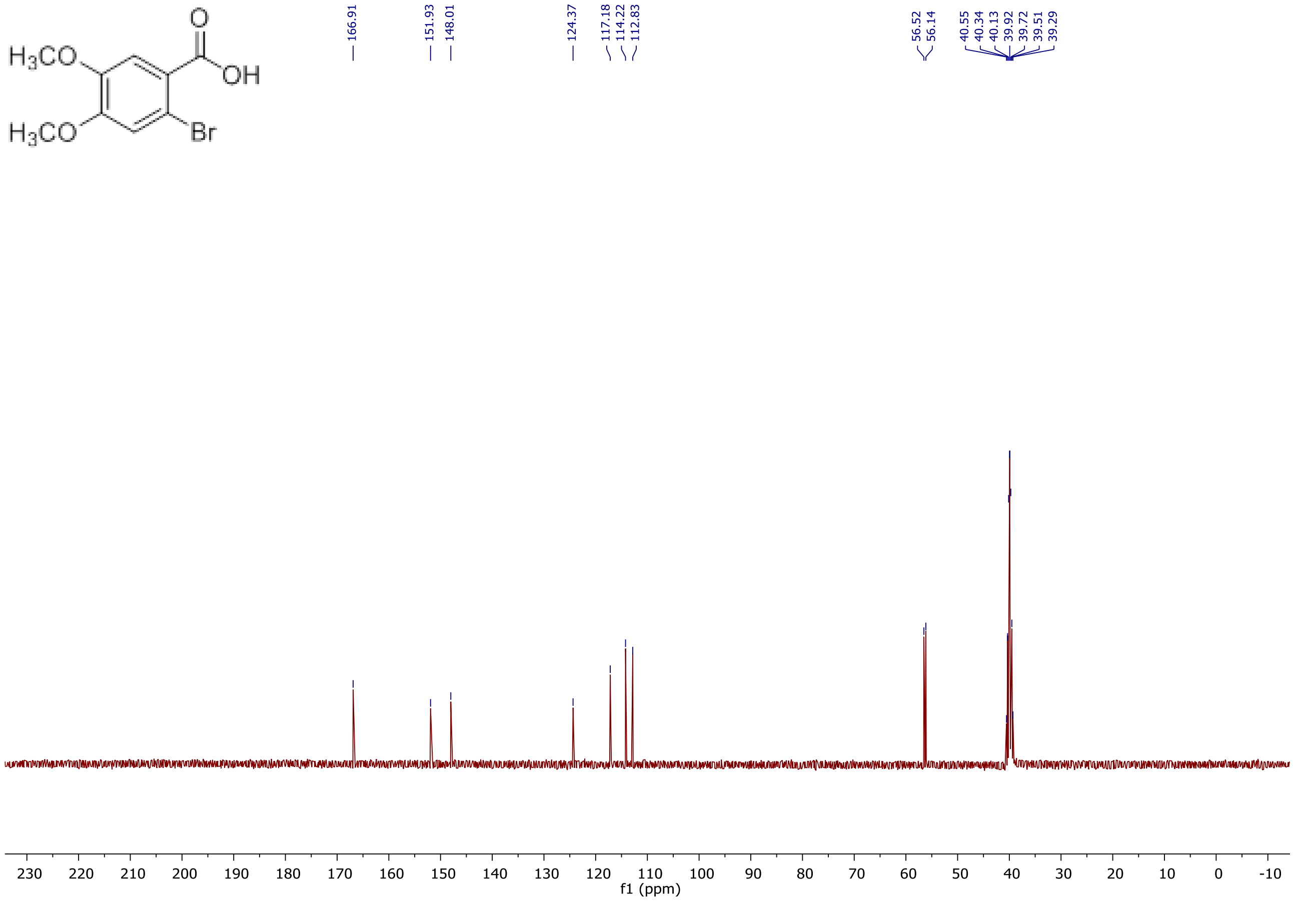

Methyl 2-bromo-4,5-dimethoxybenzoate (**9**)

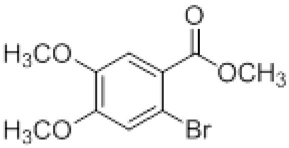

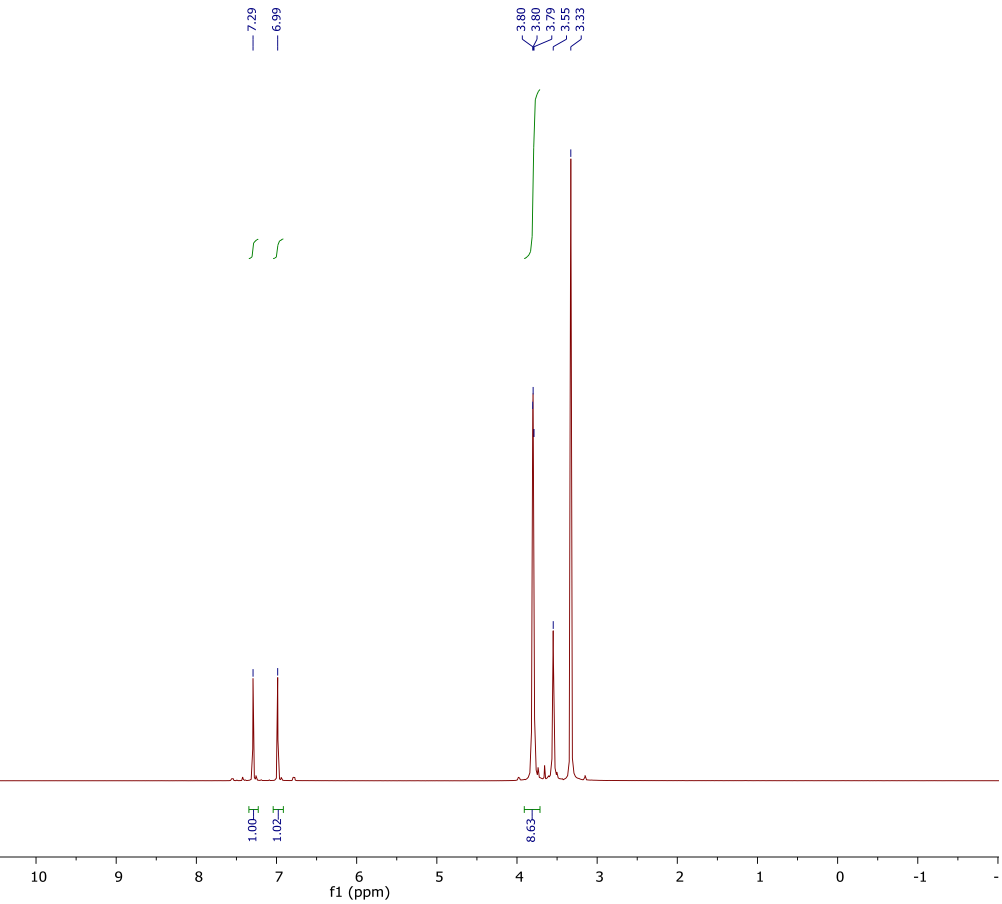

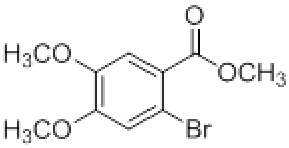

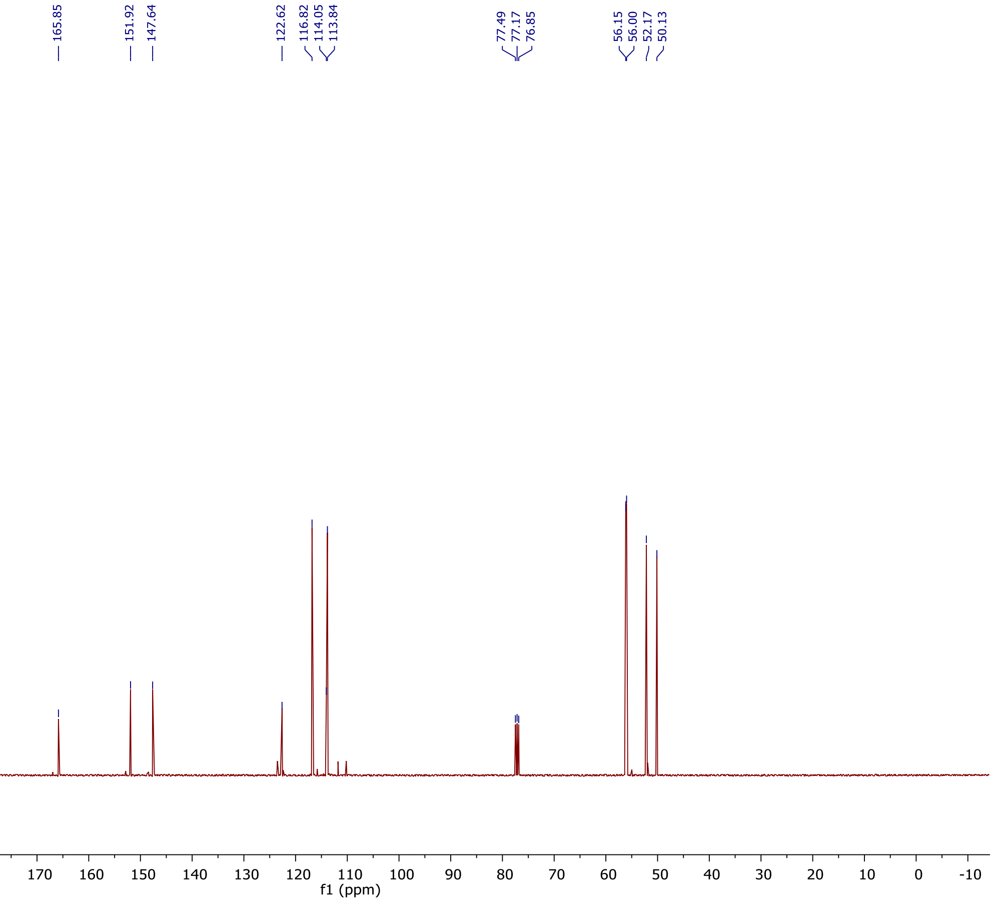

Methyl 4,5-dimethoxy-[1,1'-biphenyl]-2-carboxylate (**10**)

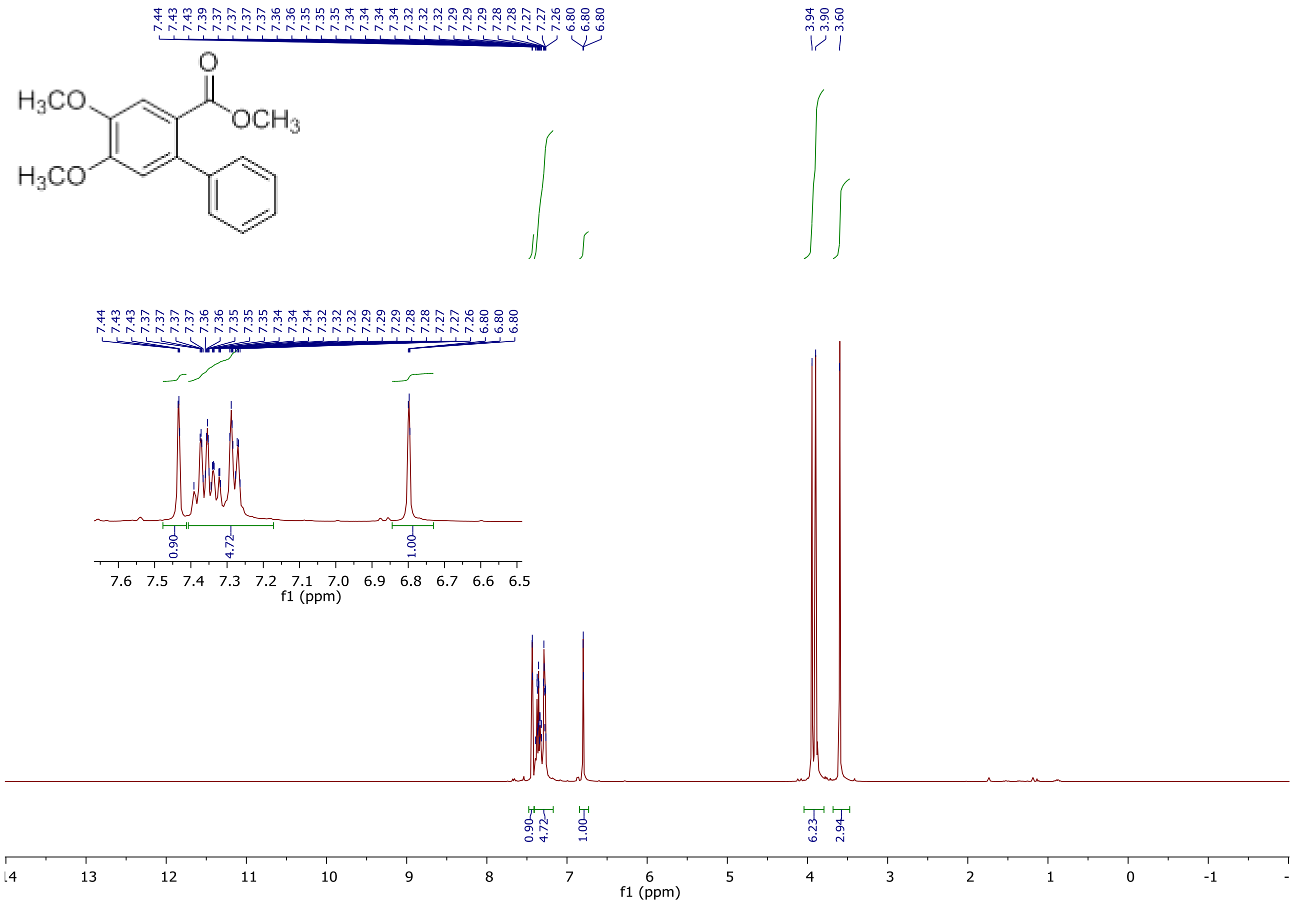

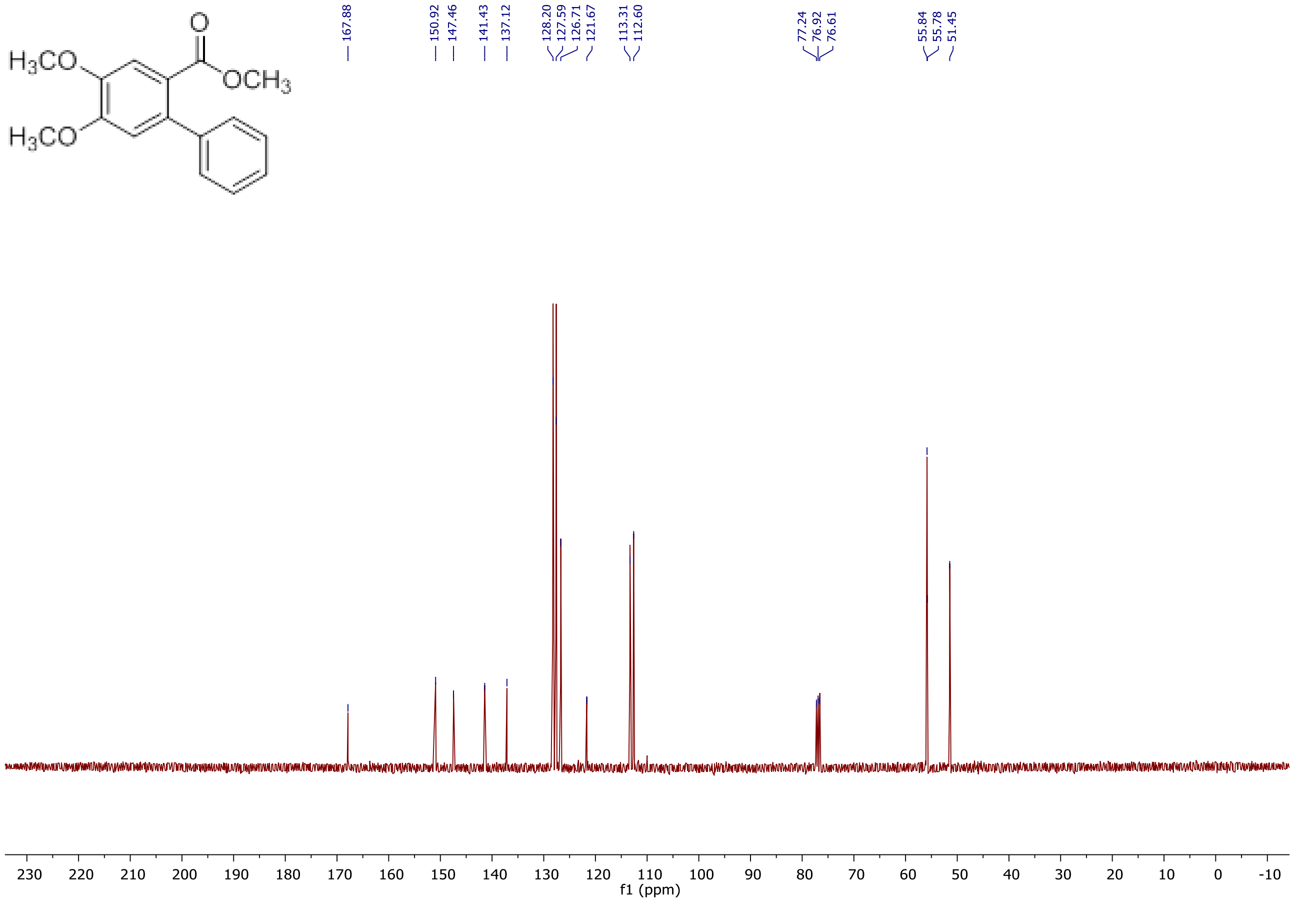

4,5-Dimethoxy-[1,1'-biphenyl]-2-carboxylic acid (**11**)

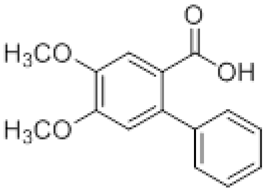

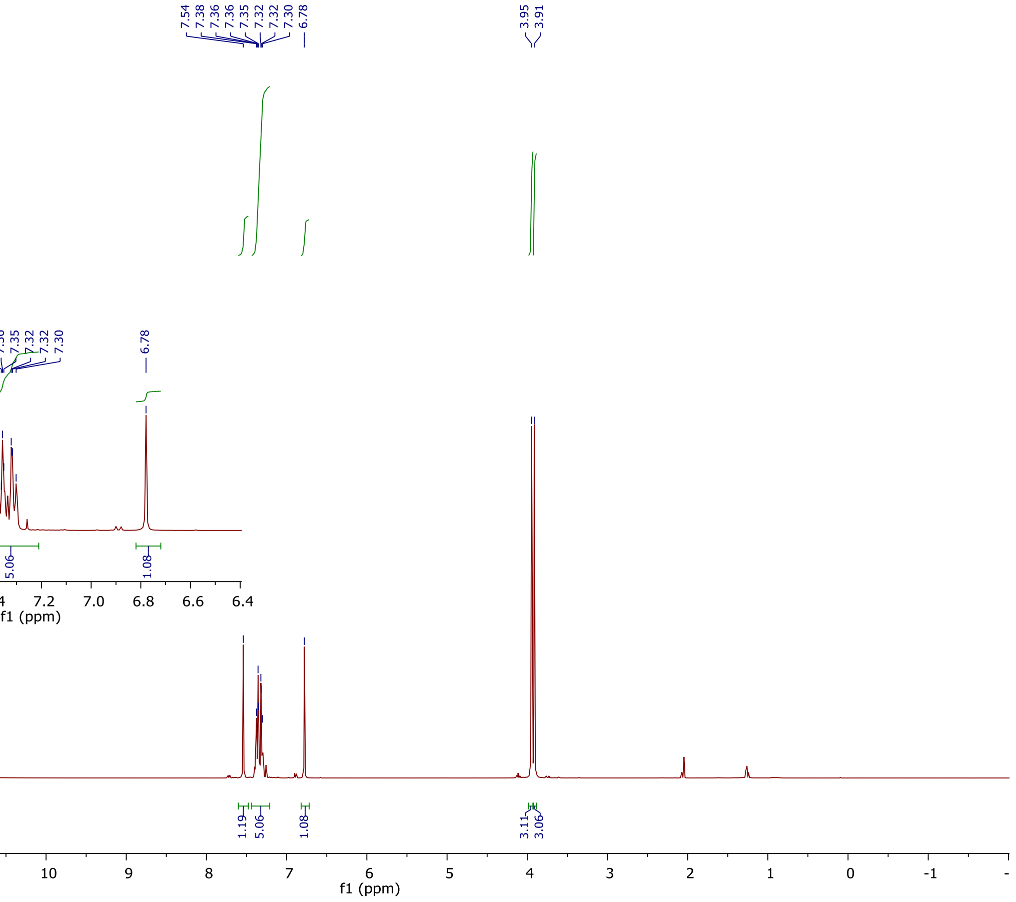

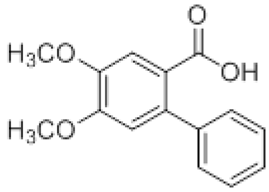

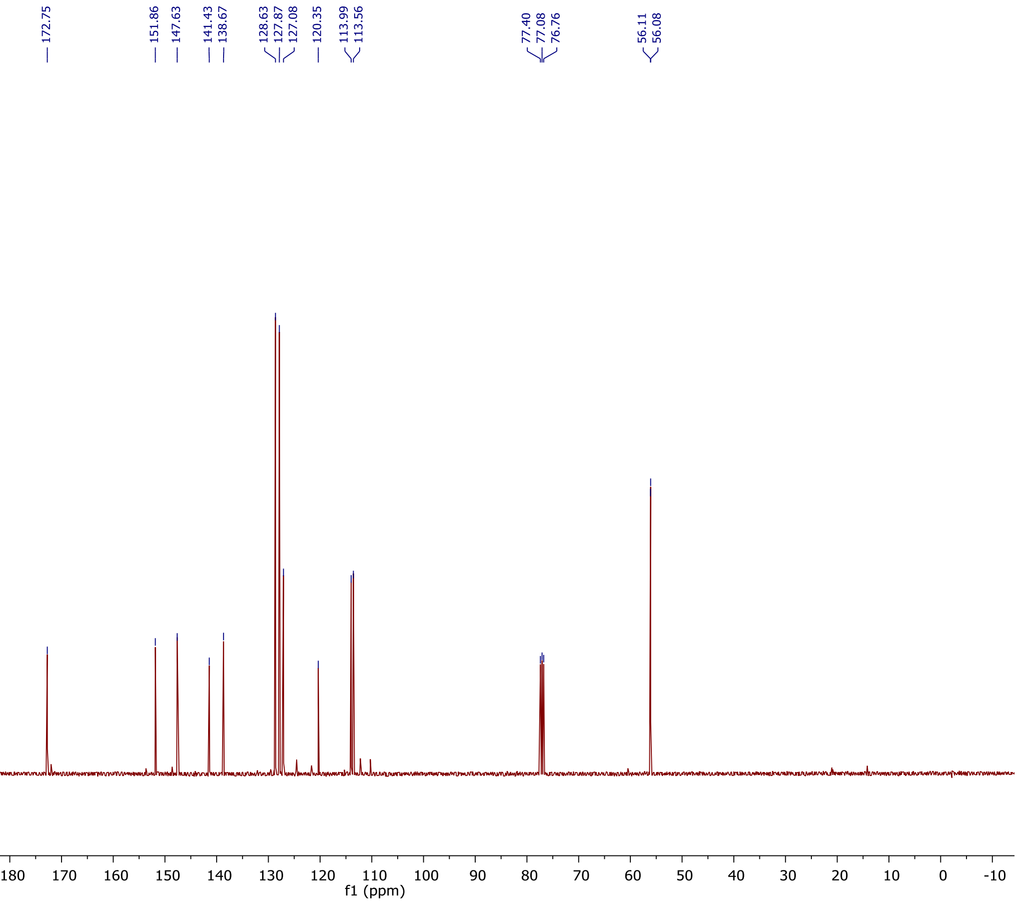

8,9-Dimethoxy-6H-benzo[c]chromen-6-one (**12**)

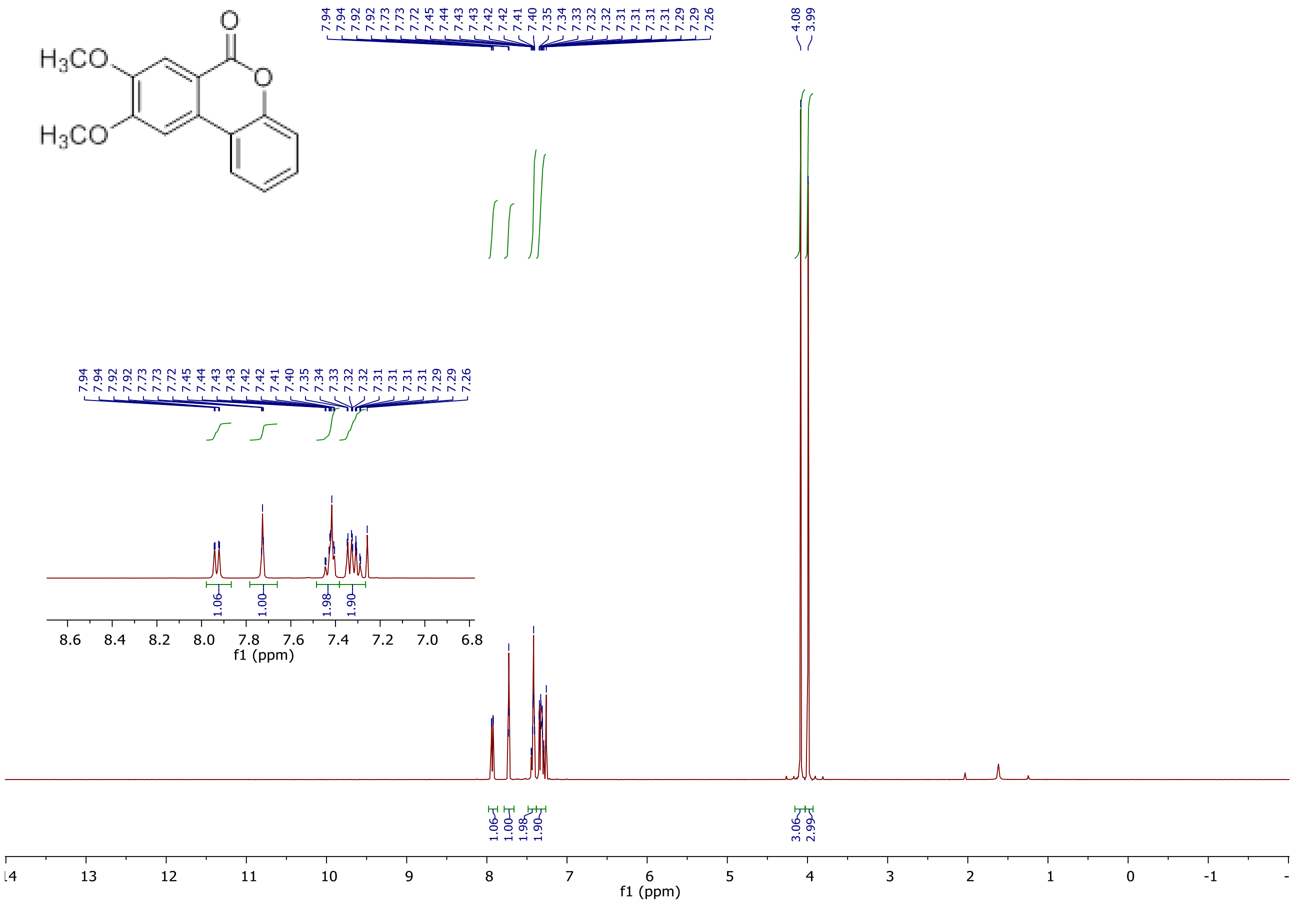

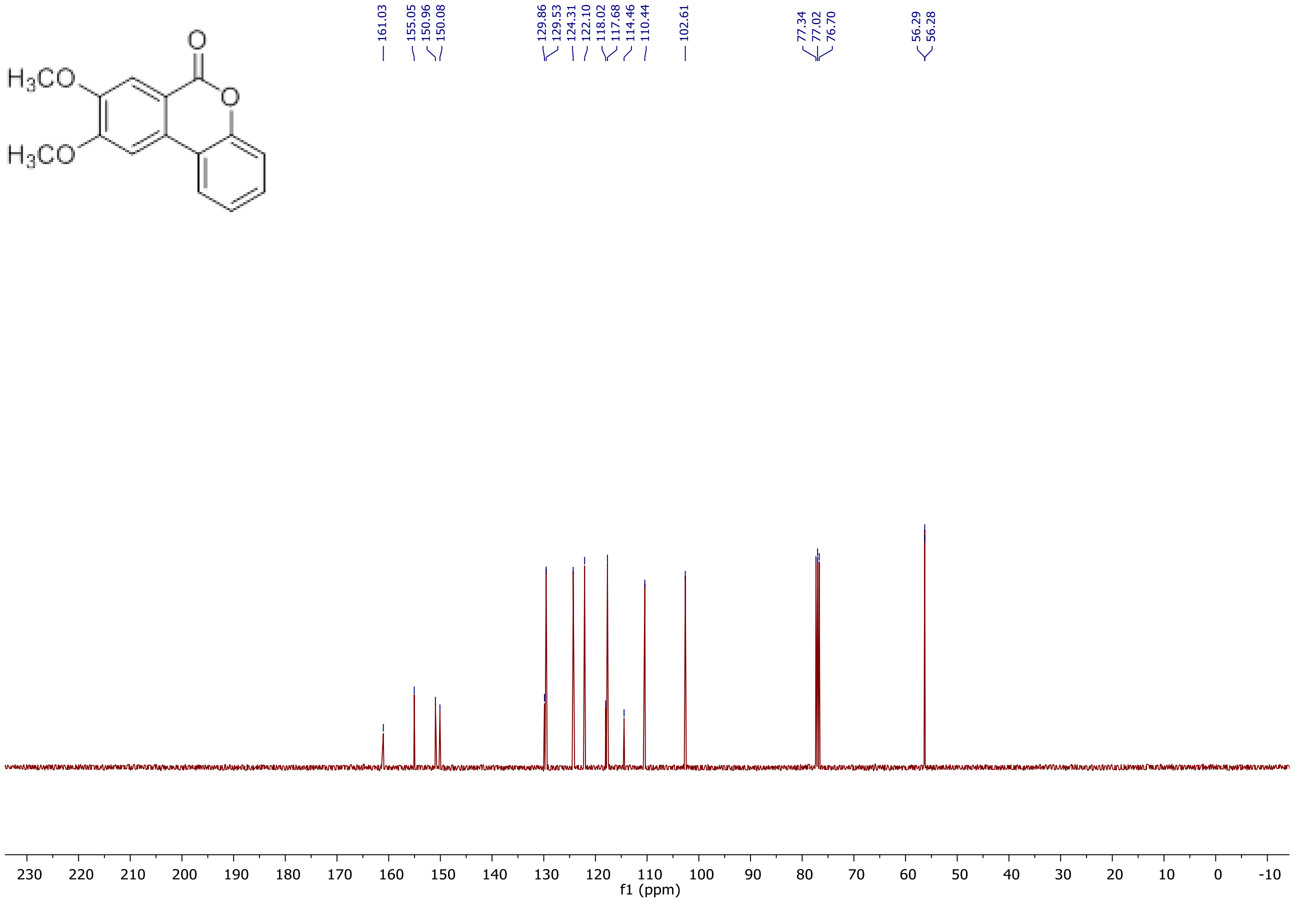

8,9-Dihydroxy-6H-benzo[c]chromen-6-one (**13**)

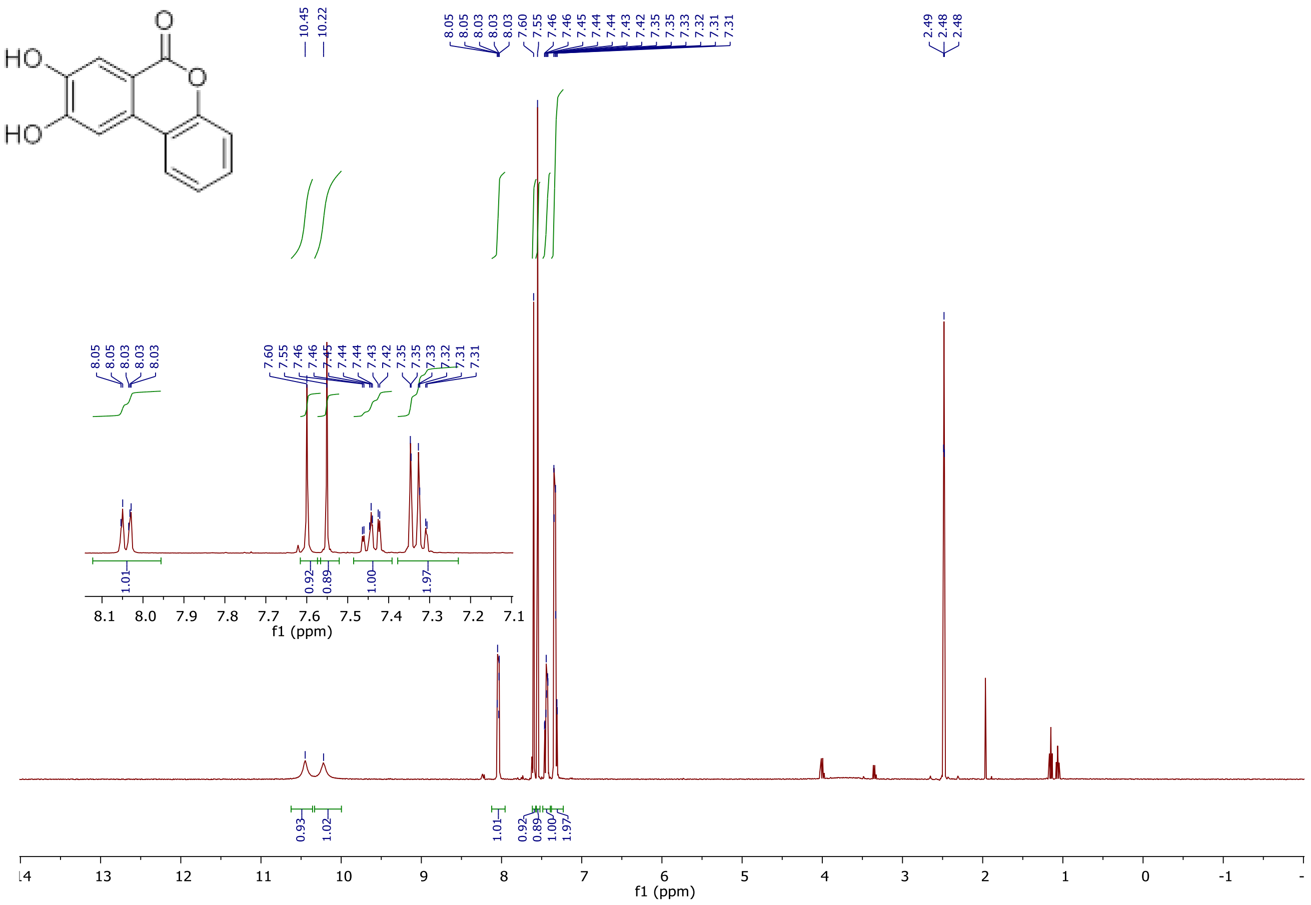

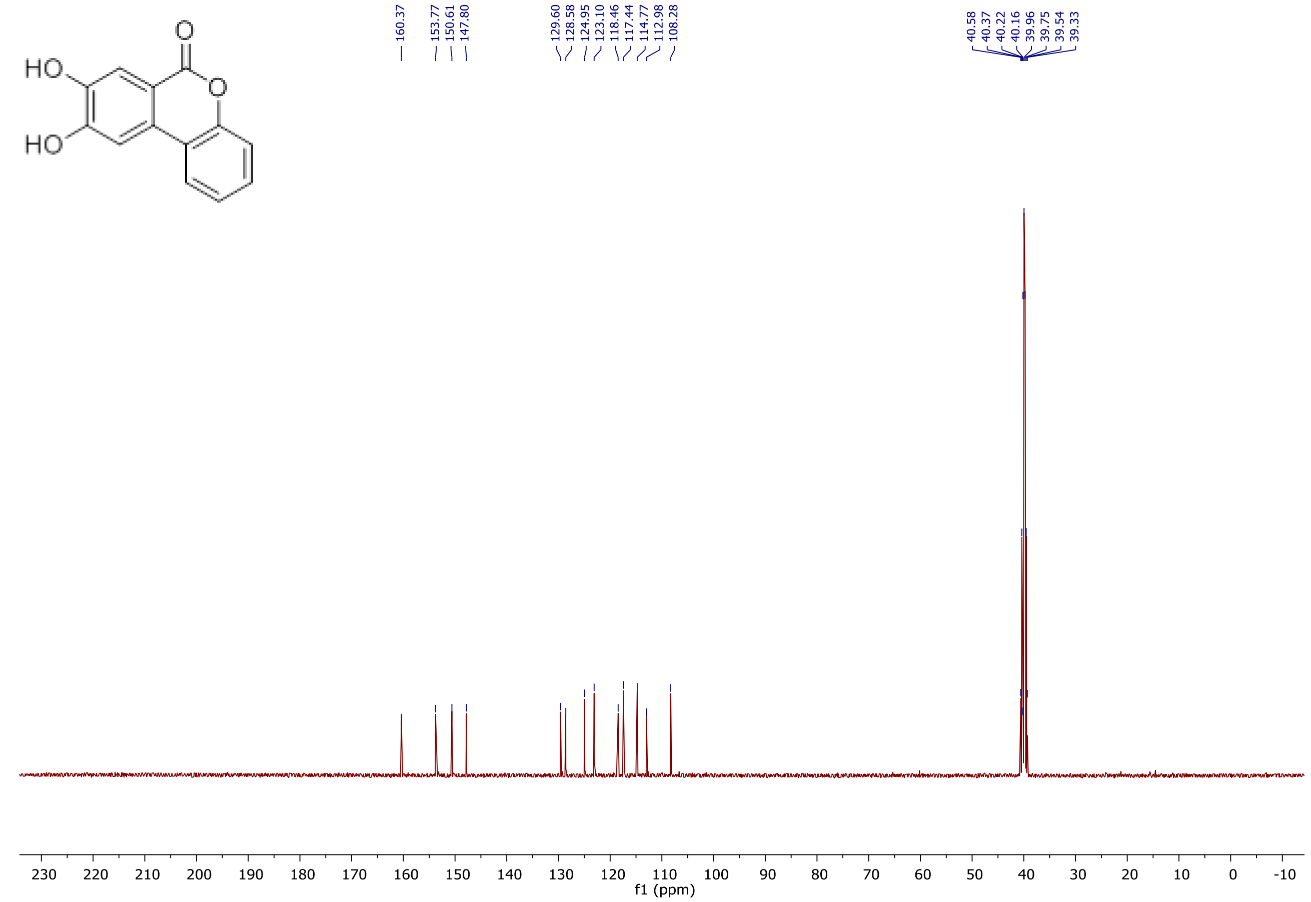

3,4,4'-Trimethoxy-1,1'-biphenyl (**16**)

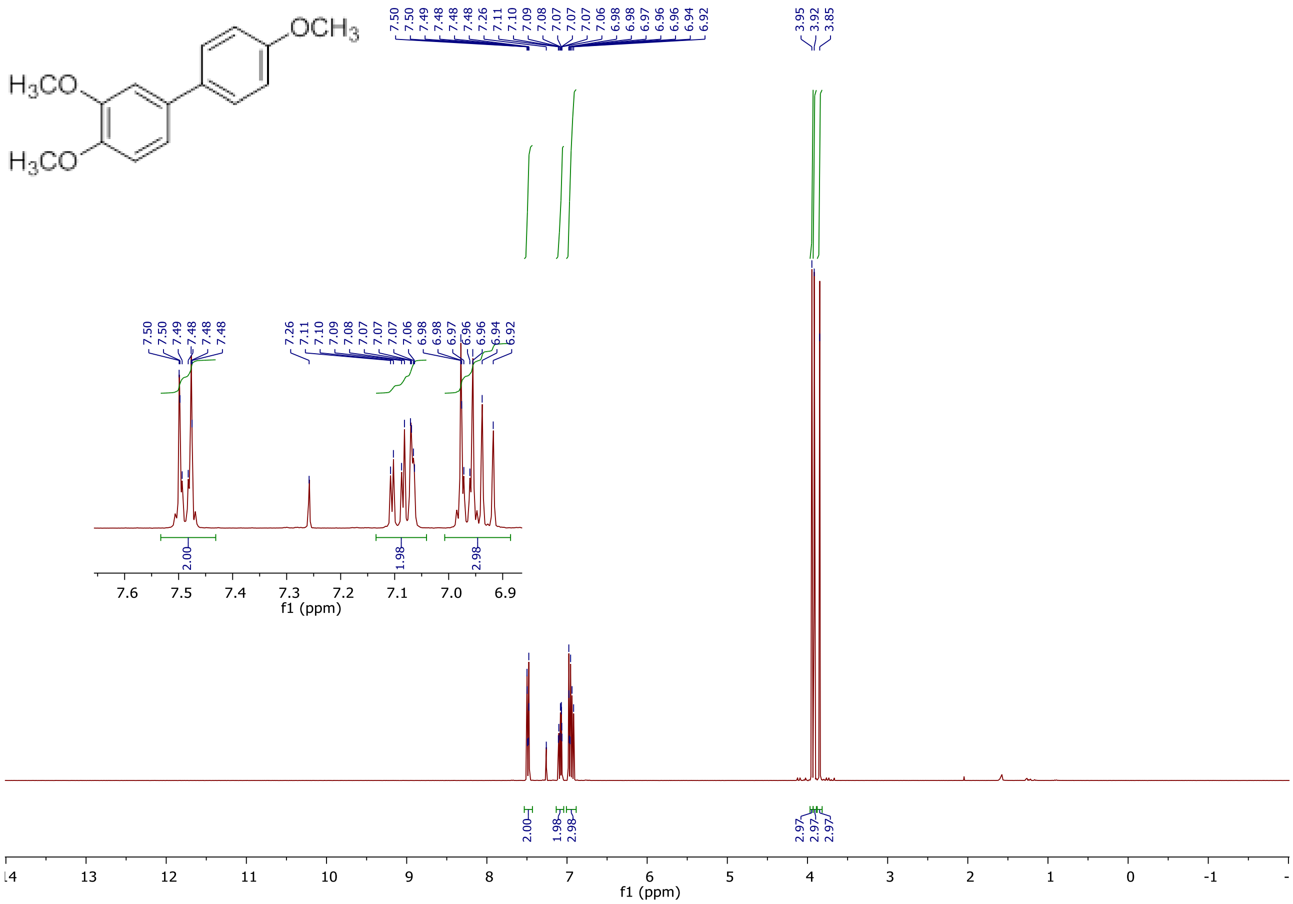

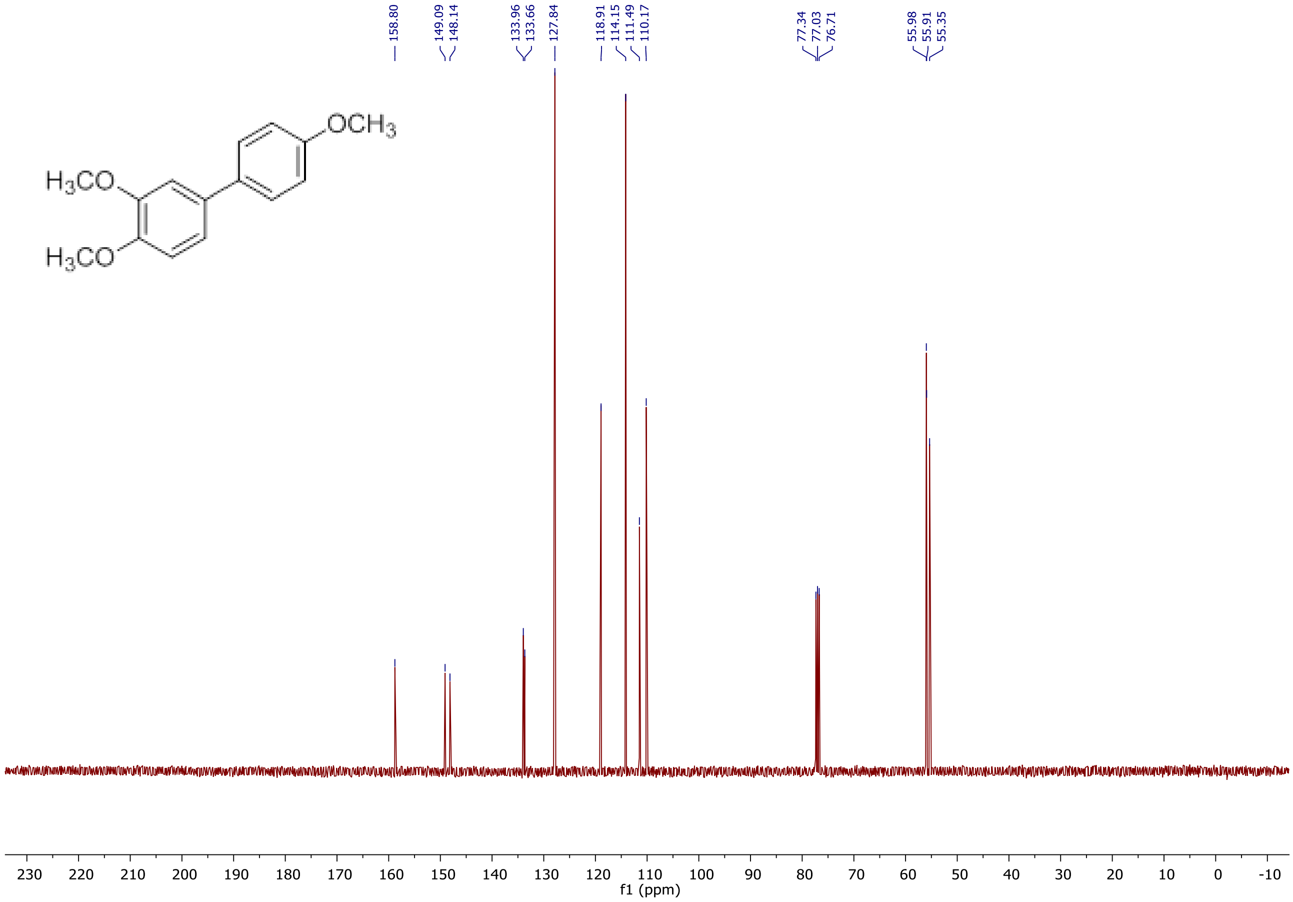

[1,1'-Biphenyl]-3,4,4'-triol (**17**)

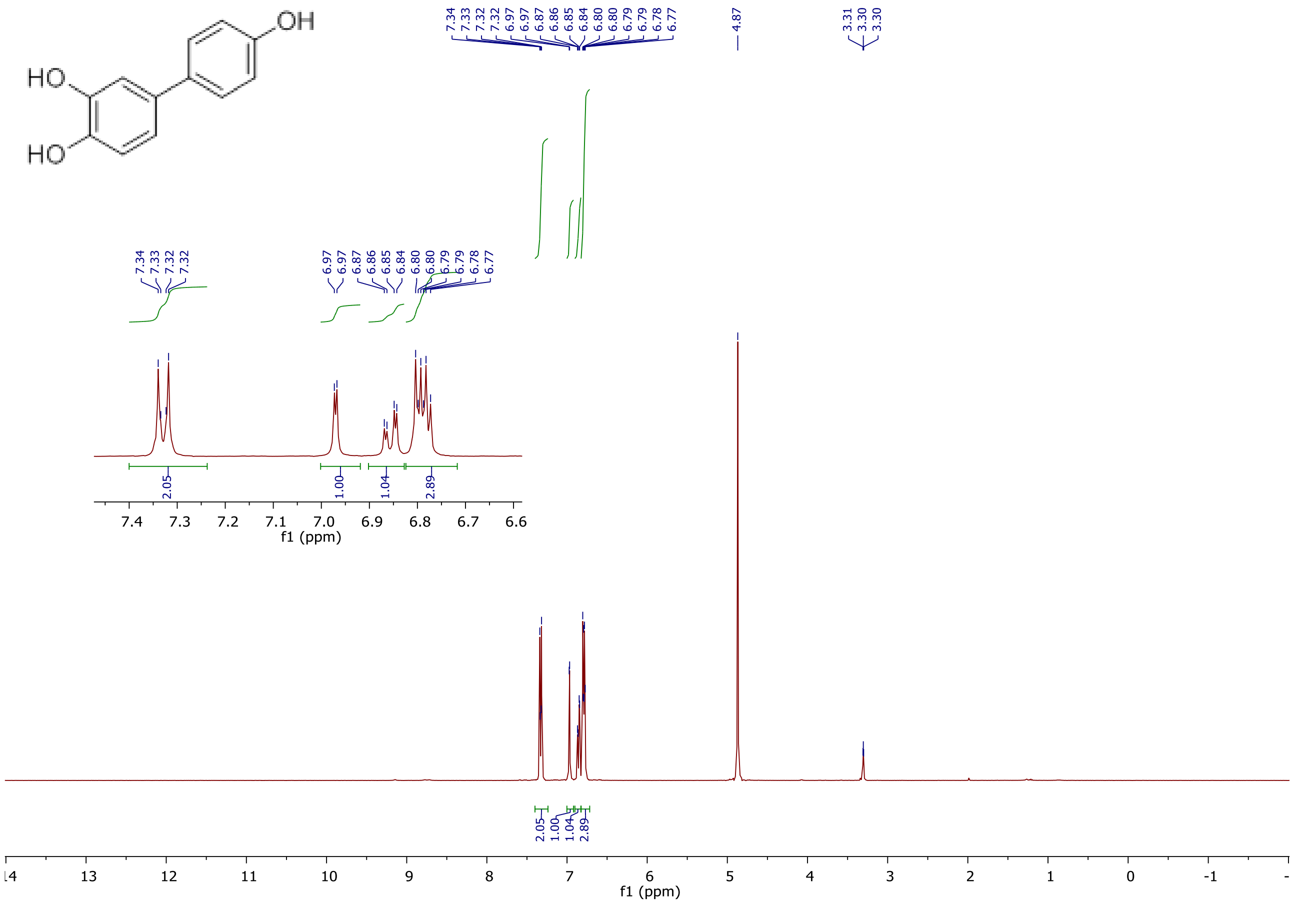

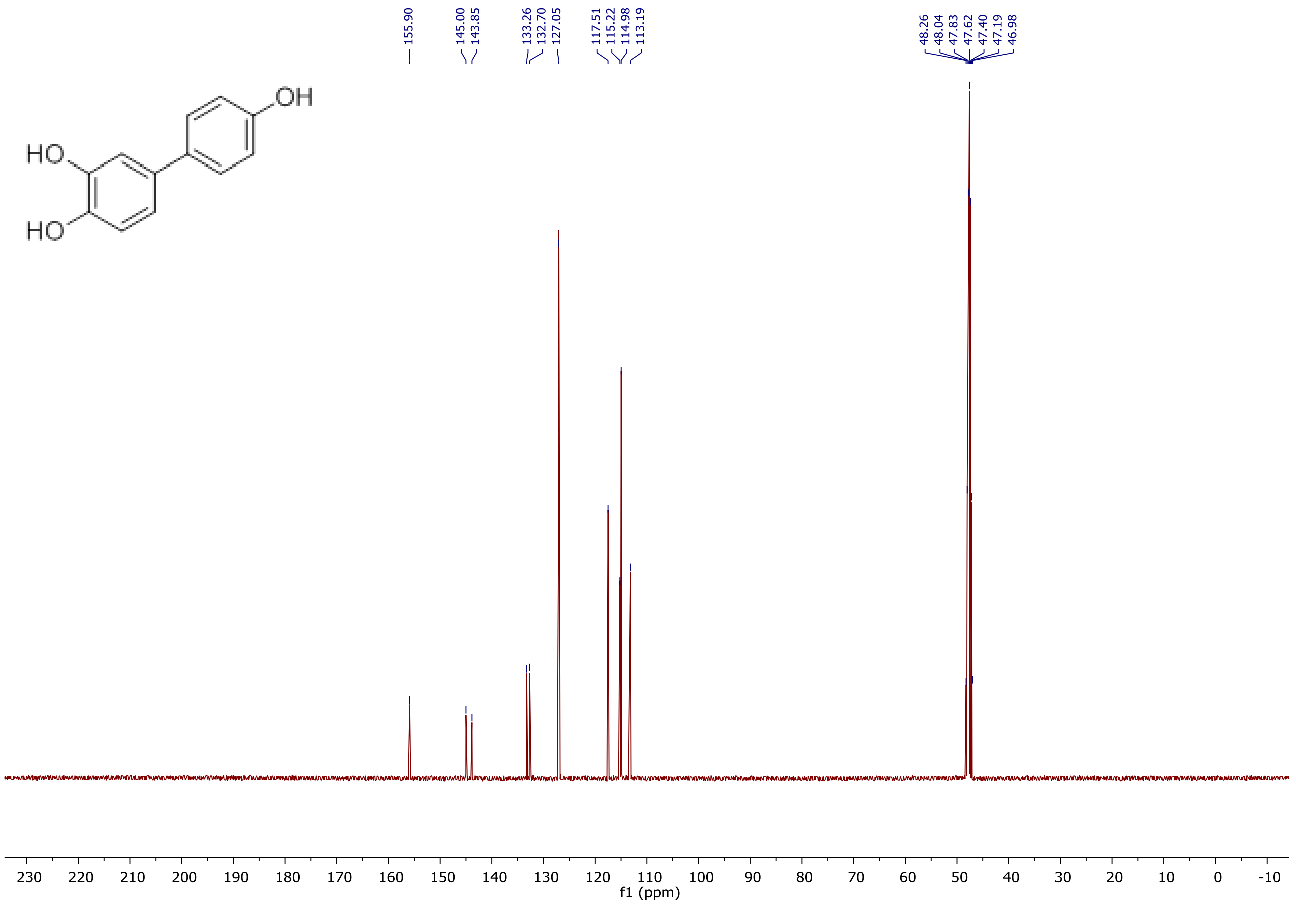

Methyl 4'-((tert-butoxycarbonyl)(methyl)amino)-4,5-dimethoxy-[1,1'-biphenyl]-2-carboxylate (**20**)

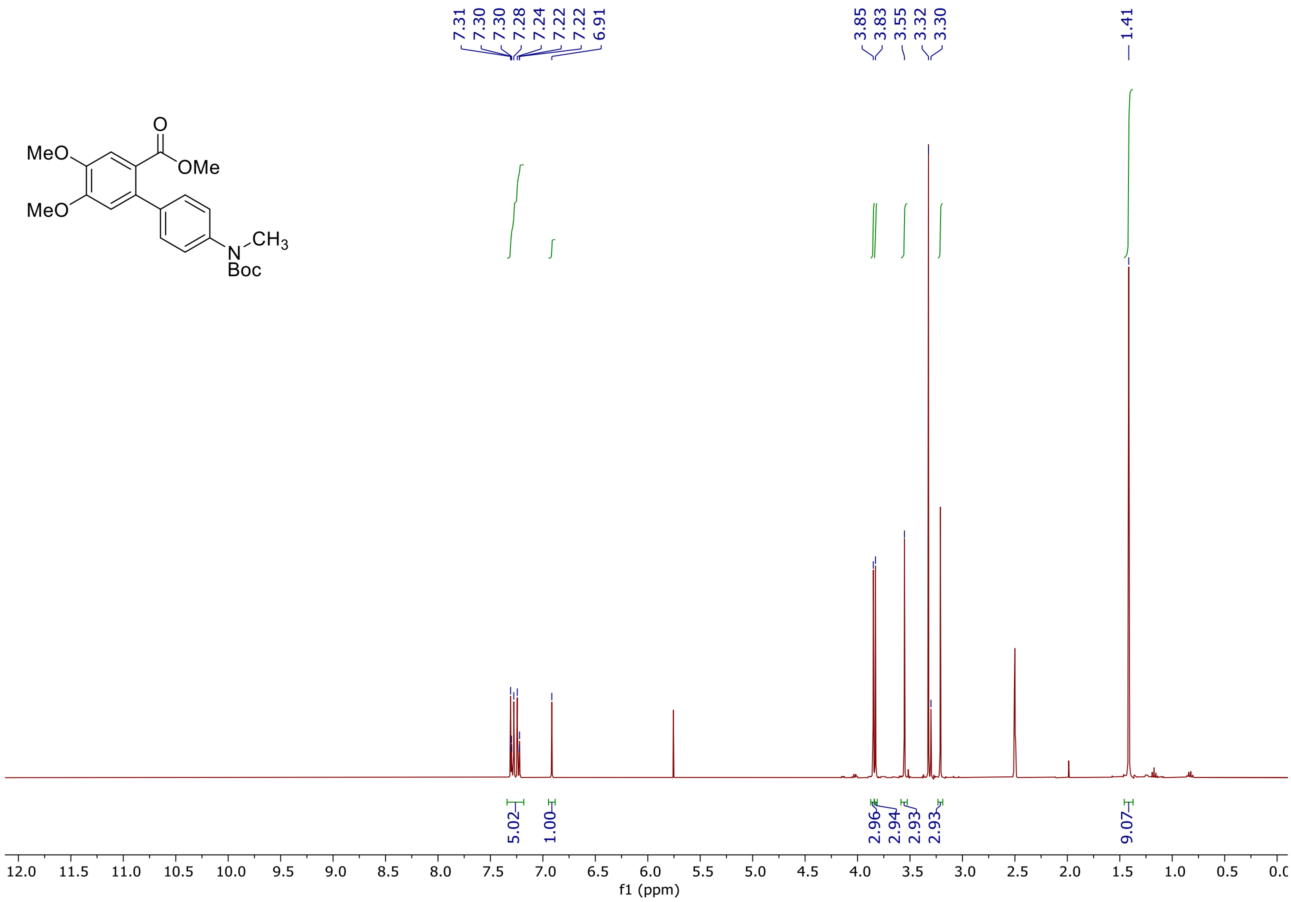

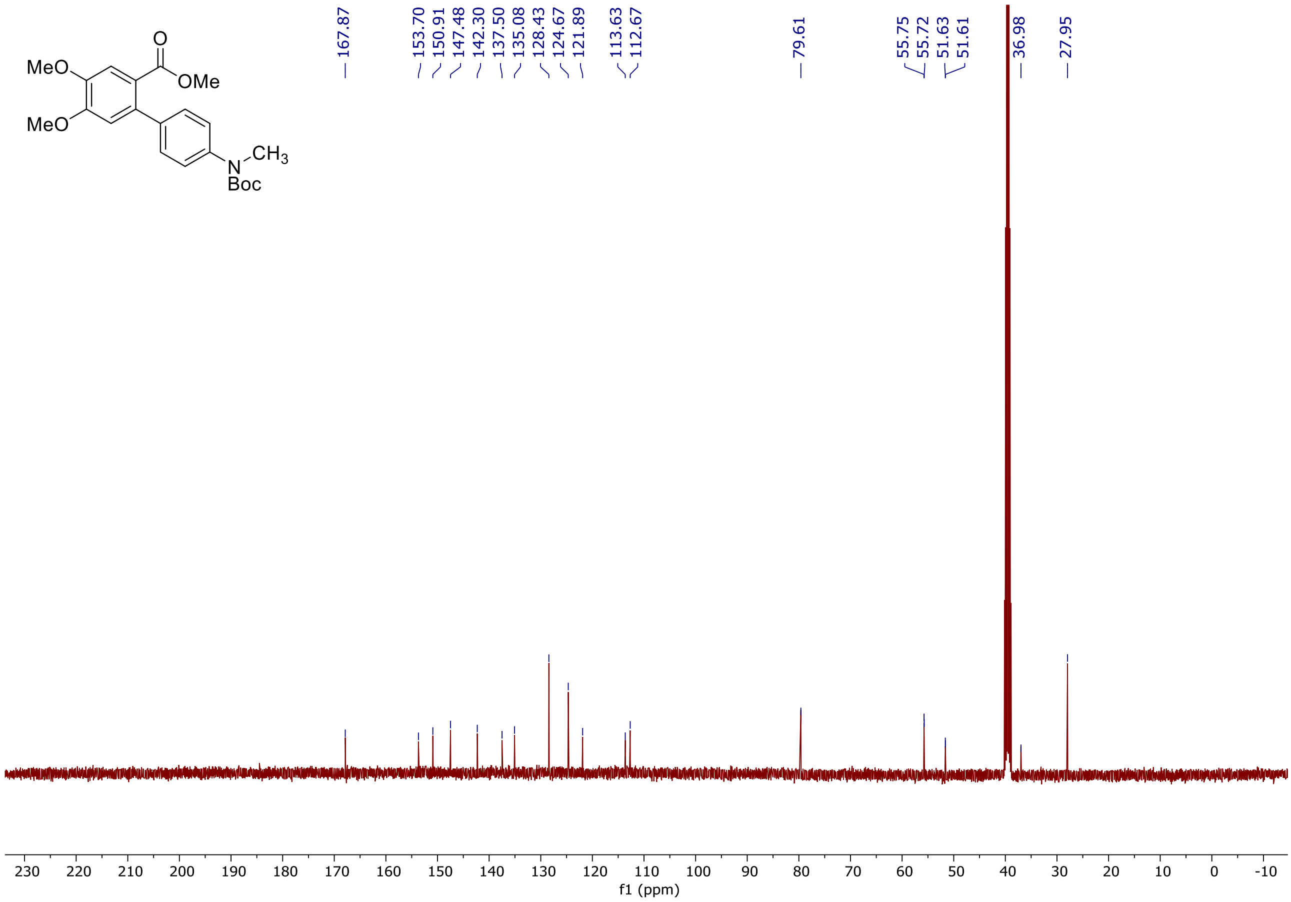

4'-((tert-butoxycarbonyl)(methyl)amino)-4,5-dimethoxy-[1,1'-biphenyl]-2-carboxylic acid (**22**)

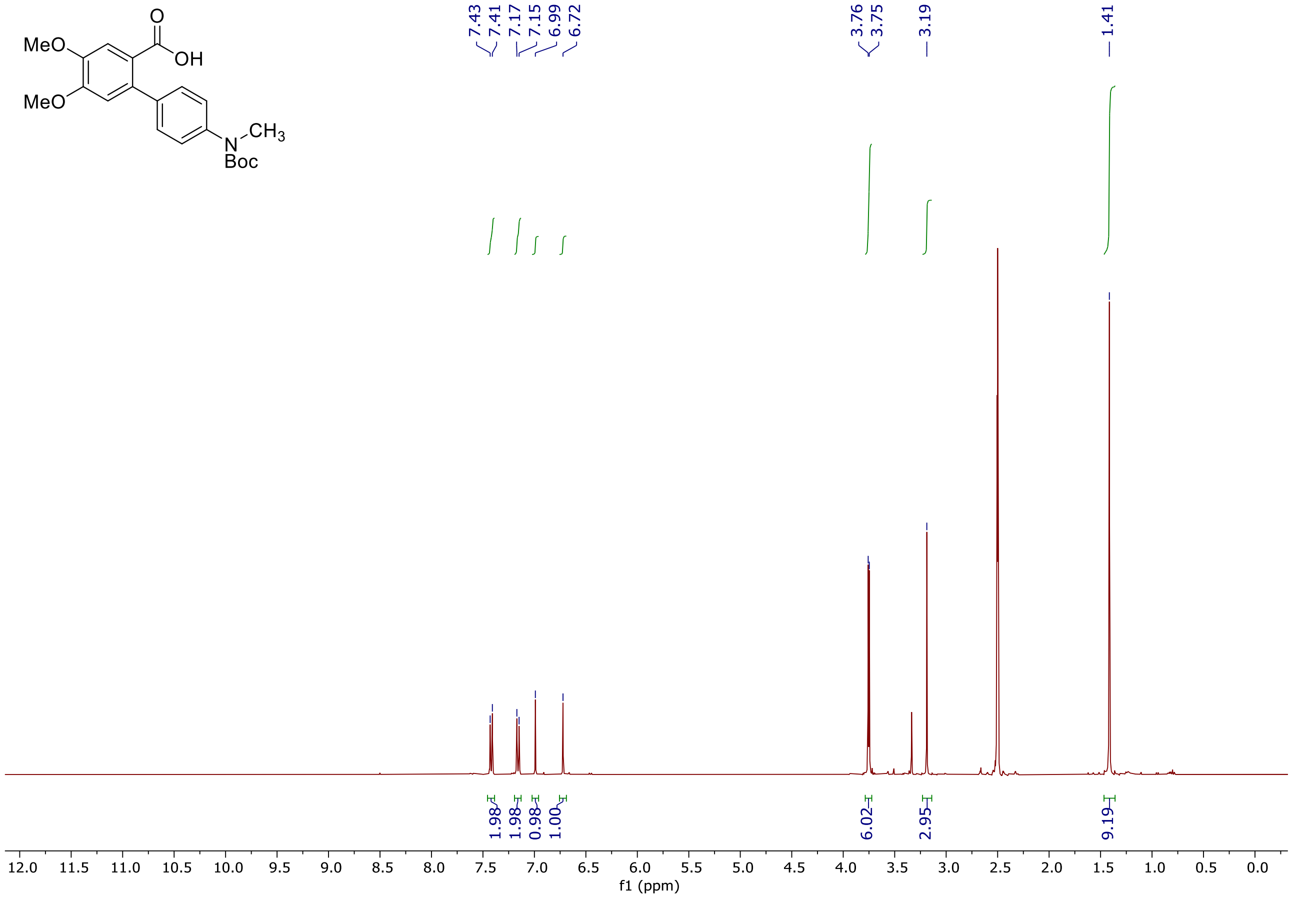

Tert-butyl (8,9-dimethoxy-6-oxo-6H-benzo[c]chromen-3-yl)(methyl)carbamate (**24**)

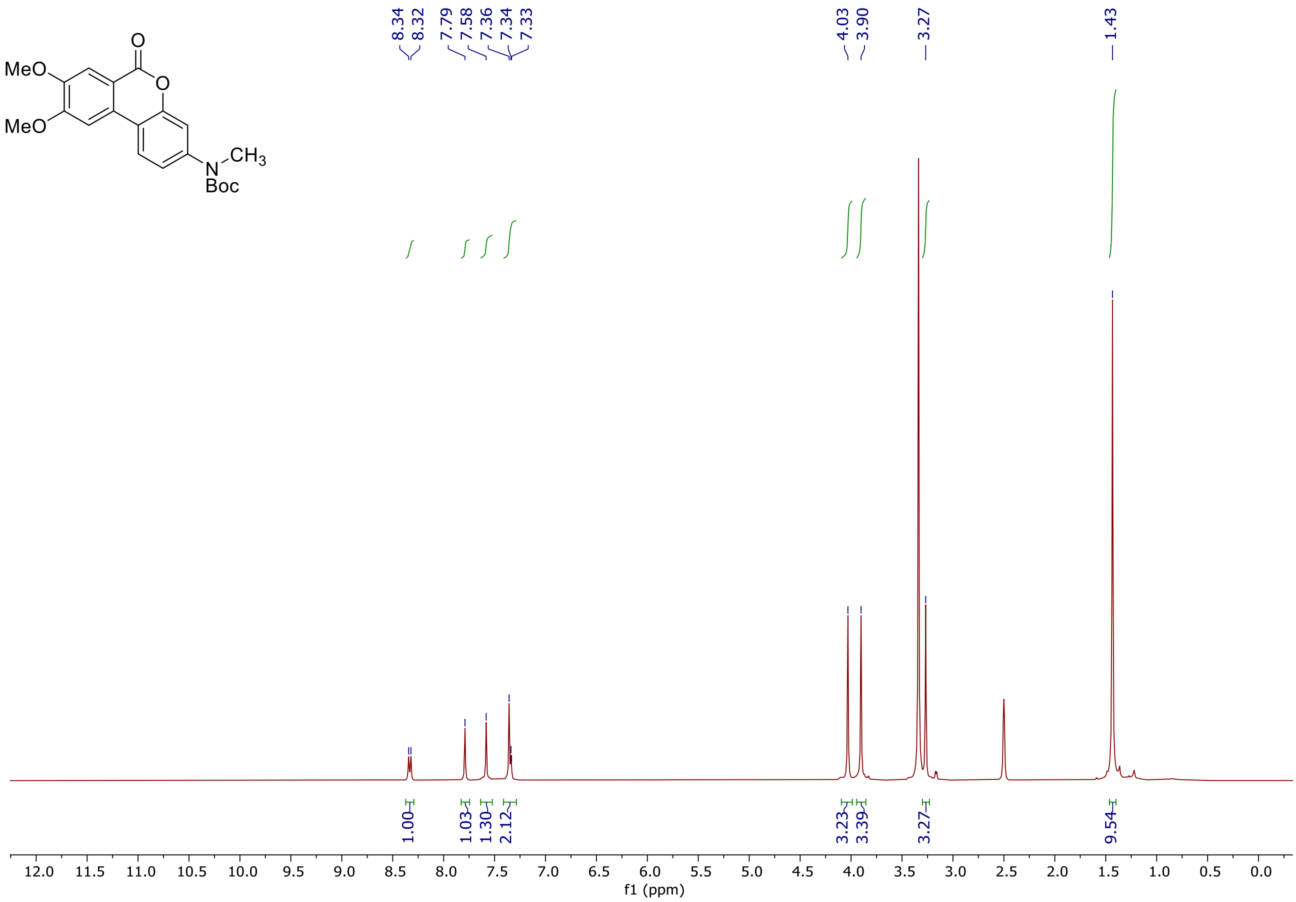

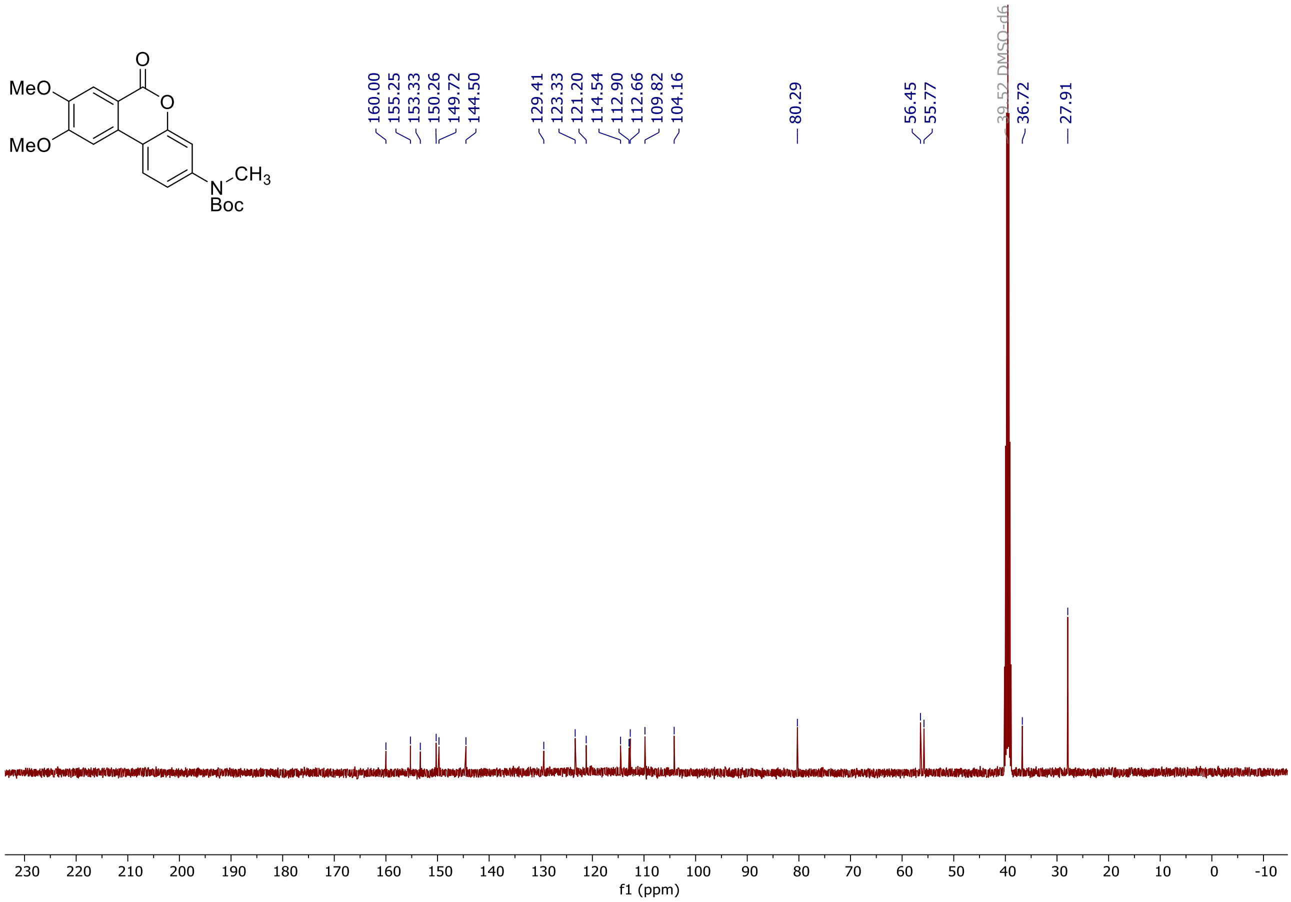

8,9-dihydroxy-3-(methylamino)-6H-benzo[c]chromen-6-one (**26**)

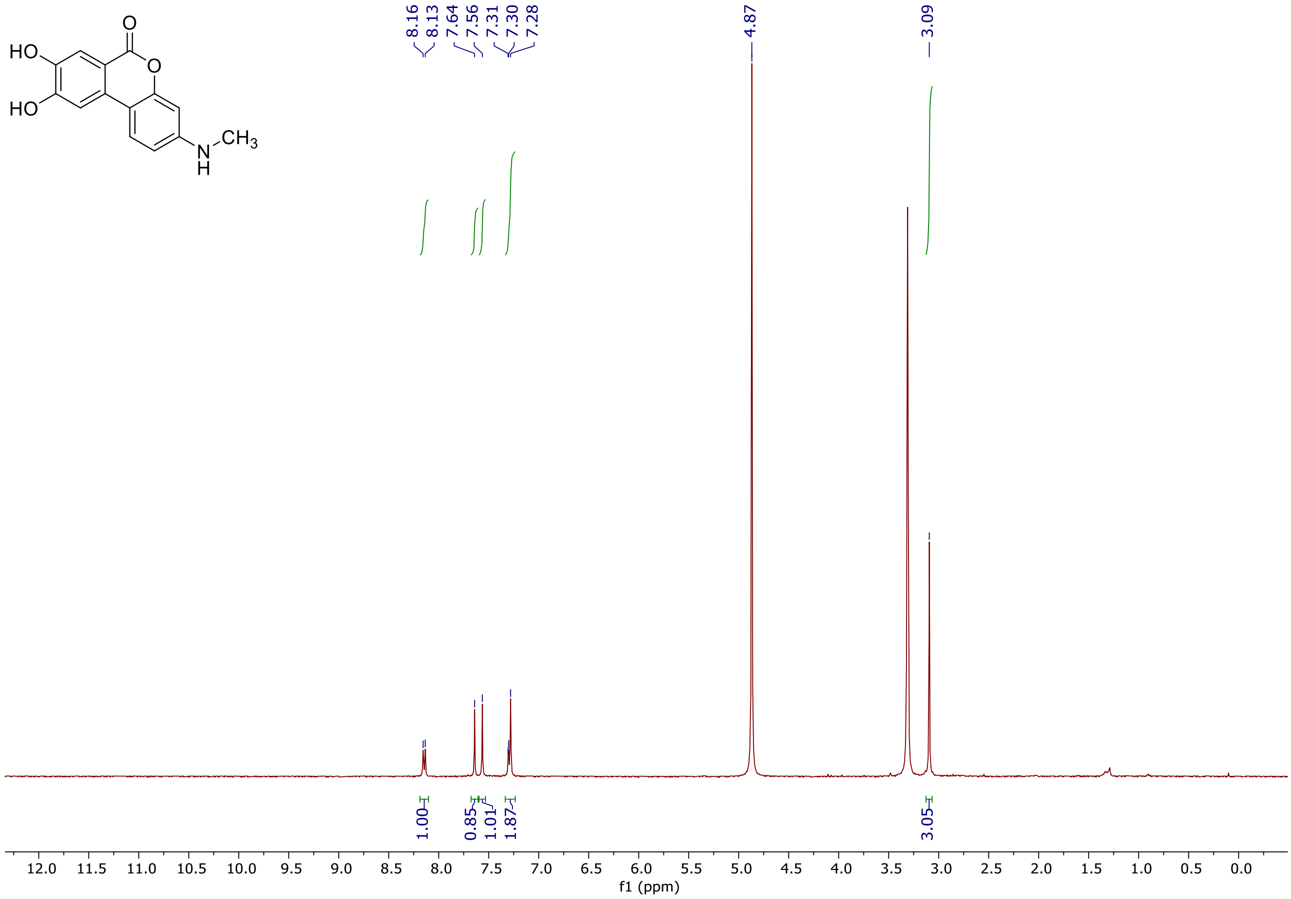

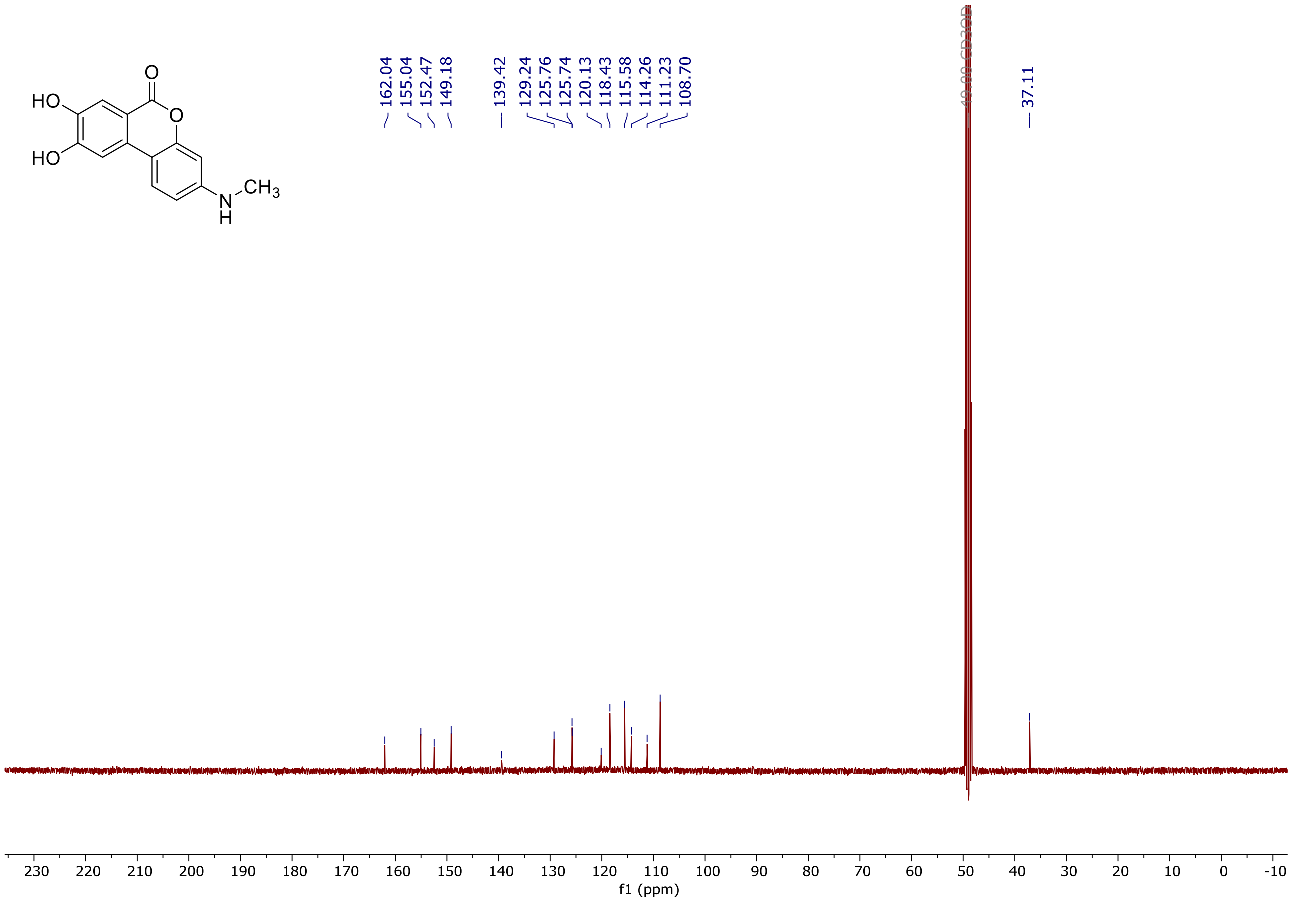

Methyl 4,5-dimethoxy-4'-nitro-[1,1'-biphenyl]-2-carboxylate (**21**)

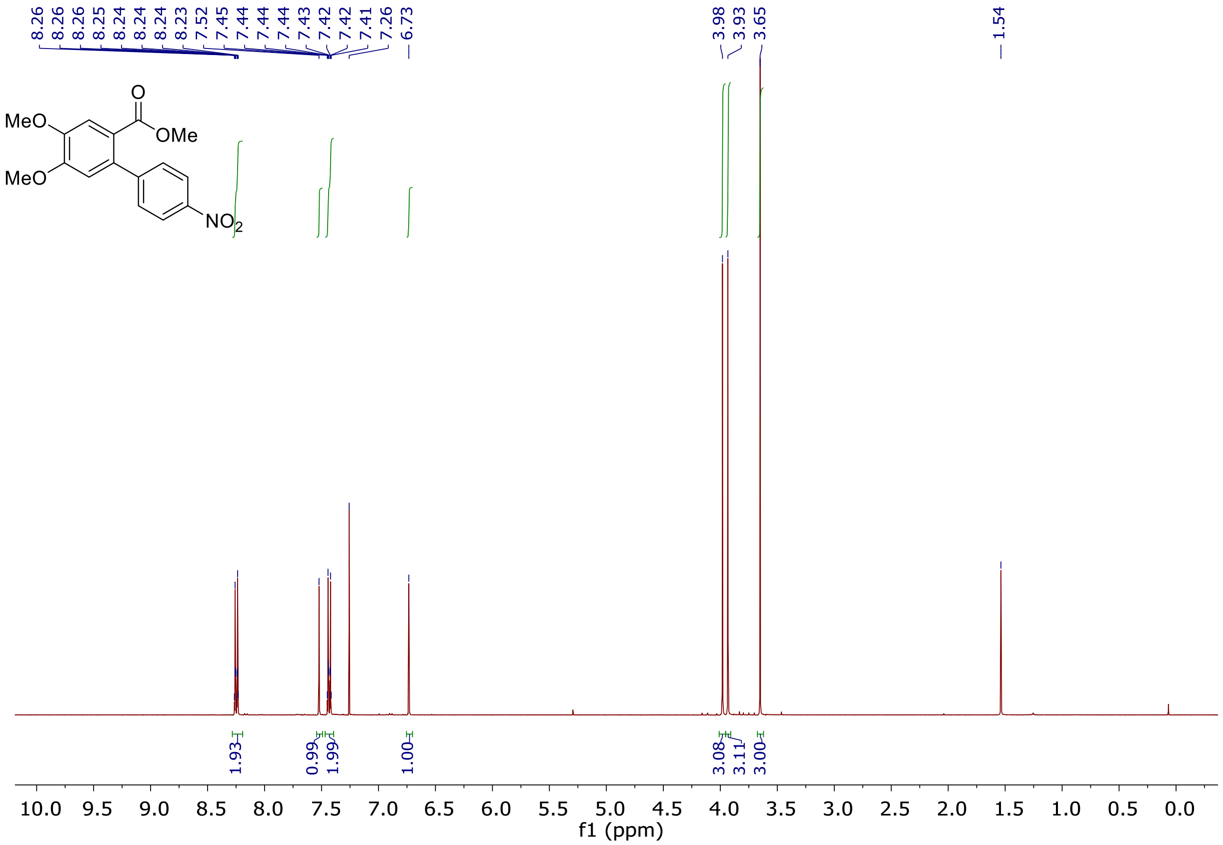

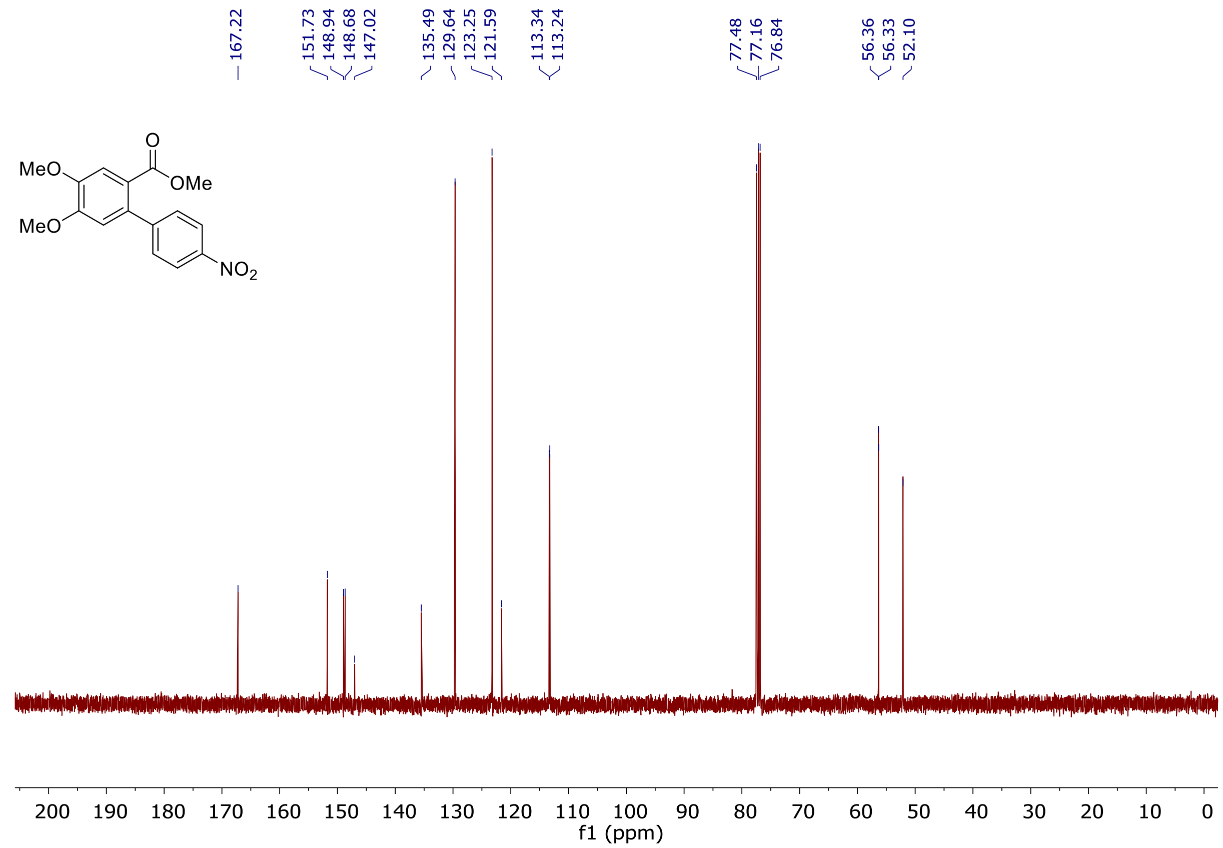

4,5-Dimethoxy-4'-nitro-[1,1'-biphenyl]-2-carboxylic acid (**23**)

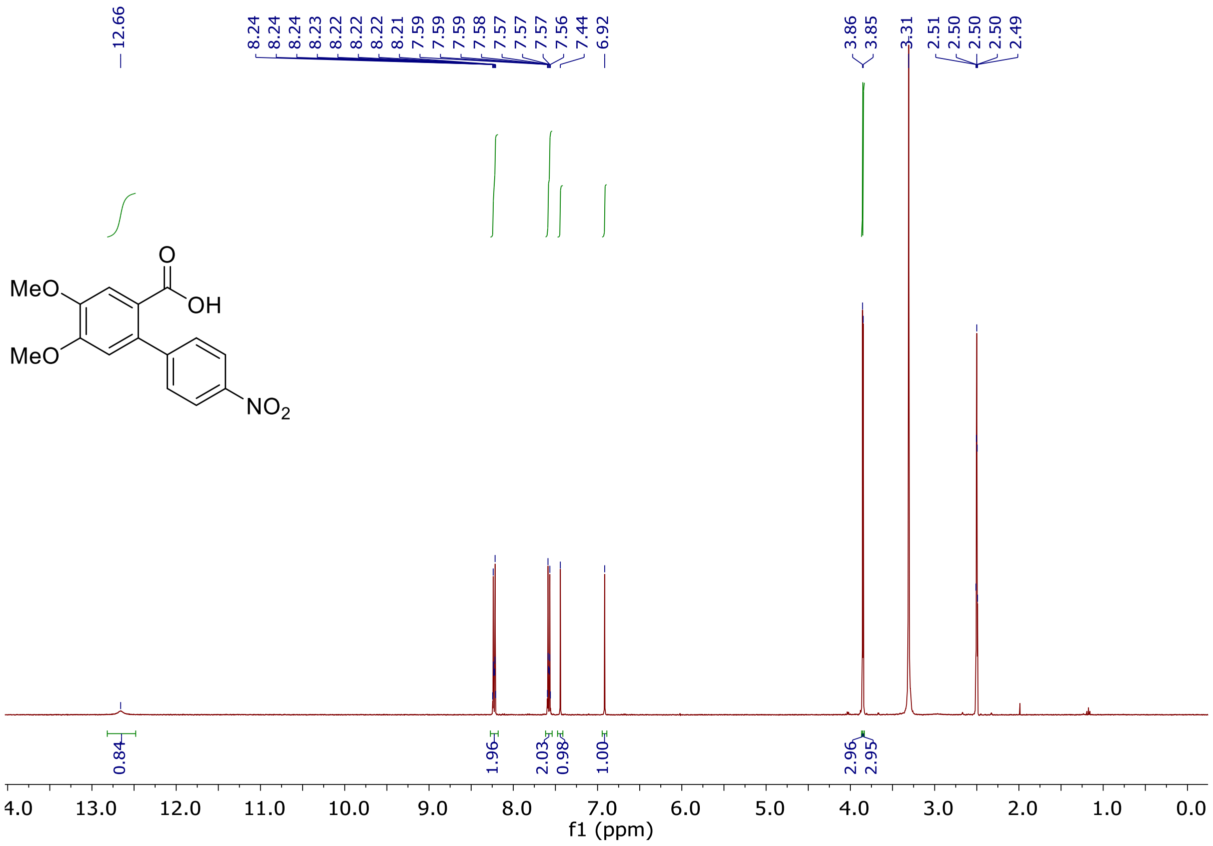

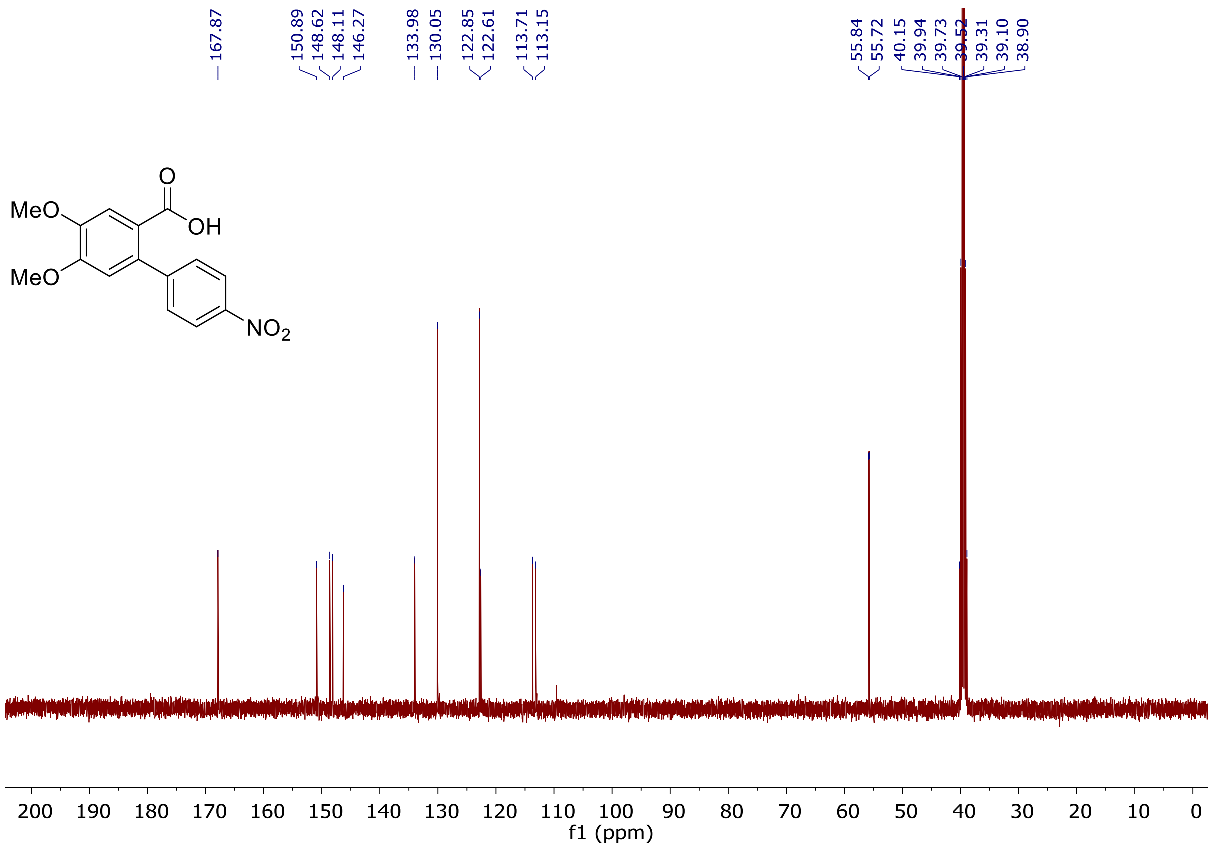

8,9-Dimethoxy-3-nitro-6H-benzo[c]chromen-6-one (**25**)

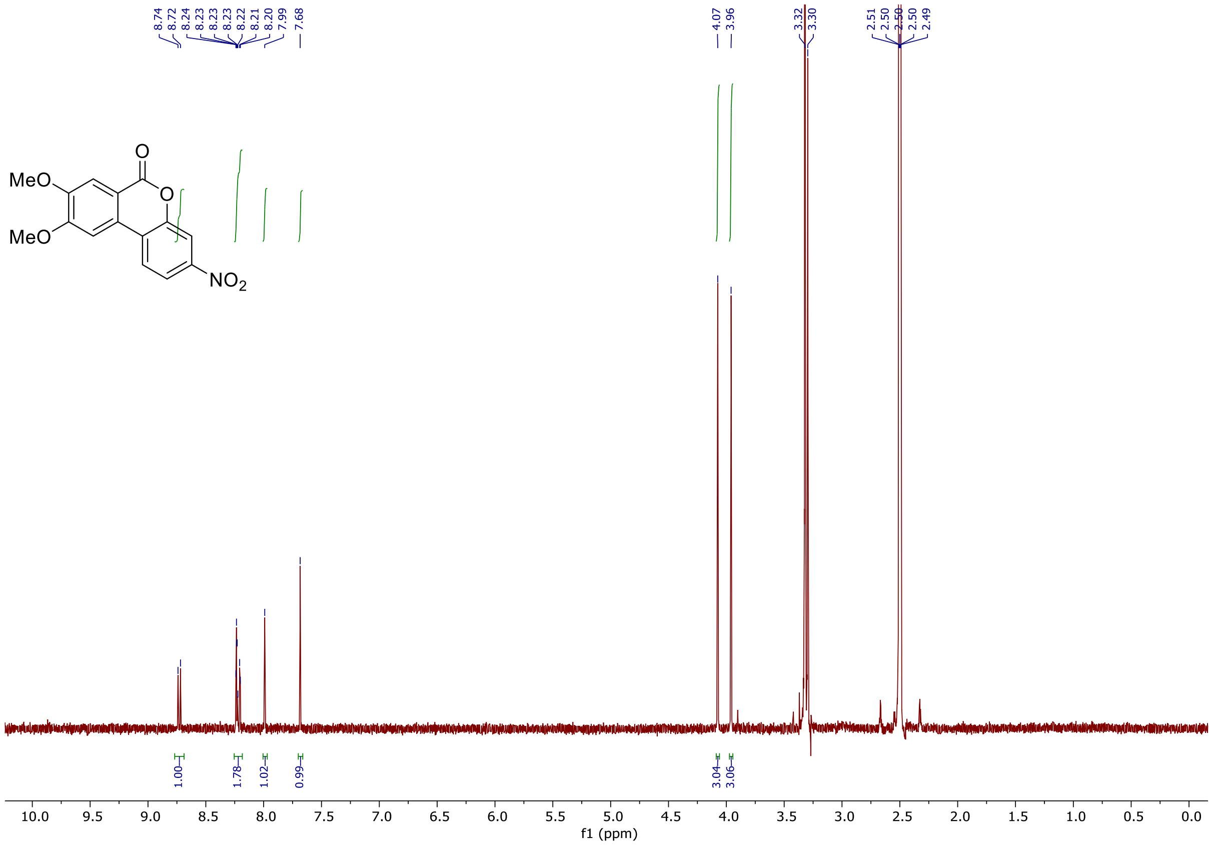

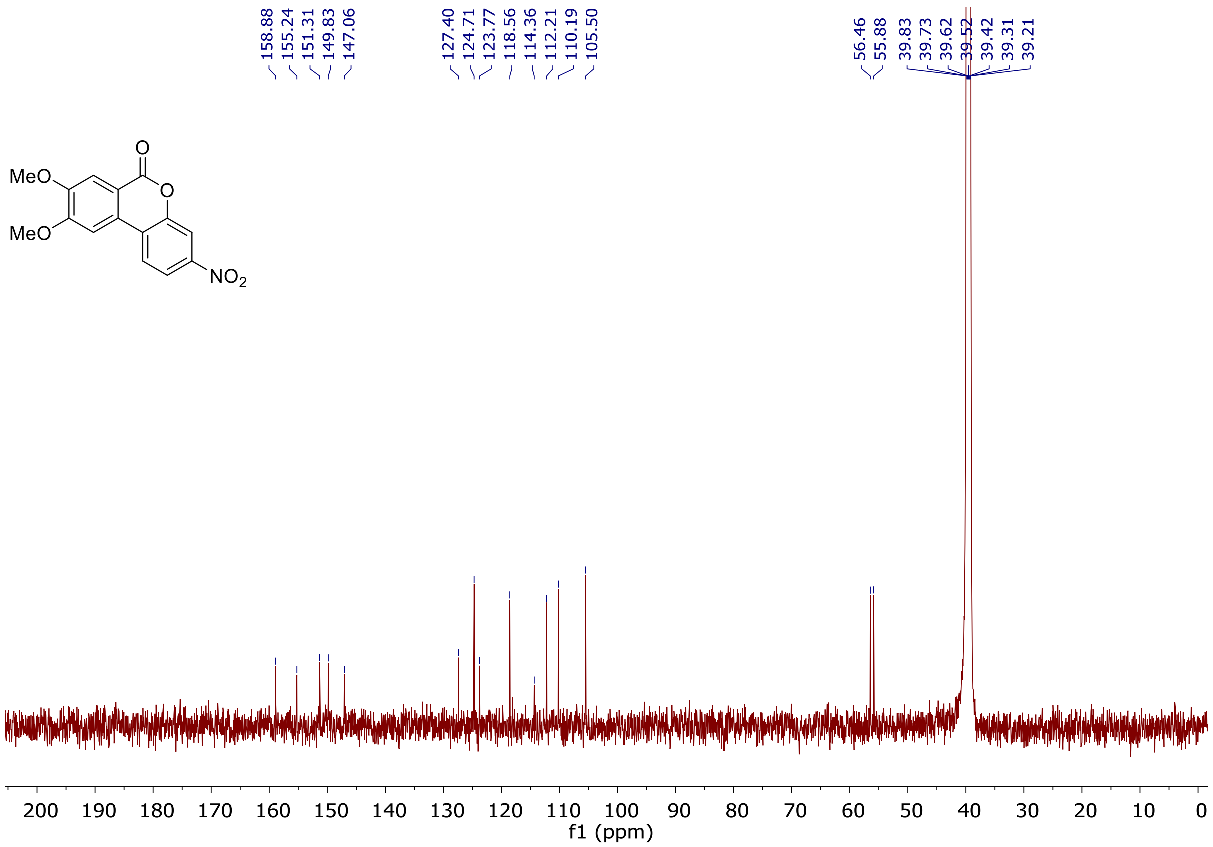

3-Amino-8,9-dimethoxy-6H-benzo[c]chromen-6-one (**27**)

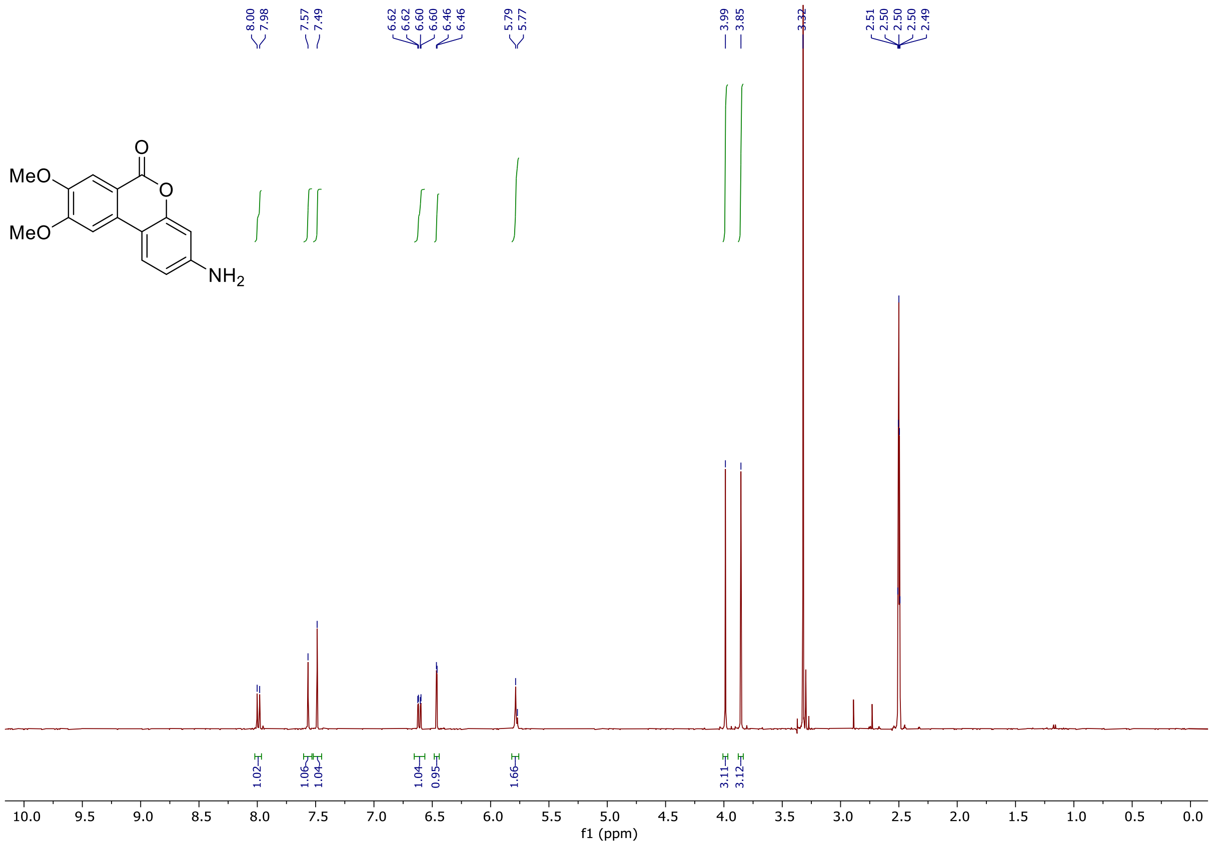

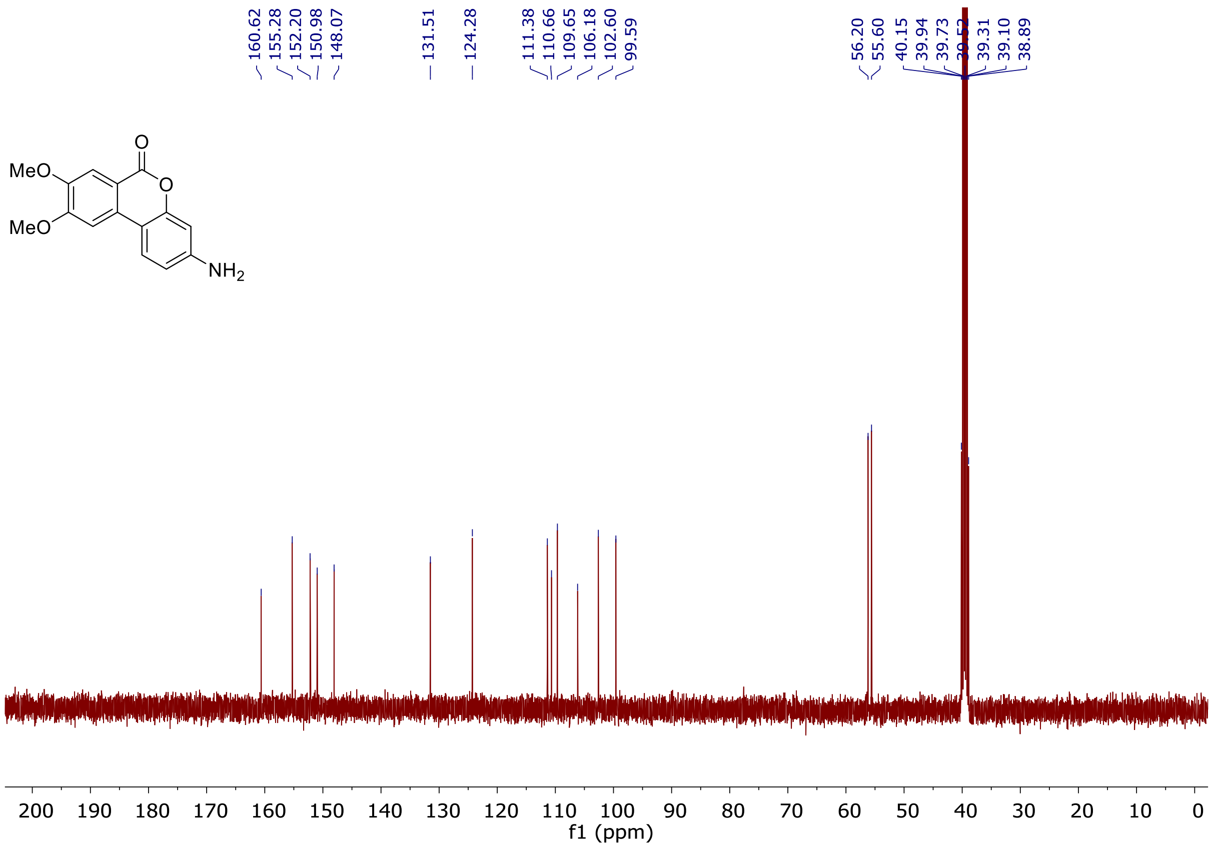

3-Amino-8,9-dihydroxy-6H-benzo[c]chromen-6-one (**28**)

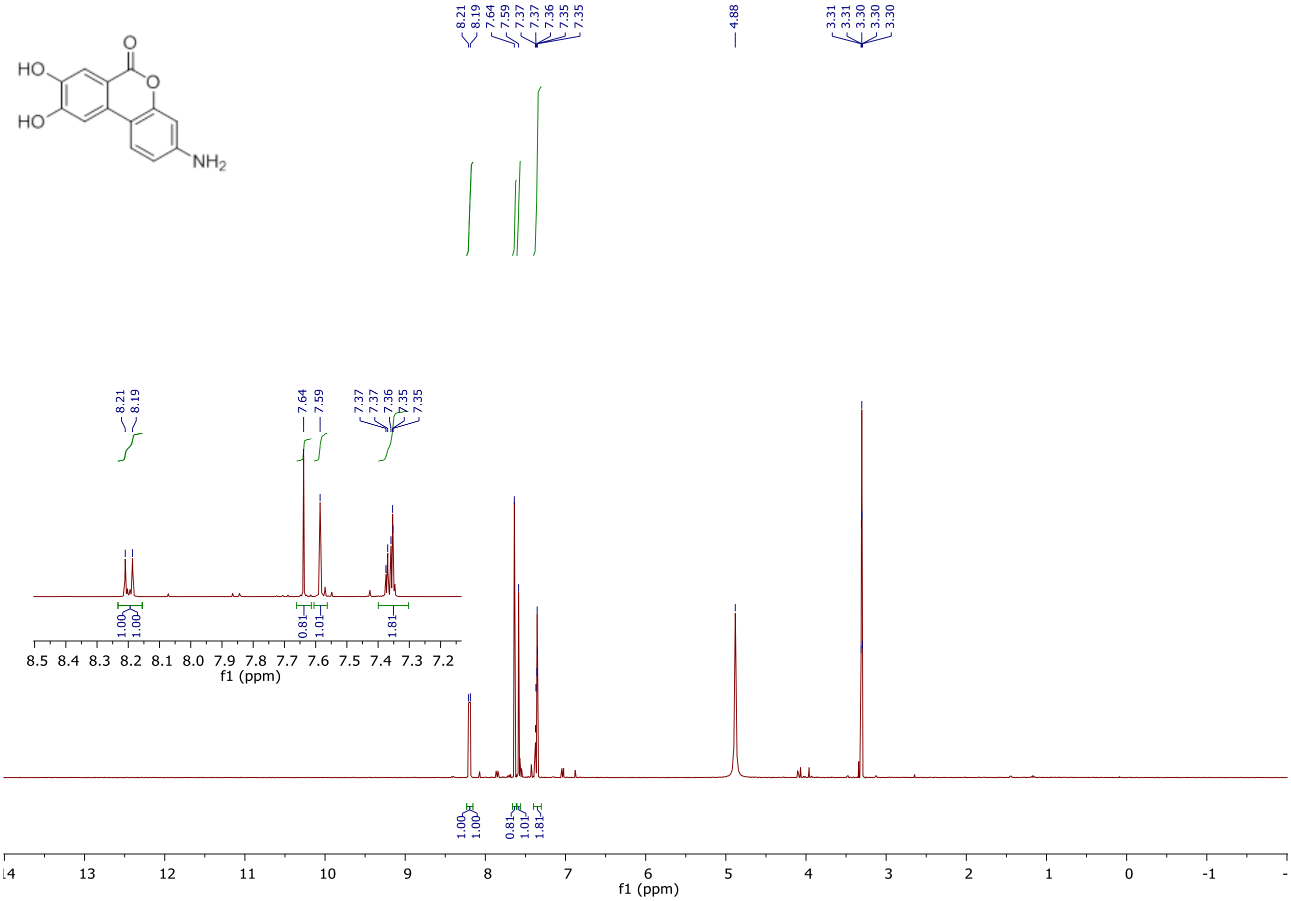

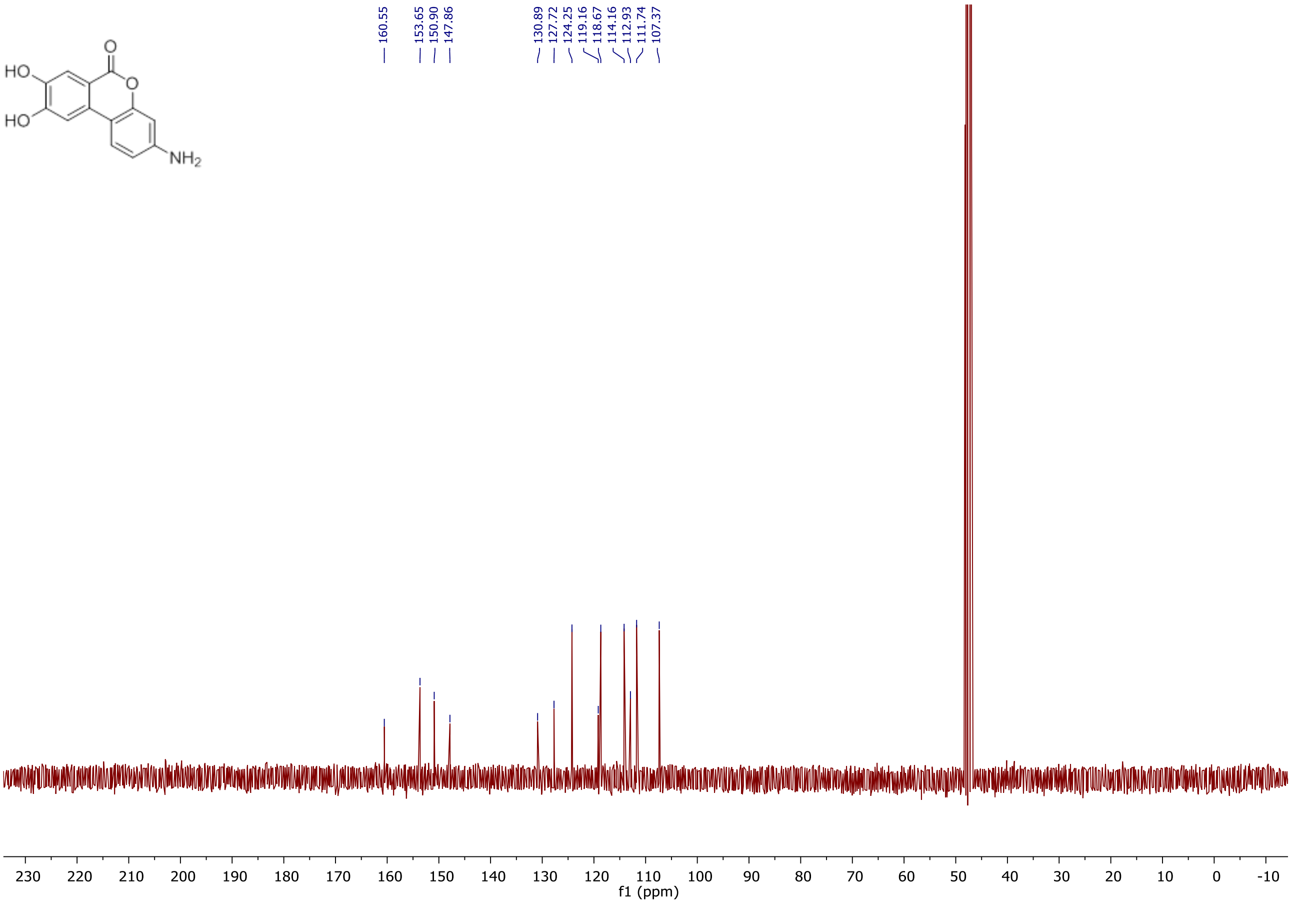

N-(8,9-dimethoxy-6-oxo-6H-benzo[c]chromen-3-yl)acetamide (**29**)

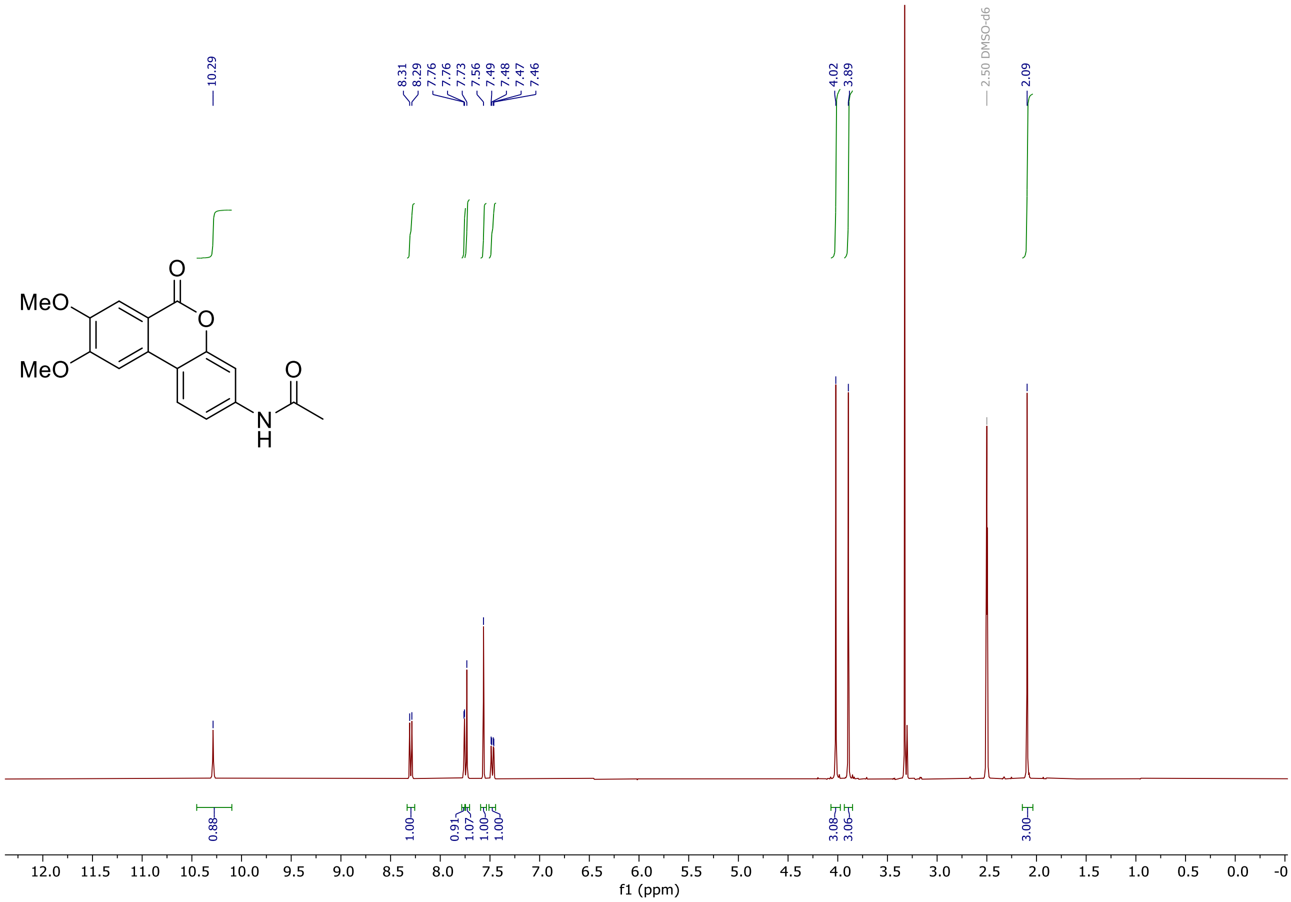

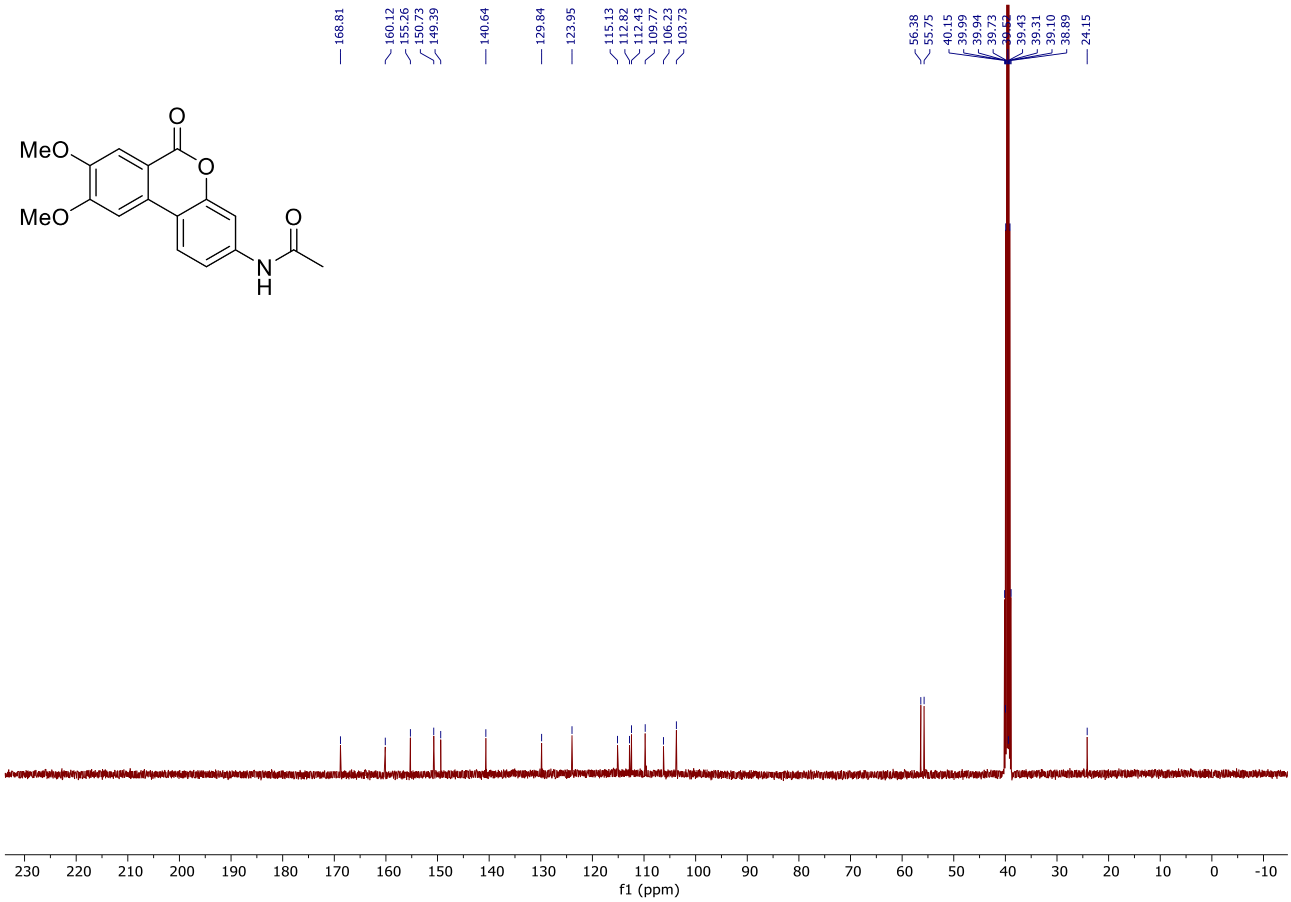

N-(8,9-dihydroxy-6-oxo-6H-benzo[c]chromen-3-yl)acetamide (**33**)

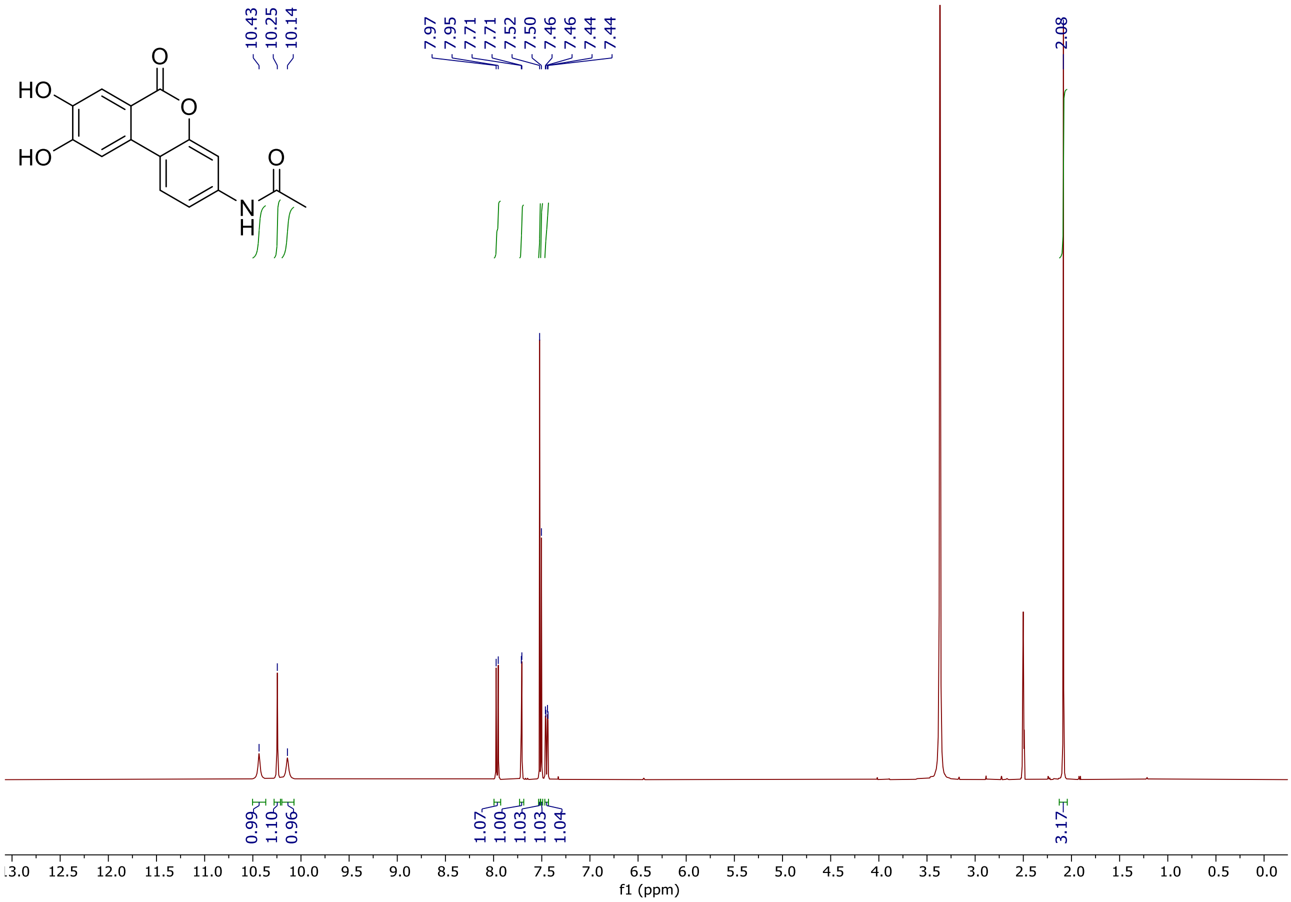

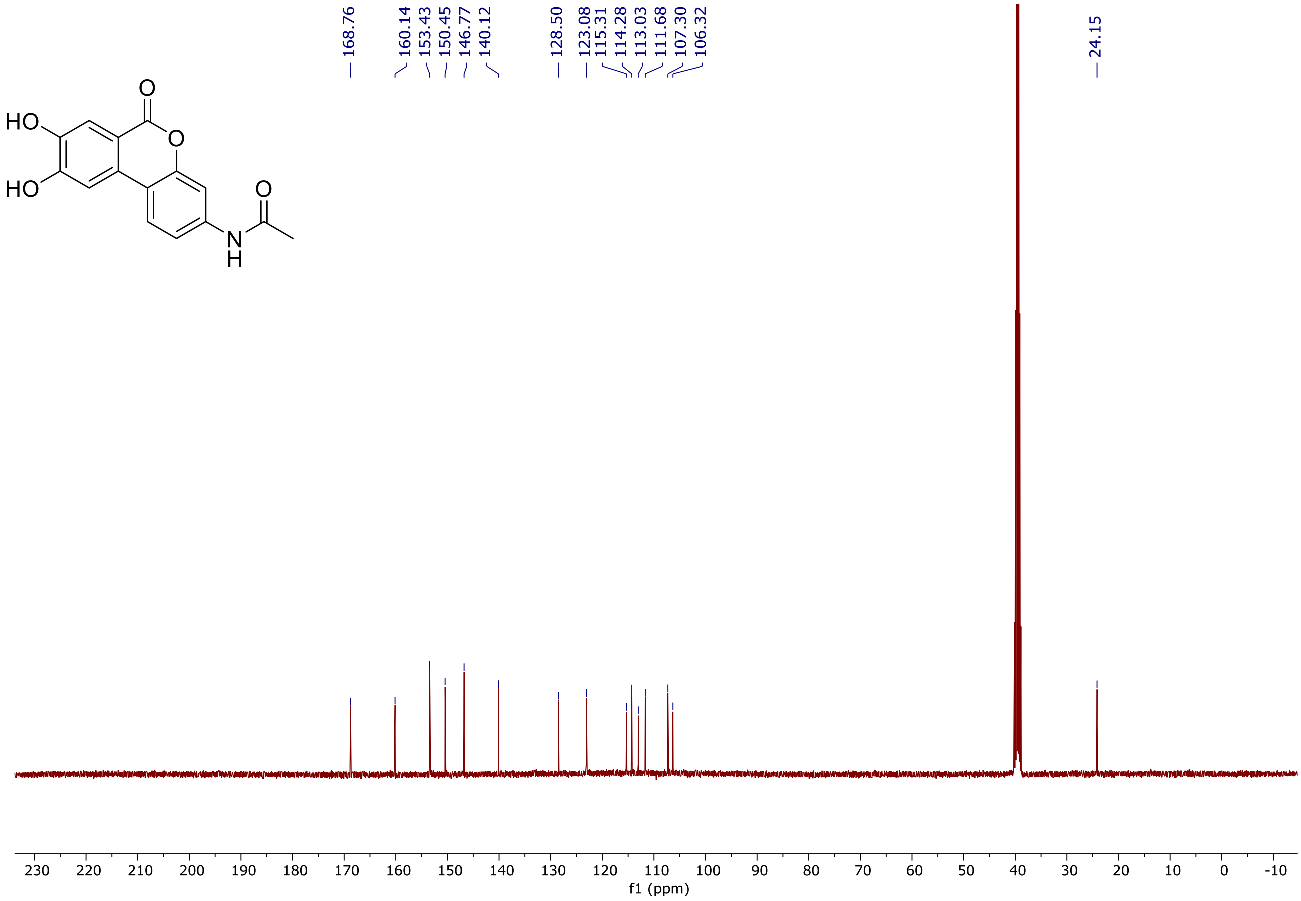

N-(8,9-dimethoxy-6-oxo-6H-benzo[c]chromen-3-yl)propionamide (**30**)

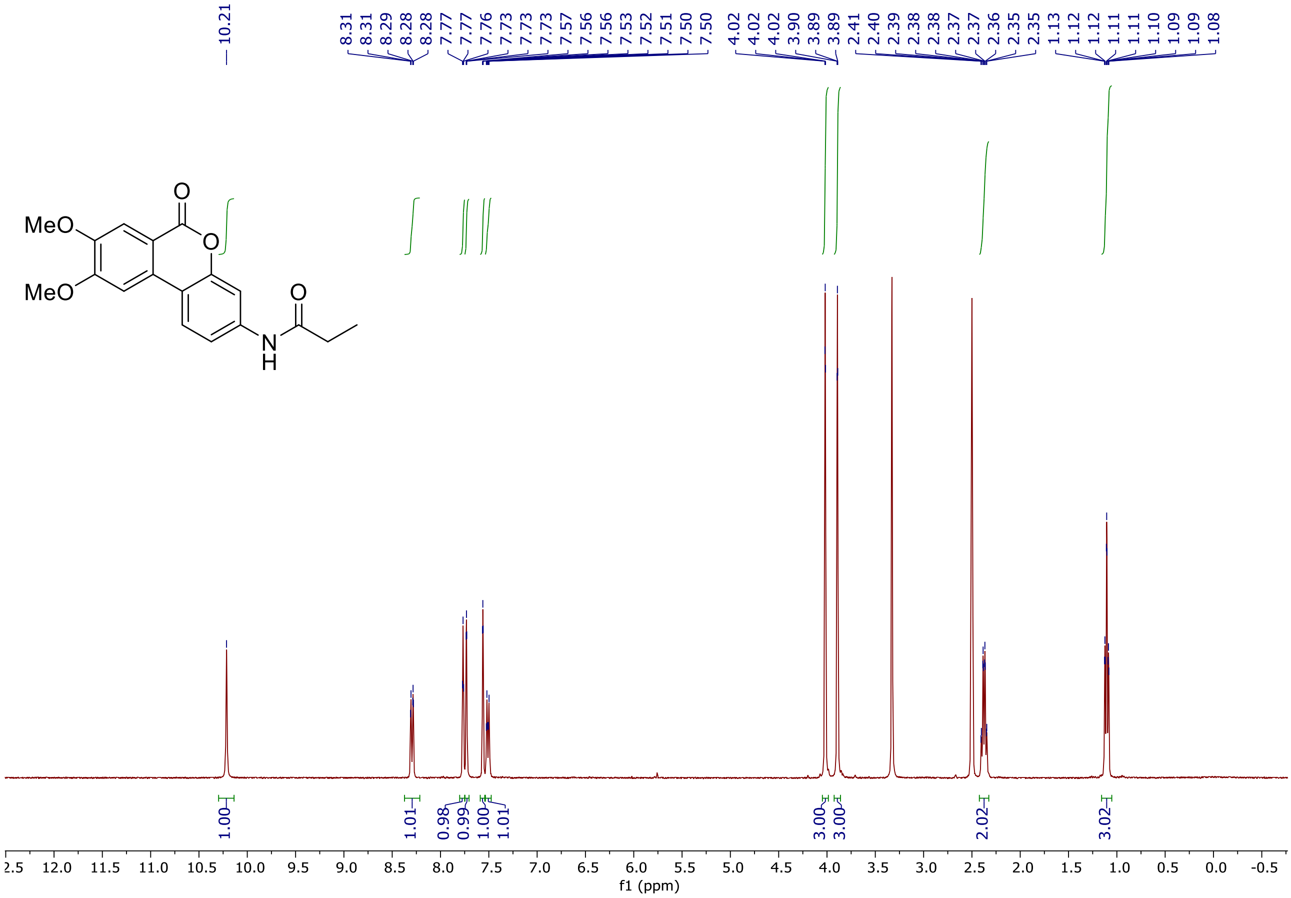

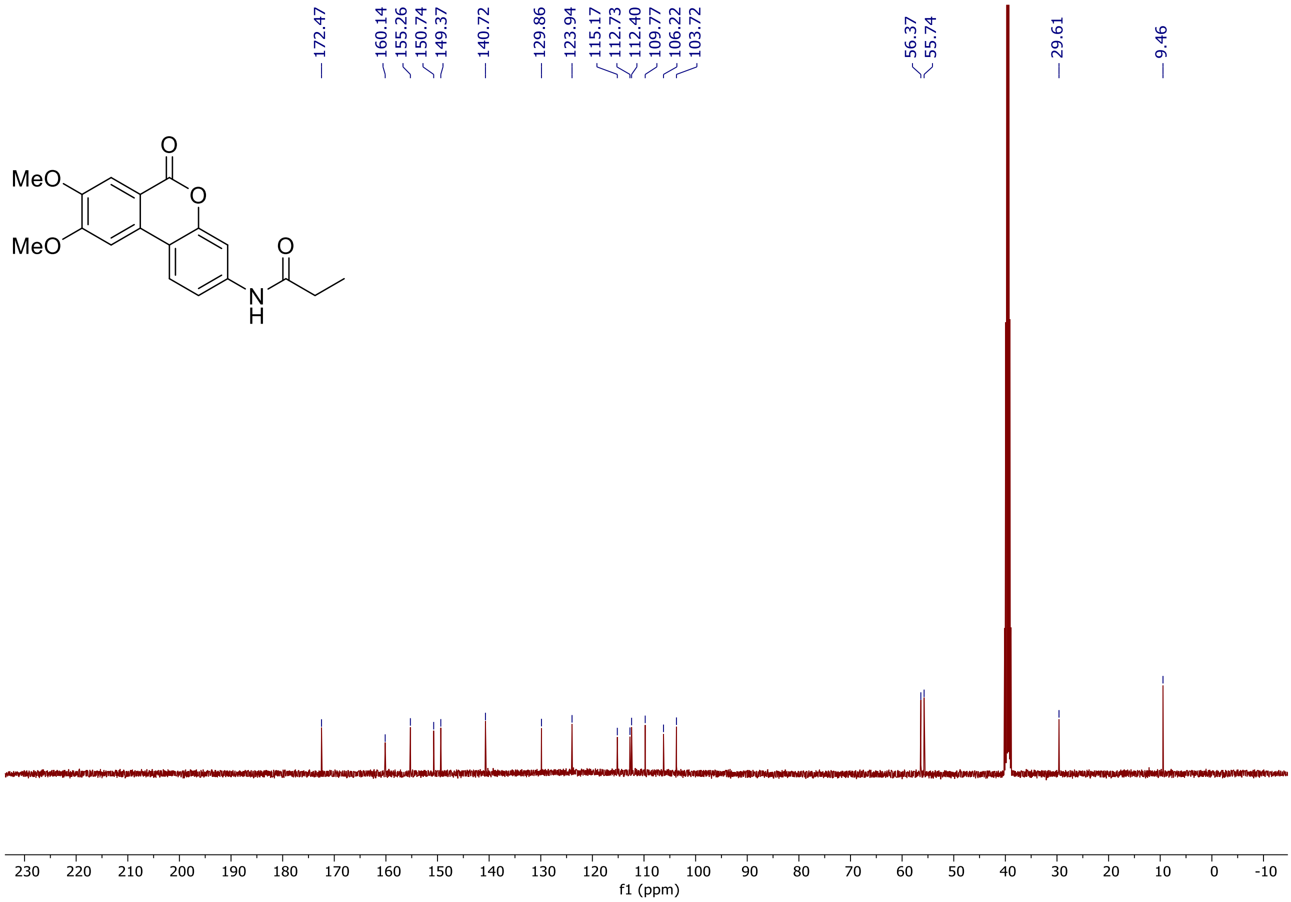

N-(8,9-dihydroxy-6-oxo-6H-benzo[c]chromen-3-yl)propionamide (**34**)

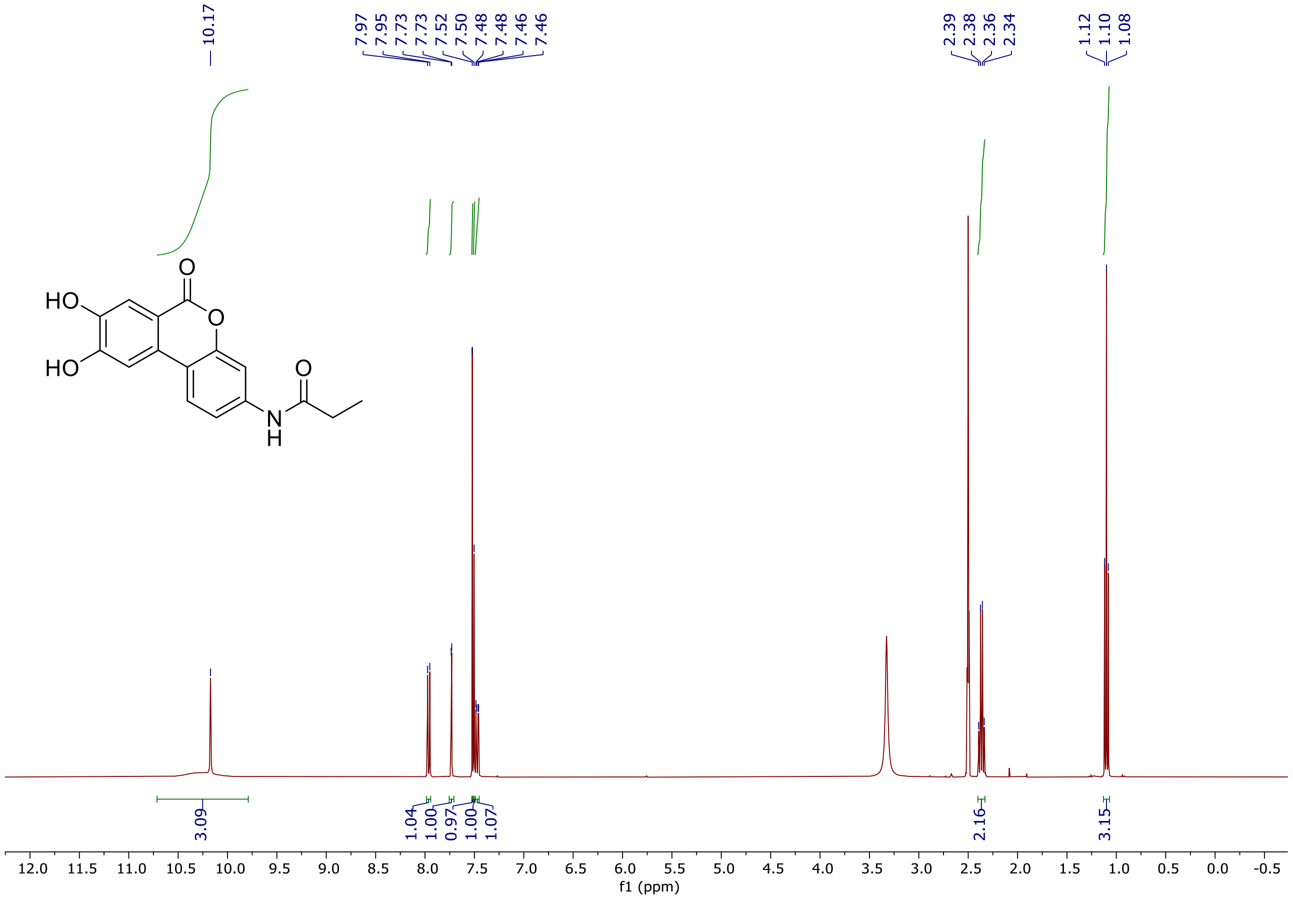

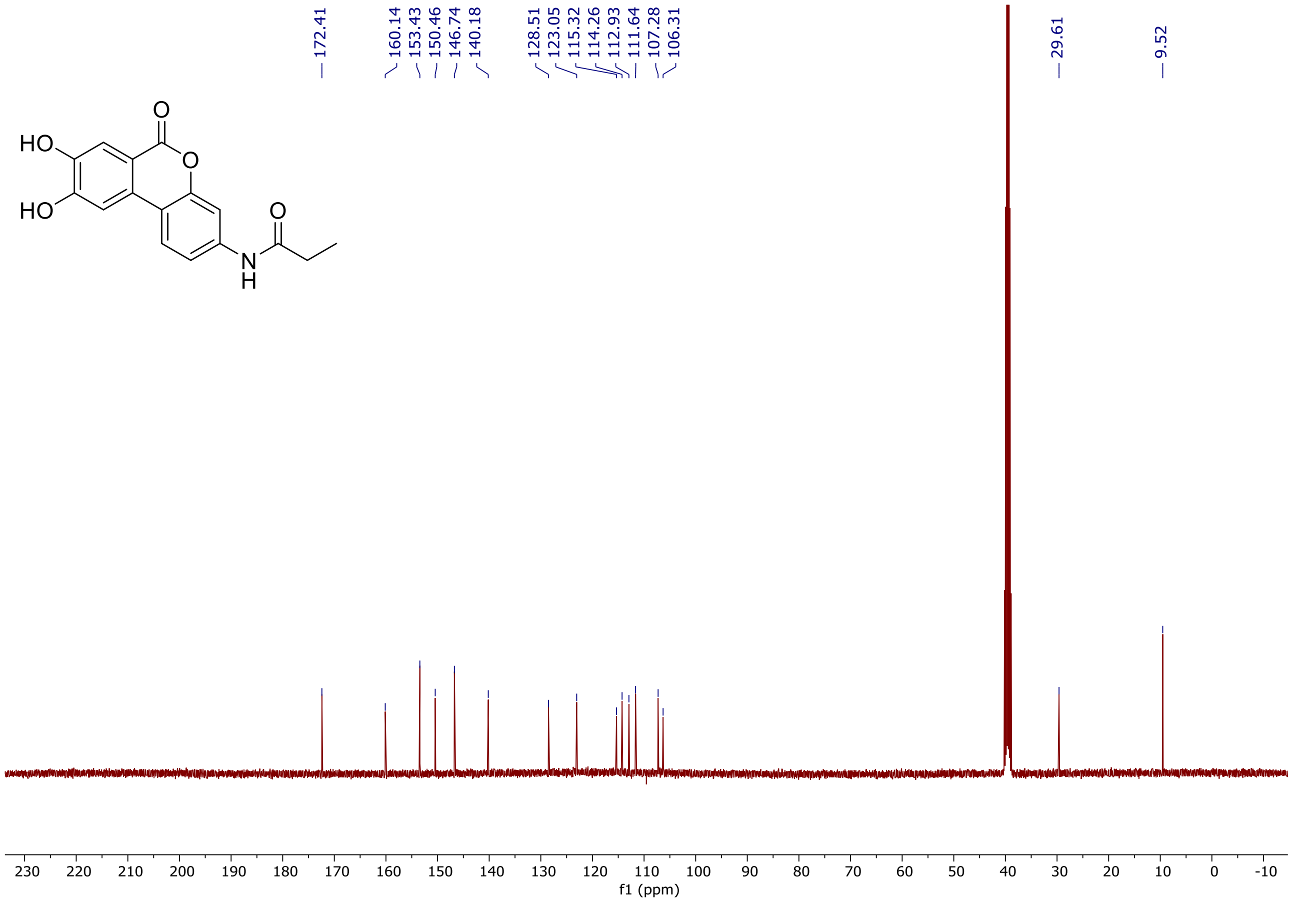

N-(8,9-dimethoxy-6-oxo-6H-benzo[c]chromen-3-yl)butyramide (**31**)

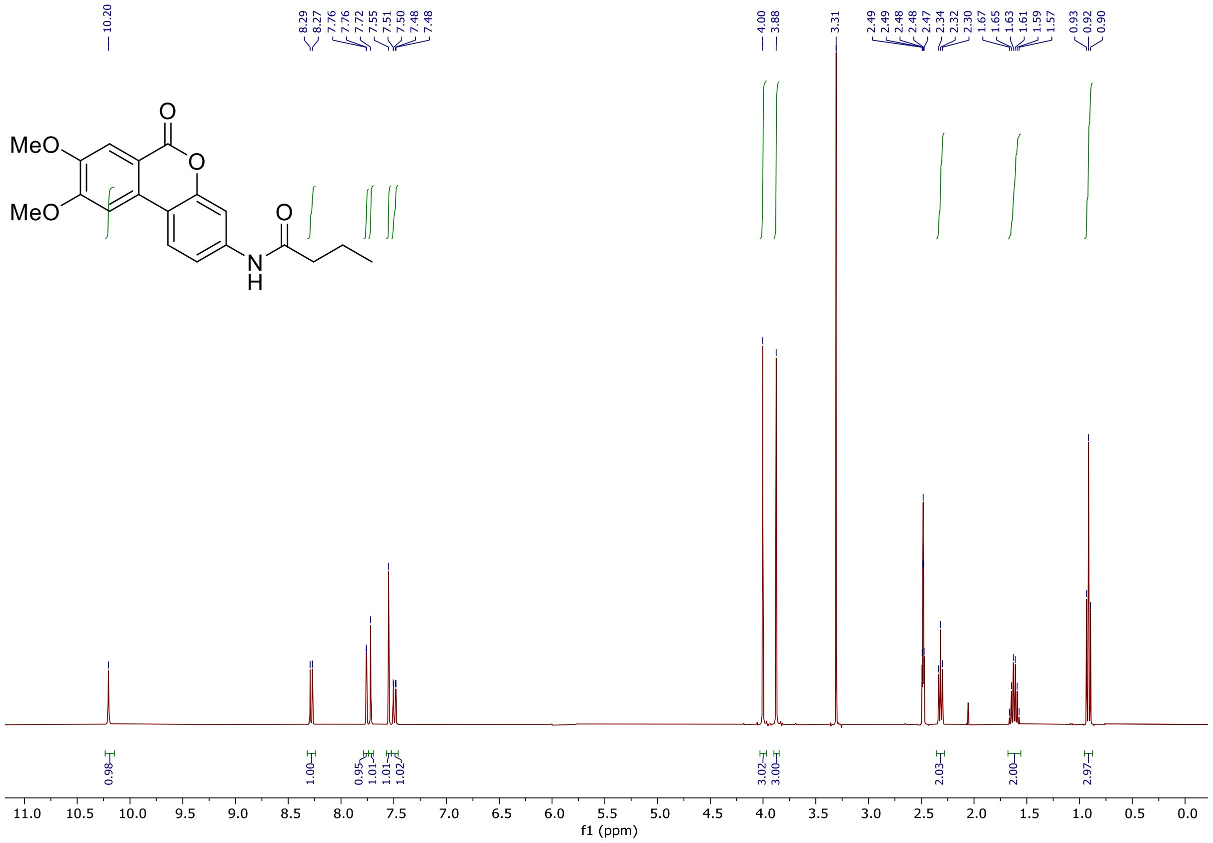

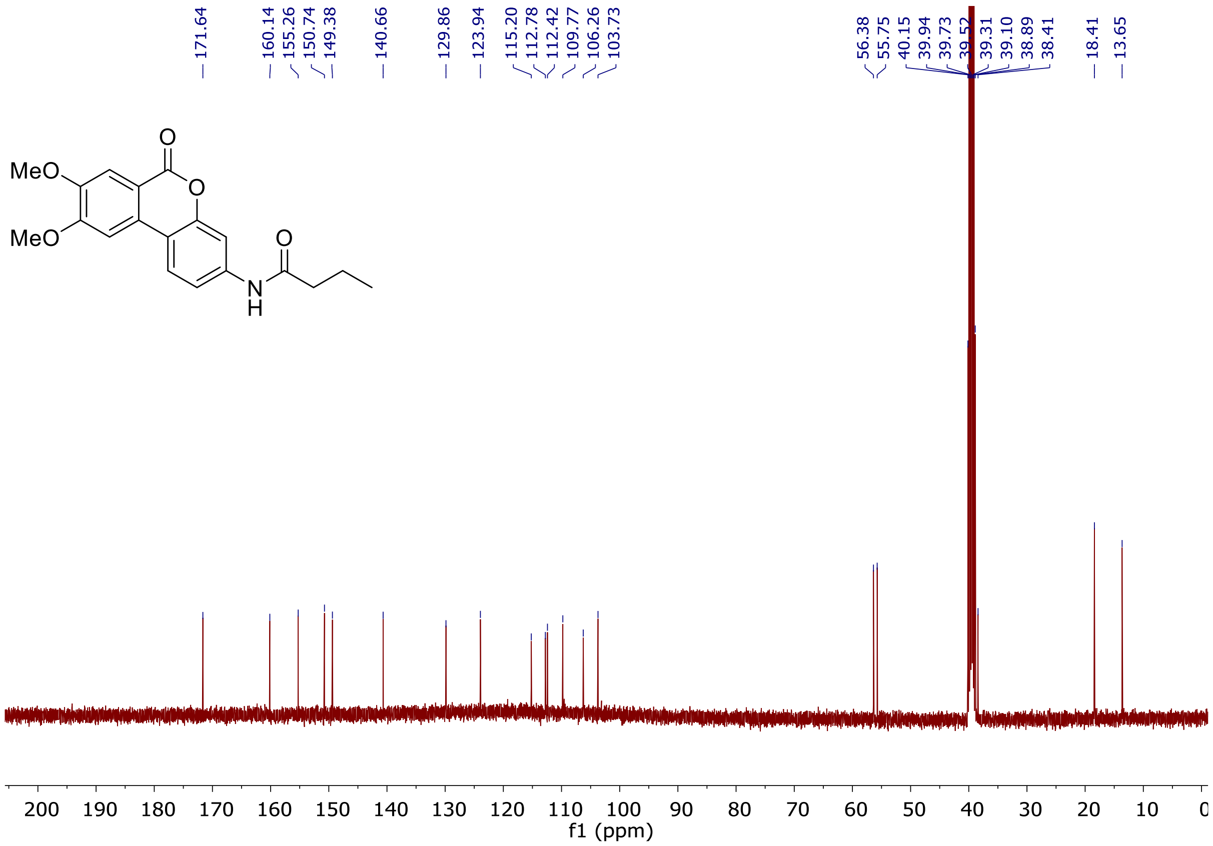

N-(8,9-dihydroxy-6-oxo-6H-benzo[c]chromen-3-yl)butyramide (**35**)

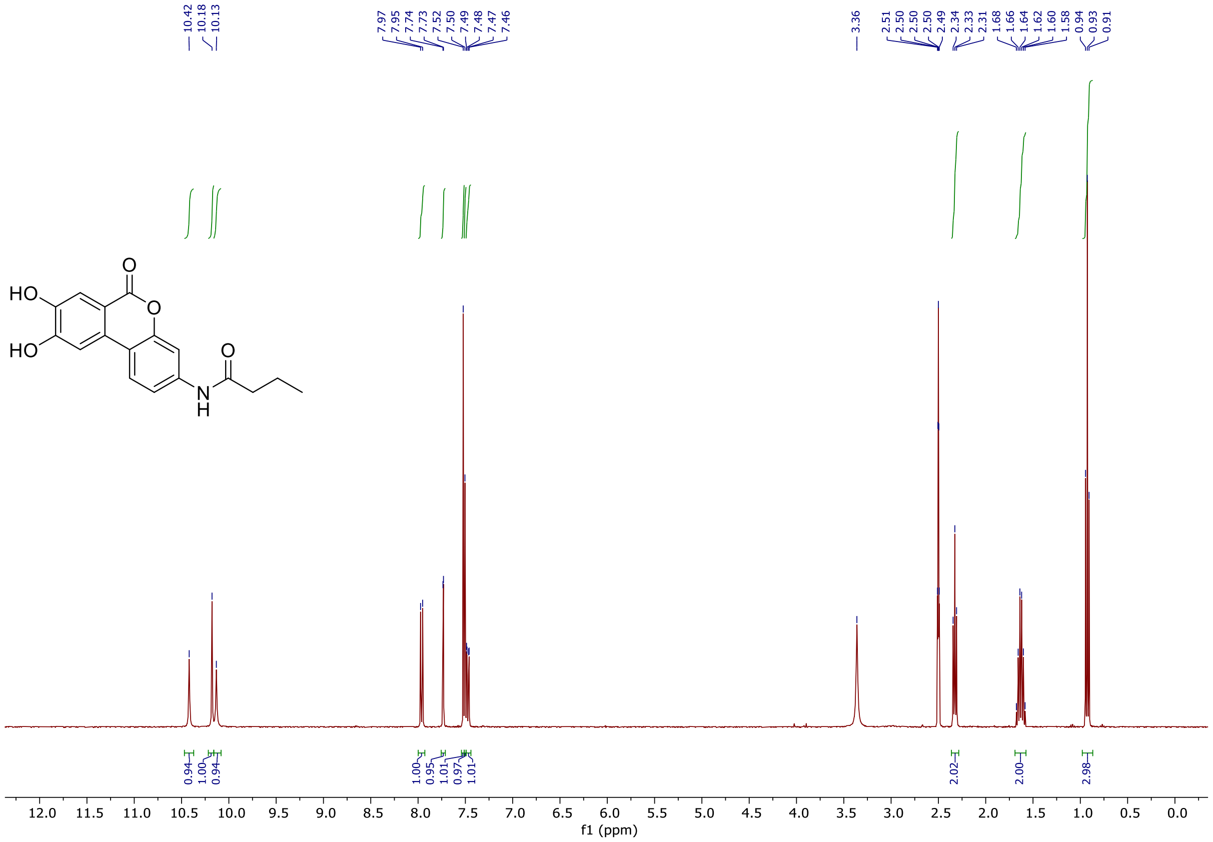

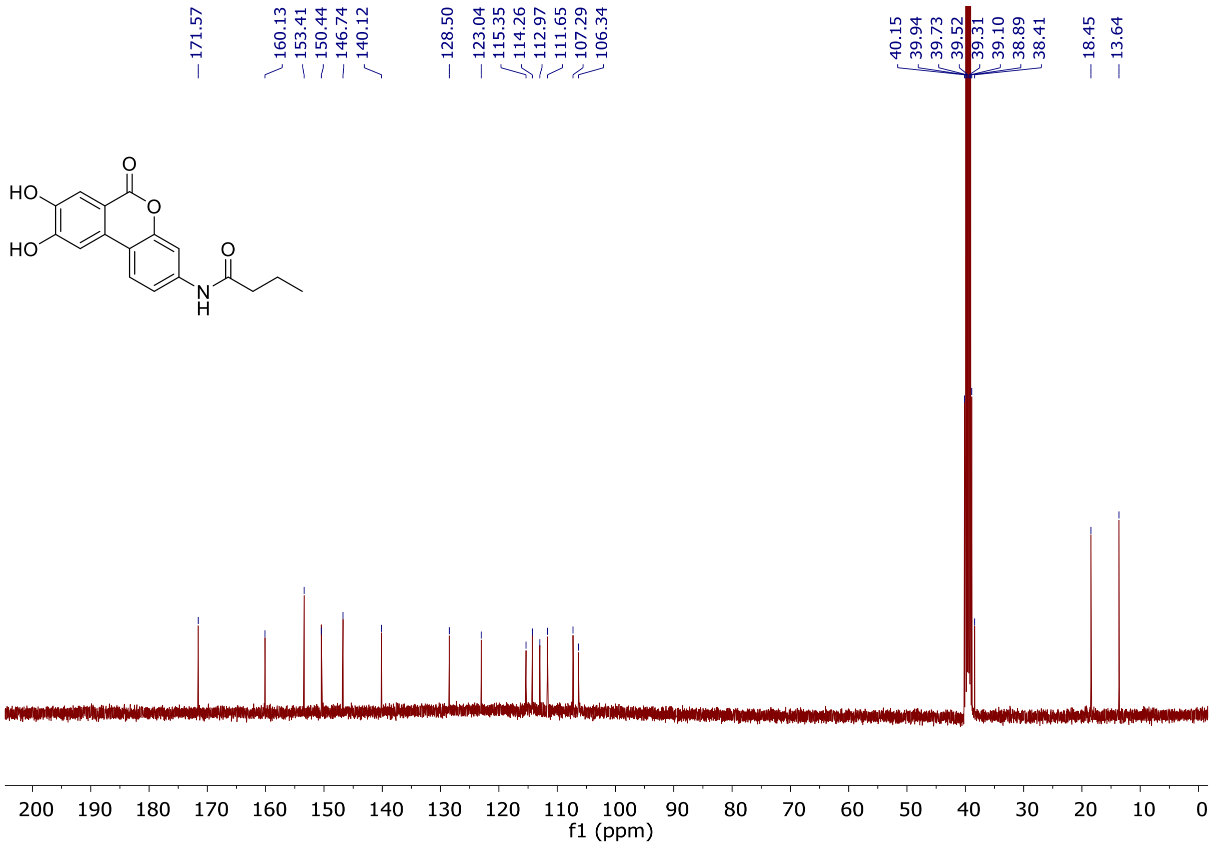

N-(8,9-Dimethoxy-6-oxo-6H-benzo[c]chromen-3-yl)methanesulfonamide (**32**)

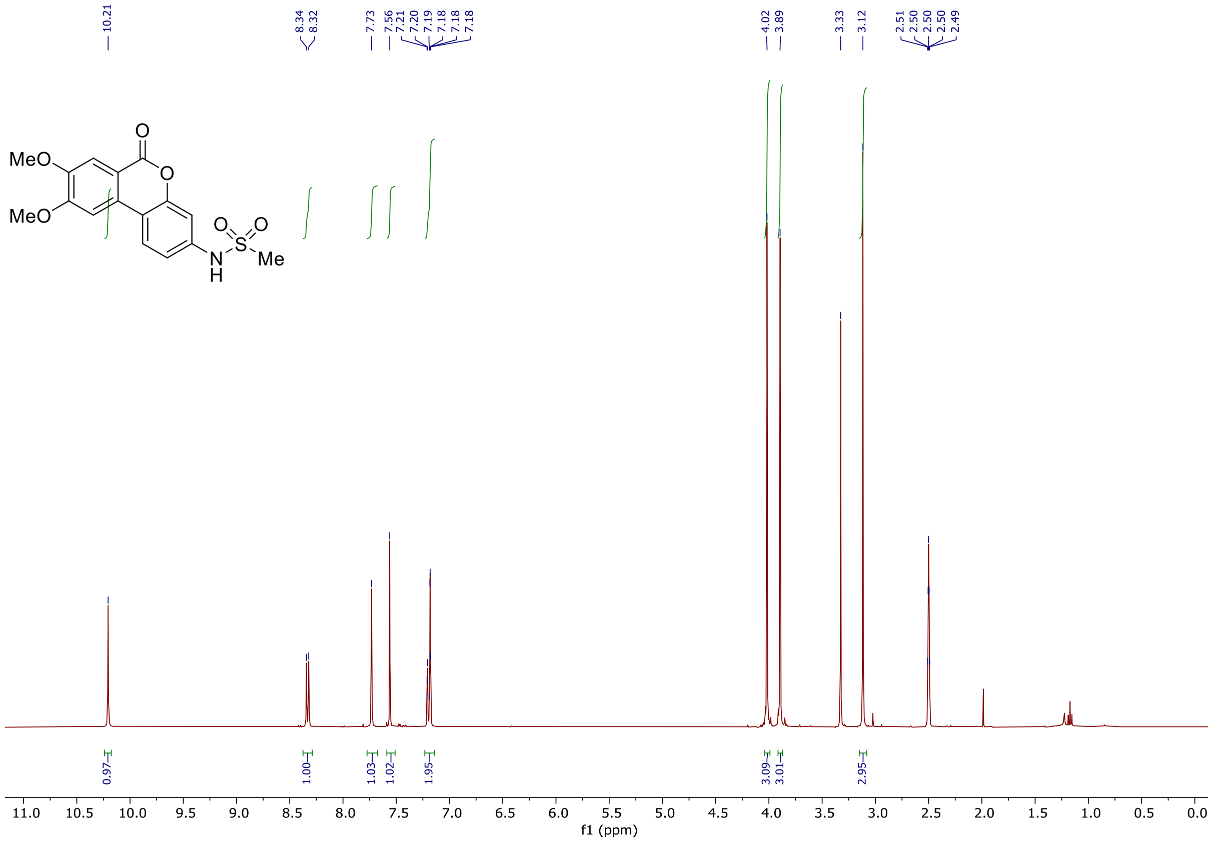

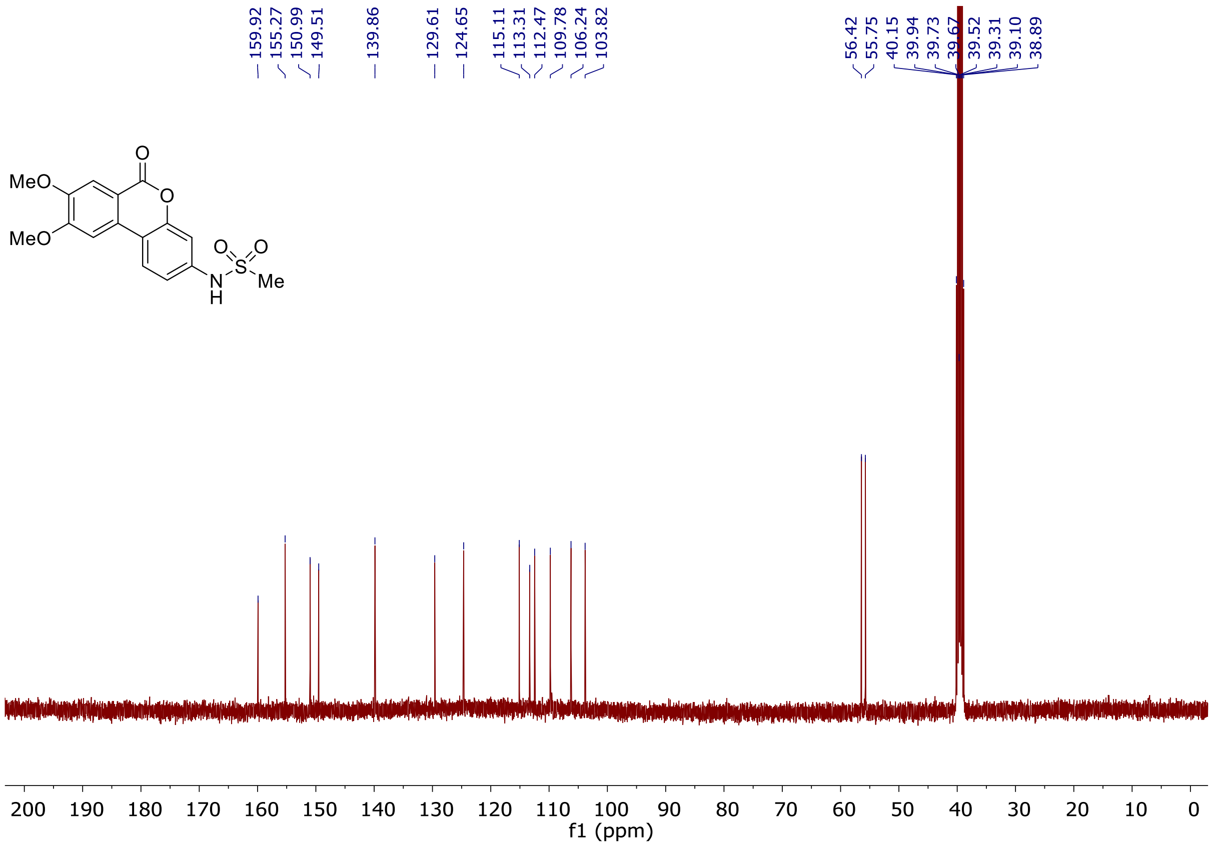

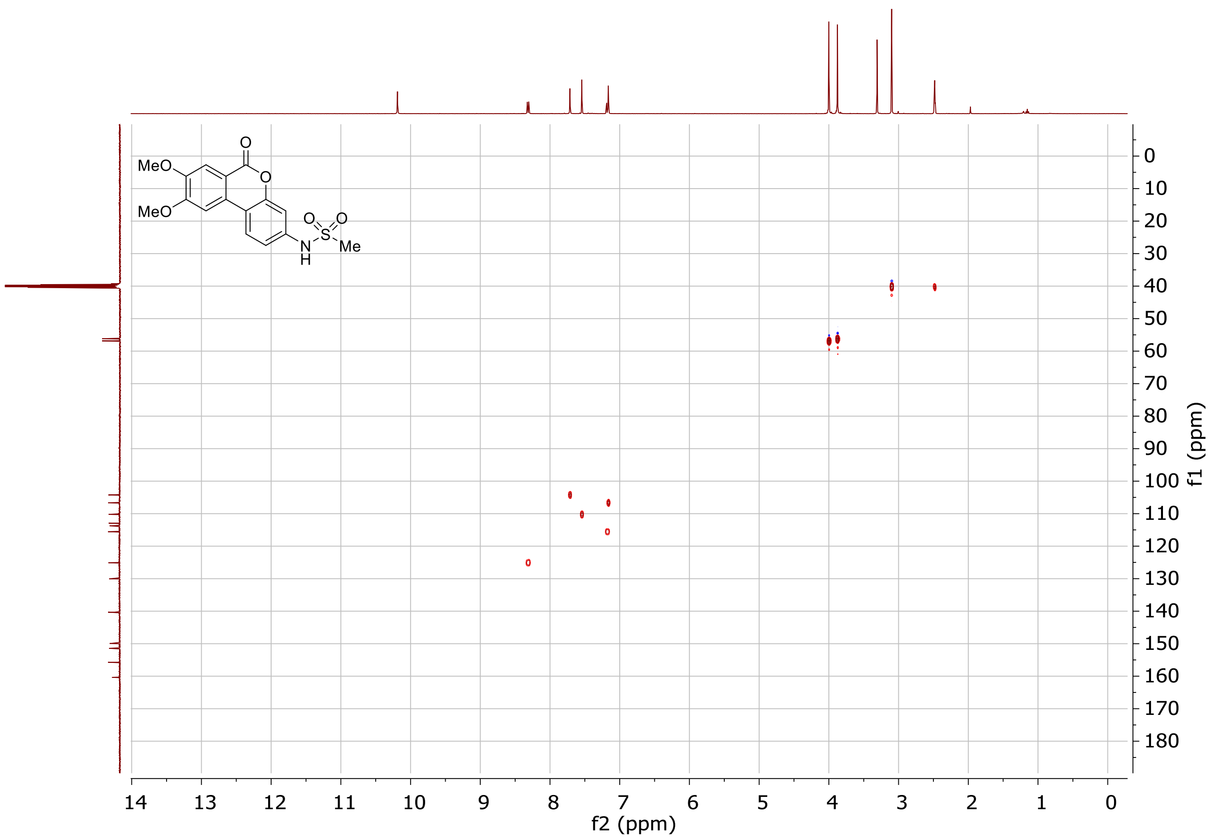

MeO
MeO
O
O
N
H
S
O
O
Me
f2 (ppm)
f1 (ppm)

N-(8,9-Dihydroxy-6-oxo-6H-benzo[c]chromen-3-yl)methanesulfonamide (**36**)

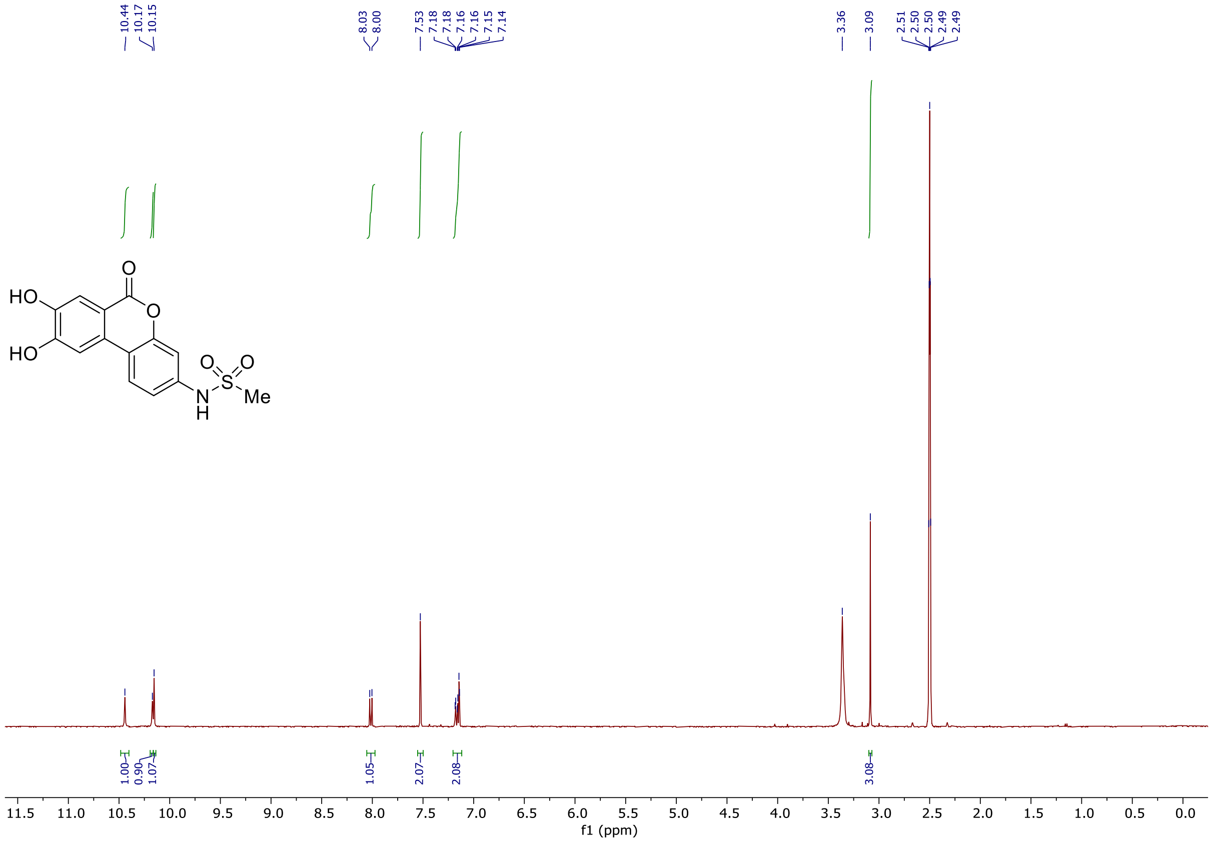

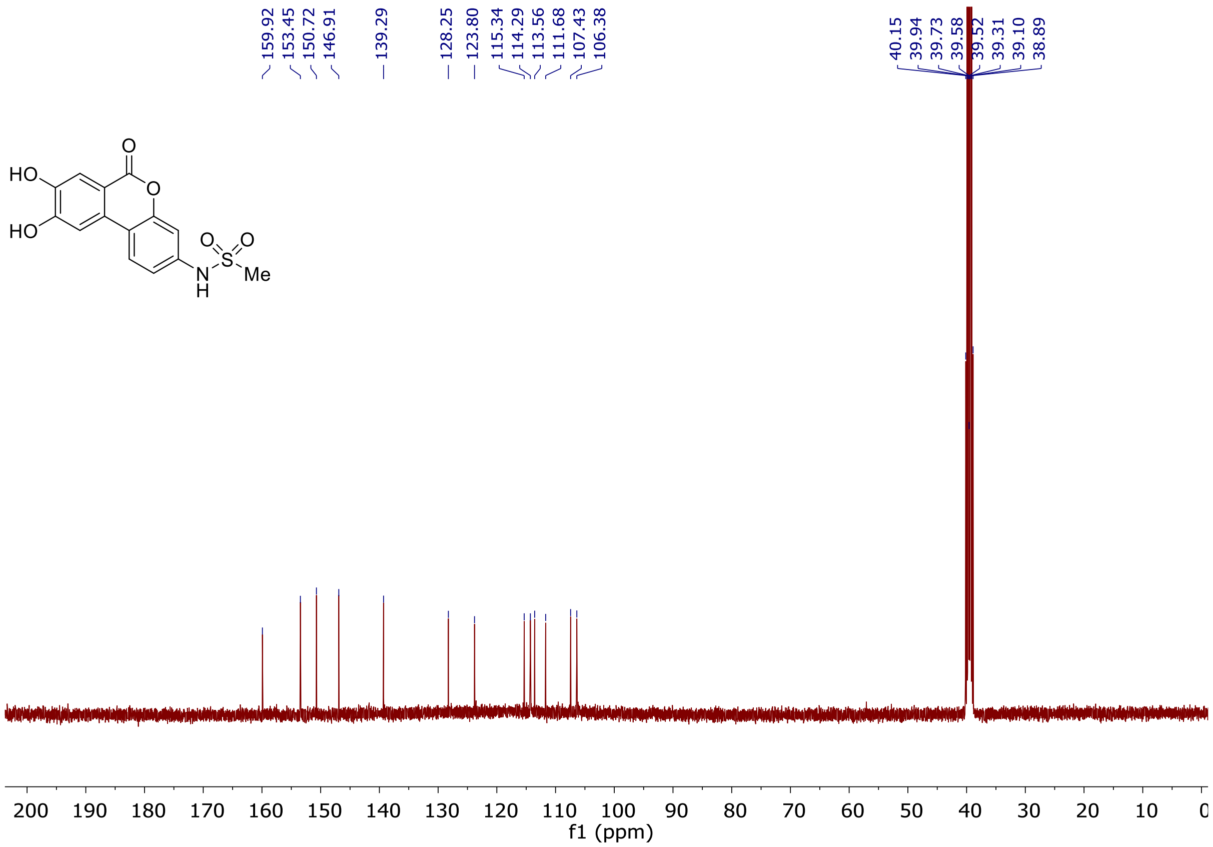

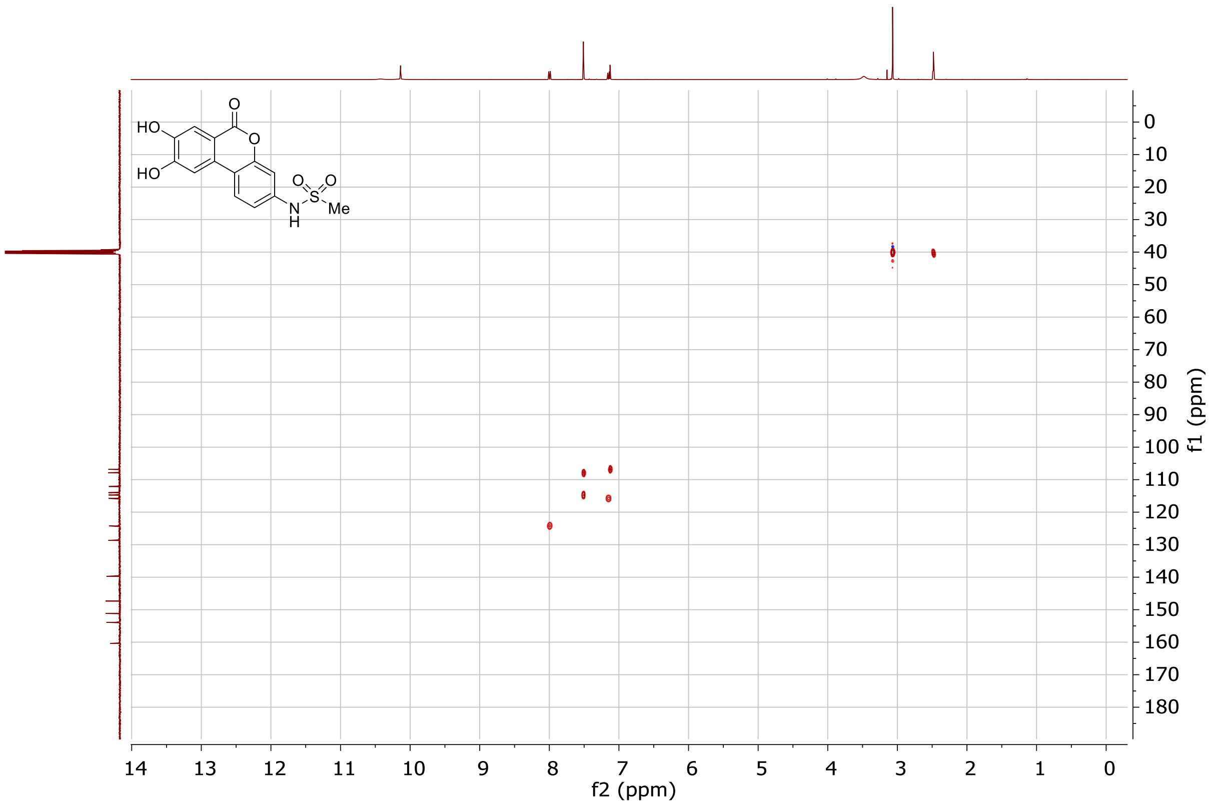
HO
HO
O
O
N
H
O
S
O
Me
f2 (ppm)
f1 (ppm)

4'-Fluoro-4,5-dimethoxy-[1,1'-biphenyl]-2-carboxylic acid (**49**)

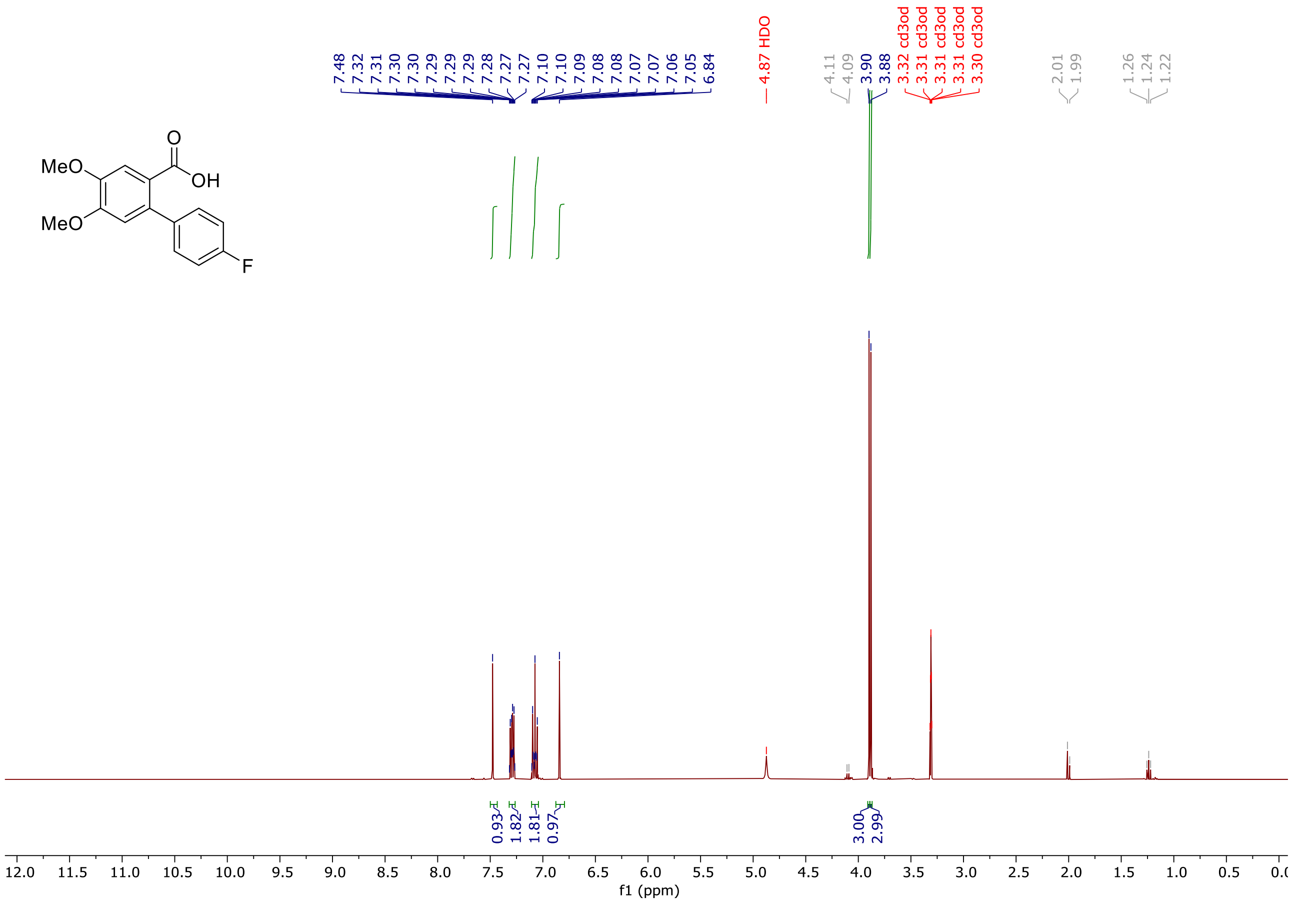

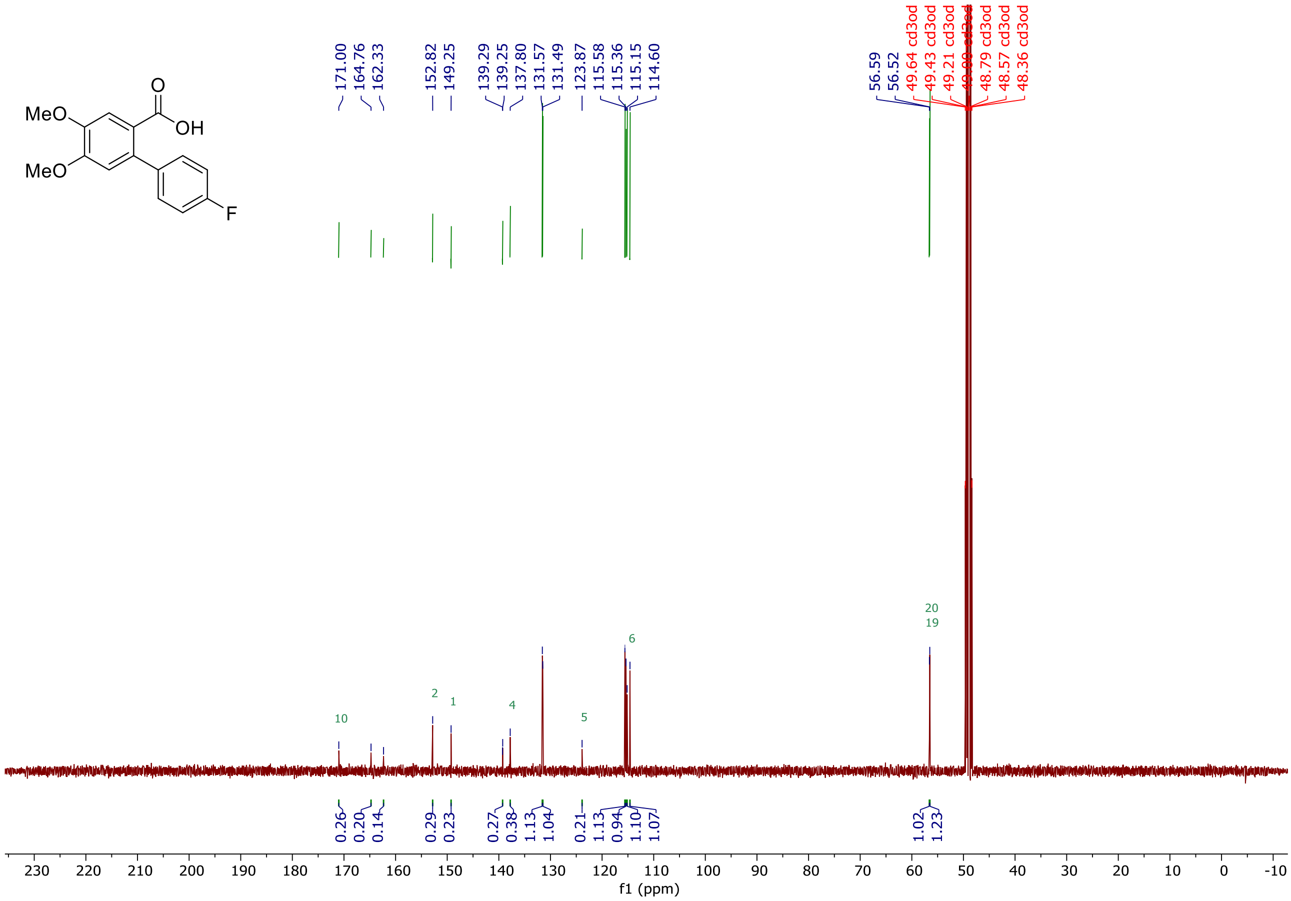

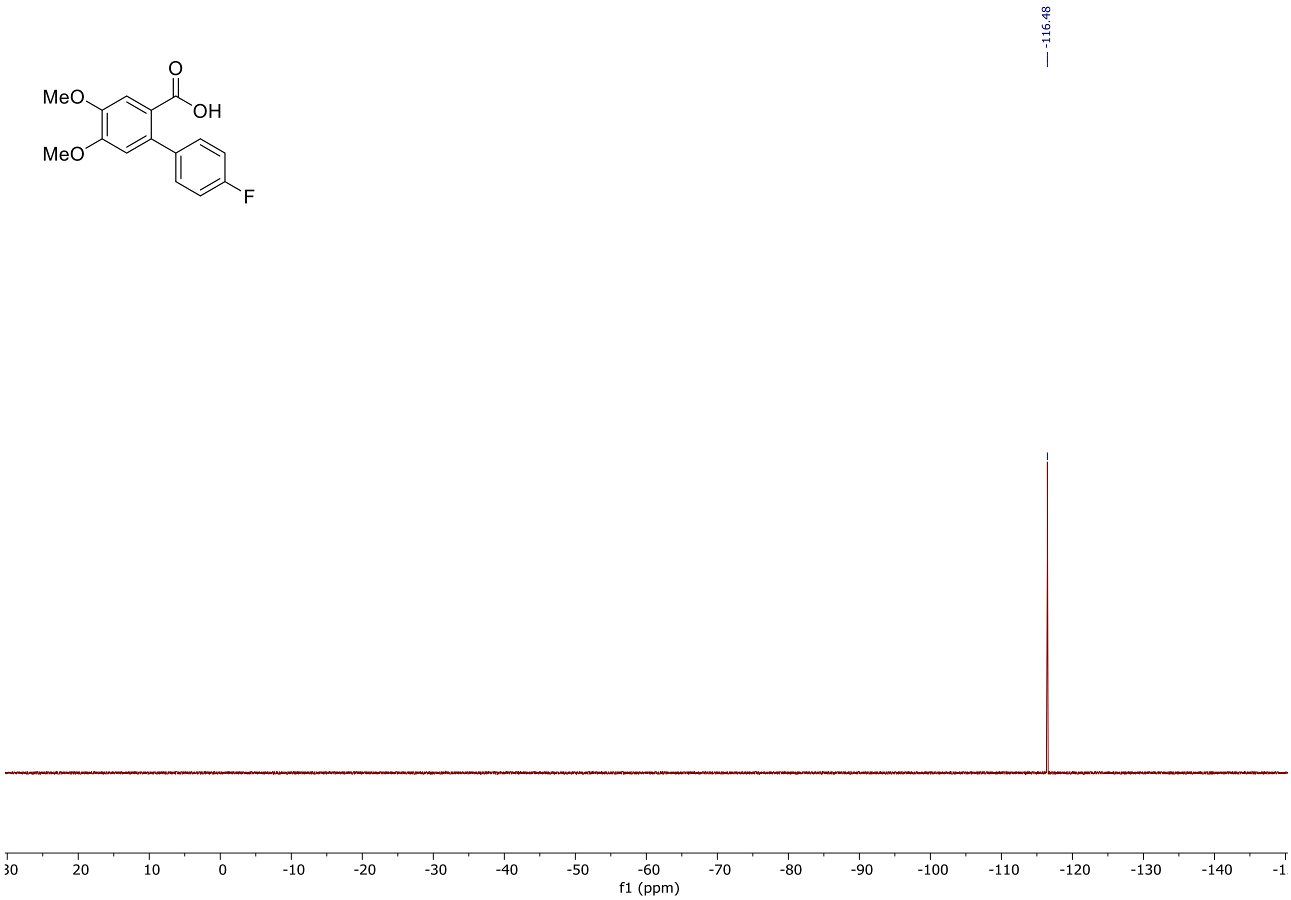
MeO
MeO
O
OH
F
-116.48
30
20
10
0
-10
-20
-30
-40
-50
-60
-70
-80
-90
-100
-110
-120
-130
-140
f1 (ppm)

3-Fluoro-8,9-dimethoxy-6H-benzo[c]chromen-6-one (**55**)

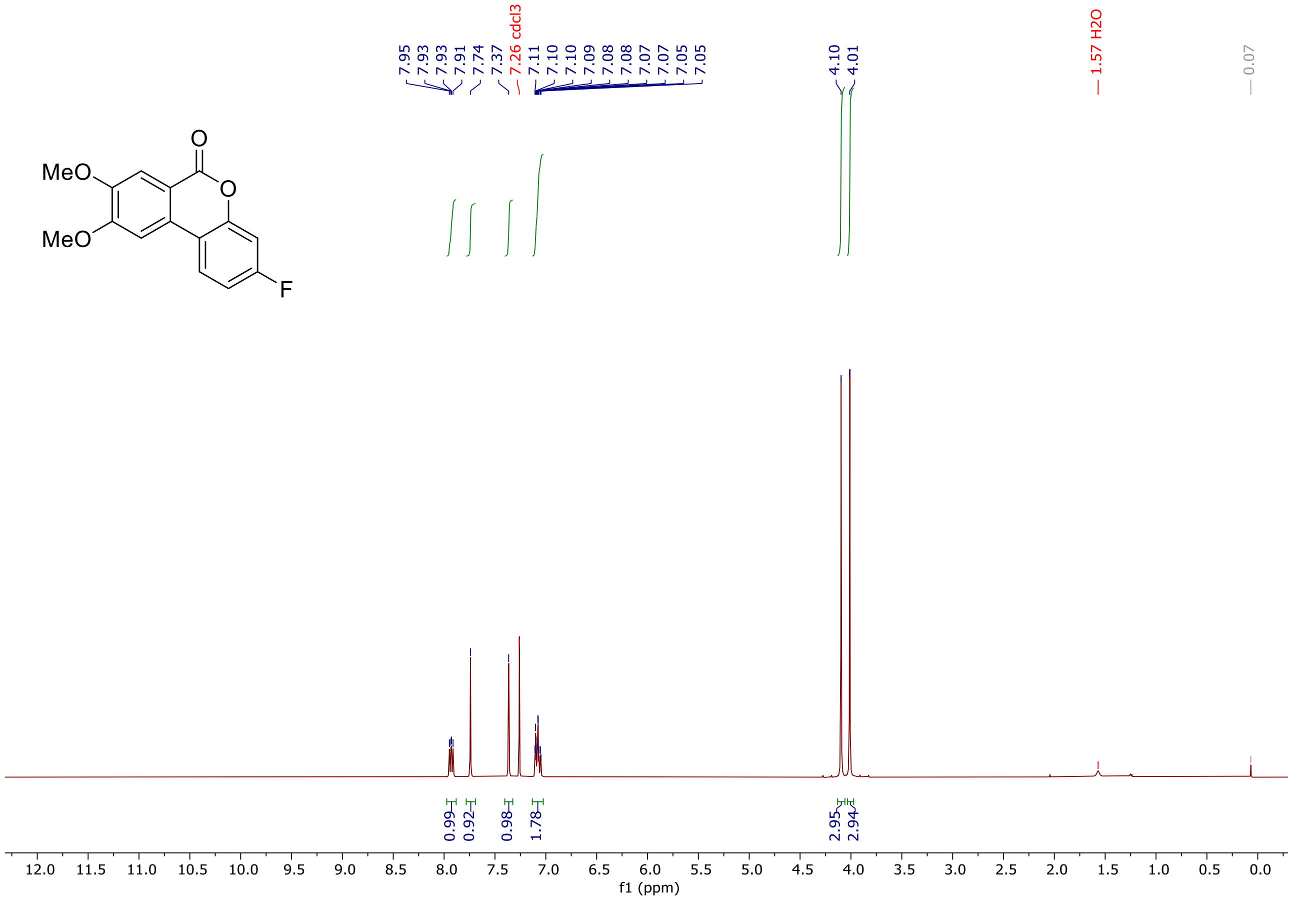

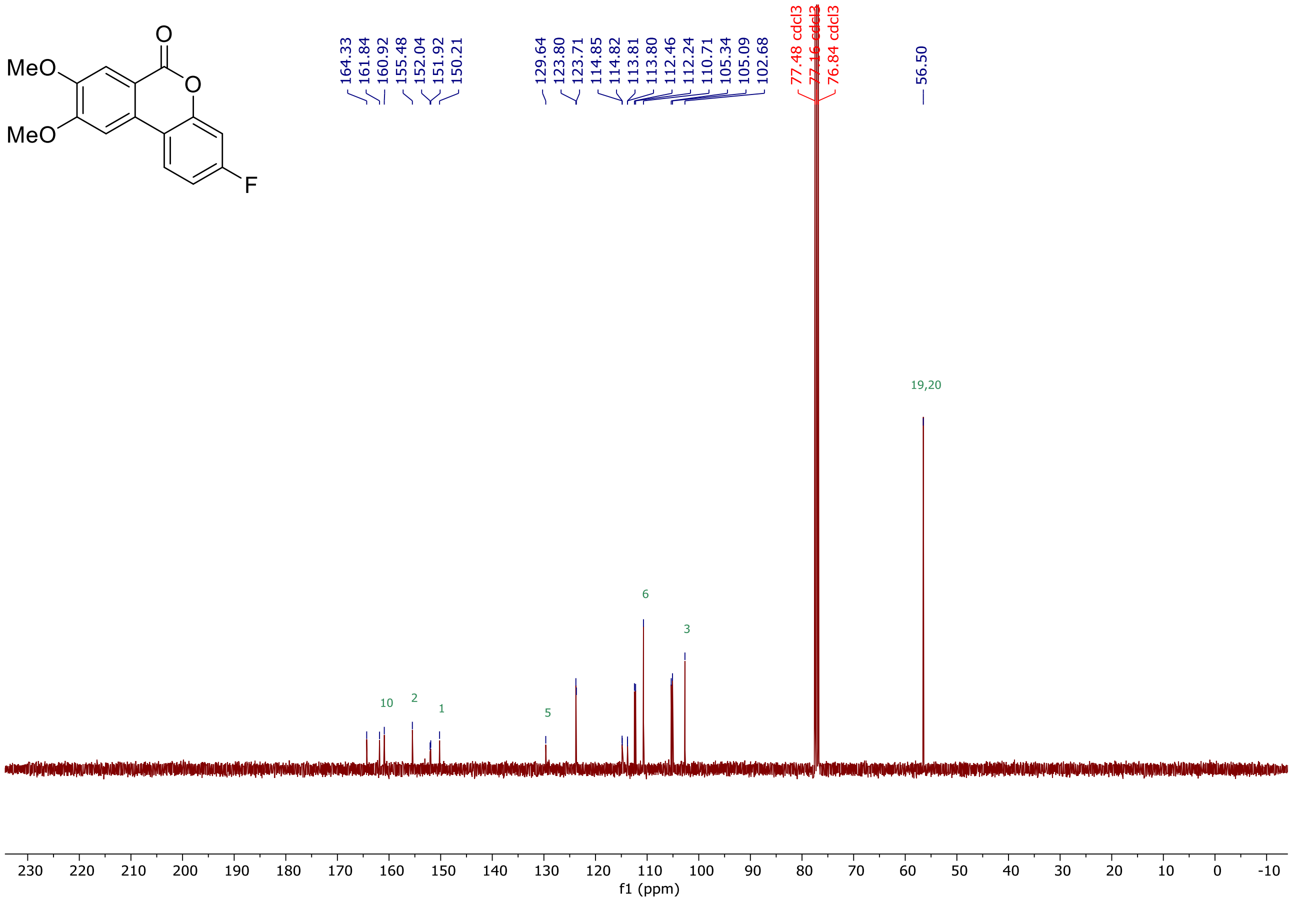

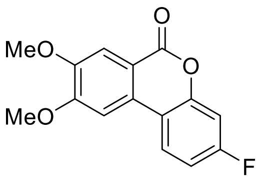
MeO
MeO
O
O
F

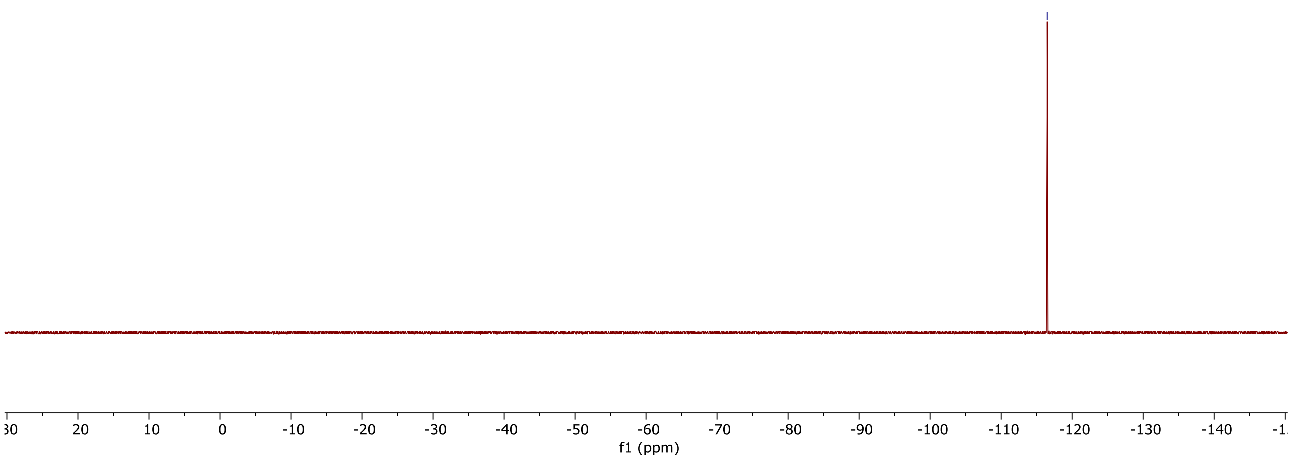
-116.48
30
20
10
0
-10
-20
-30
-40
-50
-60
-70
-80
-90
-100
-110
-120
-130
-140
f1 (ppm)

3-Fluoro-8,9-dihydroxy-6H-benzo[c]chromen-6-one (***61***)

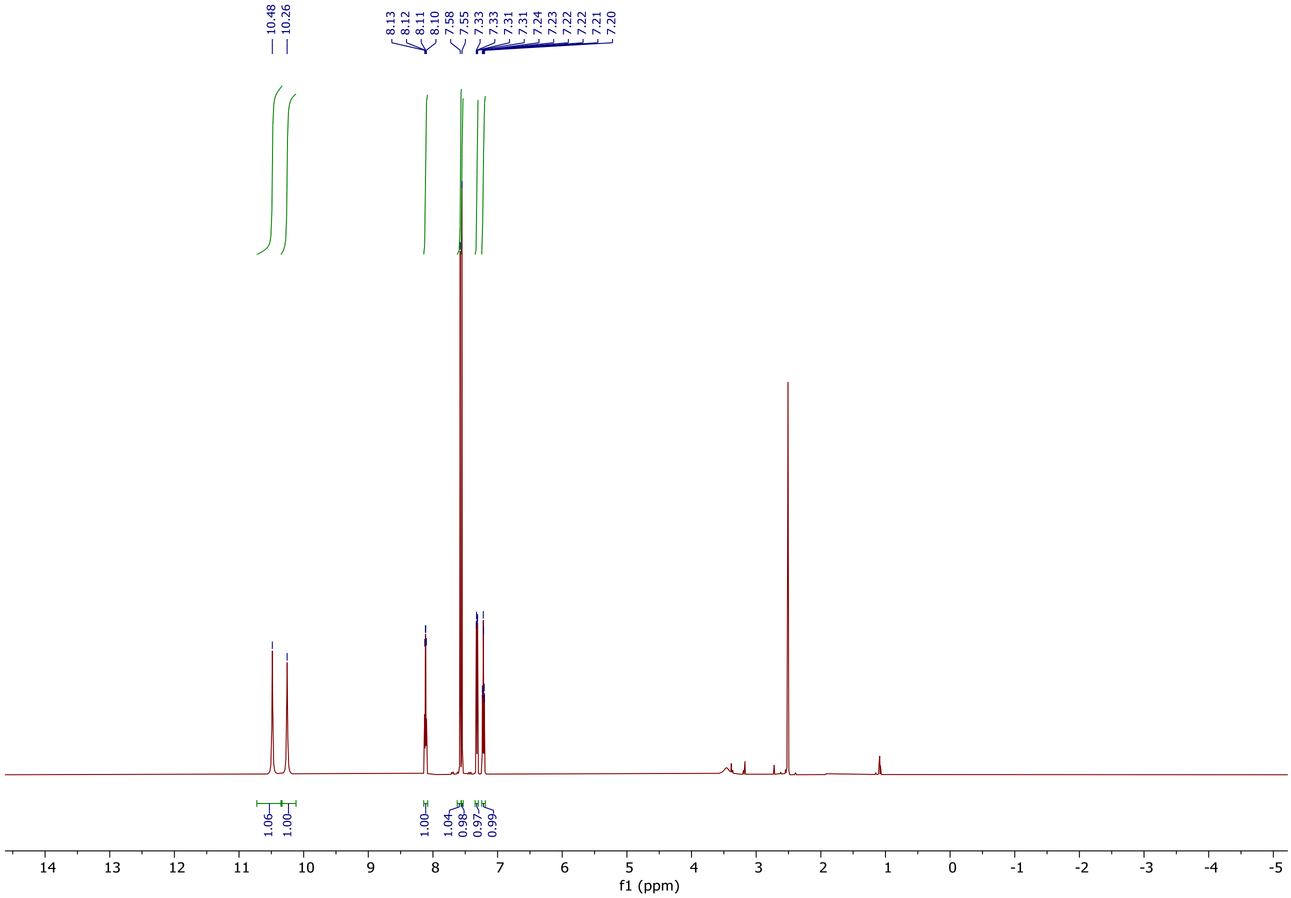

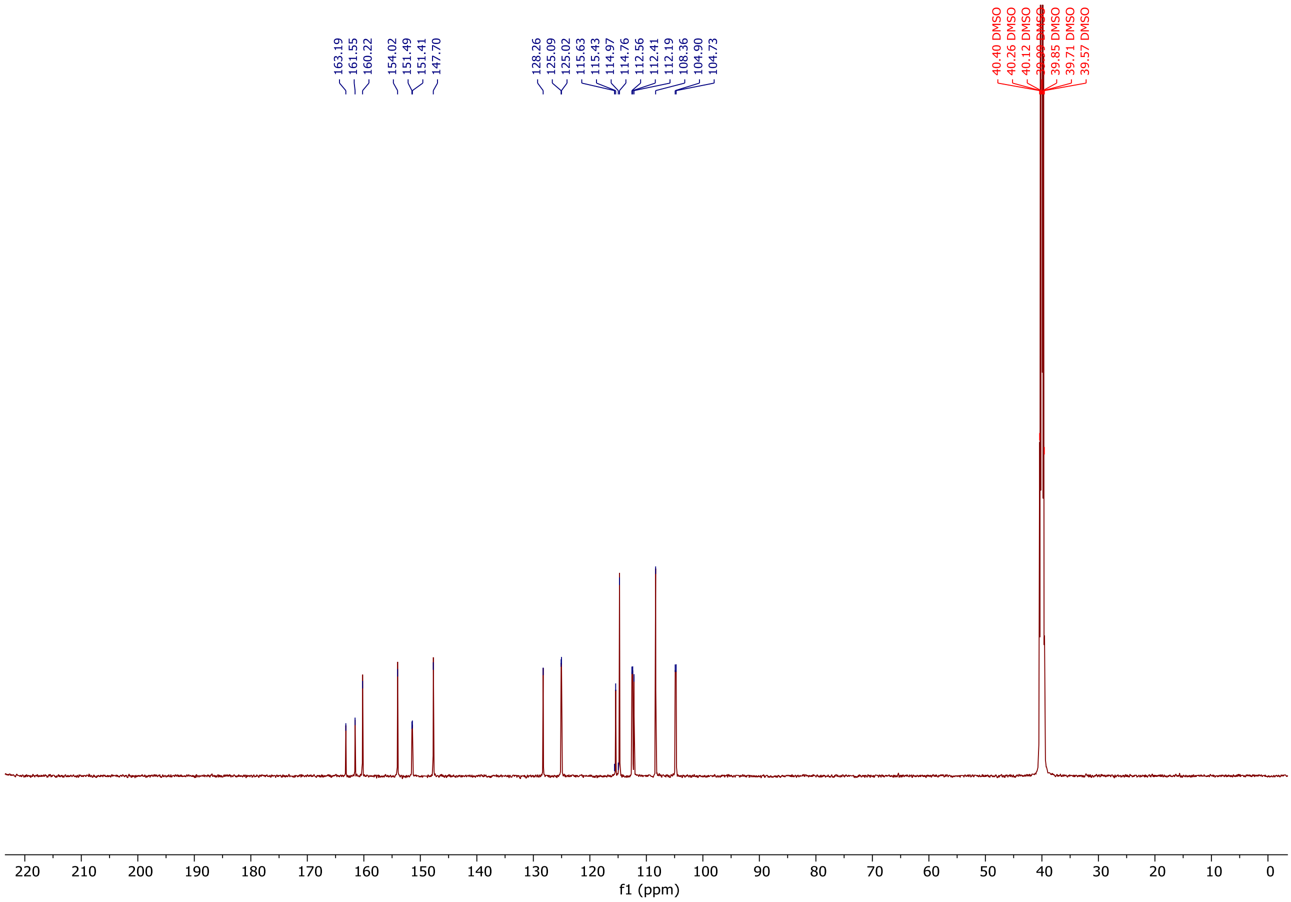

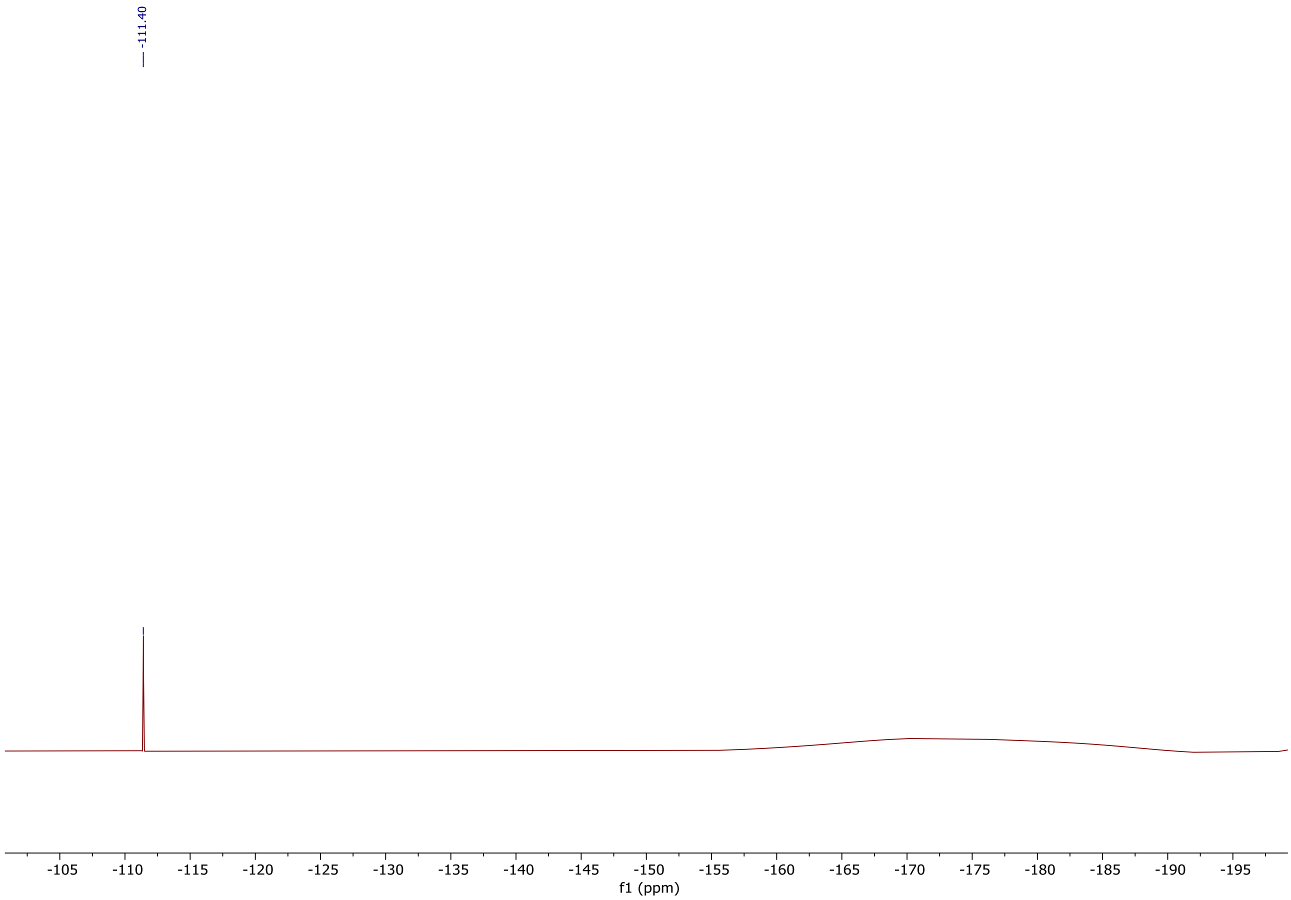
-111.40
-105
-110
-115
-120
-125
-130
-135
-140
-145
-150
-155
-160
-165
-170
-175
-180
-185
-190
-195
f1 (ppm)

Methyl 4,5-dimethoxy-4'-(trifluoromethyl)-[1,1'-biphenyl]-2-carboxylate (**44**)

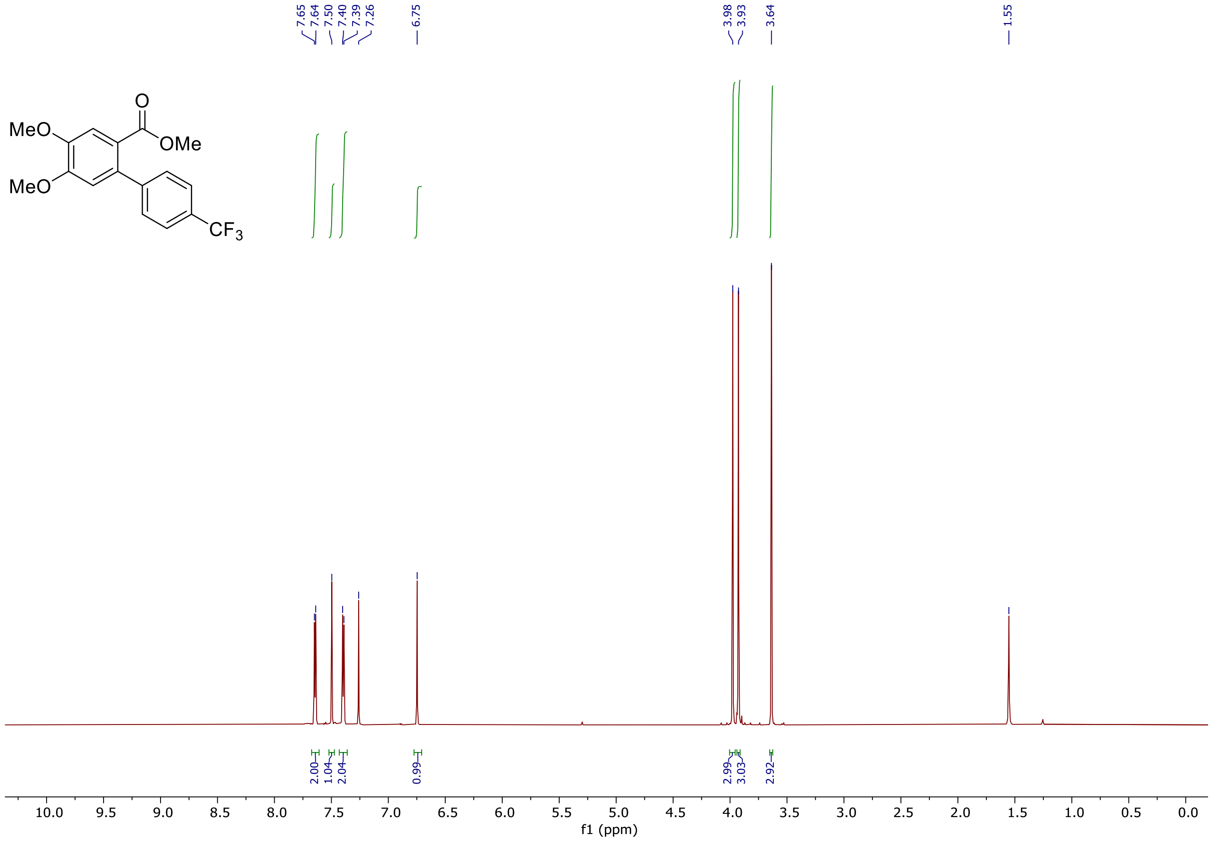

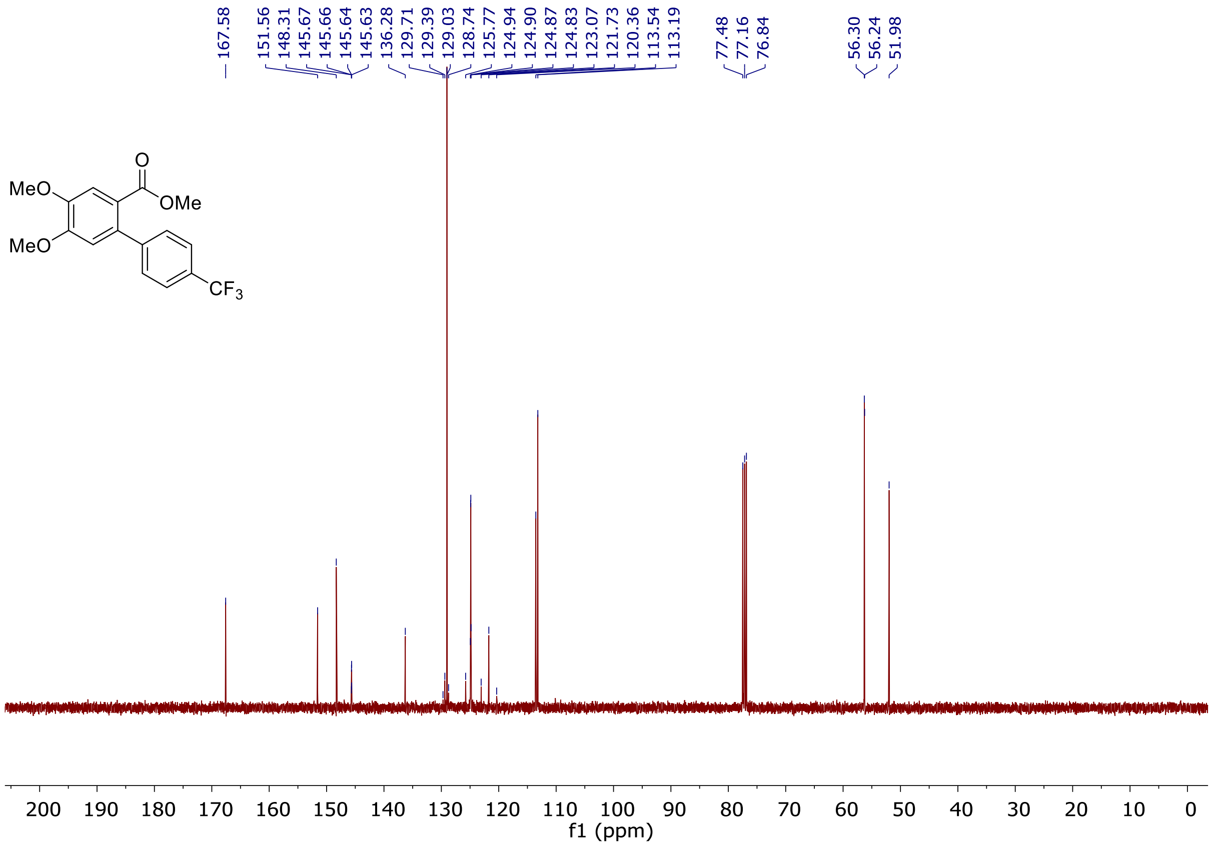

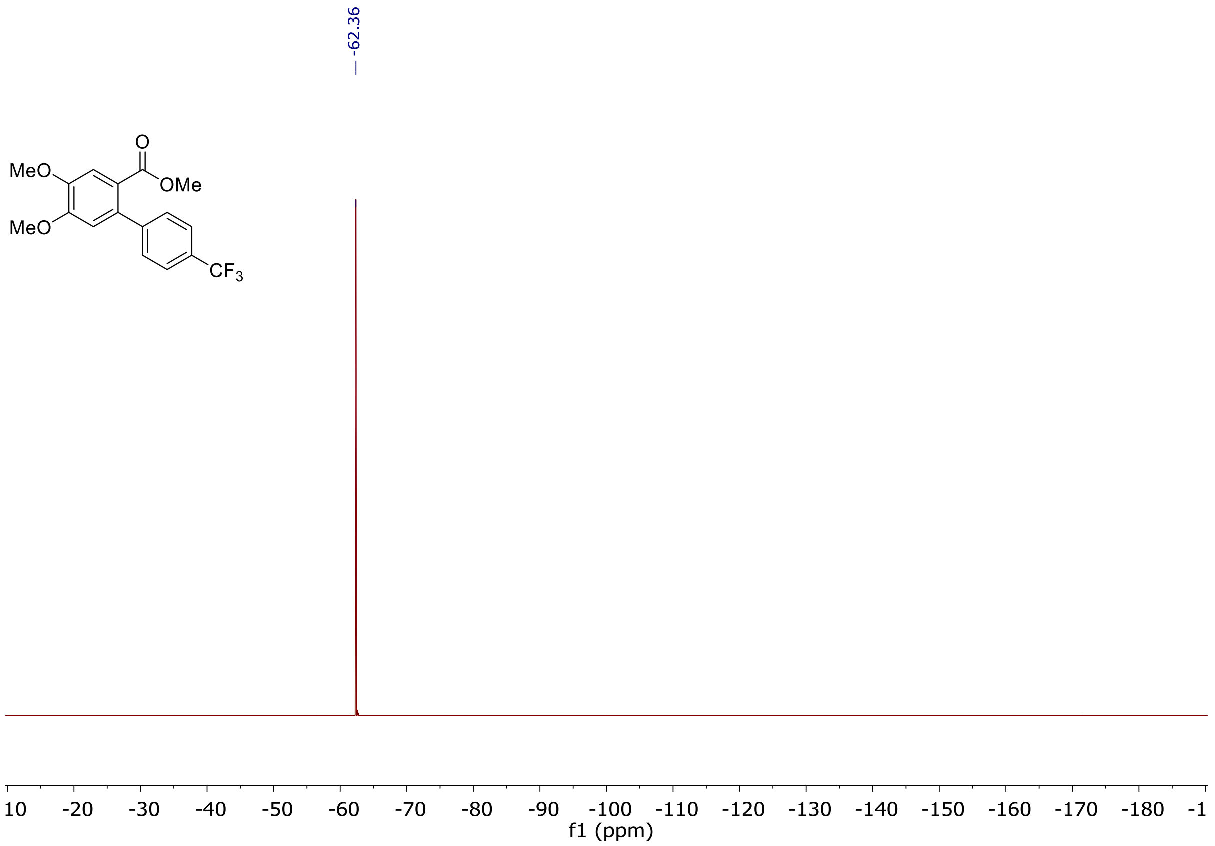
-62.36
MeO
MeO
O
OMe
CF3
10 -20 -30 -40 -50 -60 -70 -80 -90 -100 -110 -120 -130 -140 -150 -160 -170 -180 -1
f1 (ppm)

4,5-Dimethoxy-4'-(trifluoromethyl)-[1,1'-biphenyl]-2-carboxylic acid (**50**)

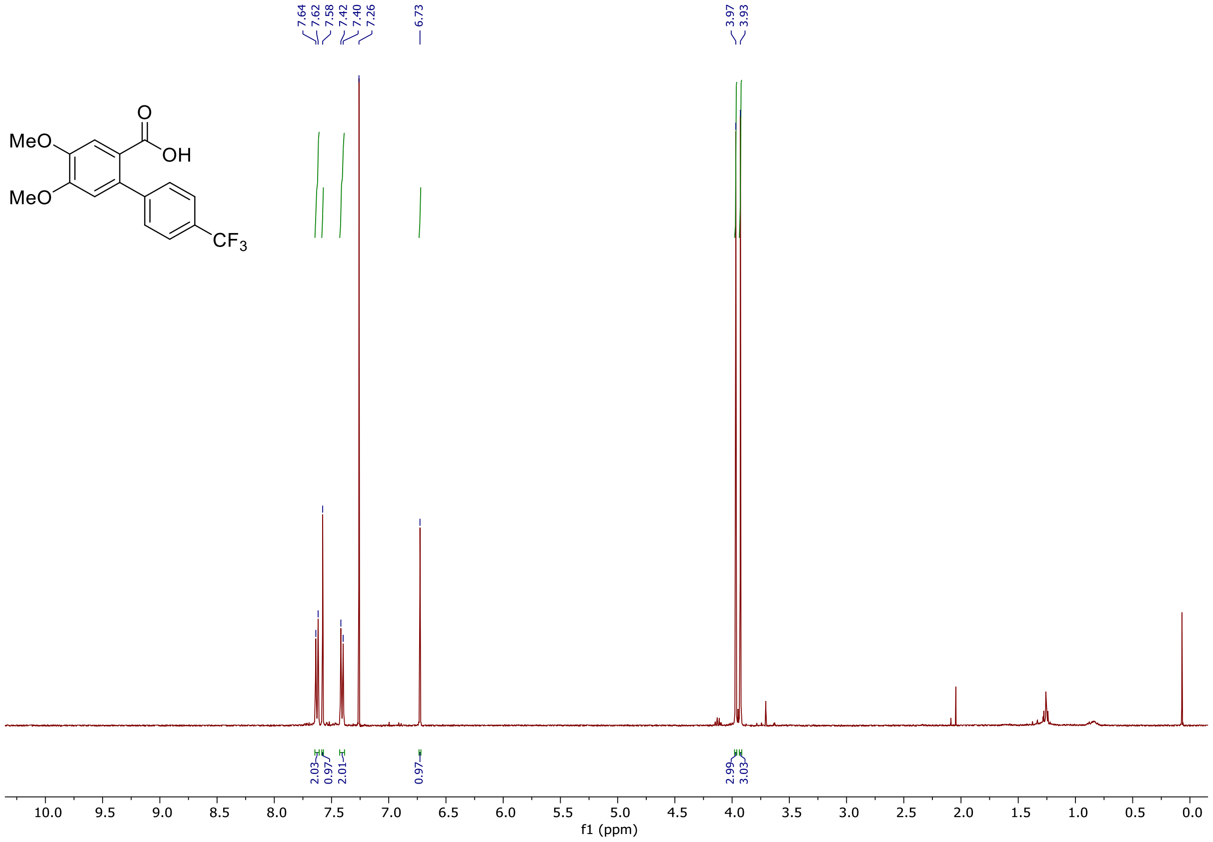

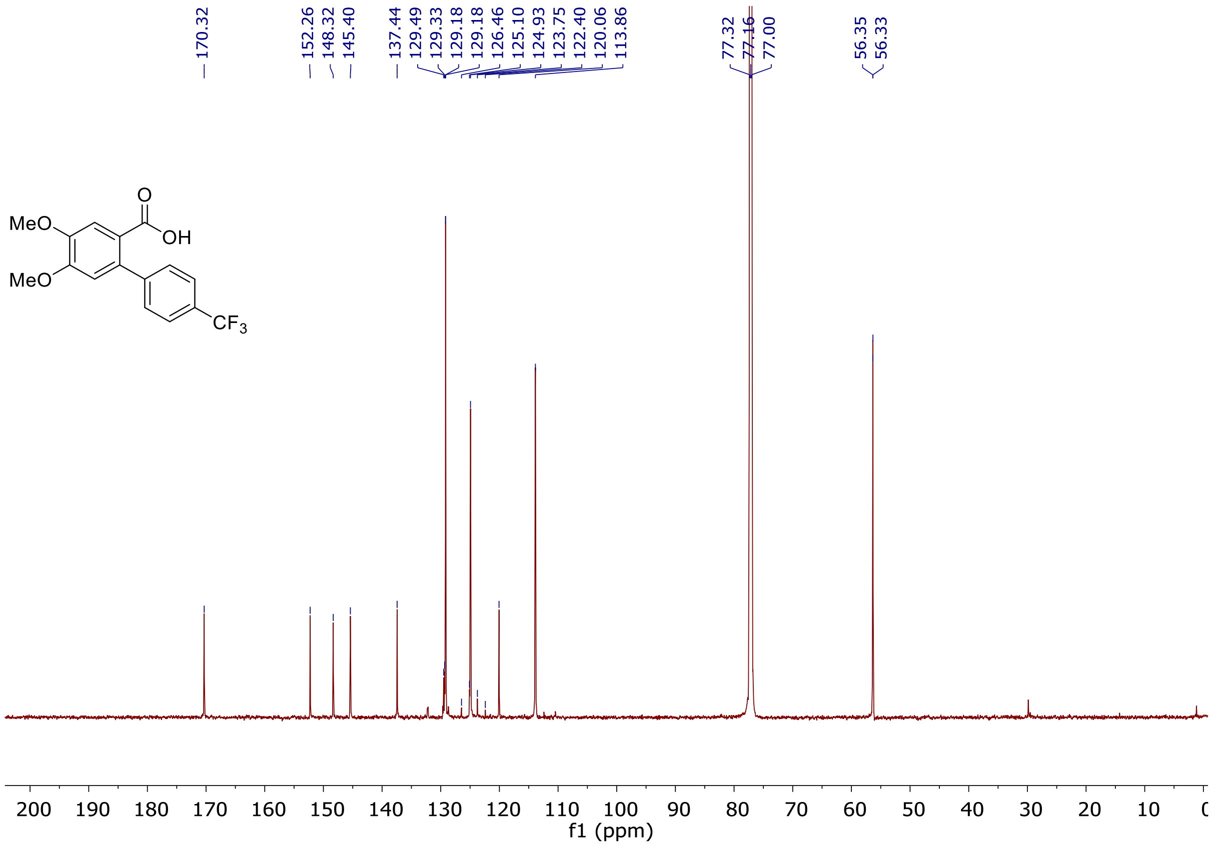

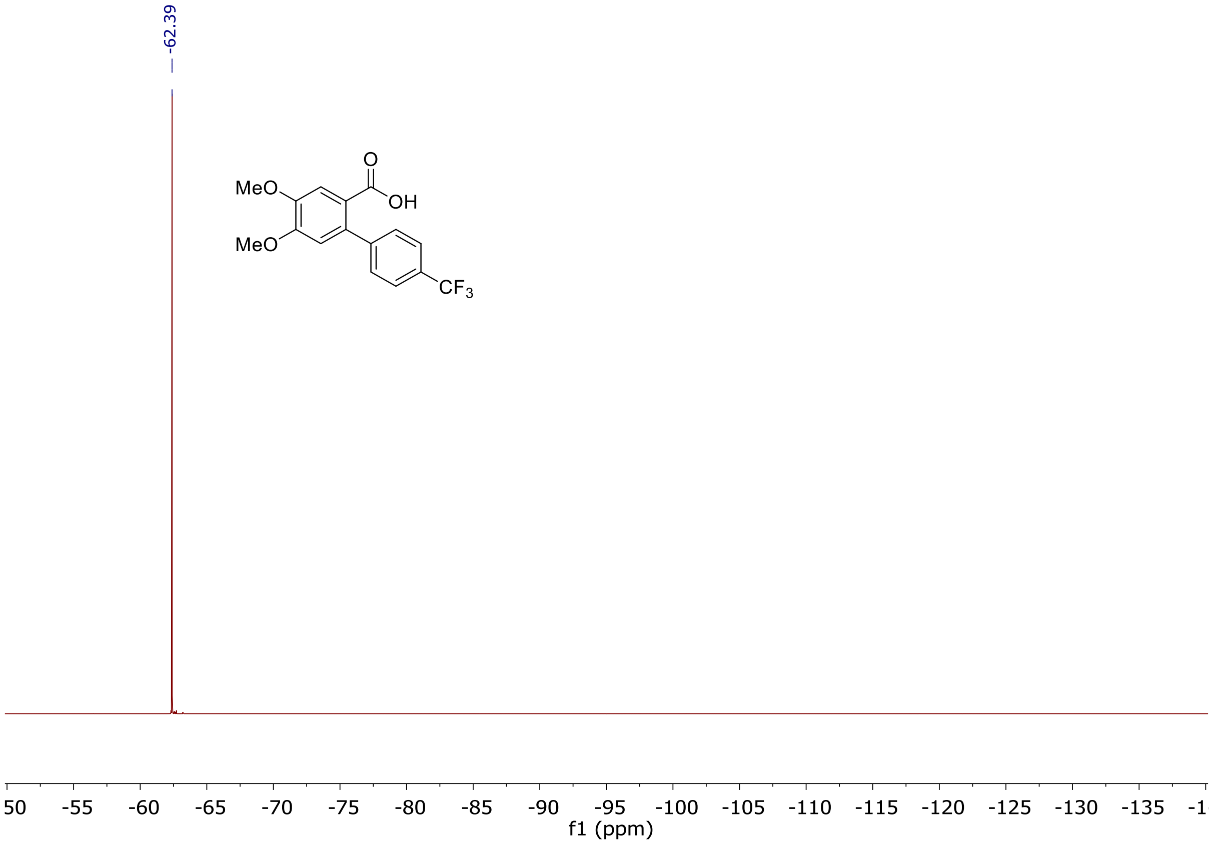
-62.39
MeO
MeO
O
OH
CF3
50
-55
-60
-65
-70
-75
-80
-85
-90
-95
-100
-105
-110
-115
-120
-125
-130
-135
-1
f1 (ppm)

8,9-Dimethoxy-3-(trifluoromethyl)-6H-benzo[c]chromen-6-one (**56**)

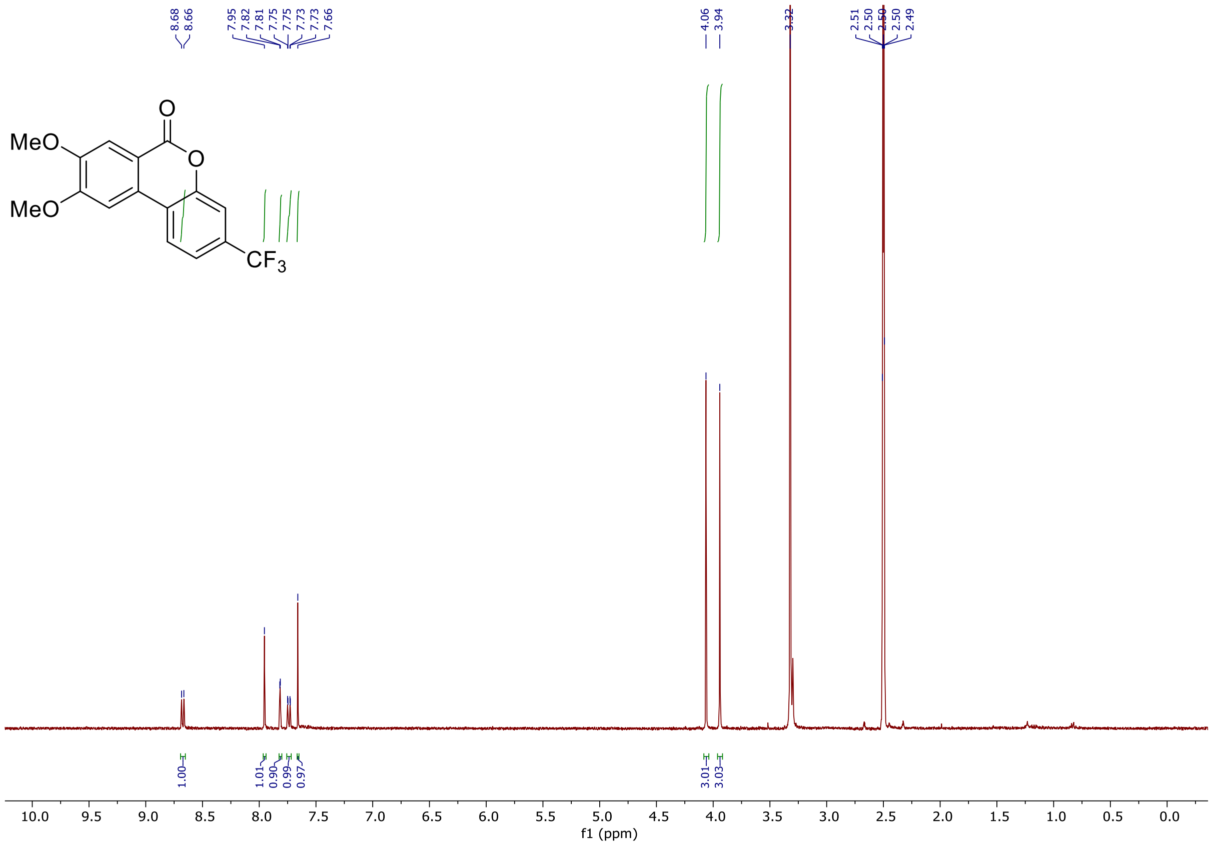

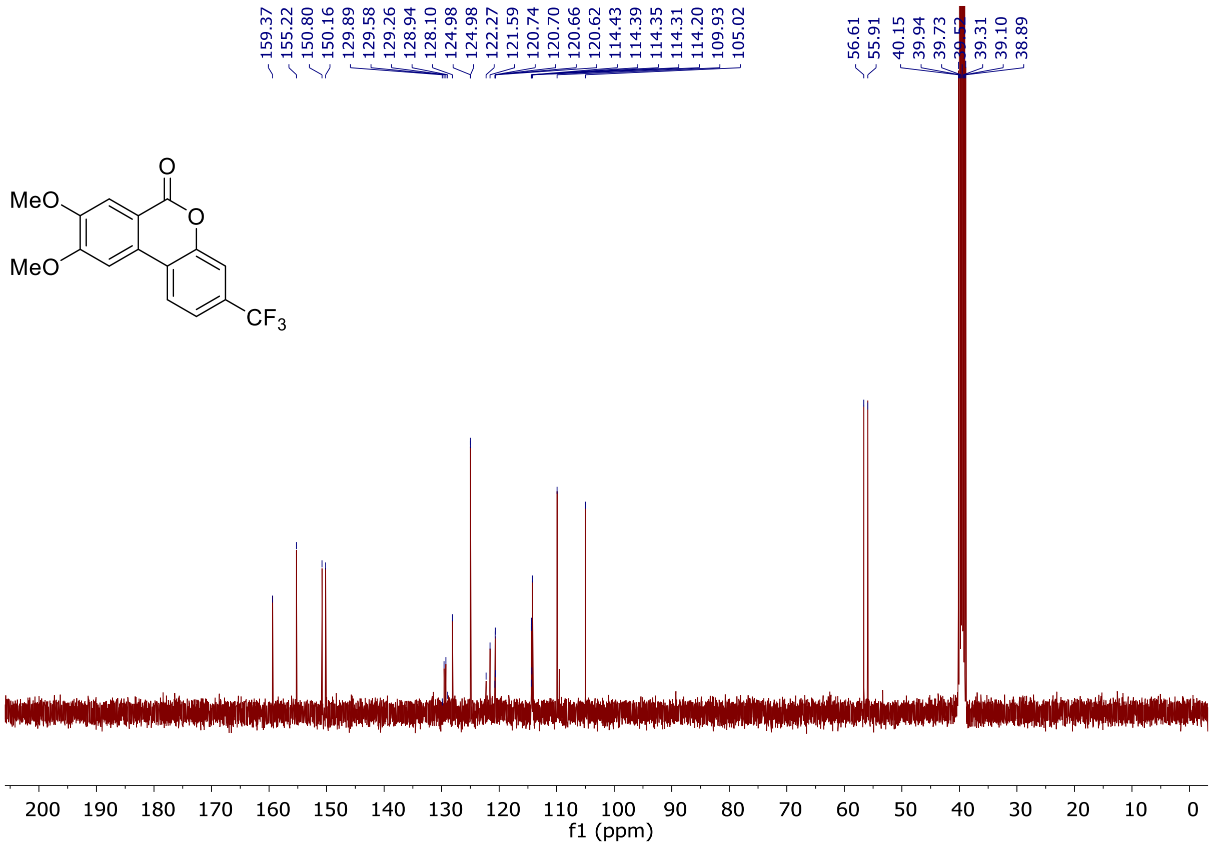

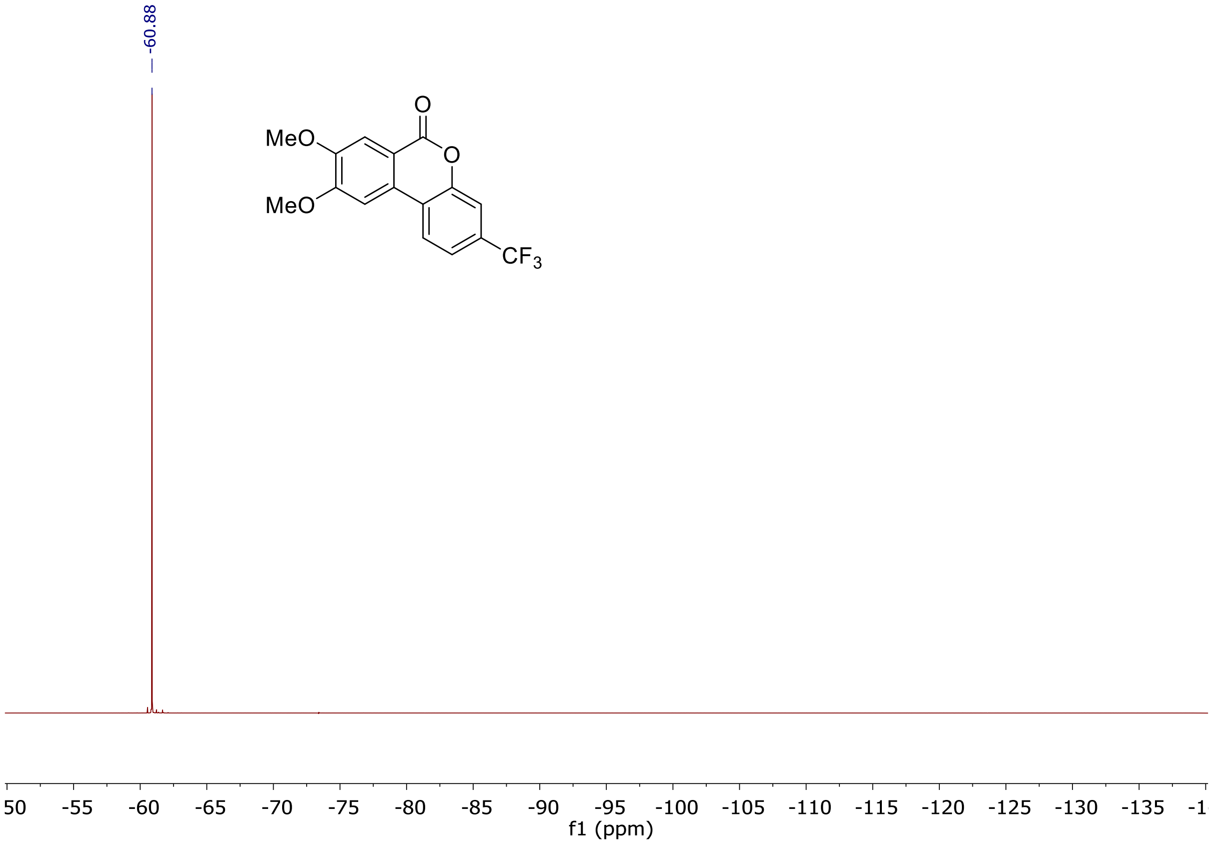

-60.88
O
MeO
O
MeO
CF3
50 -55 -60 -65 -70 -75 -80 -85 -90 -95 -100 -105 -110 -115 -120 -125 -130 -135 -1
f1 (ppm)

8,9-Dihydroxy-3-(trifluoromethyl)-6H-benzo[c]chromen-6-one (**62**)

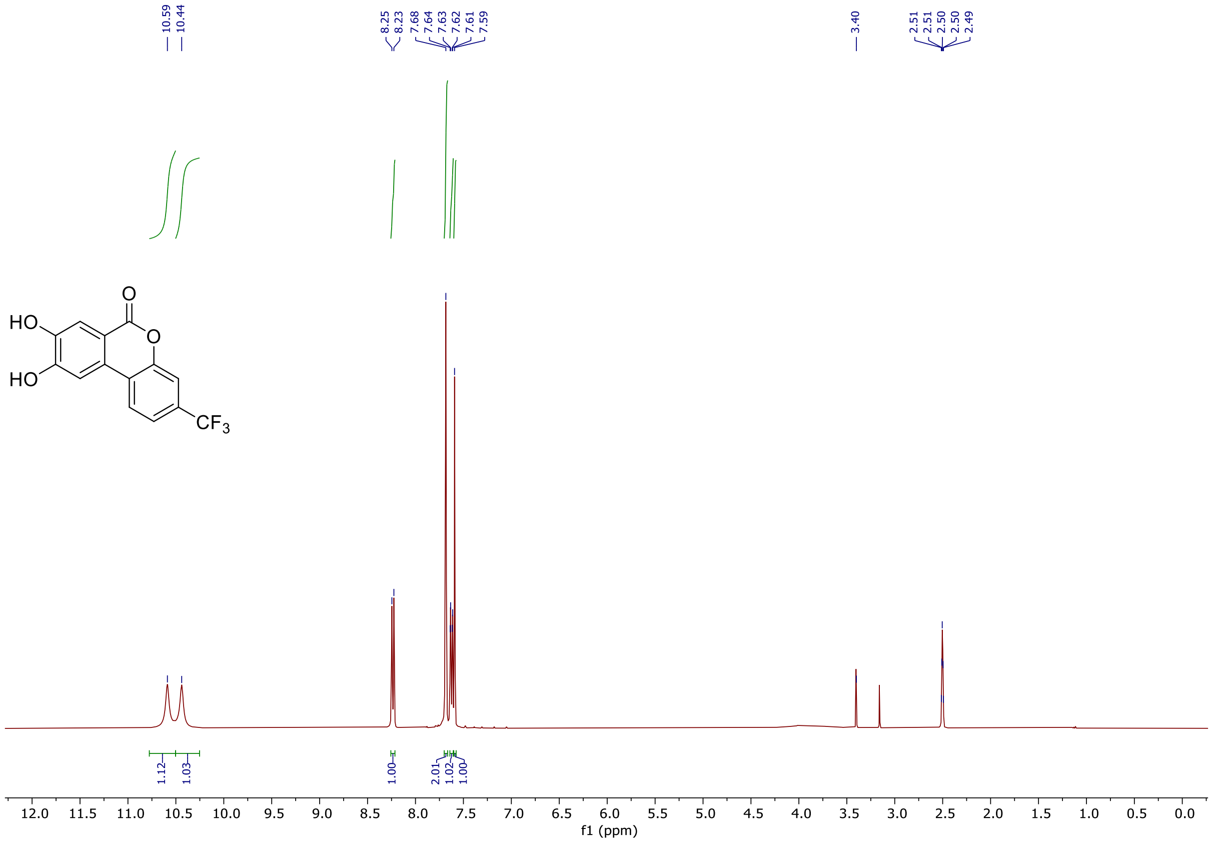

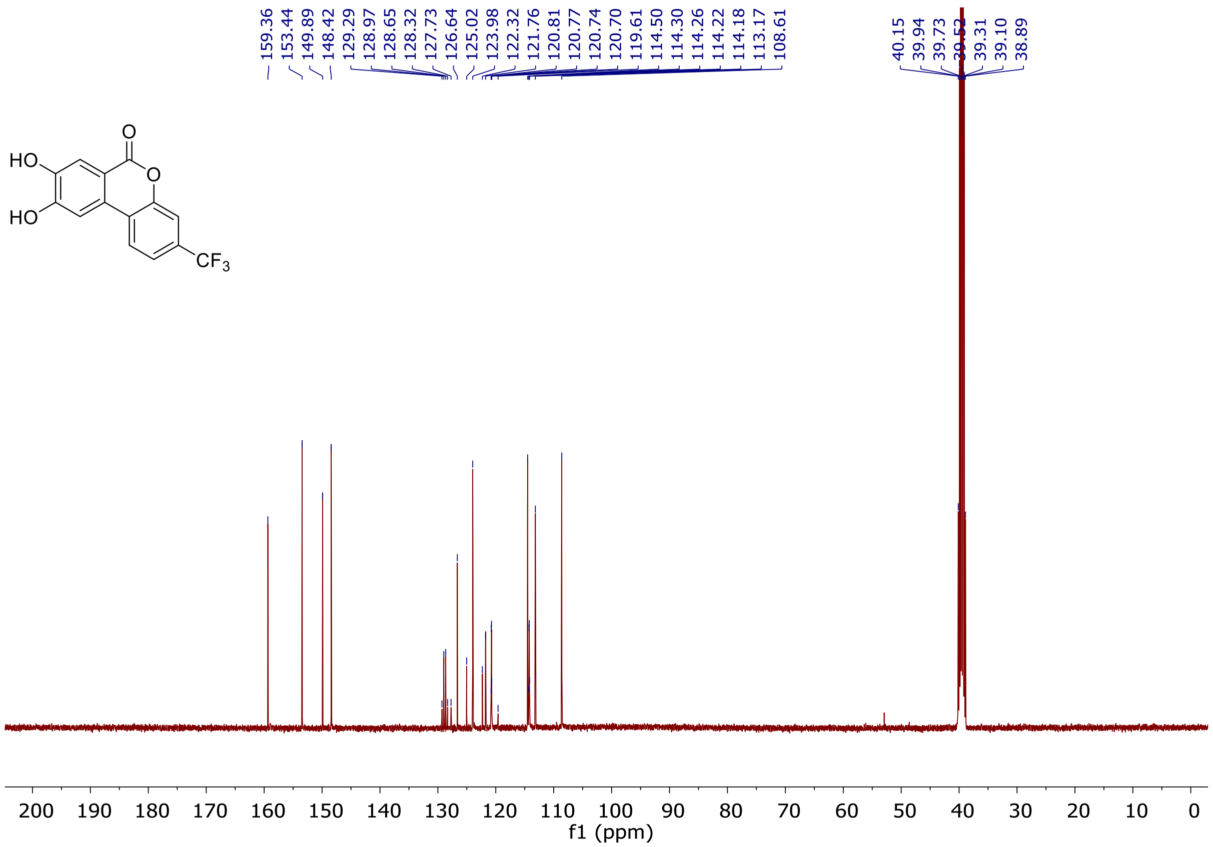

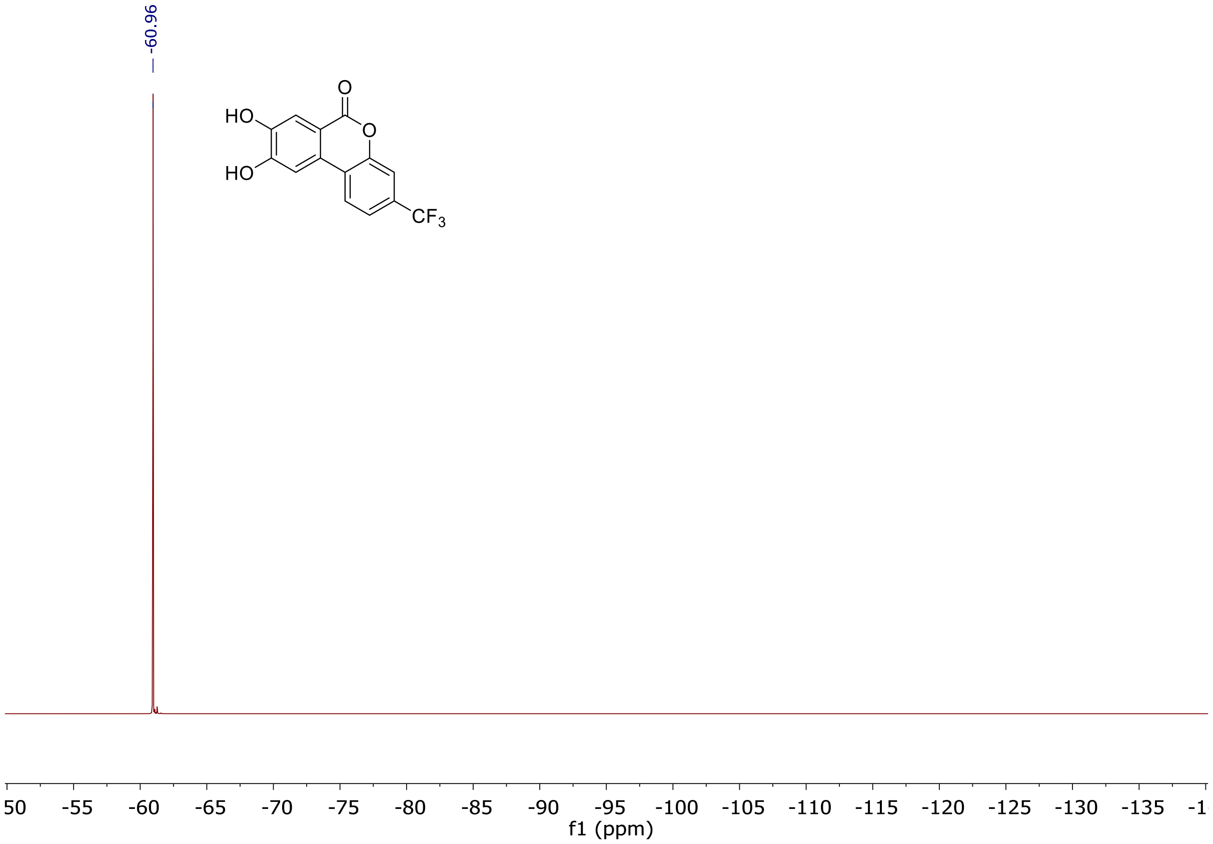

-60.96
HO
HO
O
O
CF3
50
-55
-60
-65
-70
-75
-80
-85
-90
-95
-100
-105
-110
-115
-120
-125
-130
-135
-1
f1 (ppm)

Methyl 4,5-dimethoxy-4'-methyl-[1,1'-biphenyl]-2-carboxylate (**45**)

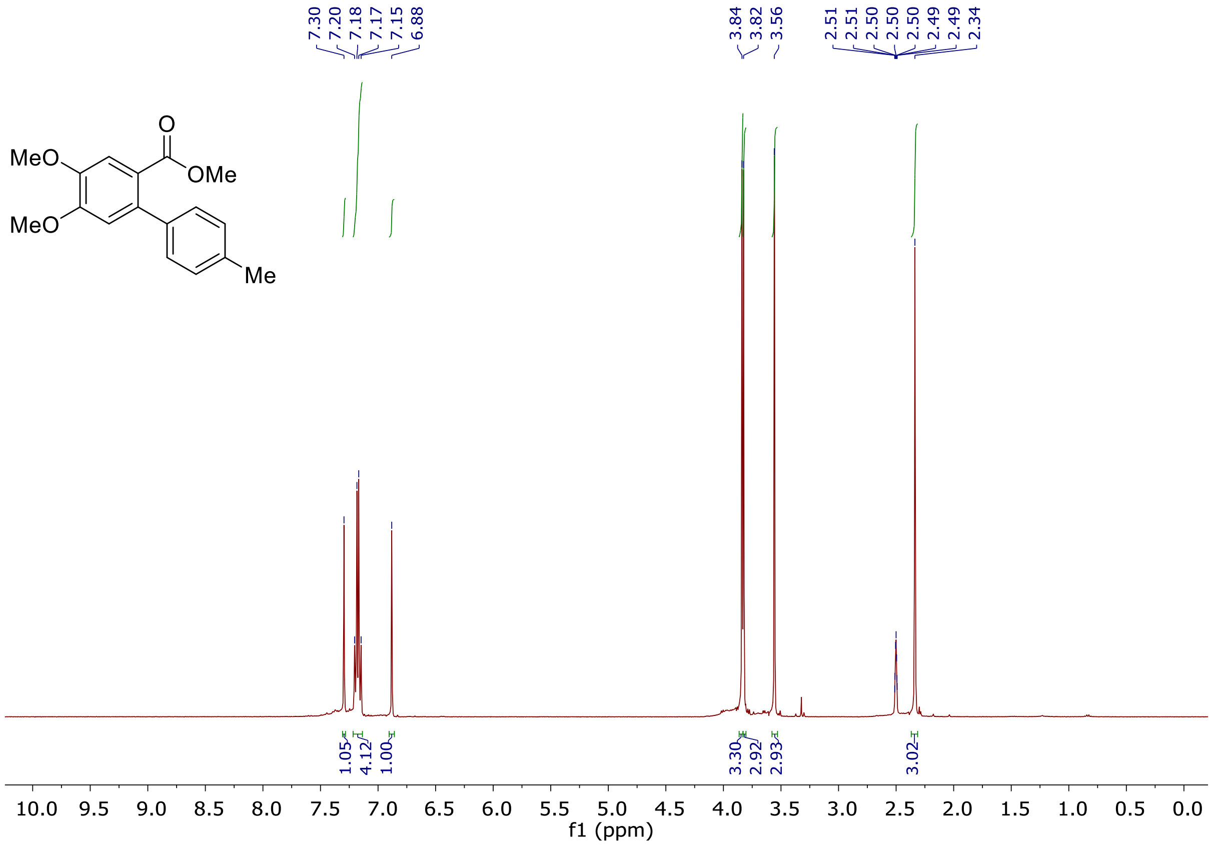

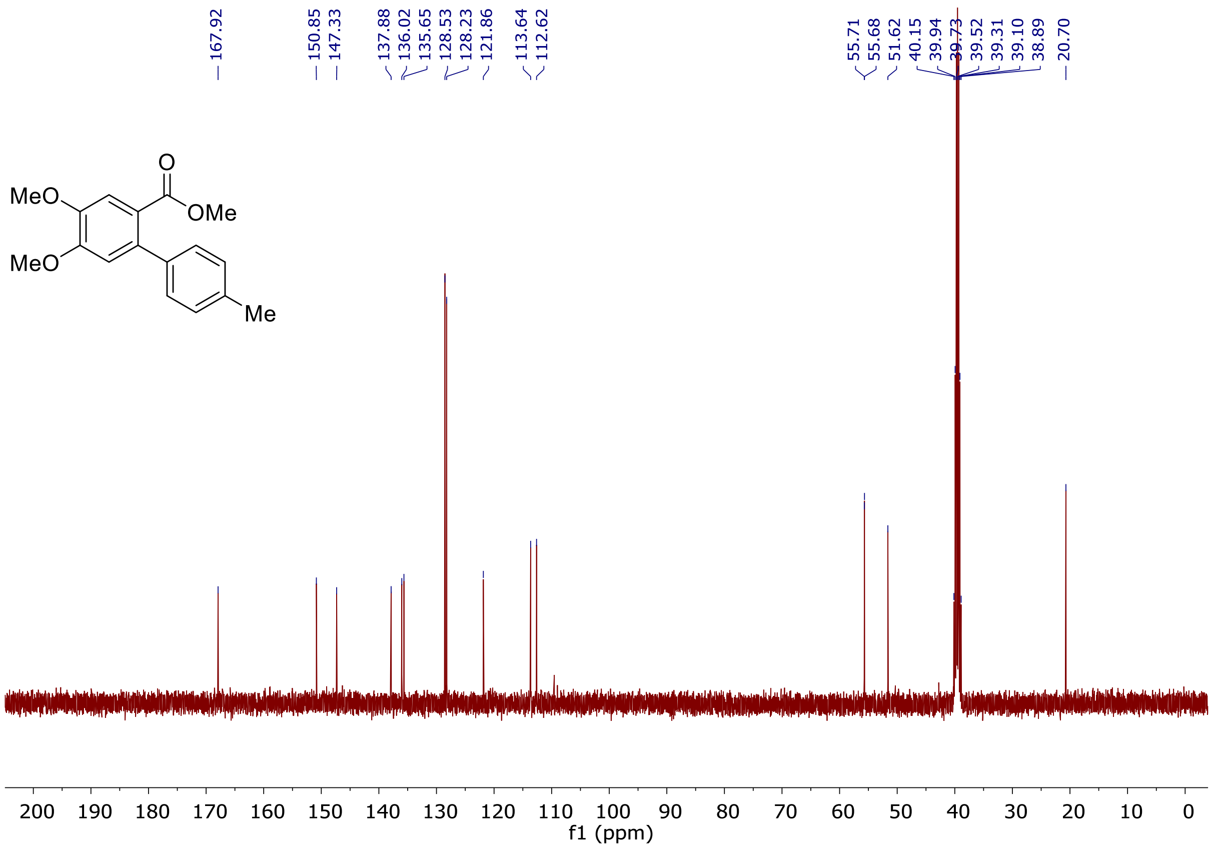

4,5-Dimethoxy-4'-methyl-[1,1'-biphenyl]-2-carboxylic acid (**51**)

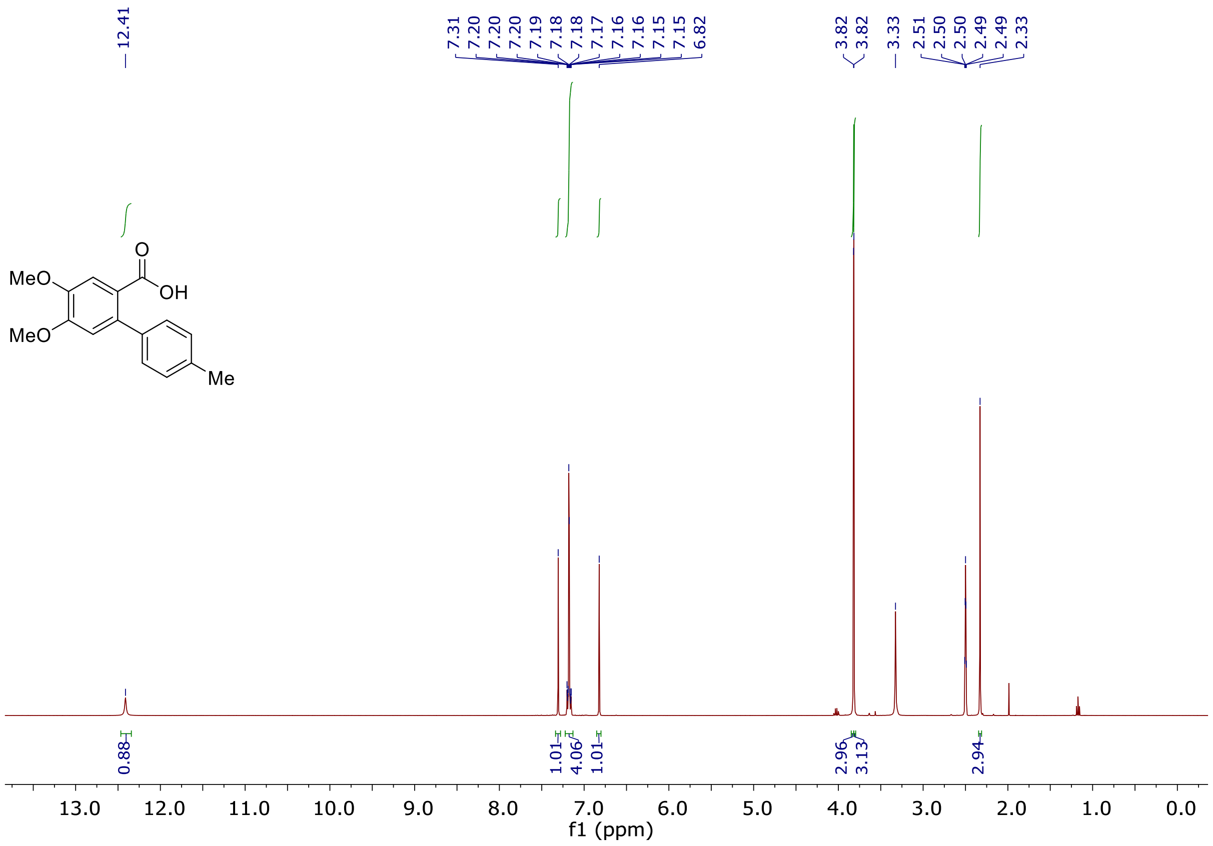

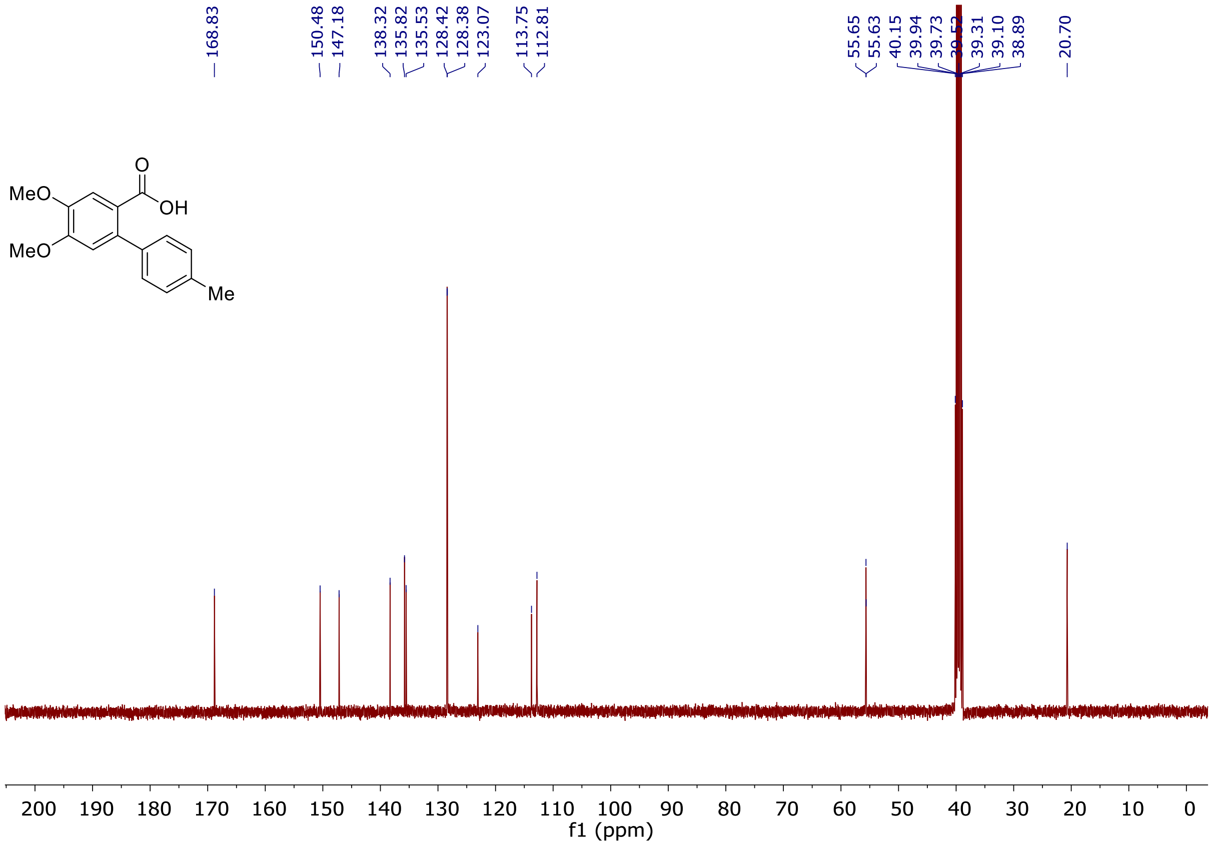

8,9-Dimethoxy-3-methyl-6H-benzo[c]chromen-6-one (**57**)

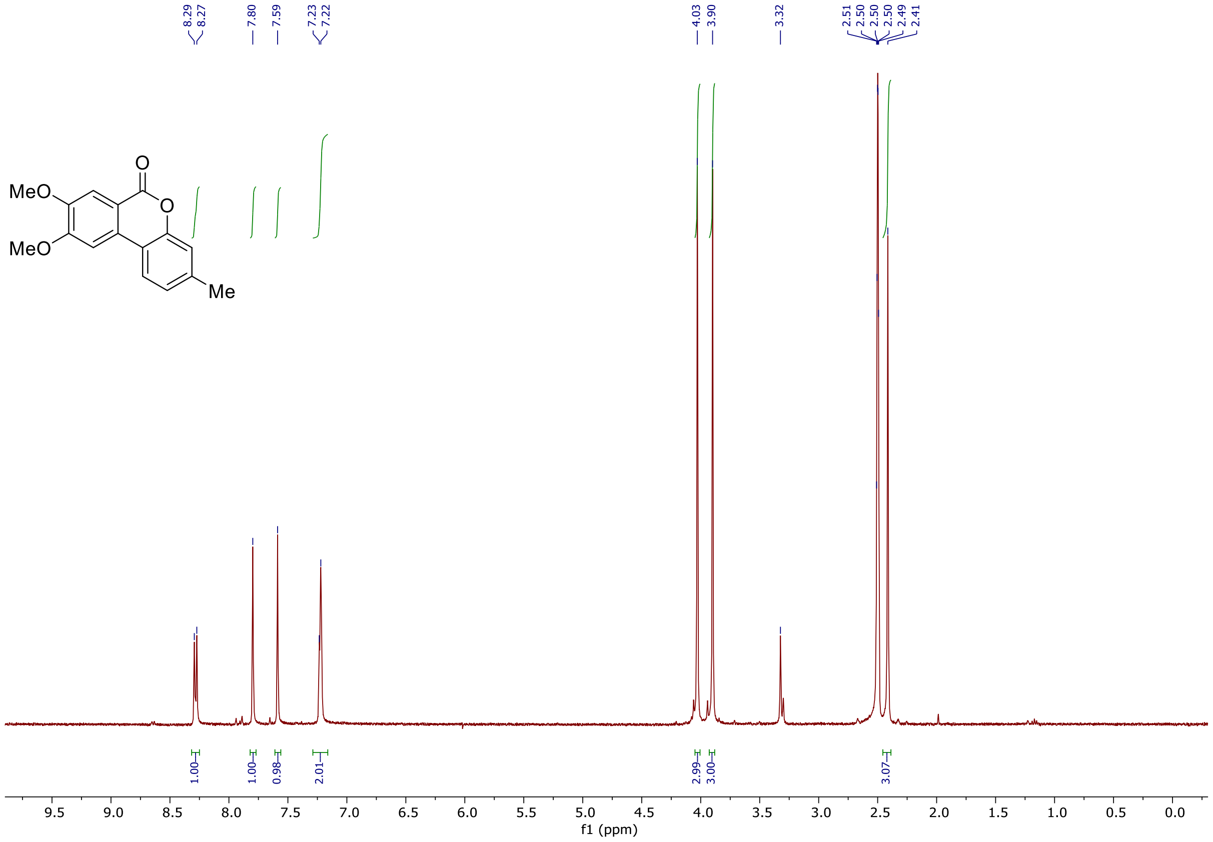

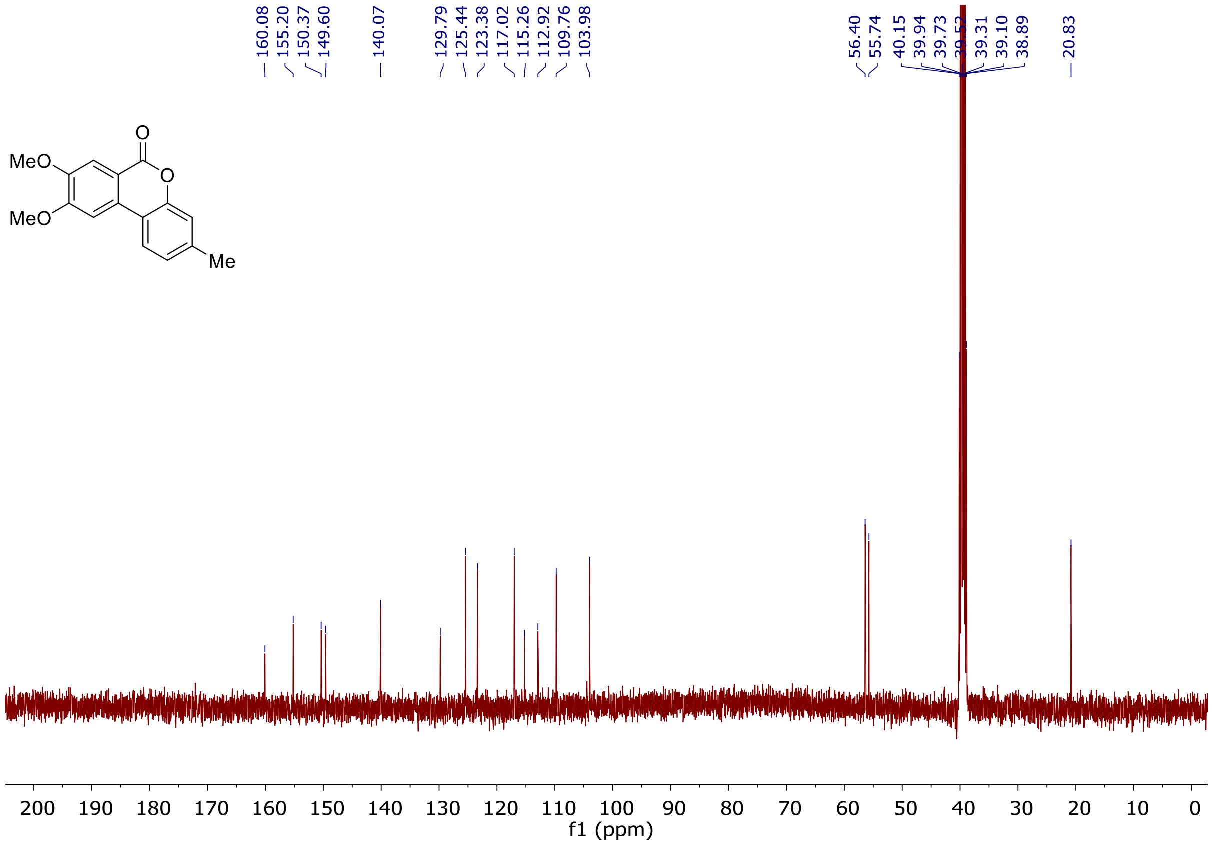

8,9-Dihydroxy-3-methyl-6H-benzo[c]chromen-6-one (**63**)

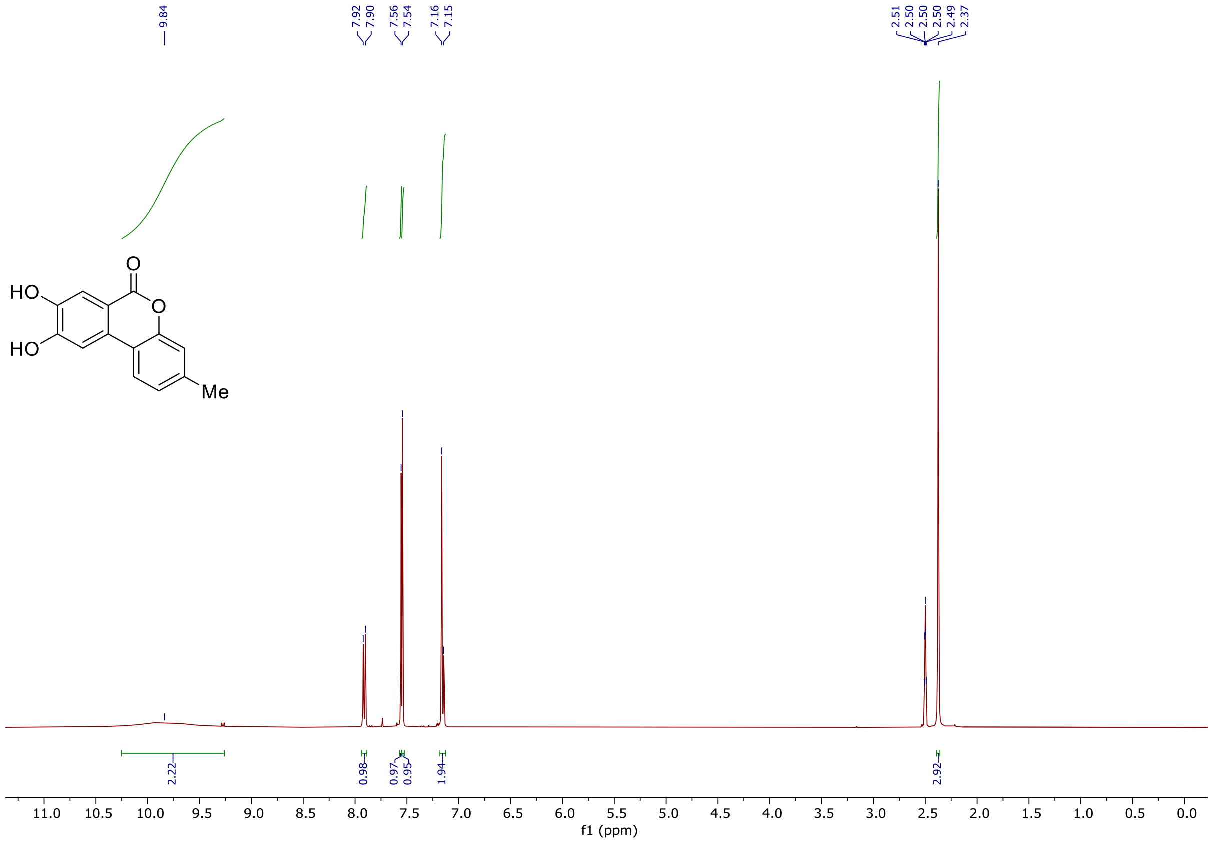

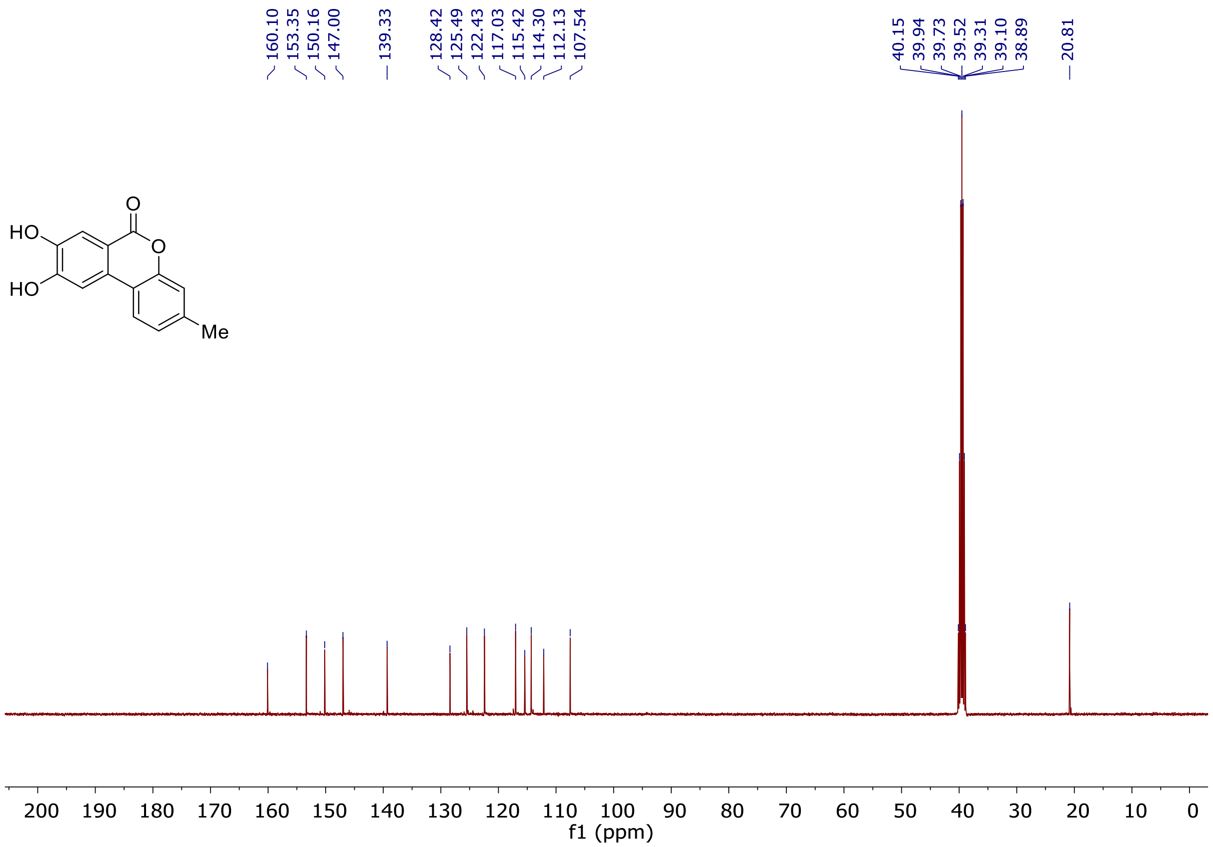

Methyl 4'-formyl-4,5-dimethoxy-[1,1'-biphenyl]-2-carboxylate (**46**)

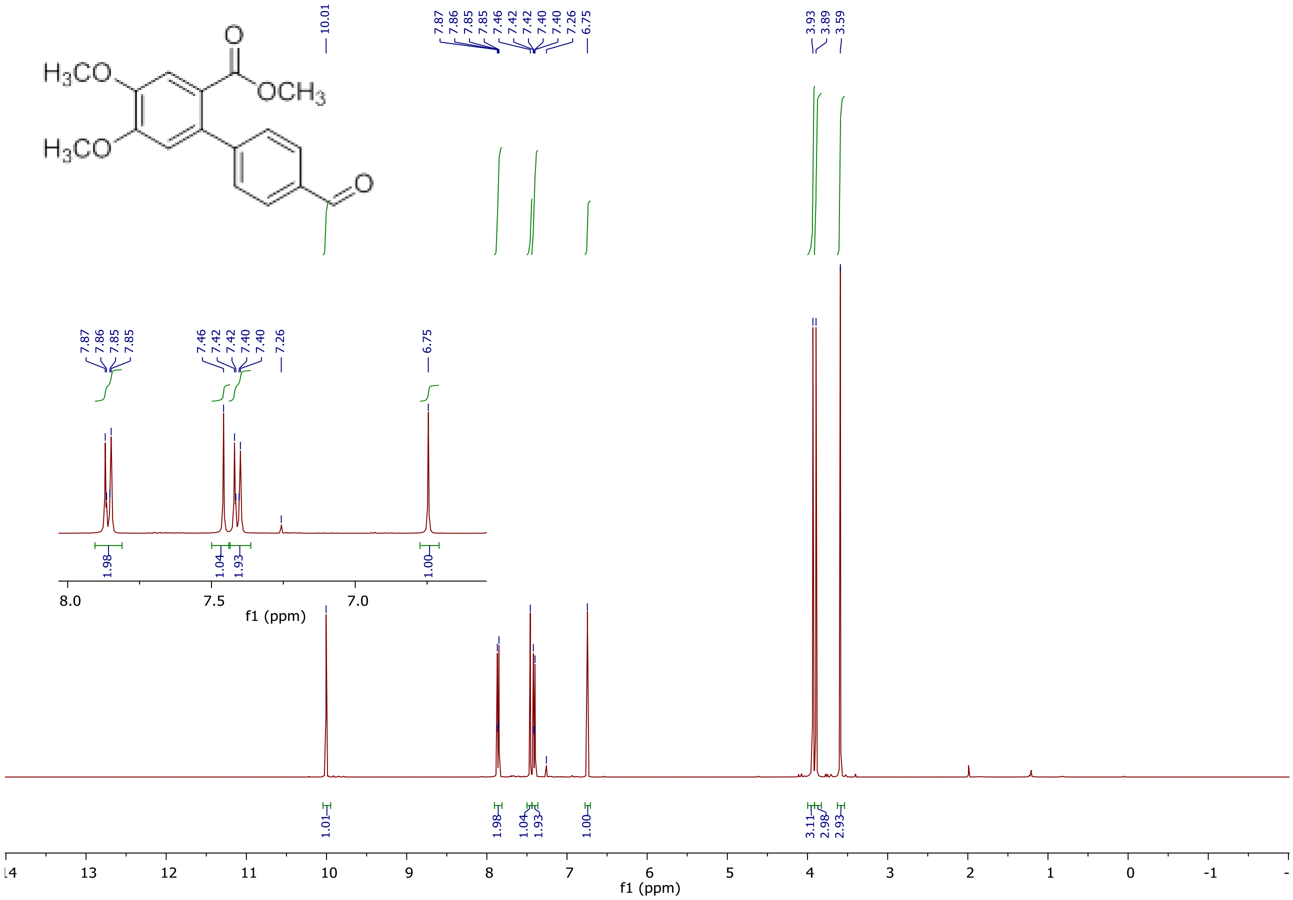

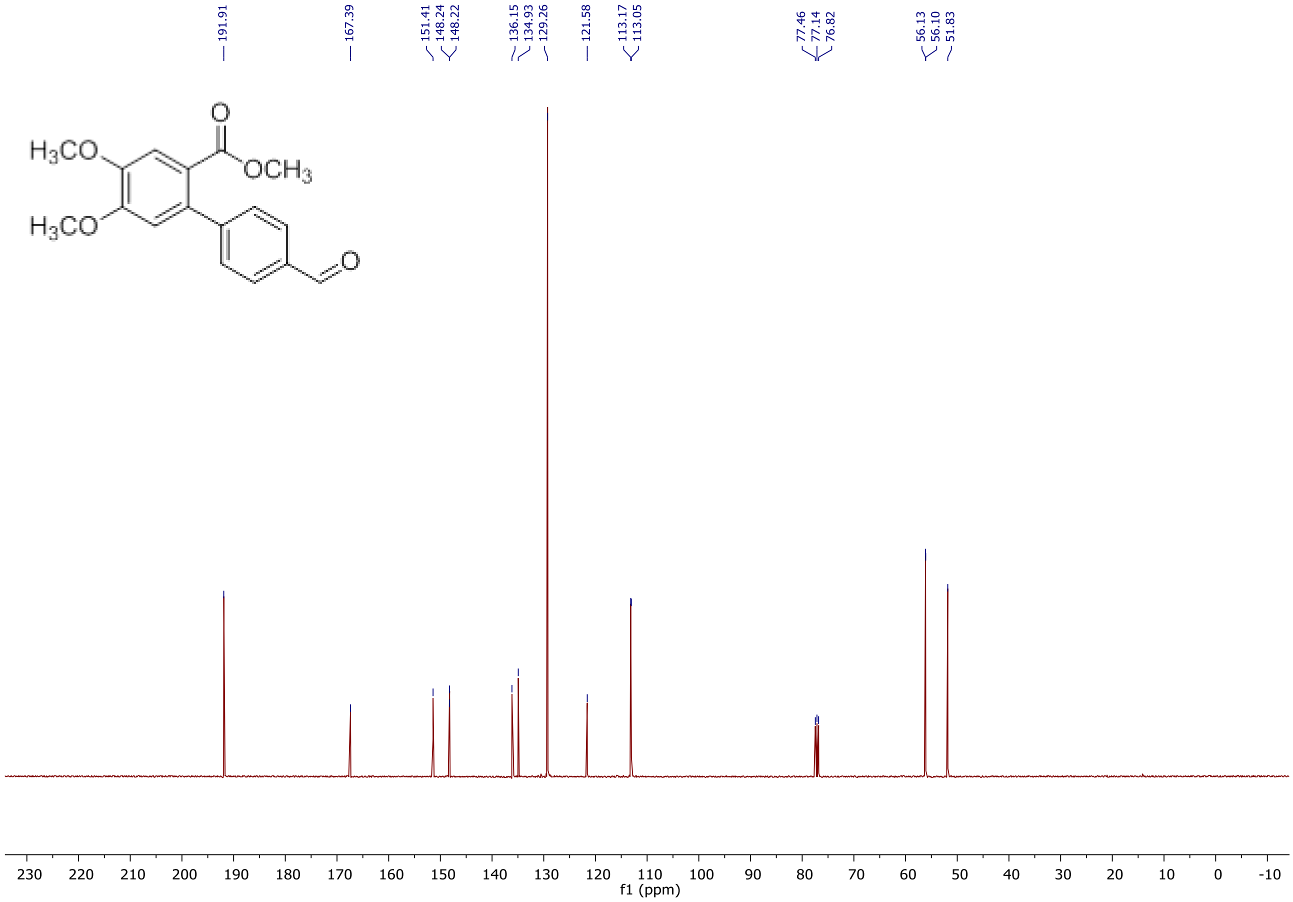

*4'-Formyl-4,5-dimethoxy-[1,1'-biphenyl]-2-carboxylic acid (**52**)*

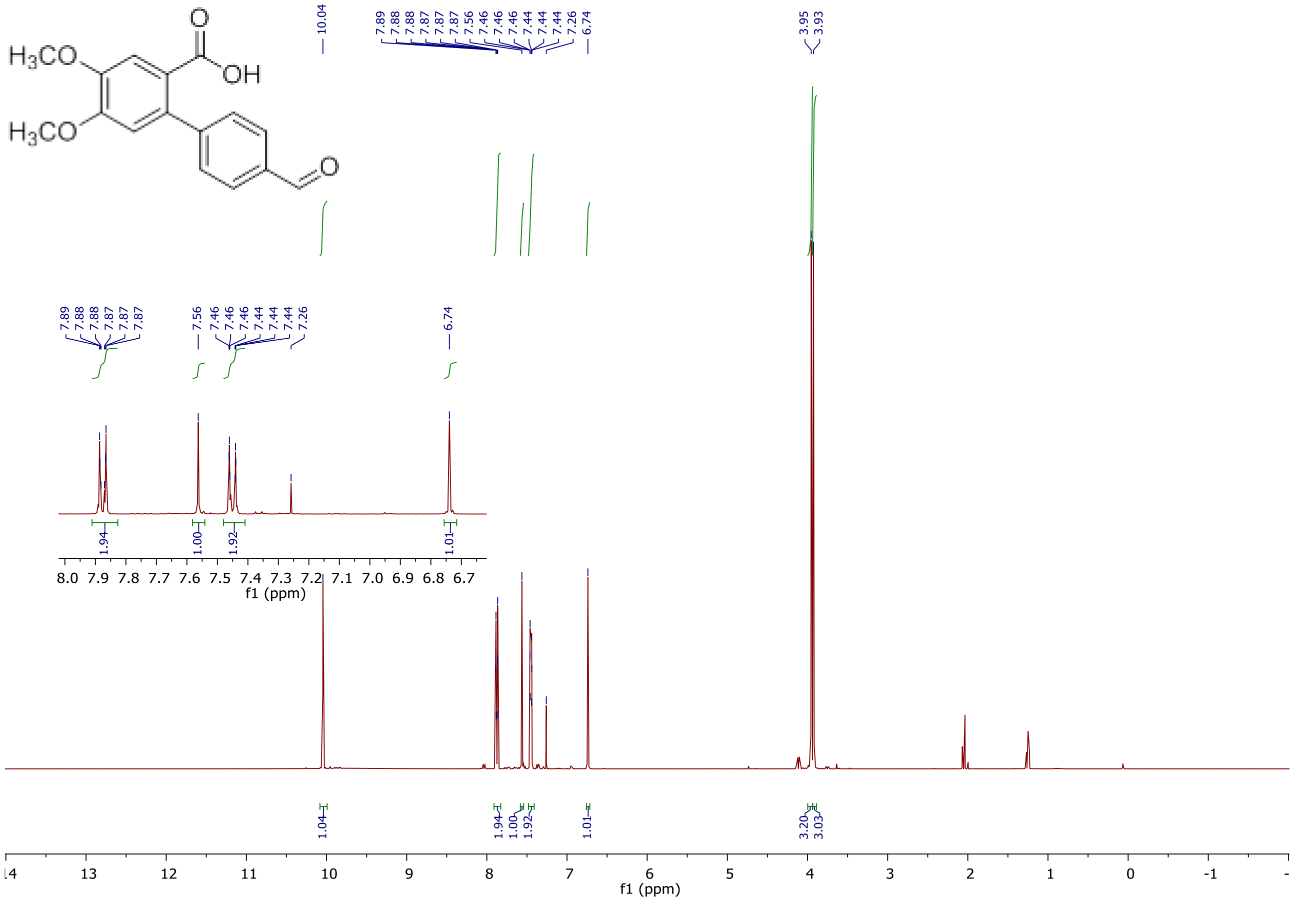

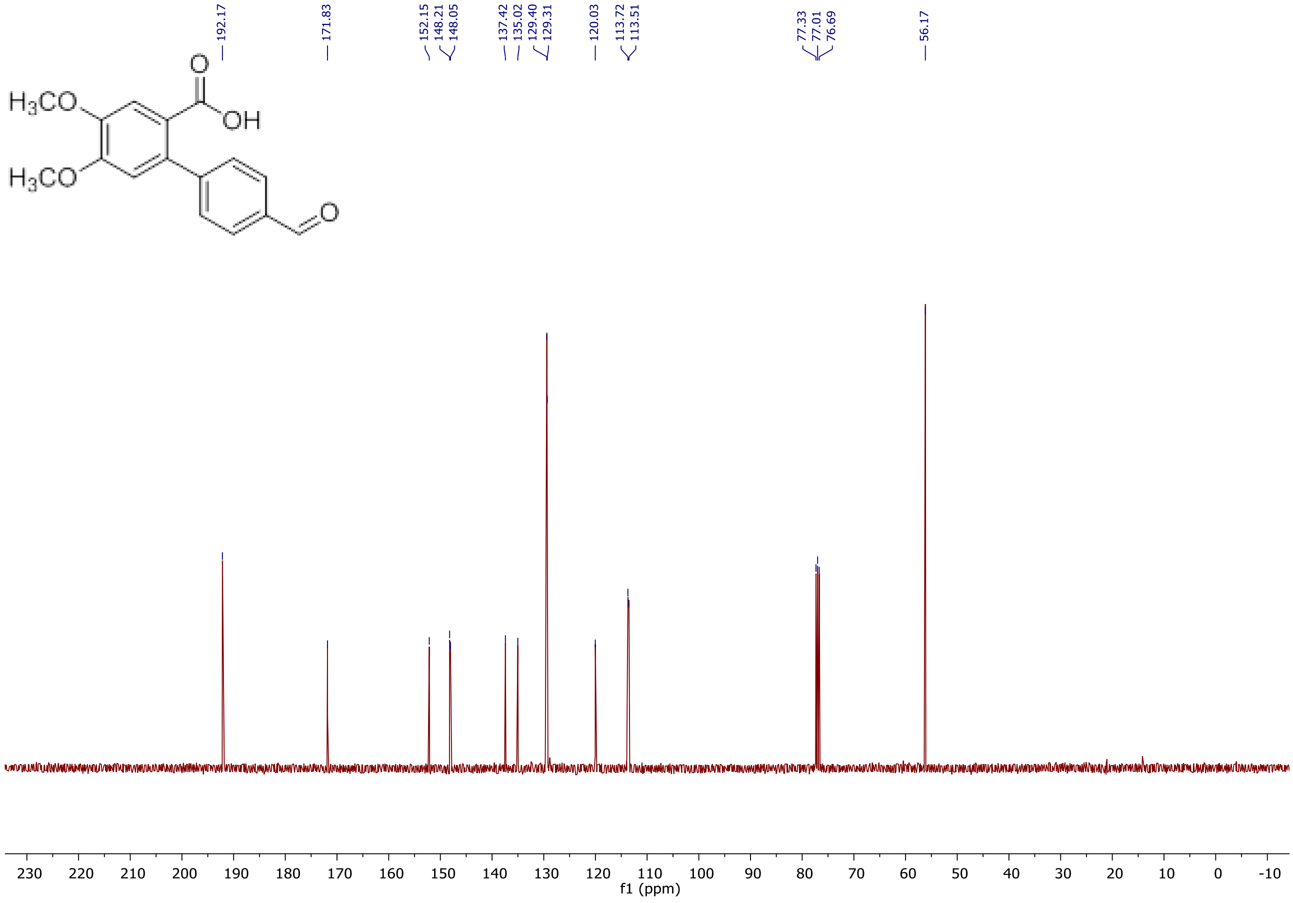

8,9-Dimethoxy-6-oxo-6H-benzo[c]chromene-3-carbaldehyde (**58**)

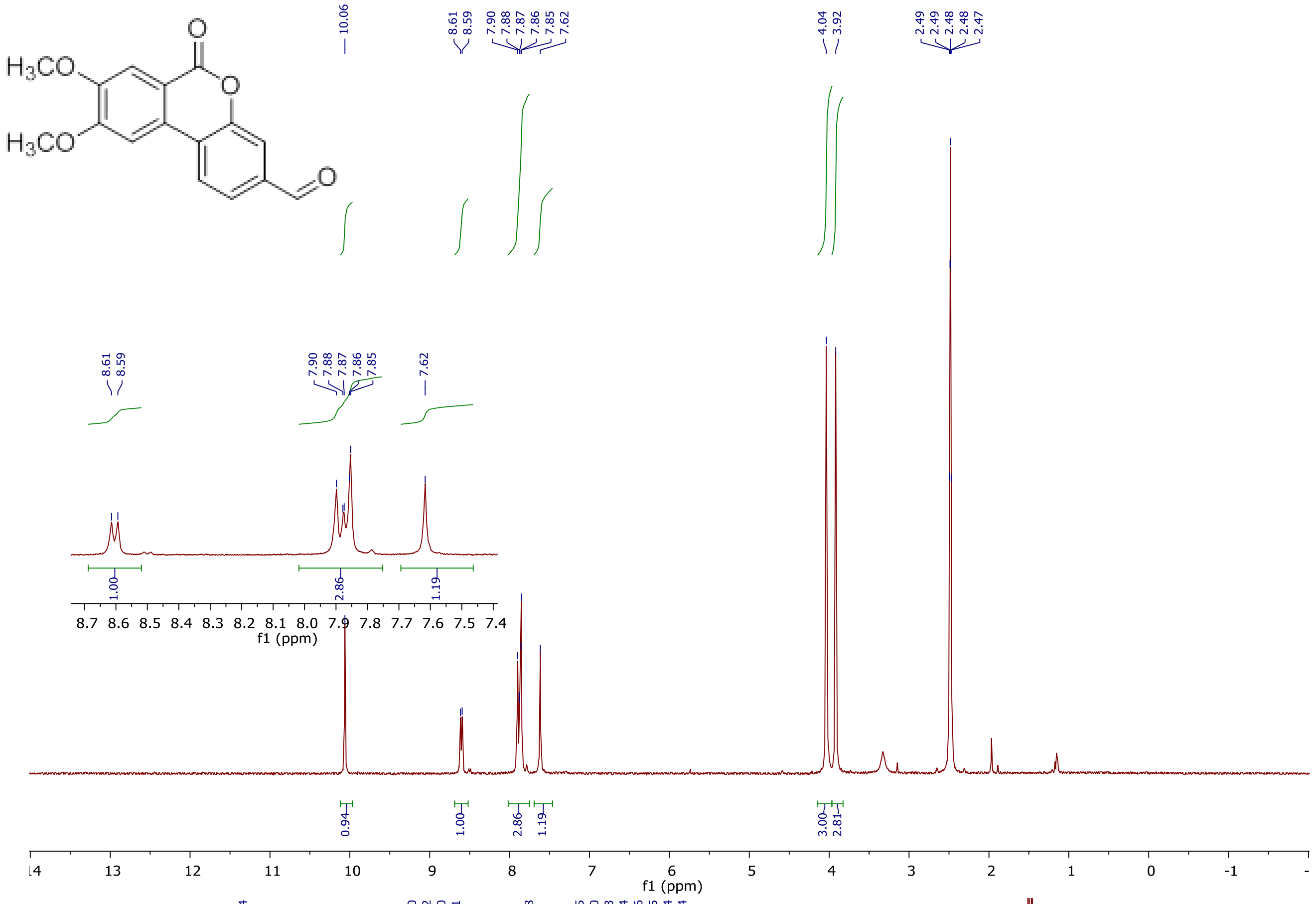

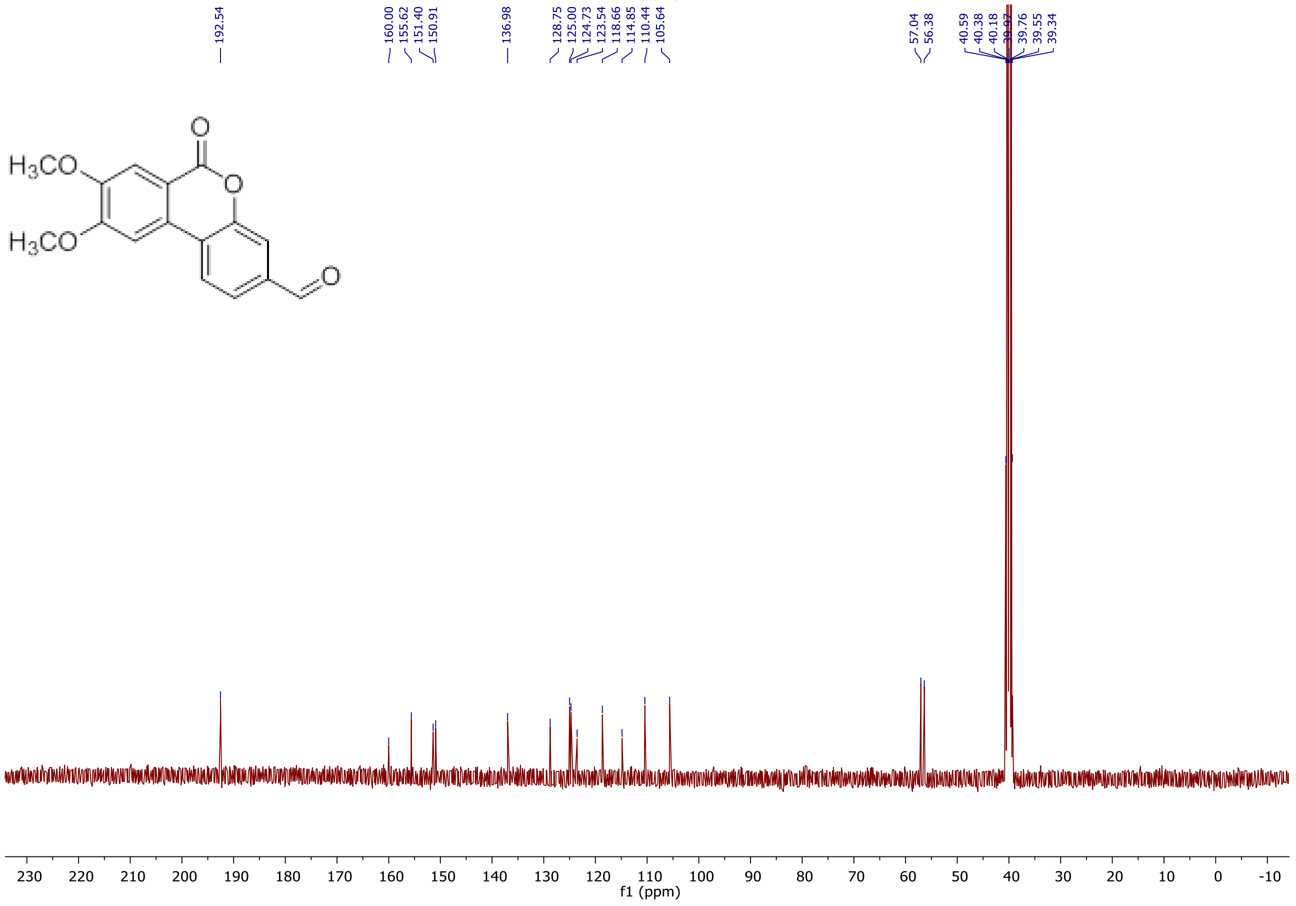

8,9-Dihydroxy-6-oxo-6H-benzo[c]chromene-3-carbaldehyde (***64***)

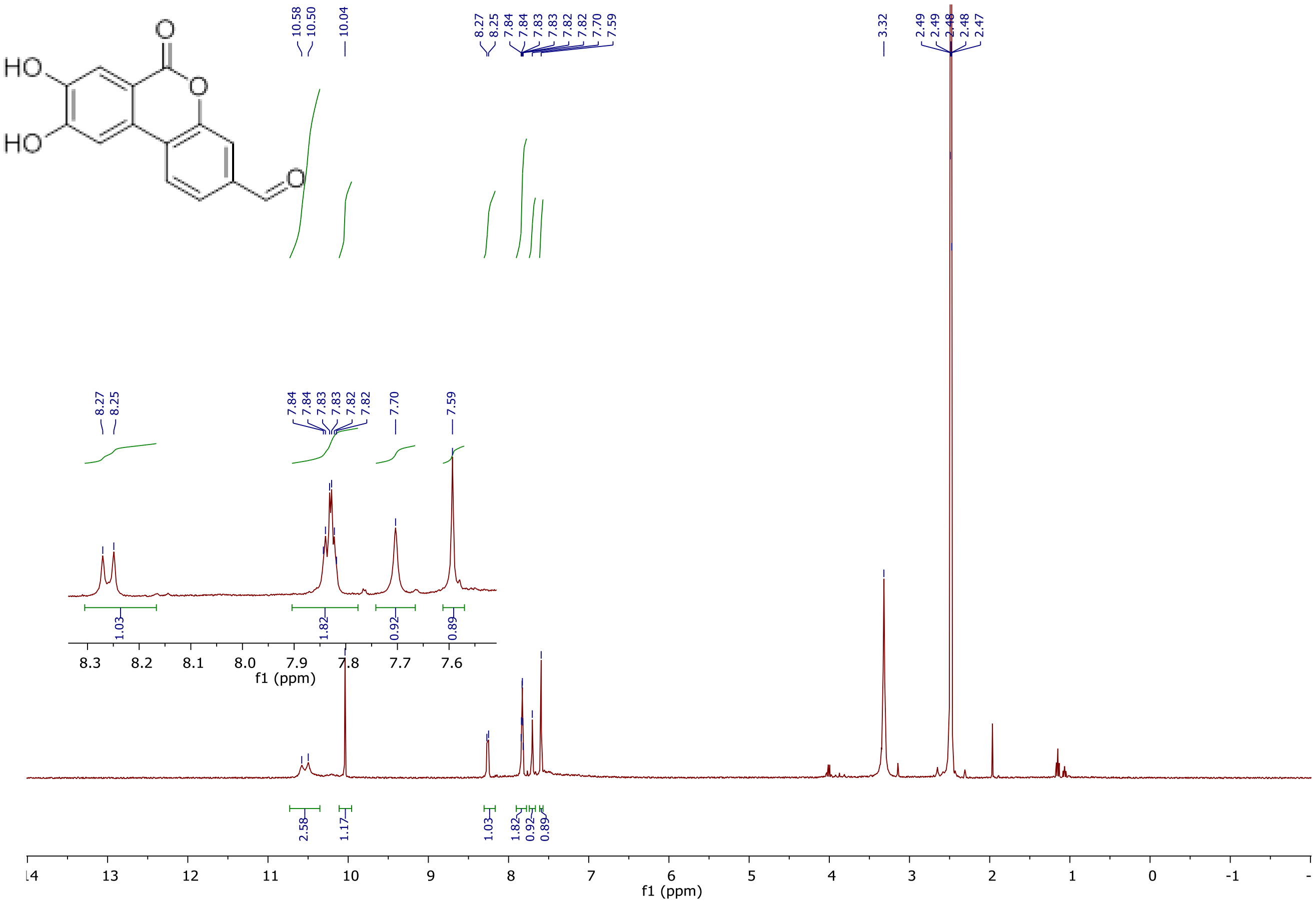

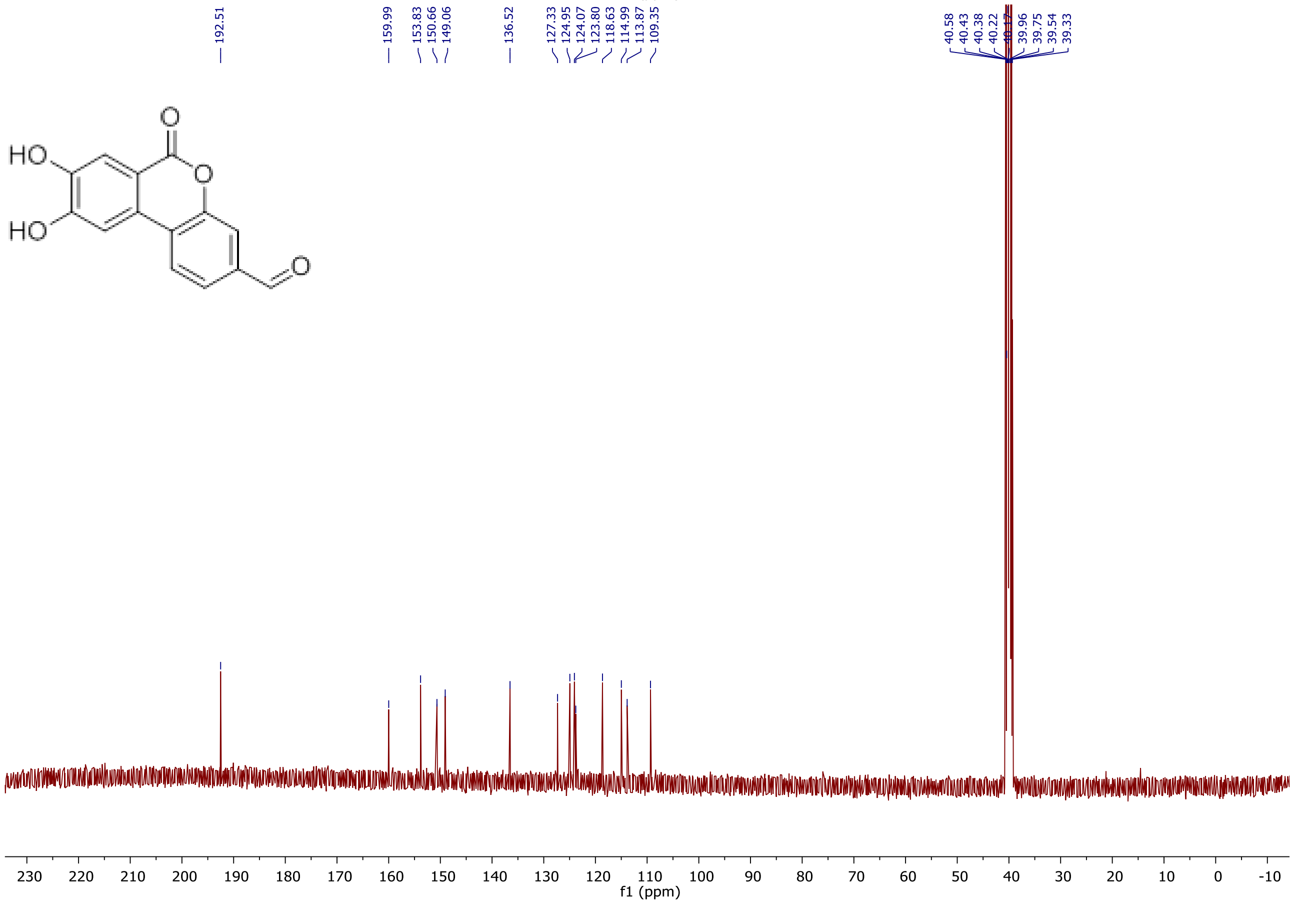

Methyl 4,5-dimethoxy-4'-(methylsulfonyl)-[1,1'-biphenyl]-2-carboxylate (**47**)

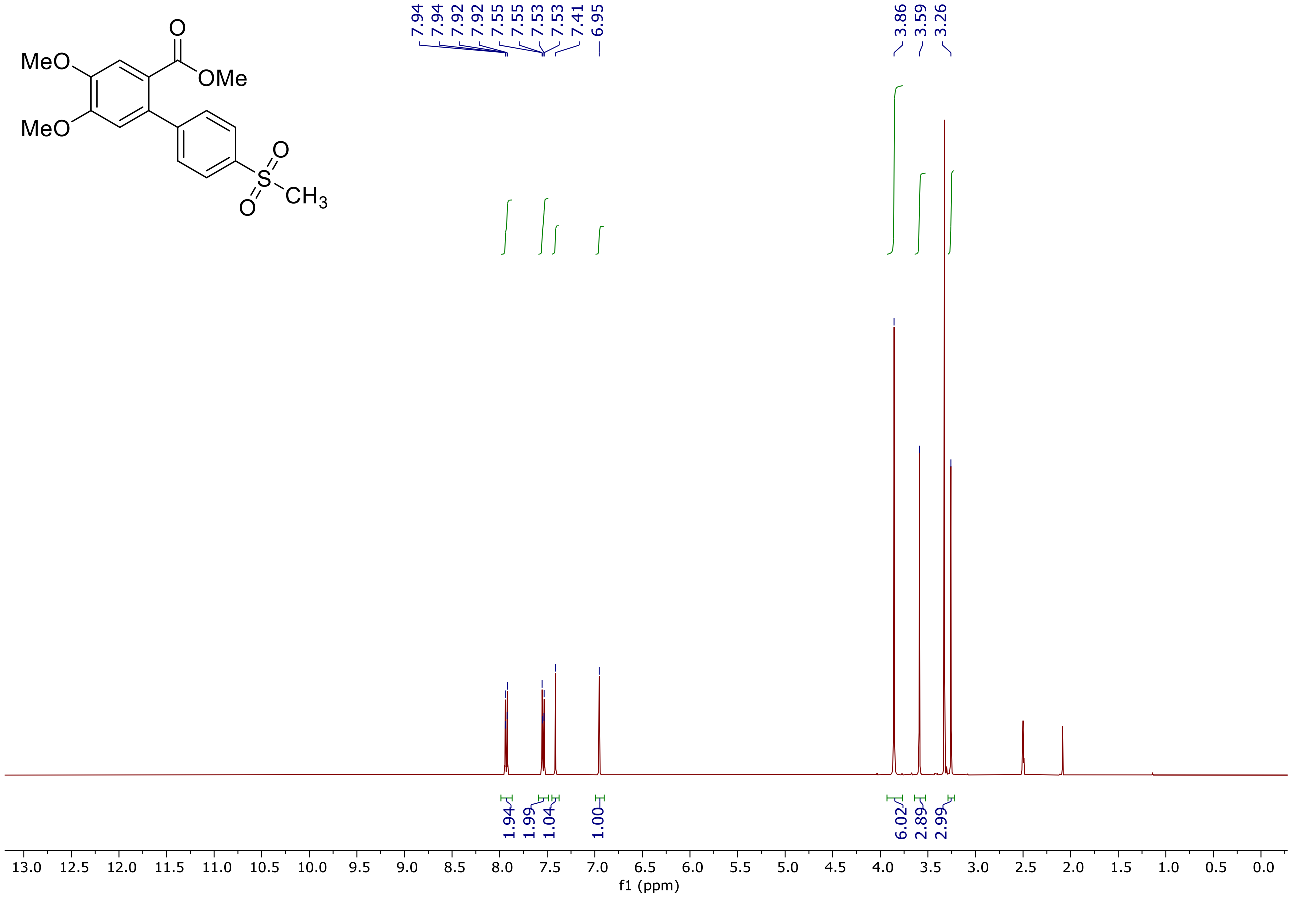

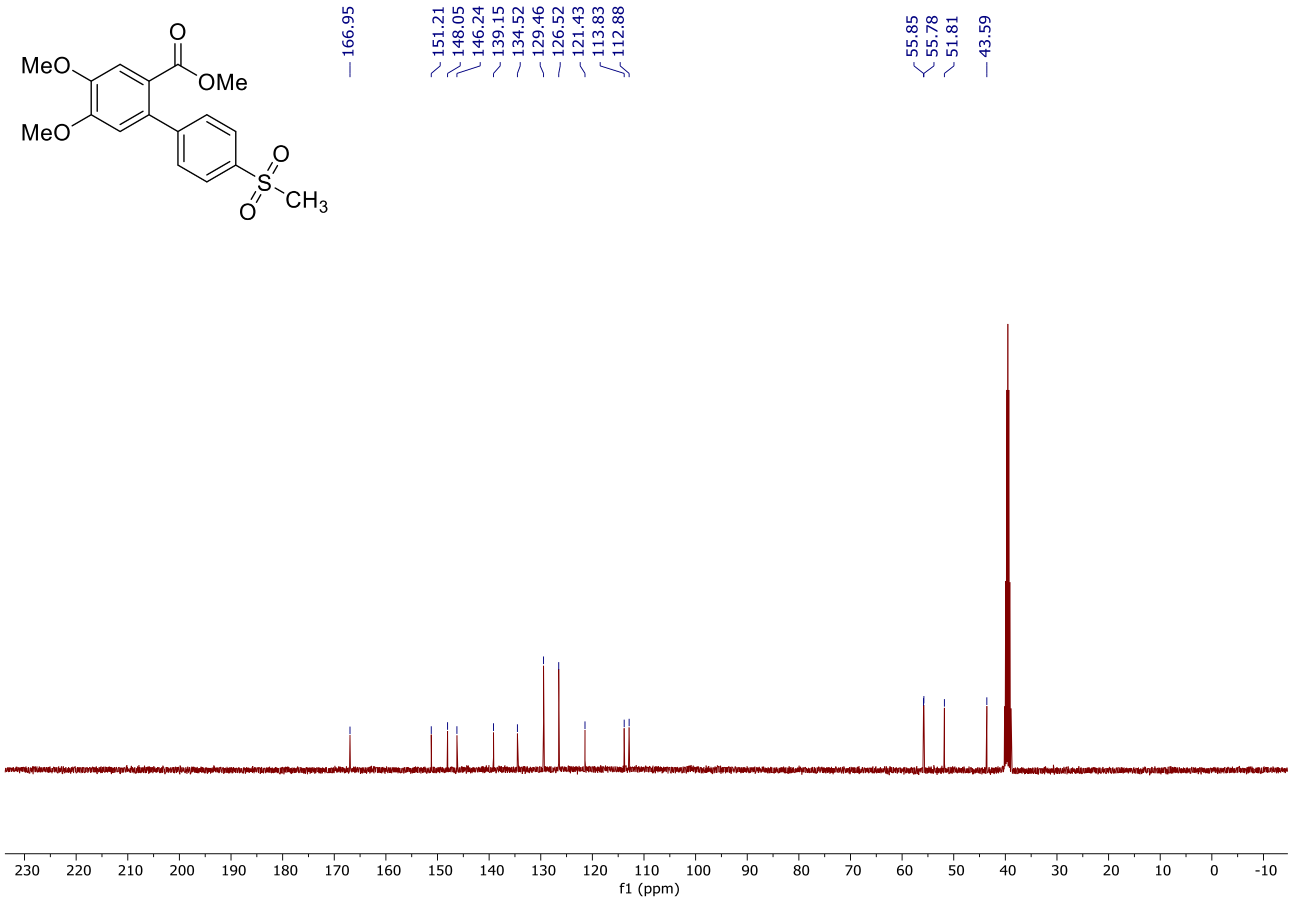

4,5-Dimethoxy-4'-(methylsulfonyl)-[1,1'-biphenyl]-2-carboxylic acid (**53**)

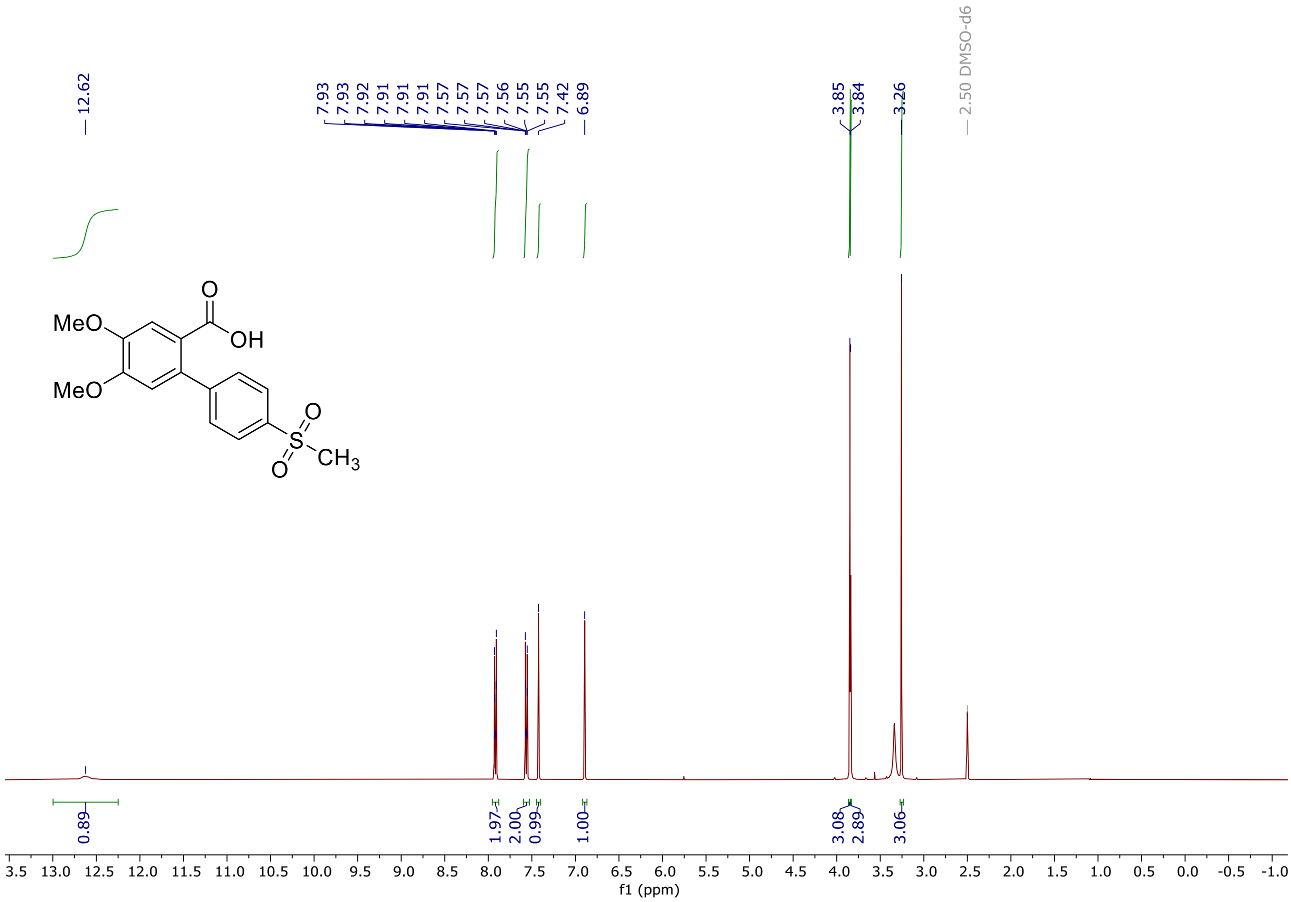

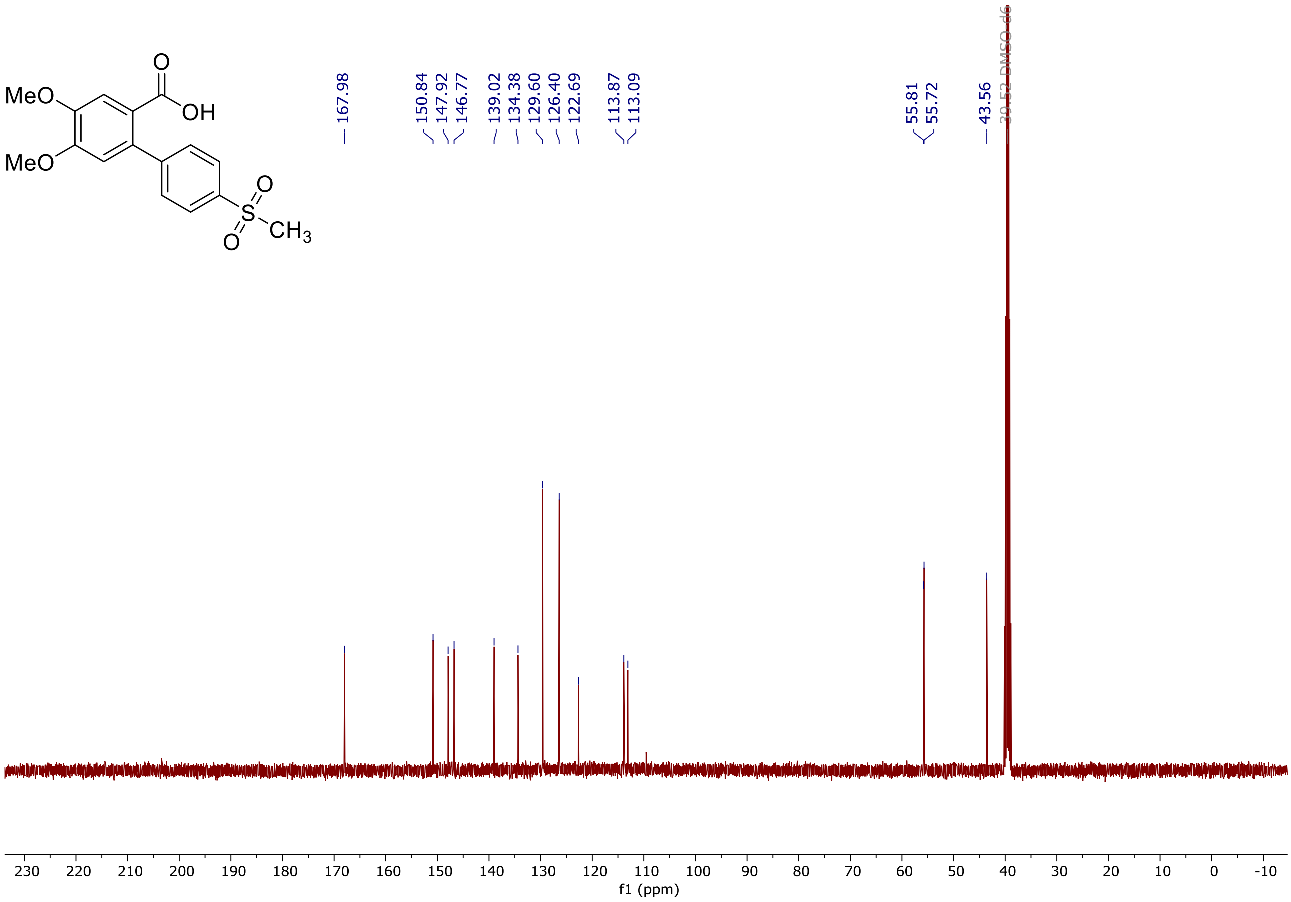

8,9-Dimethoxy-3-(methylsulfonyl)-6H-benzo[c]chromen-6-one (**59**)

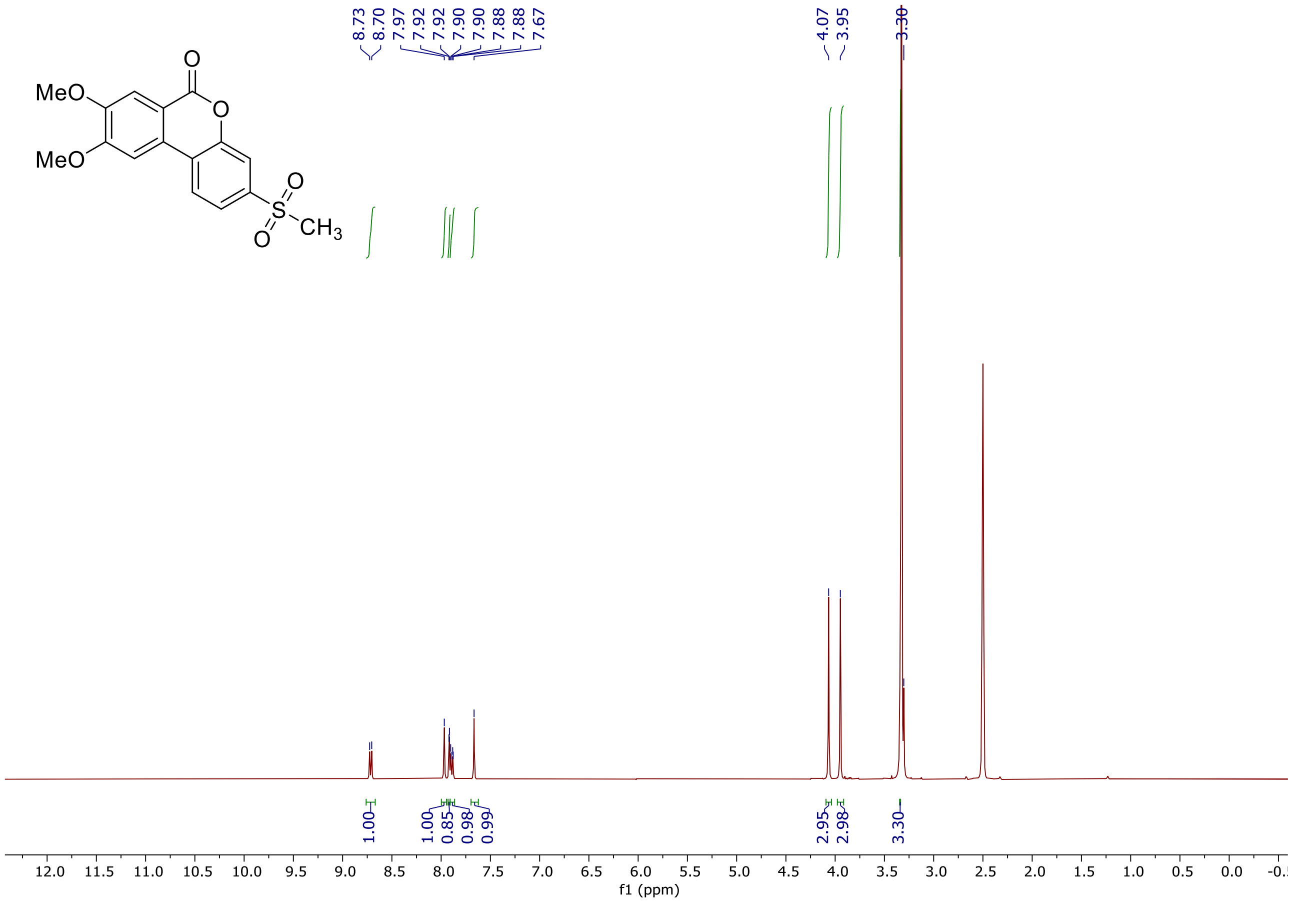

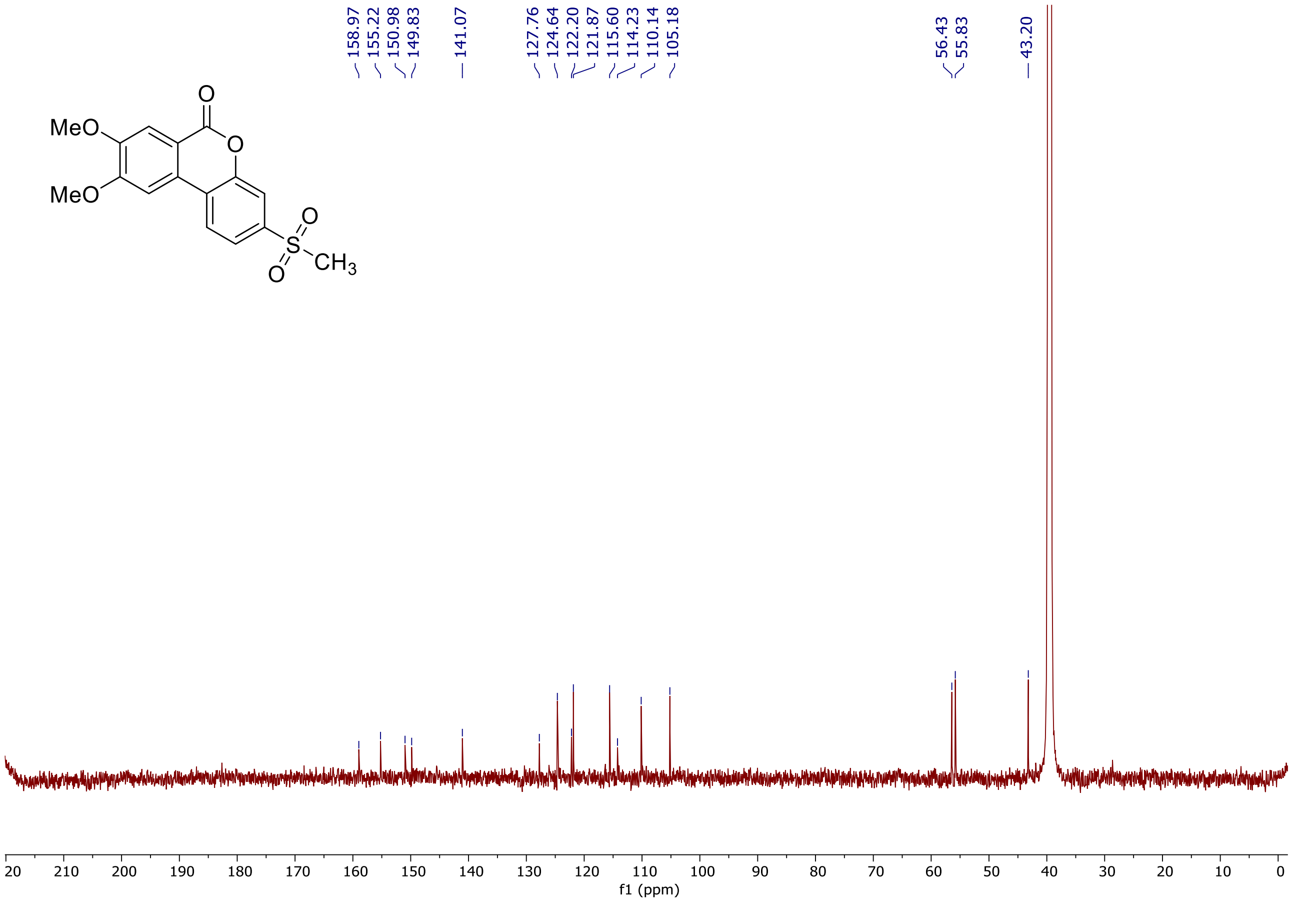

8,9-Dihydroxy-3-(methylsulfonyl)-6H-benzo[c]chromen-6-one (**65**)

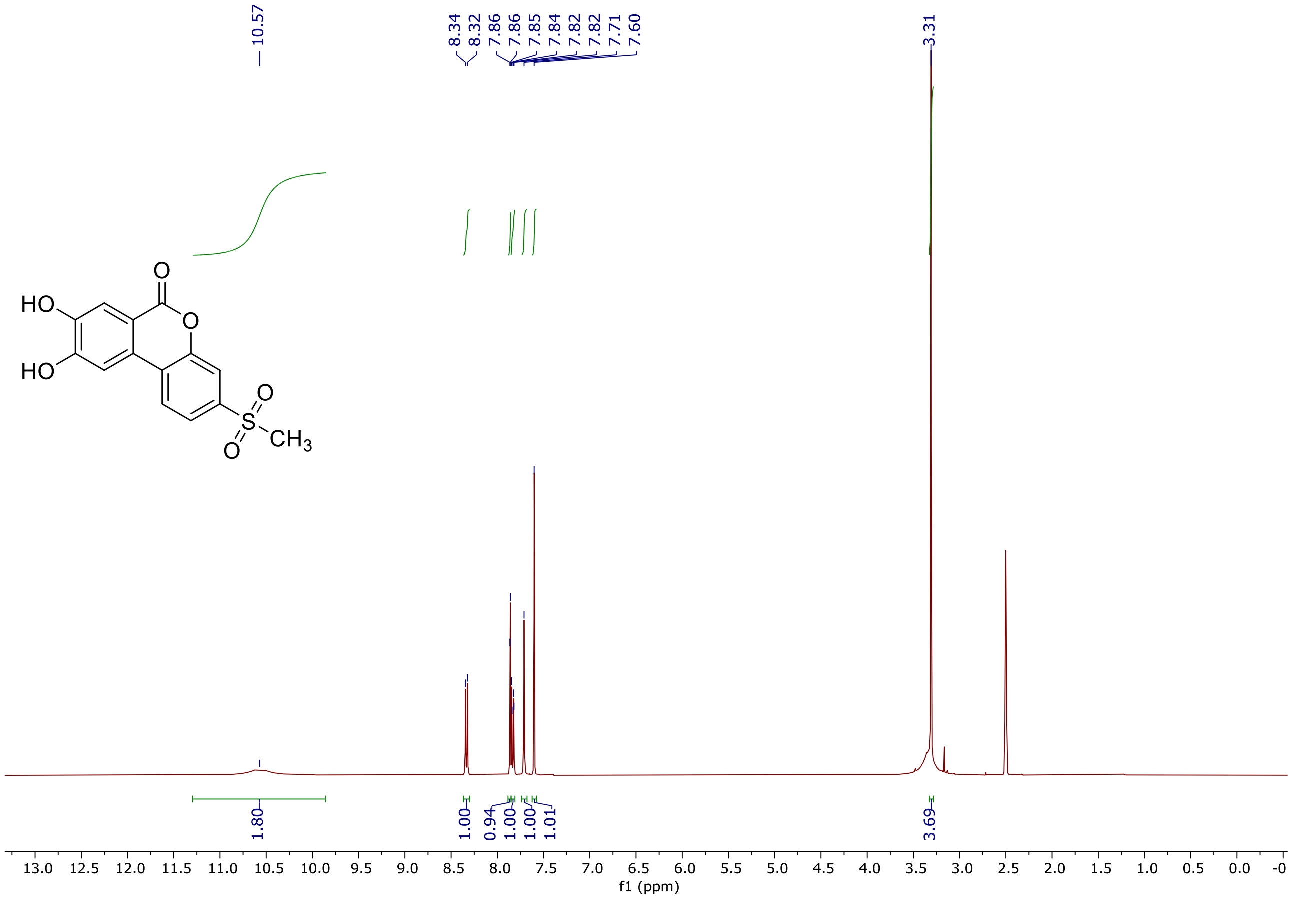

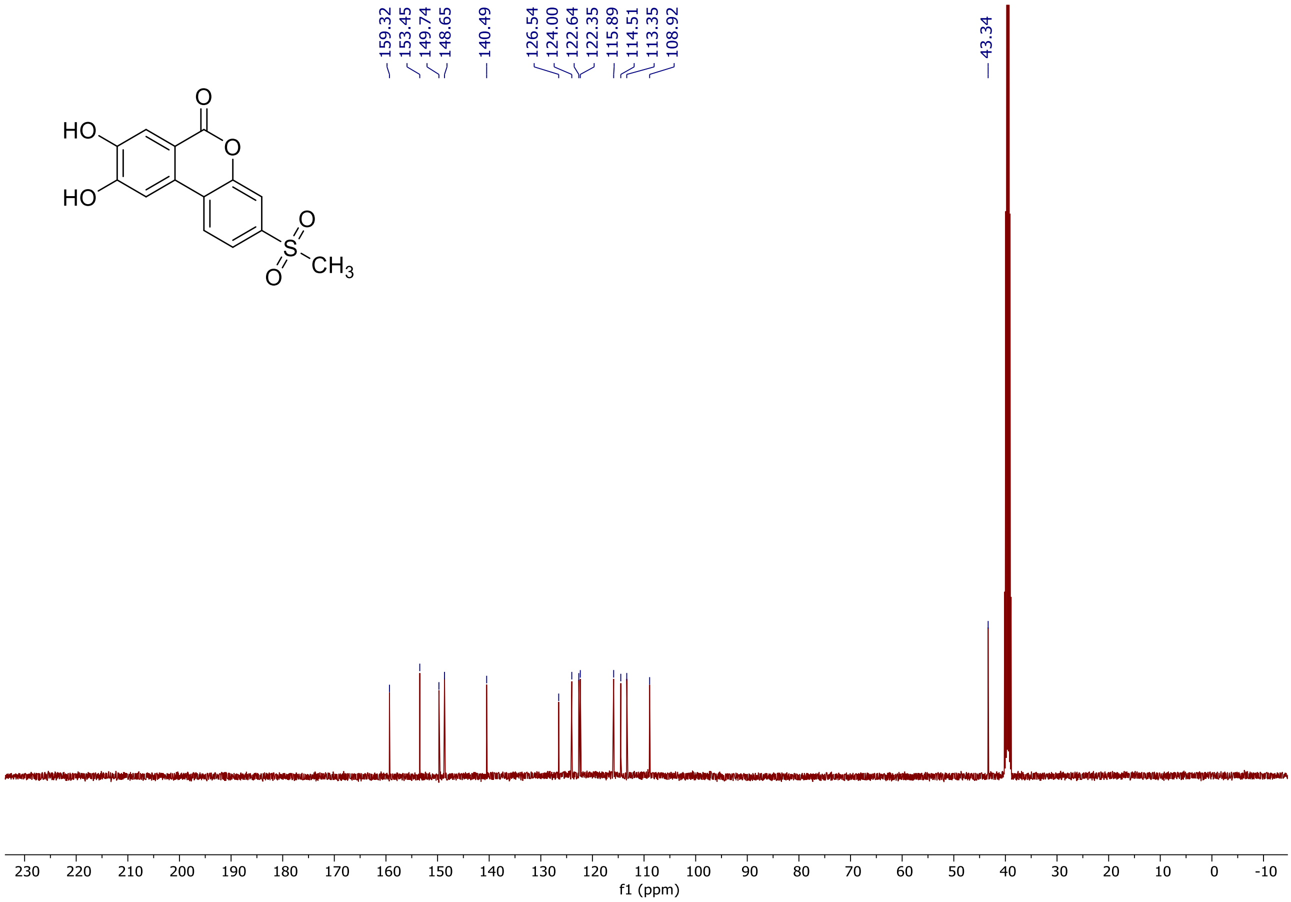

Methyl 4,5-dimethoxy-4'-sulfamoyl-[1,1'-biphenyl]-2-carboxylate (**48**)

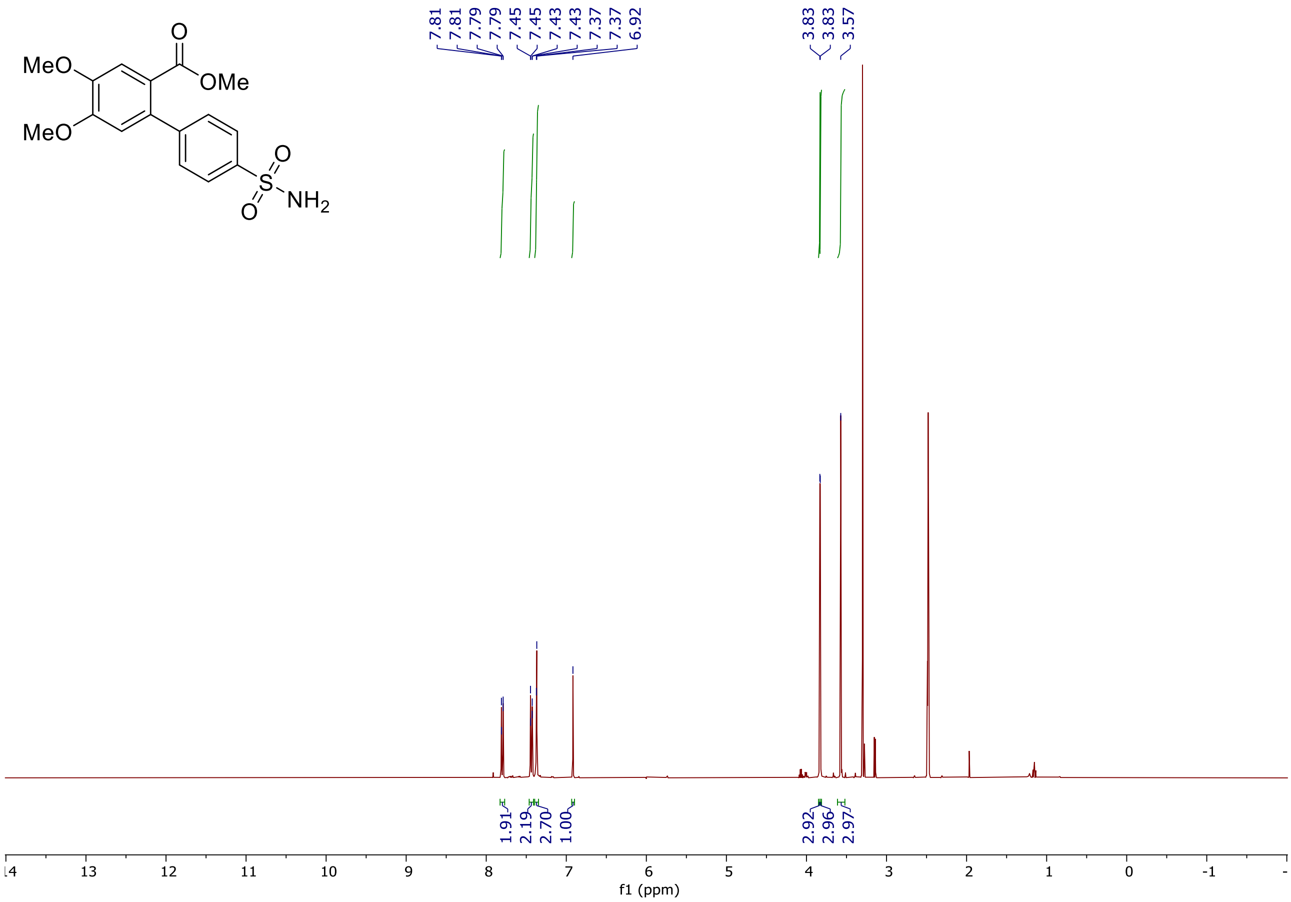

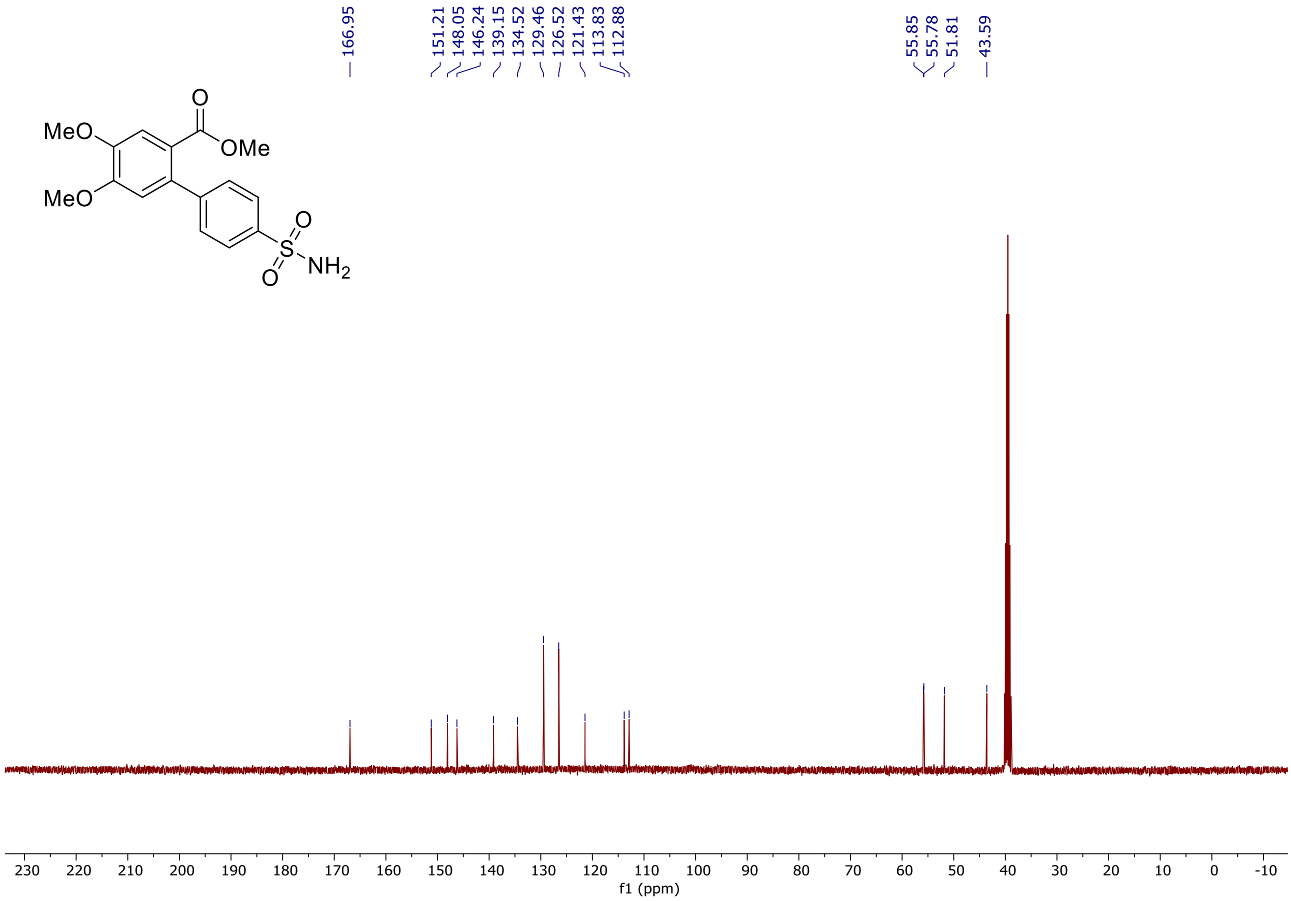

4,5-Dimethoxy-4'-sulfamoyl-[1,1'-biphenyl]-2-carboxylic acid (**54**)

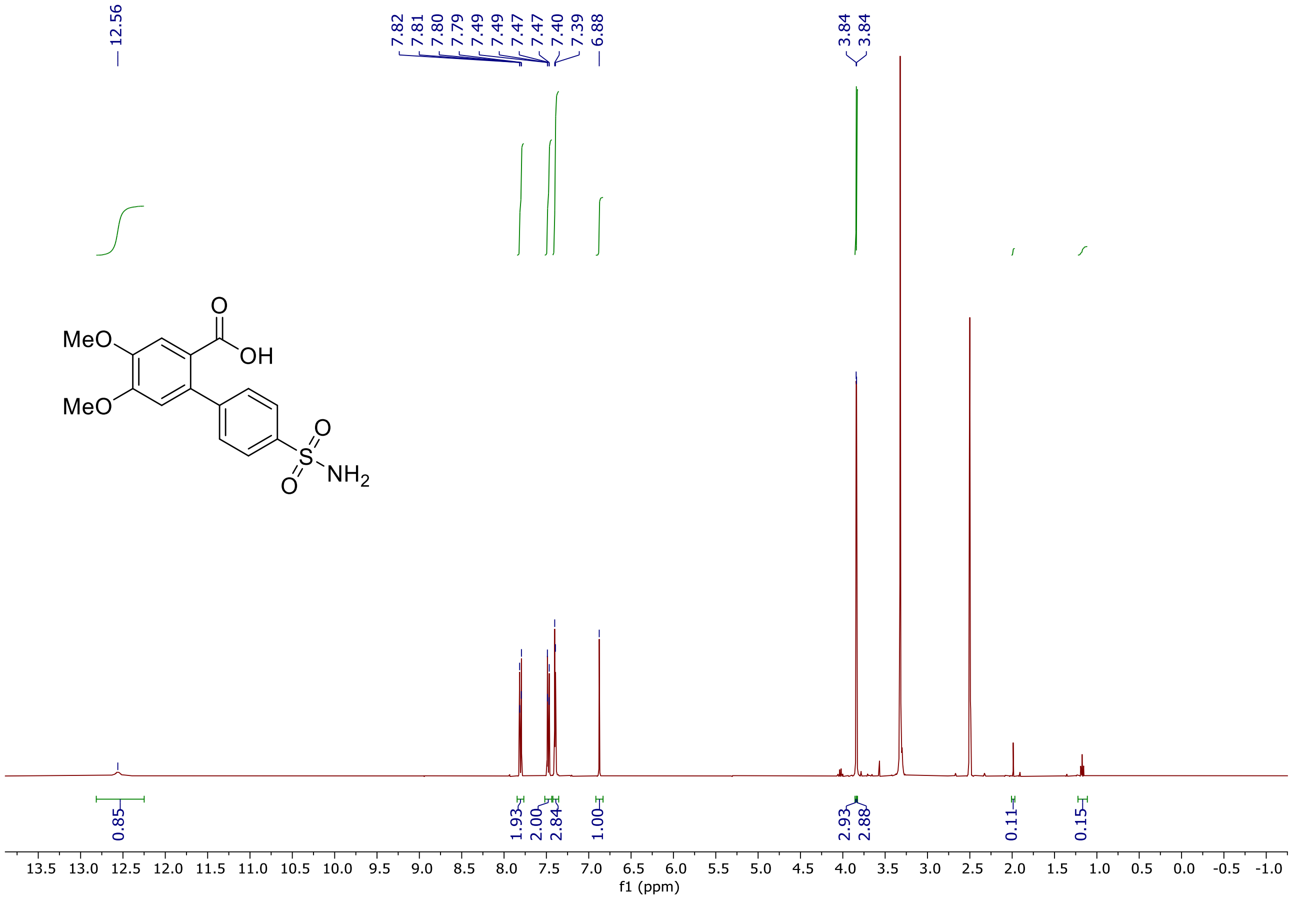

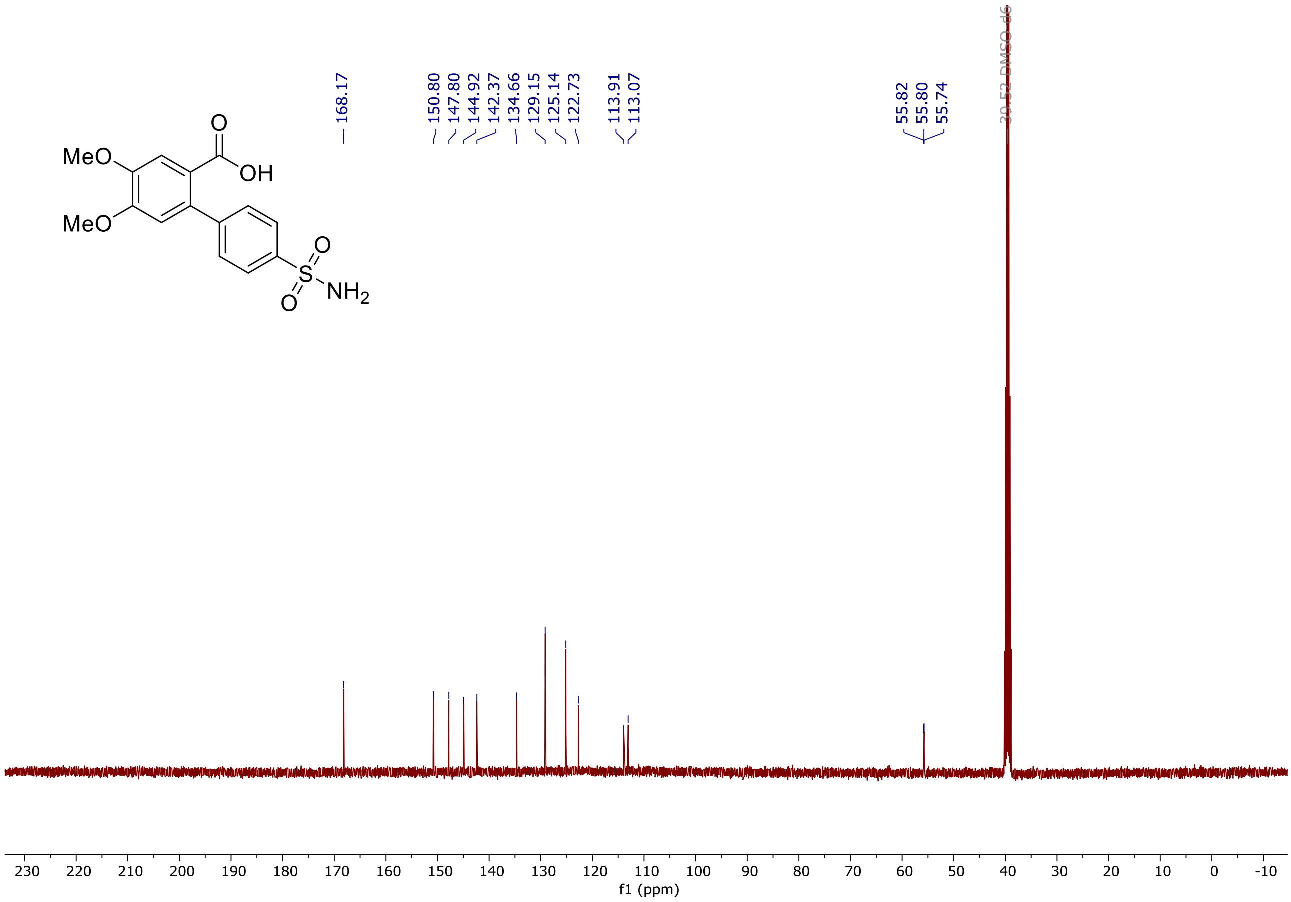

8,9-Dimethoxy-6-oxo-6H-benzo[c]chromene-3-sulfonamide (**60**)

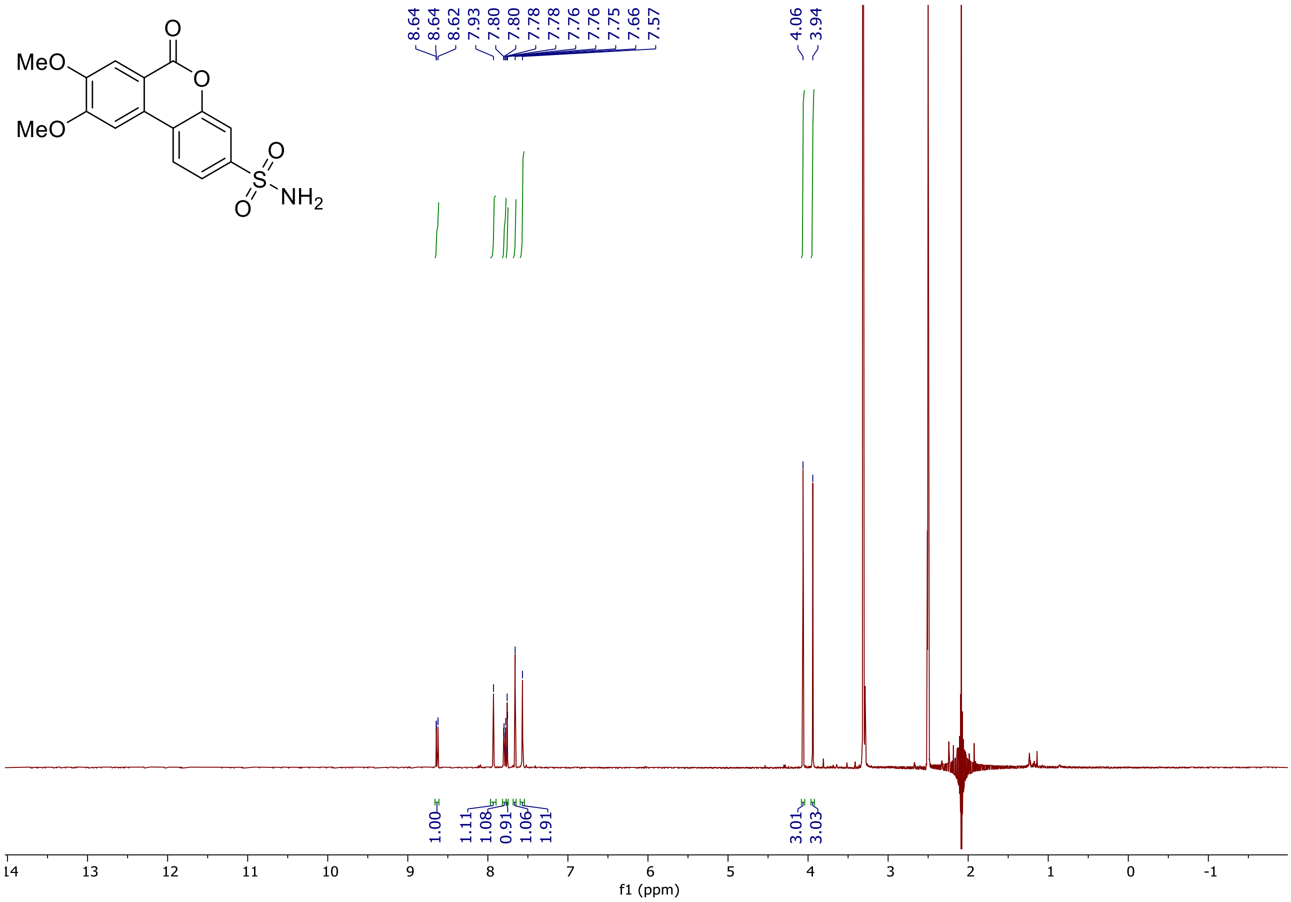

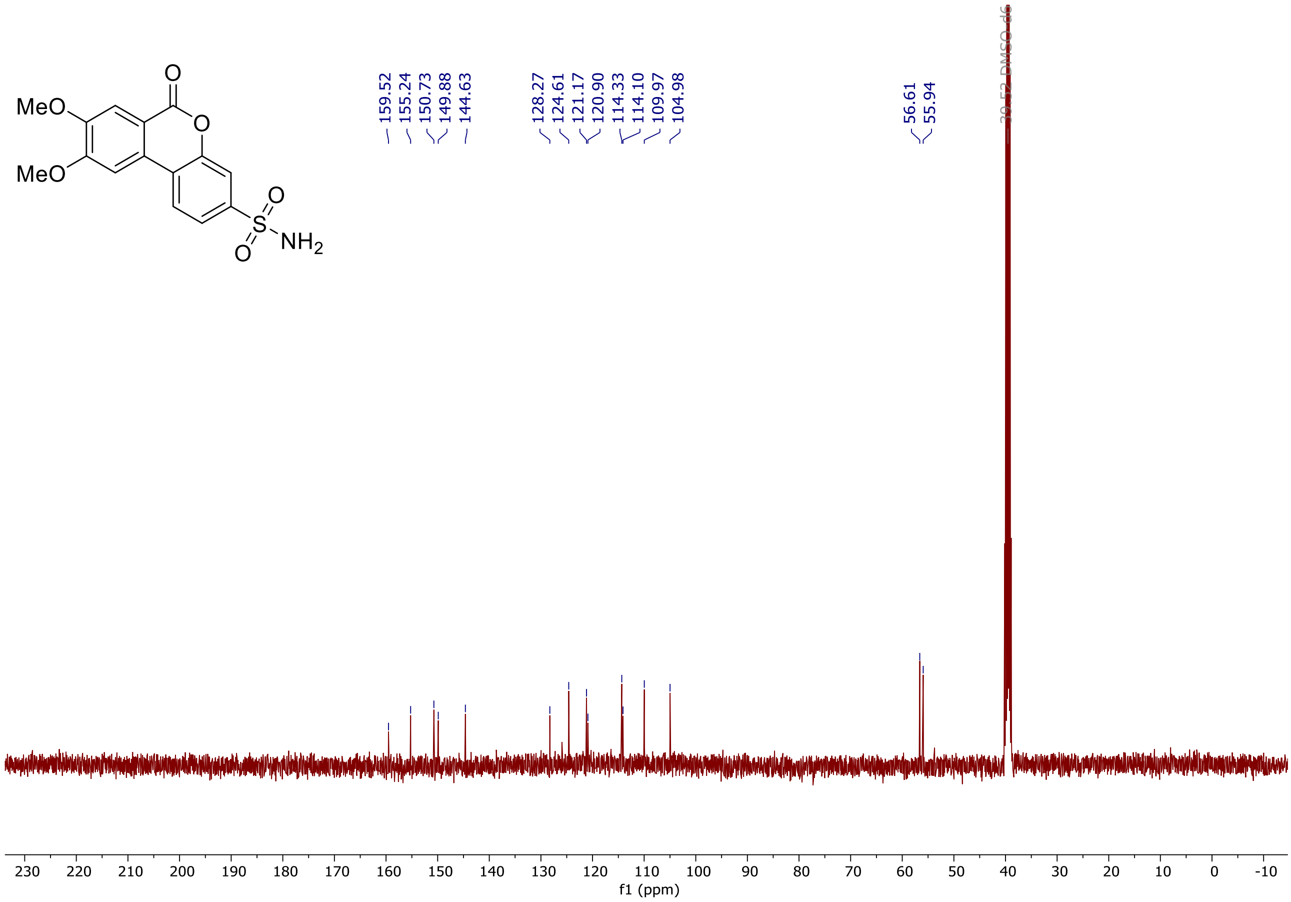

8,9-Dihydroxy-6-oxo-6H-benzo[c]chromene-3-sulfonamide (**66**)

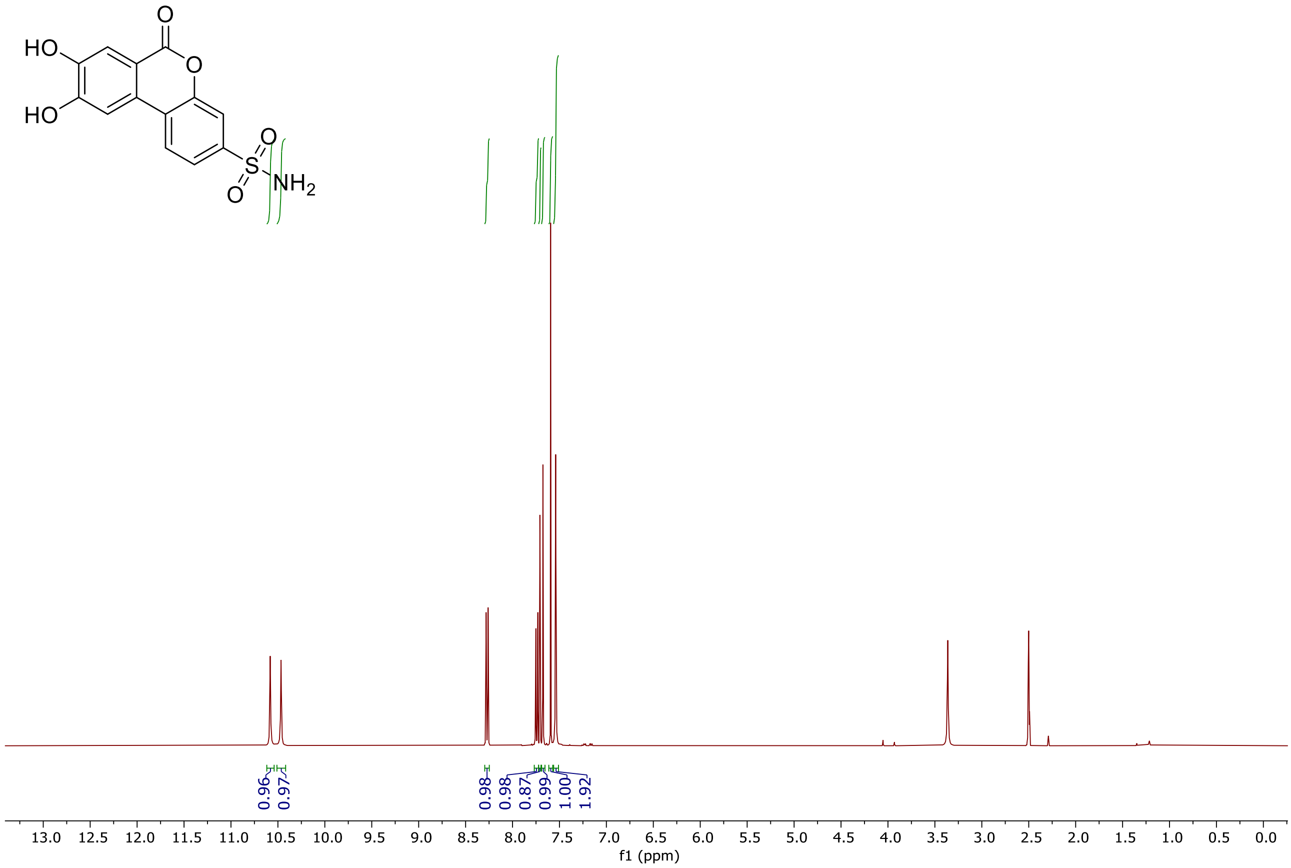

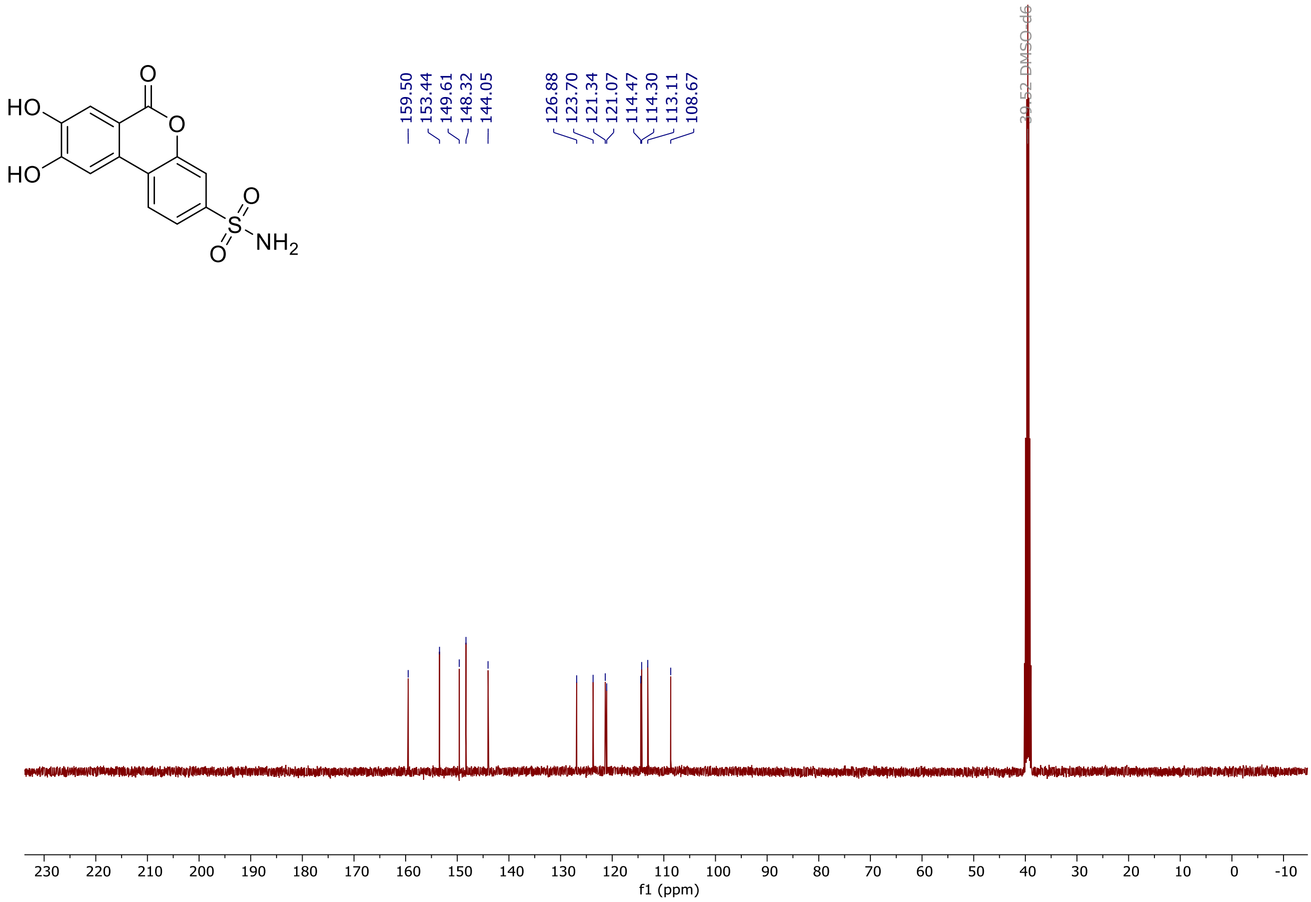

2-Bromo-4,5-dimethoxybenzaldehyde (**68**)

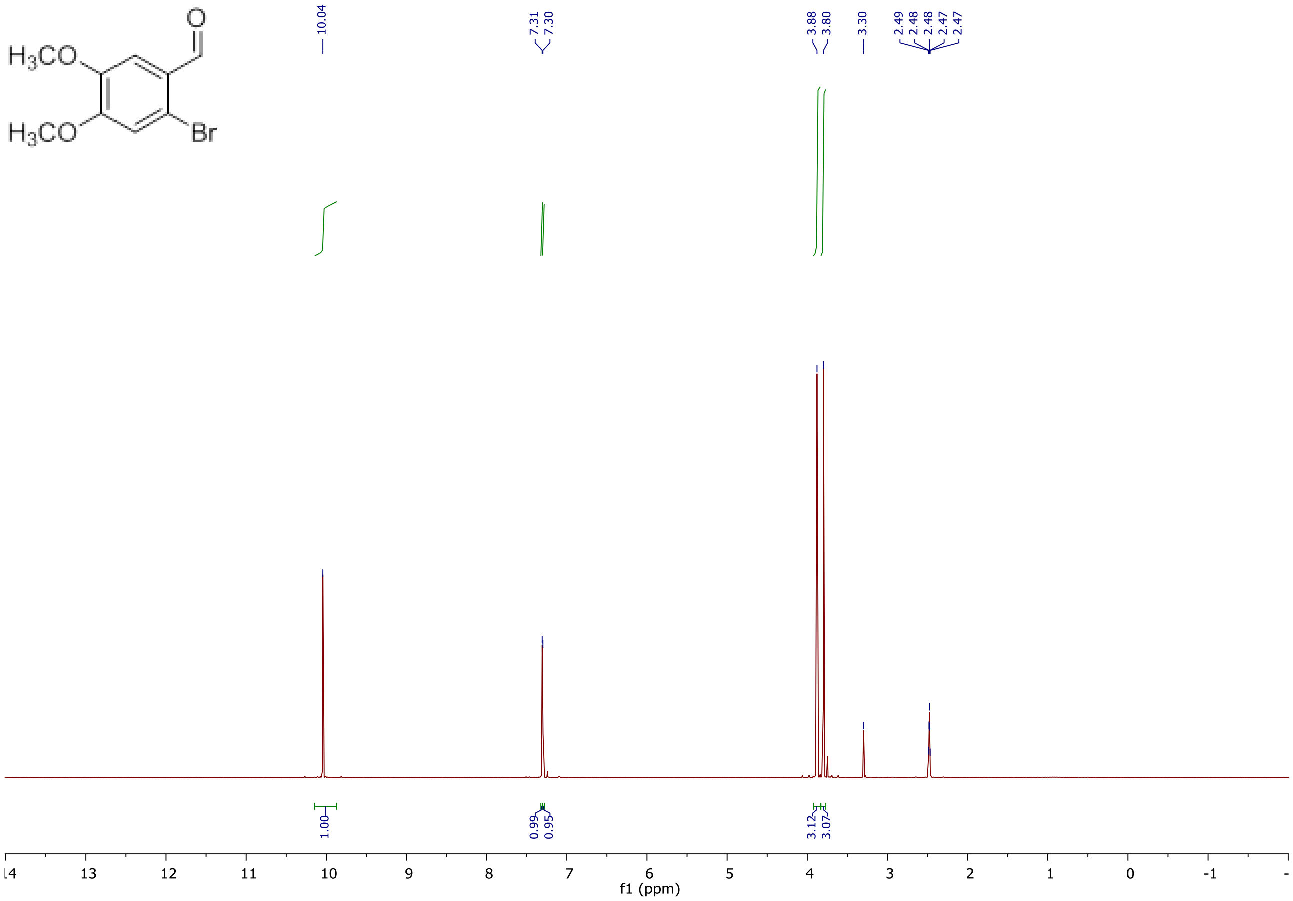

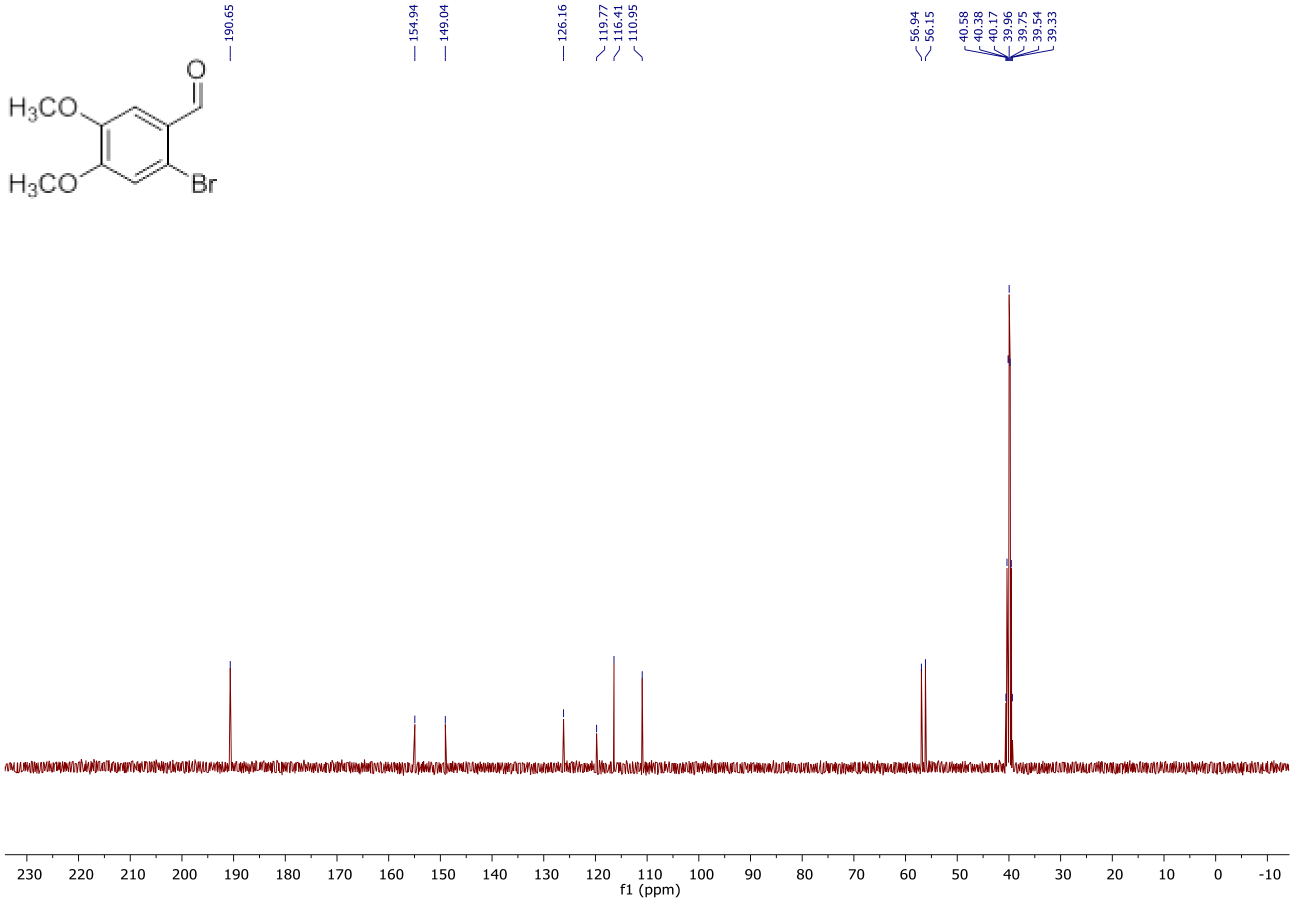

Ethyl 2'-formyl-4',5'-dimethoxy-[1,1'-biphenyl]-4-carboxylate (**72**)

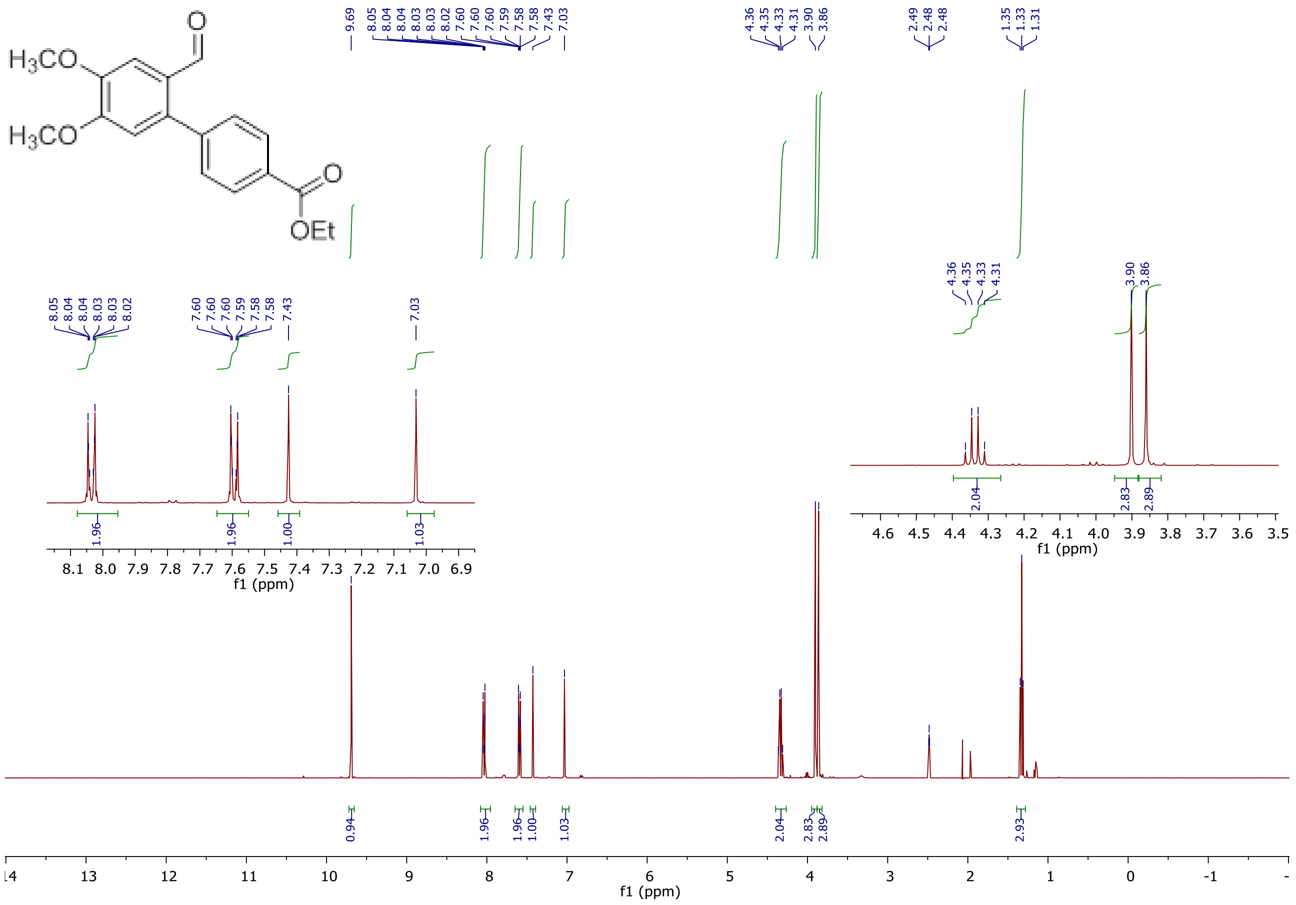

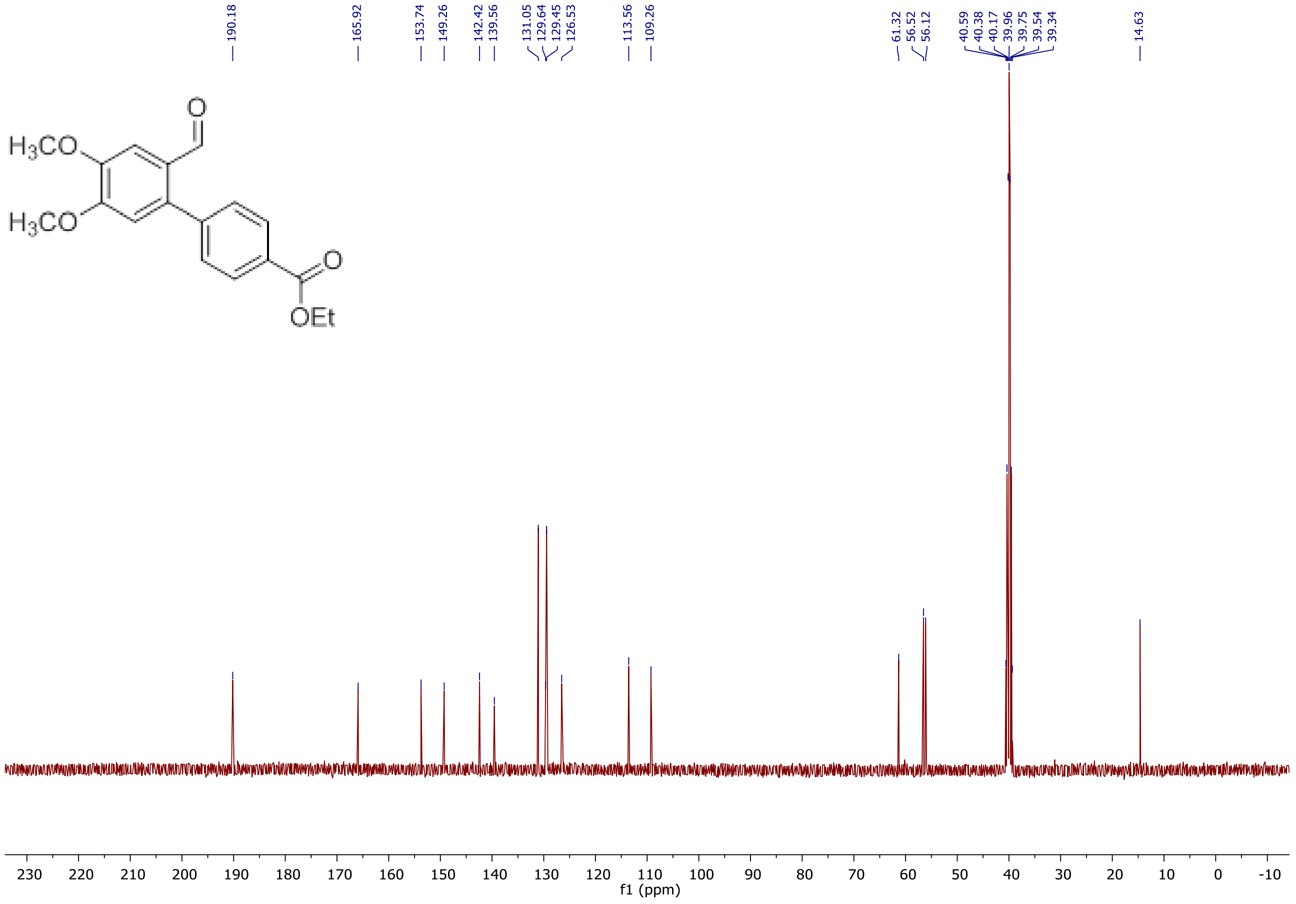

4'-(Ethoxycarbonyl)-4,5-dimethoxy-[1,1'-biphenyl]-2-carboxylic acid (**75**)

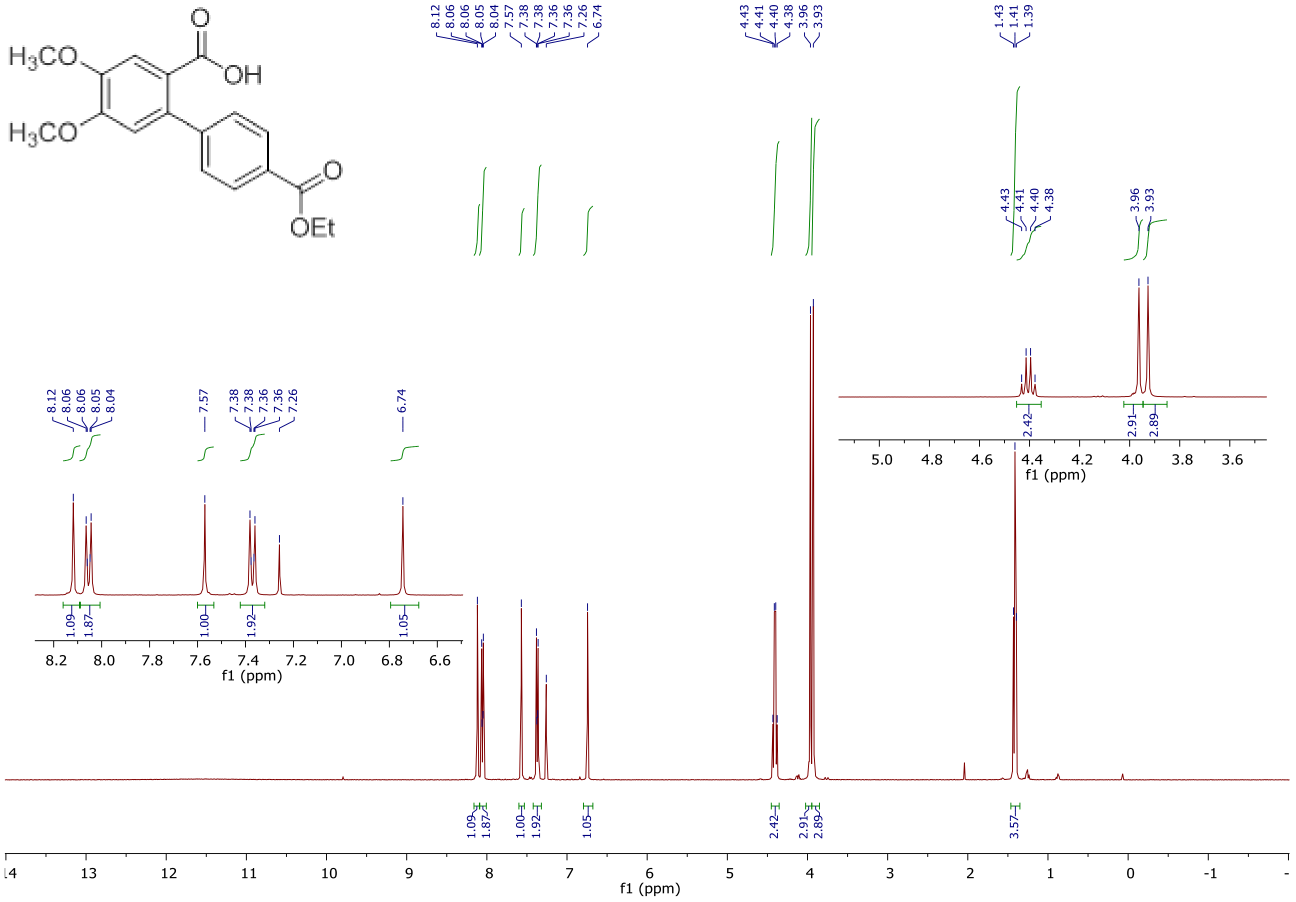

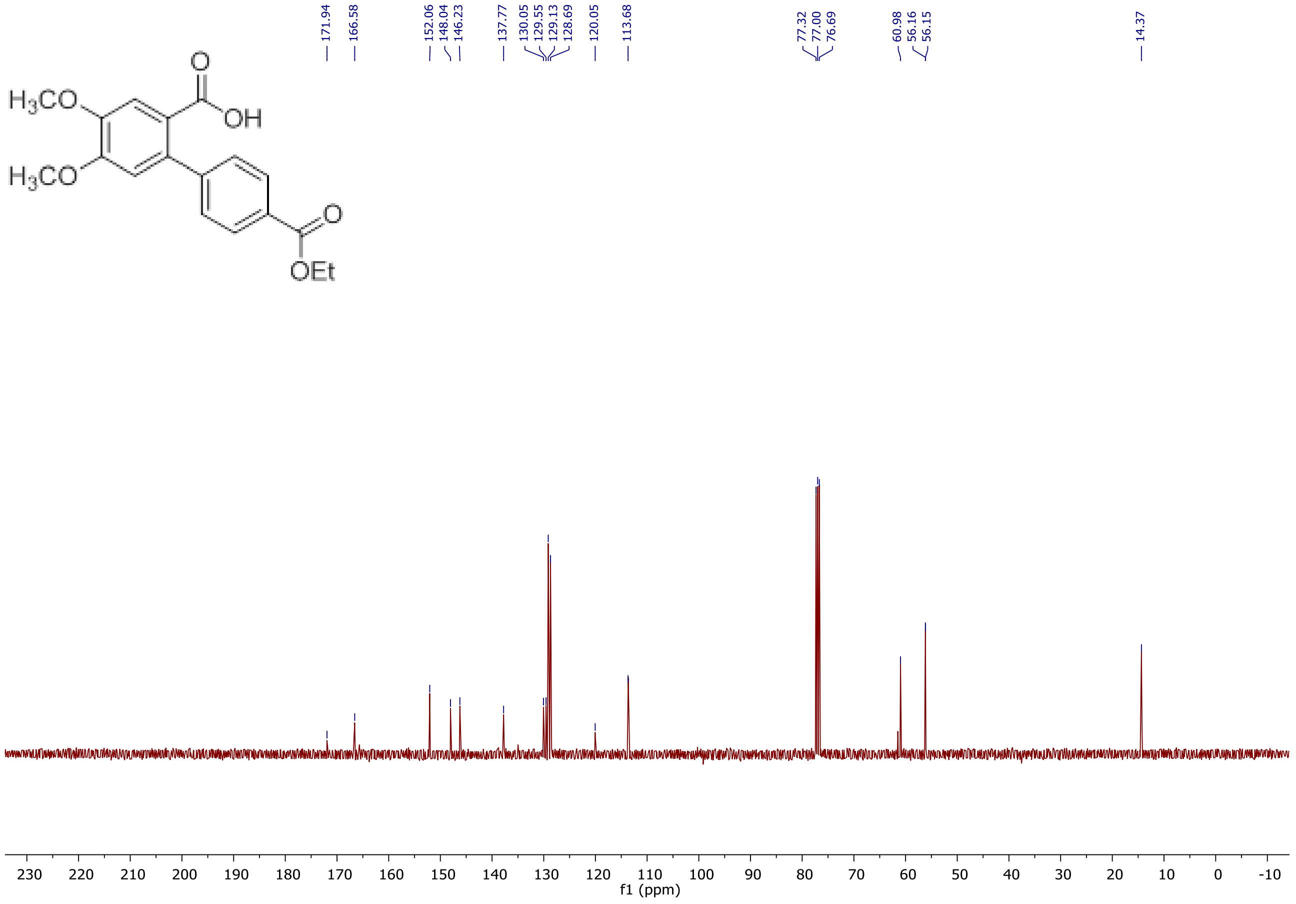

*Ethyl 8,9-dimethoxy-6-oxo-6H-benzo[c]chromene-3-carboxylate (**78**)*

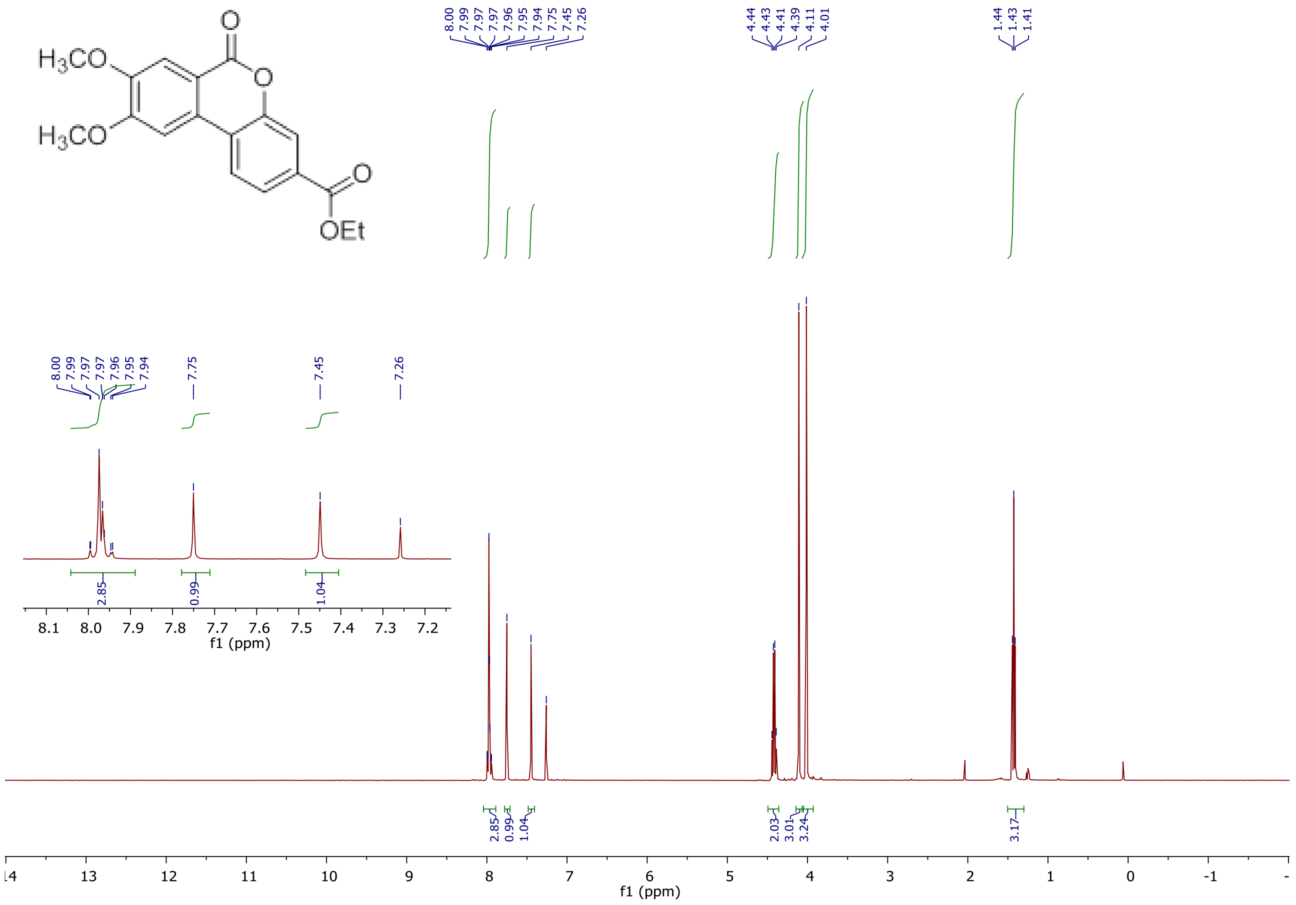

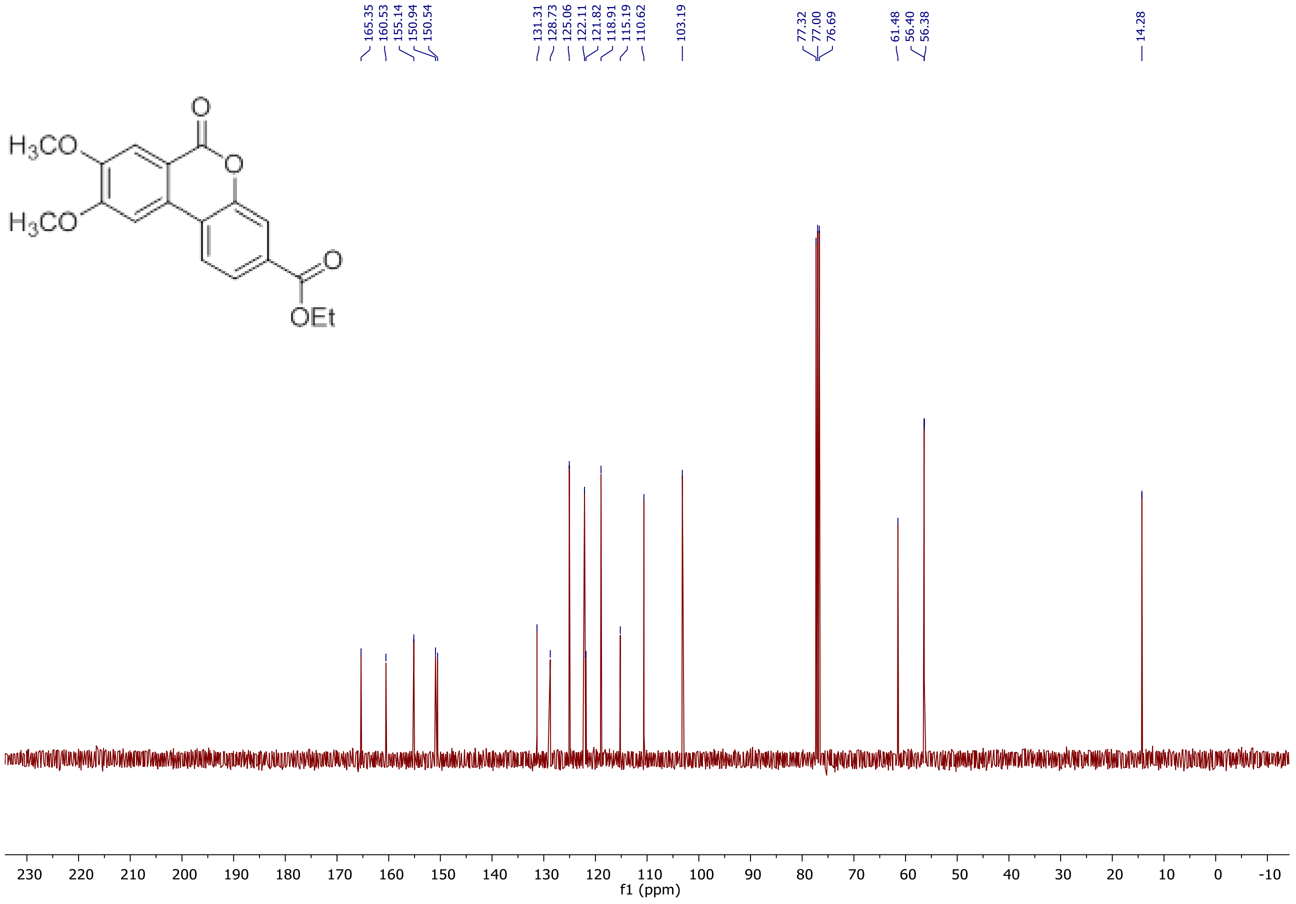

8,9-Dihydroxy-6-oxo-6H-benzo[c]chromene-3-carboxylic acid (**81**)

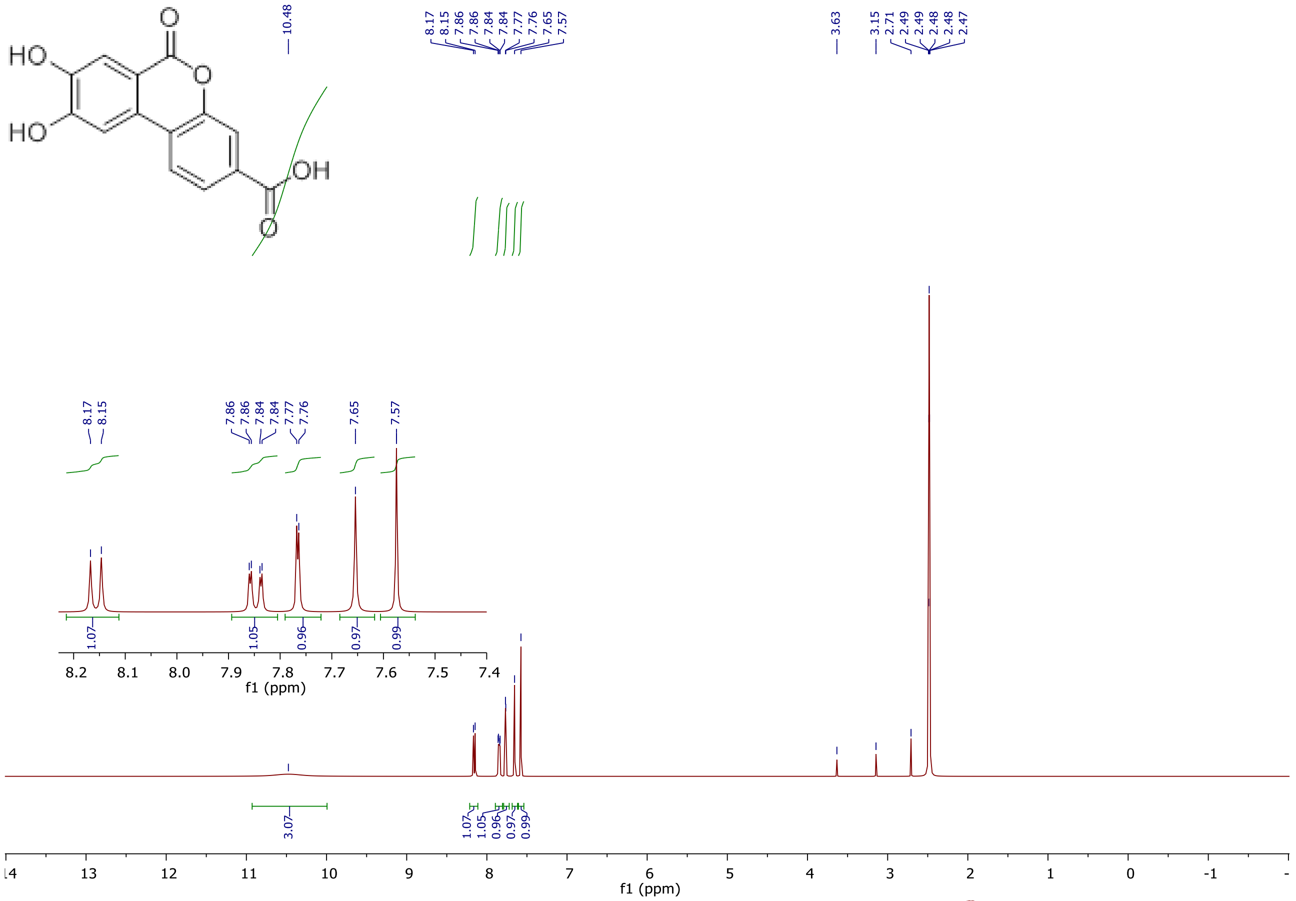

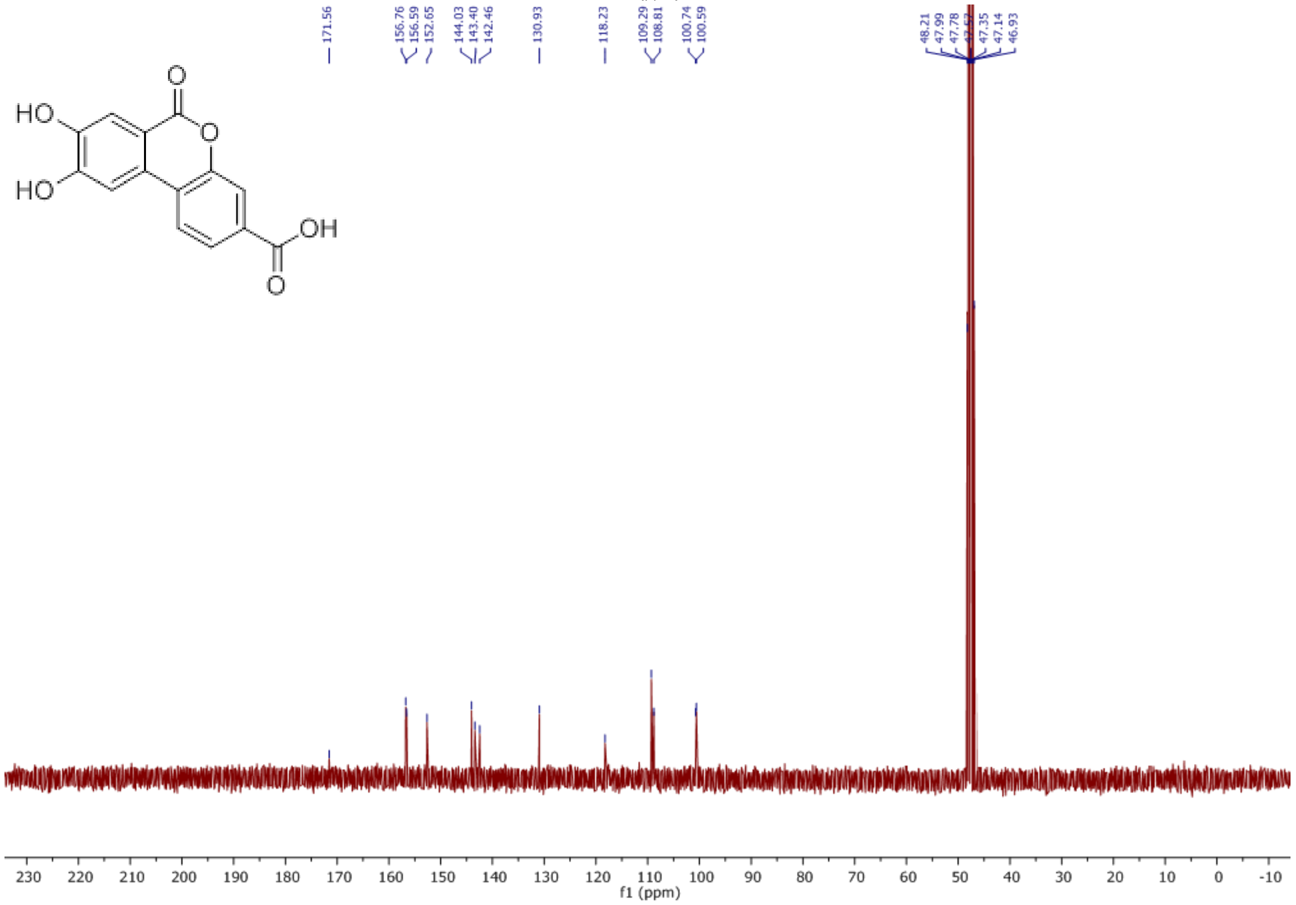

2'-Formyl-4',5'-dimethoxy-[1,1'-biphenyl]-4-carboxamide (**73**)

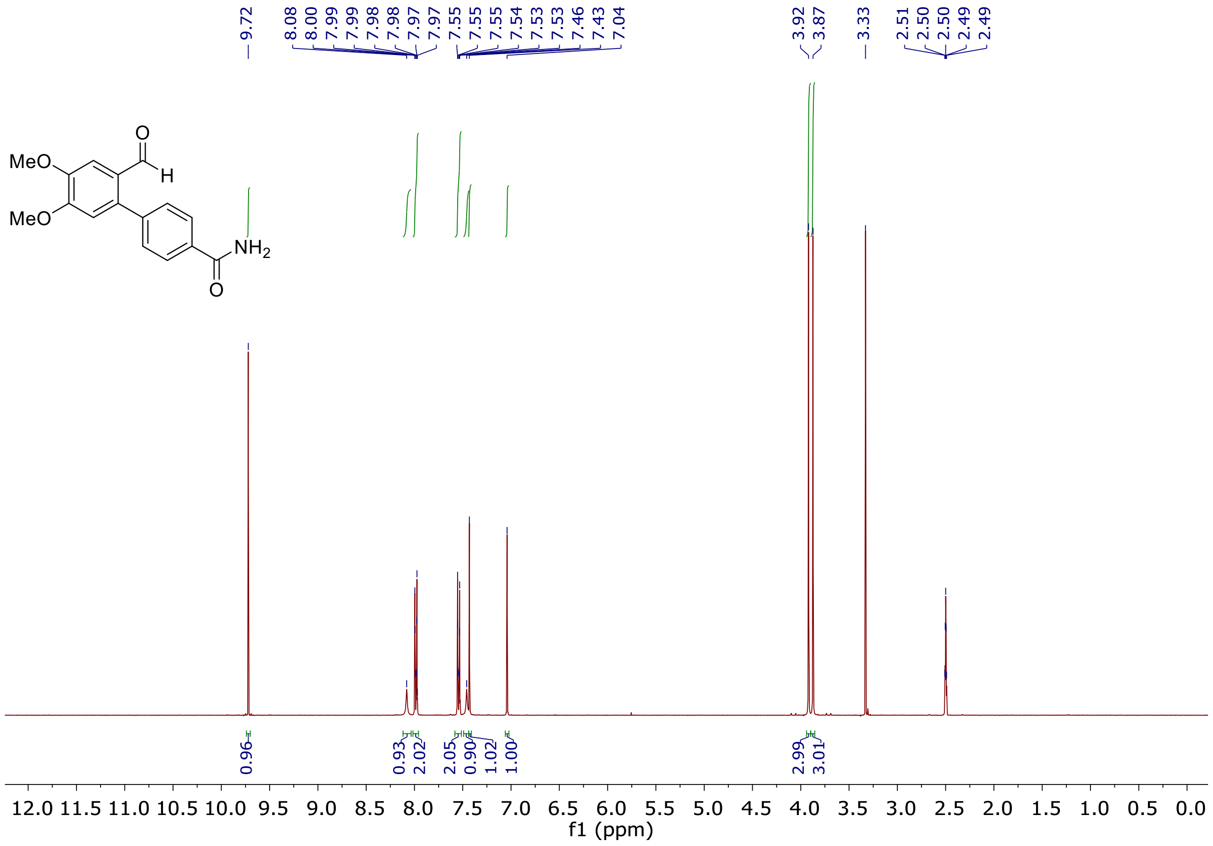

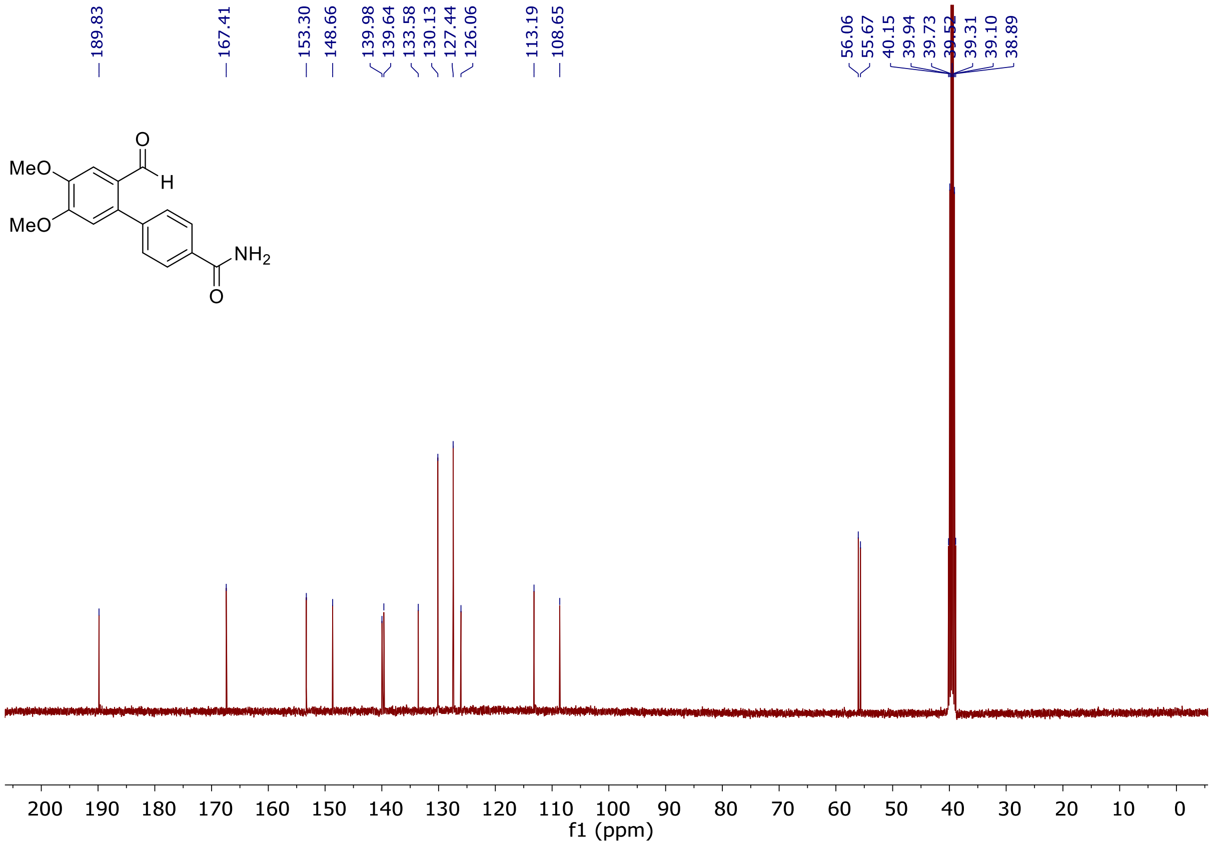

4'-Carbamoyl-4,5-dimethoxy-[1,1'-biphenyl]-2-carboxylic acid (**76**)

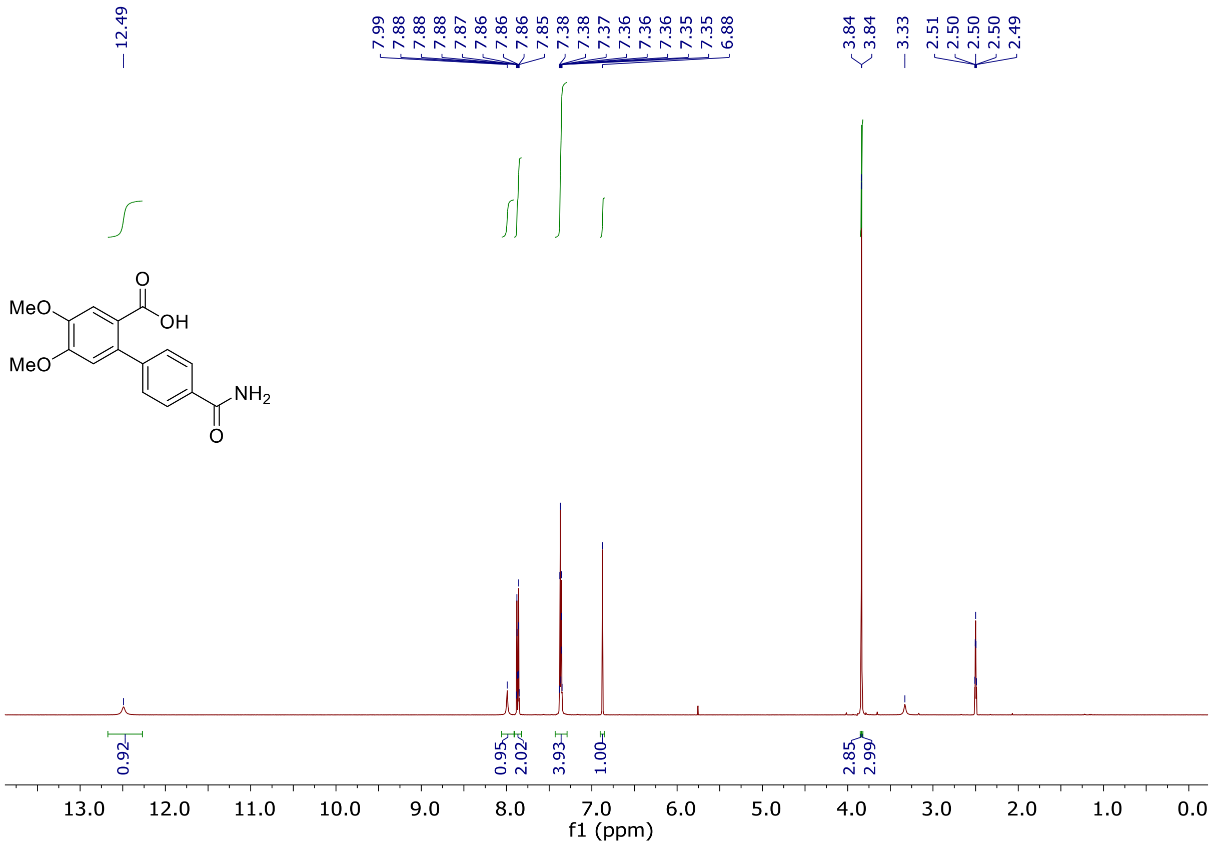

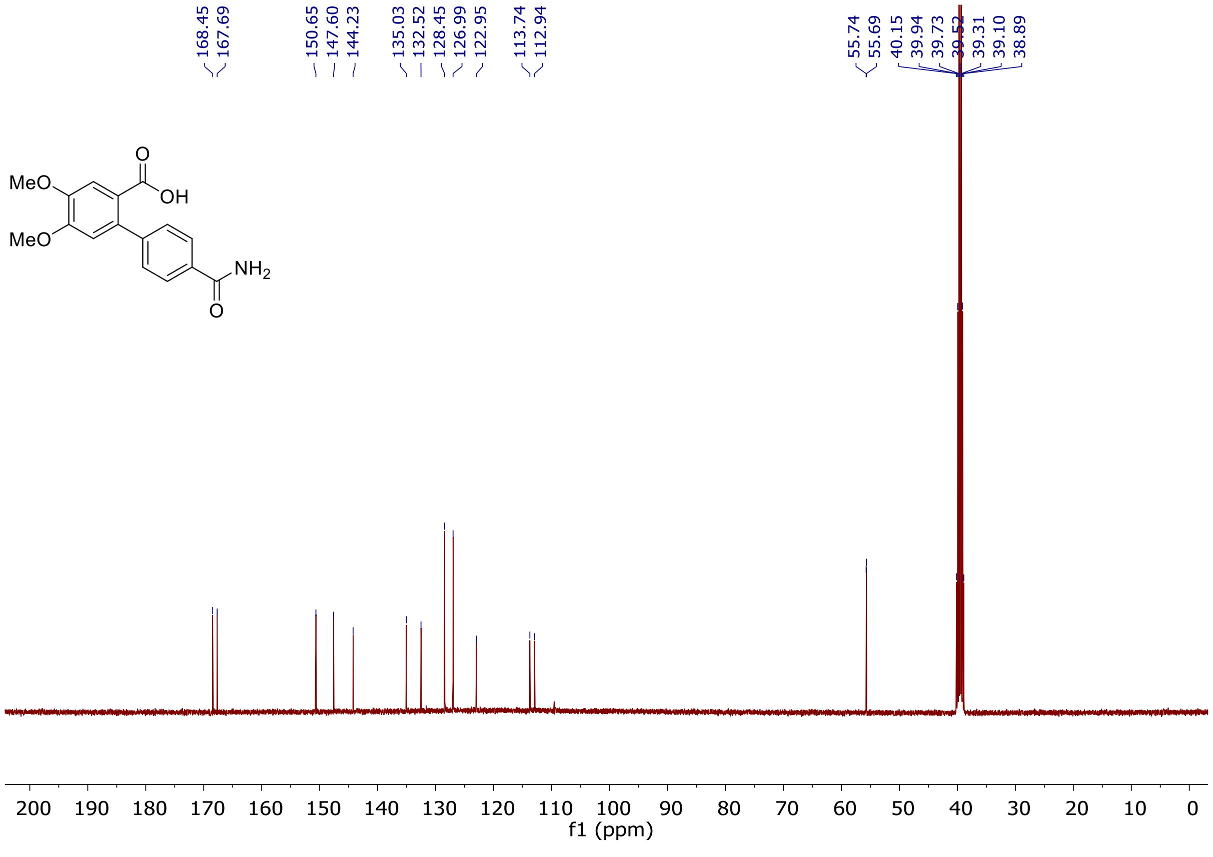

8,9-Dimethoxy-6-oxo-6H-benzo[c]chromene-3-carboxamide (**79**)

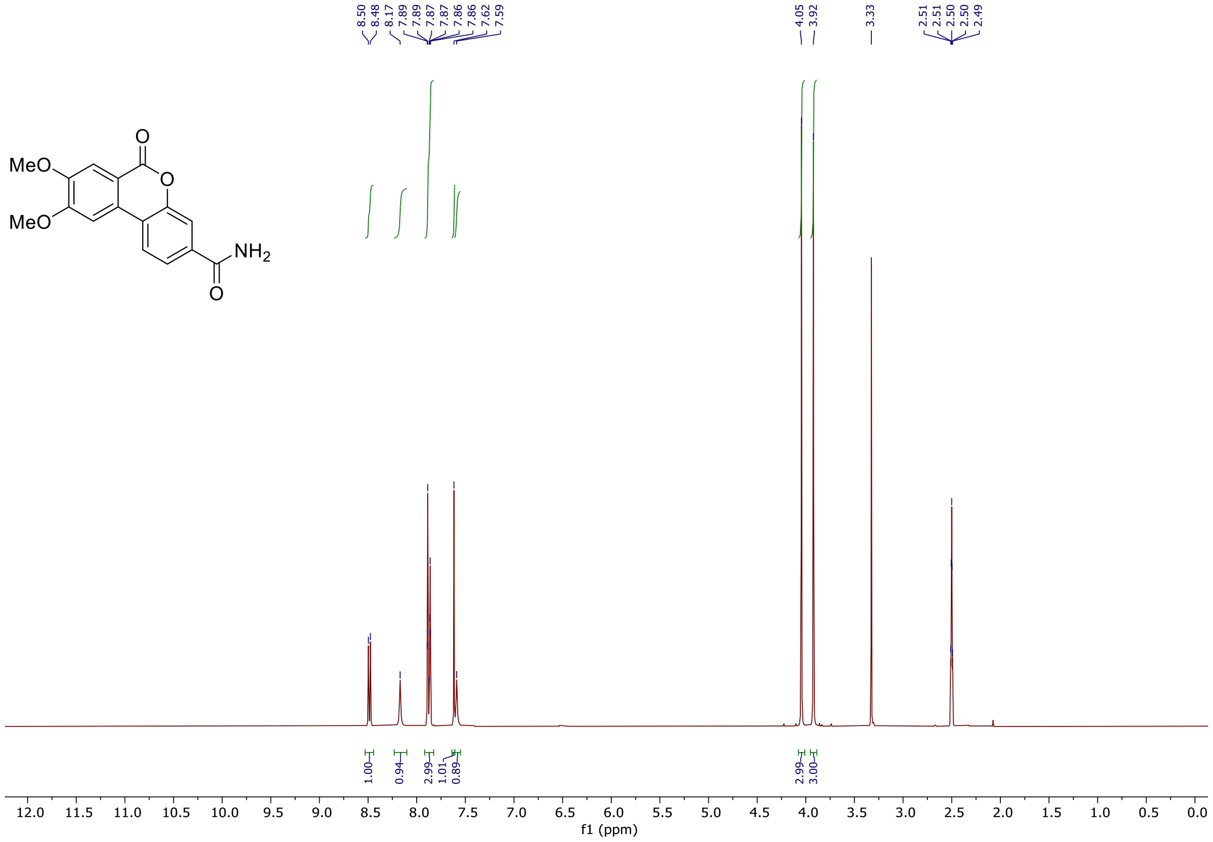

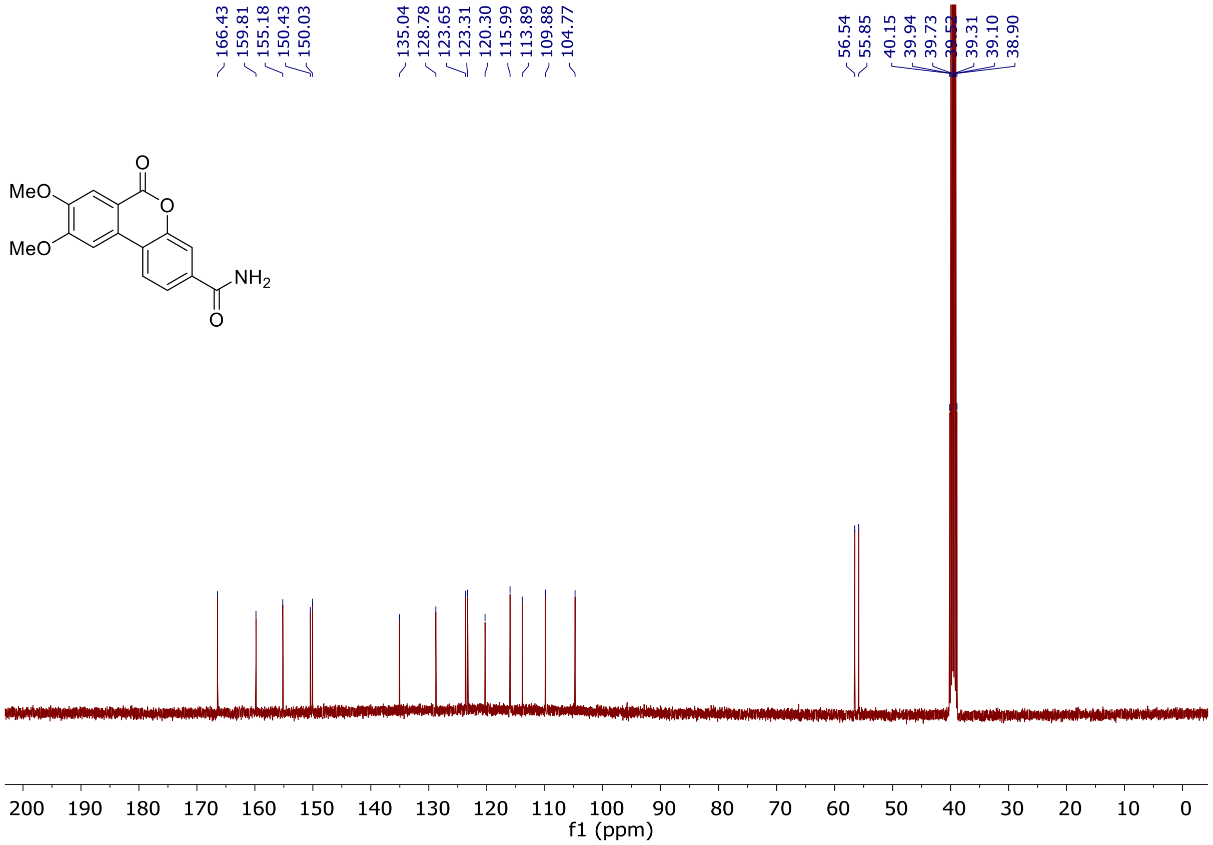

8,9-Dihydroxy-6-oxo-6H-benzo[c]chromene-3-carboxamide (**82**)

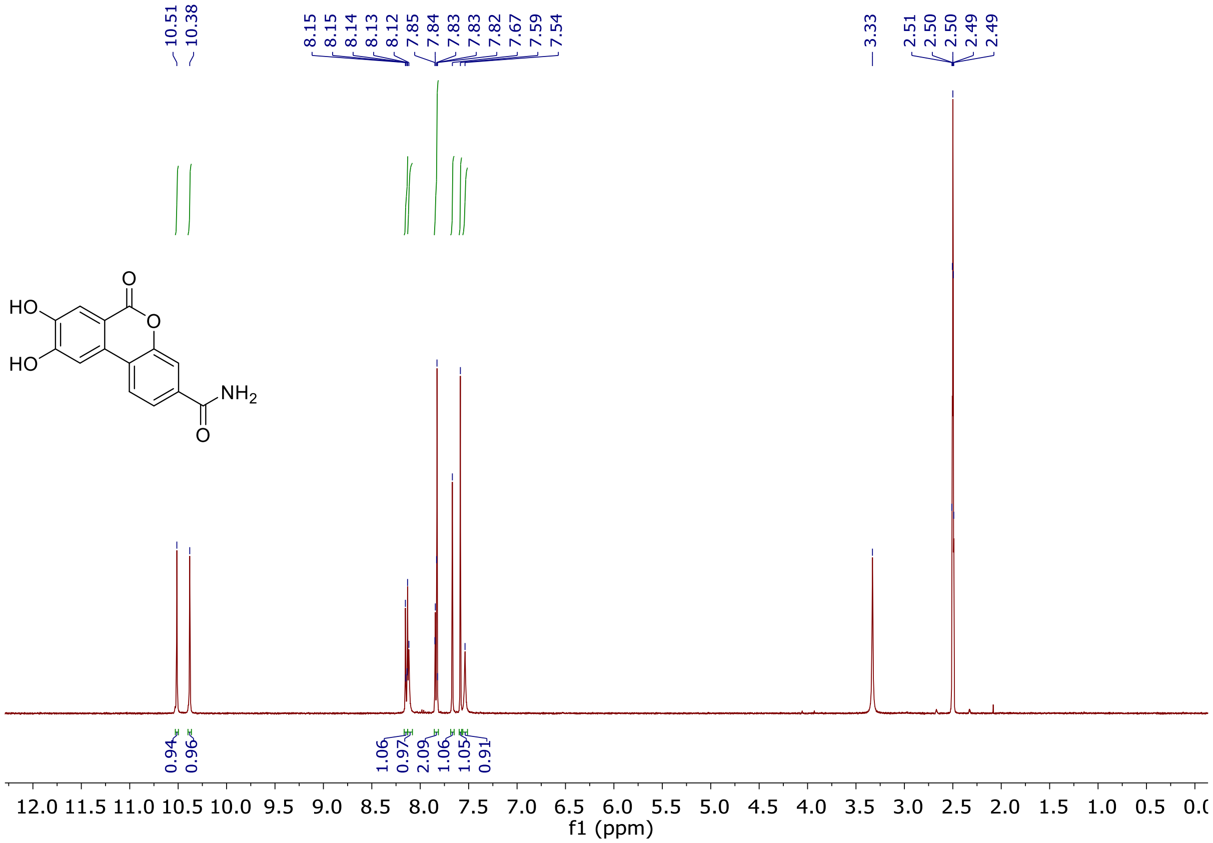

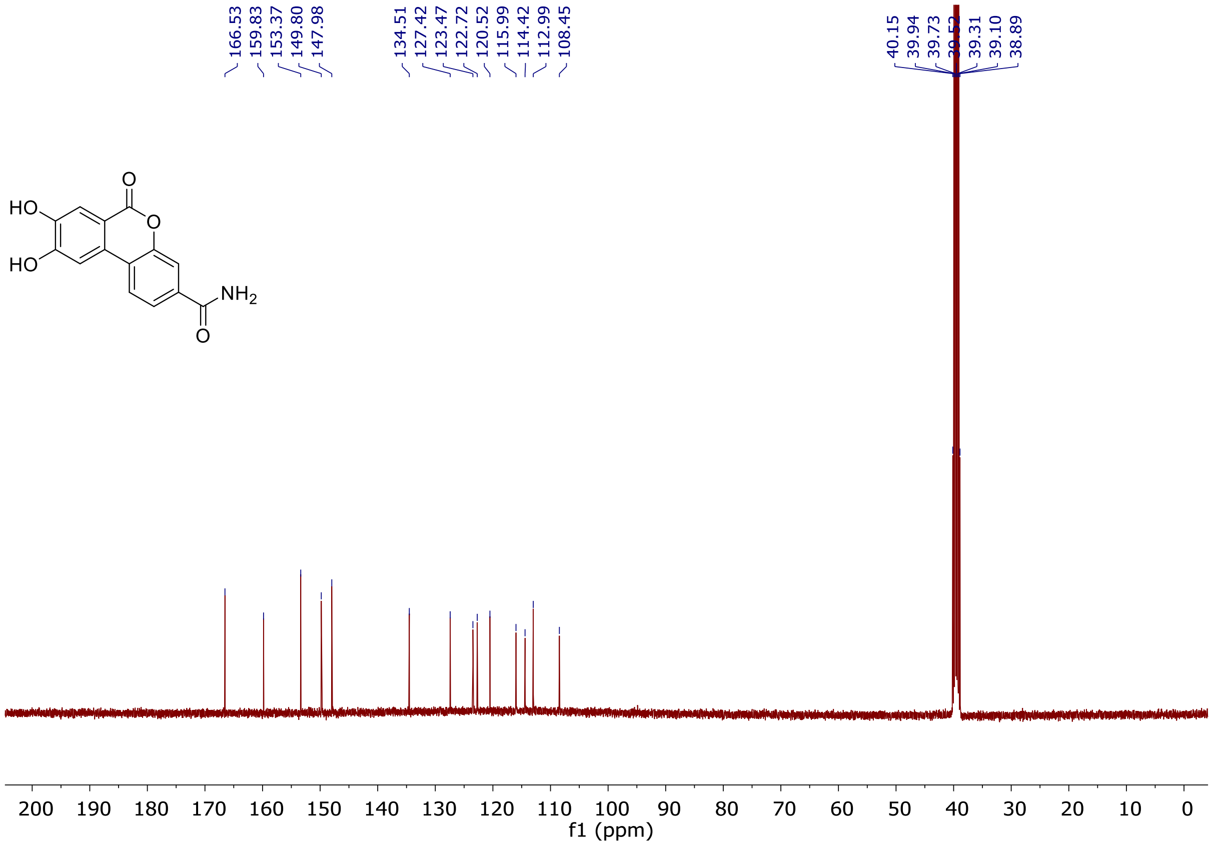

2'-Formyl-4',5'-dimethoxy-[1,1'-biphenyl]-4-carbonitrile (**74**)

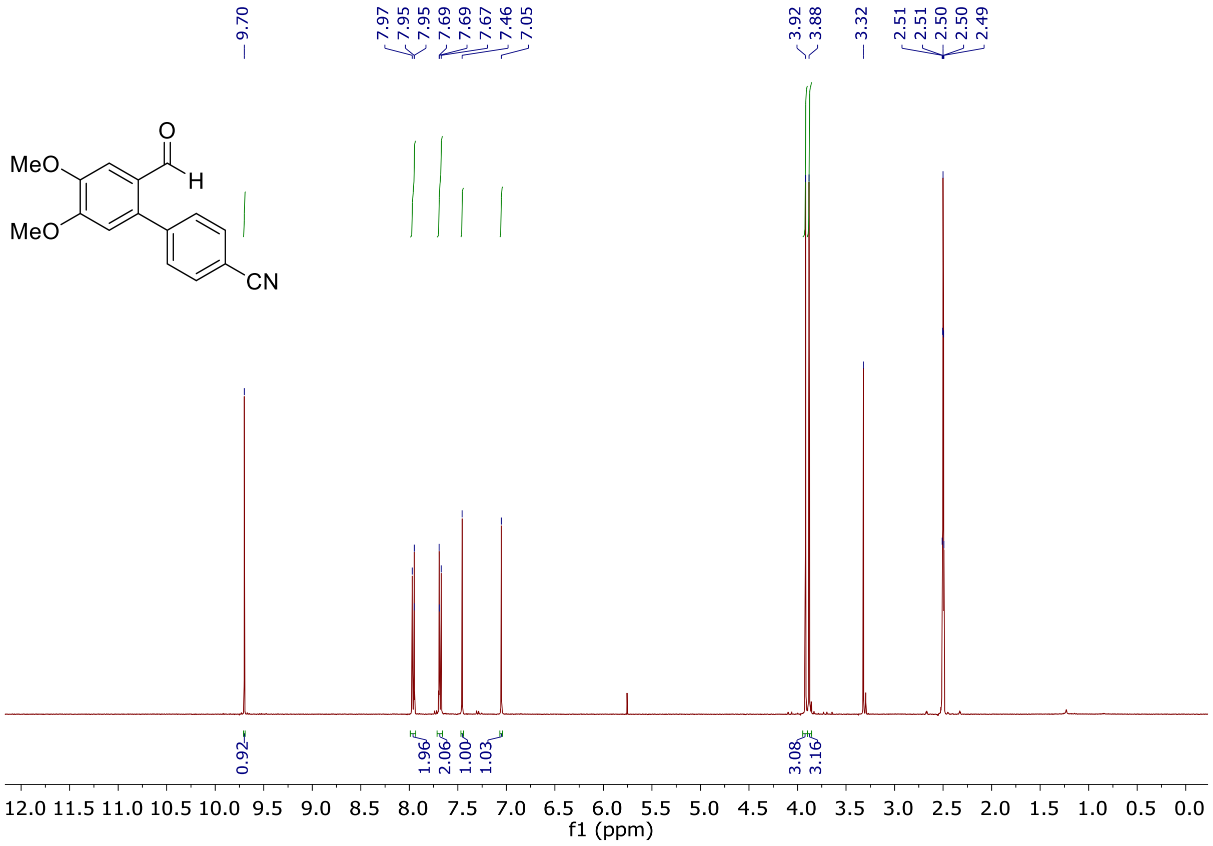

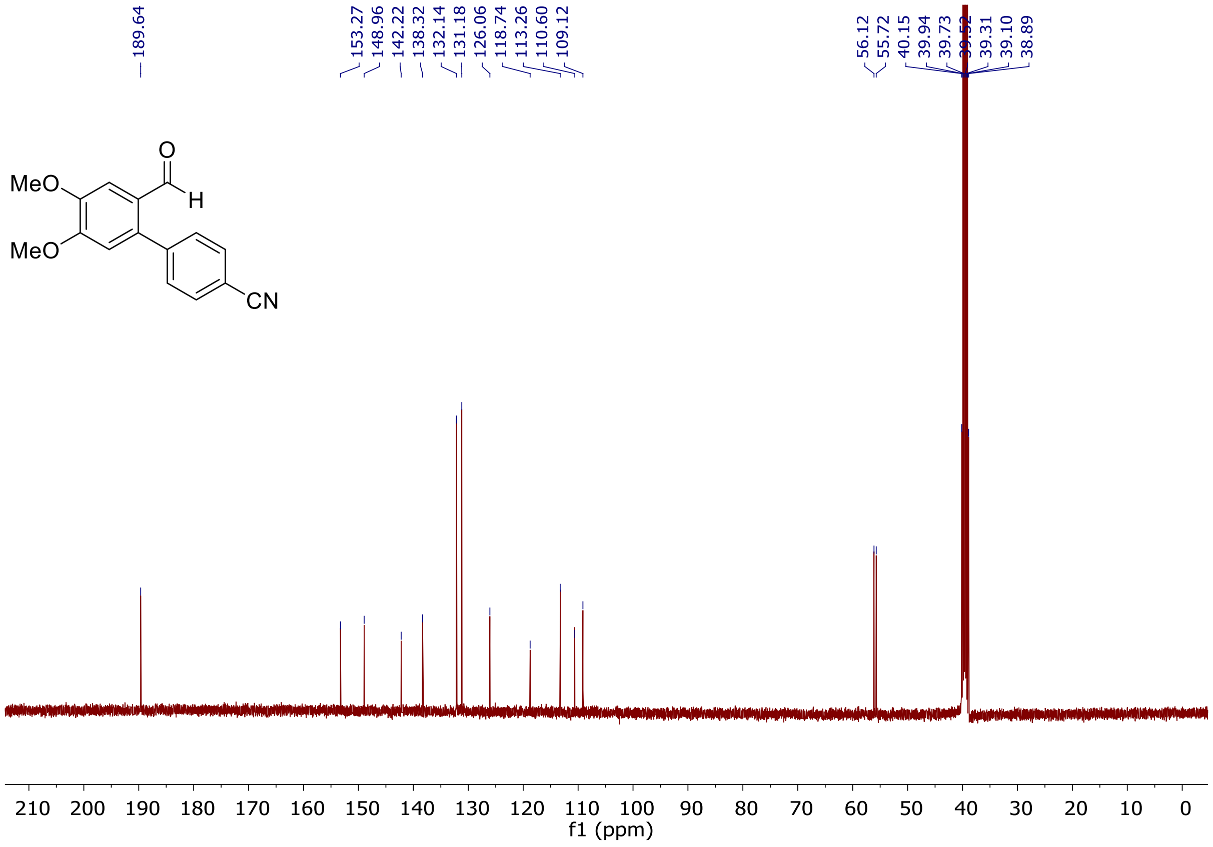

4'-Cyano-4,5-dimethoxy-[1,1'-biphenyl]-2-carboxylic acid (**77**)

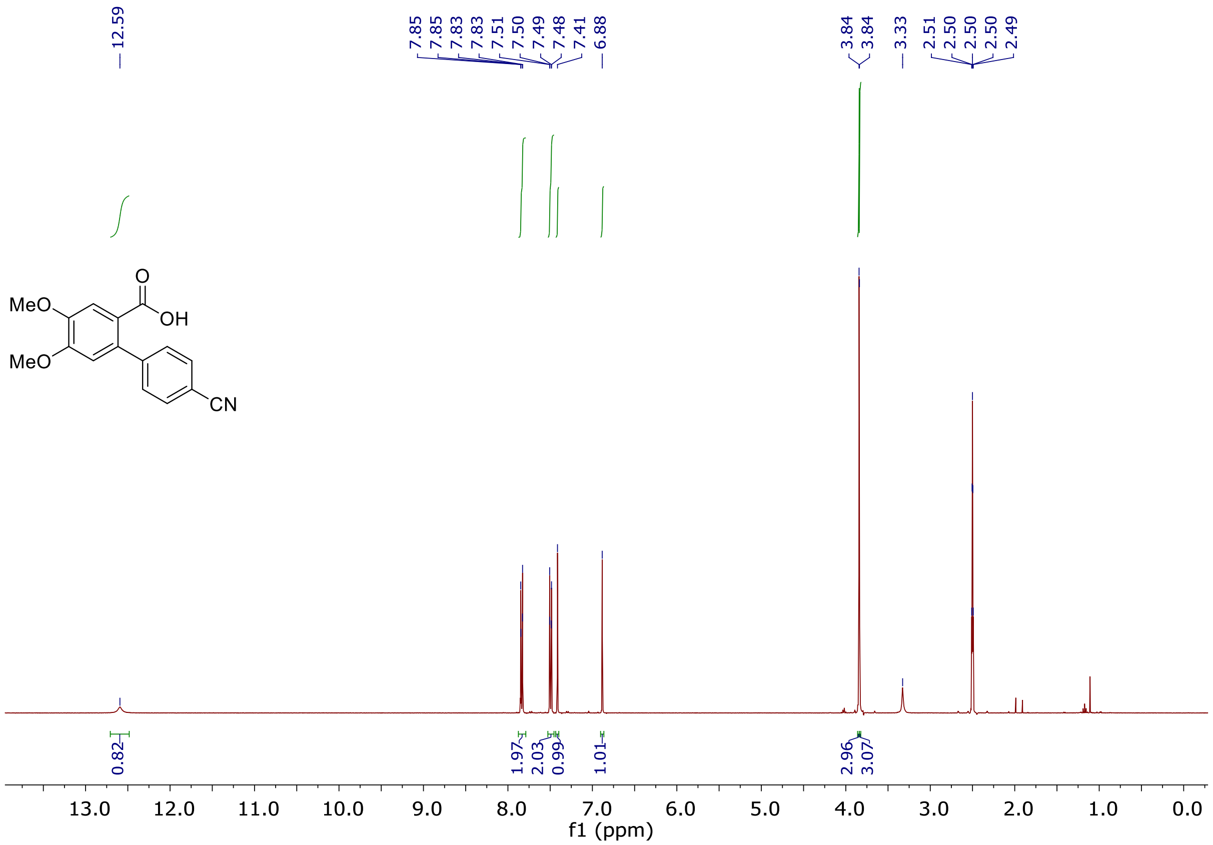

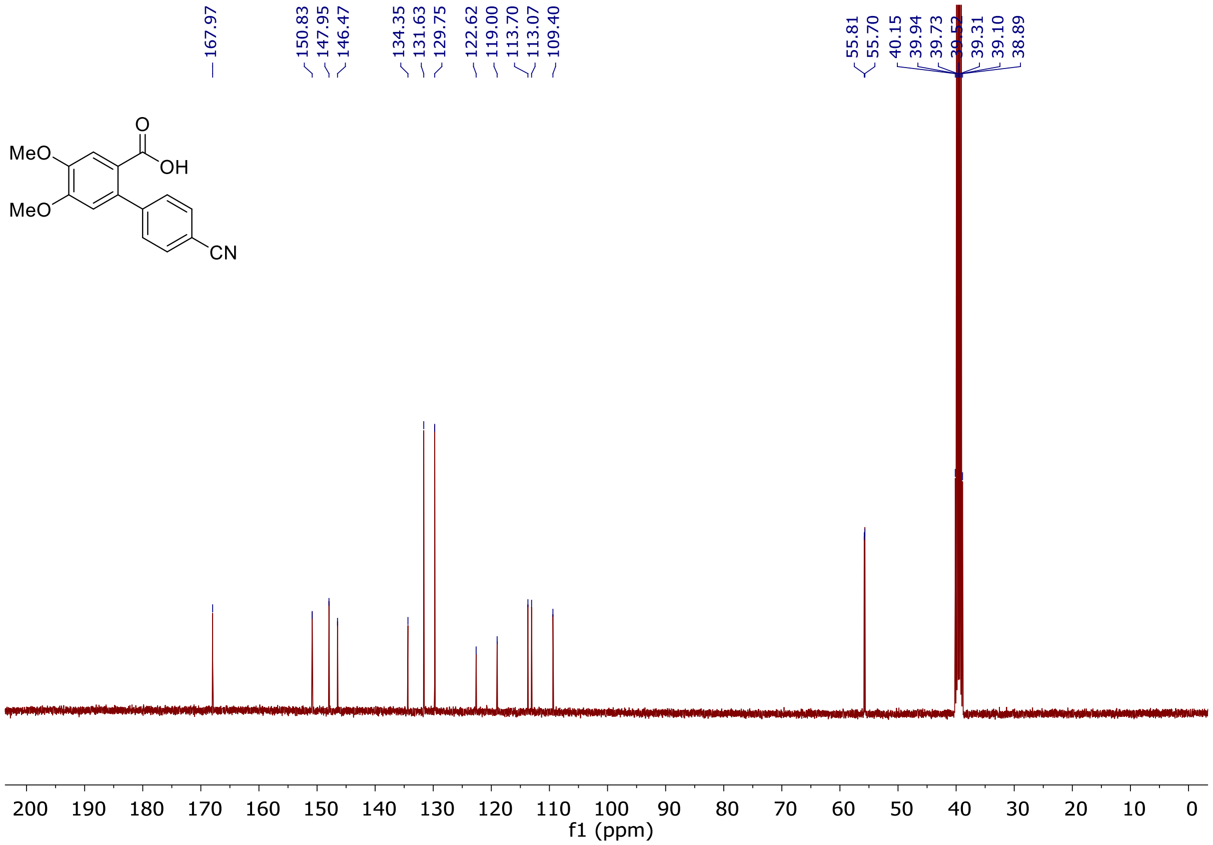

8,9-Dimethoxy-6-oxo-6H-benzo[c]chromene-3-carbonitrile (**80**)

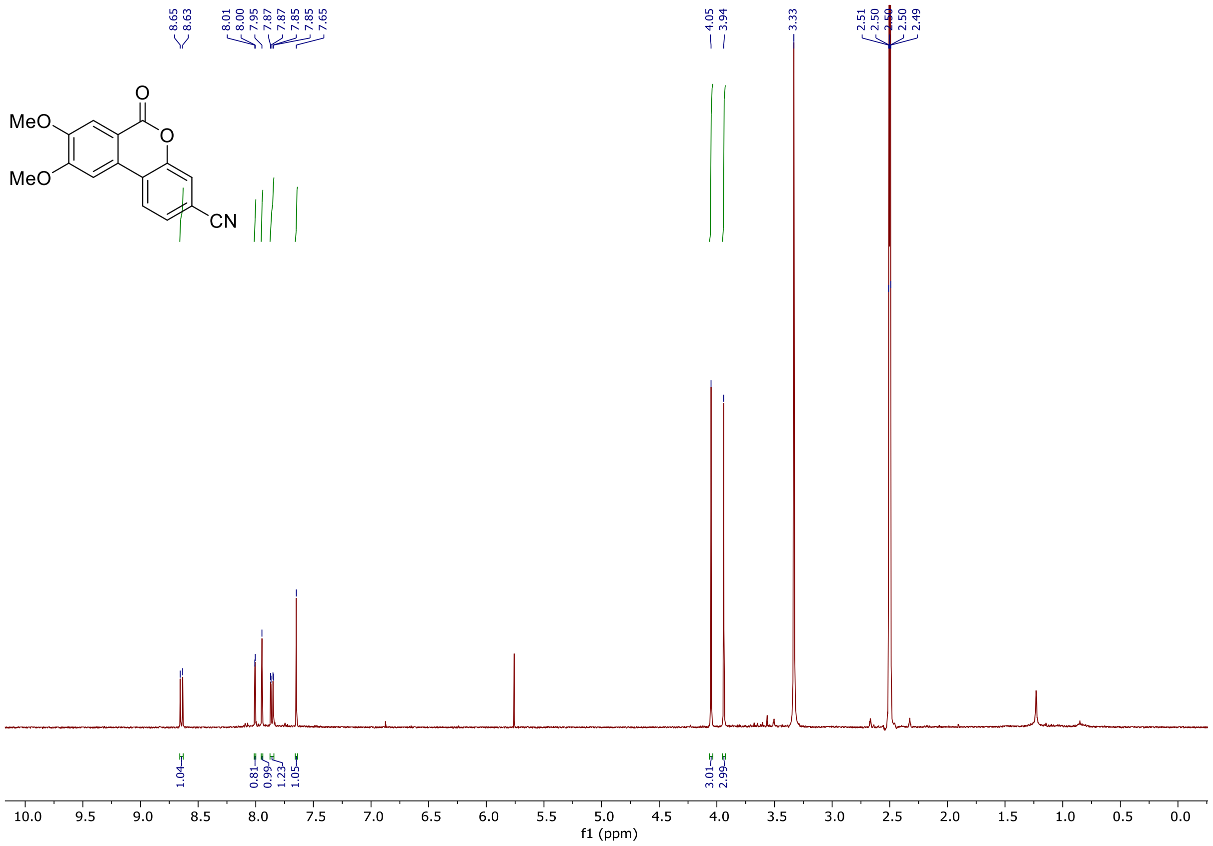

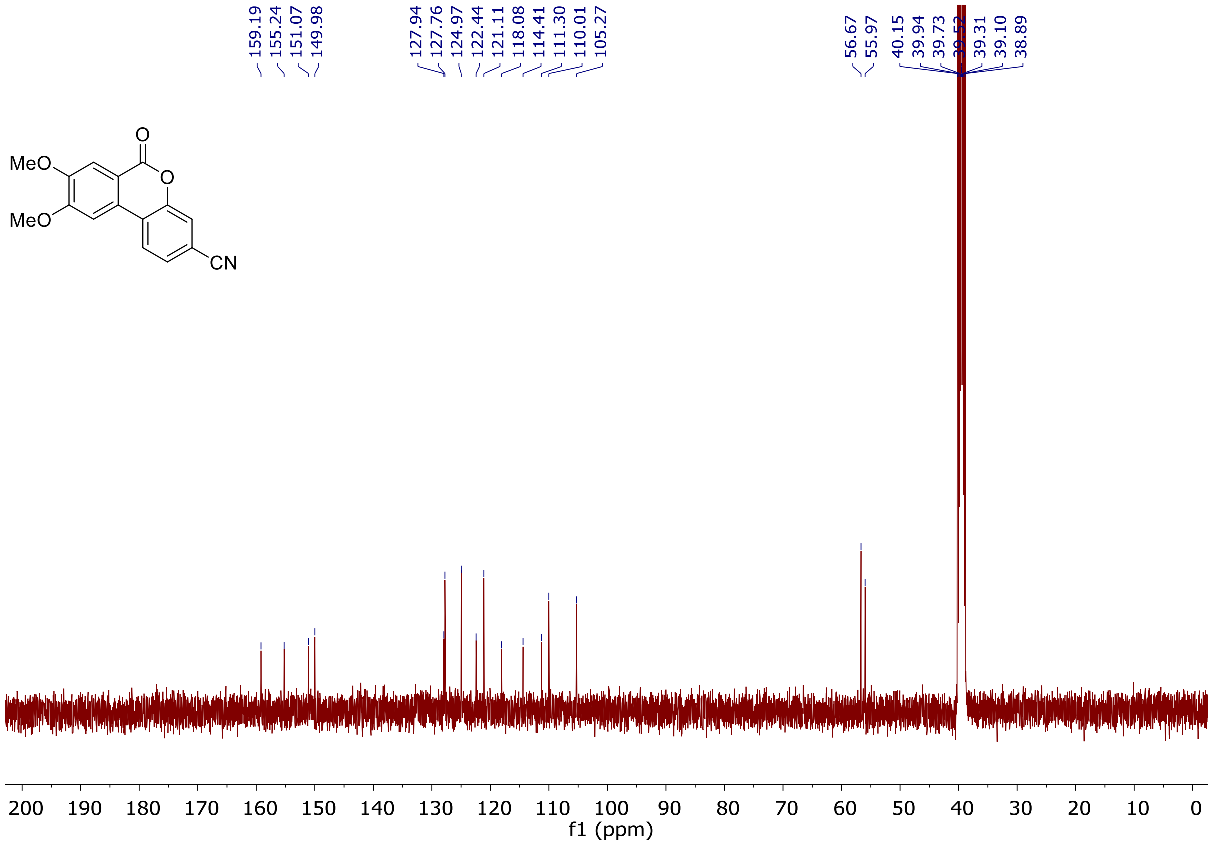

8,9-Dihydroxy-6-oxo-6H-benzo[c]chromene-3-carbonitrile (**83**)

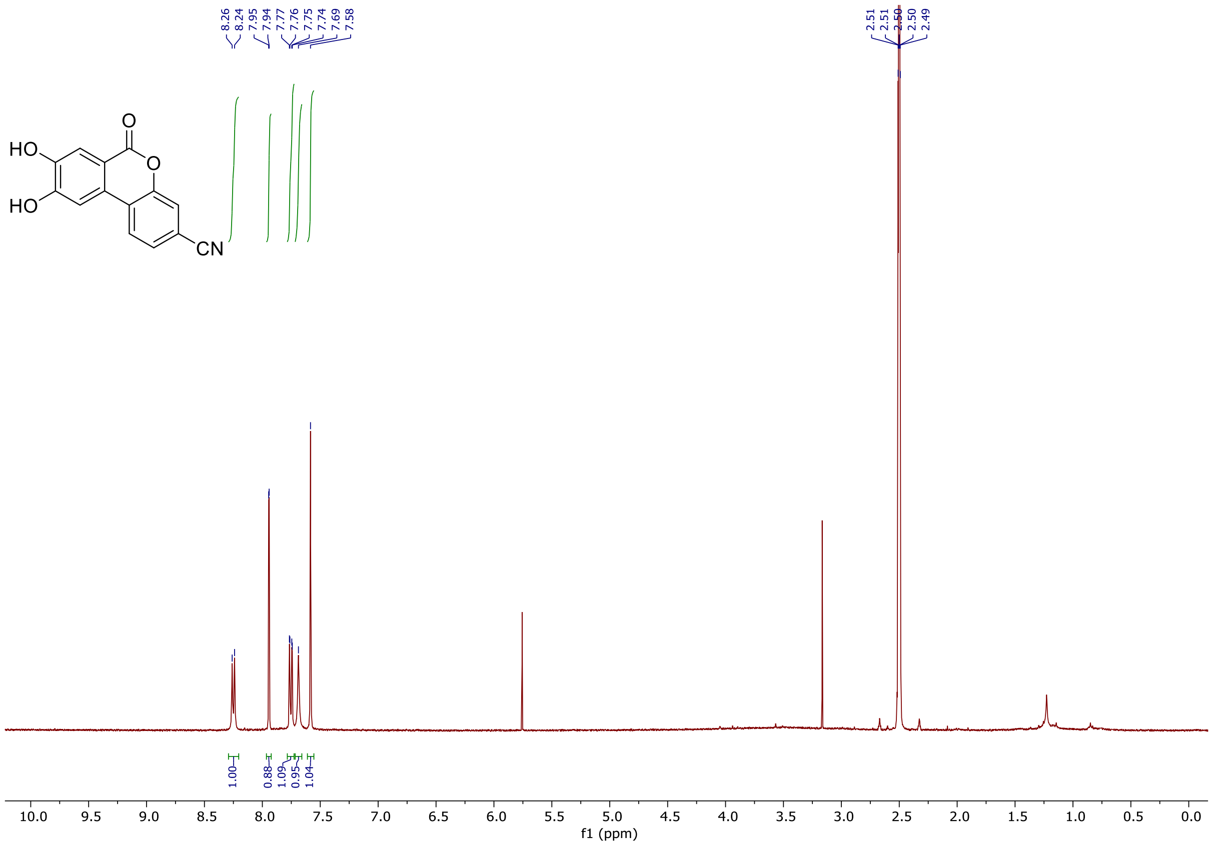

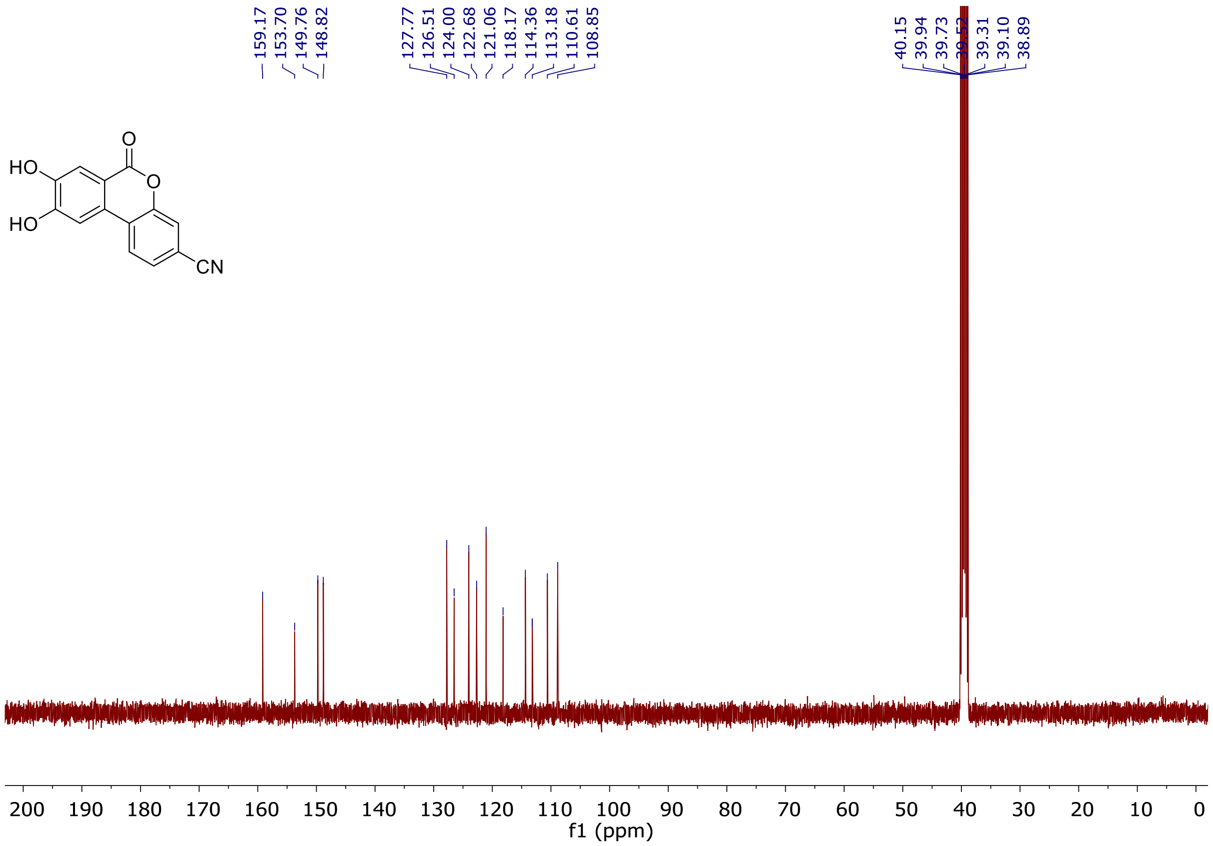

Methyl 4-(benzyloxy)-2-bromo-5-hydroxybenzoate (**84**)

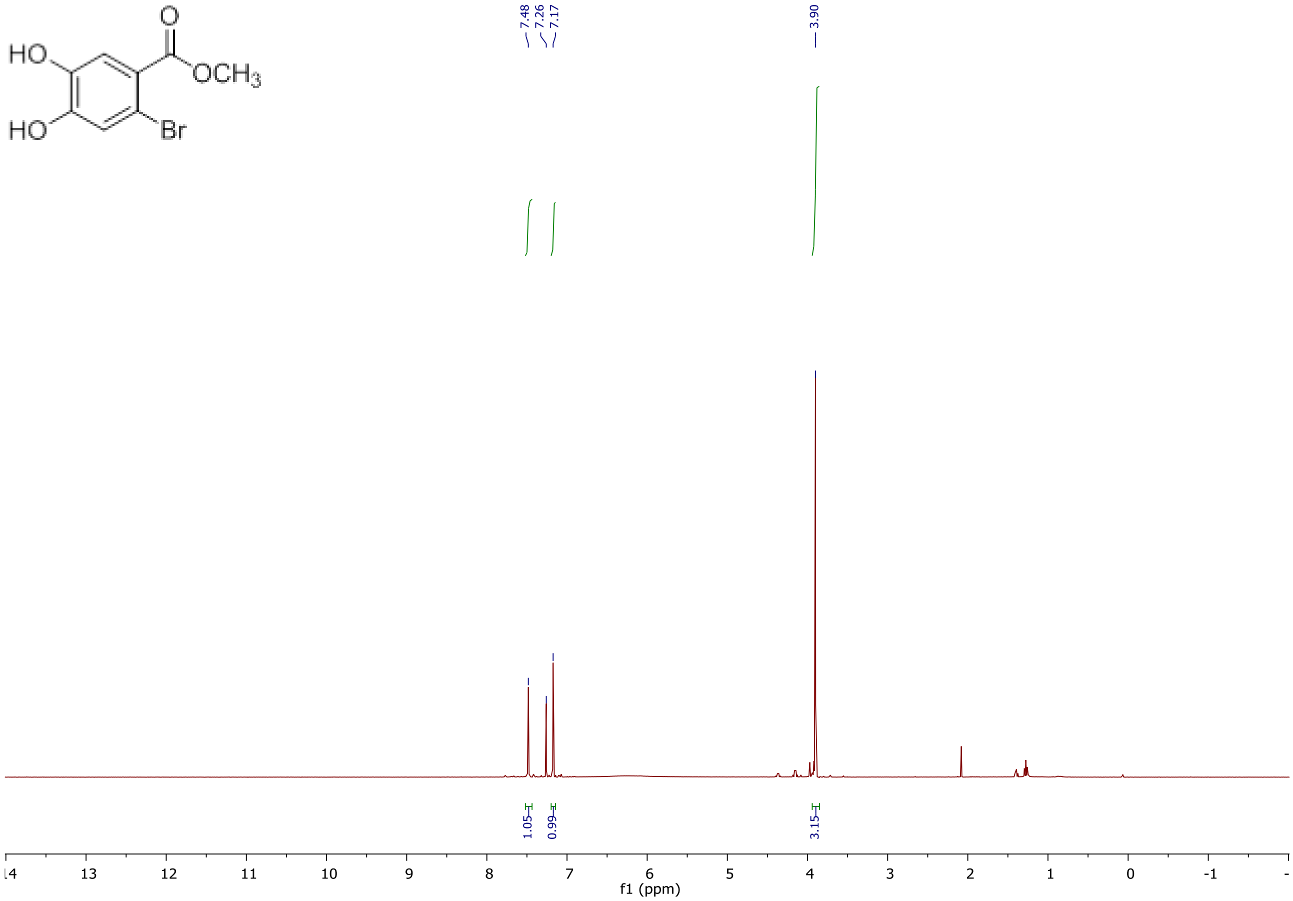

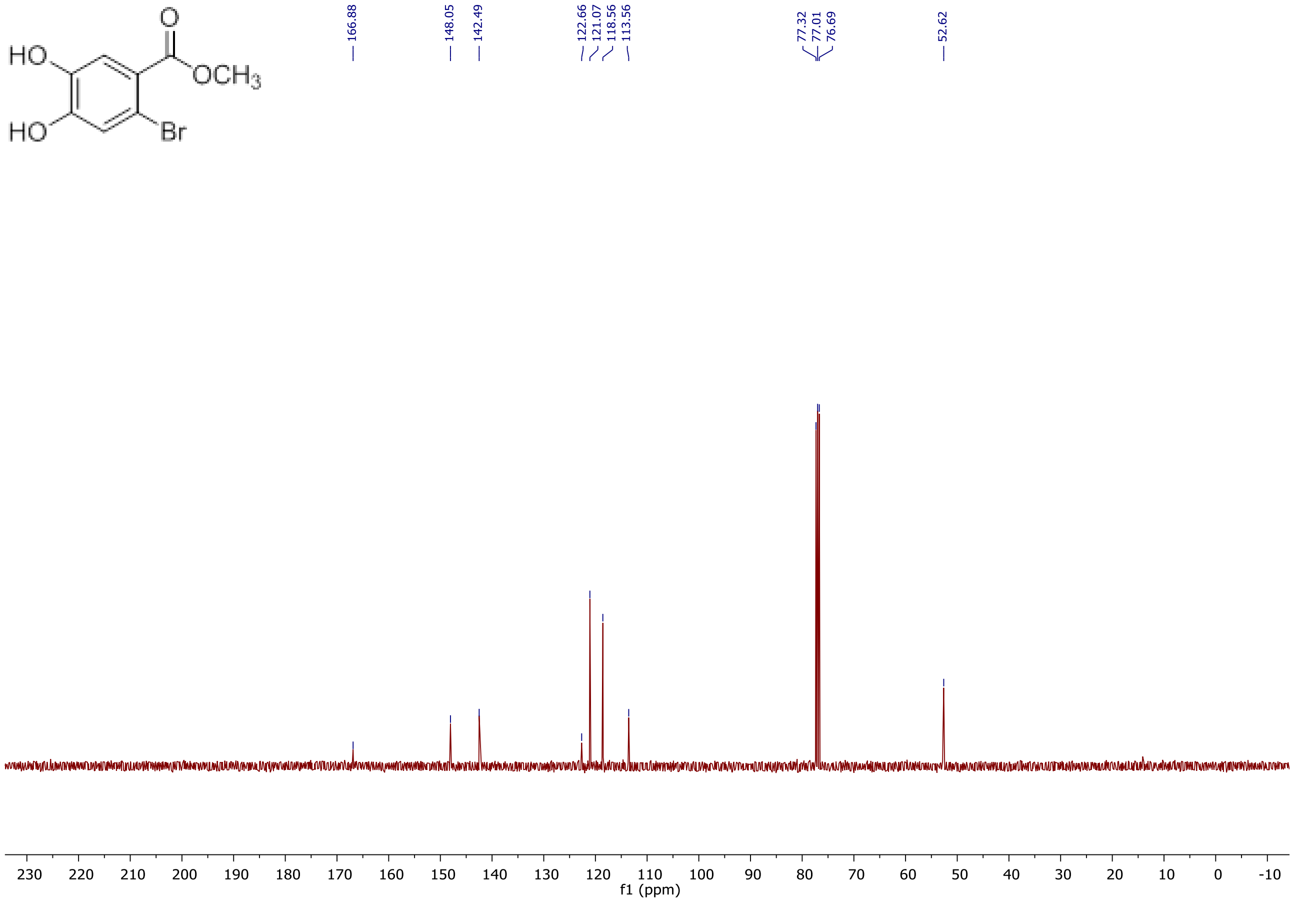

Methyl 4,5-bis(benzyloxy)-2-bromobenzoate (**85**)

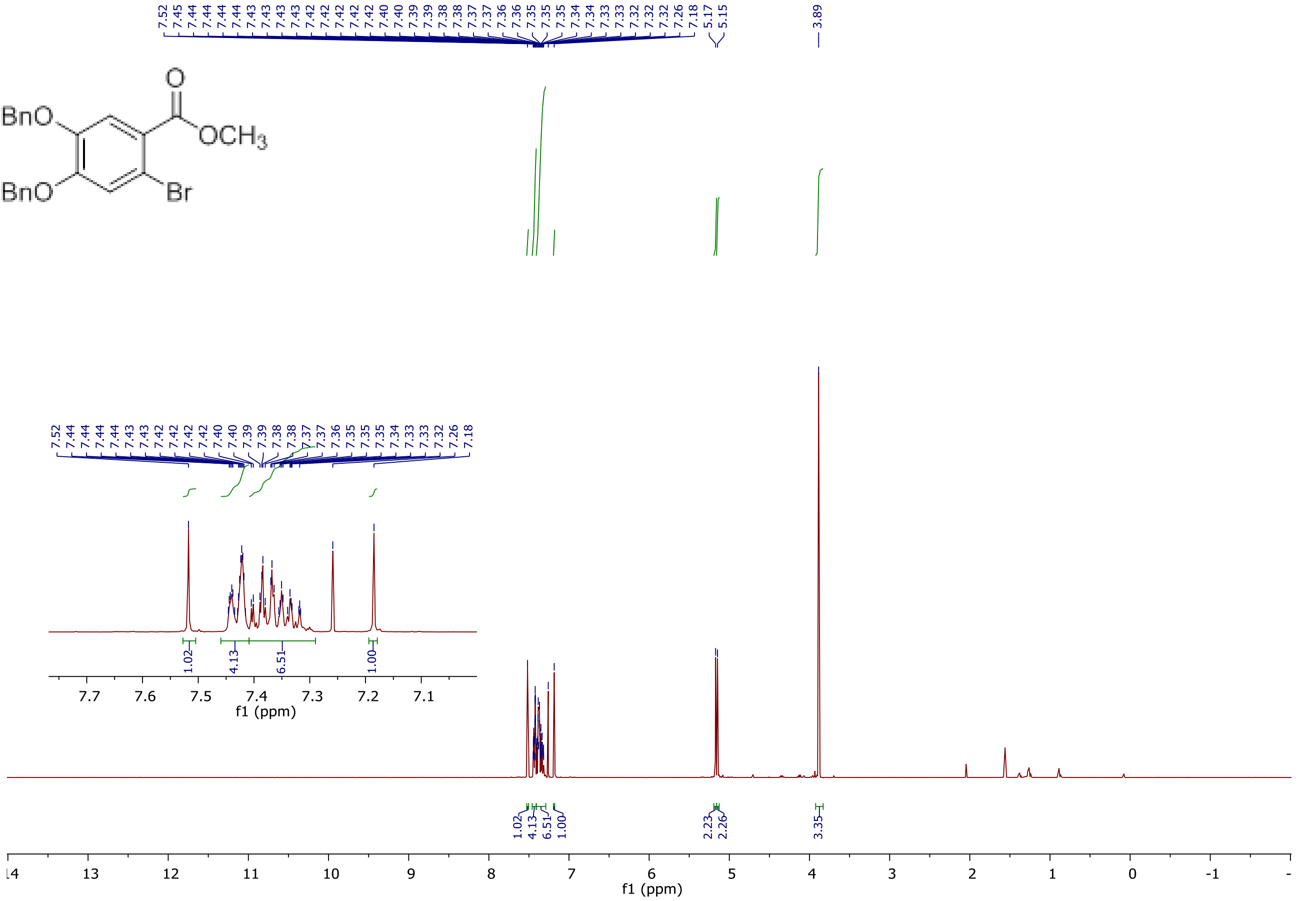

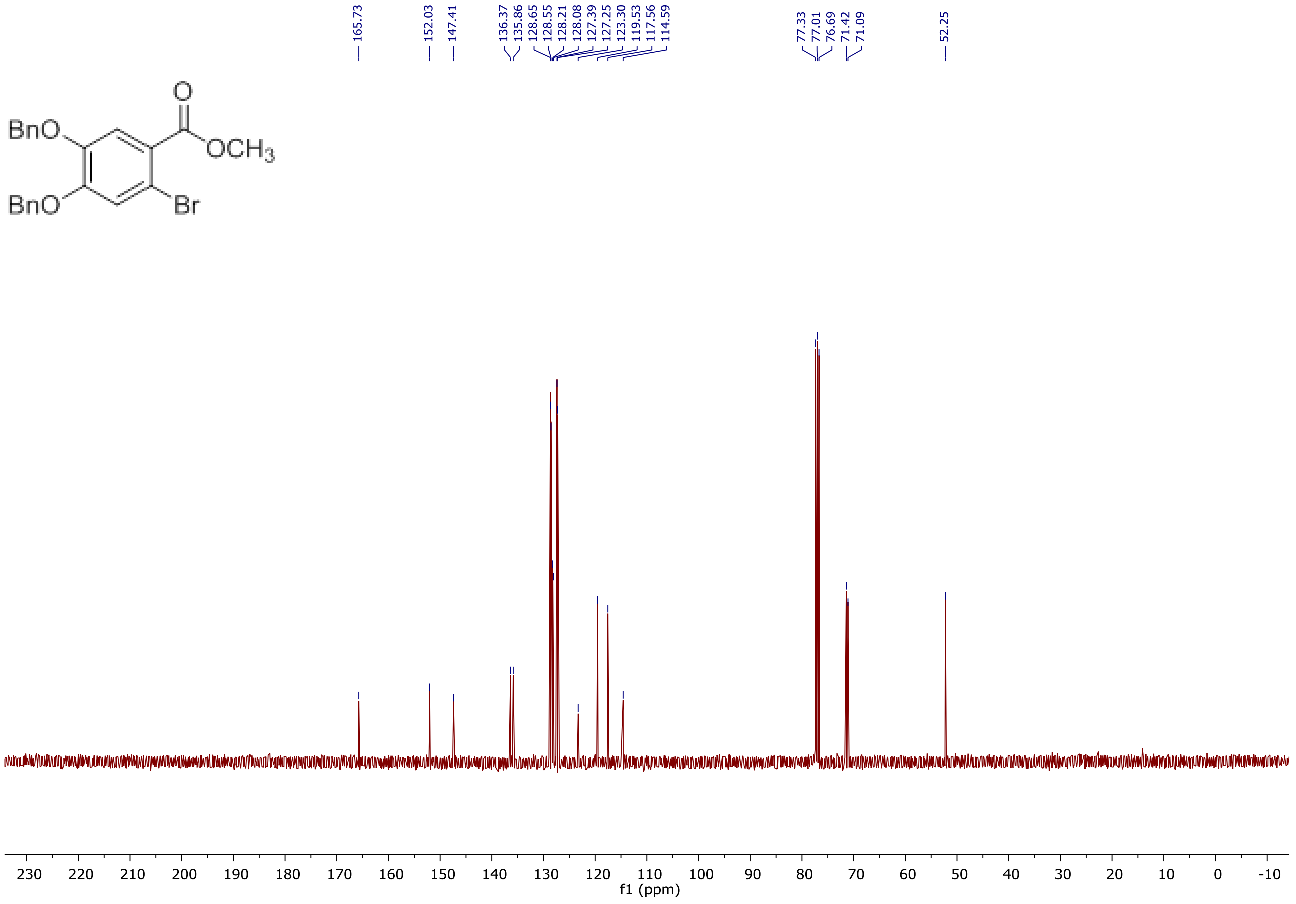

Methyl 4,5-bis(benzyloxy)-4'-methoxy-[1,1'-biphenyl]-2-carboxylate (**87**)

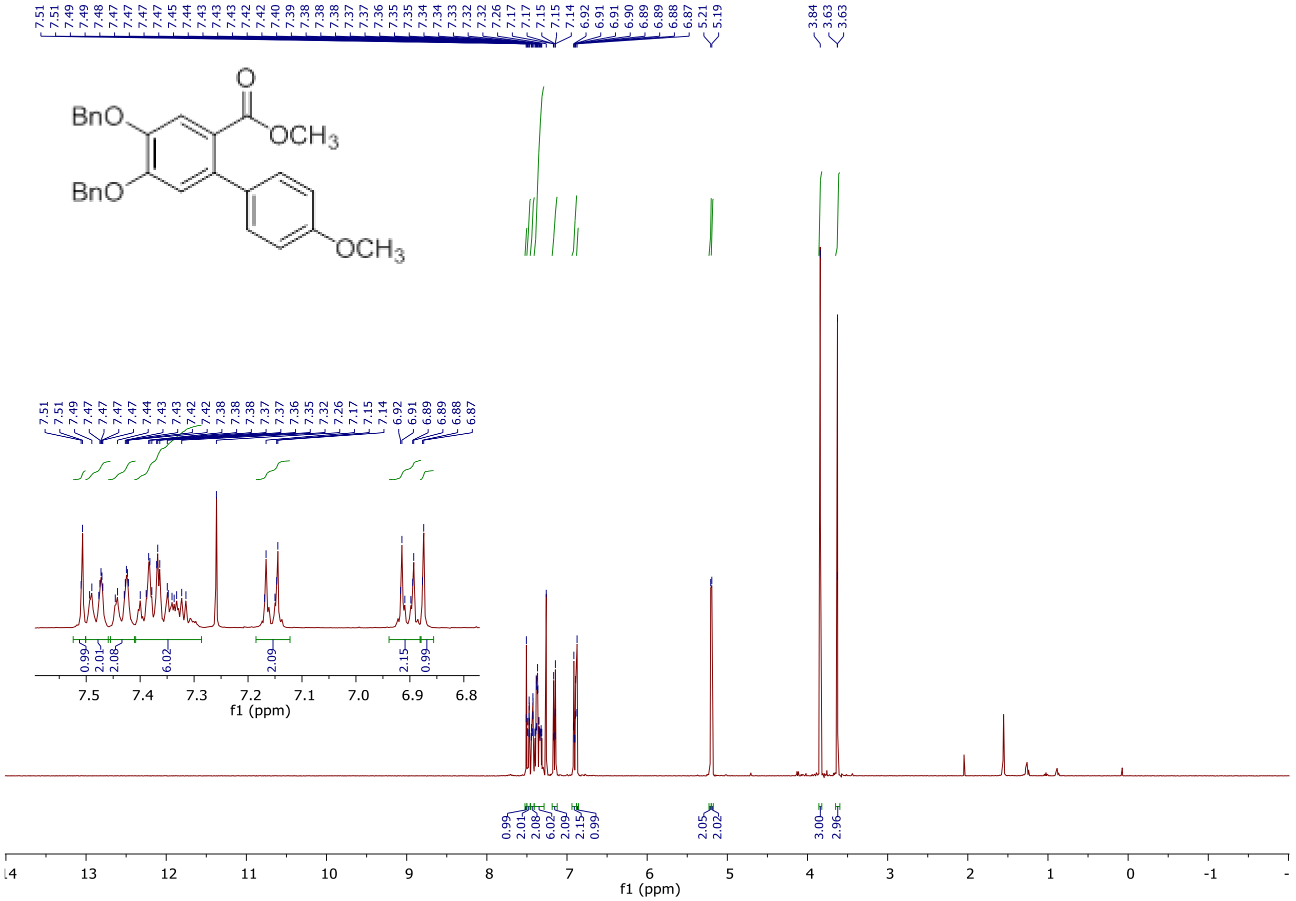

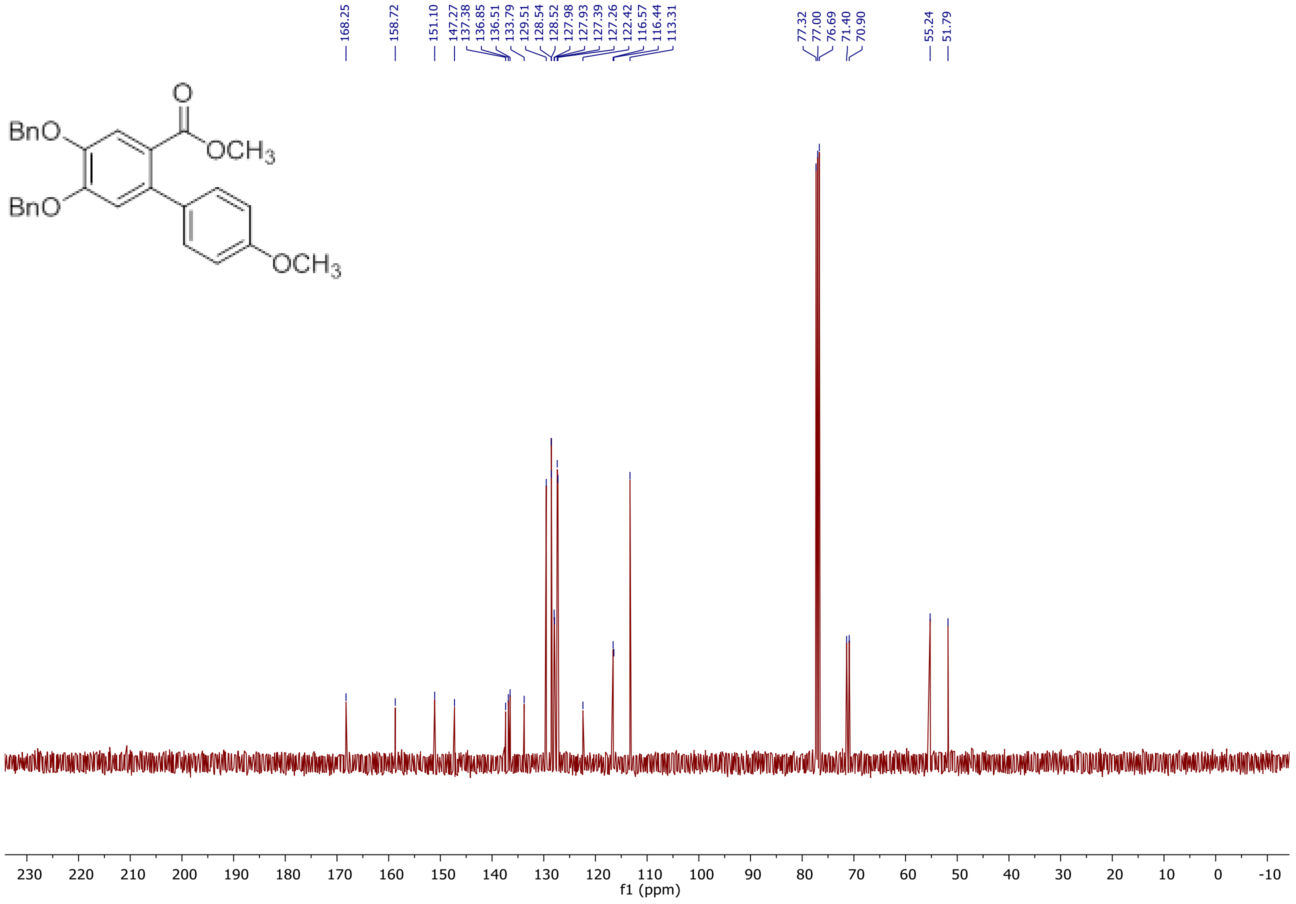

4,5-Bis(benzyloxy)-4'-methoxy-[1,1'-biphenyl]-2-carboxylic acid (**88**)

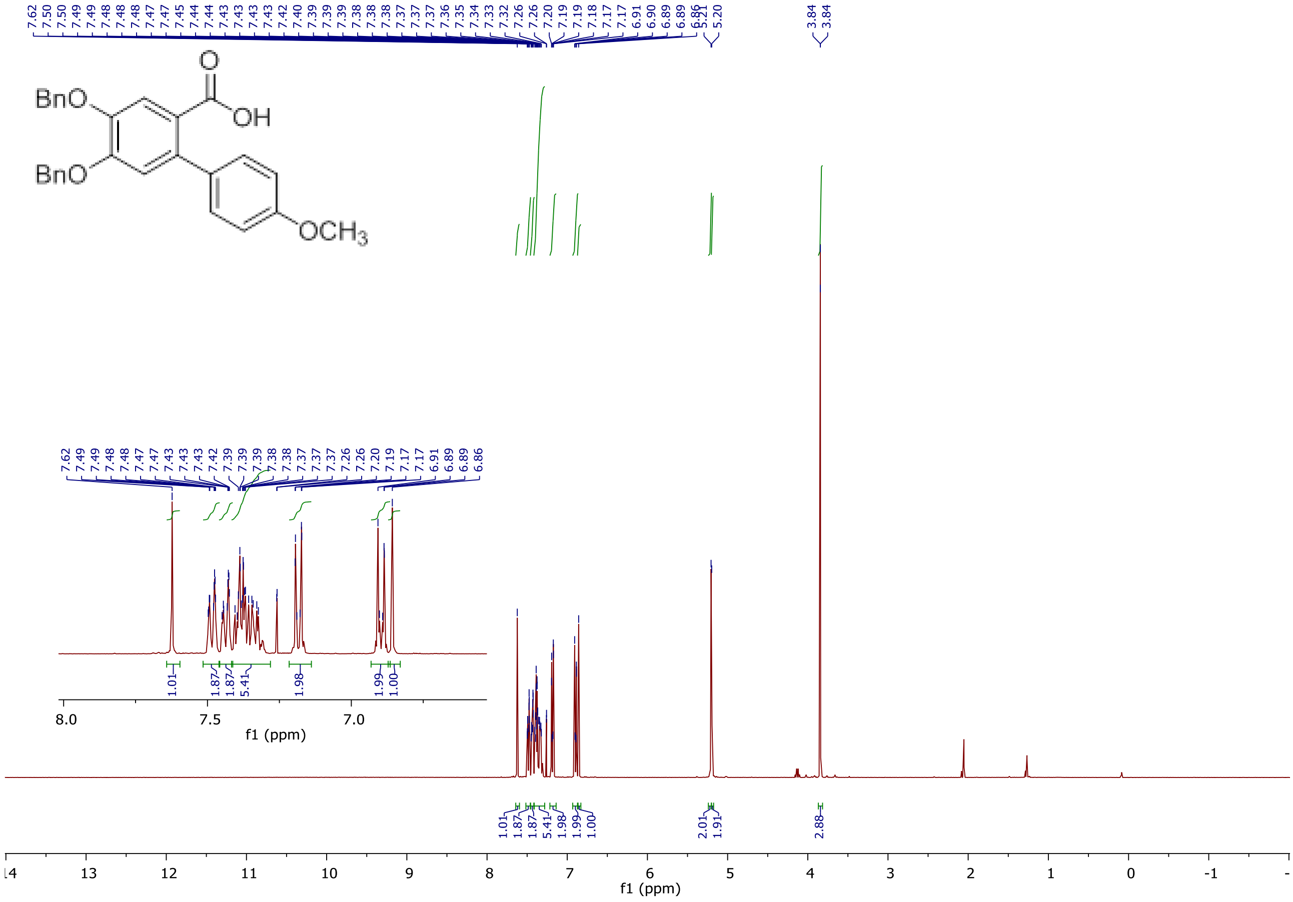

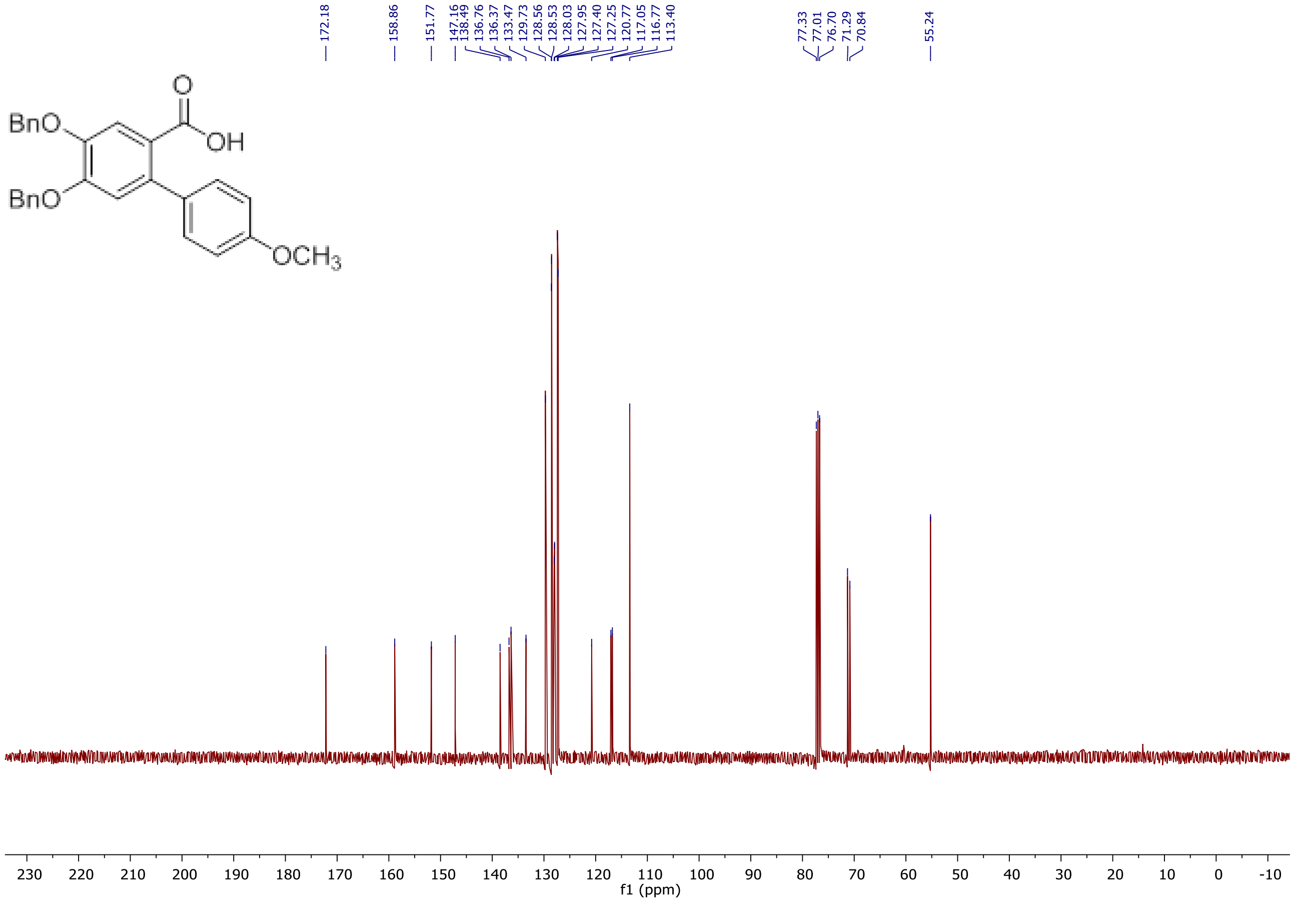

*8,9-Bis(benzyloxy)-3-methoxy-6H-benzo[c]chromen-6-one (**89**)*

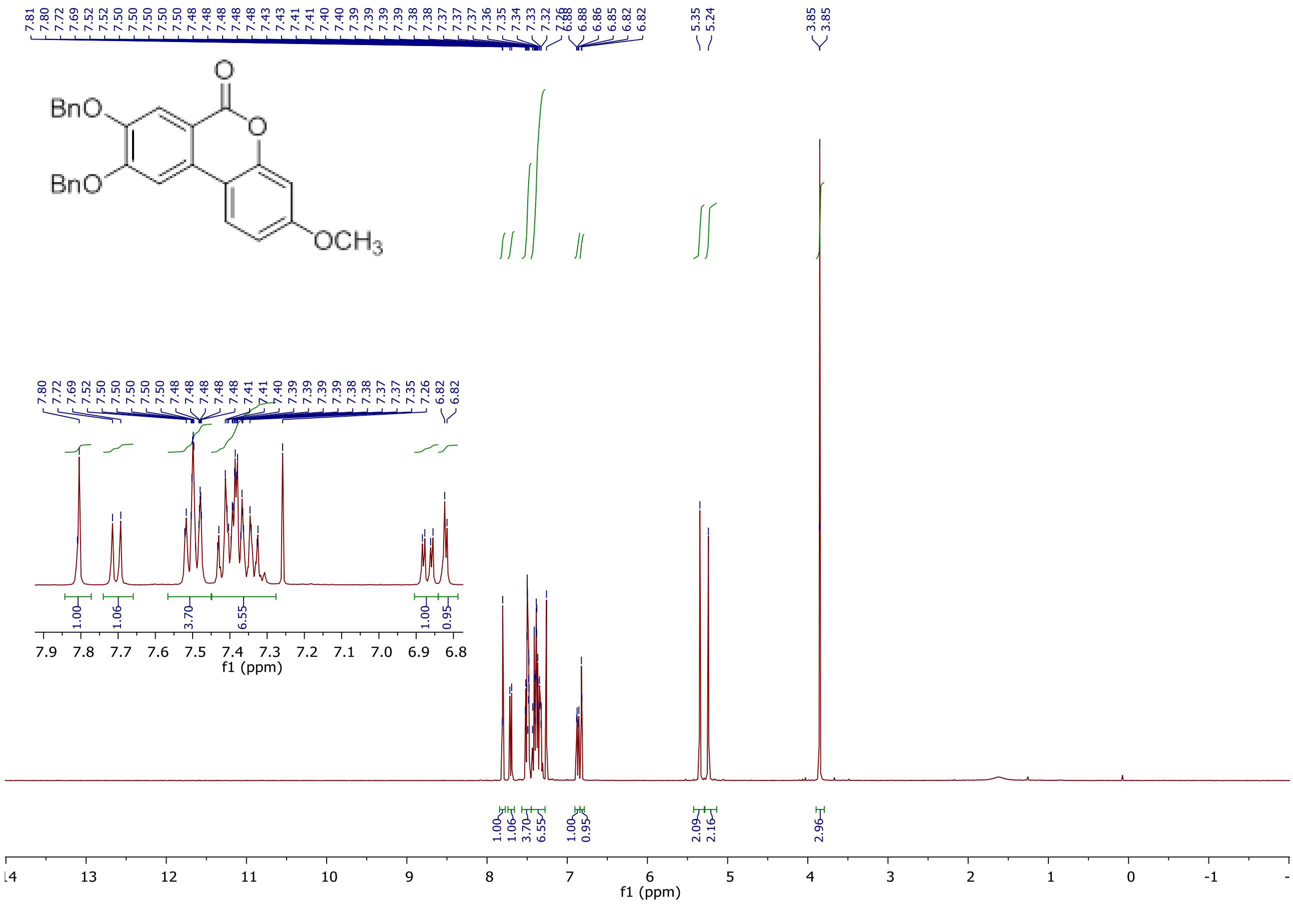

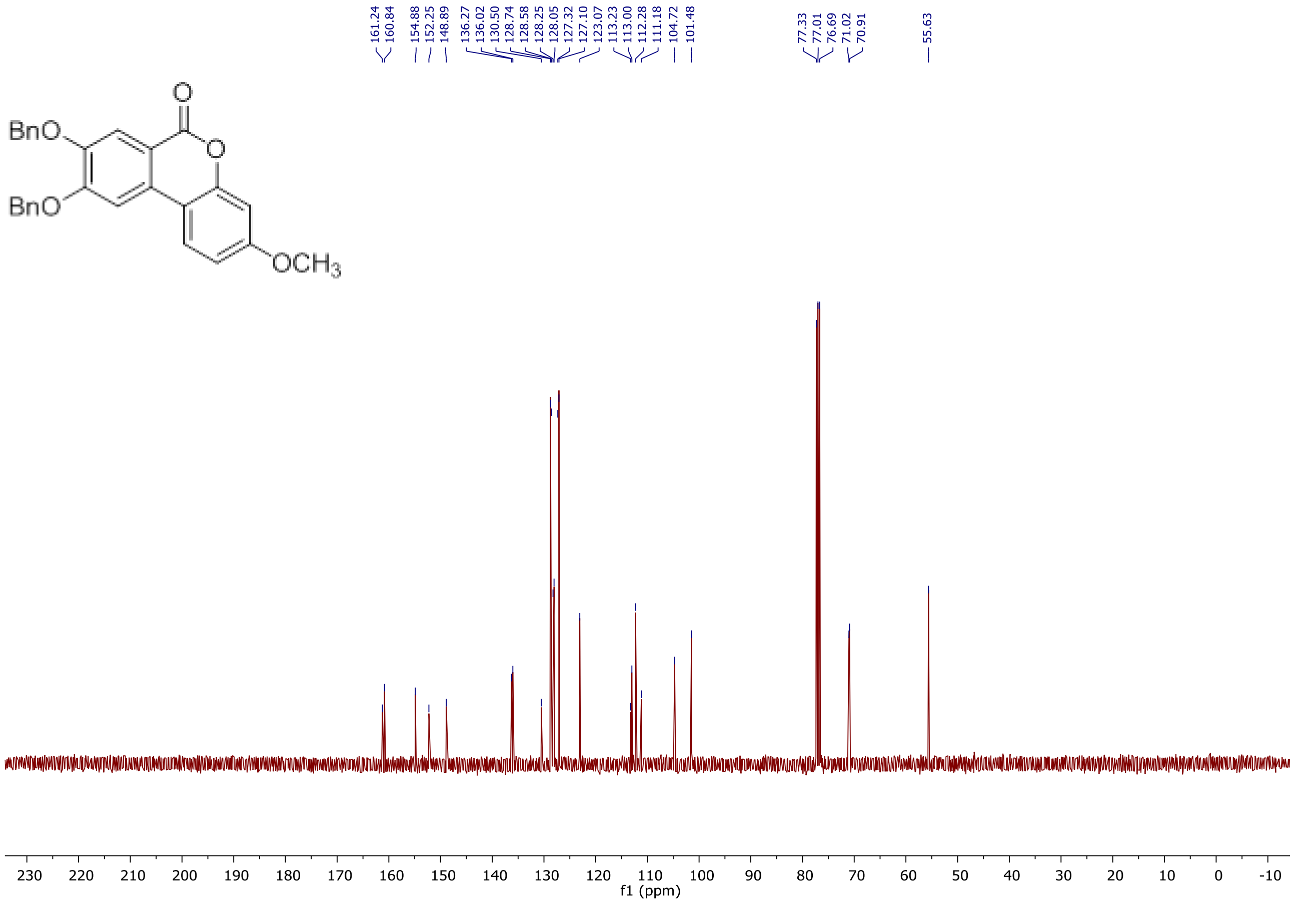

8,9-Dihydroxy-3-methoxy-6H-benzo[c]chromen-6-one (***90***)

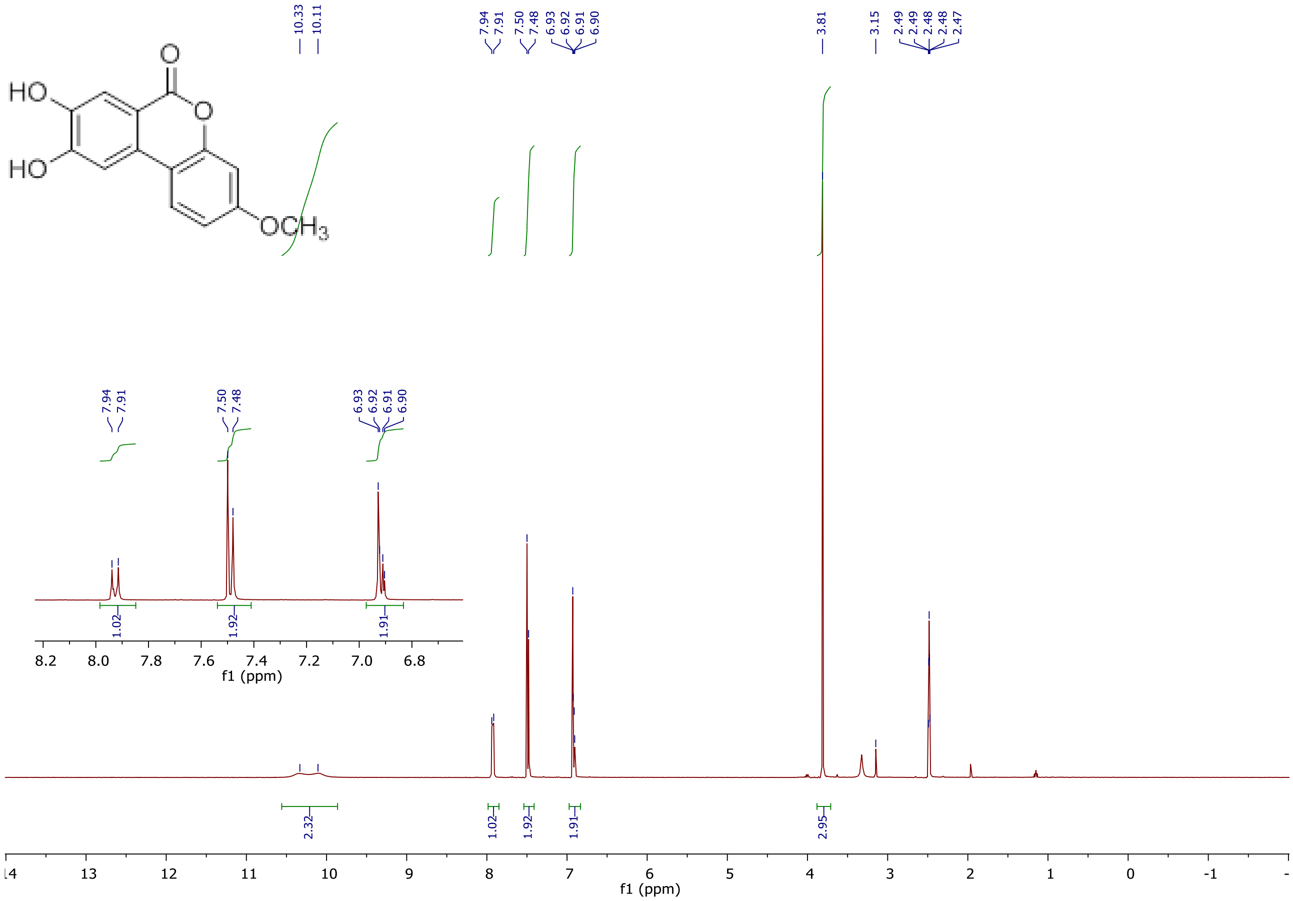

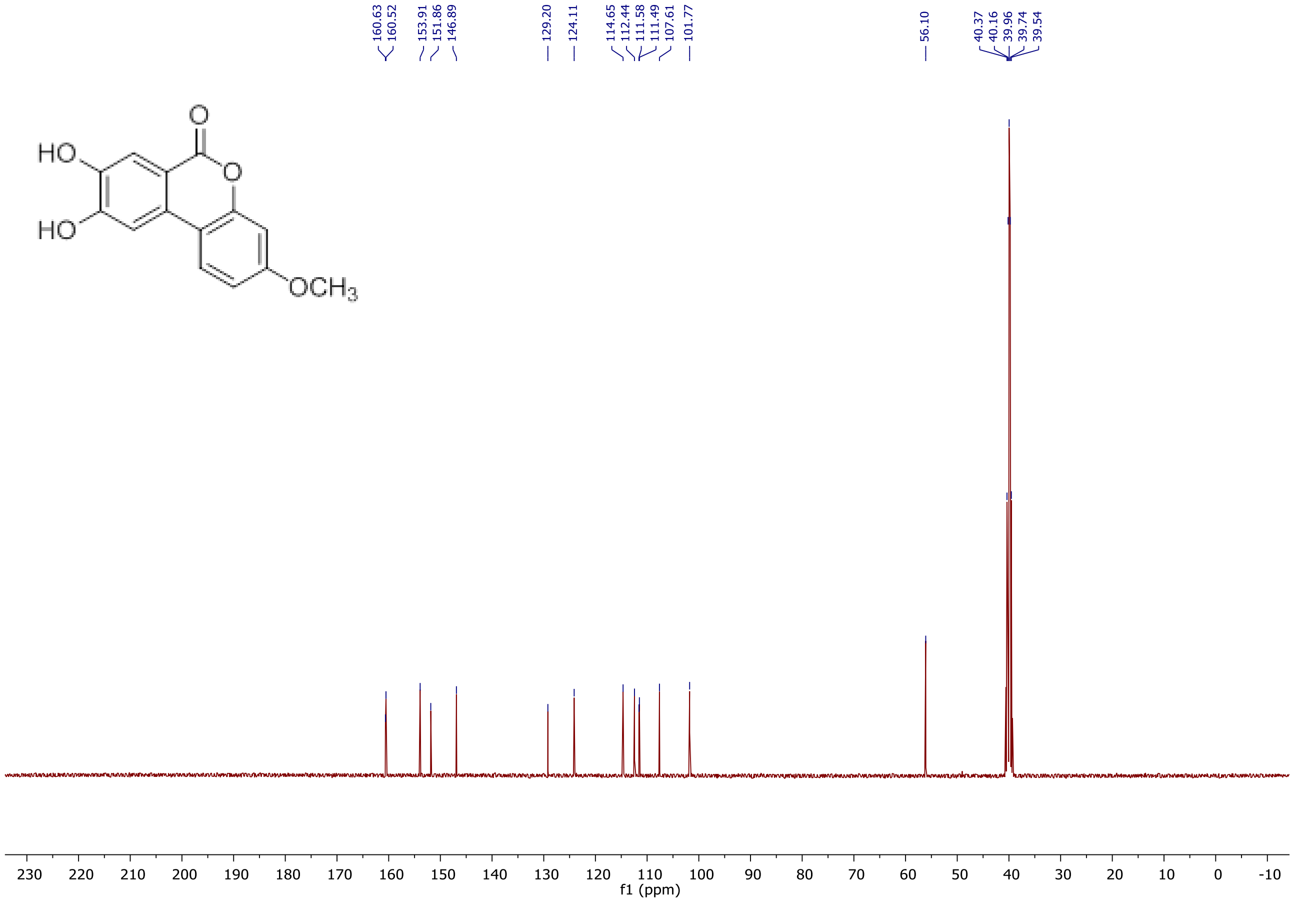

4,5-Bis(benzyloxy)-2-bromobenzoic acid (**91**)

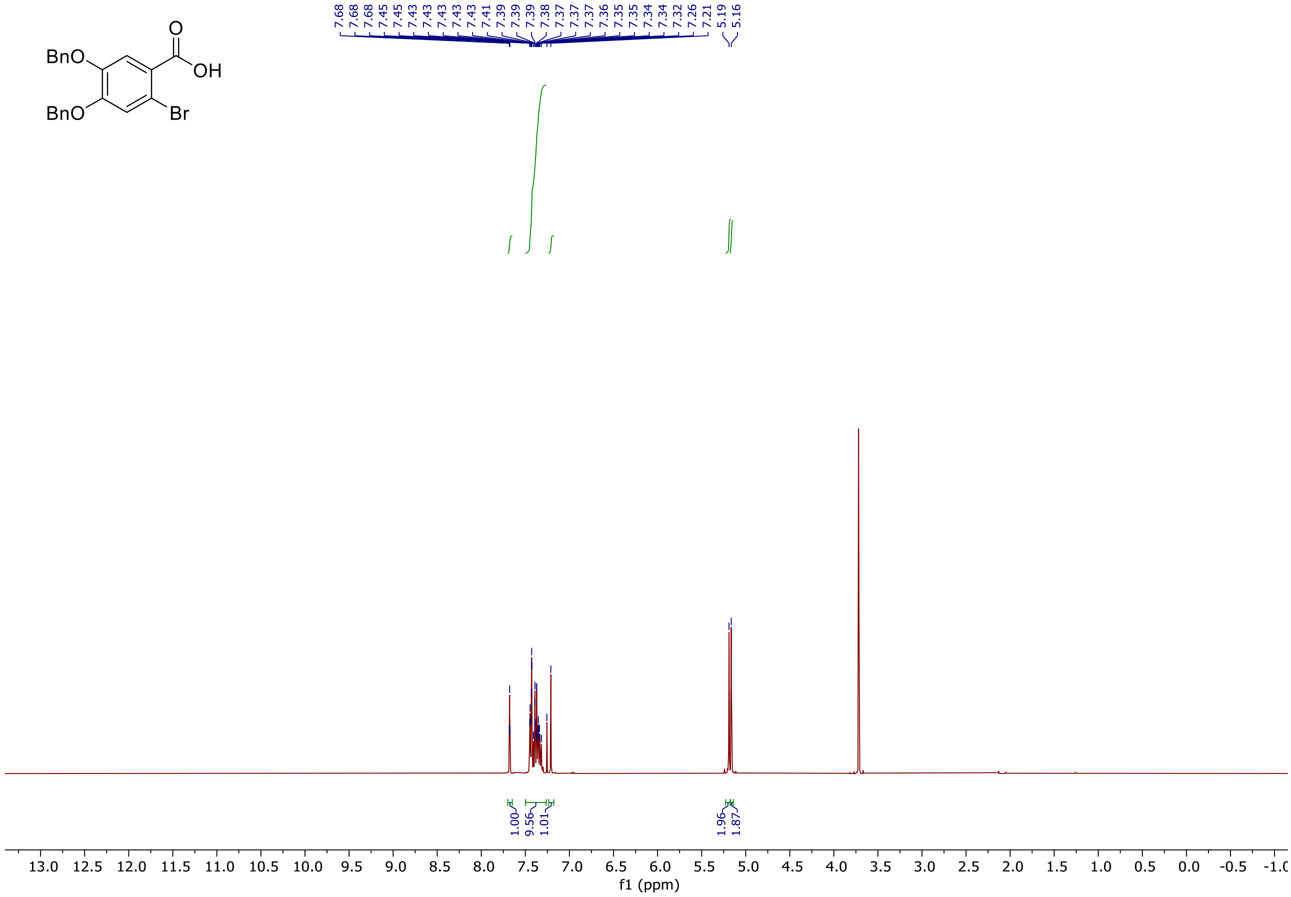

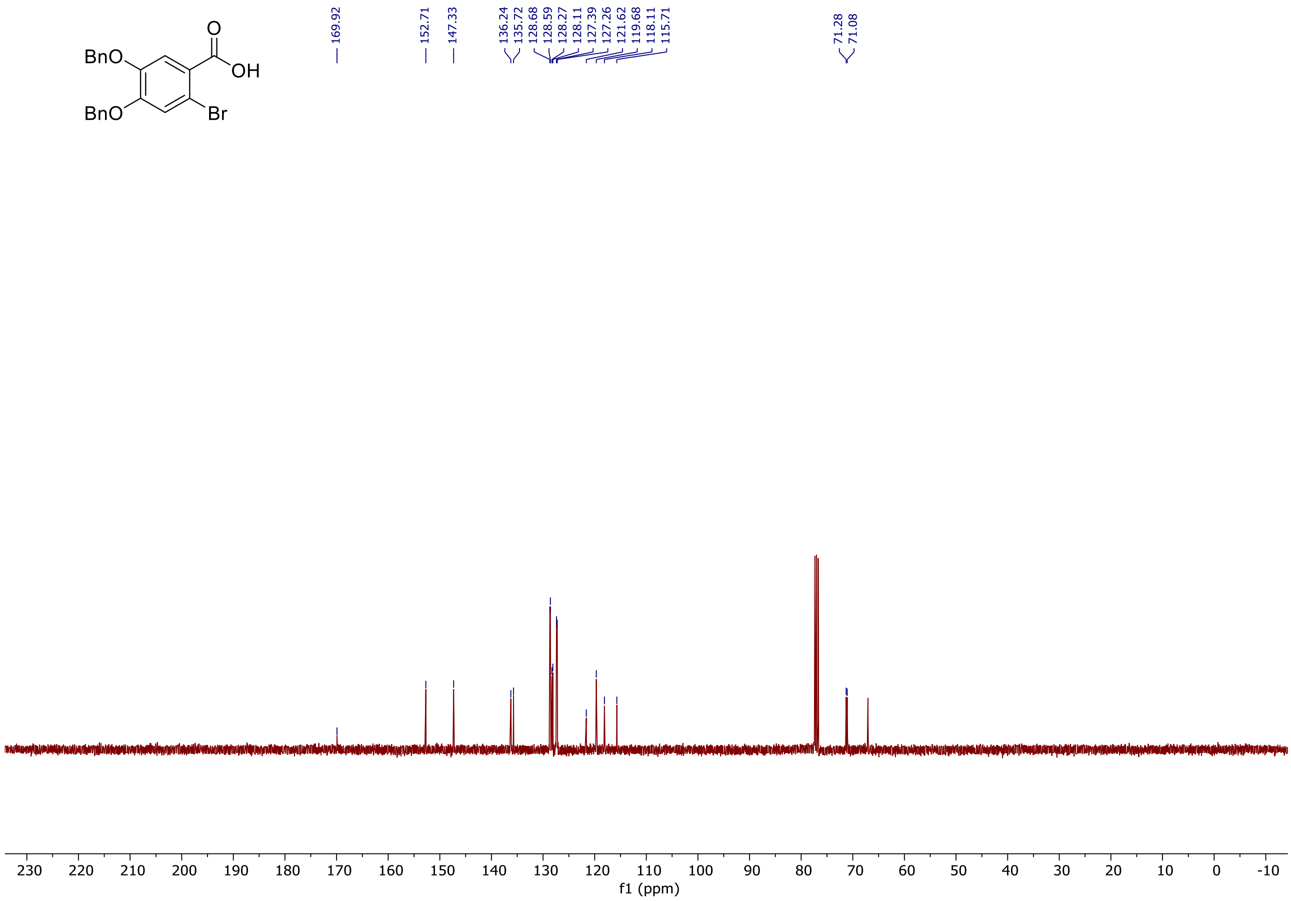

8,9-Bis(benzyloxy)-3-hydroxy-6H-benzo[c]chromen-6-one (***93***)

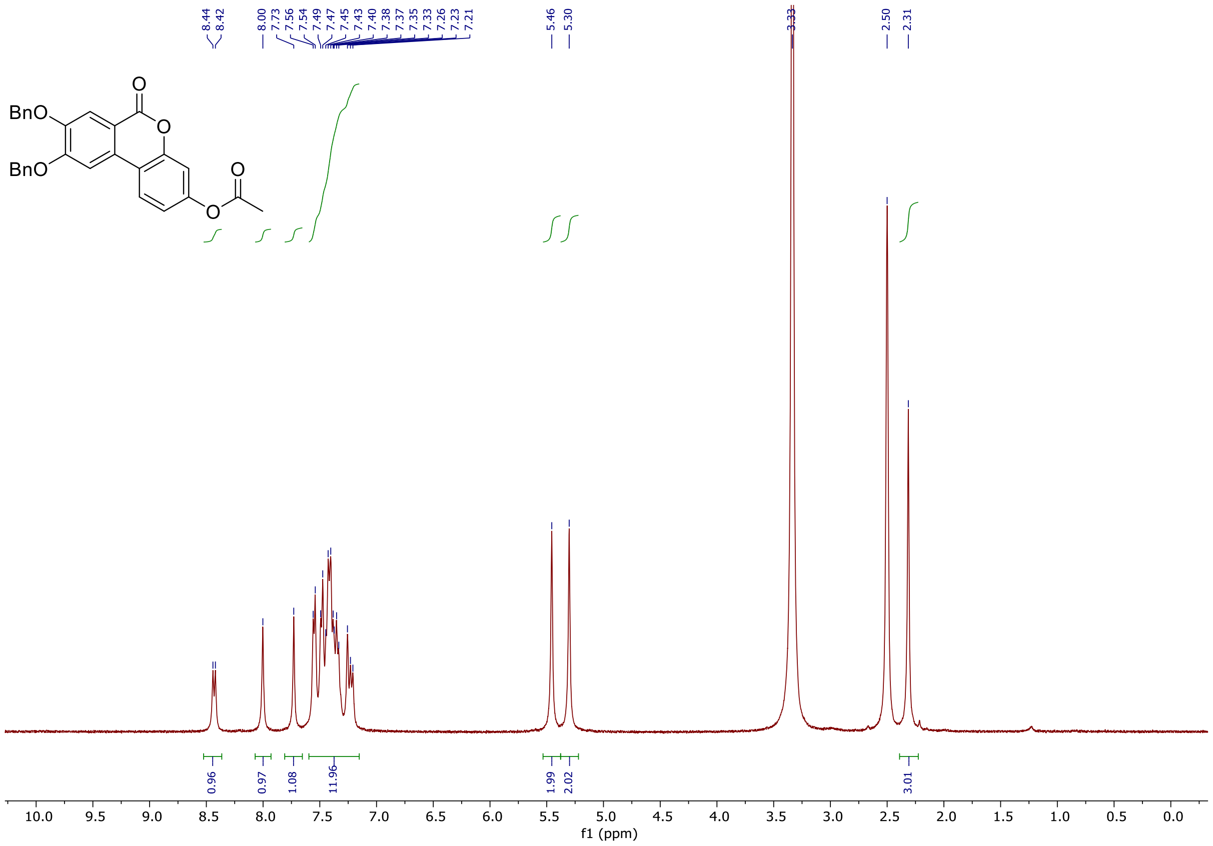

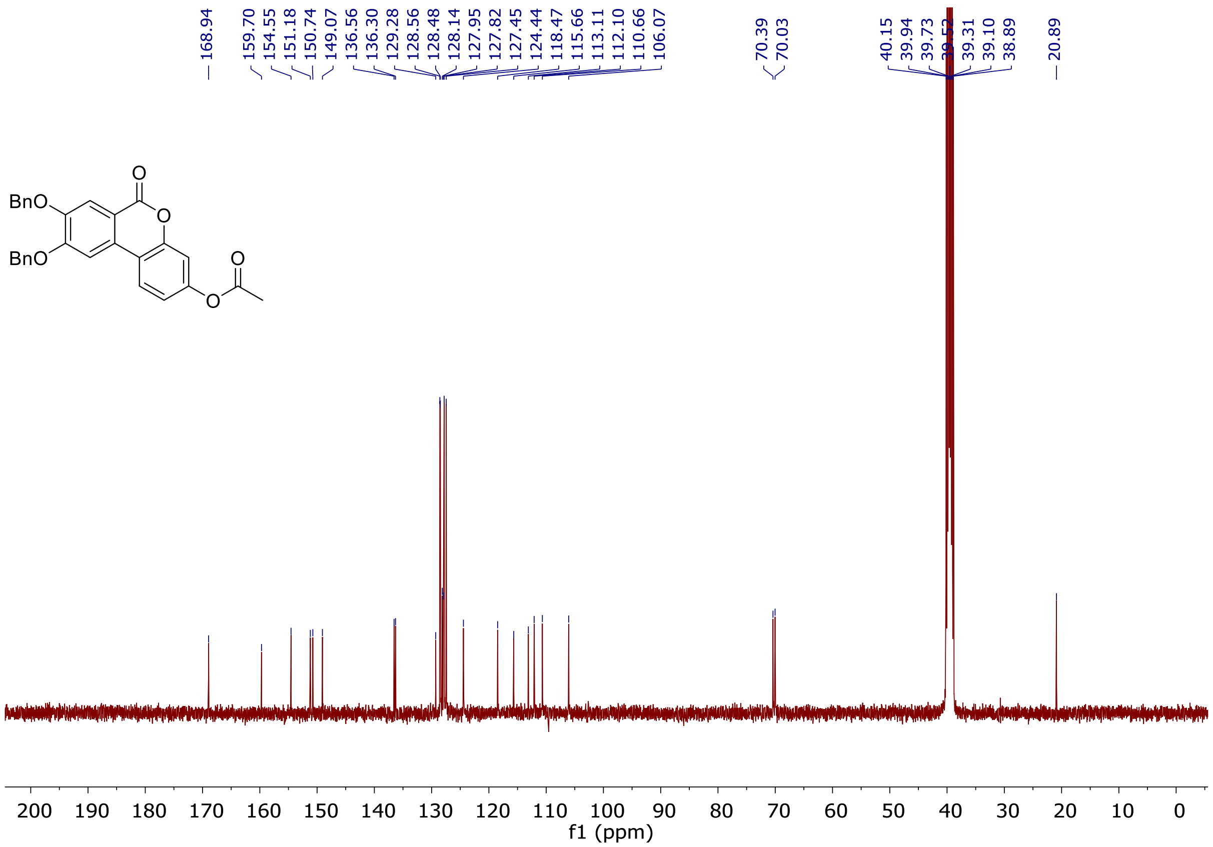

8,9-Dihydroxy-6-oxo-6H-benzo[c]chromen-3-yl acetate (**94**)

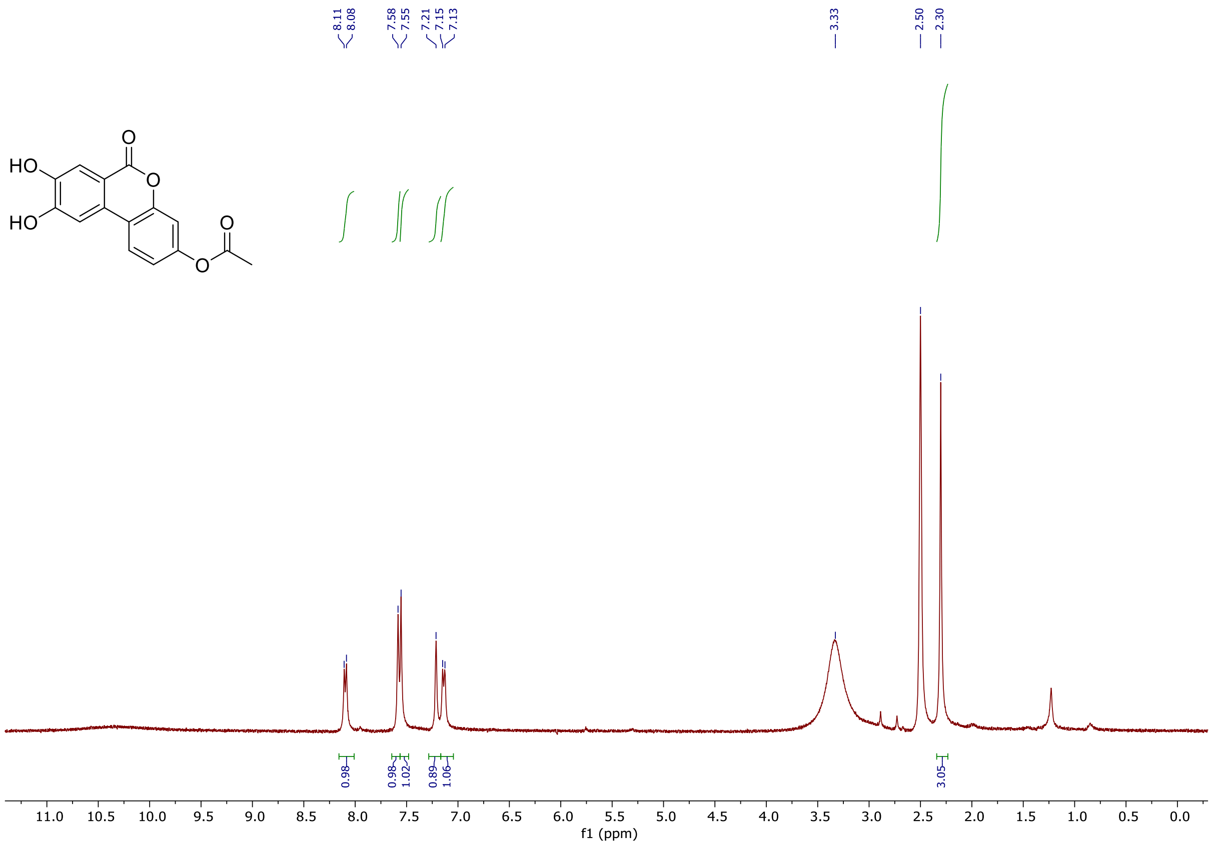

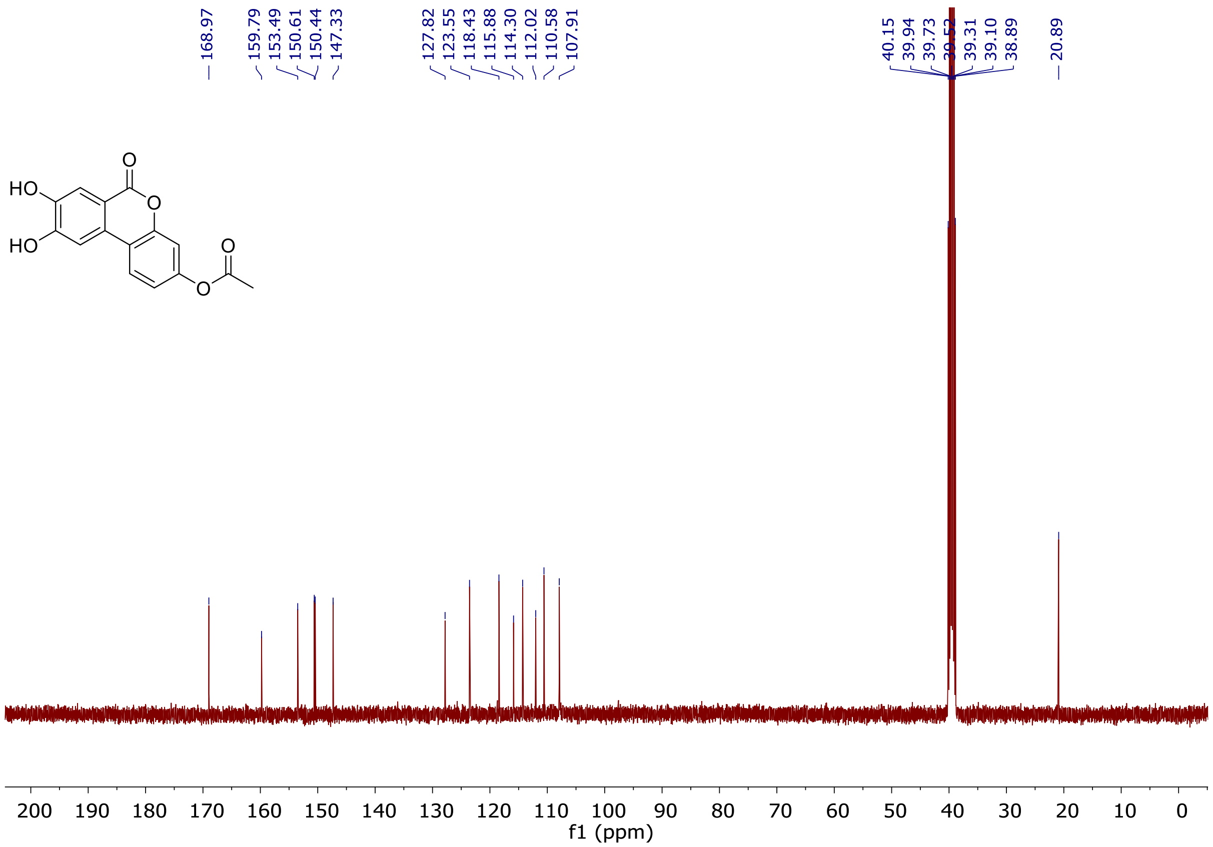

1H-Indol-4-yl 4,5-bis(benzyloxy)-2-bromobenzoate (**96**)

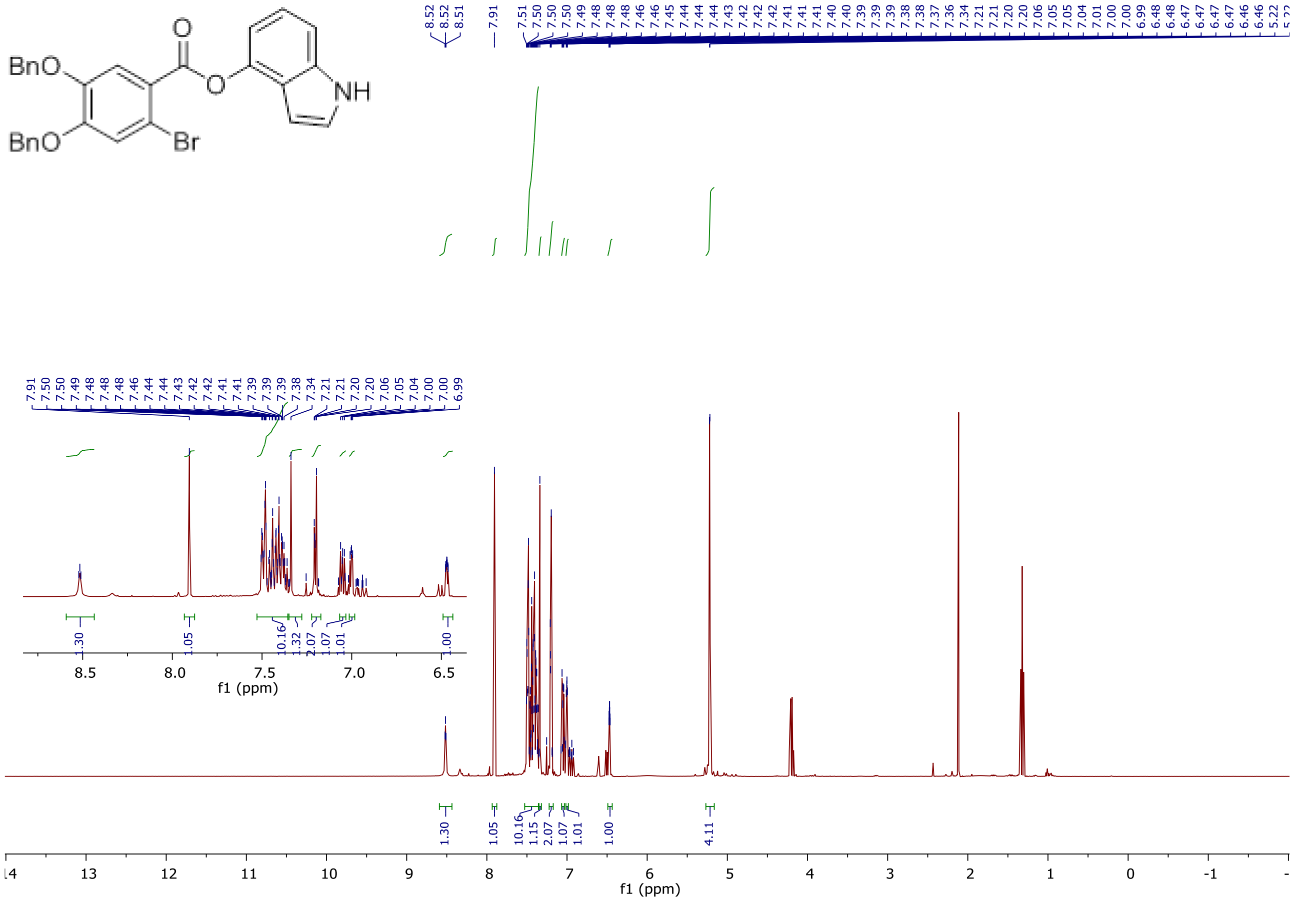

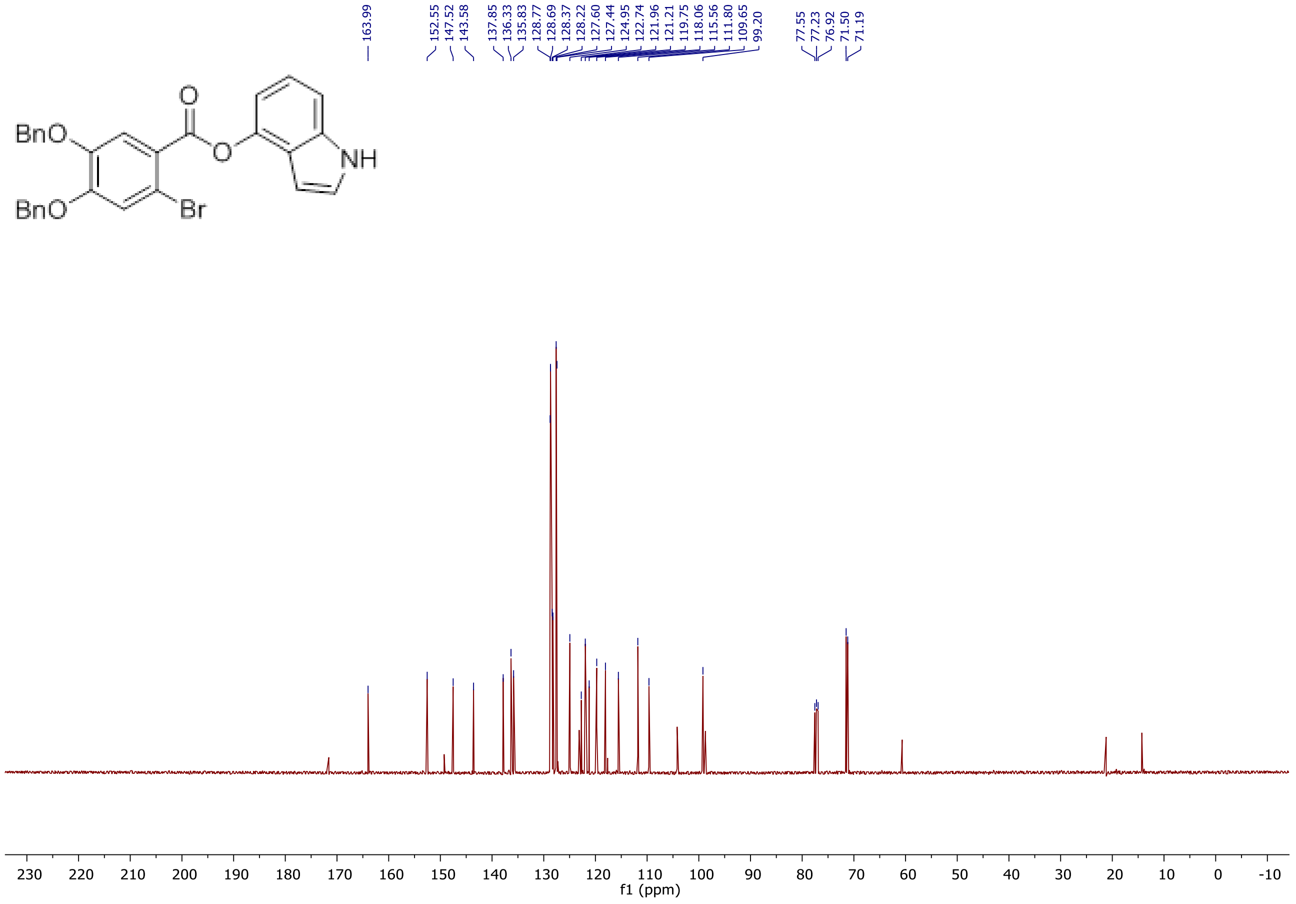

7,8-Bis(benzyloxy)isochromeno[3,4-e]indol-5(1H)-one (**97**)

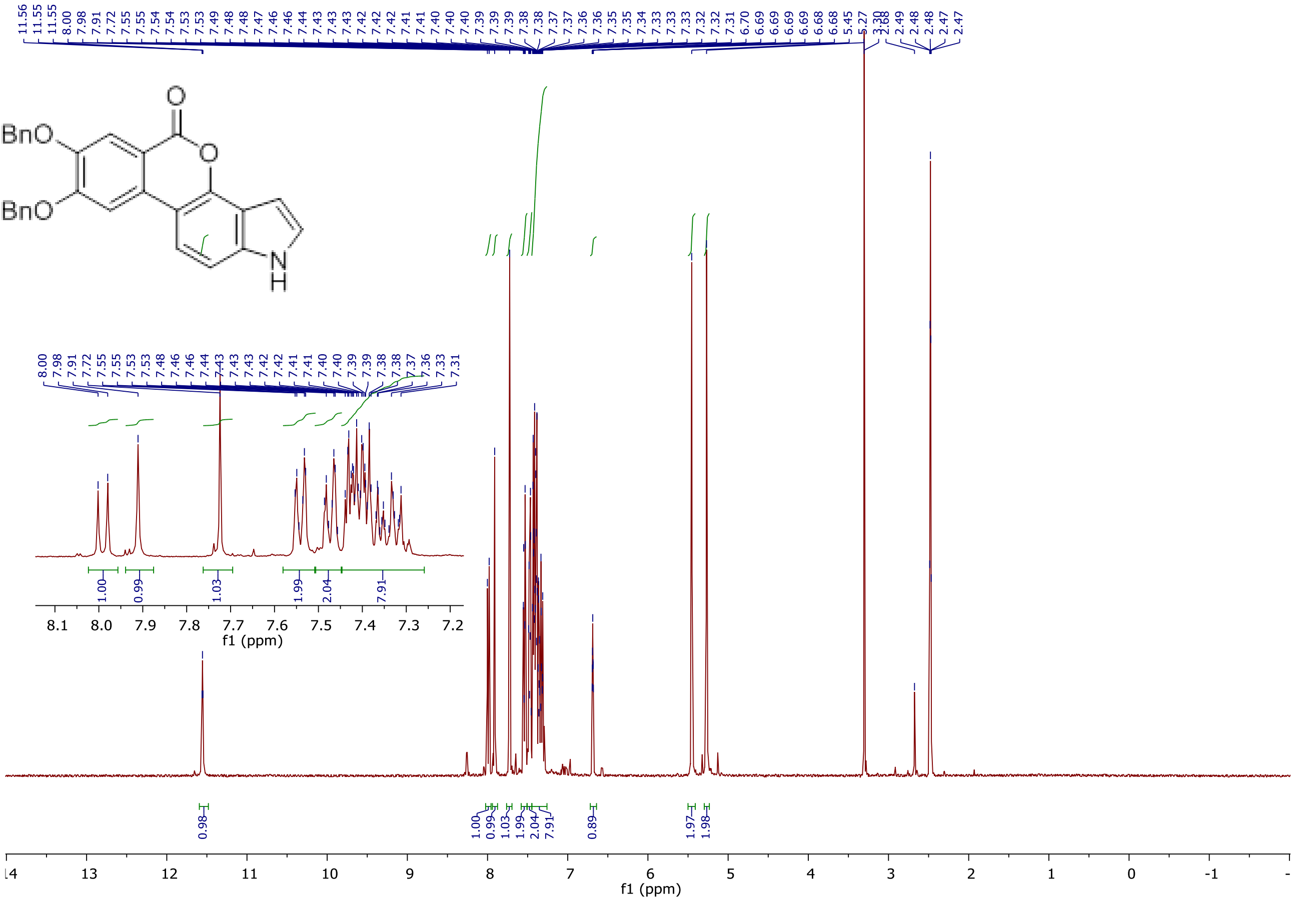

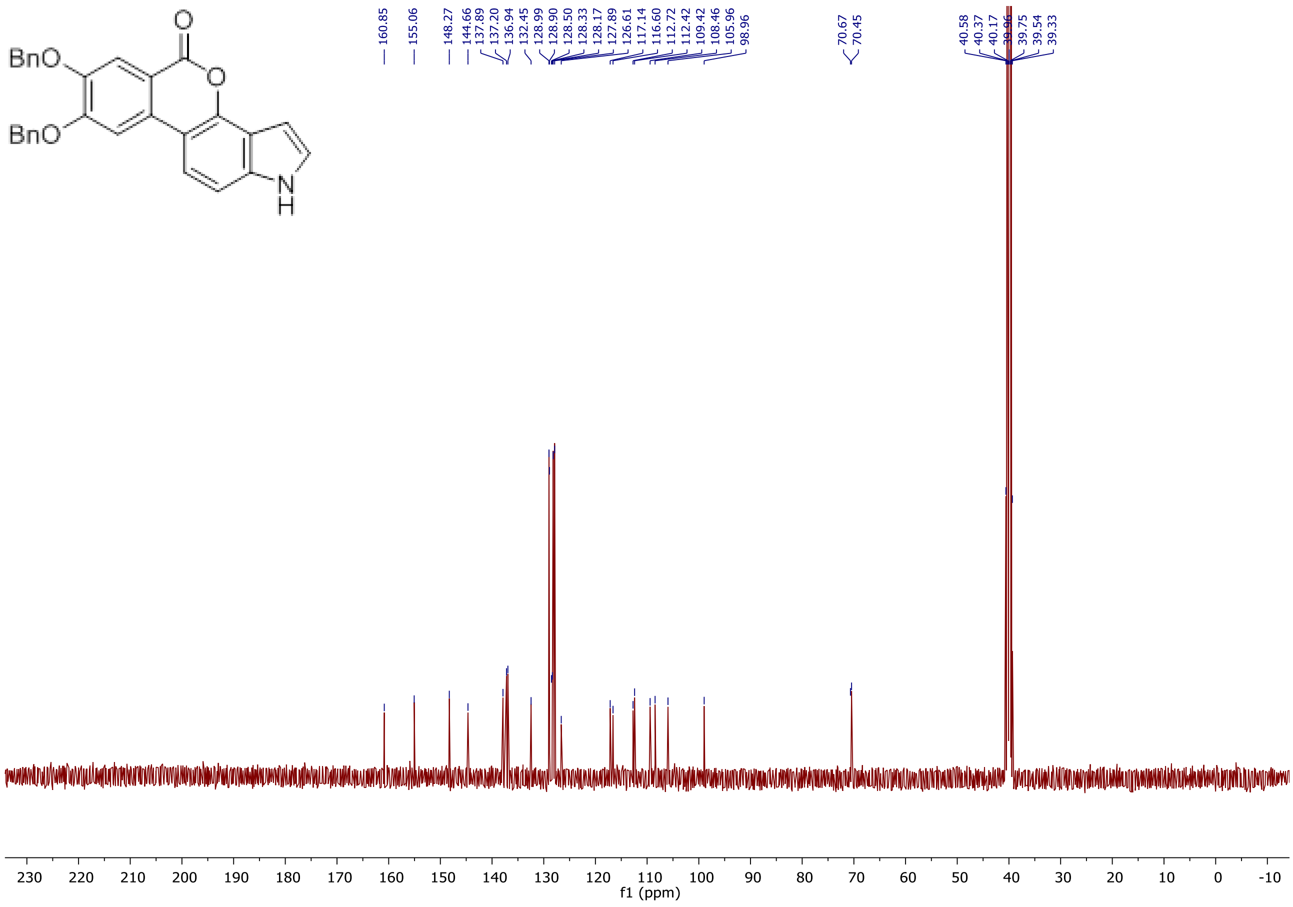

7,8-Dihydroxyisochromeno[3,4-e]indol-5(1H)-one (**98**)

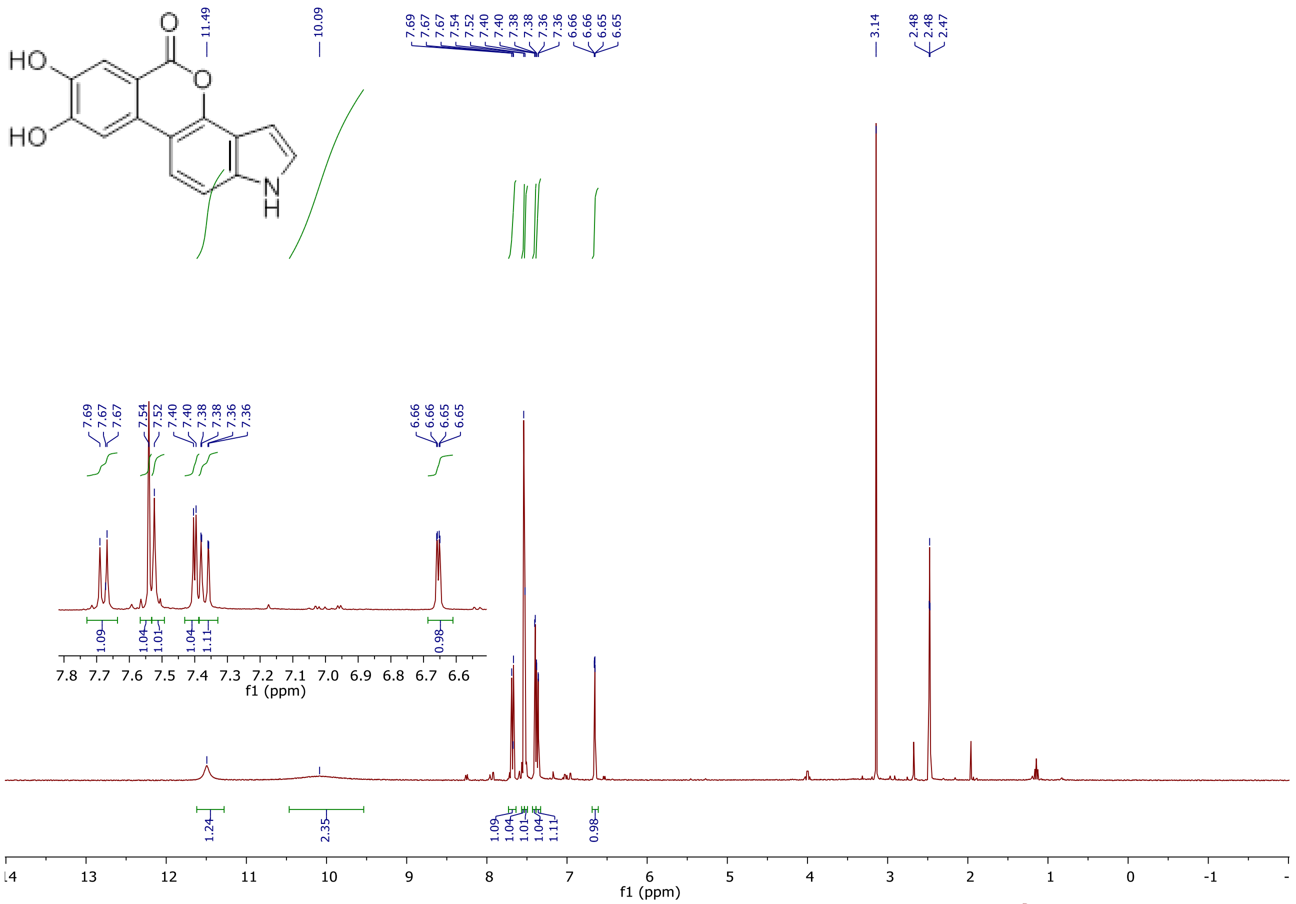

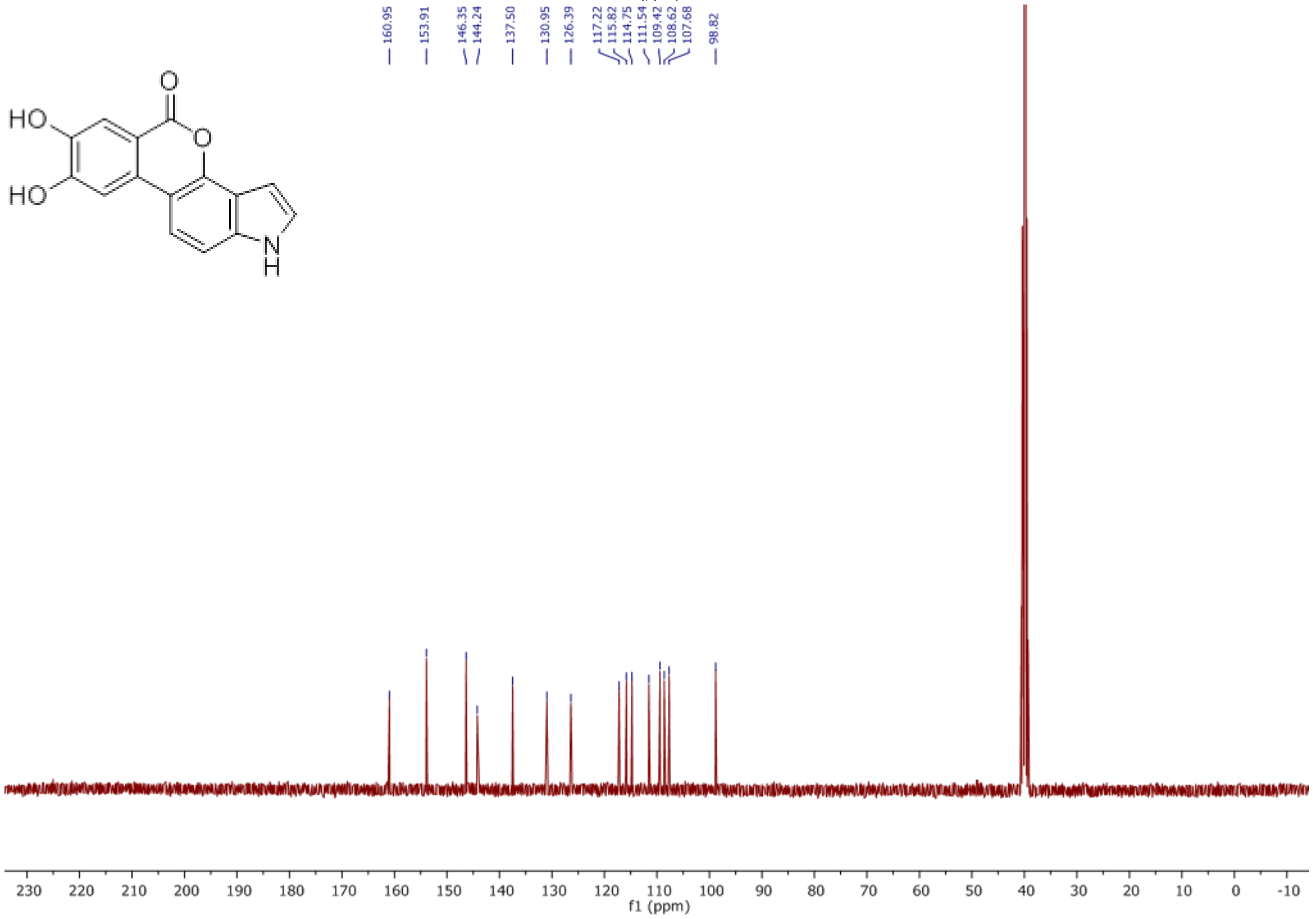

Methyl 4,5-dimethoxy-2-(naphthalen-2-yl)benzoate (**100**)

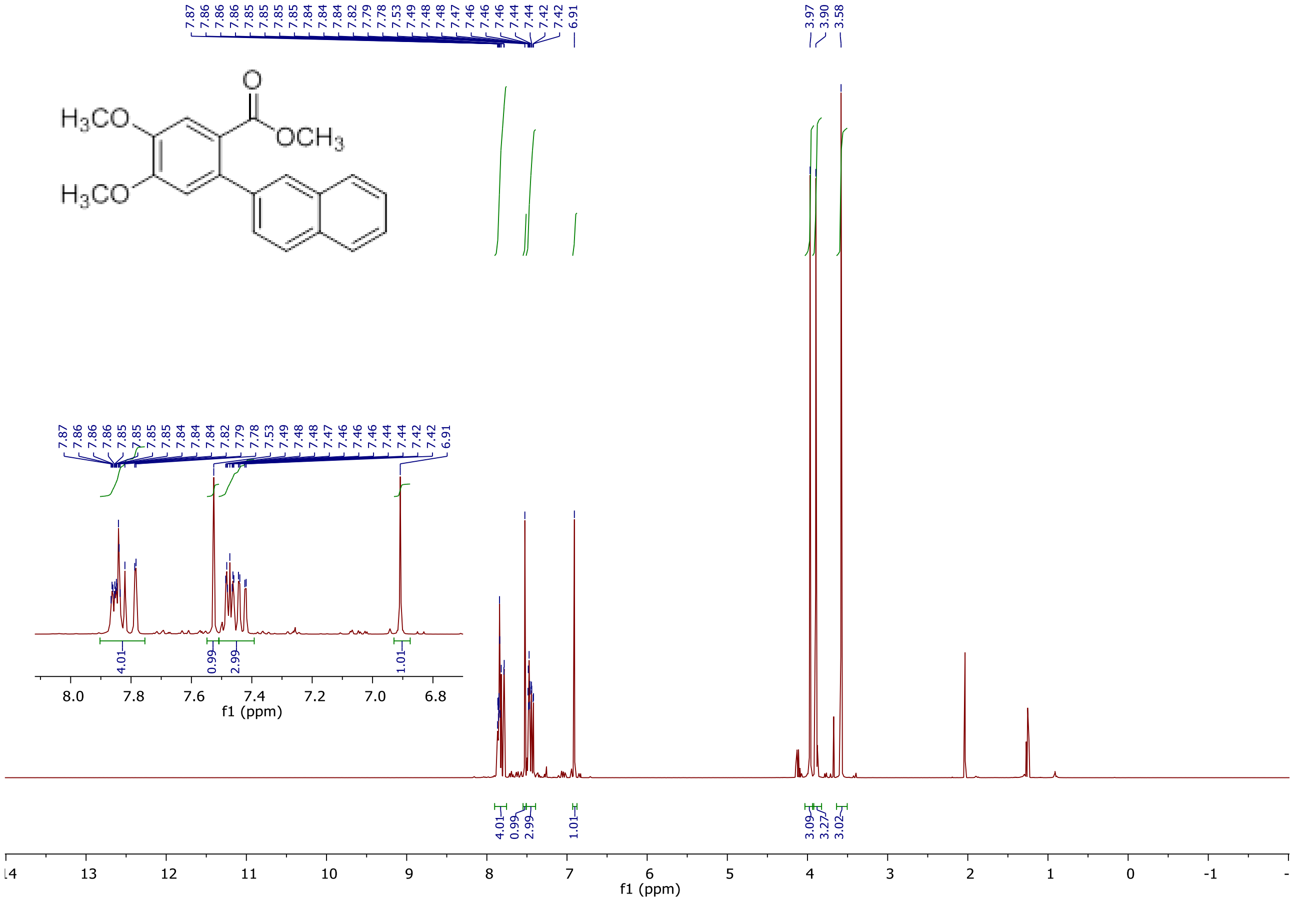

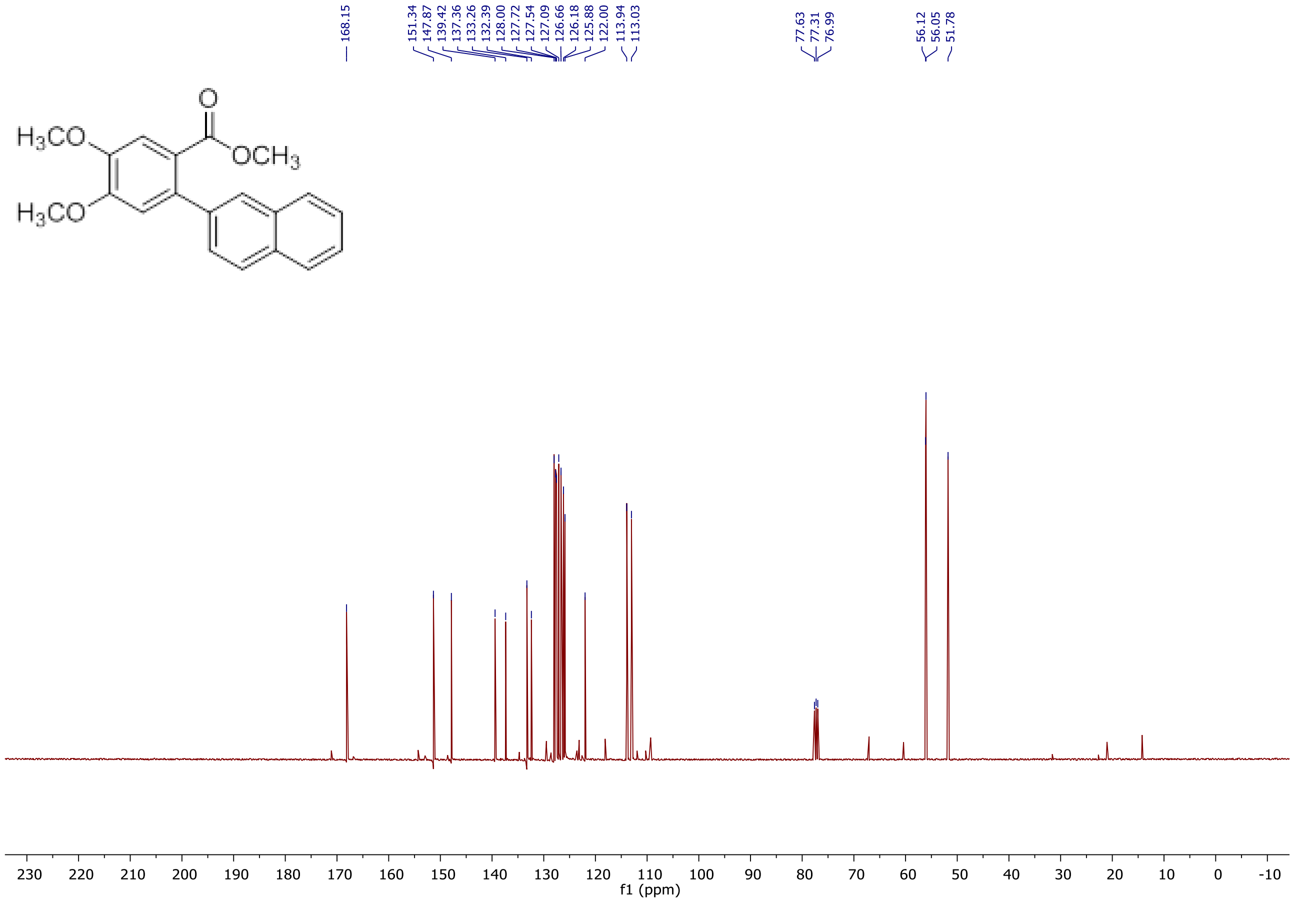

4,5-Dimethoxy-2-(naphthalen-2-yl)benzoic acid (**101**)

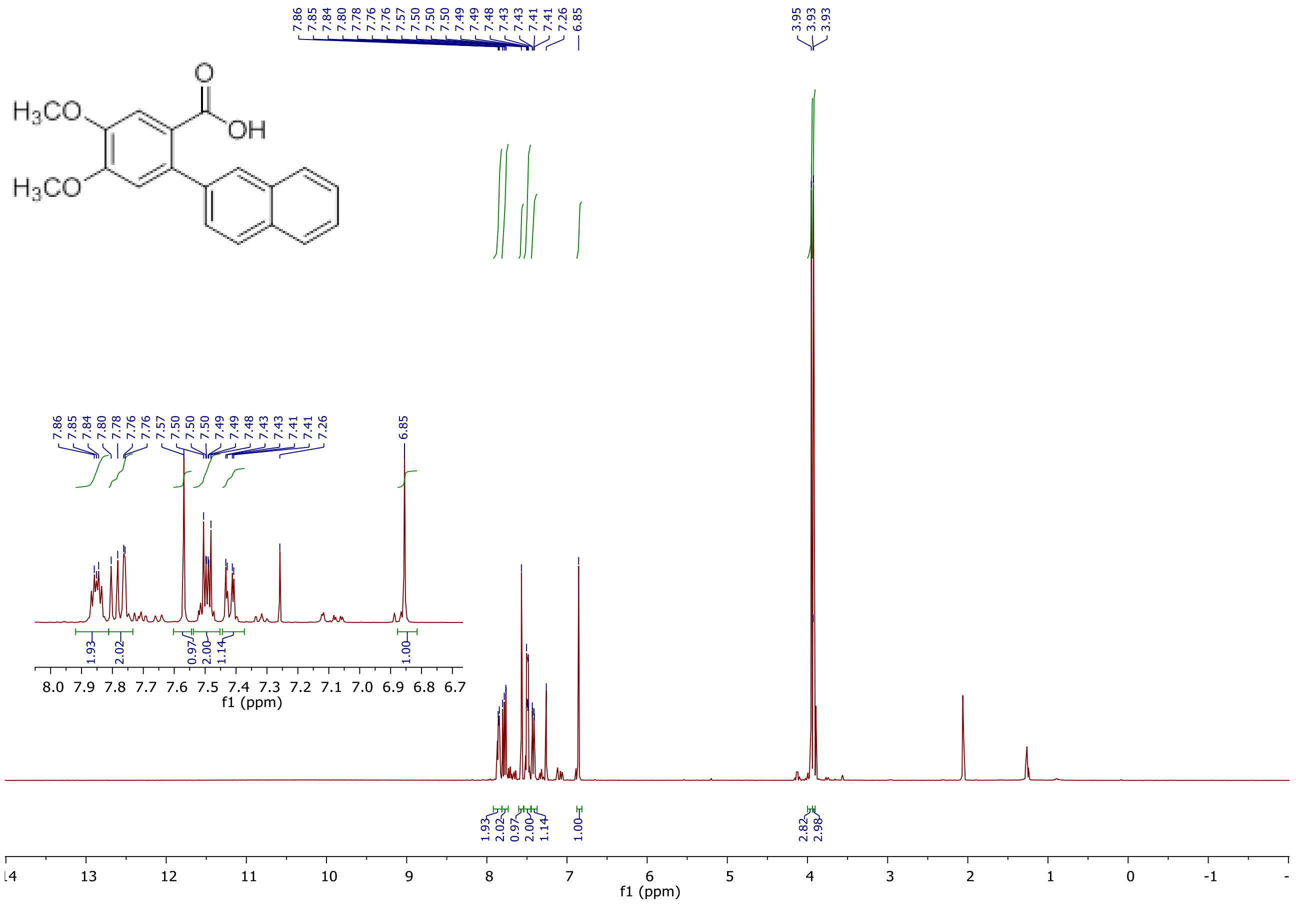

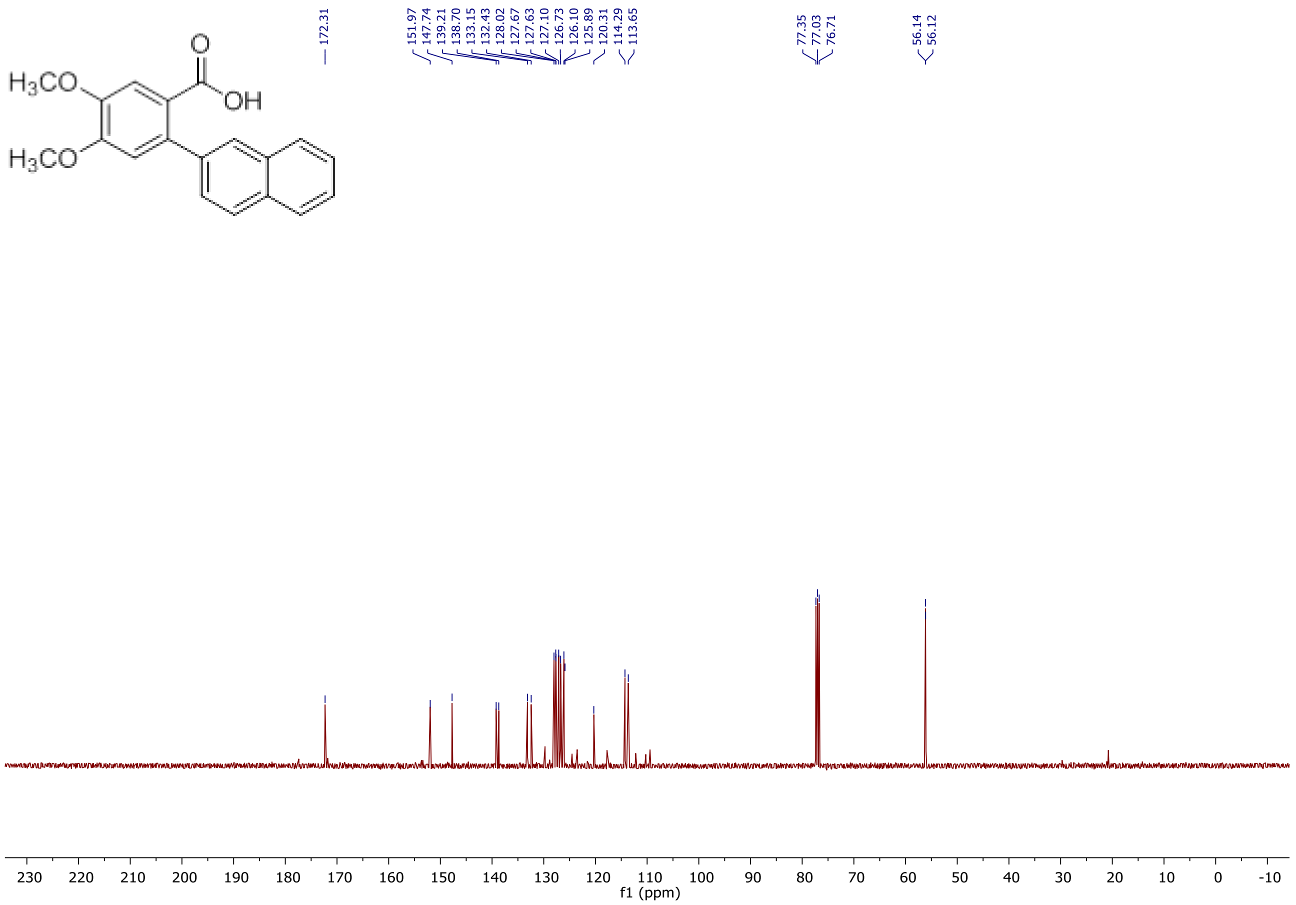

8,9-dimethoxy-6H-dibenzo[c,h]chromen-6-one (**102**)

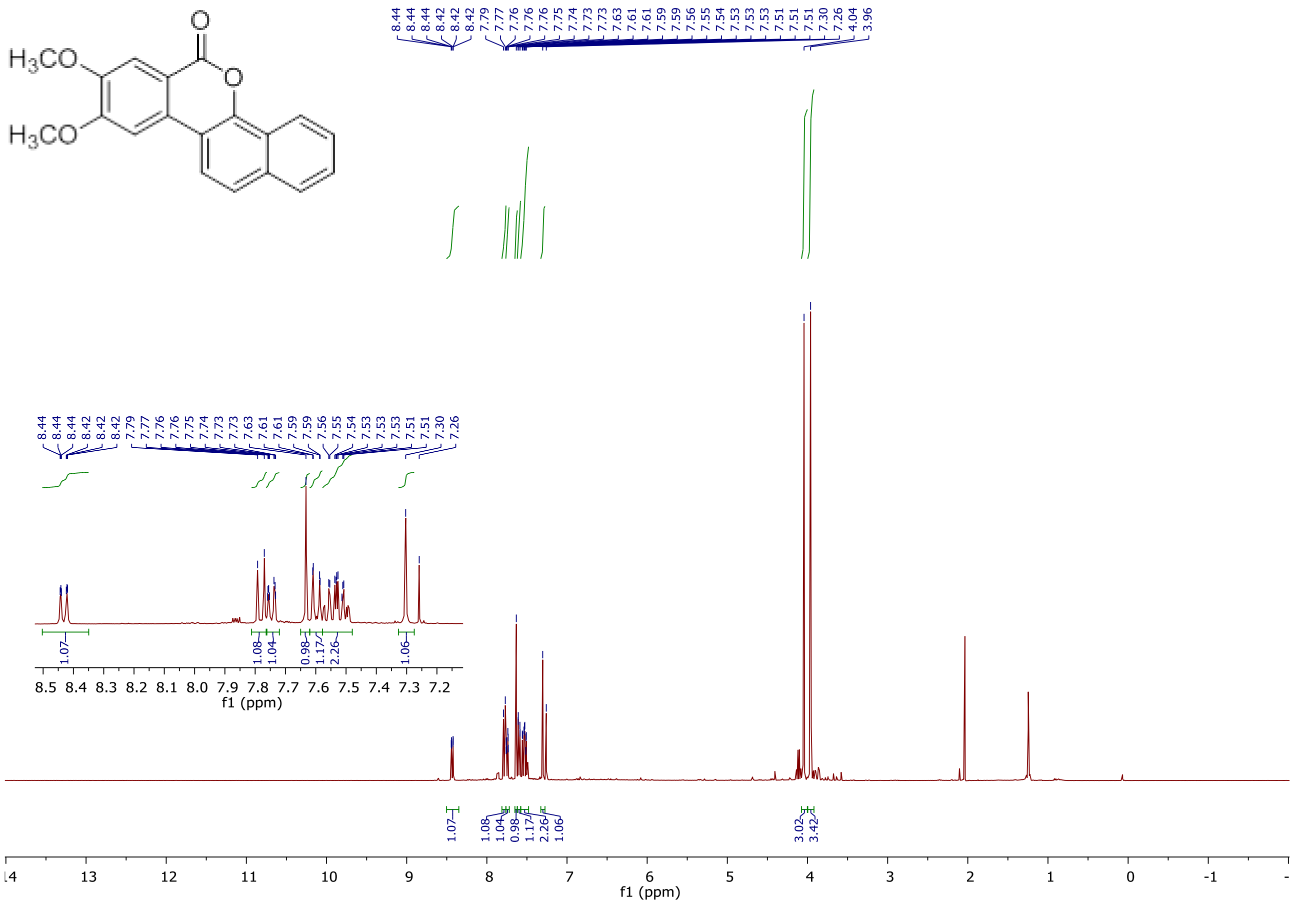

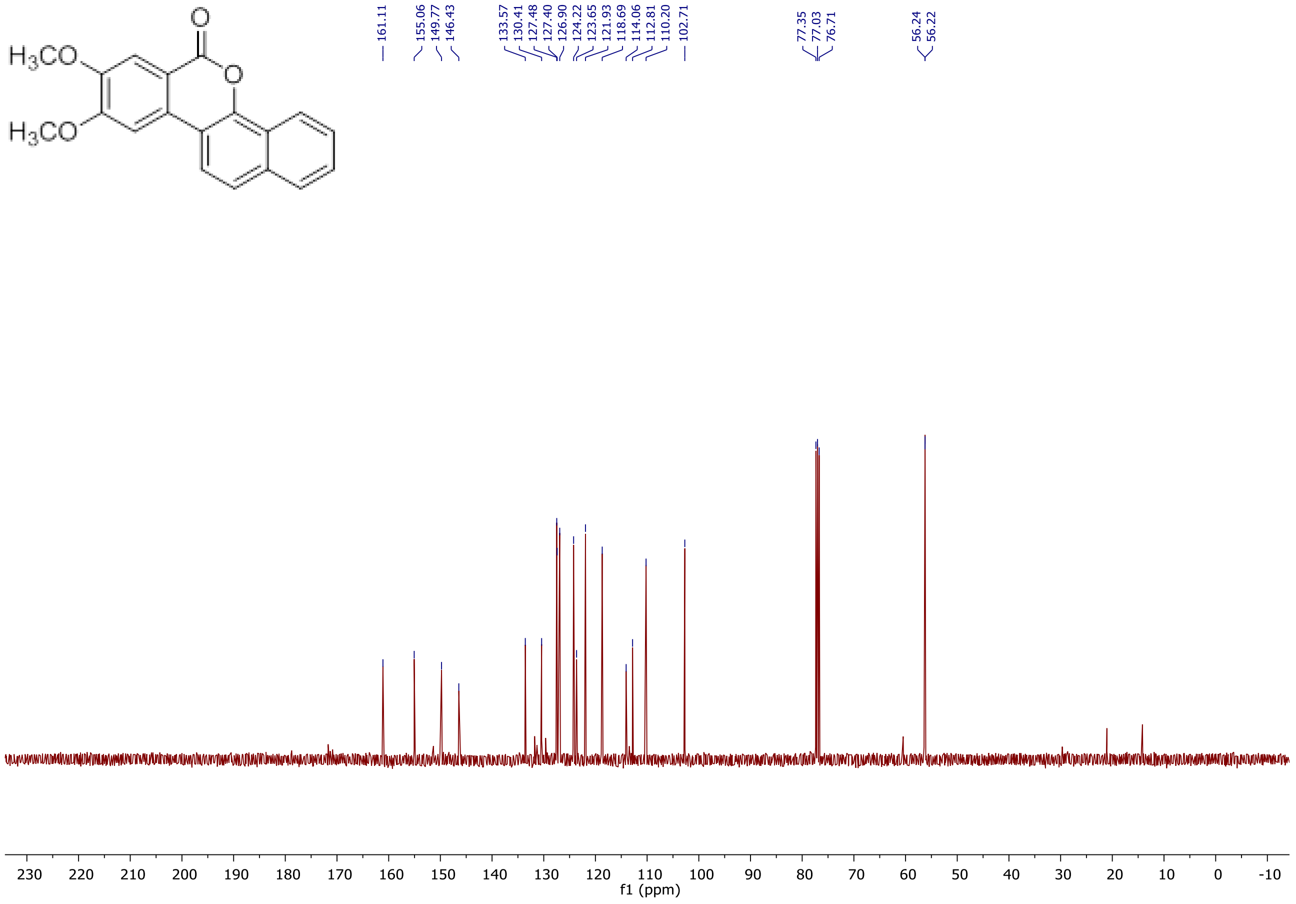

8,9-Dihydroxy-6H-dibenzo[c,h]chromen-6-one (**103**)

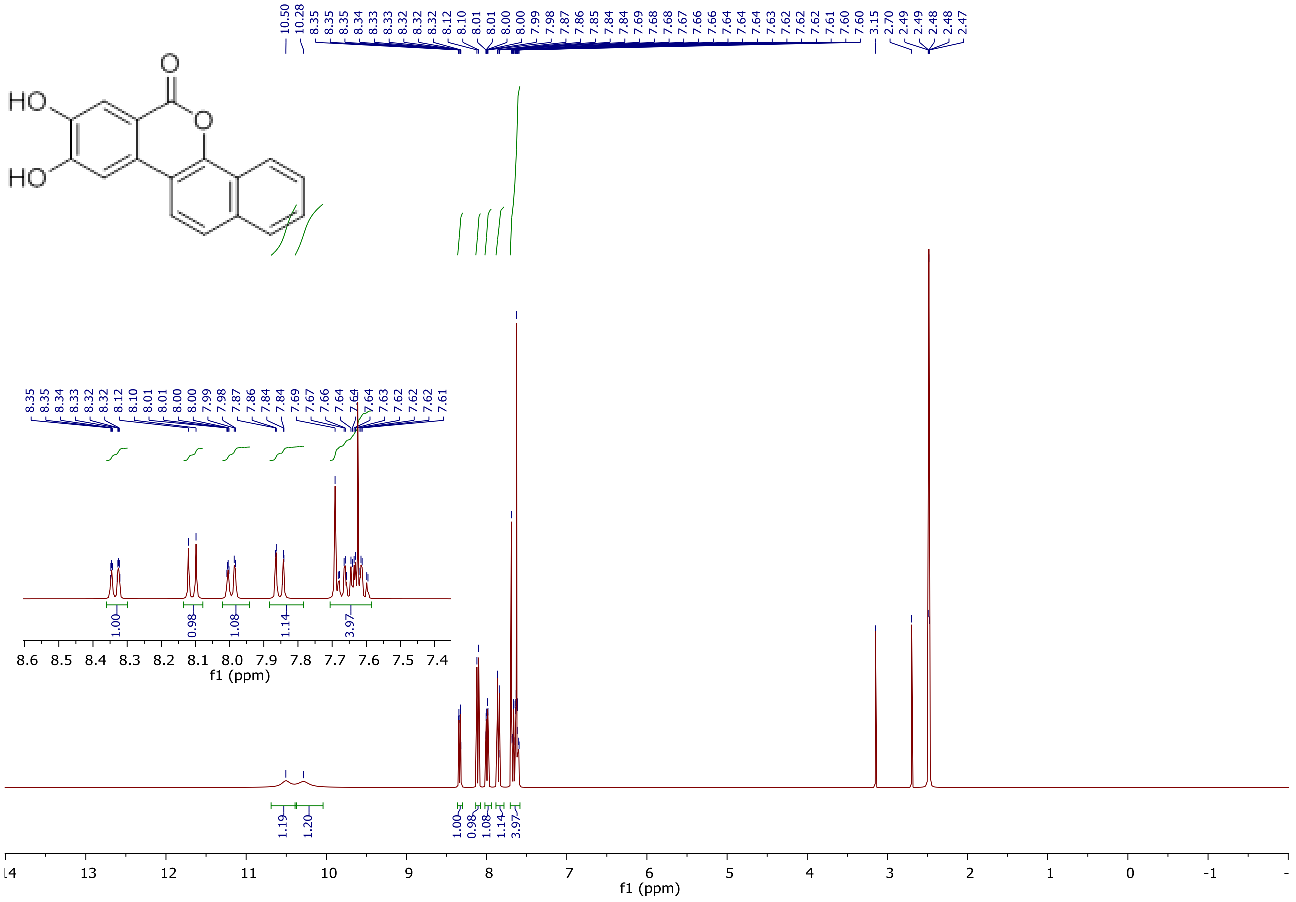

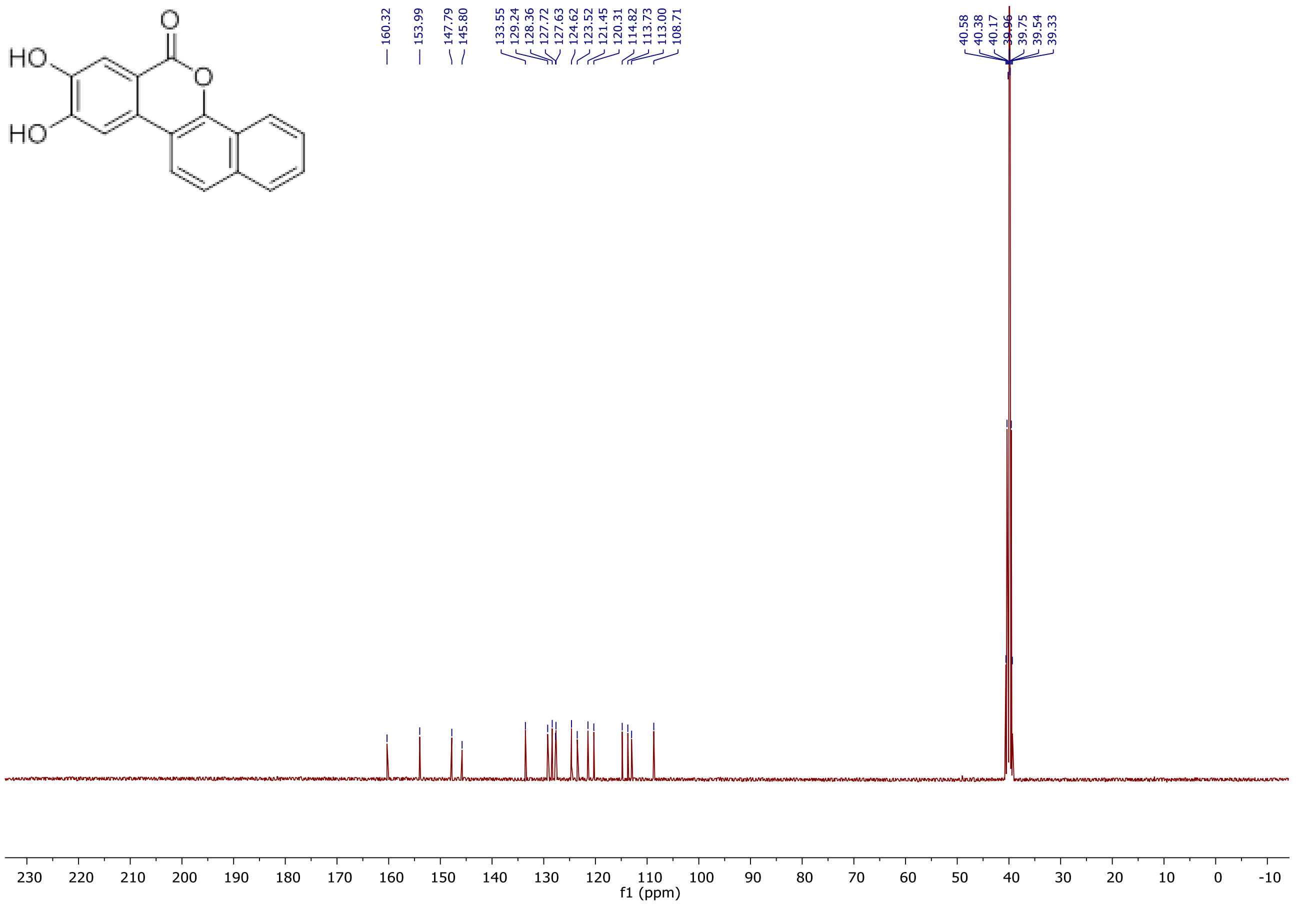

3-Hydroxy-8,9-dimethoxy-6H-benzo[c]chromen-6-one (**118**)

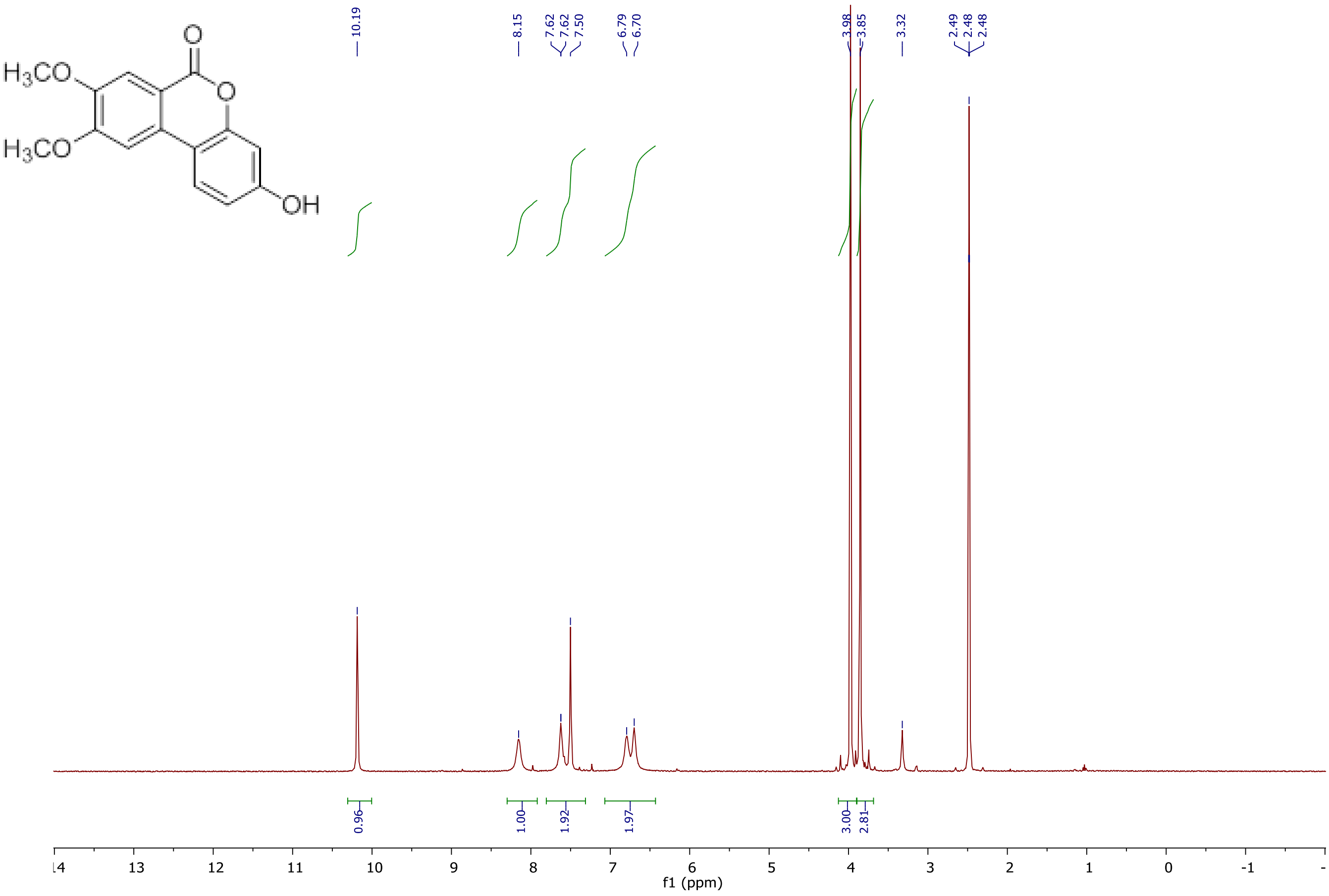

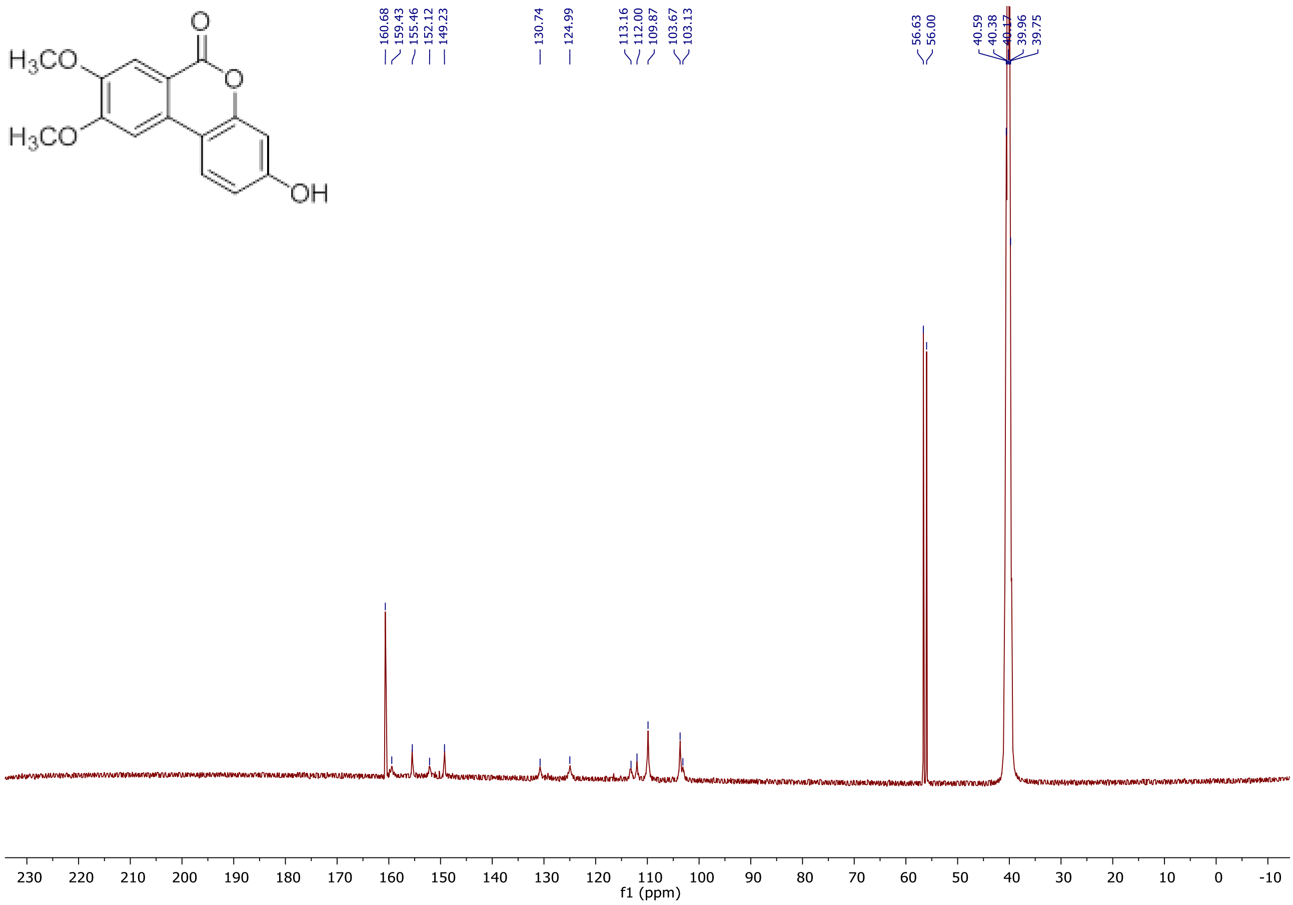

5-Bromo-4-formyl-2-methoxyphenyl acetate (**106**)

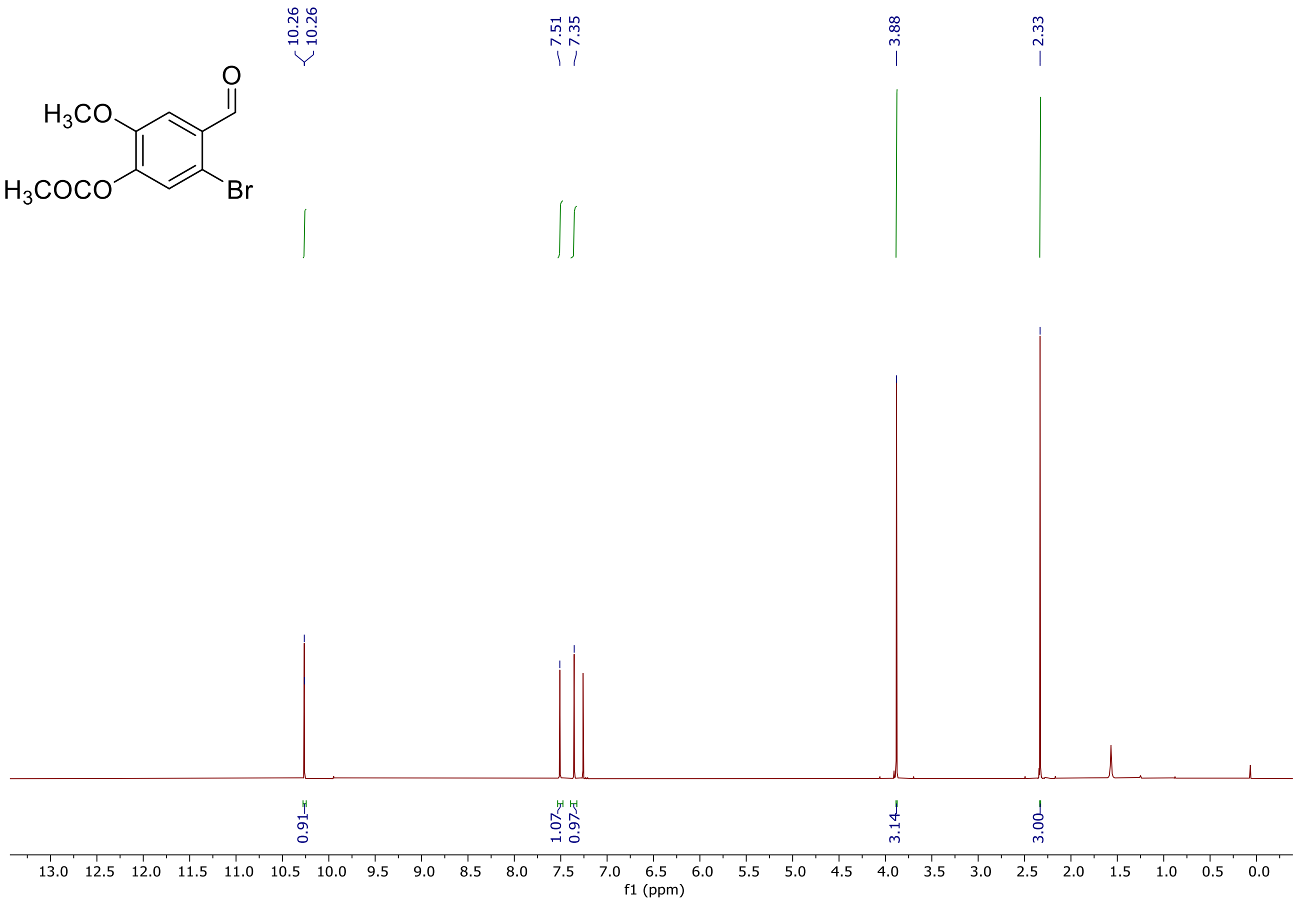

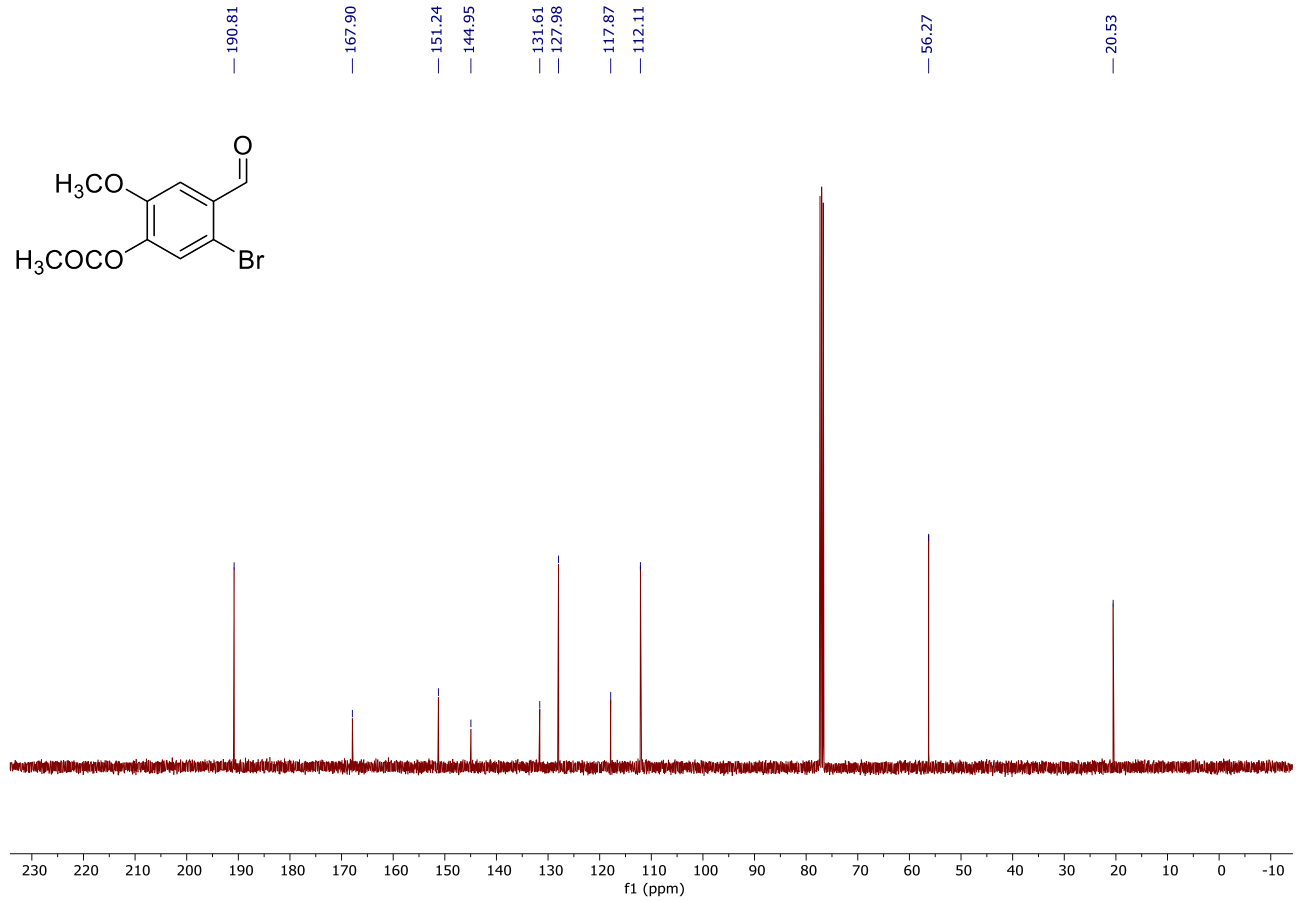

2-Bromo-4-hydroxy-5-methoxybenzoic acid (**113**)

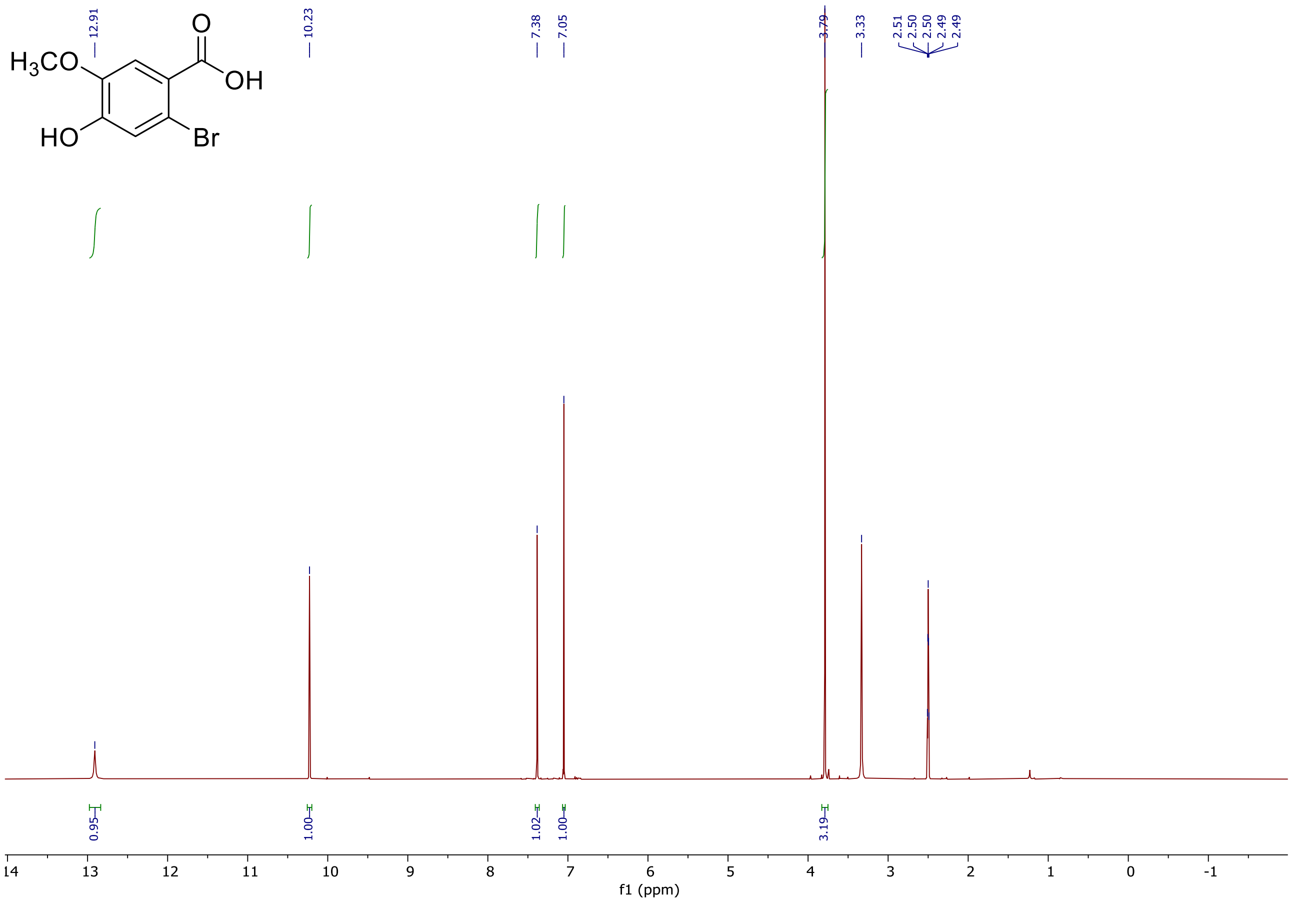

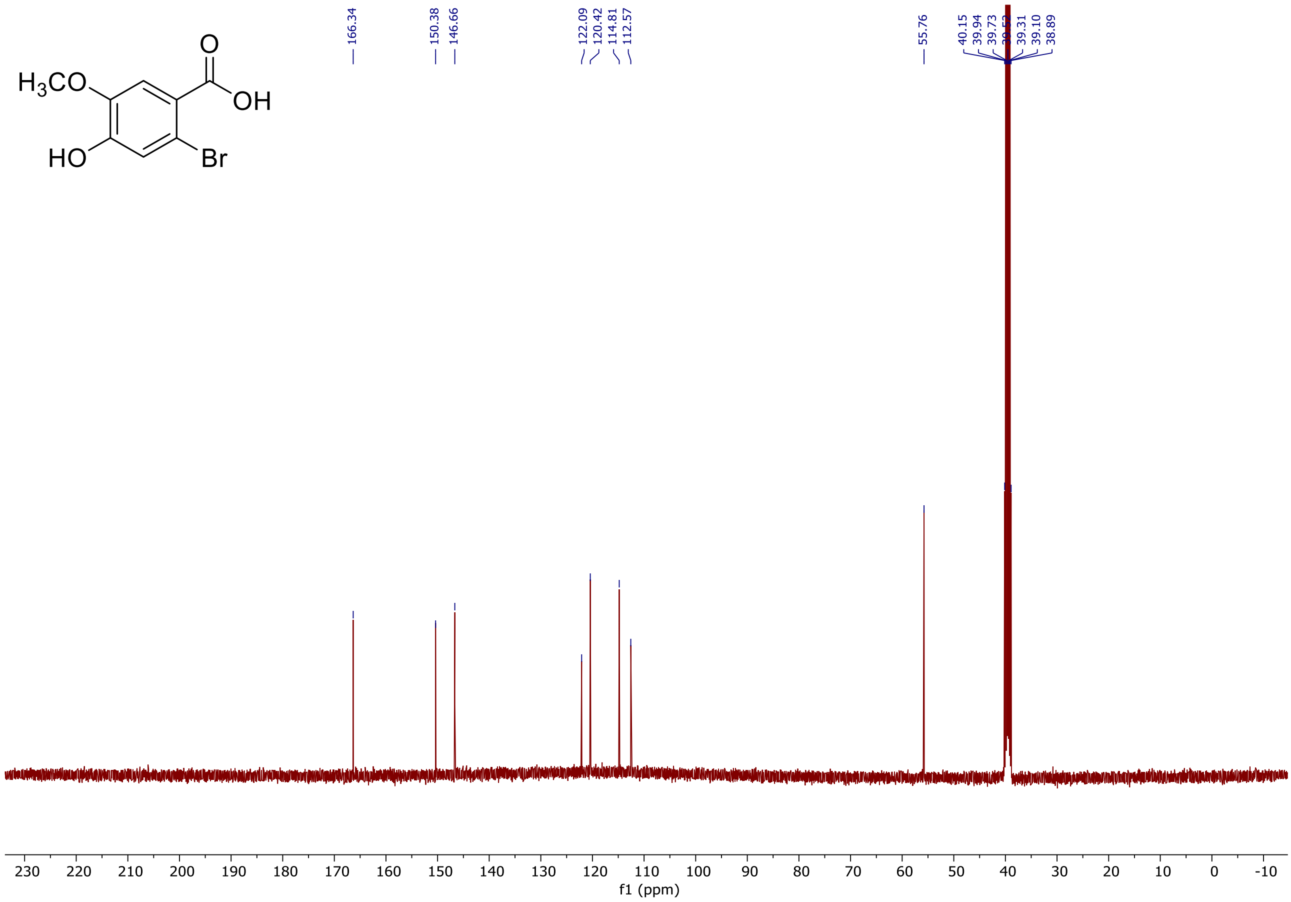

3,9-Dihydroxy-8-methoxy-6H-benzo[c]chromen-6-one (**119**)

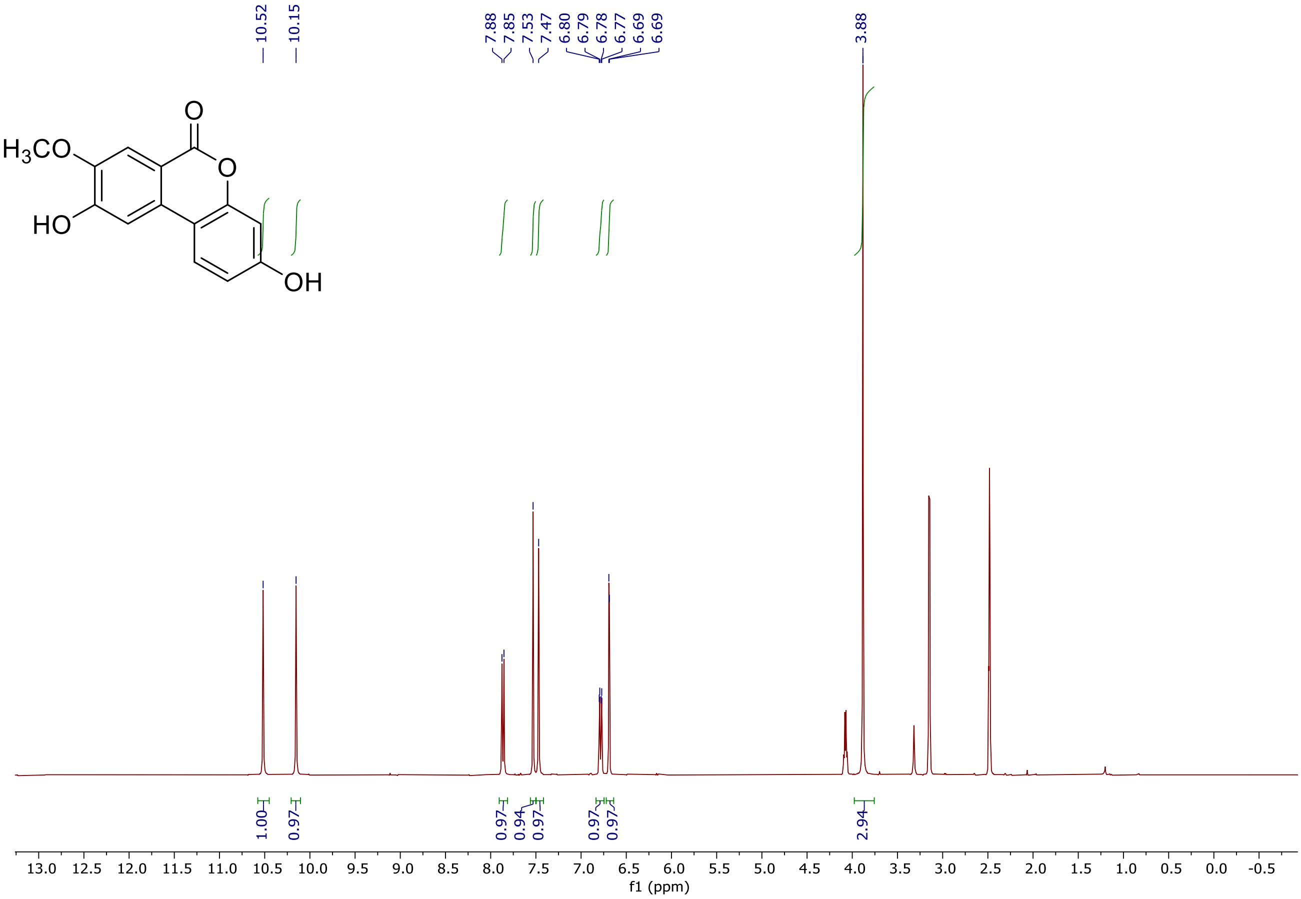

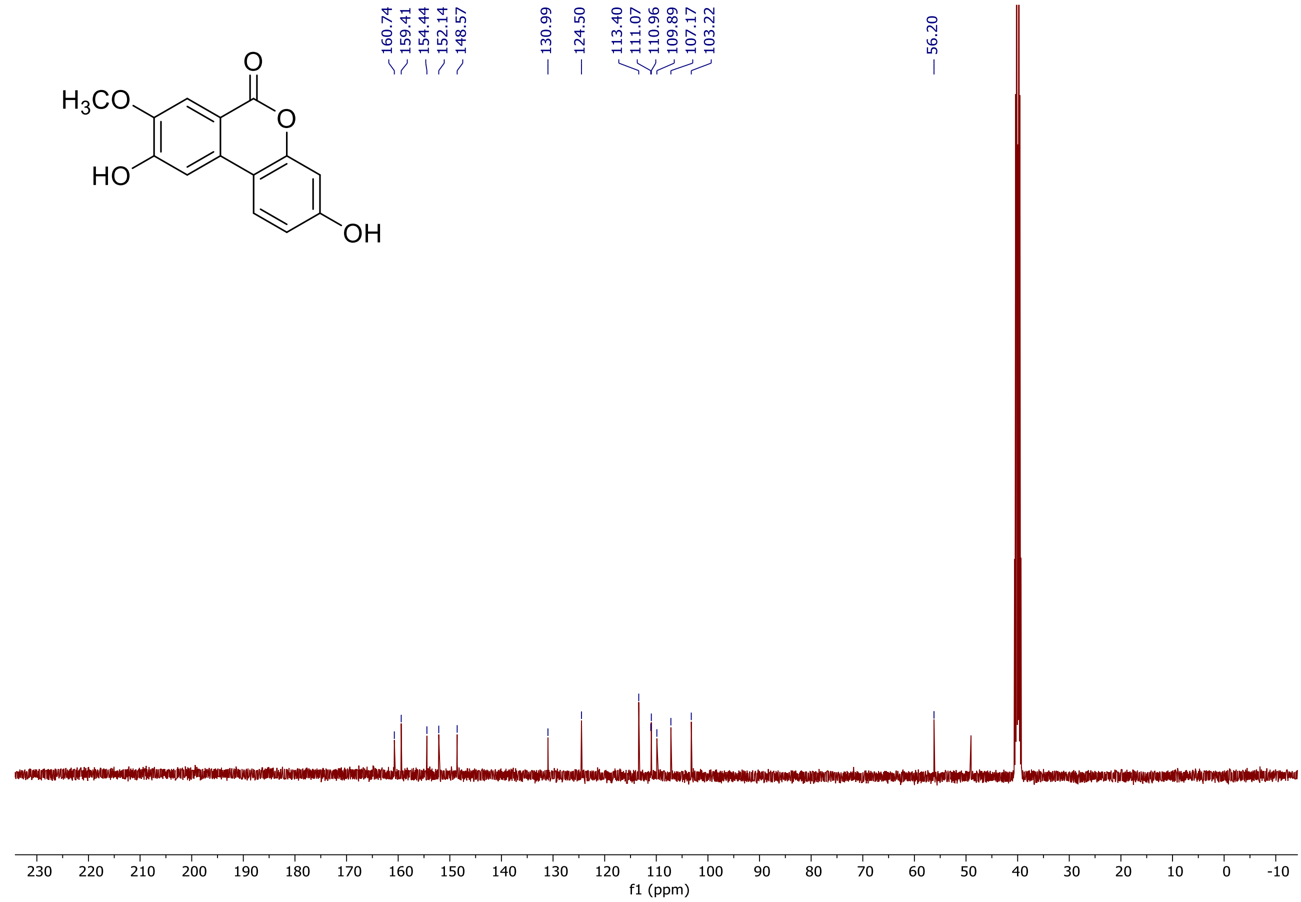

4-Bromo-5-formyl-2-methoxyphenyl acetate (**107**)

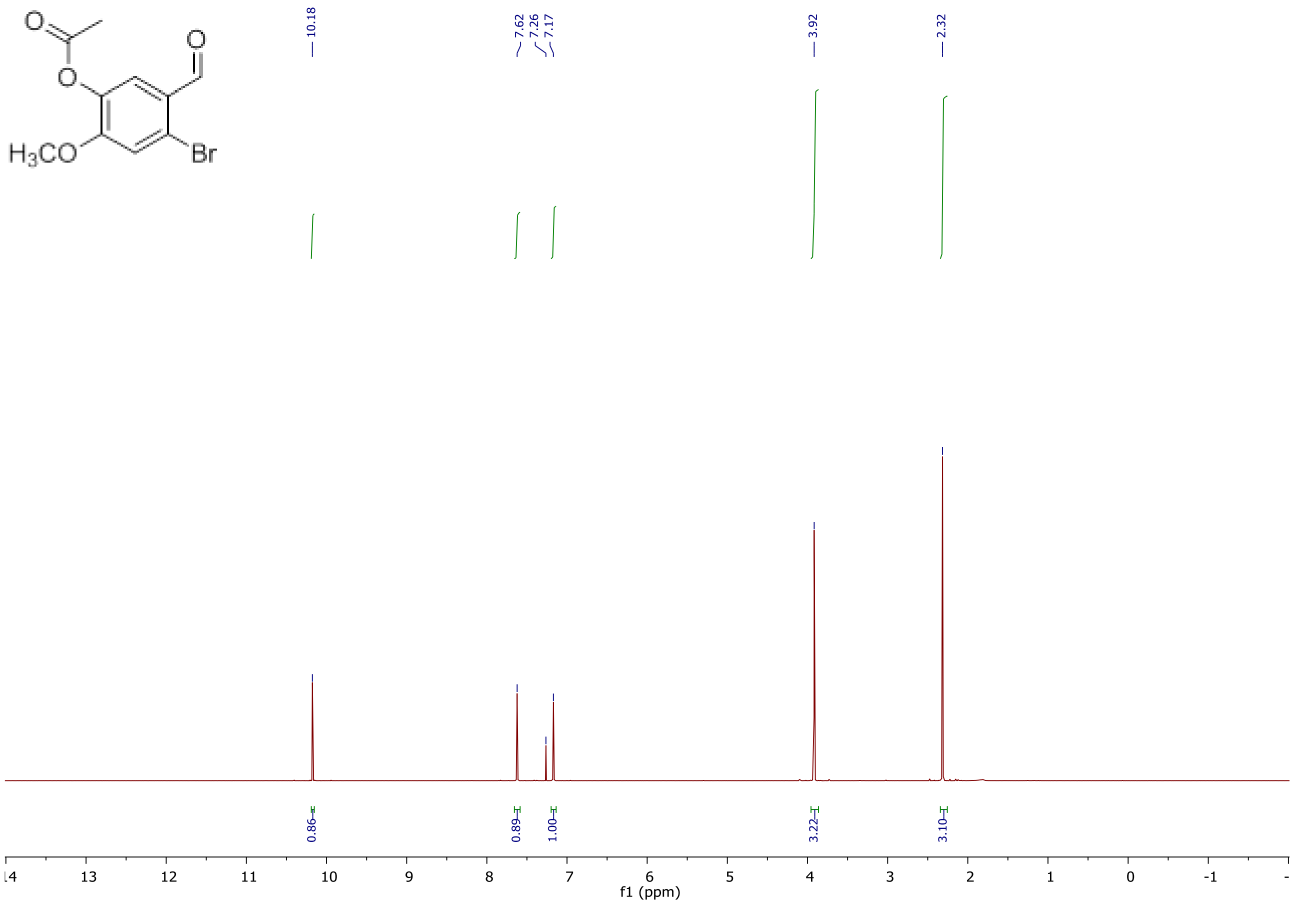

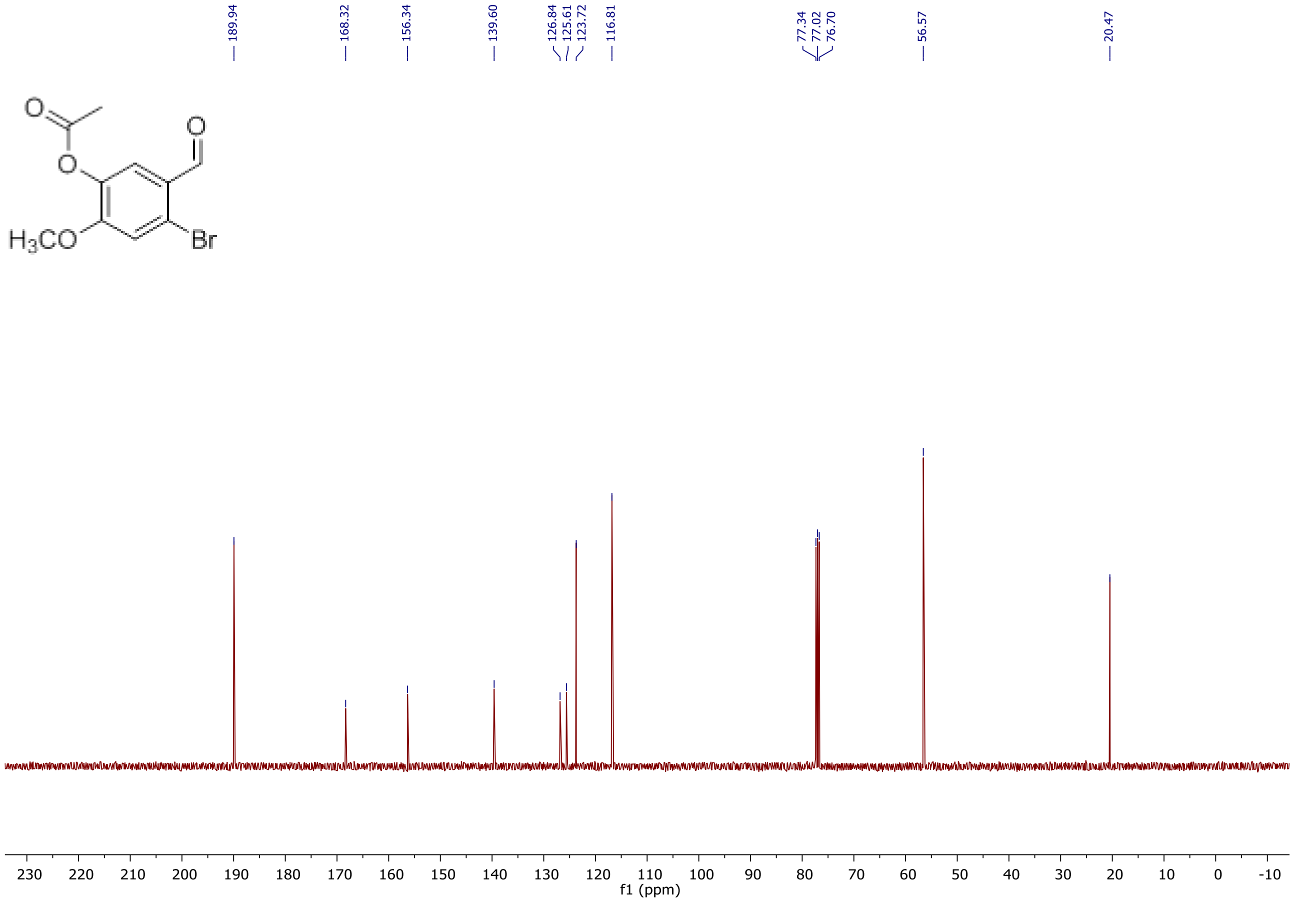

5-Acetoxy-2-bromo-4-methoxybenzoic acid (**114**)

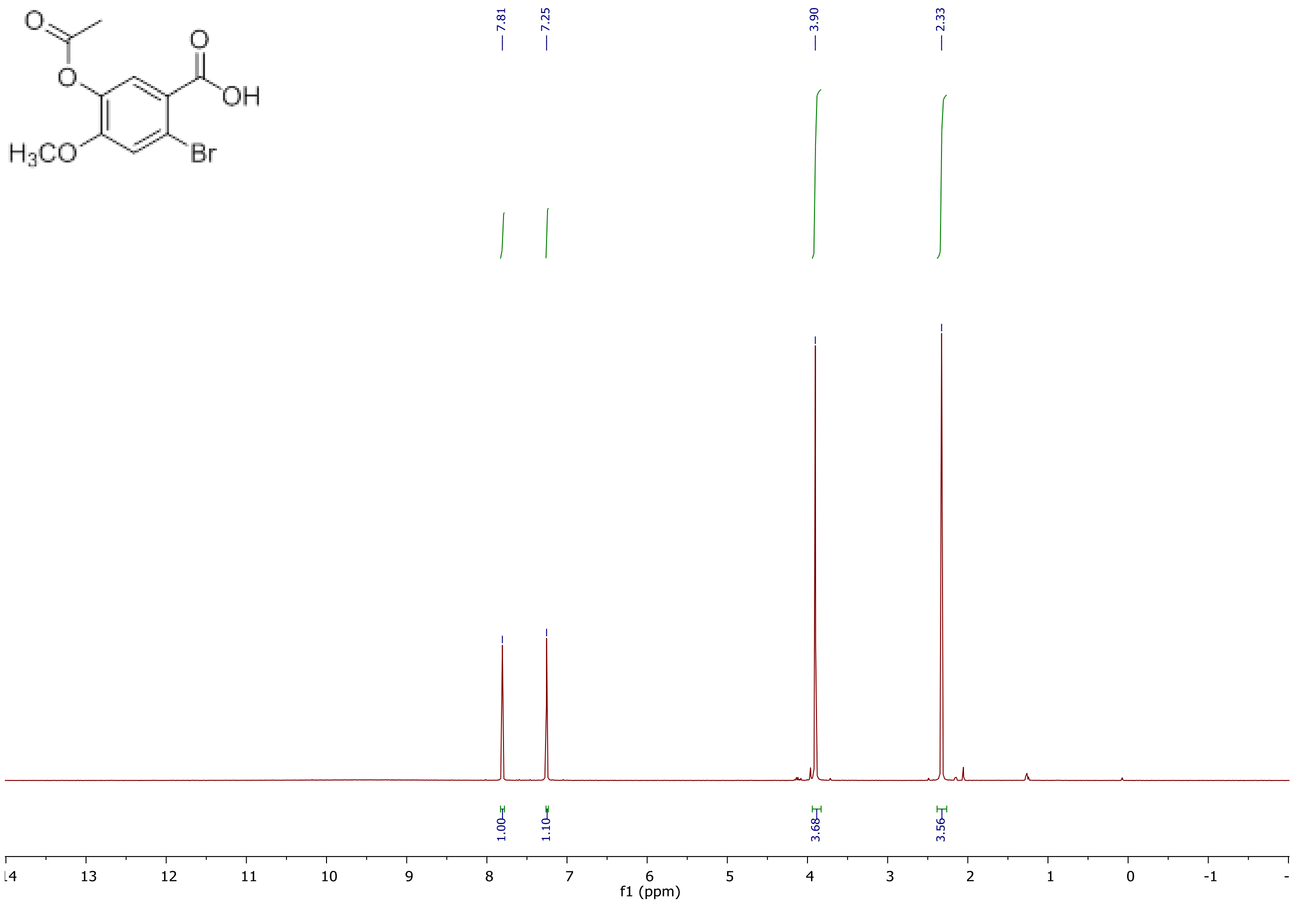

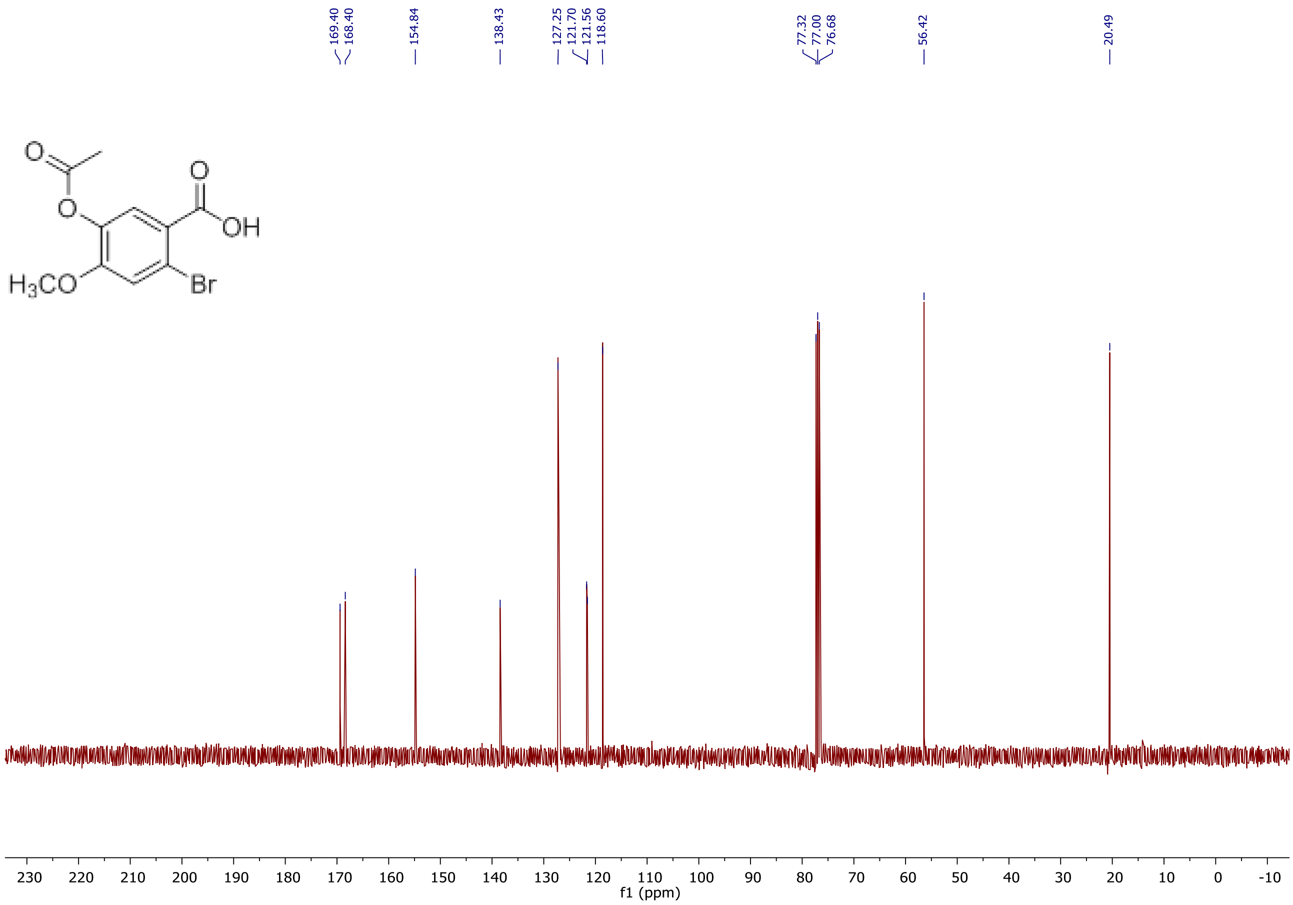

2-Bromo-5-hydroxy-4-methoxybenzoic acid (**115**)

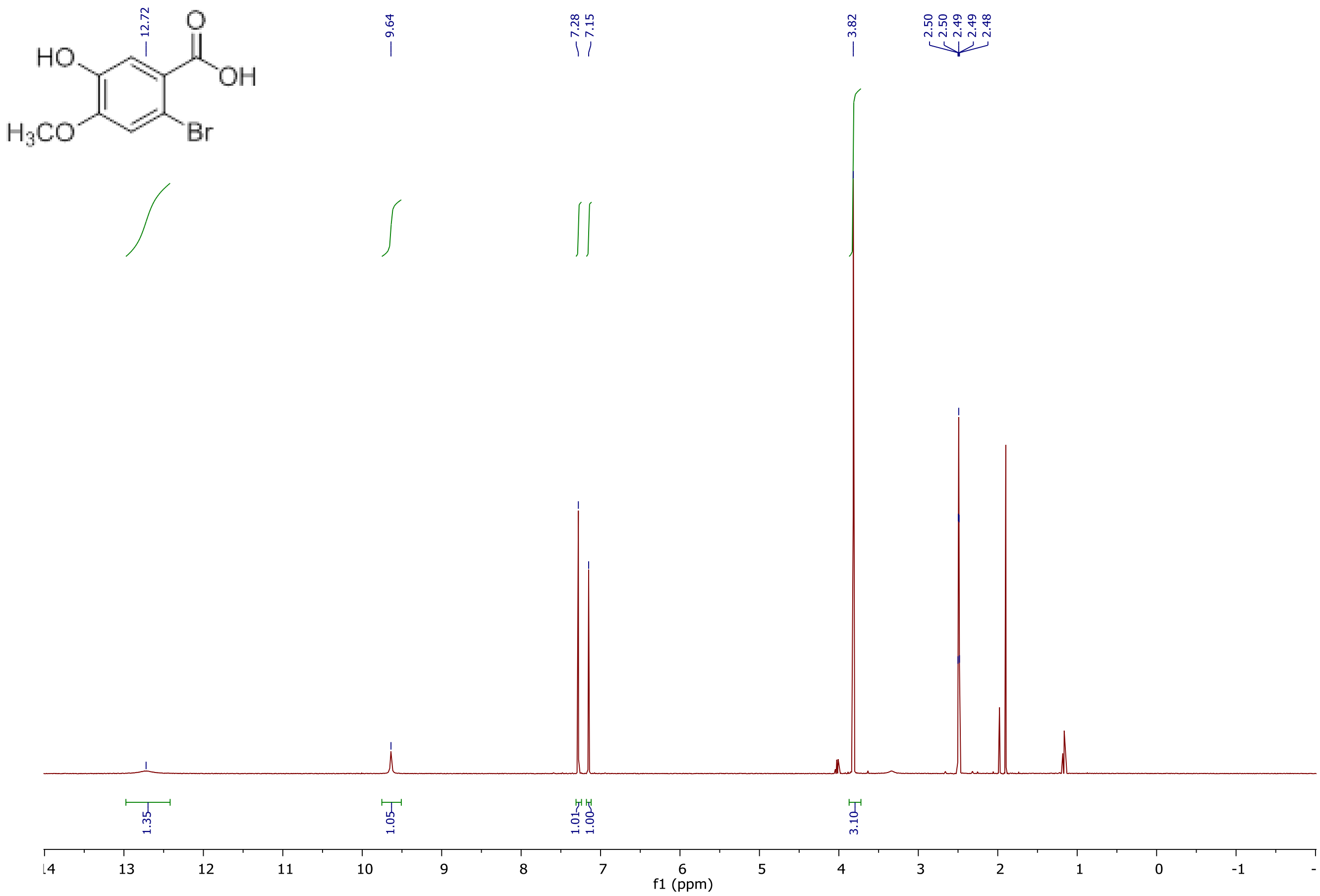

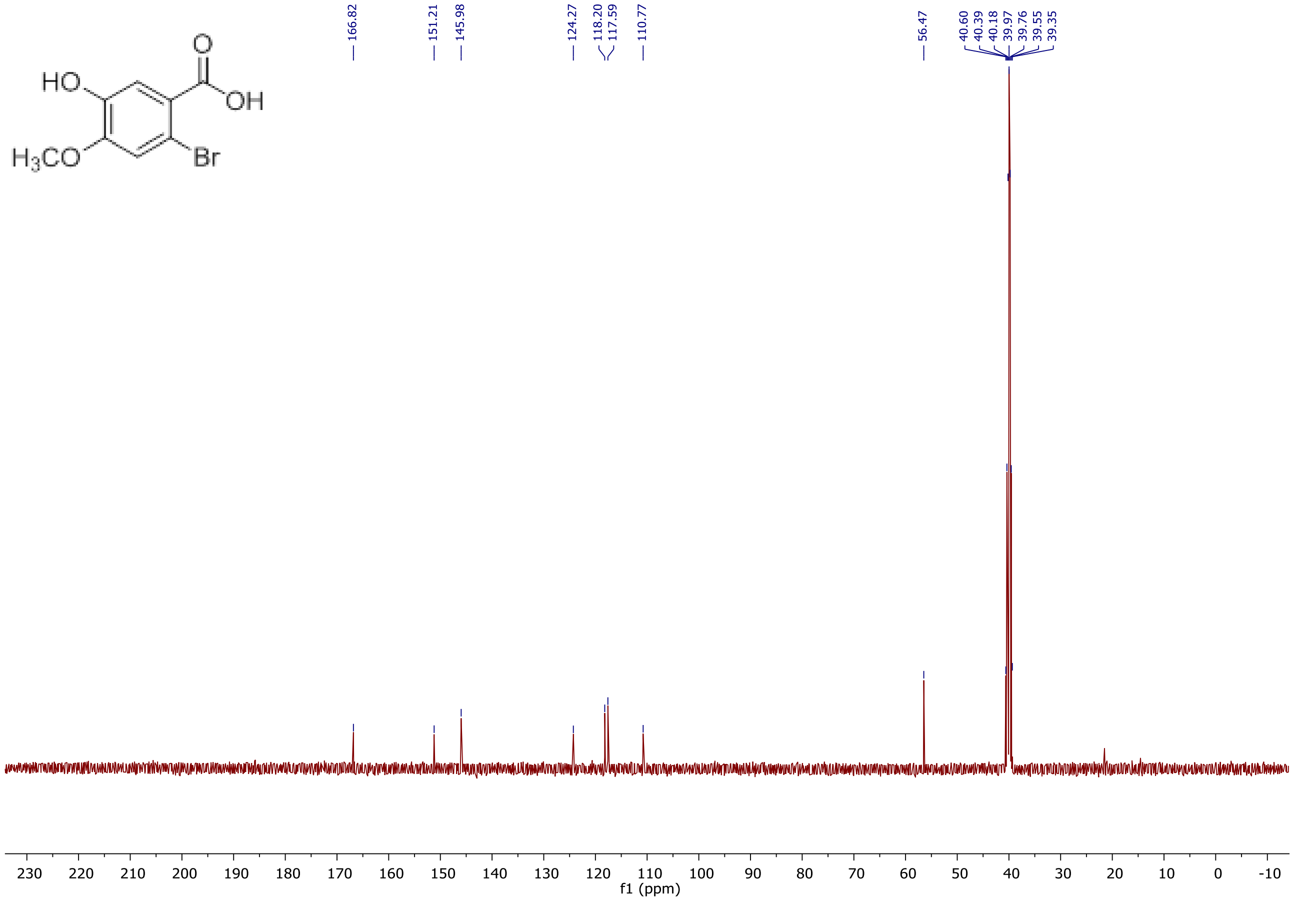

3,8-Dihydroxy-9-methoxy-6H-benzo[c]chromen-6-one (**120**)

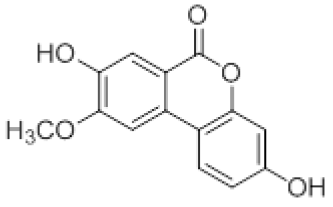

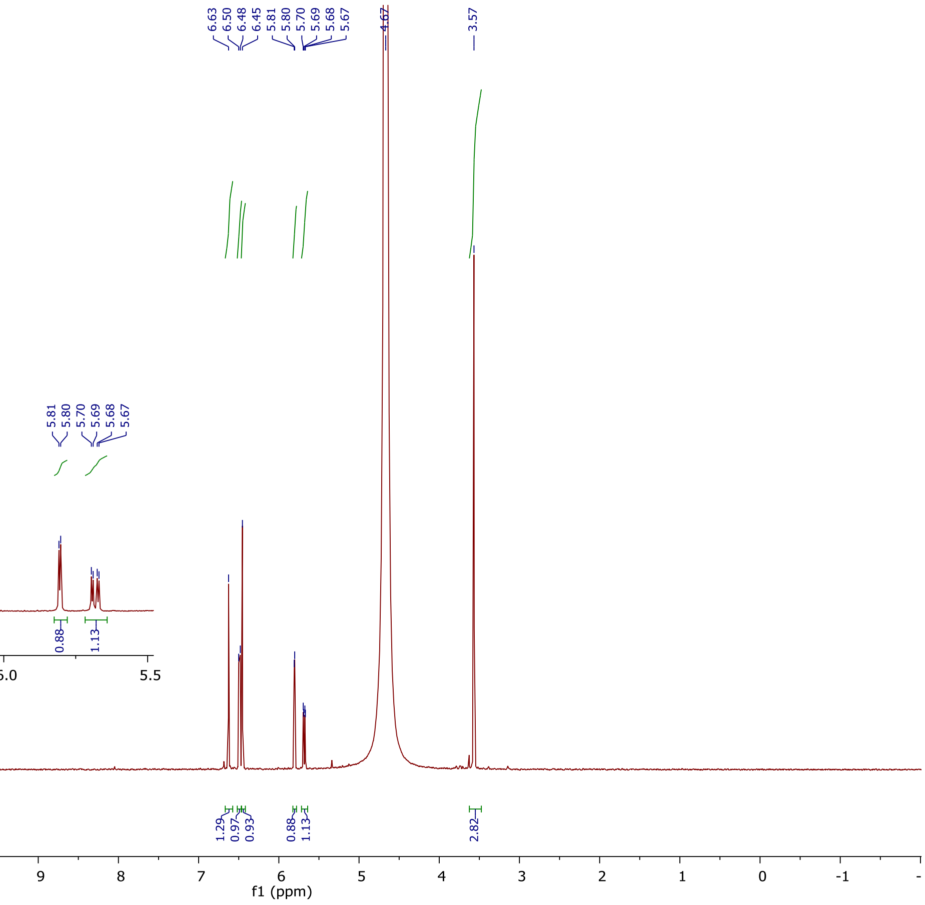

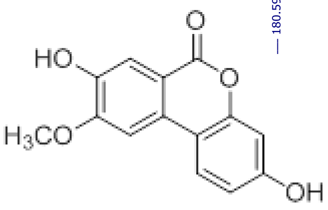

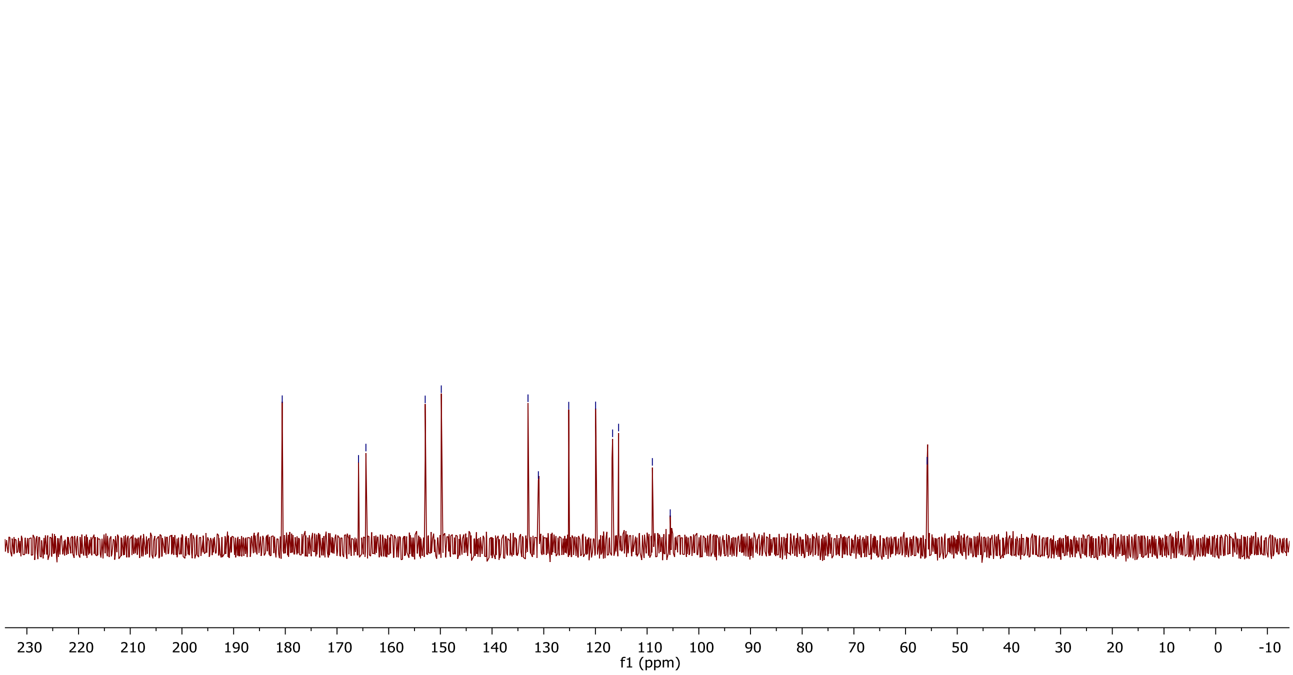

2-Bromo-5-methoxy-4-nitrobenzoic acid (**116**)

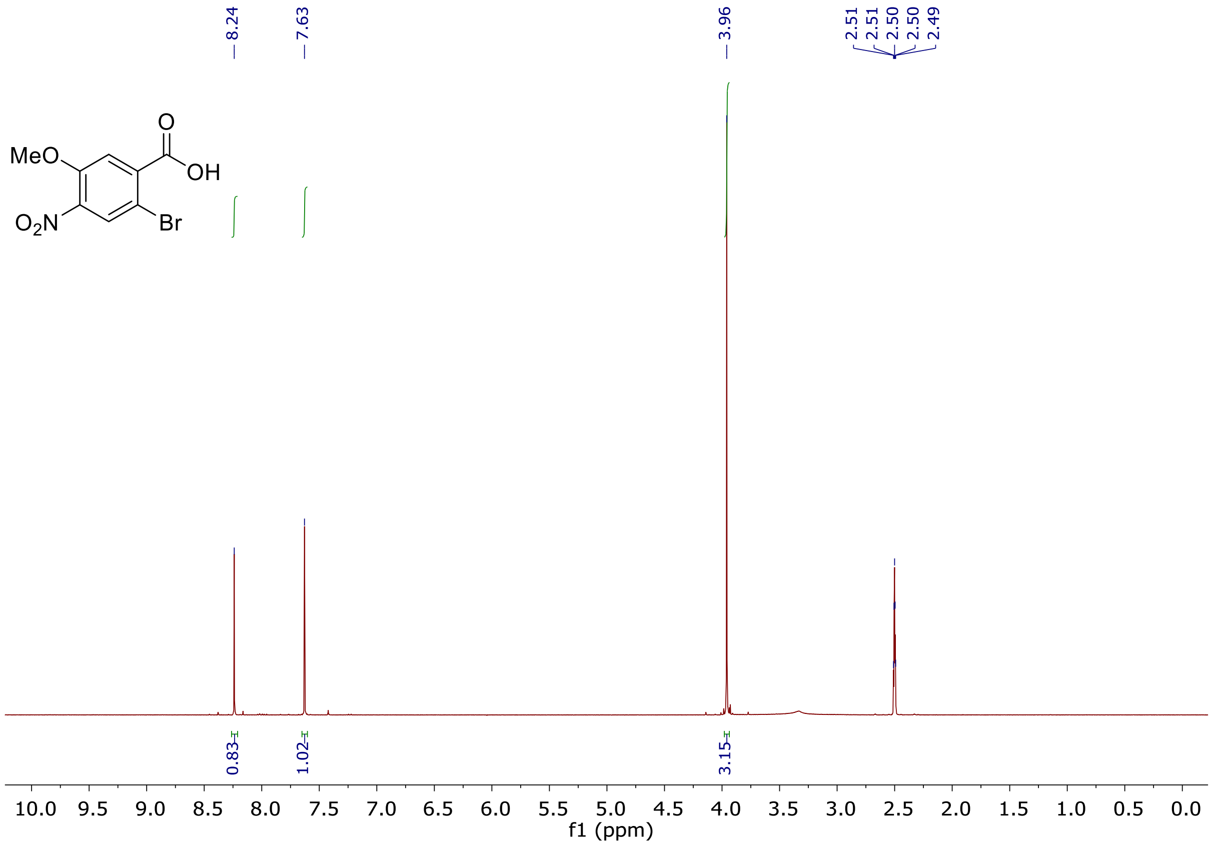

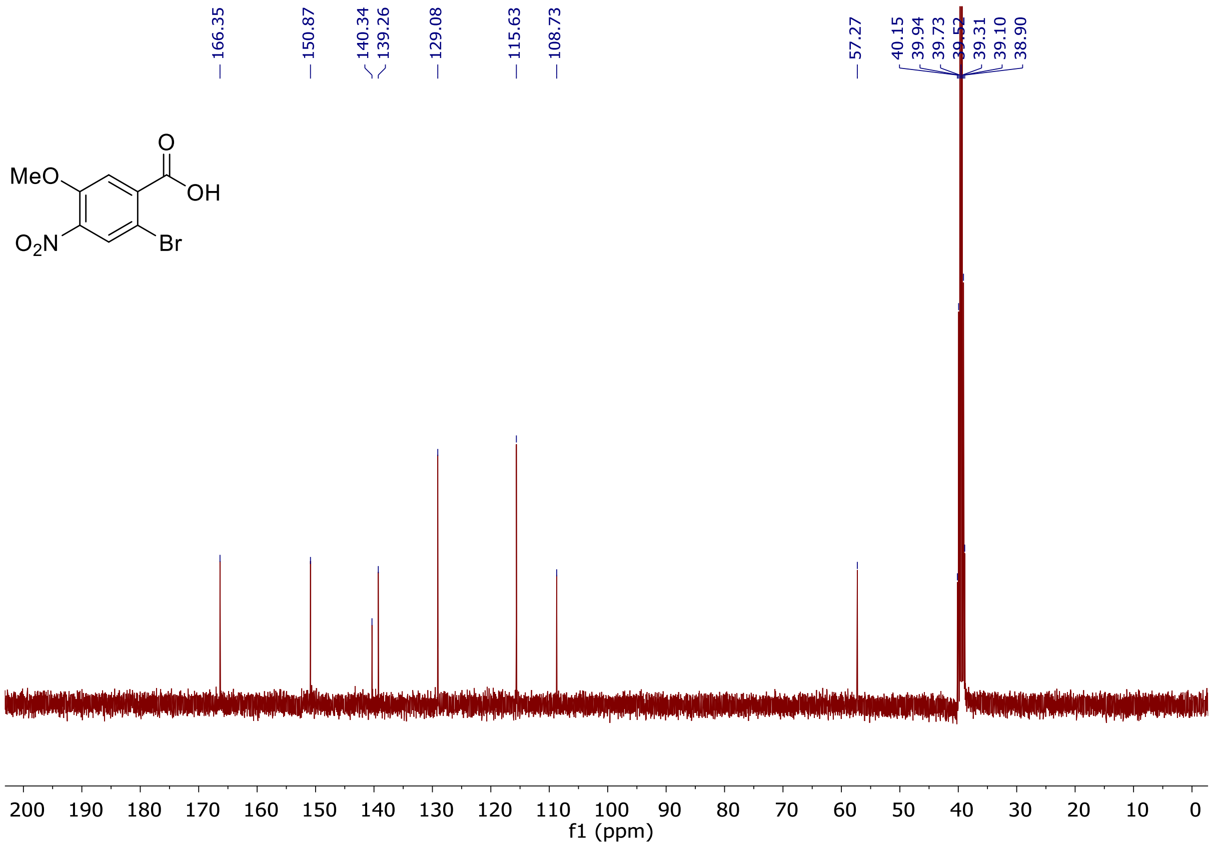

3-Hydroxy-8-methoxy-9-nitro-6H-benzo[c]chromen-6-one (**121**)

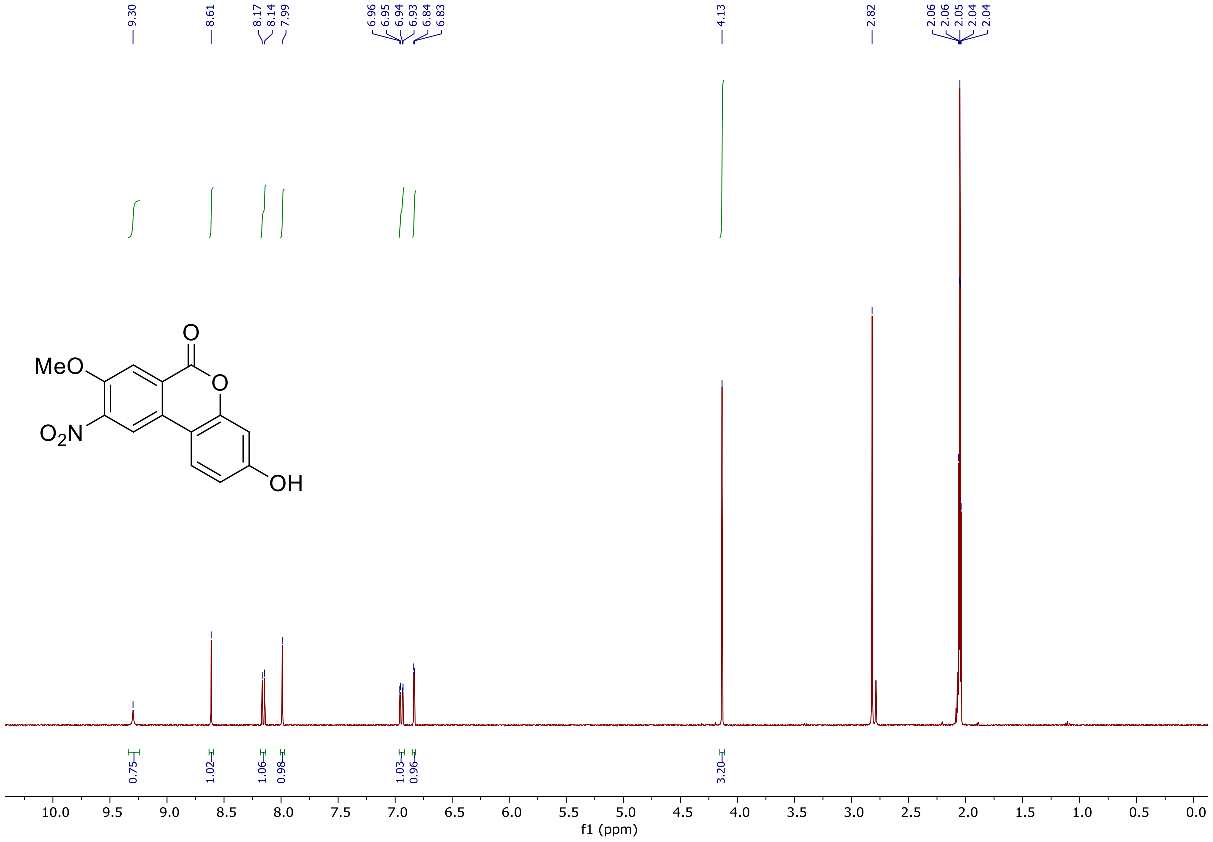

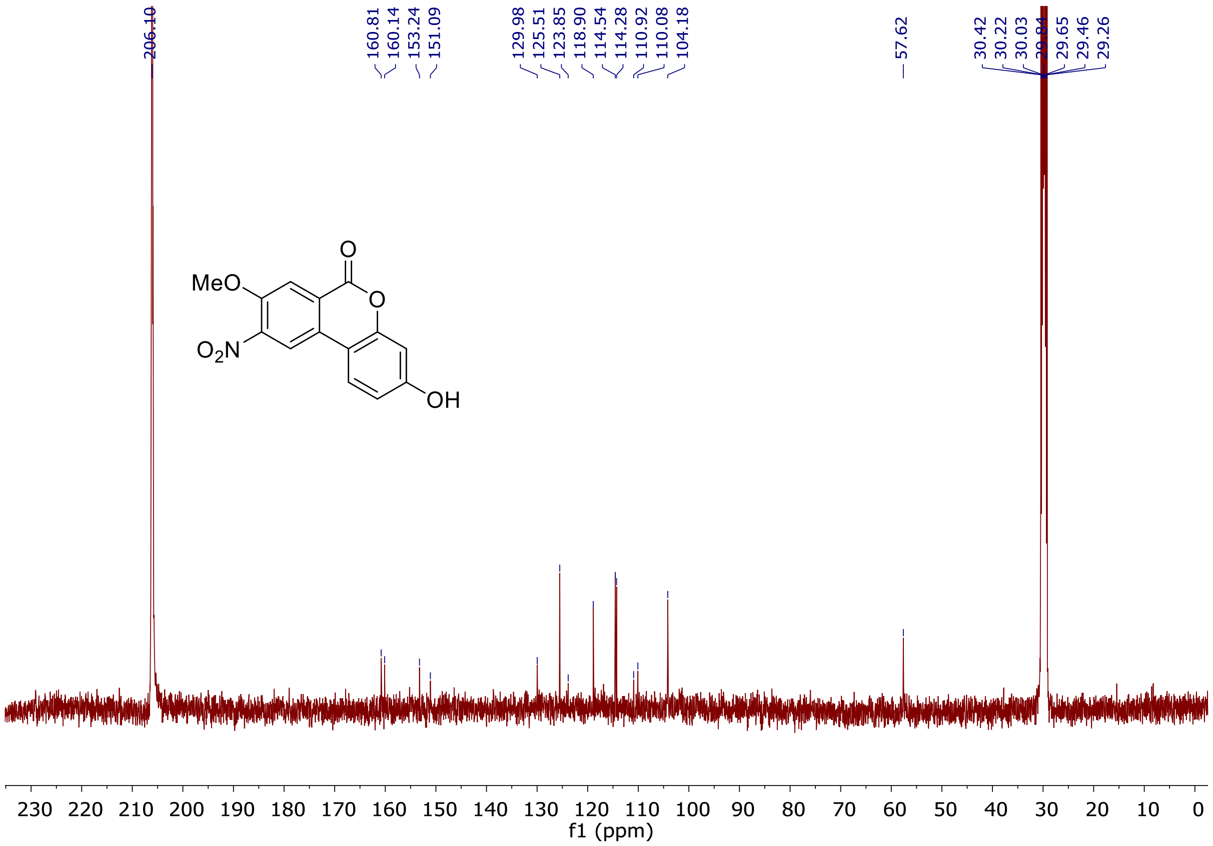

9-Amino-3-hydroxy-8-methoxy-6H-benzo[c]chromen-6-one (**123**)

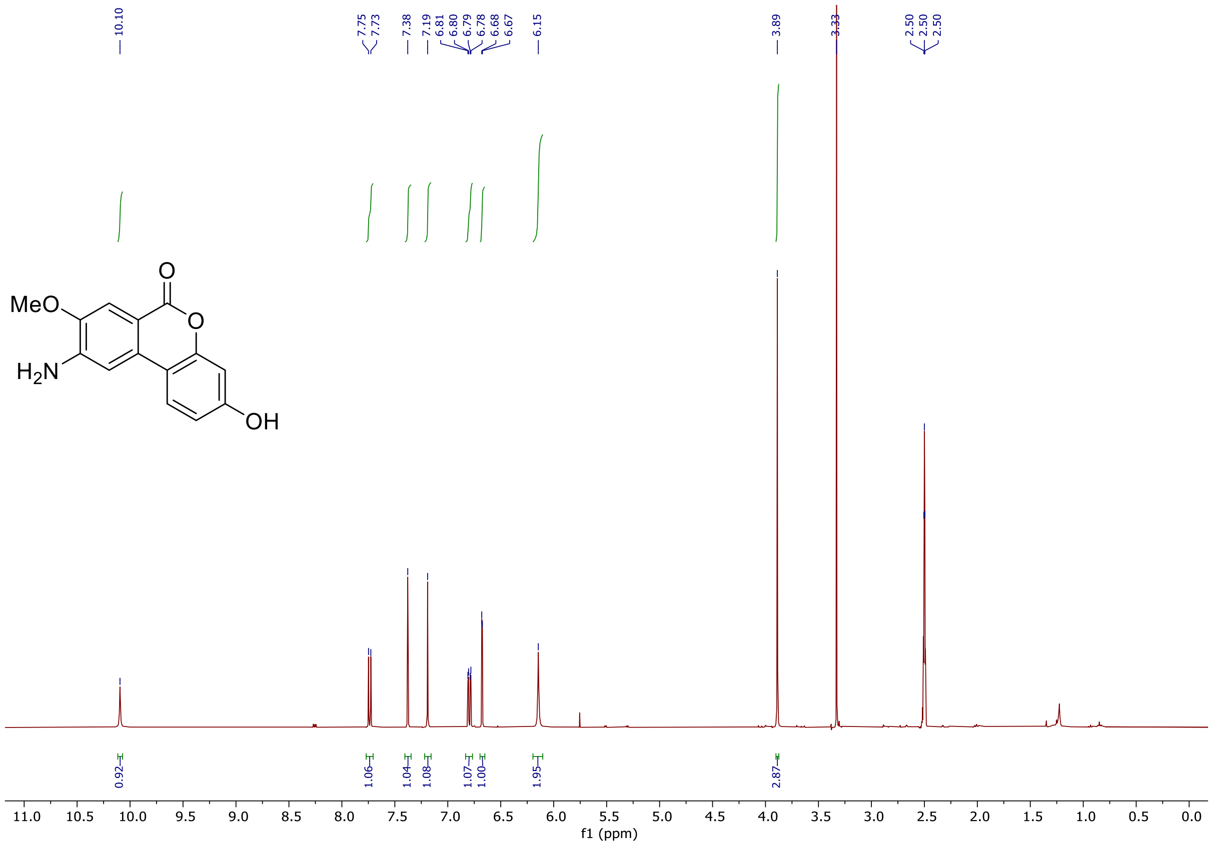

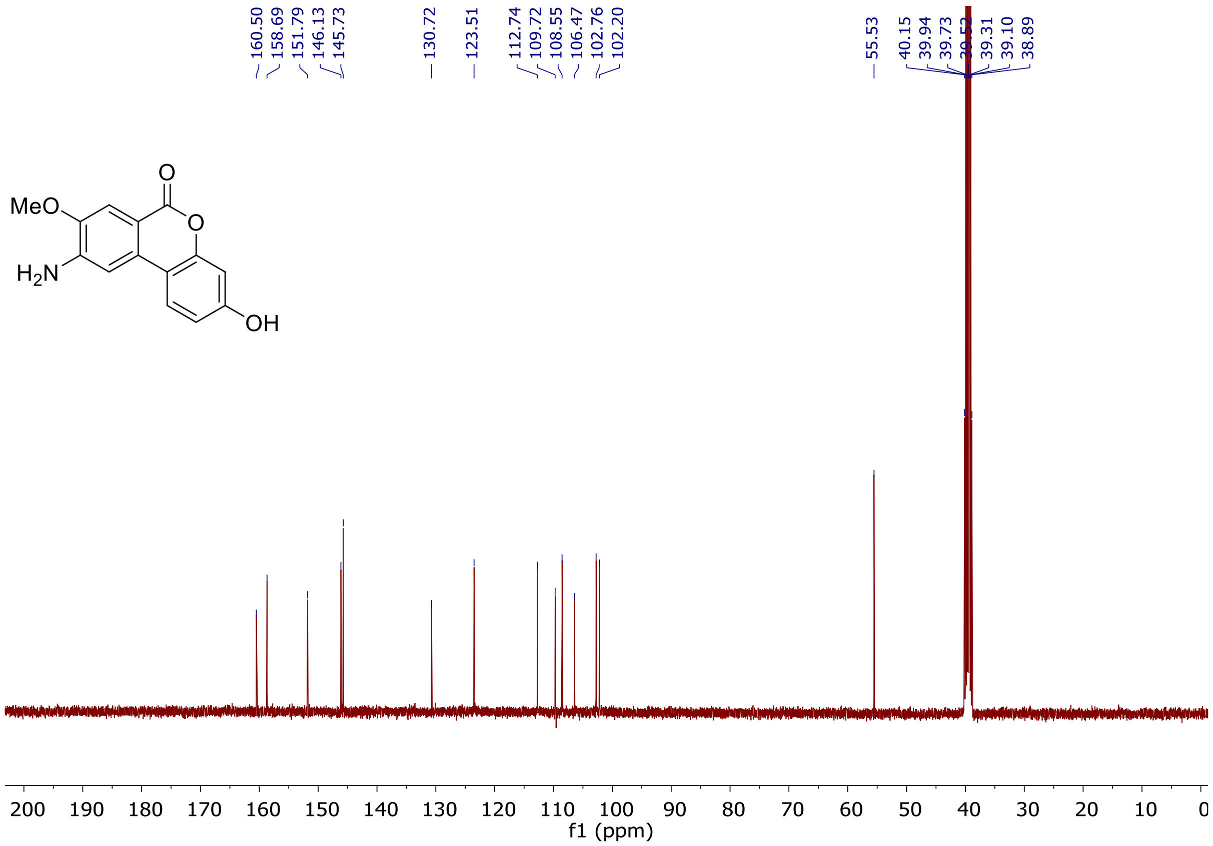

9-Amino-3,8-dihydroxy-6H-benzo[c]chromen-6-one (**124**)

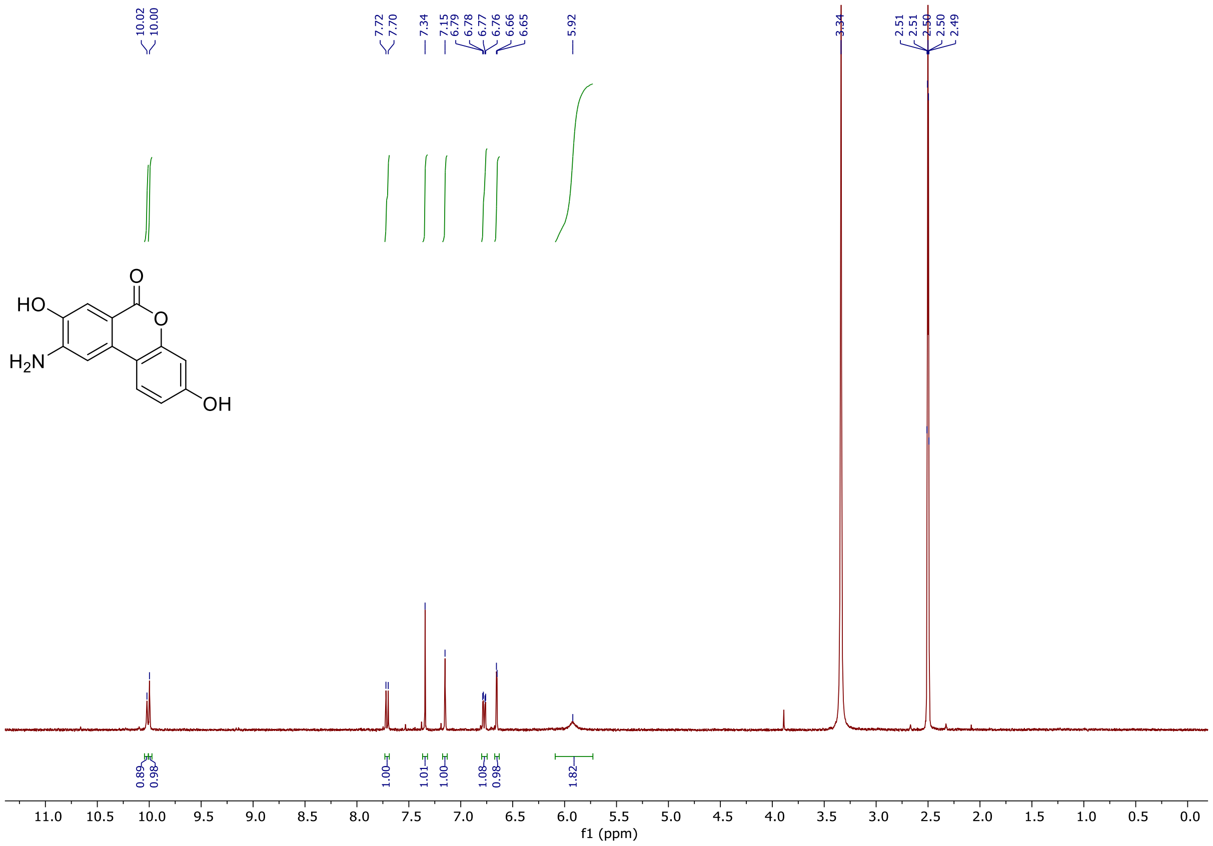

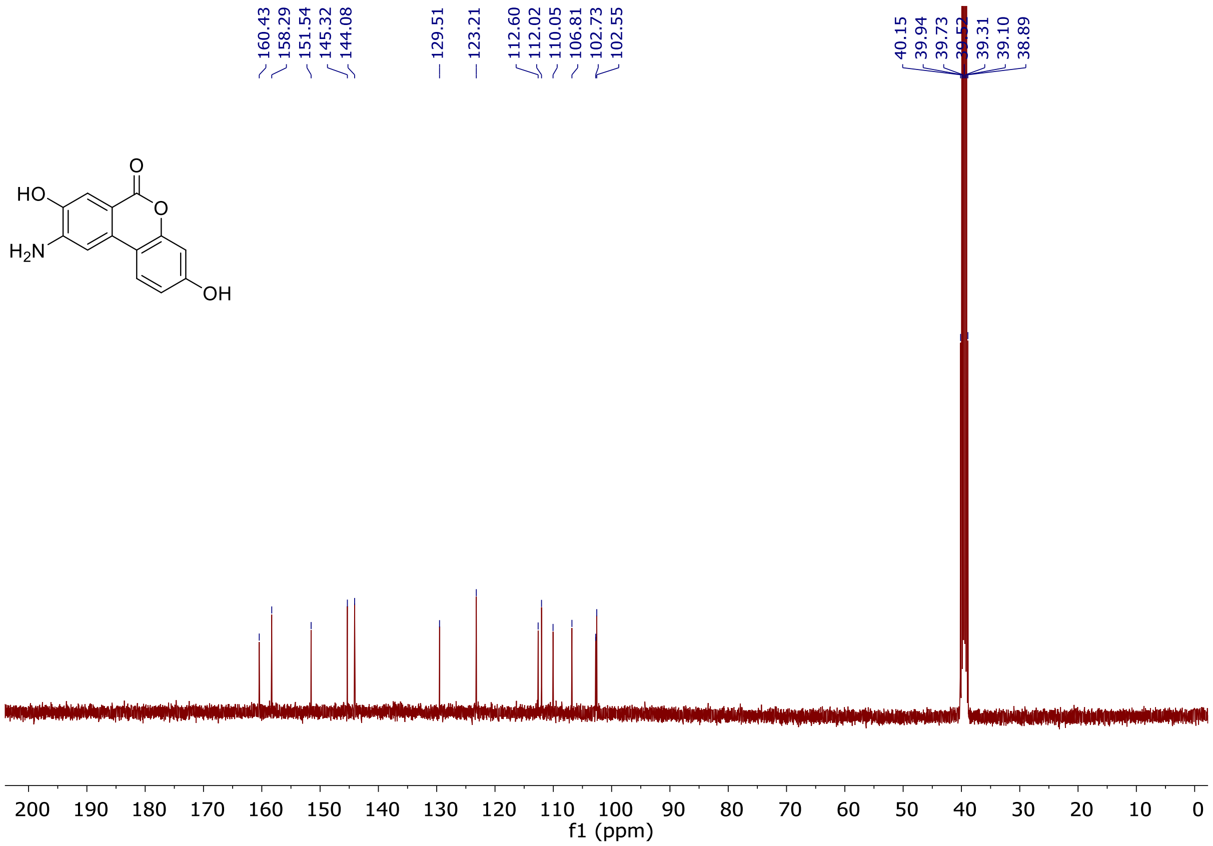

N-(3,8-Dihydroxy-6-oxo-6H-benzo[c]chromen-9-yl)acetamide (**127**)

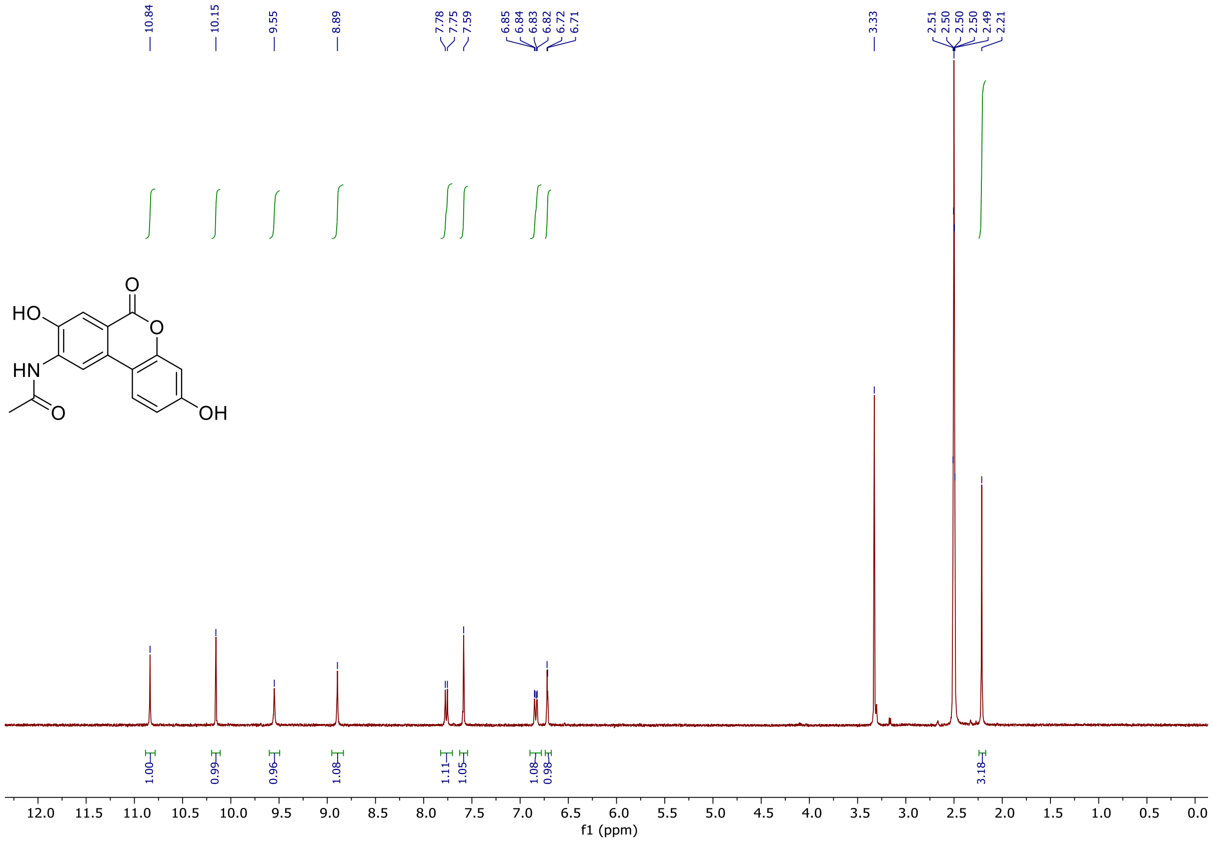

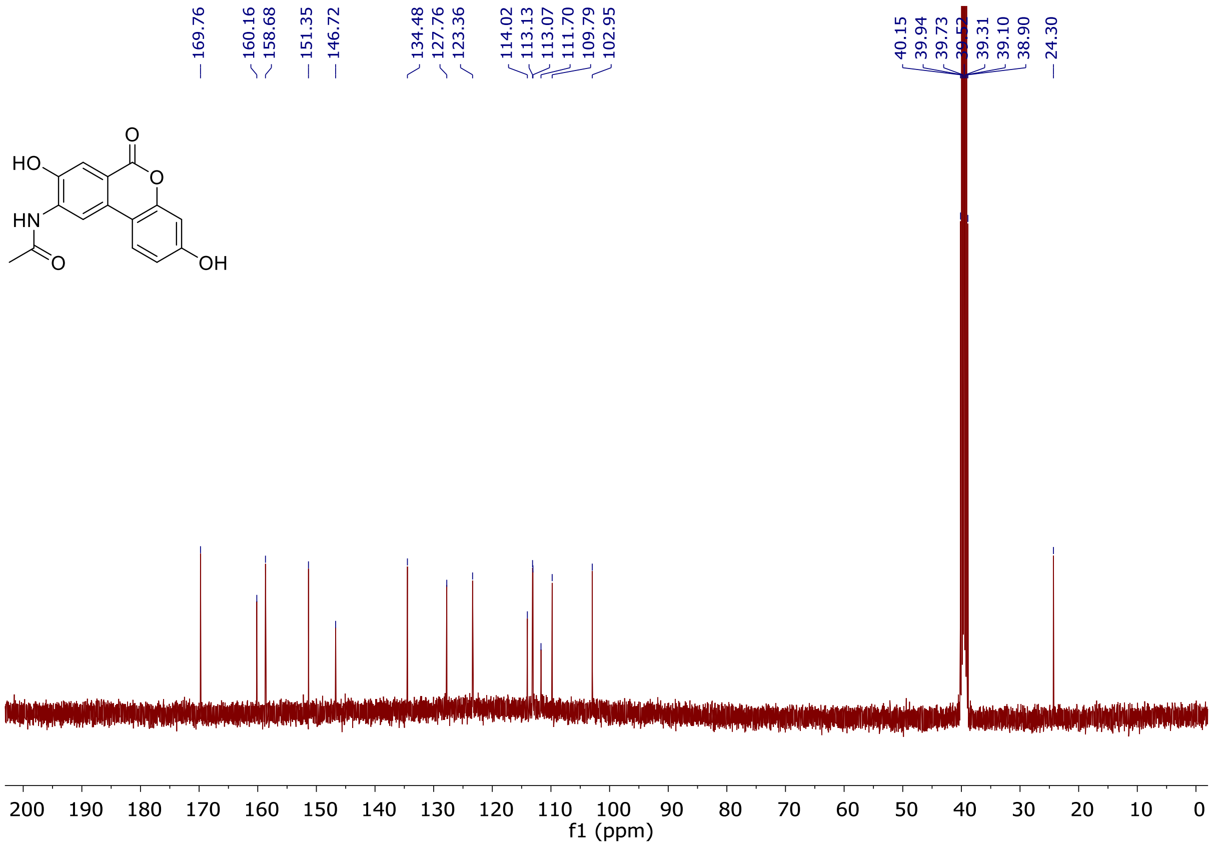

Methyl 2-bromo-4,5-dinitrobenzoate (**110**)

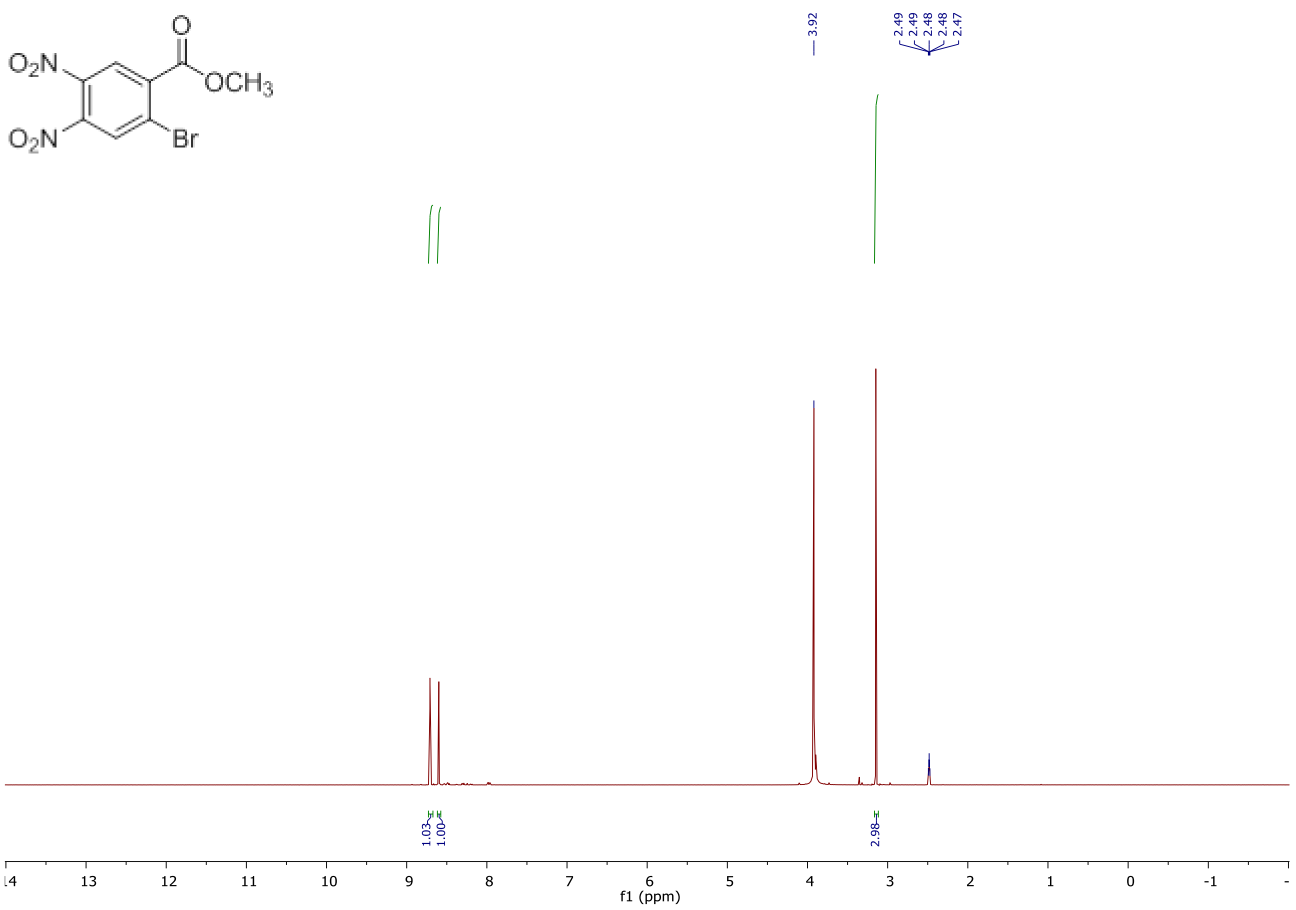

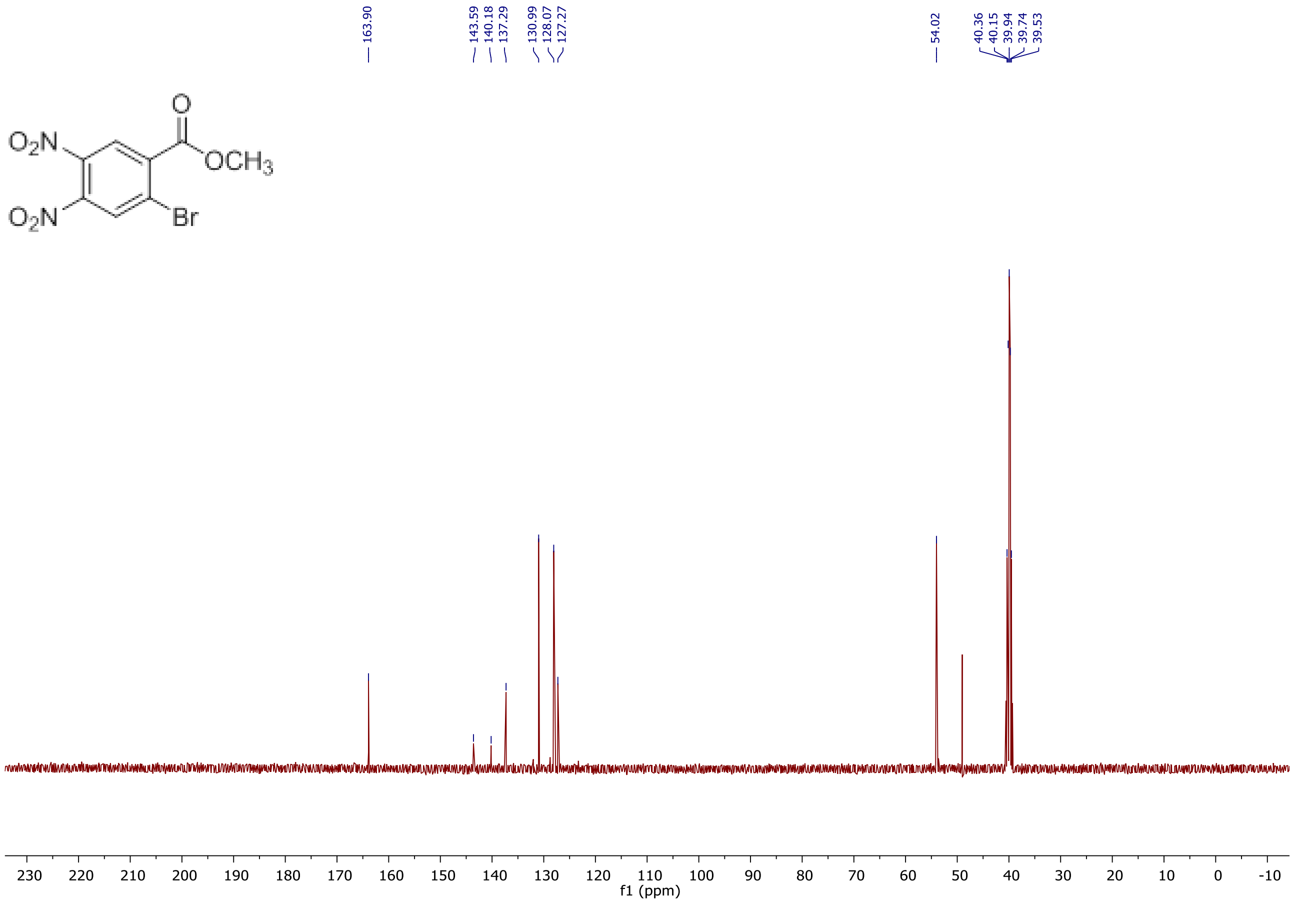

Methyl 2-bromo-4-methoxy-5-nitrobenzoate (**111**)

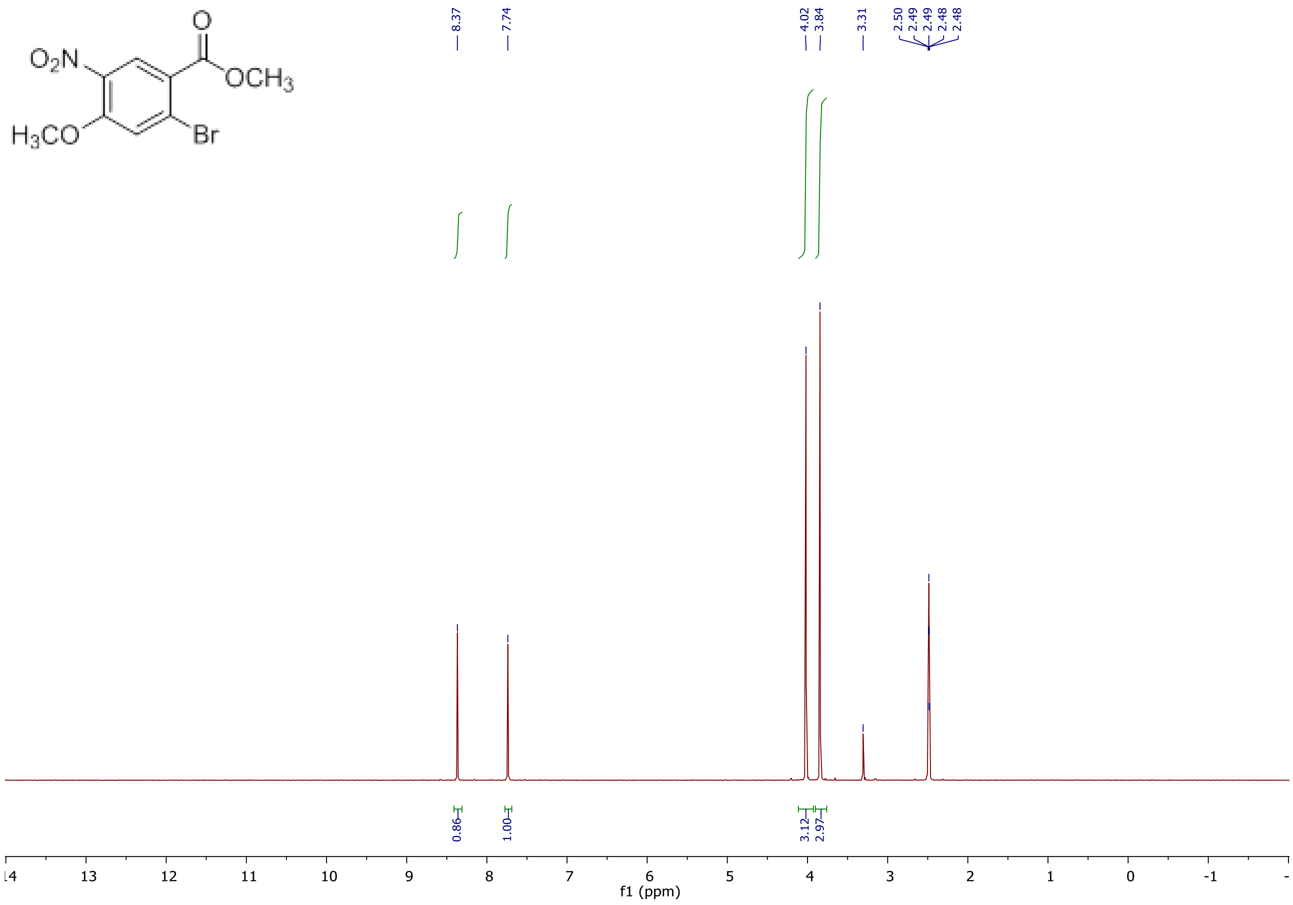

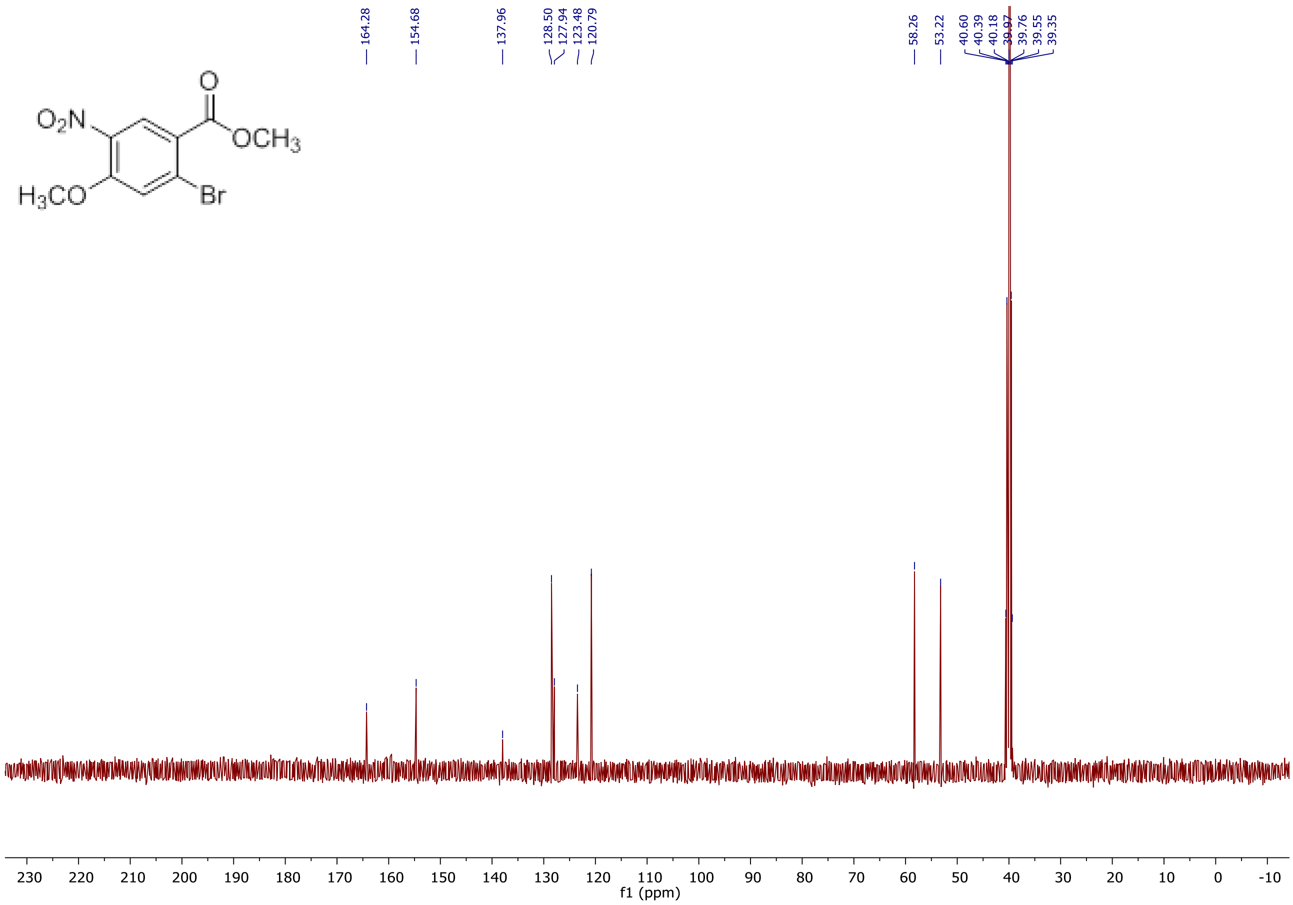

2-Bromo-4-methoxy-5-nitrobenzoic acid (**117**)

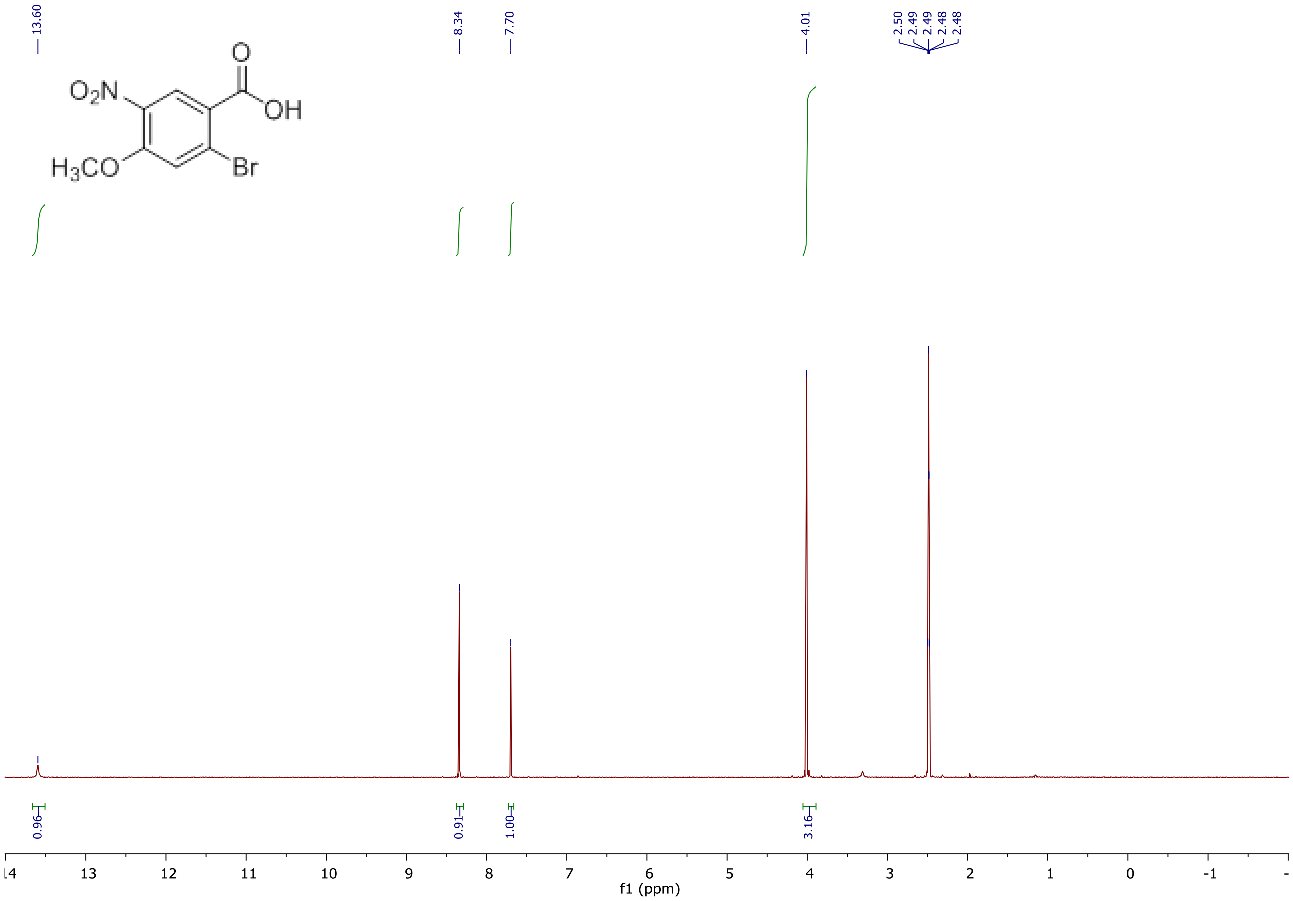

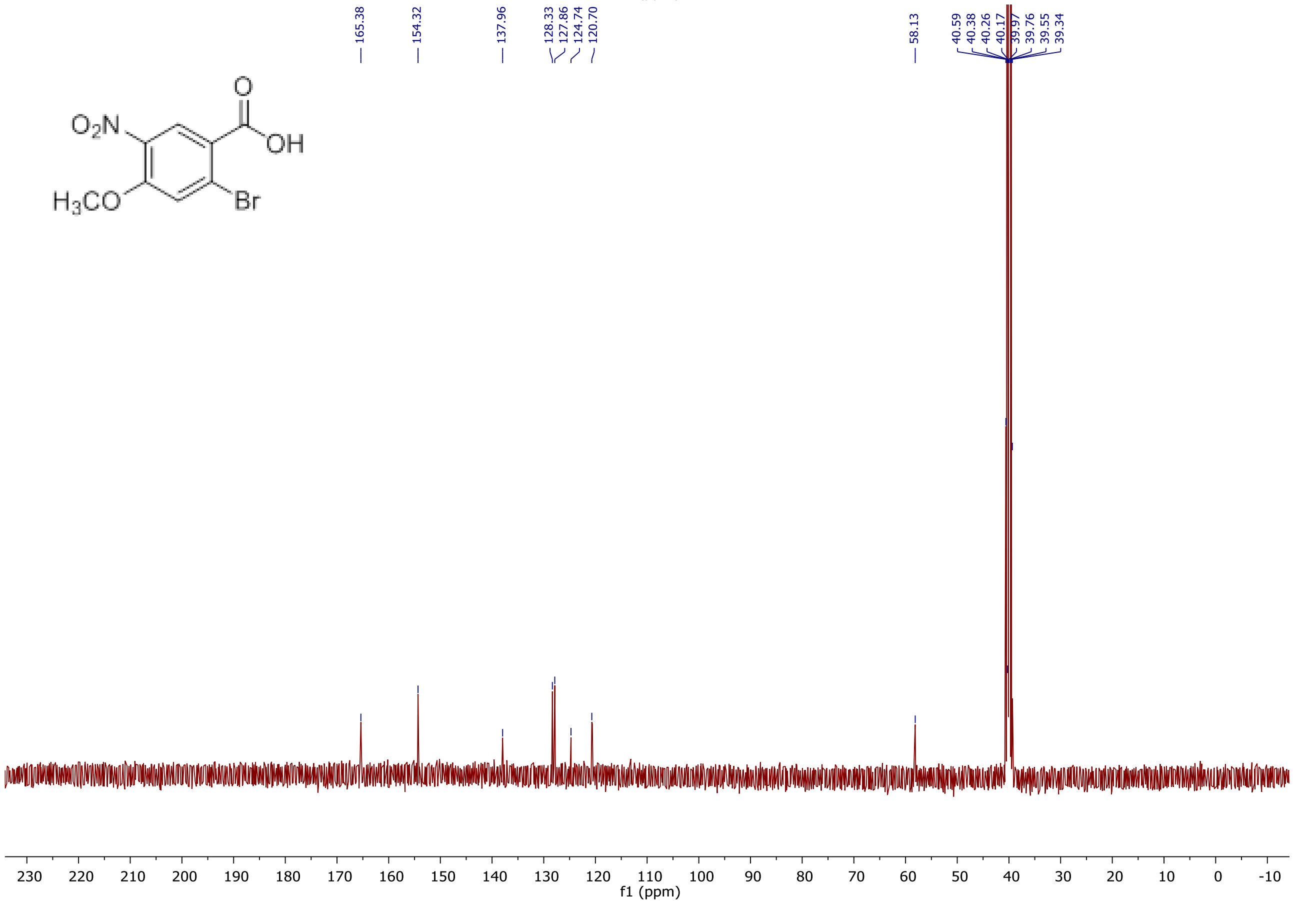

3-Hydroxy-9-methoxy-8-nitro-6H-benzo[c]chromen-6-one (**122**)

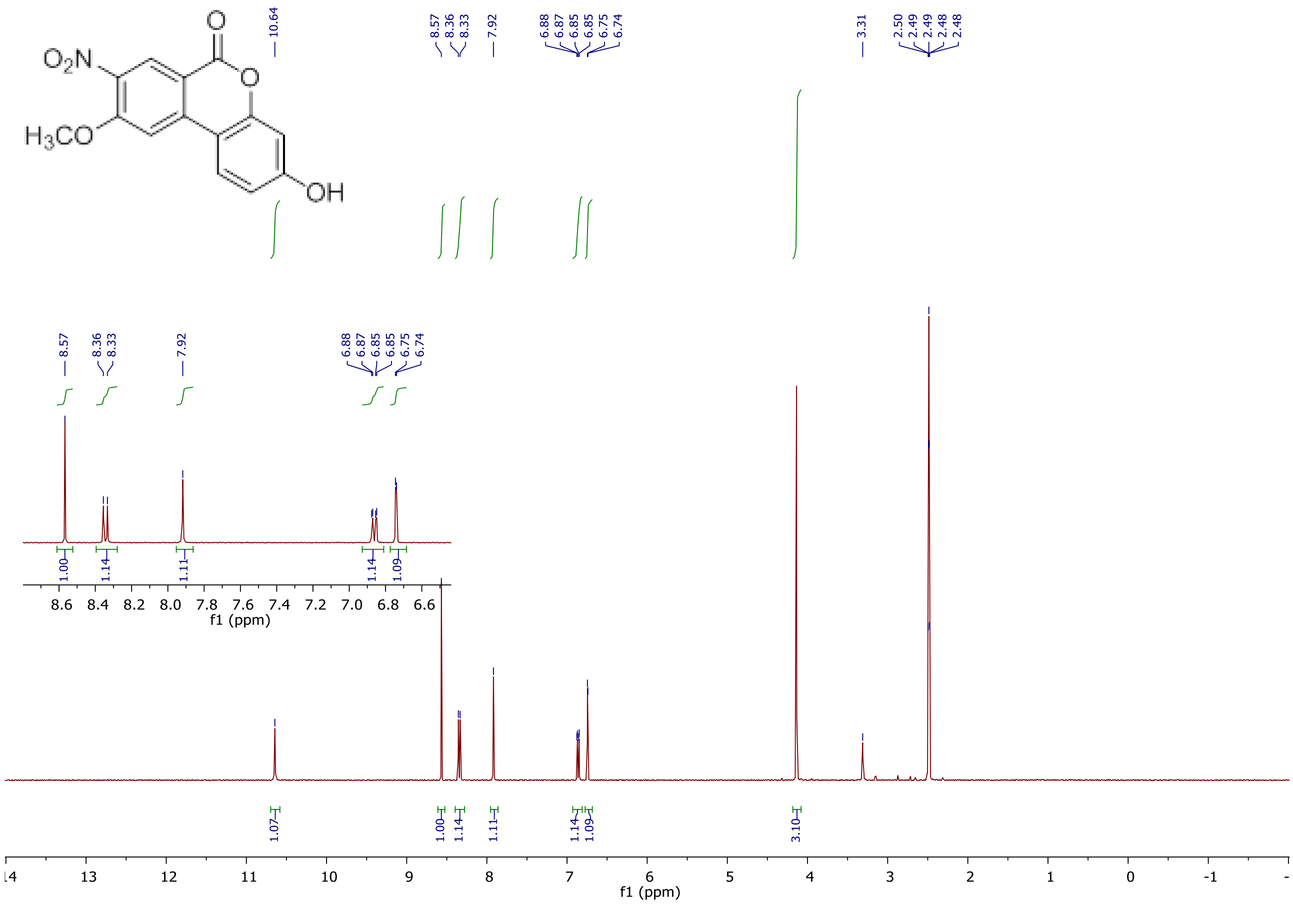

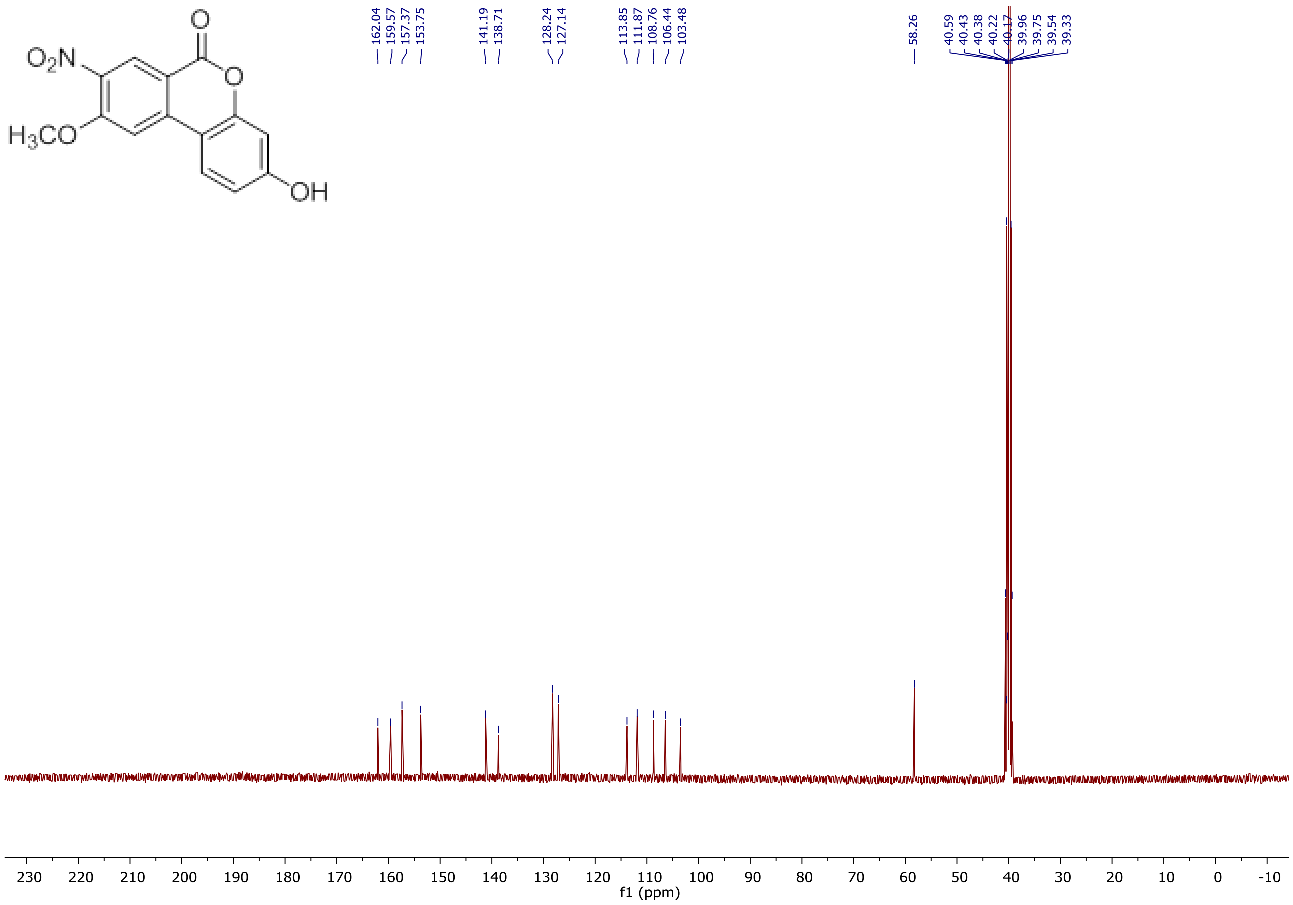

8-Amino-3-hydroxy-9-methoxy-6H-benzo[c]chromen-6-one (**125**)

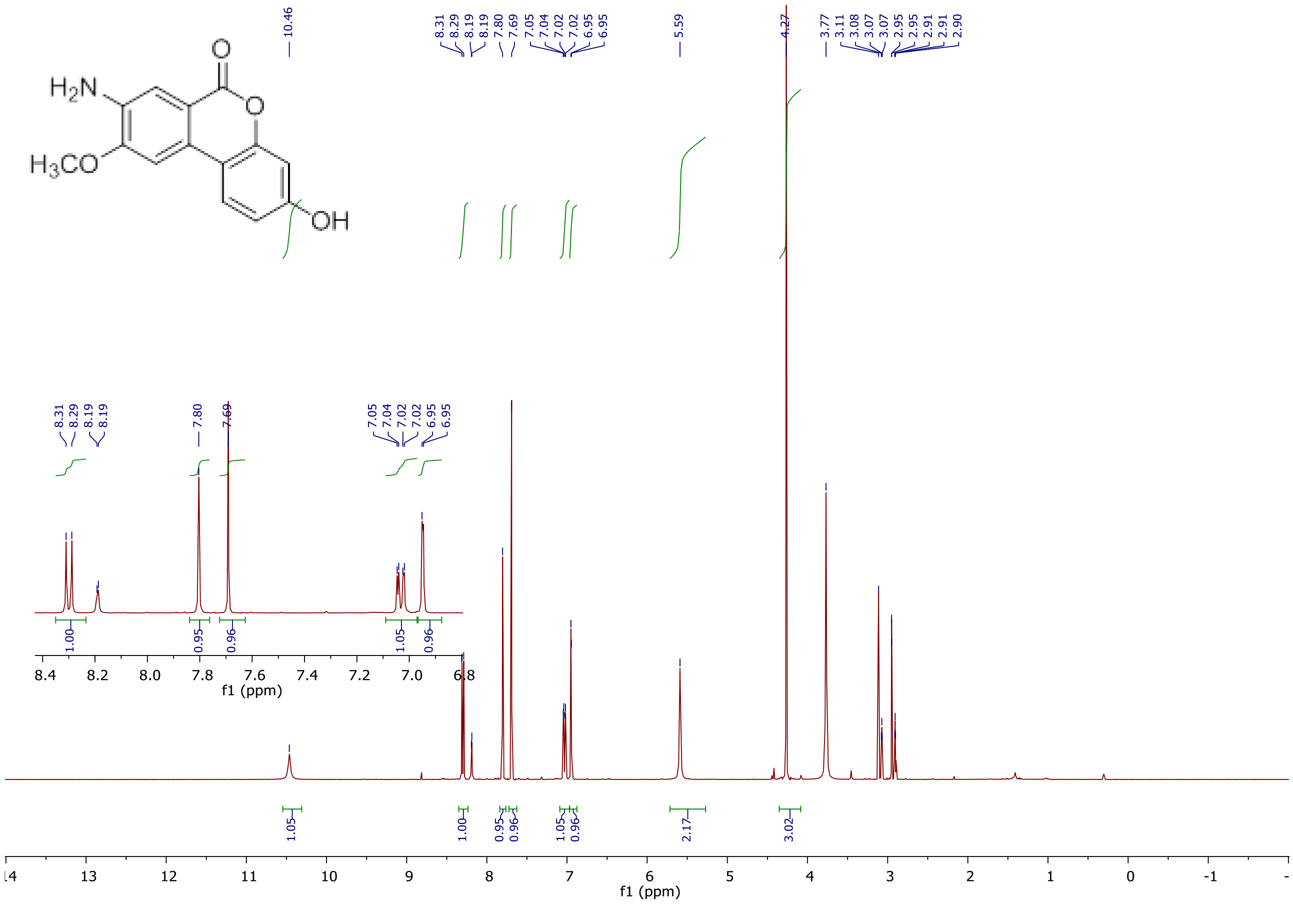

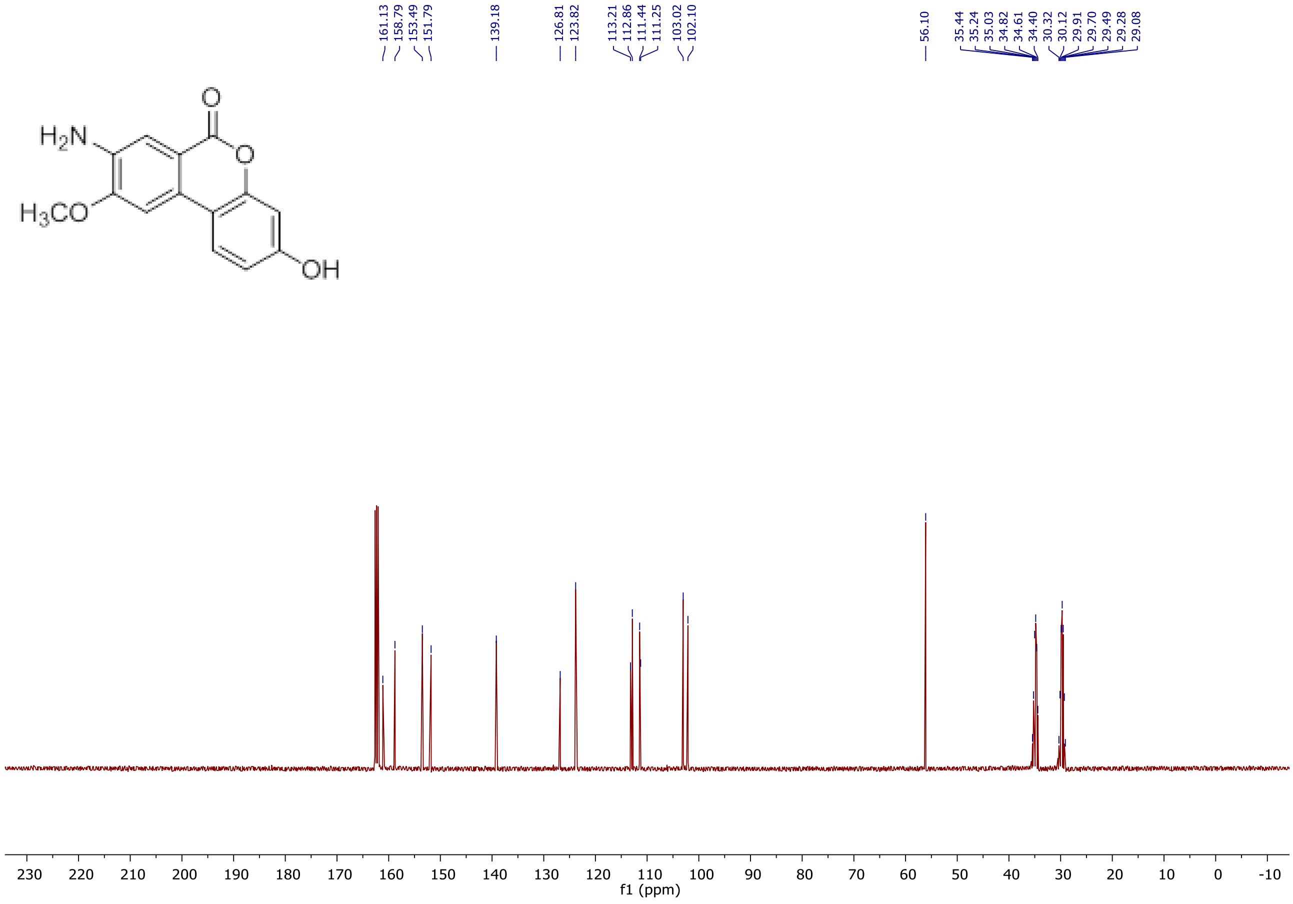

8-Amino-3,9-dihydroxy-6H-benzo[c]chromen-6-one (***126***)

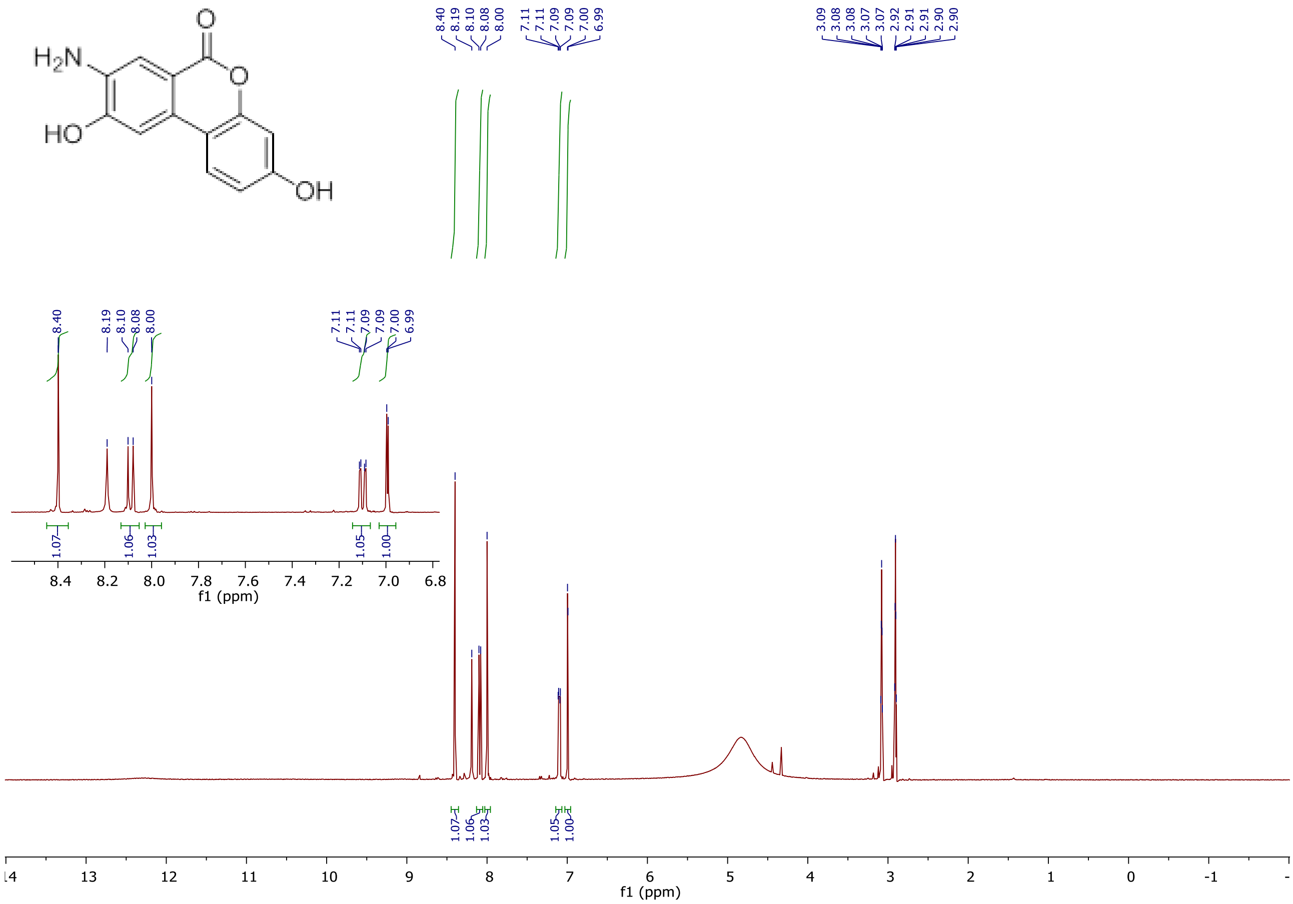

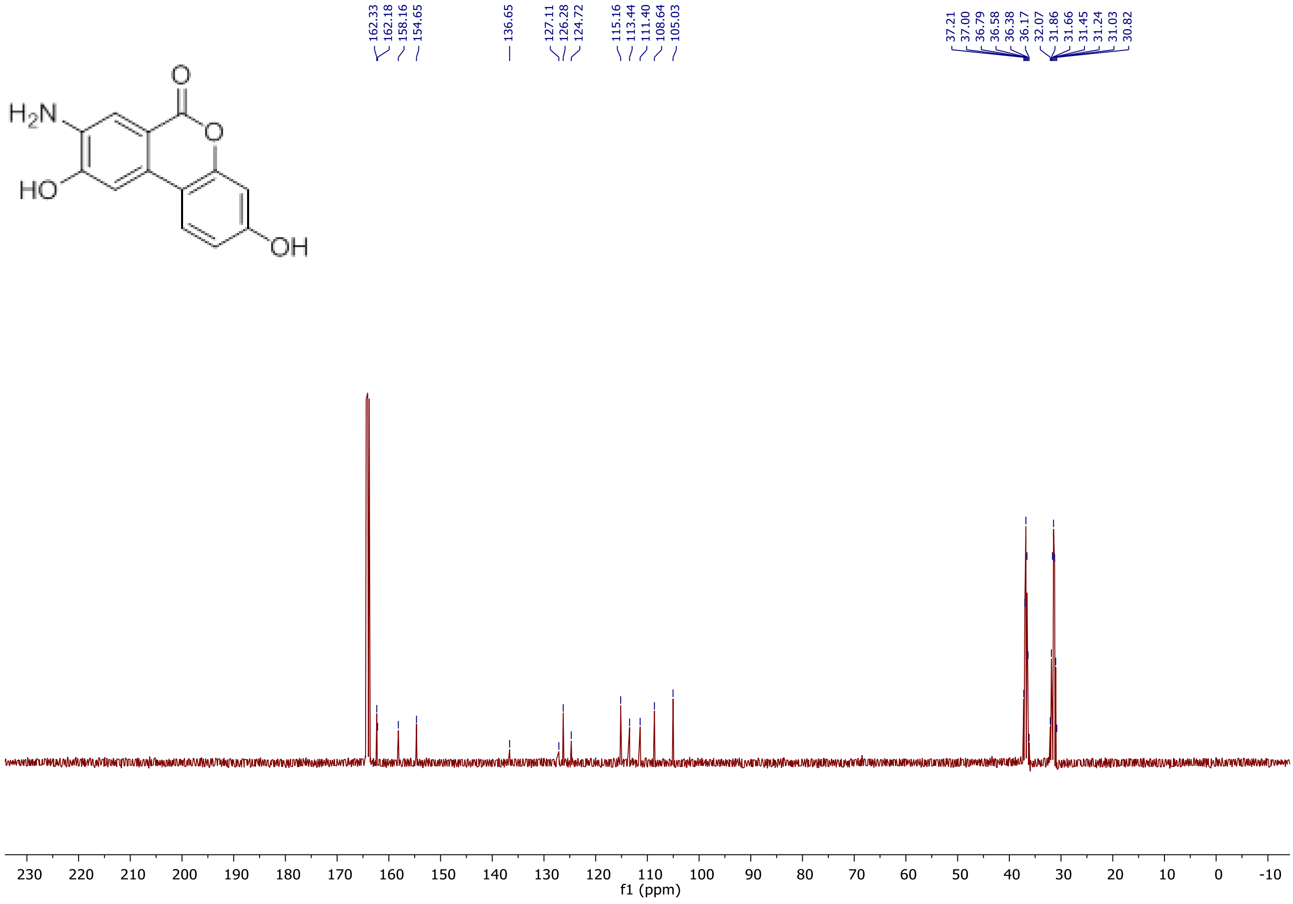

N-(3,9-Dihydroxy-6-oxo-6*H*-benzo[*c*]chromen-8-yl)acetamide (**128**)

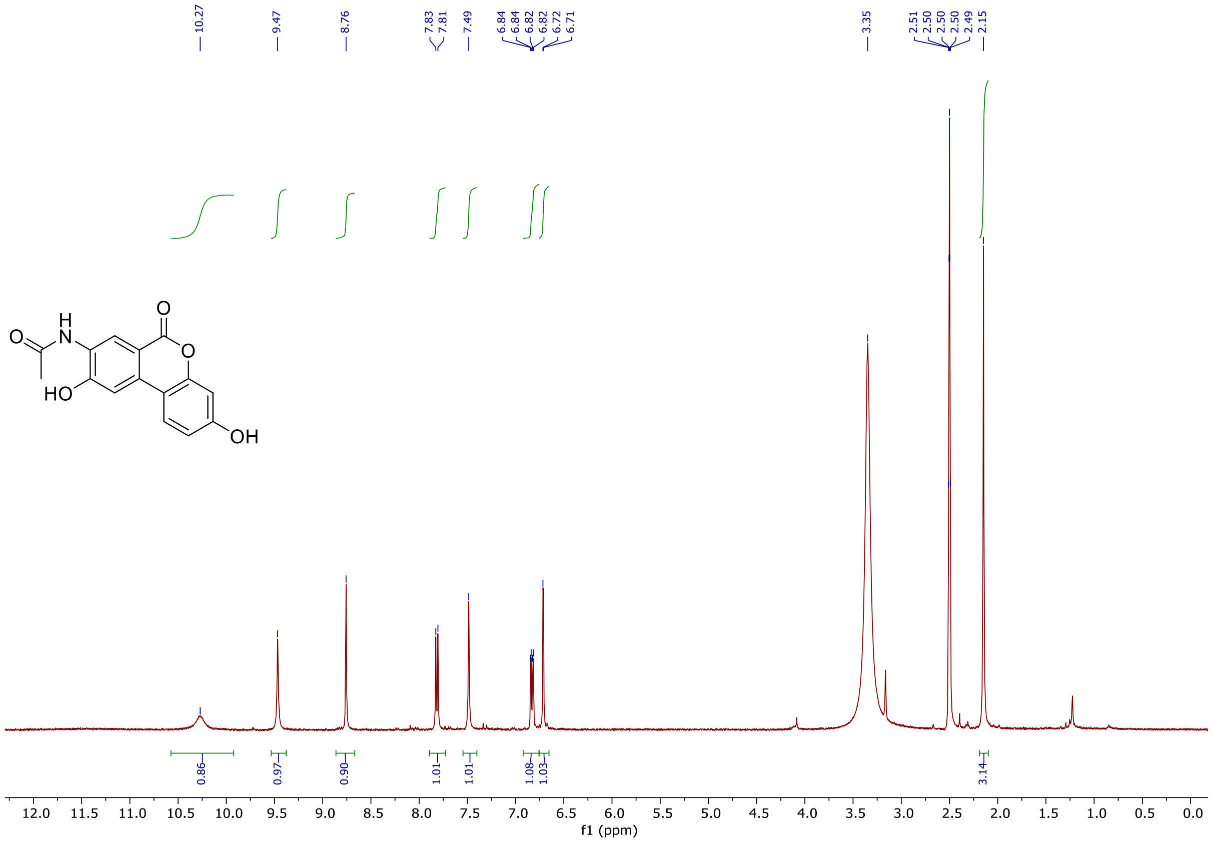

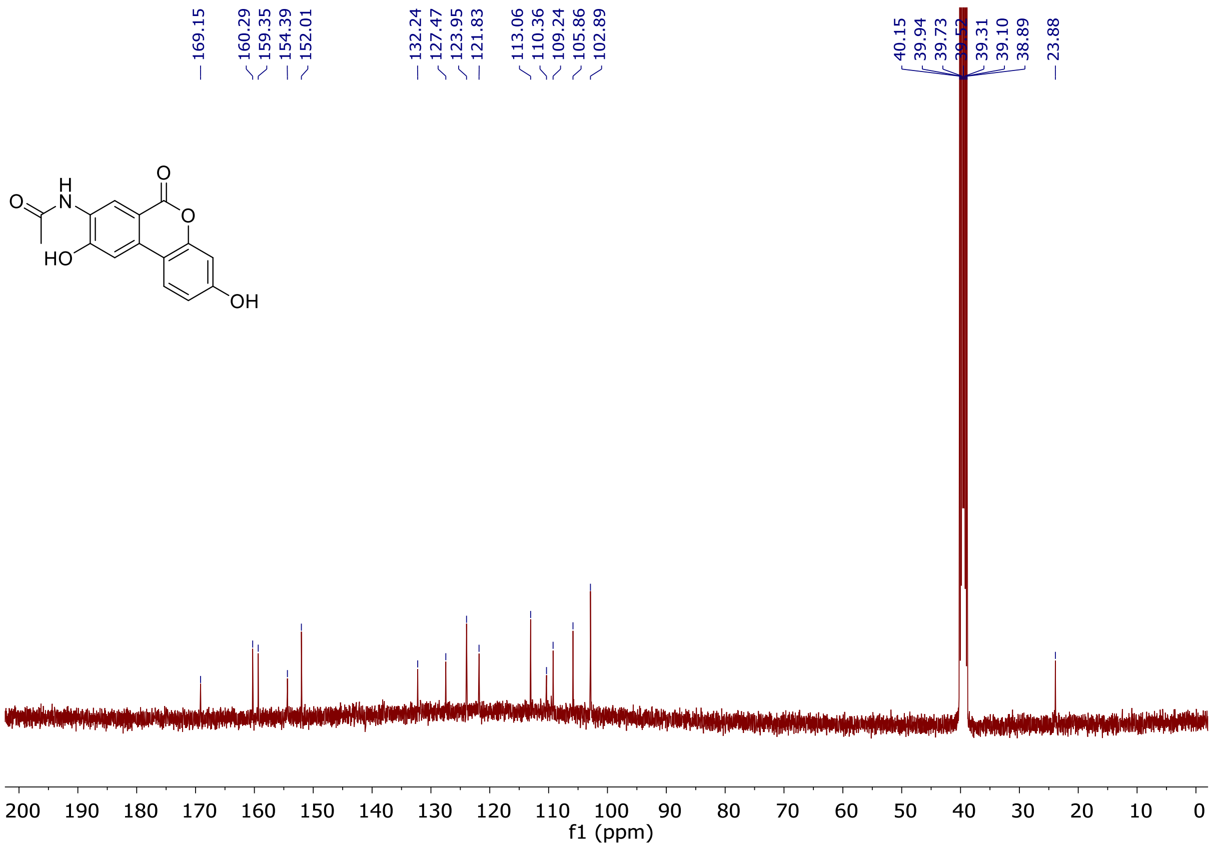

2-Bromo-4,5-dinitrobenzoic acid (**129**)

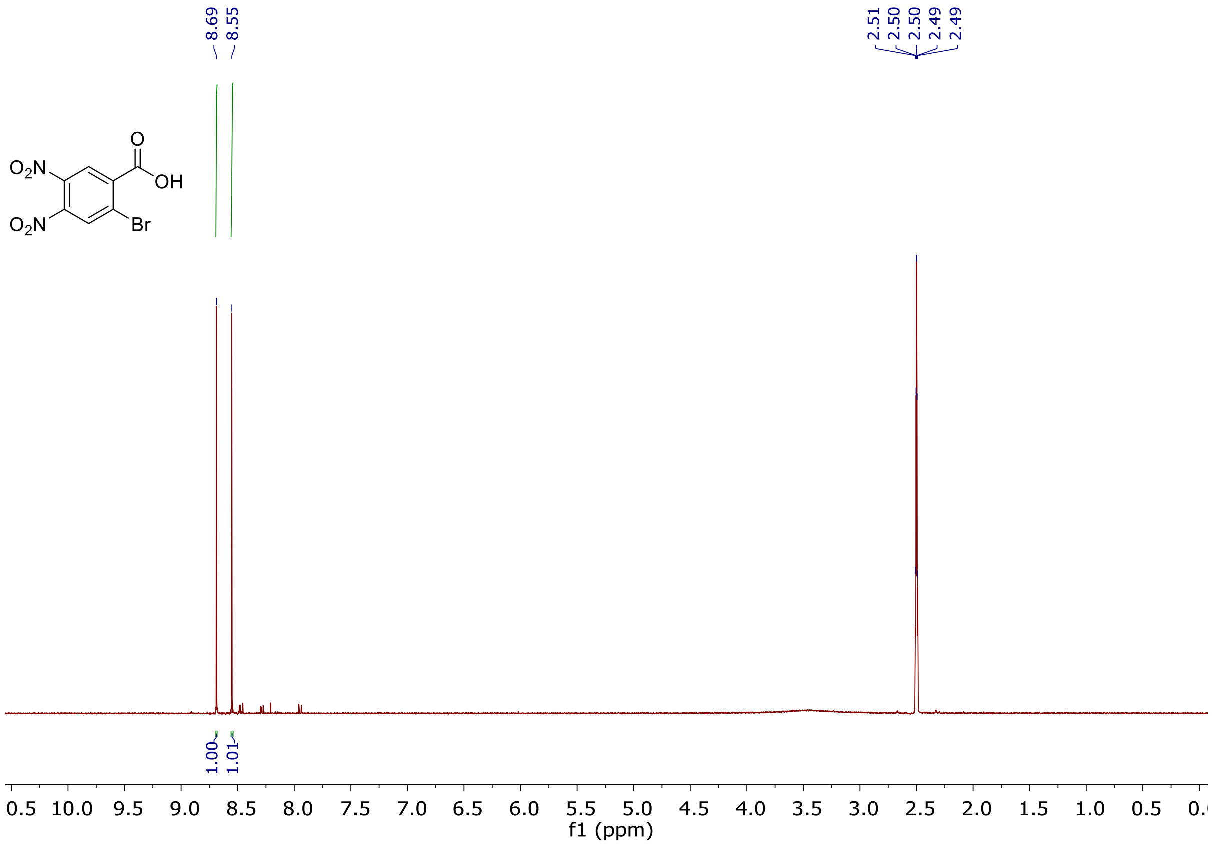

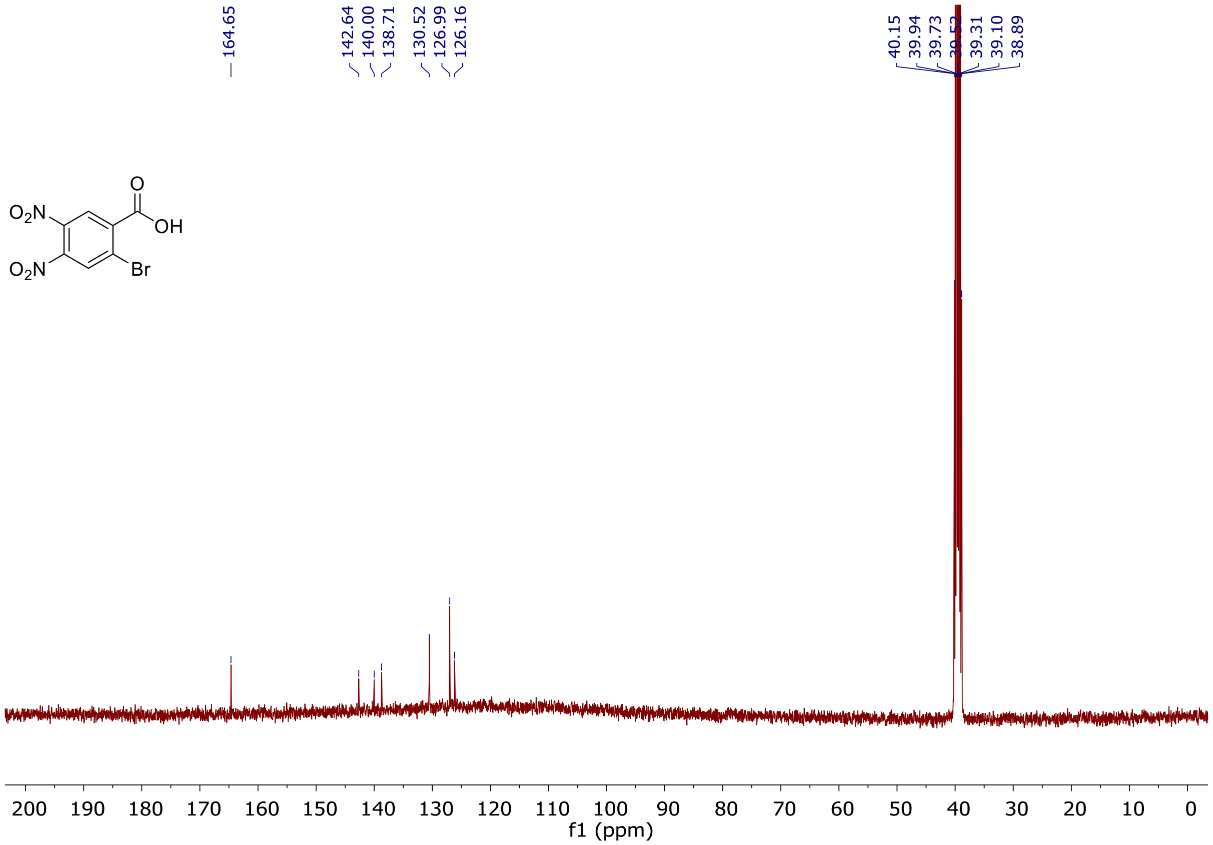

Methyl 4-(benzylamino)-2-bromo-5-nitrobenzoate (**130**)

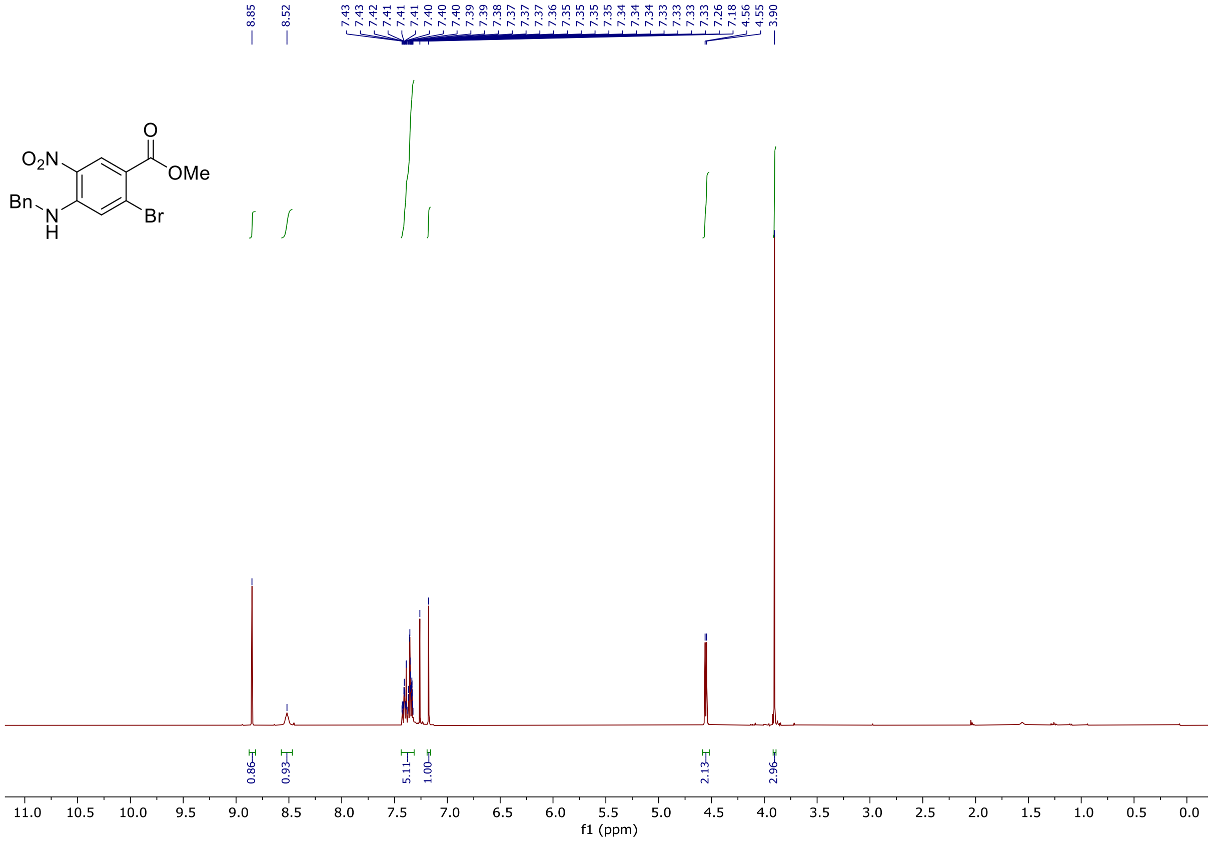

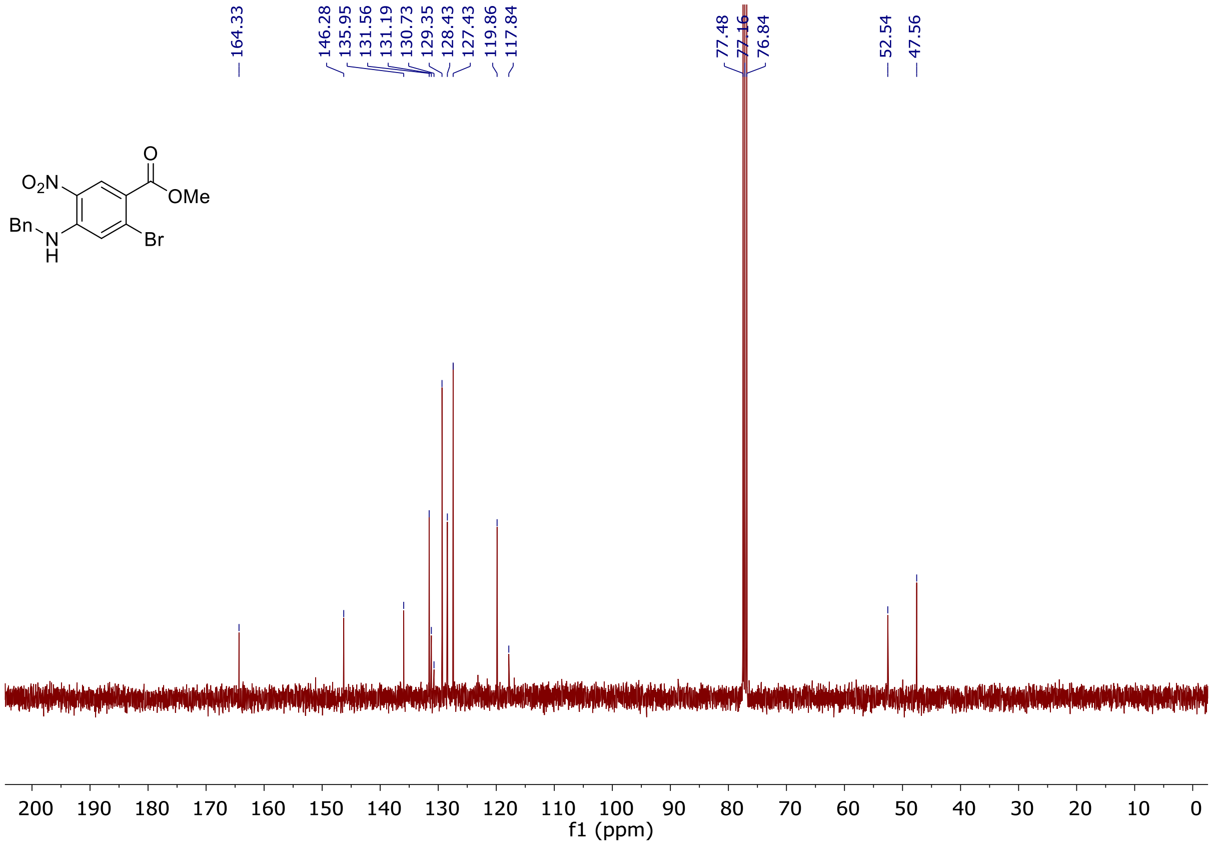

Methyl 5-(benzylamino)-4'-(benzyloxy)-4-nitro-[1,1'-biphenyl]-2-carboxylate (**132**)

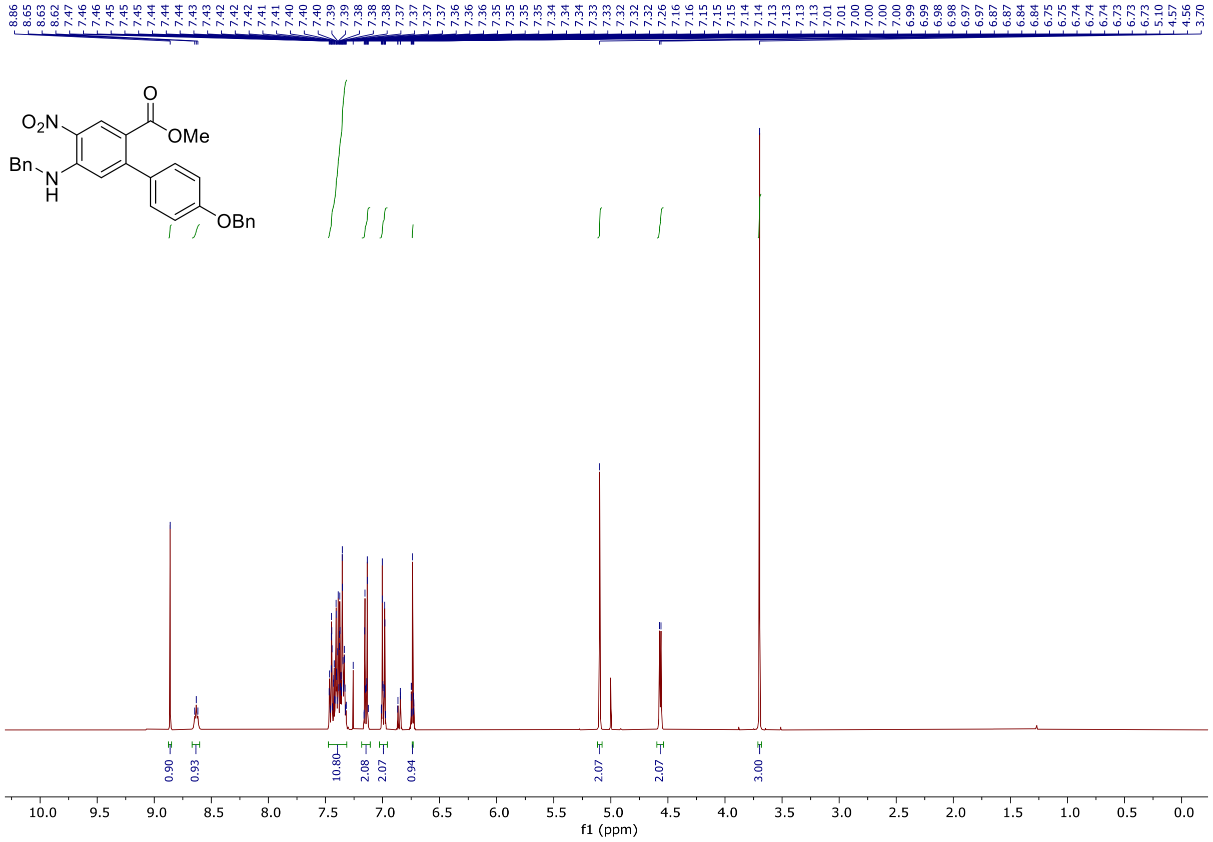

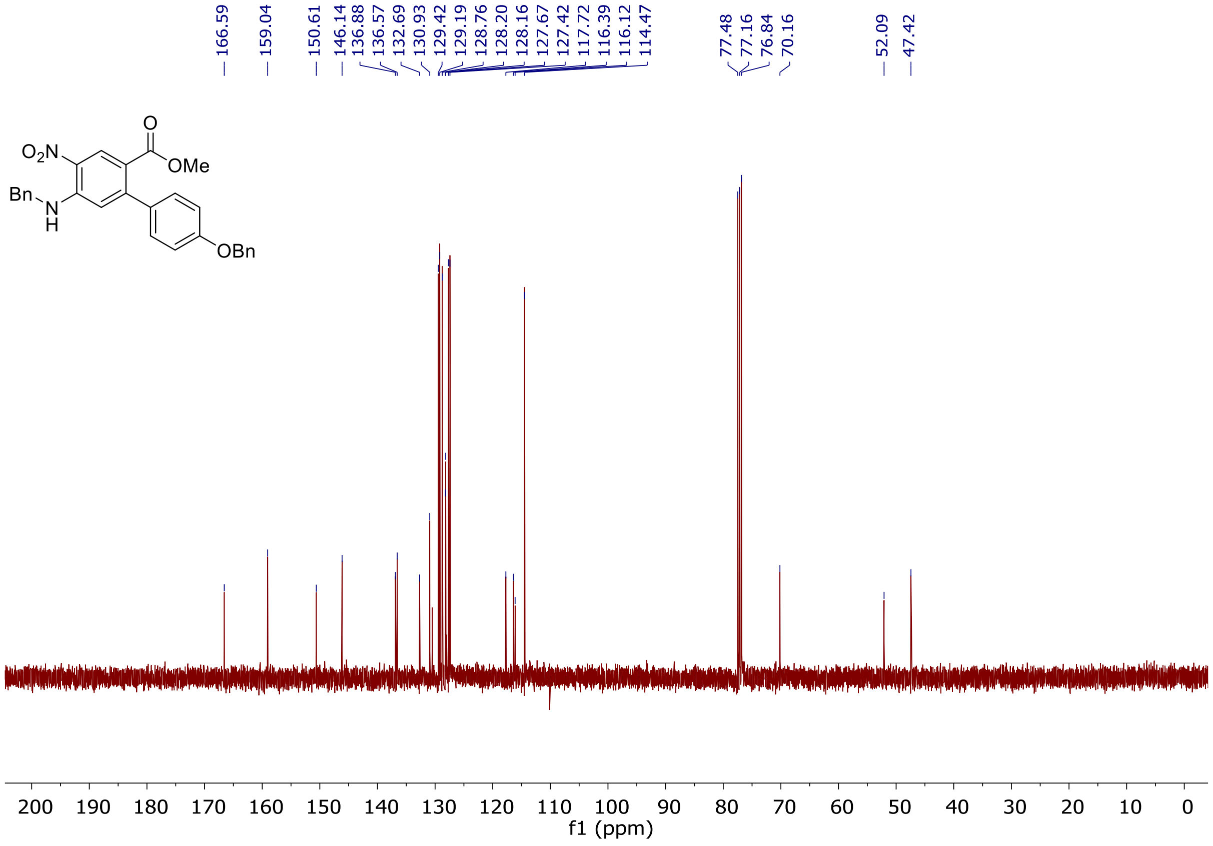

5-(Benzylamino)-4'-(benzyloxy)-4-nitro-[1,1'-biphenyl]-2-carboxylic acid (**133**)

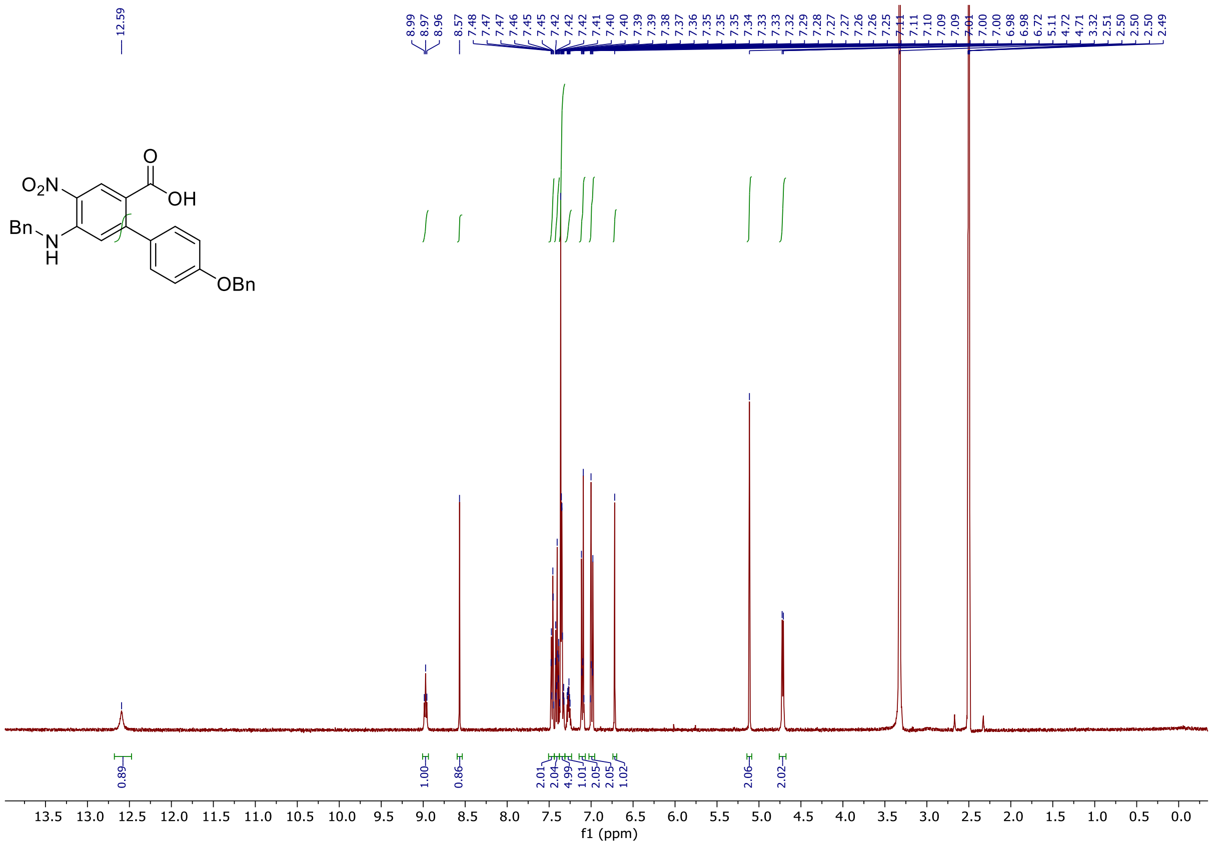

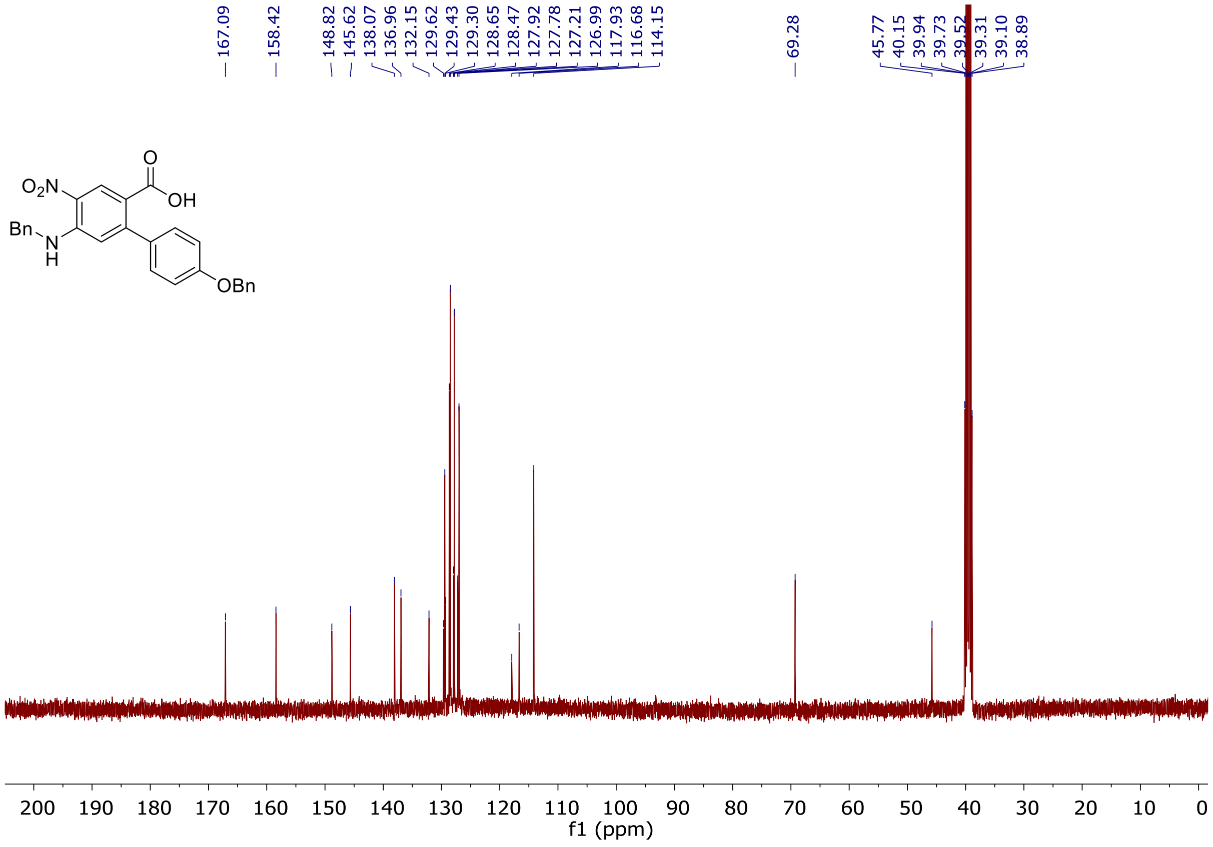

9-(Benzylamino)-3-(benzyloxy)-8-nitro-6H-benzo[c]chromen-6-one (**134**)

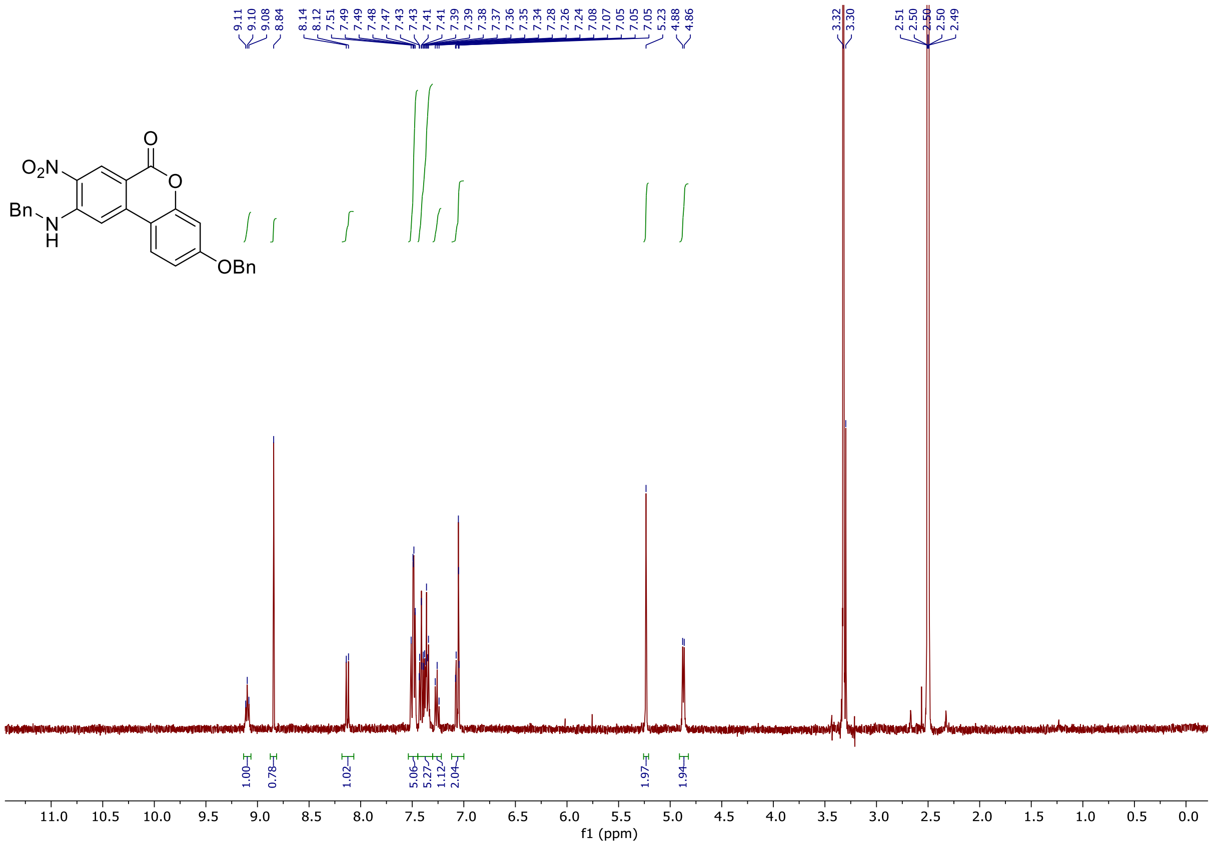

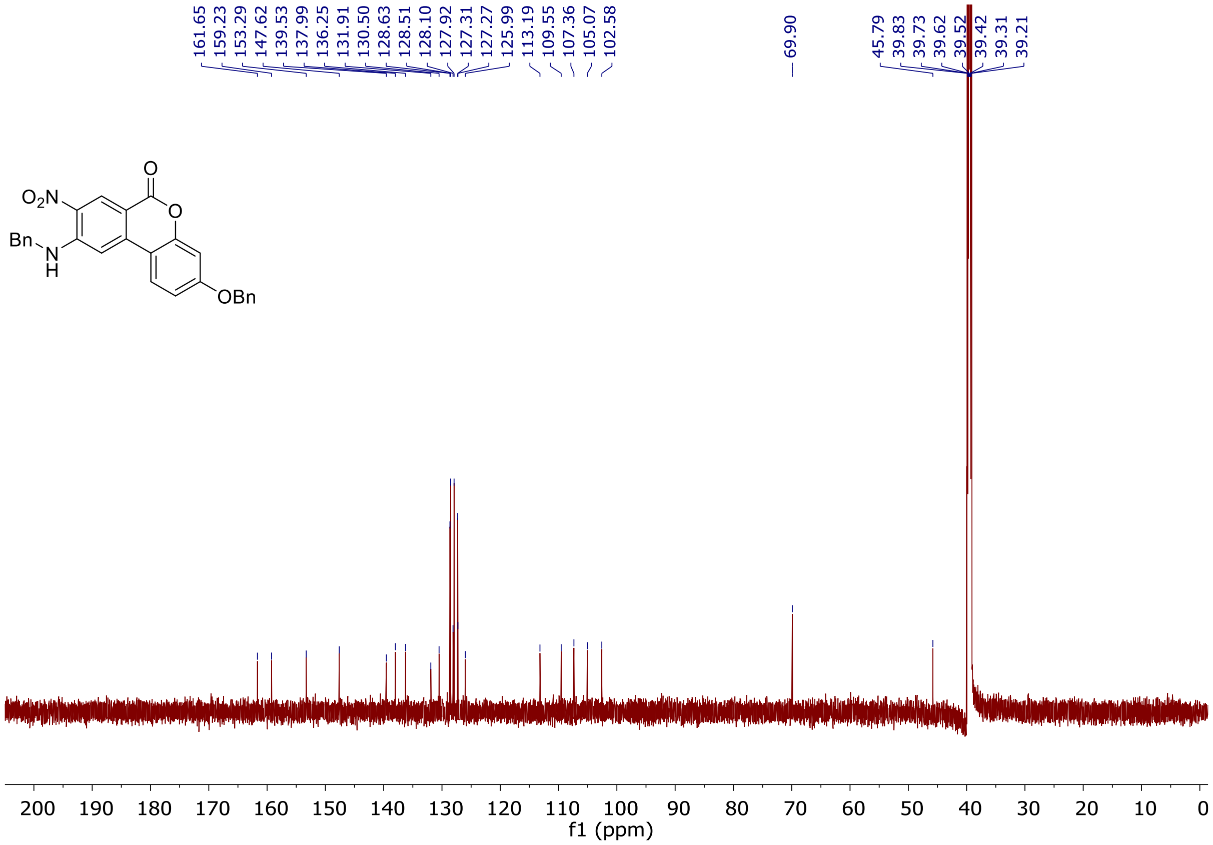

8,9-Diamino-3-hydroxy-6H-benzo[c]chromen-6-one (**135**)

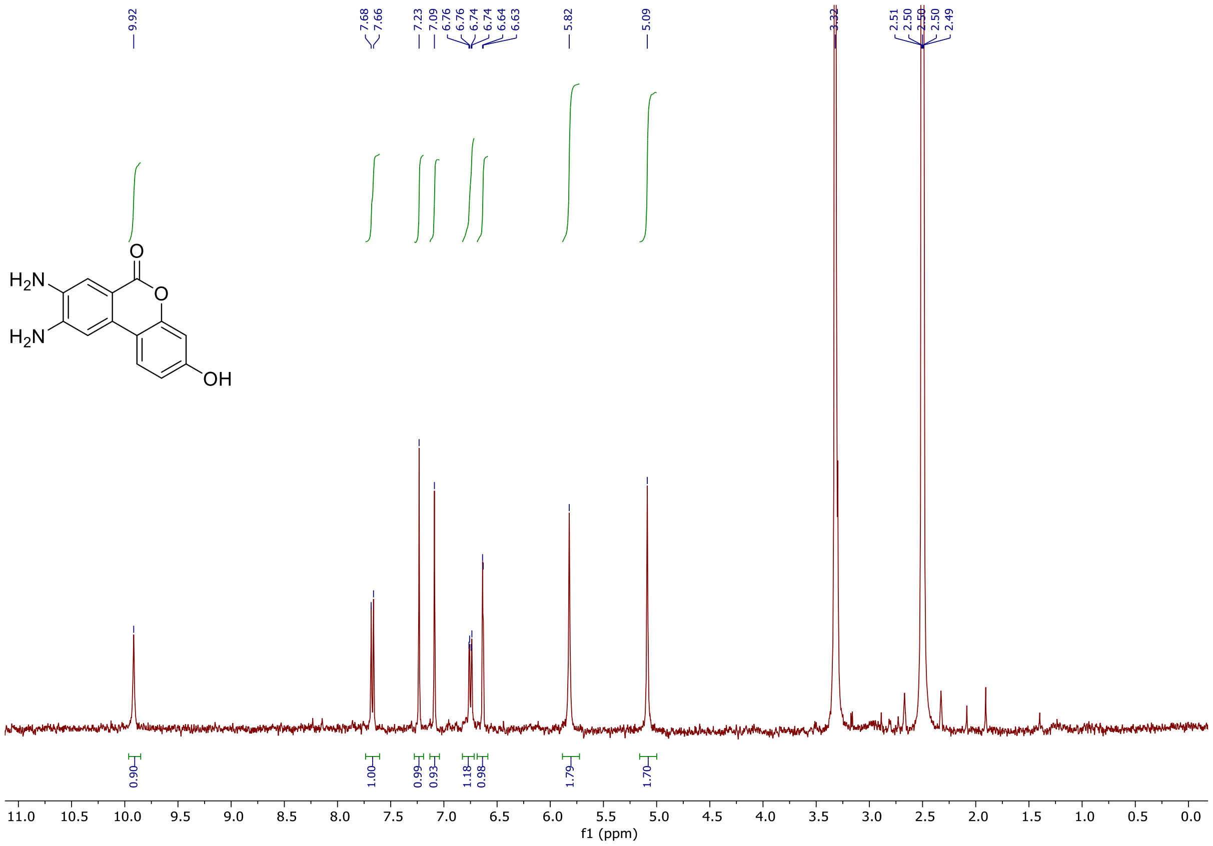

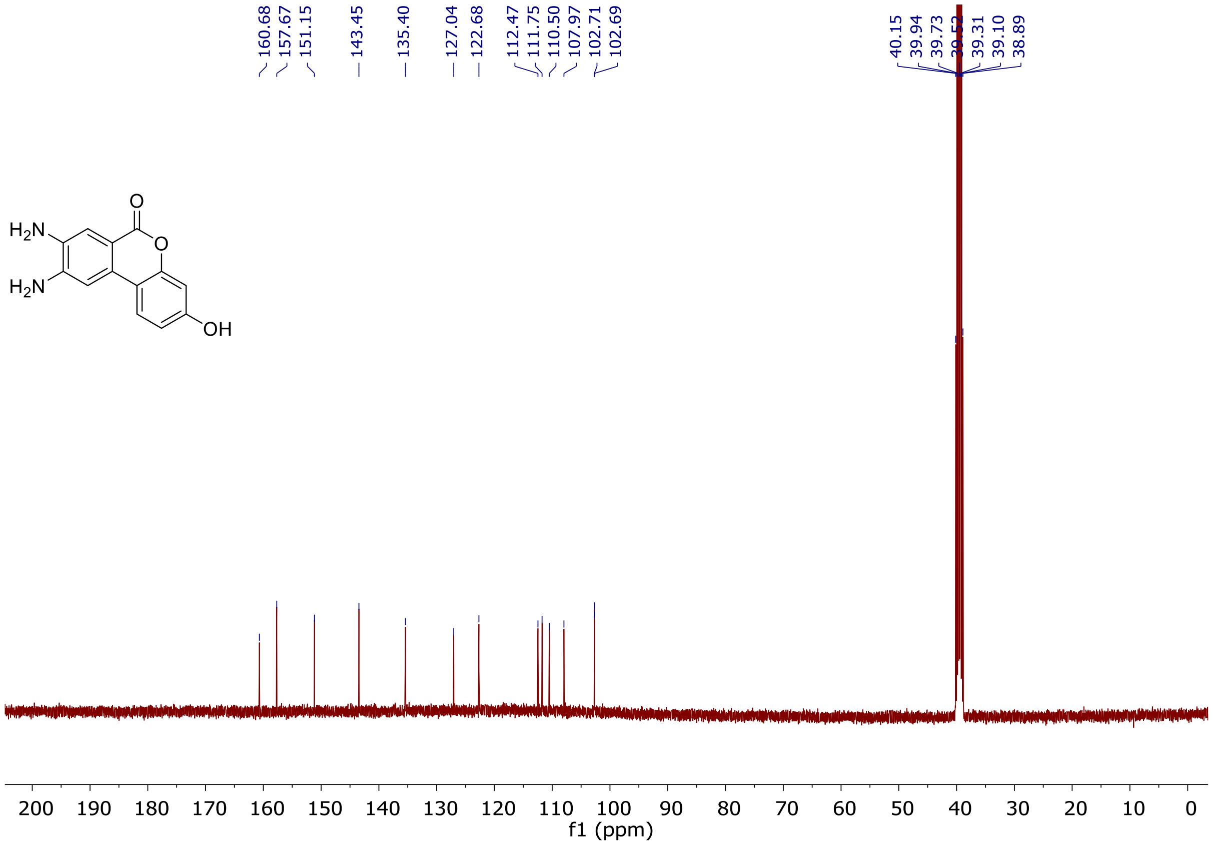

Methyl 4,5-diamino-2-bromobenzoate (**136**)

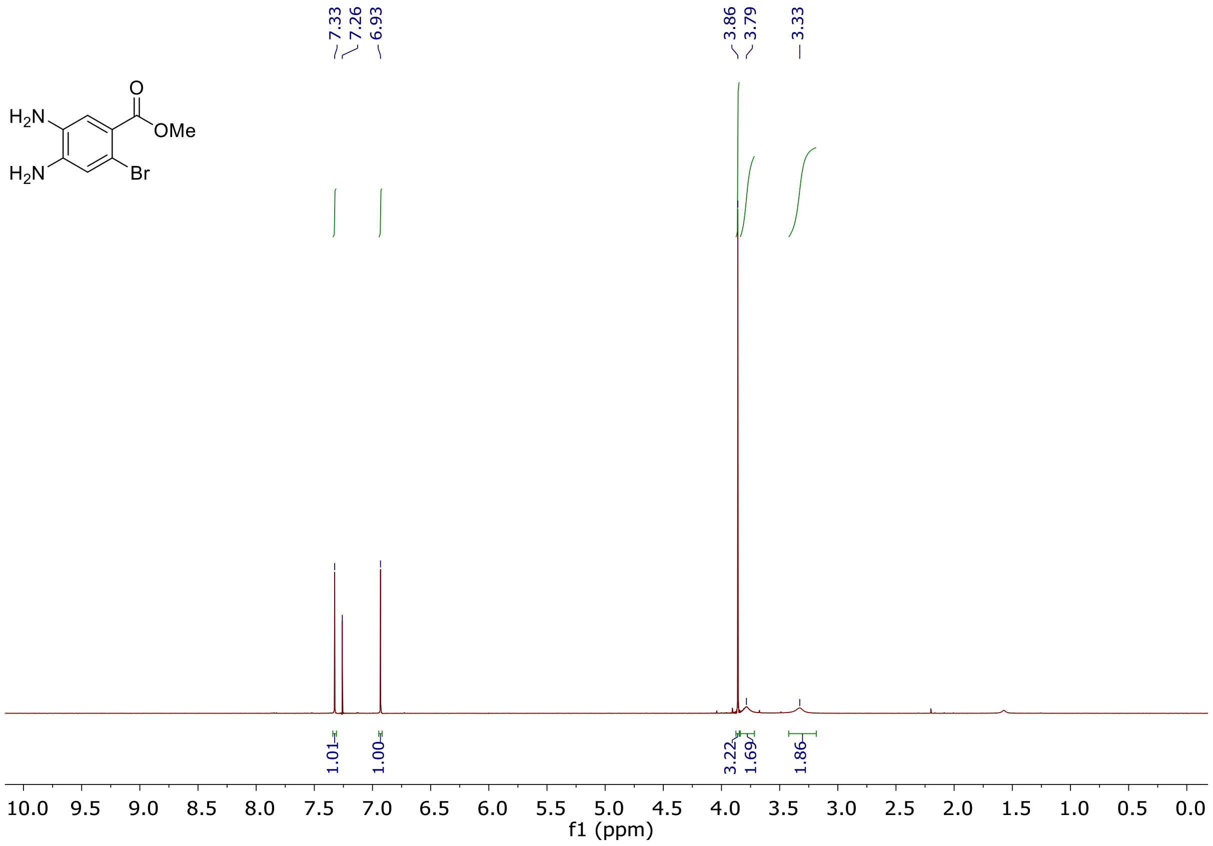

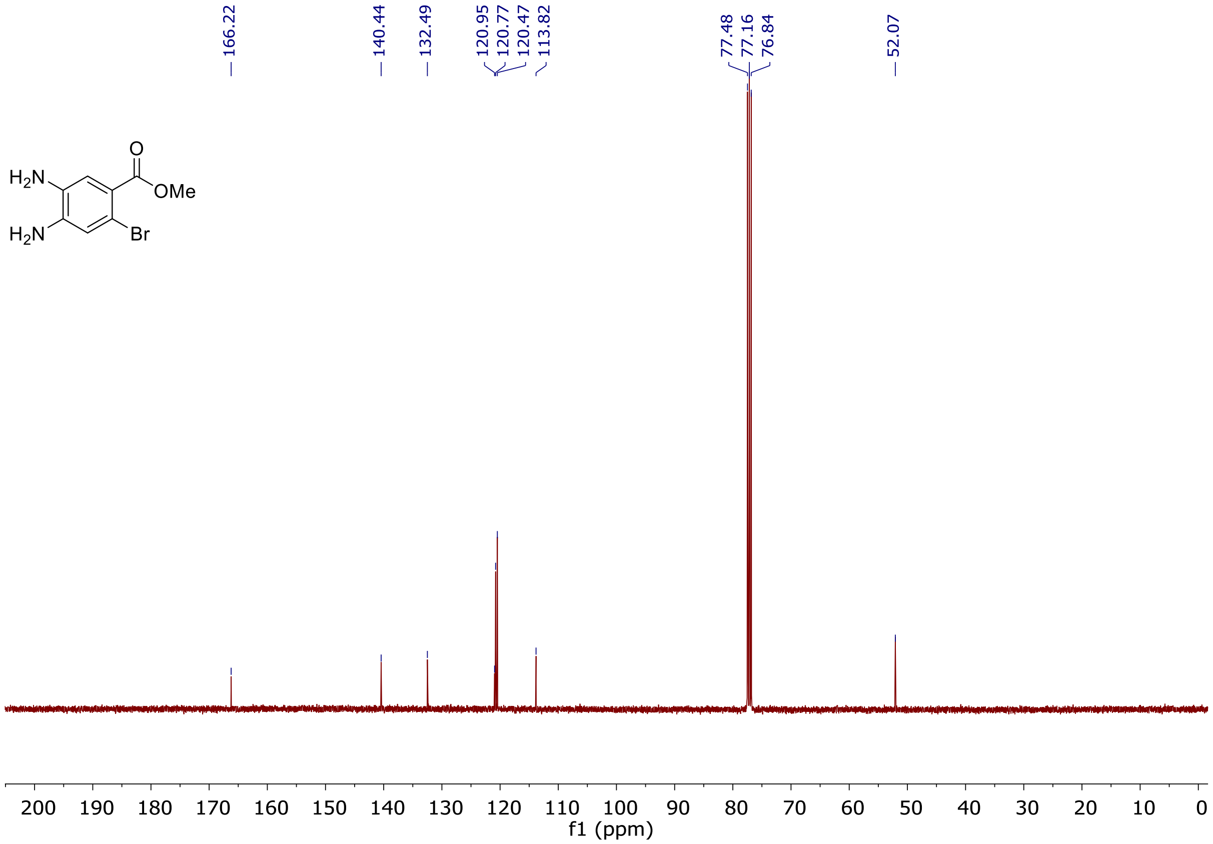

Methyl 4,5-diacetamido-2-bromobenzoate (**137**)

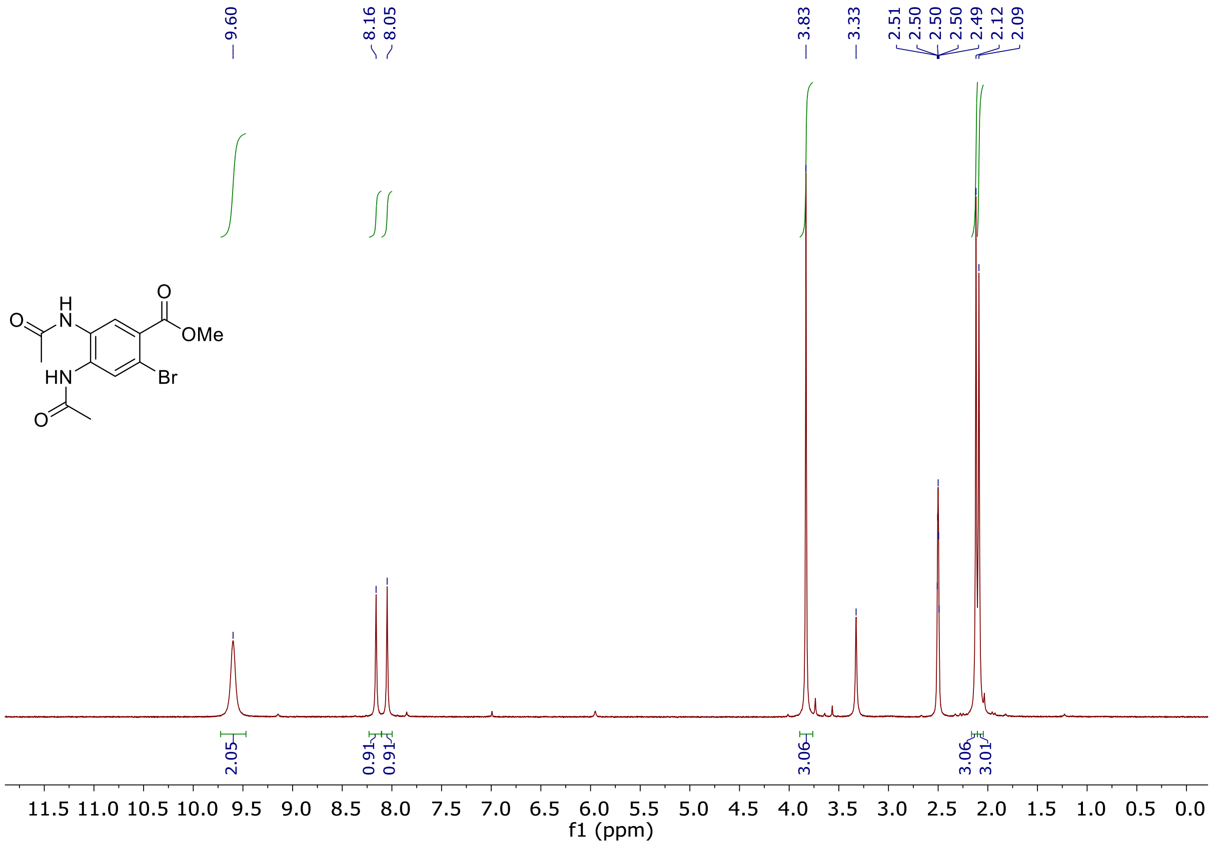

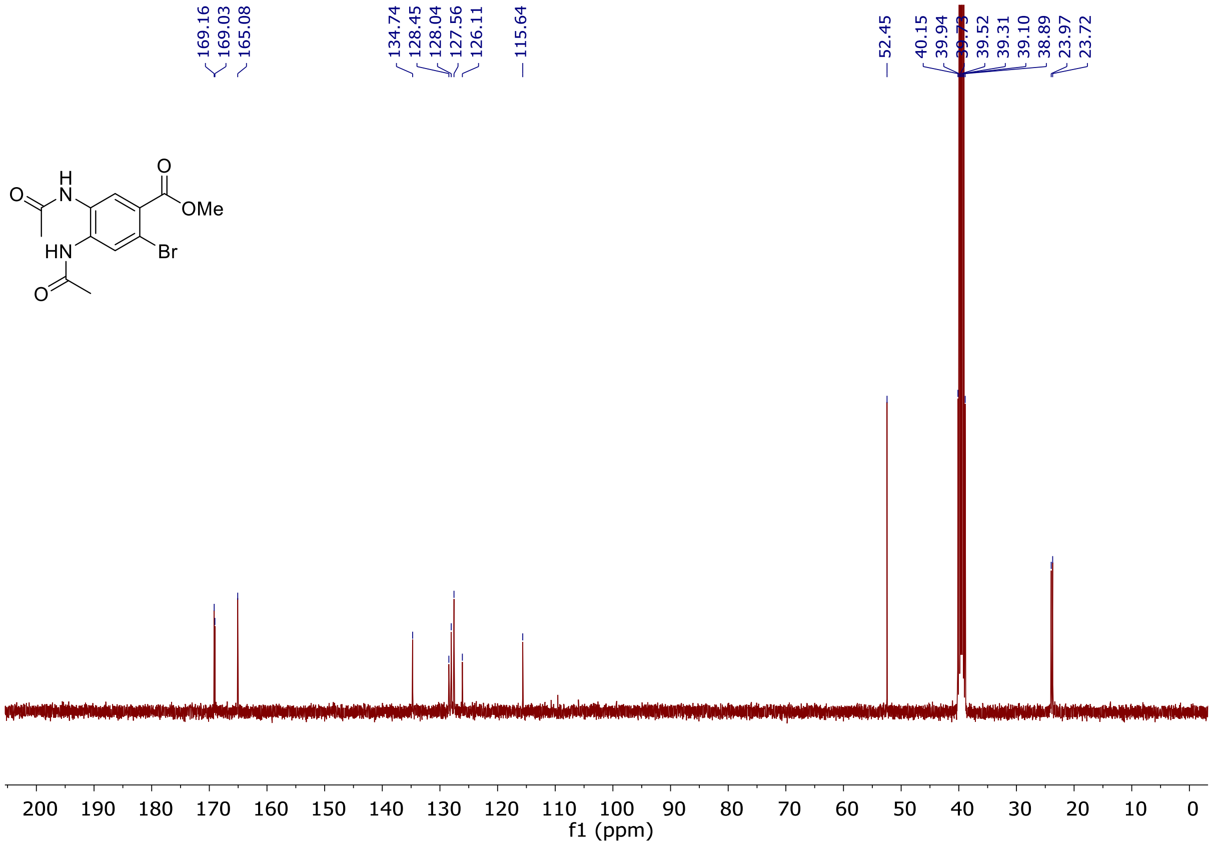

Methyl 4,5-diacetamido-2',4'-dimethoxy-[1,1'-biphenyl]-2-carboxylate (**139**)

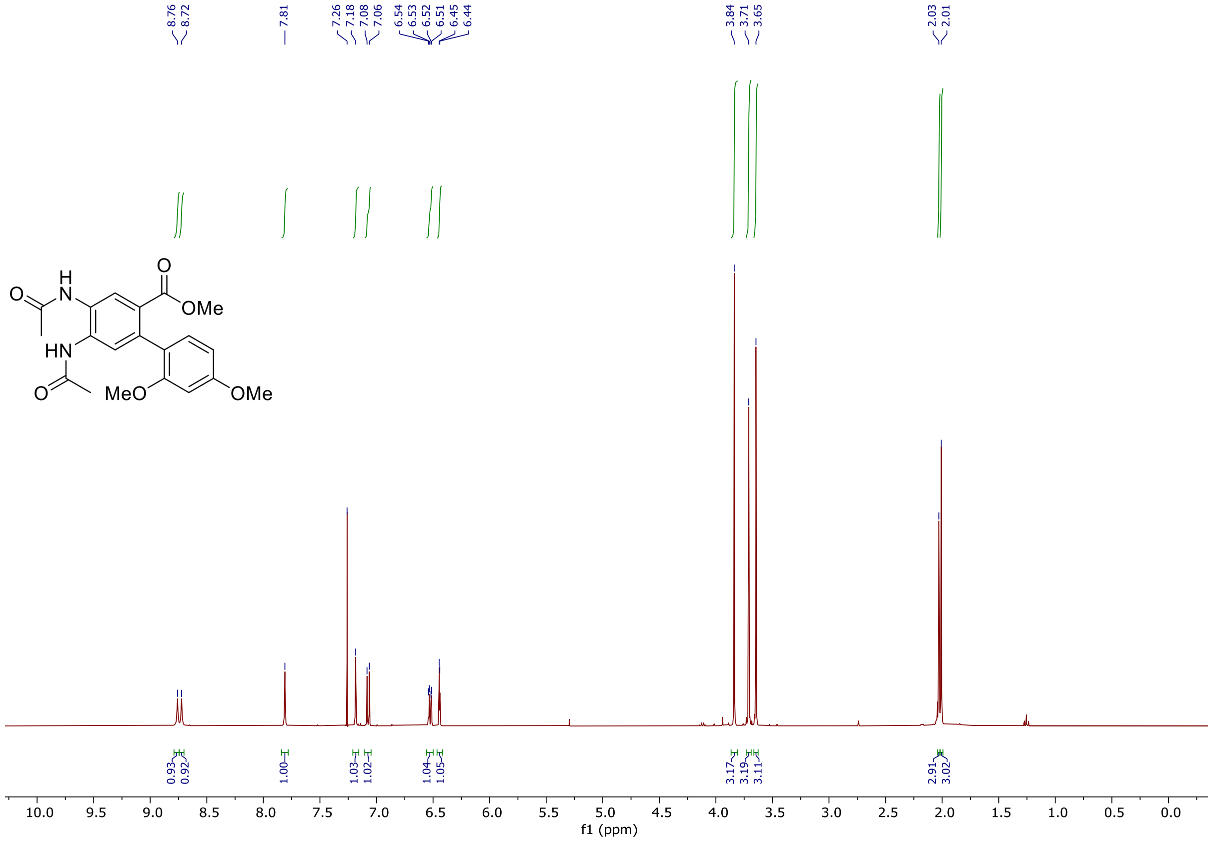

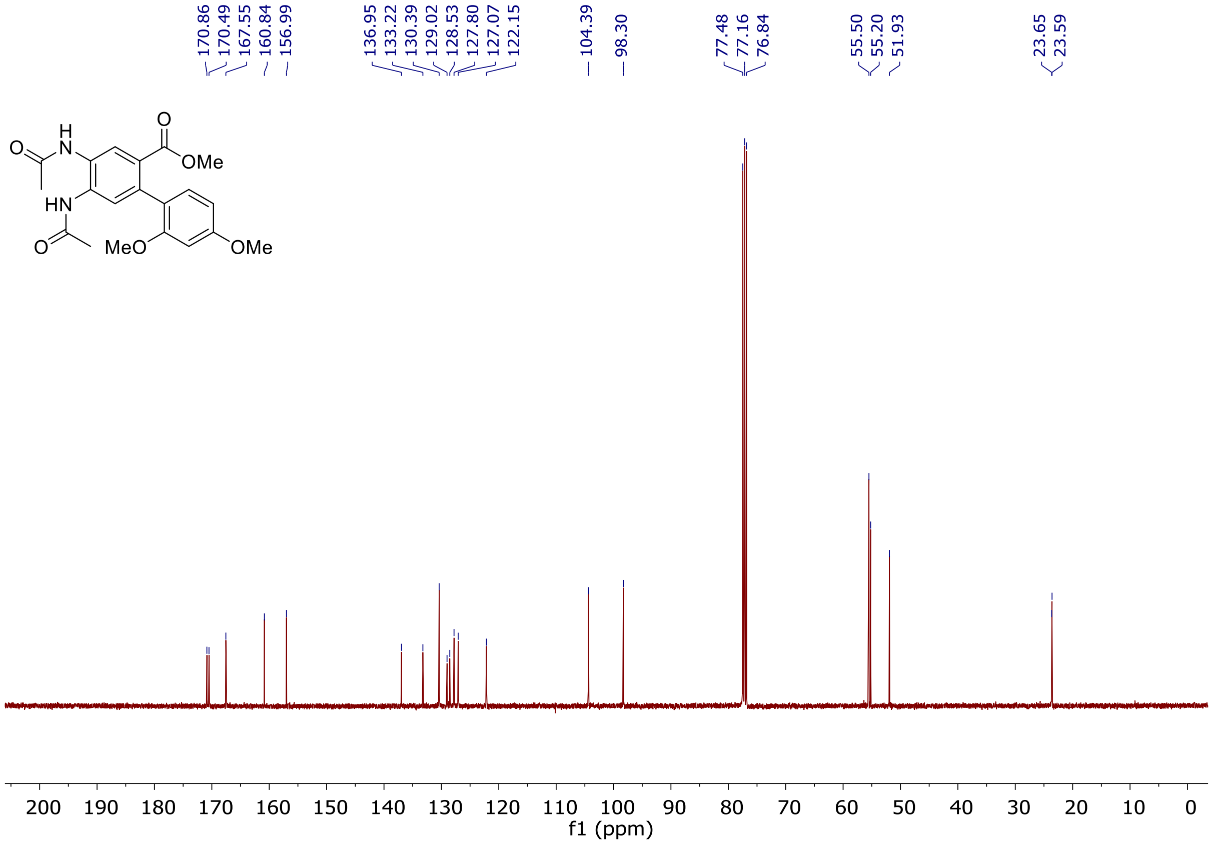

N,N'-(3-Hydroxy-6-oxo-6H-benzo[c]chromene-8,9-diyl)diacetamide (**140**)

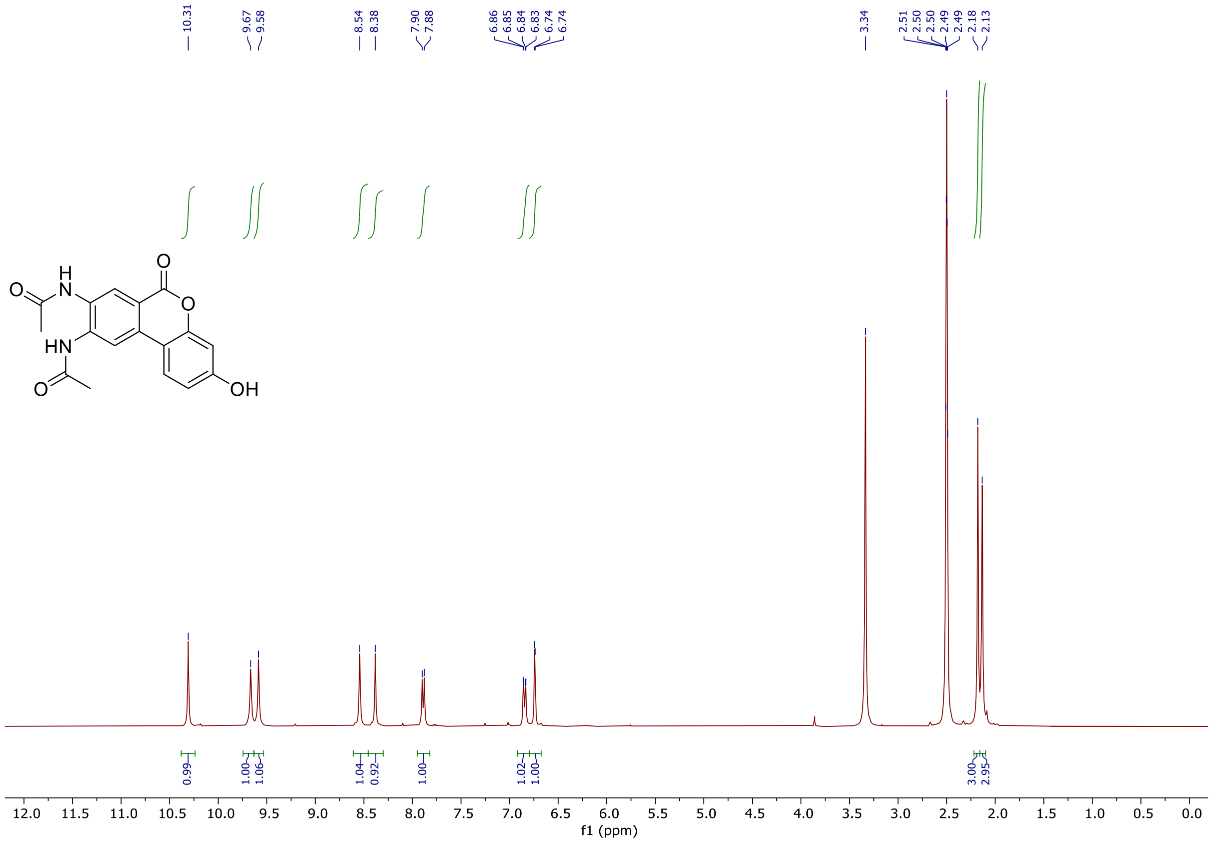

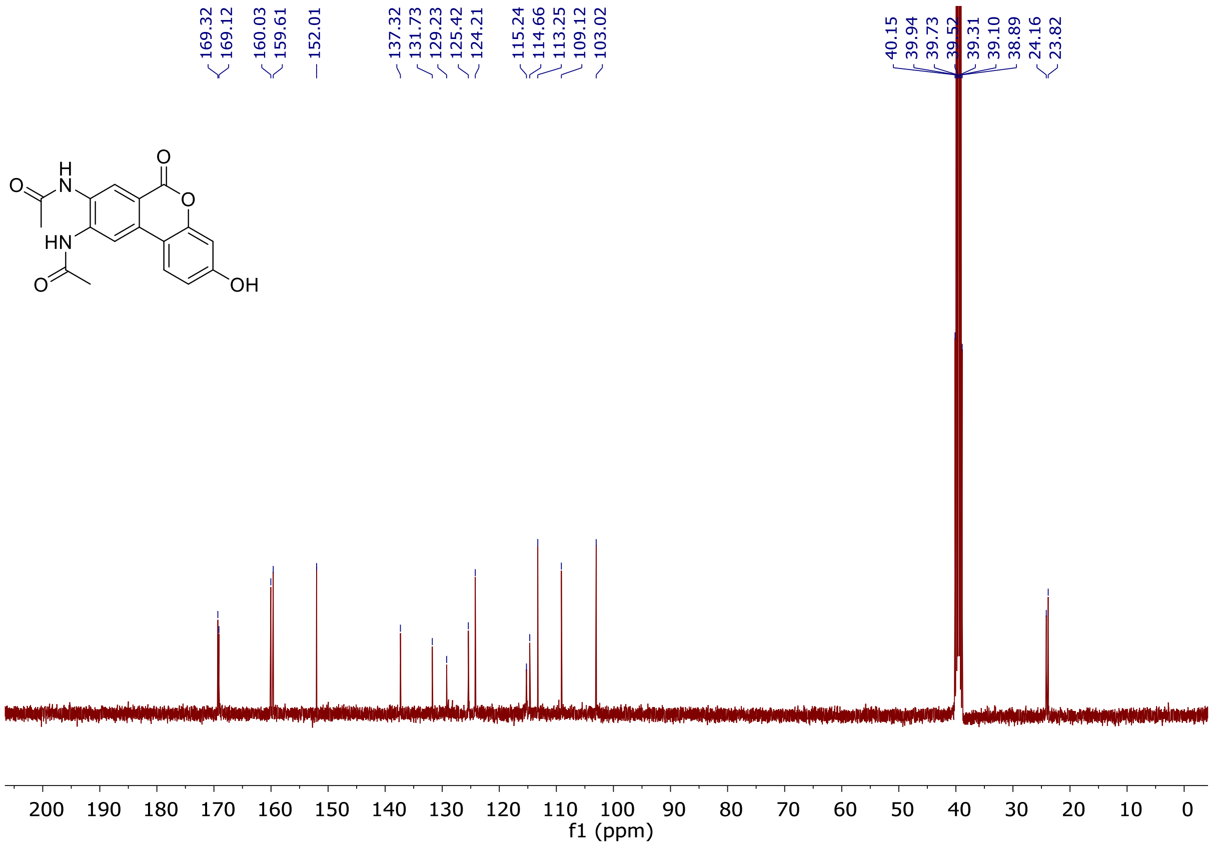

3-Hydroxy-9-methylbenzo[3,4]isochromeno[6,7-*d*]imidazol-6(10*H*)-one (**141**)

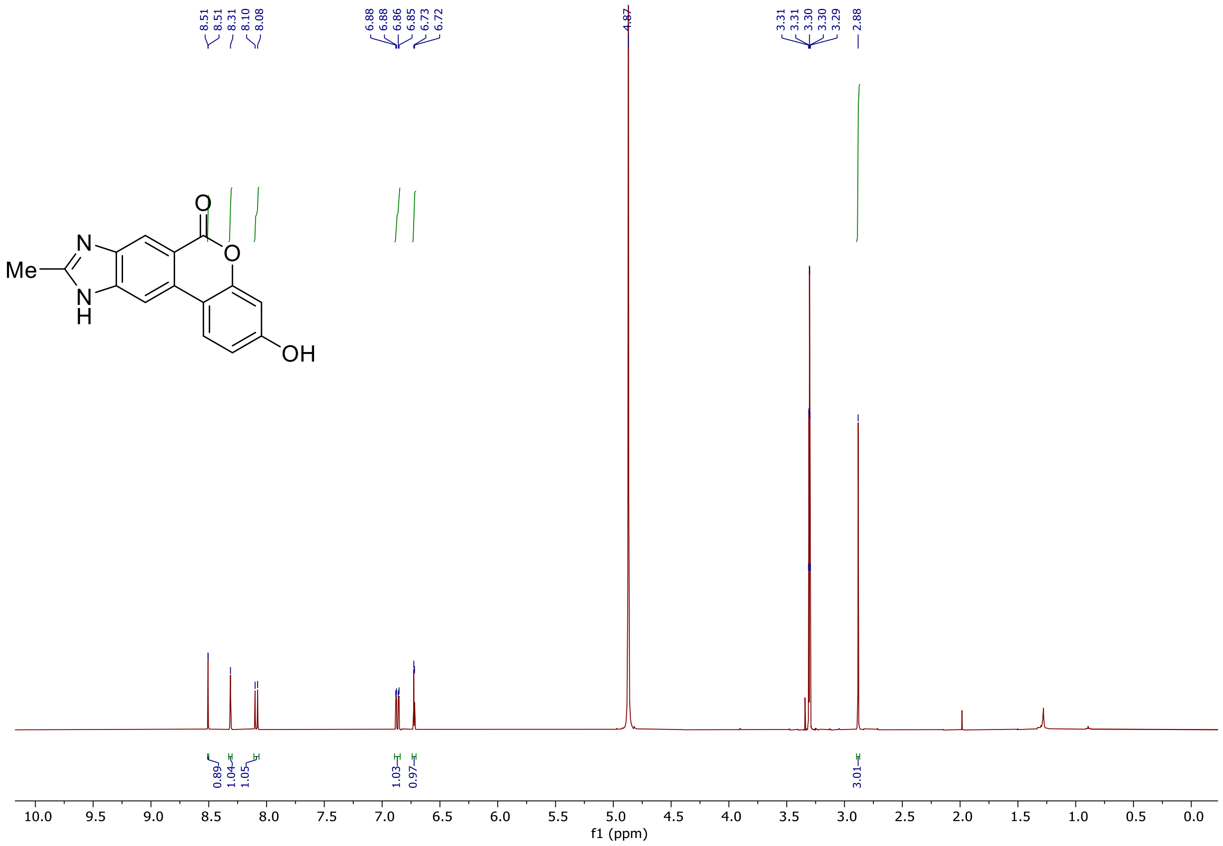

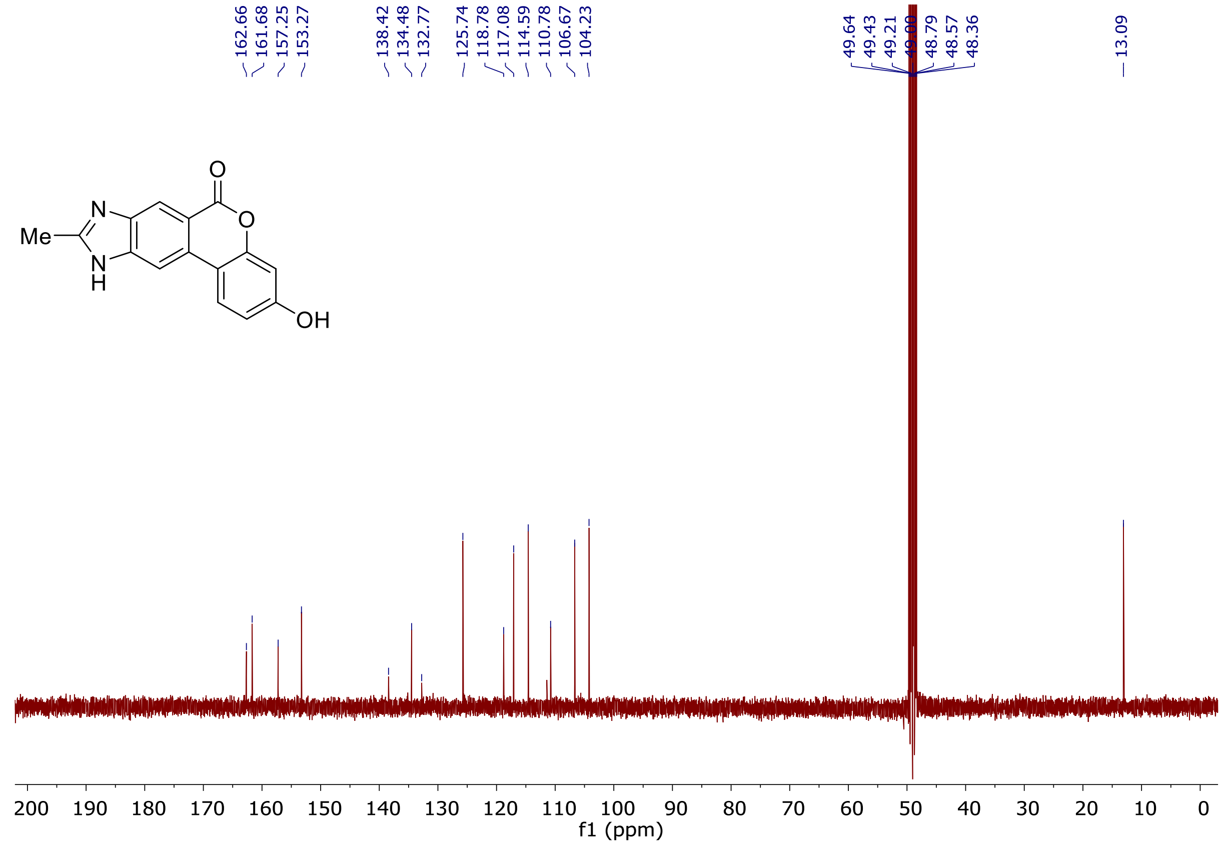

3-Hydroxy-6H-[1,3]dioxolo[4',5':4,5]benzo[1,2-c]chromen-6-one (**142**)

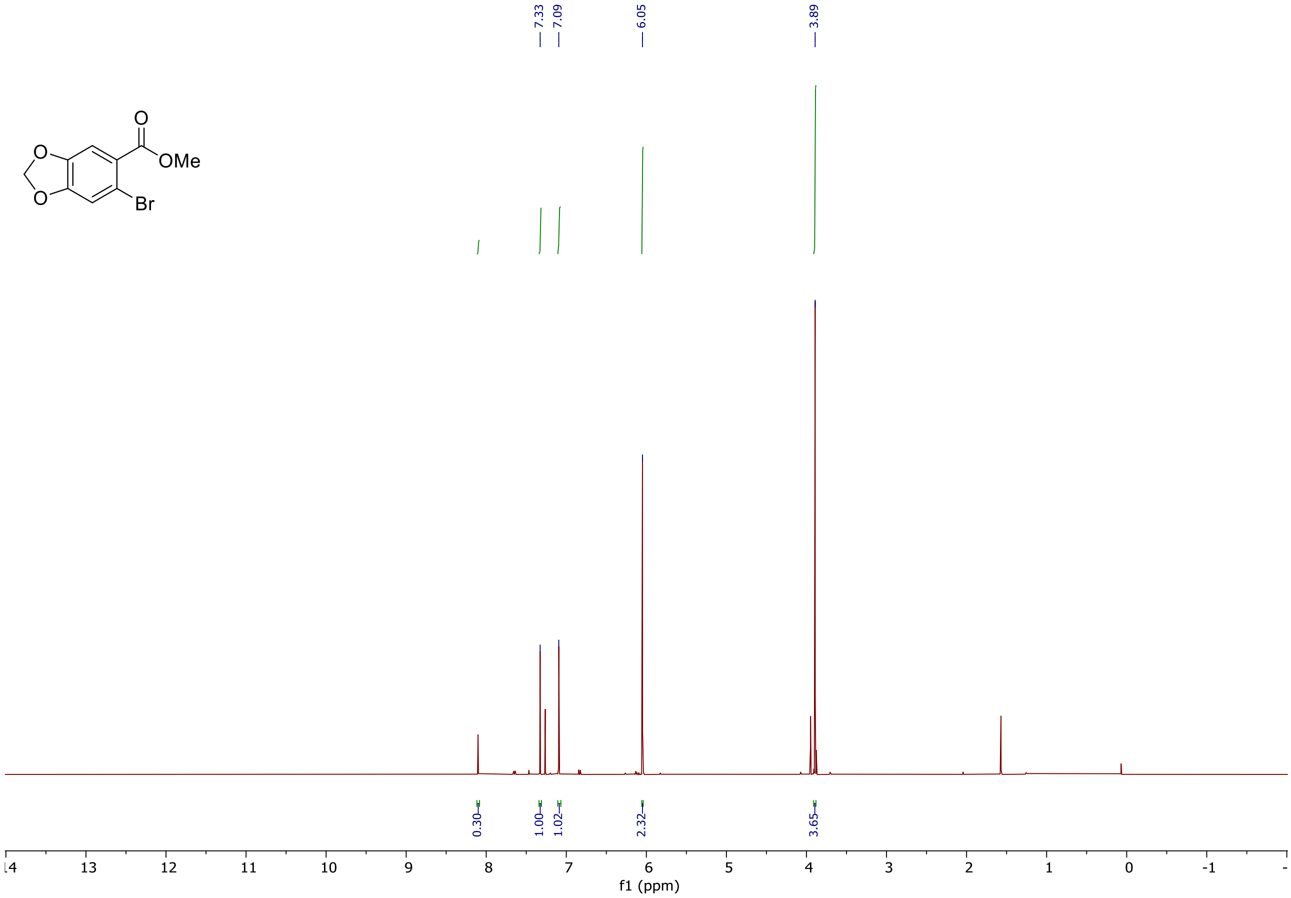

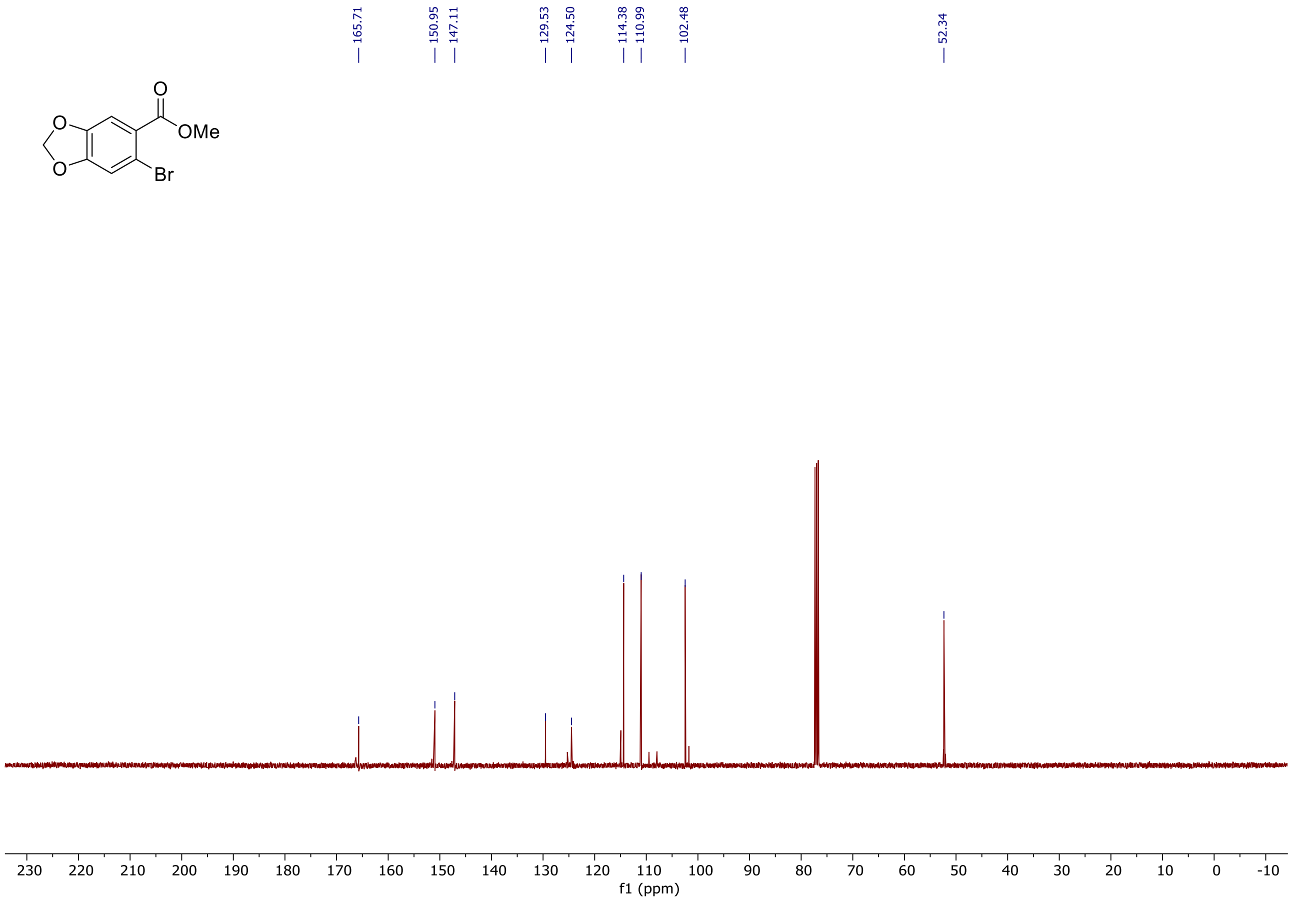

*6-bromobenzo[d][1,3]dioxole-5-carboxylic acid (**143**)*

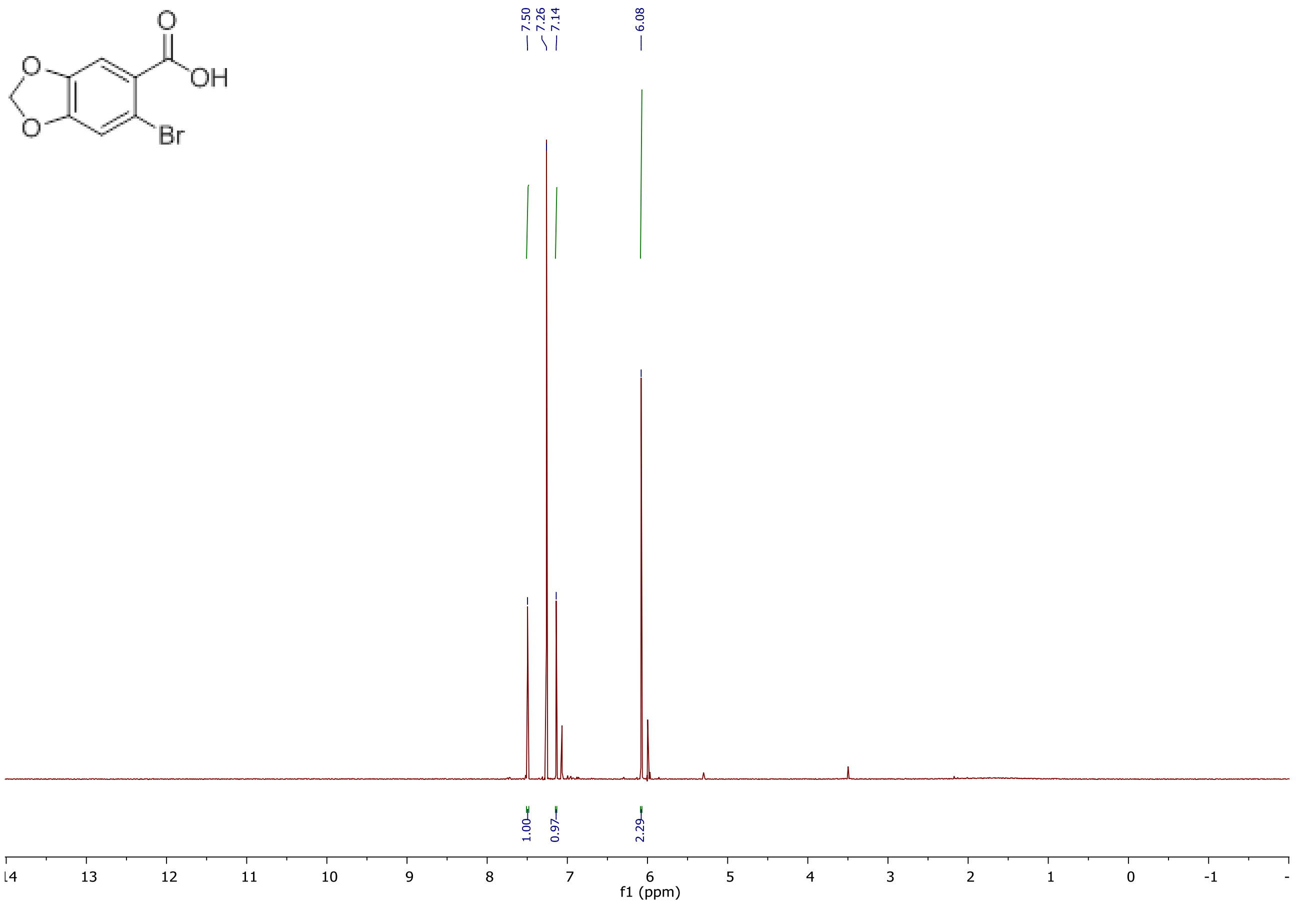

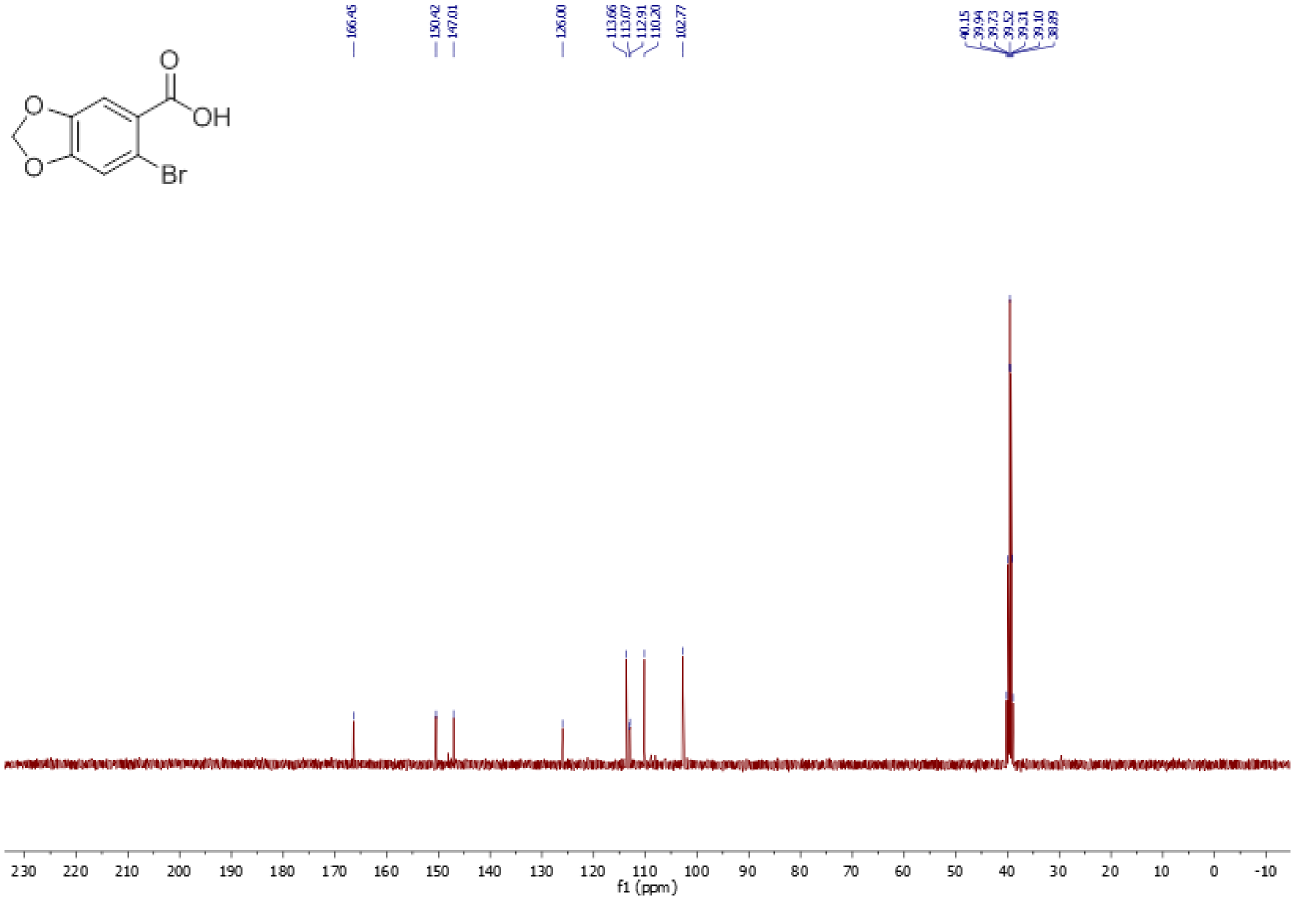

*3-Hydroxy-6H-[1,3]dioxolo[4',5':4,5]benzo[1,2-c]chromen-6-one (**144**)*

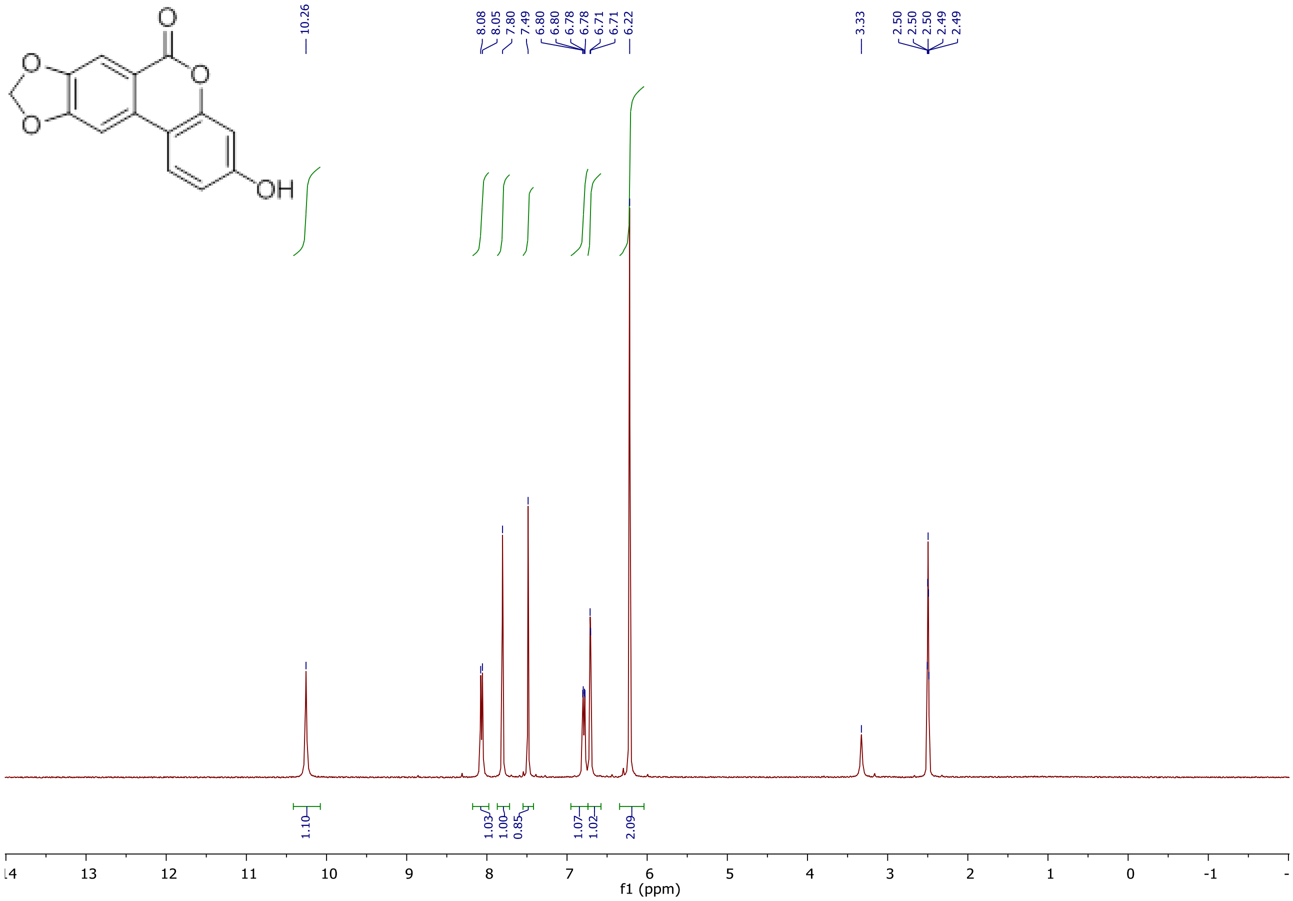

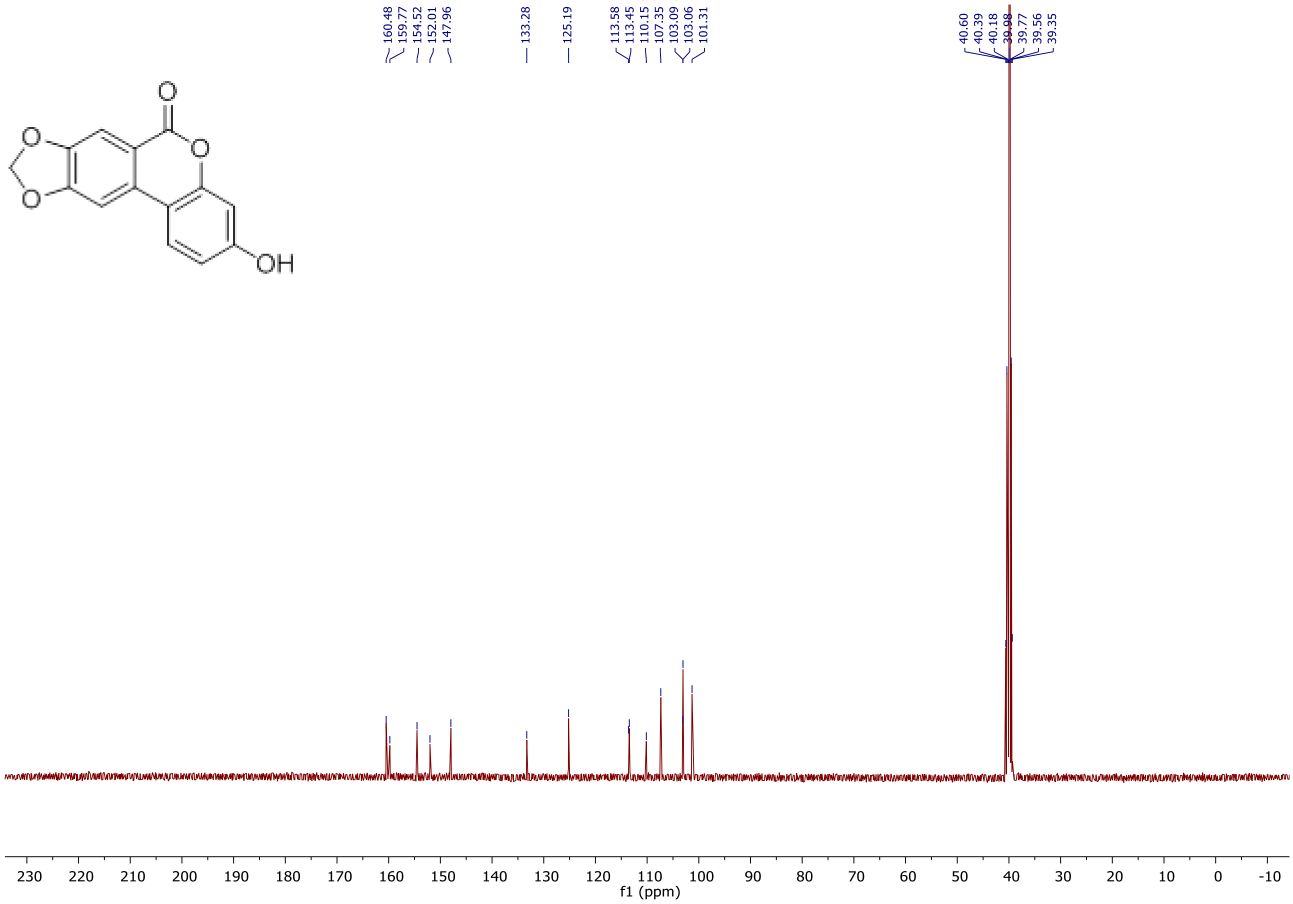

Methyl 6-bromo-2-oxo-2,3-dihydro-1H-benzo[d]imidazole-5-carboxylate (**145**)

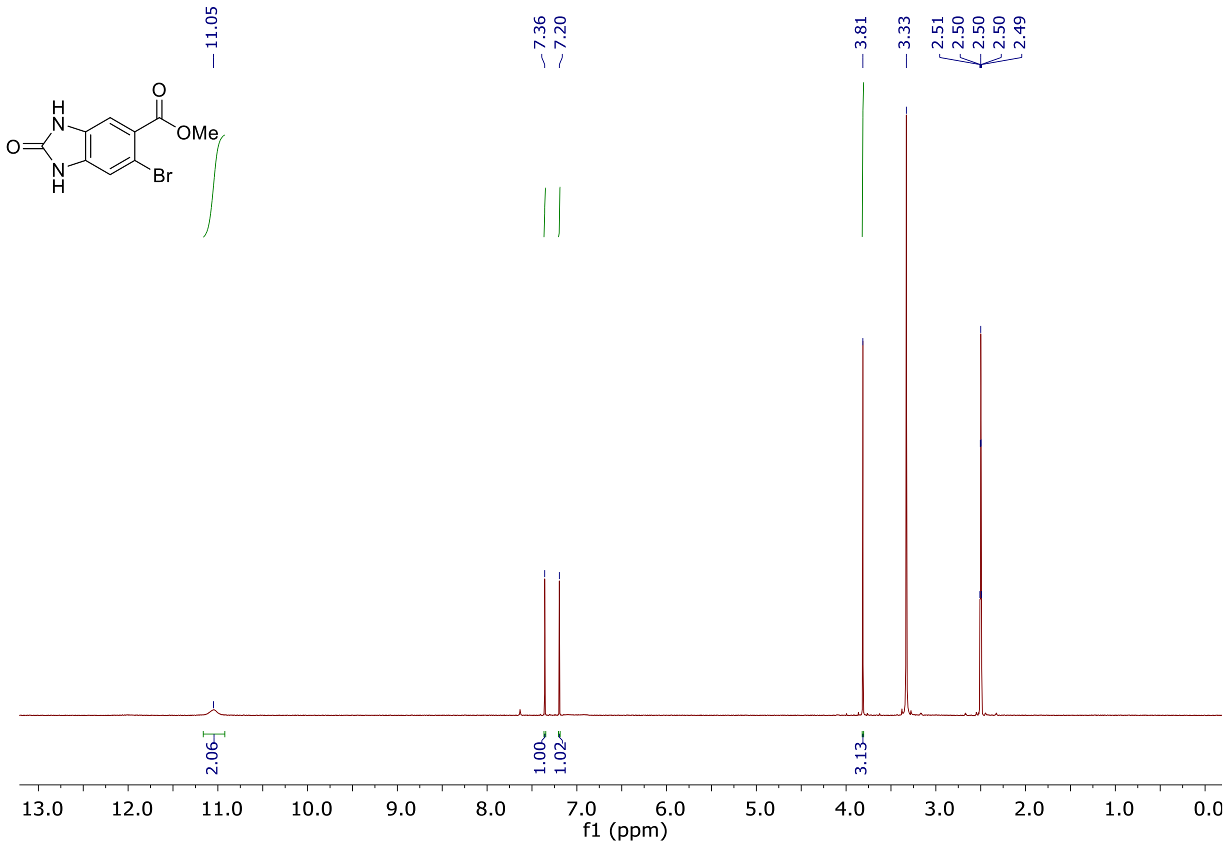

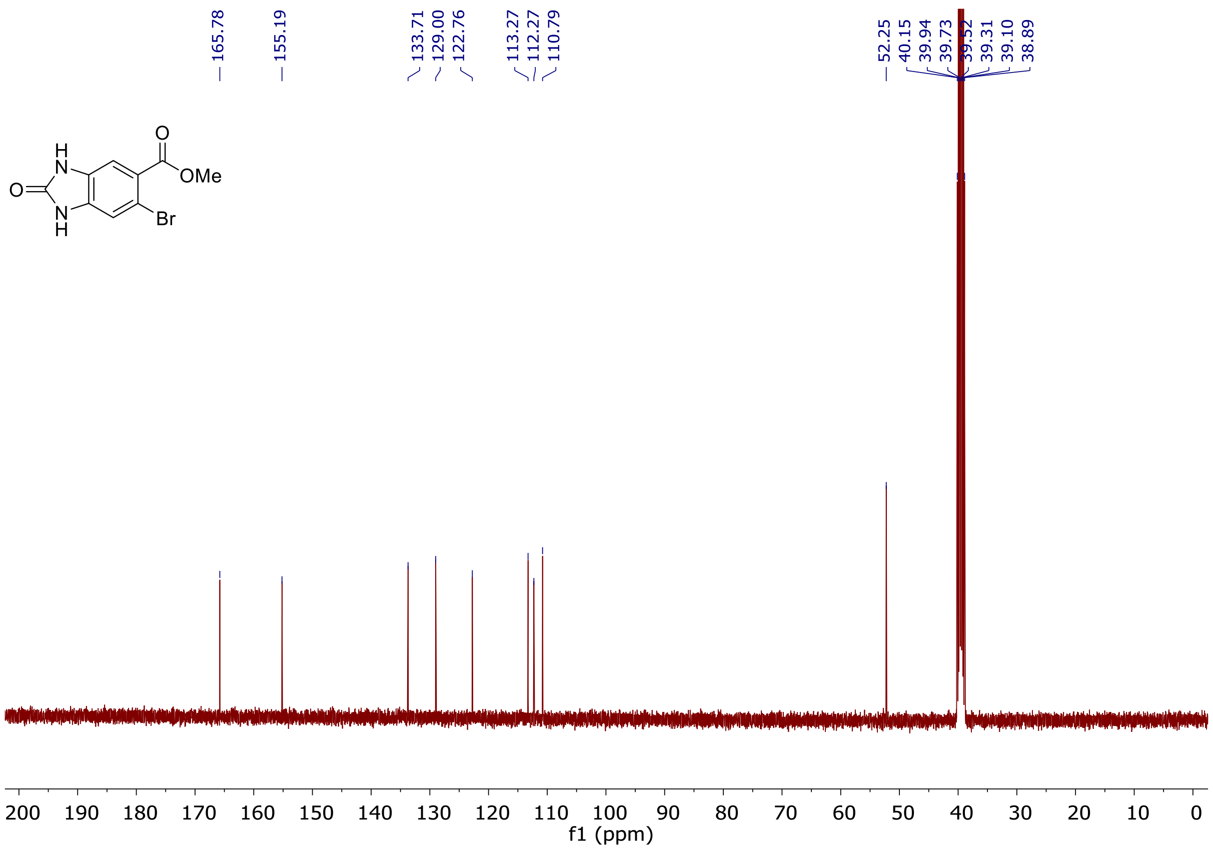

6-Bromo-2-oxo-2,3-dihydro-1H-benzo[d]imidazole-5-carboxylic acid (**149**)

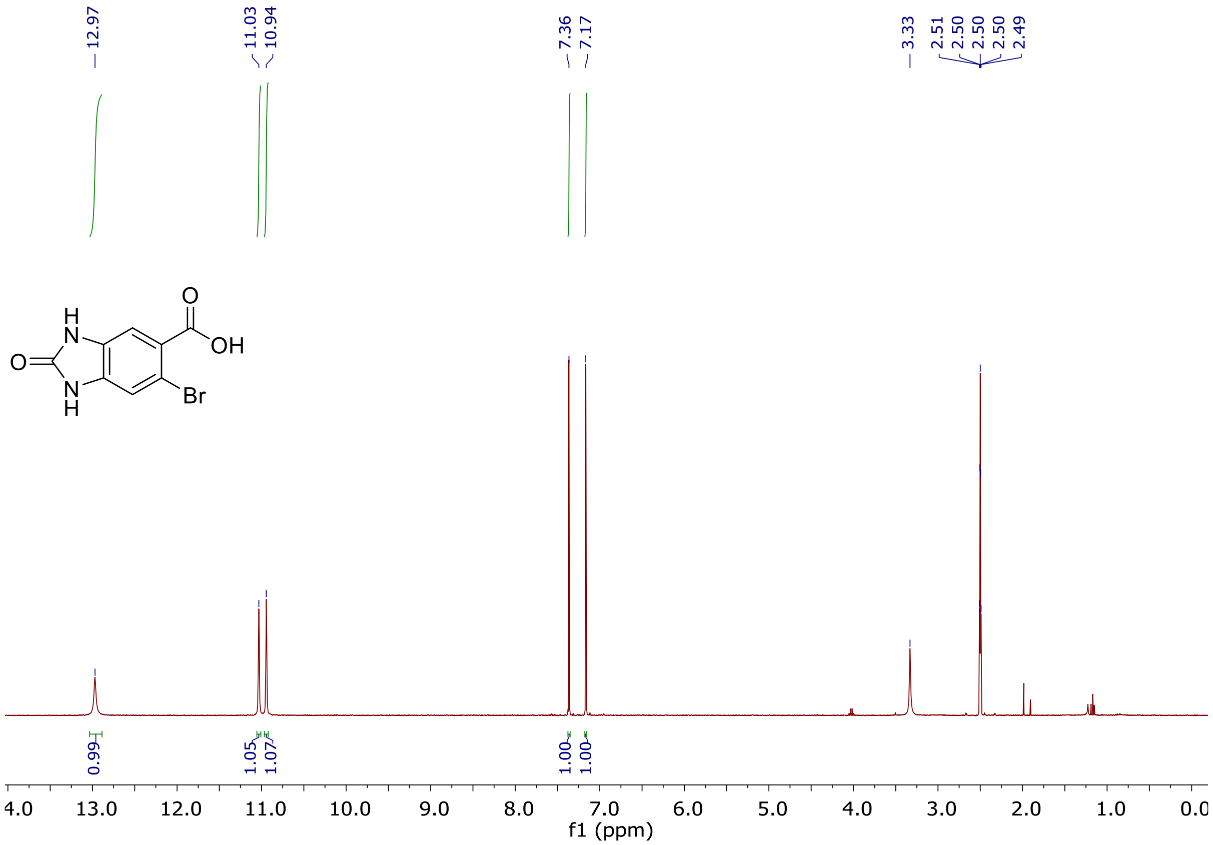

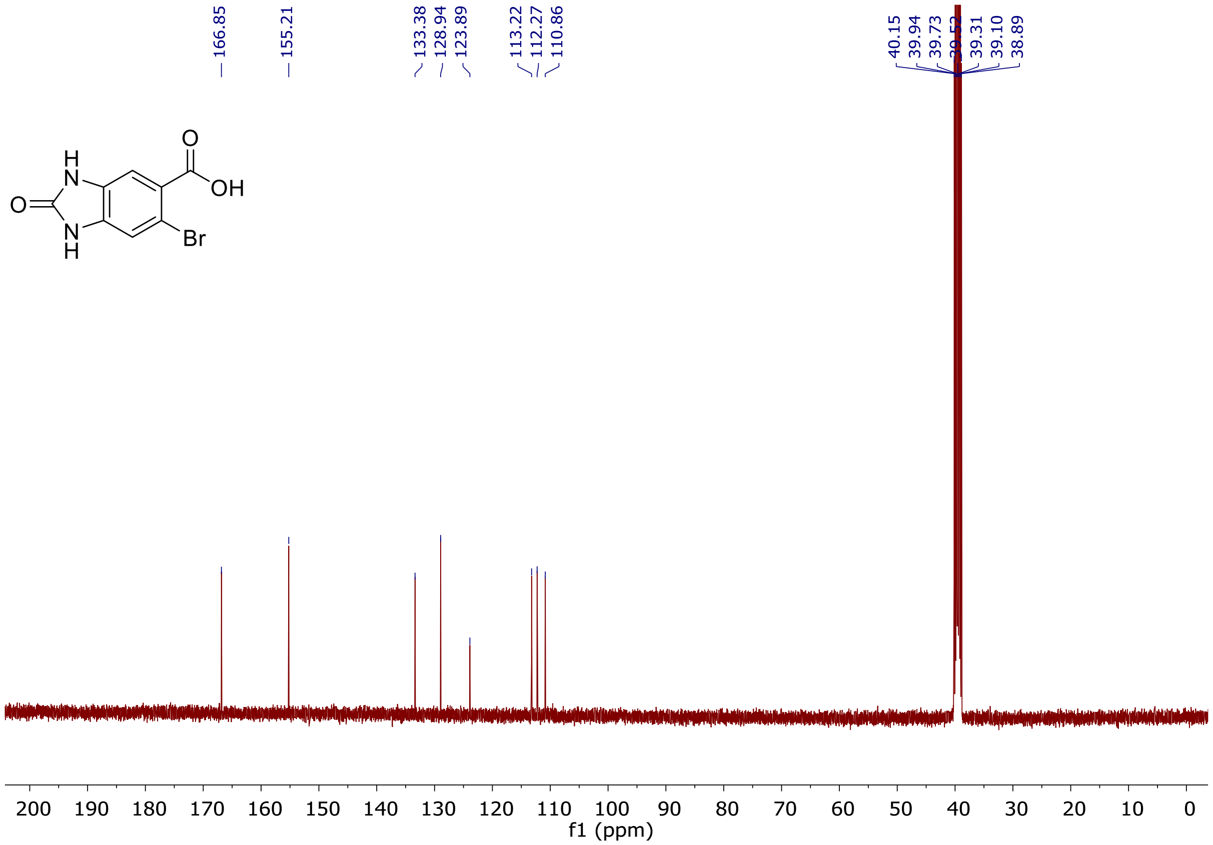

3-Hydroxy-8,10-dihydrobenzo[3,4]isochromeno[6,7-d]imidazole-6,9-dione (**150**)

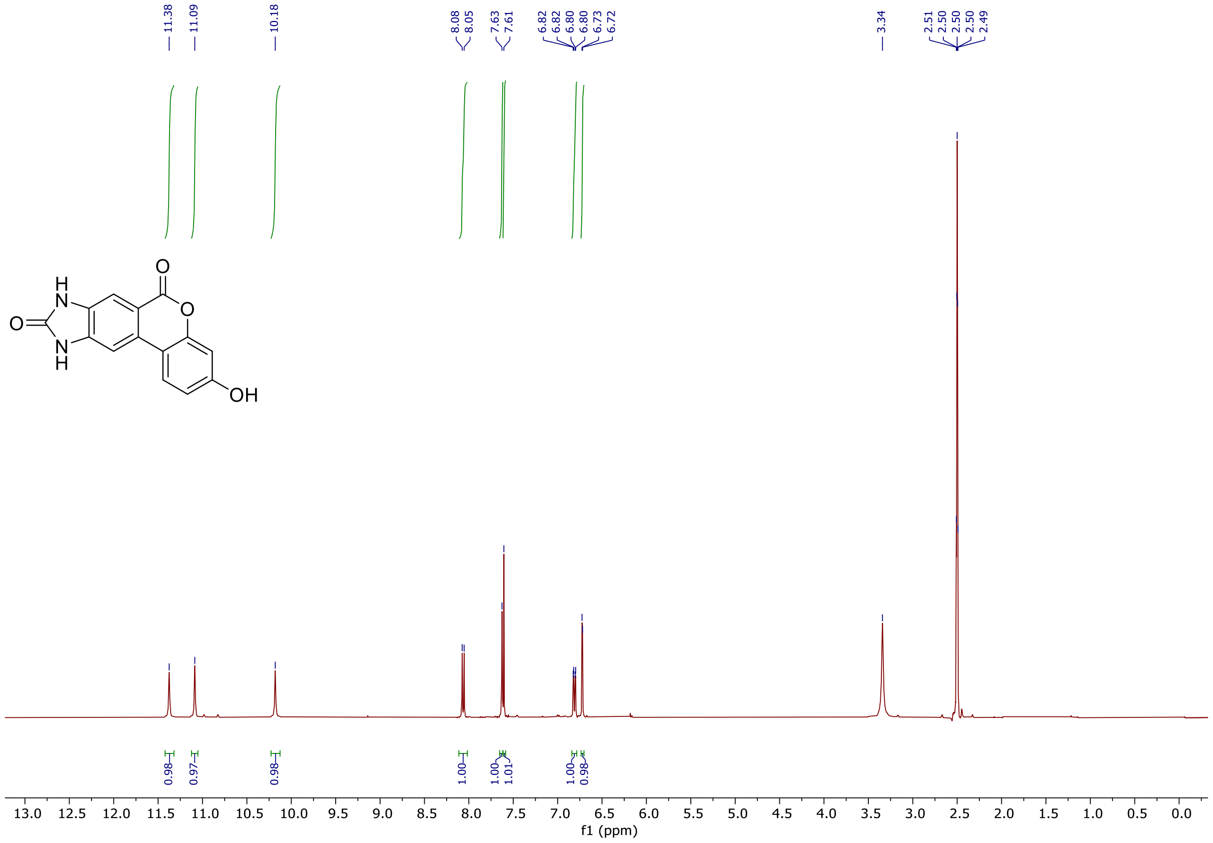

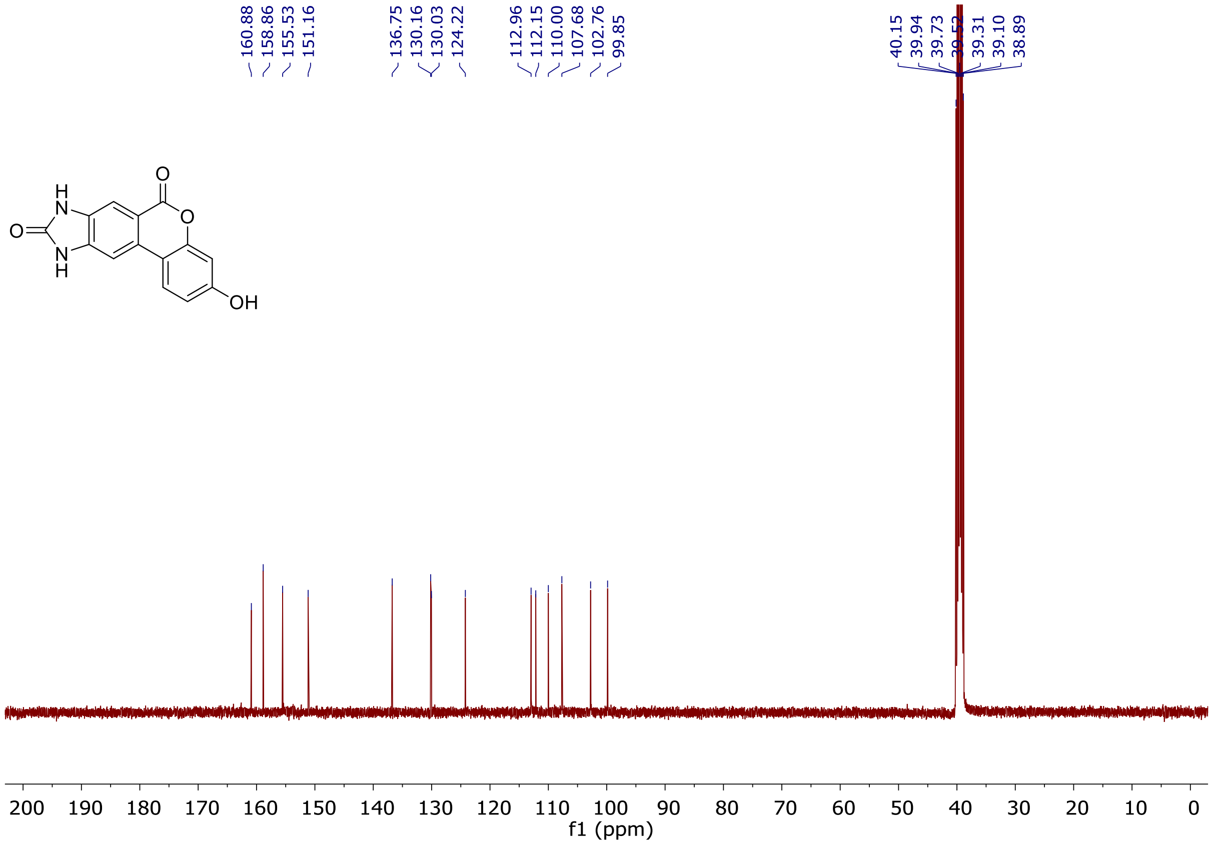

Methyl 6-bromo-1H-benzo[d]imidazole-5-carboxylate (**146**)

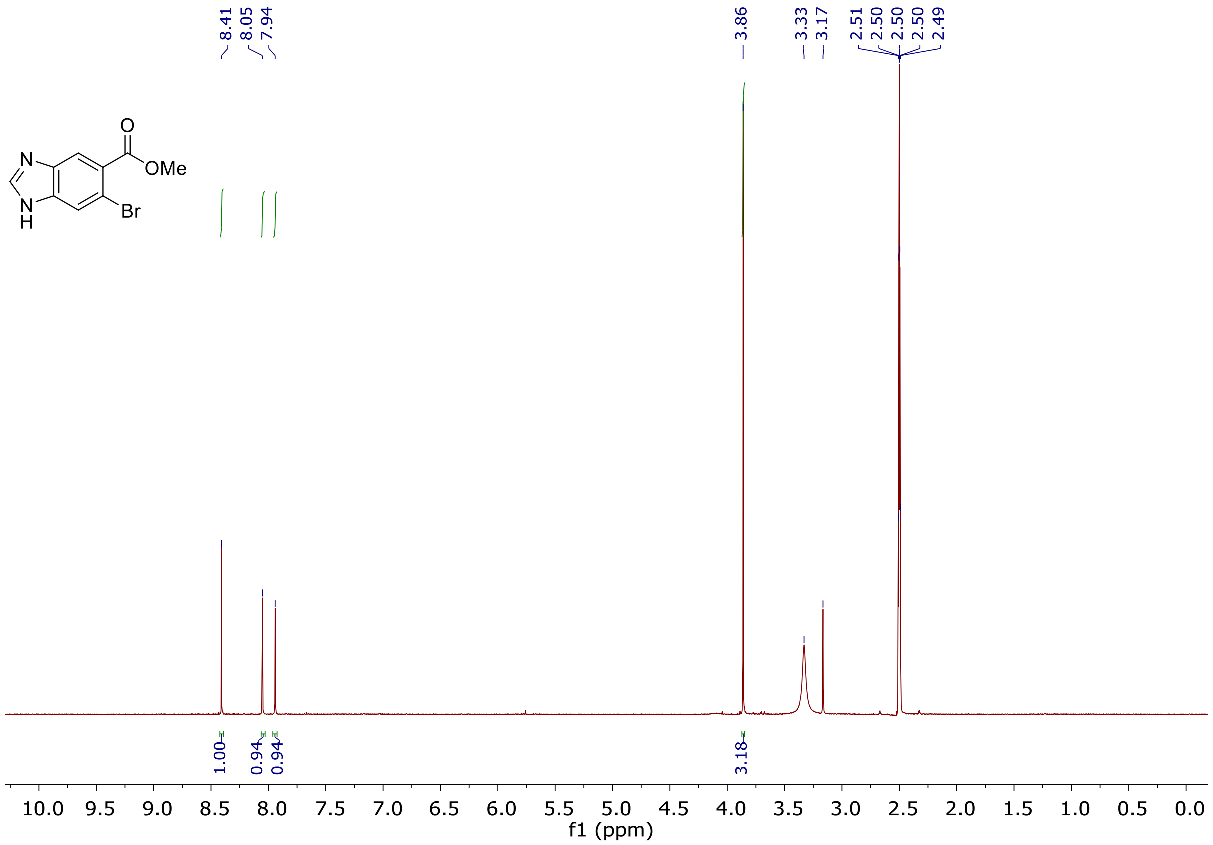

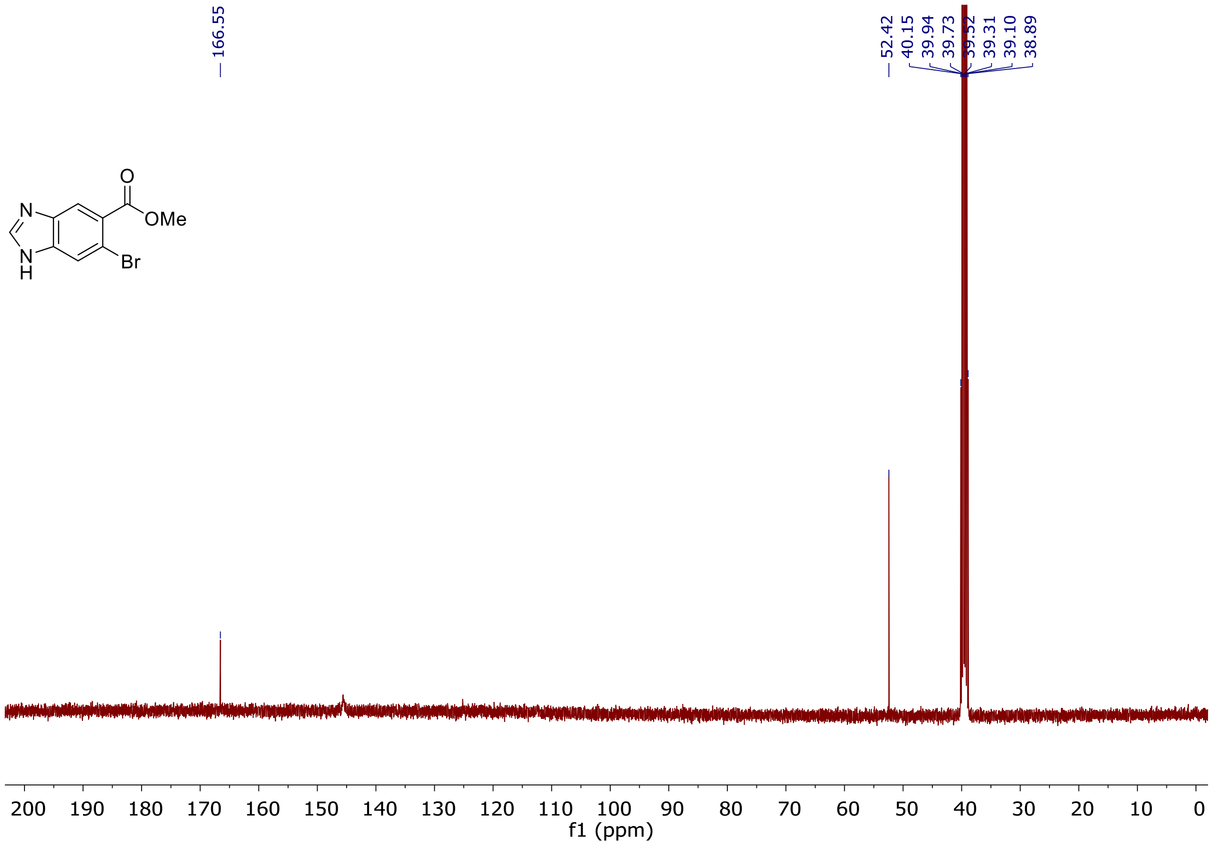

Methyl 6-(2,4-dimethoxyphenyl)-1H-benzo[d]imidazole-5-carboxylate (**151**)

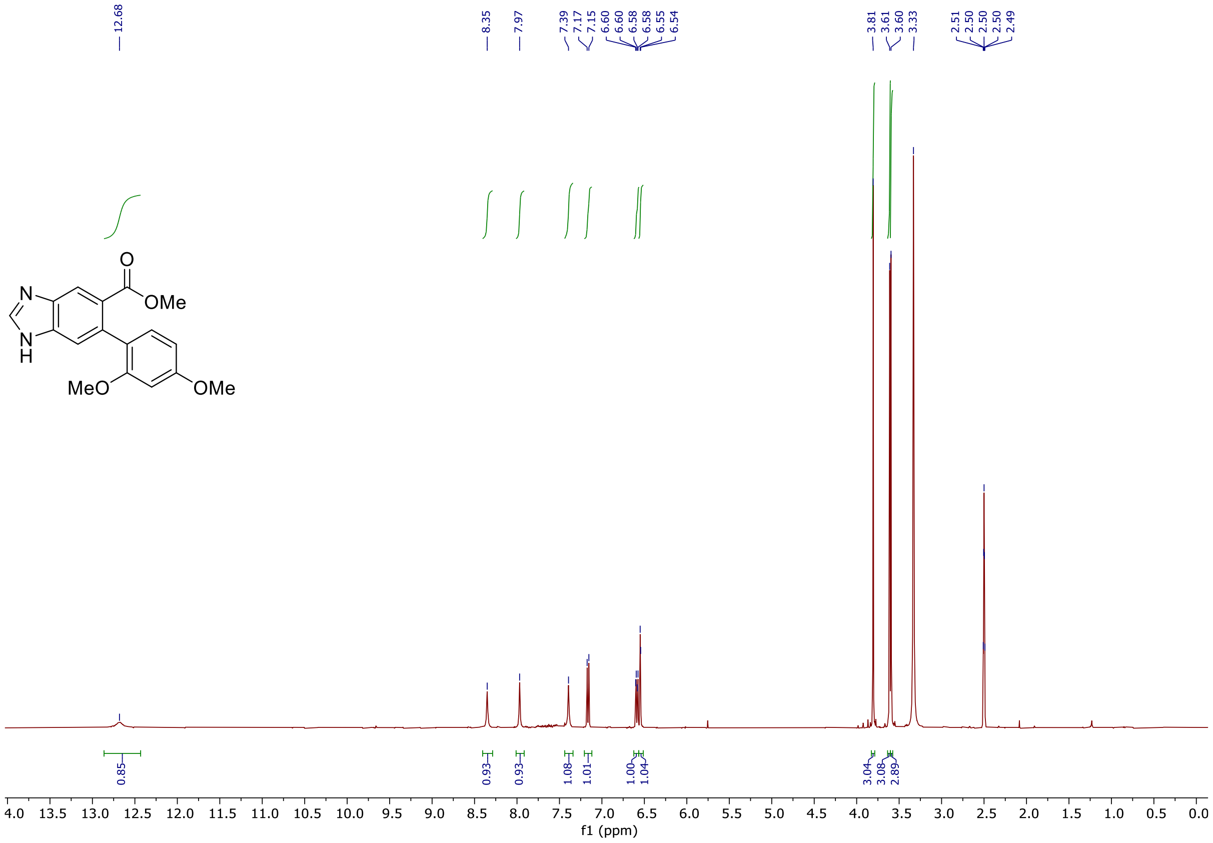

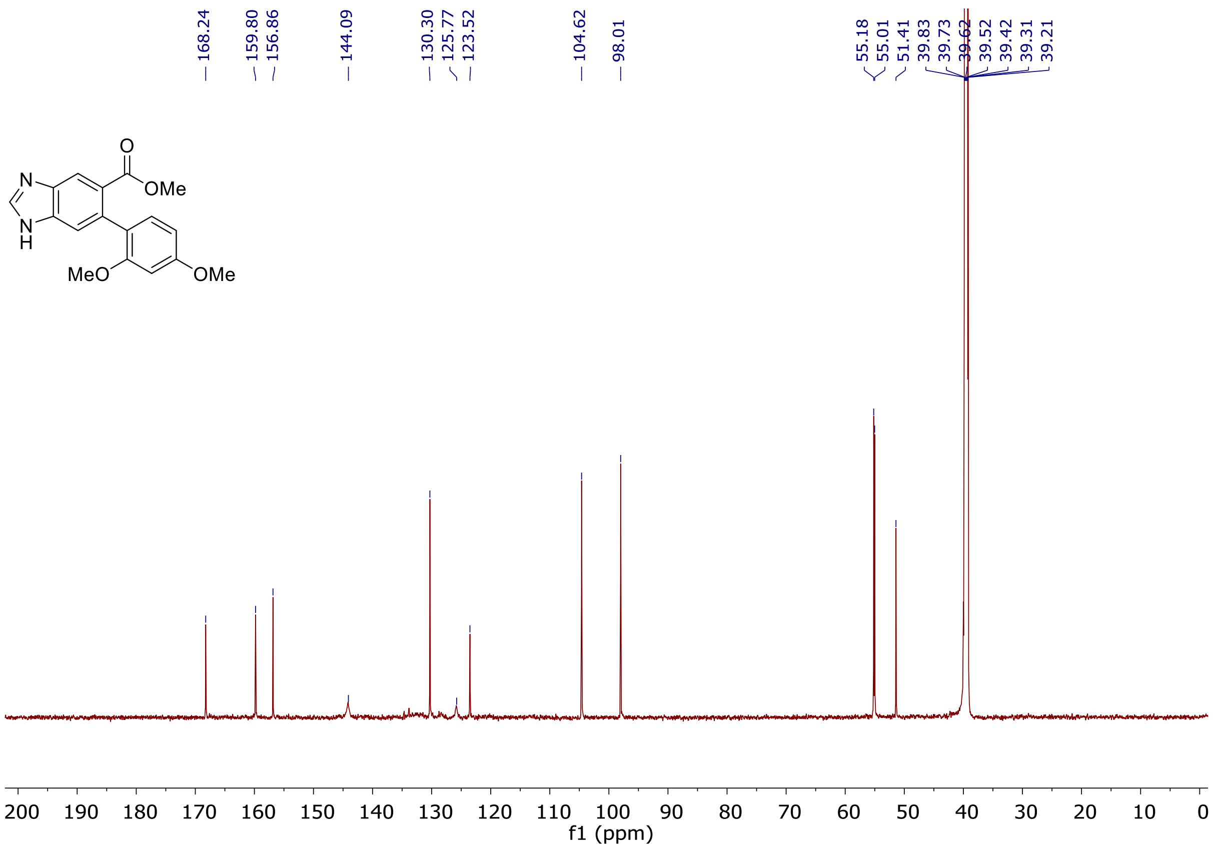

3-Hydroxybenzo[3,4]isochromeno[6,7-d]imidazol-6(10H)-one (**153**)

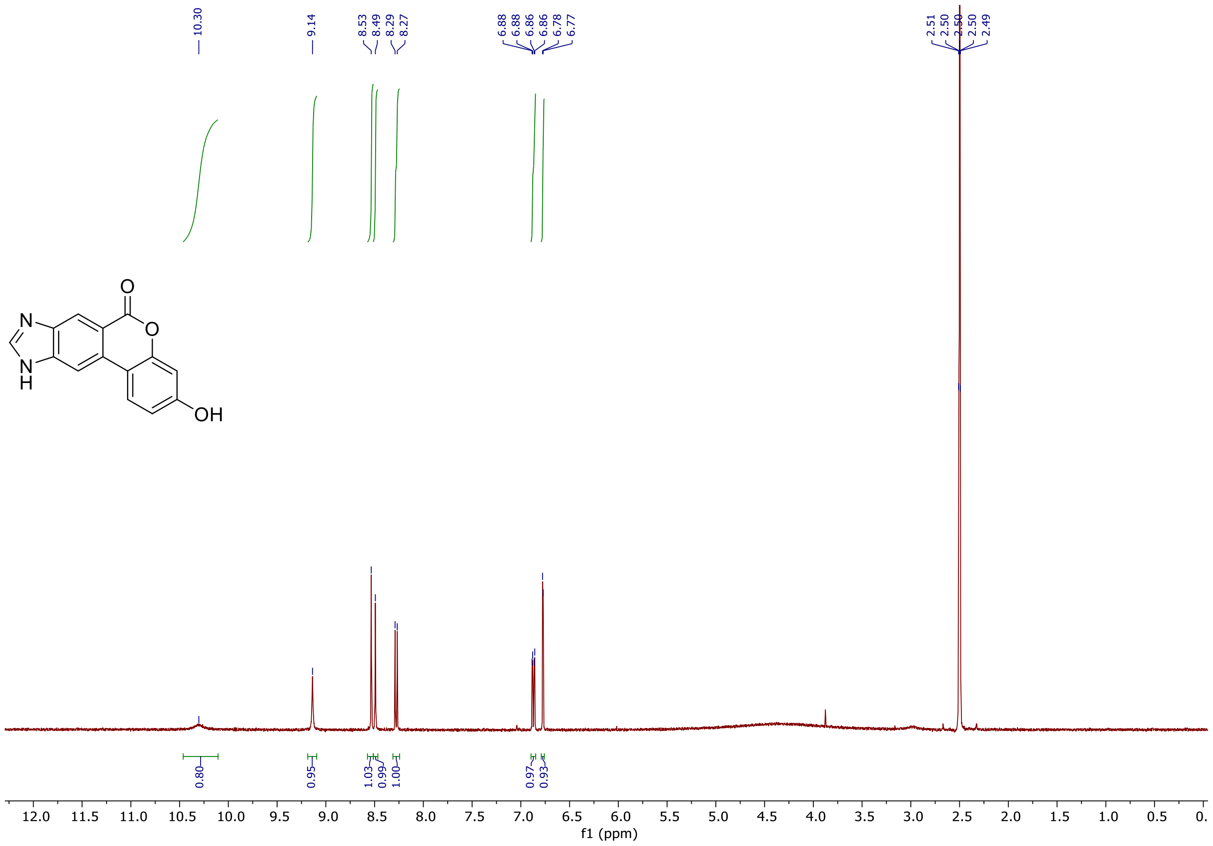

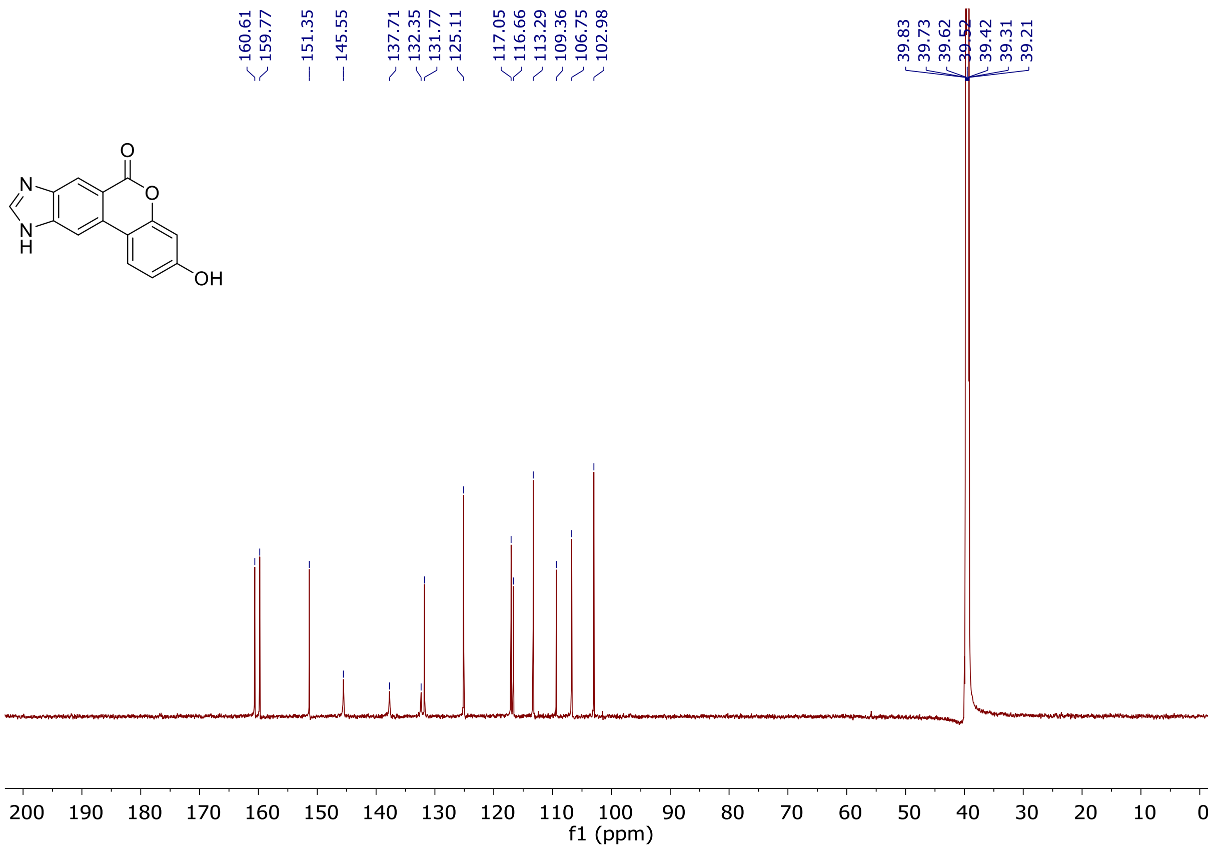

Methyl 6-bromo-1H-benzo[d][1,2,3]triazole-5-carboxylate (**147**)

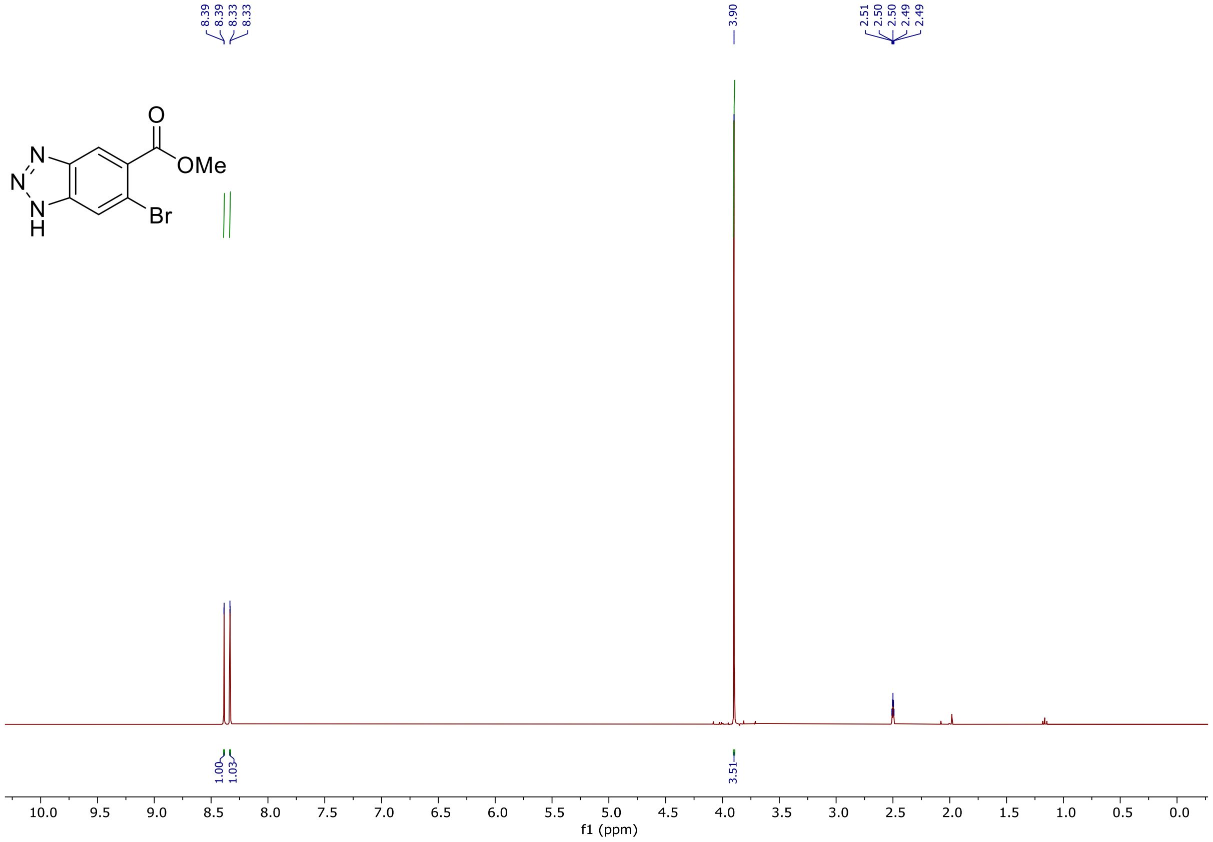

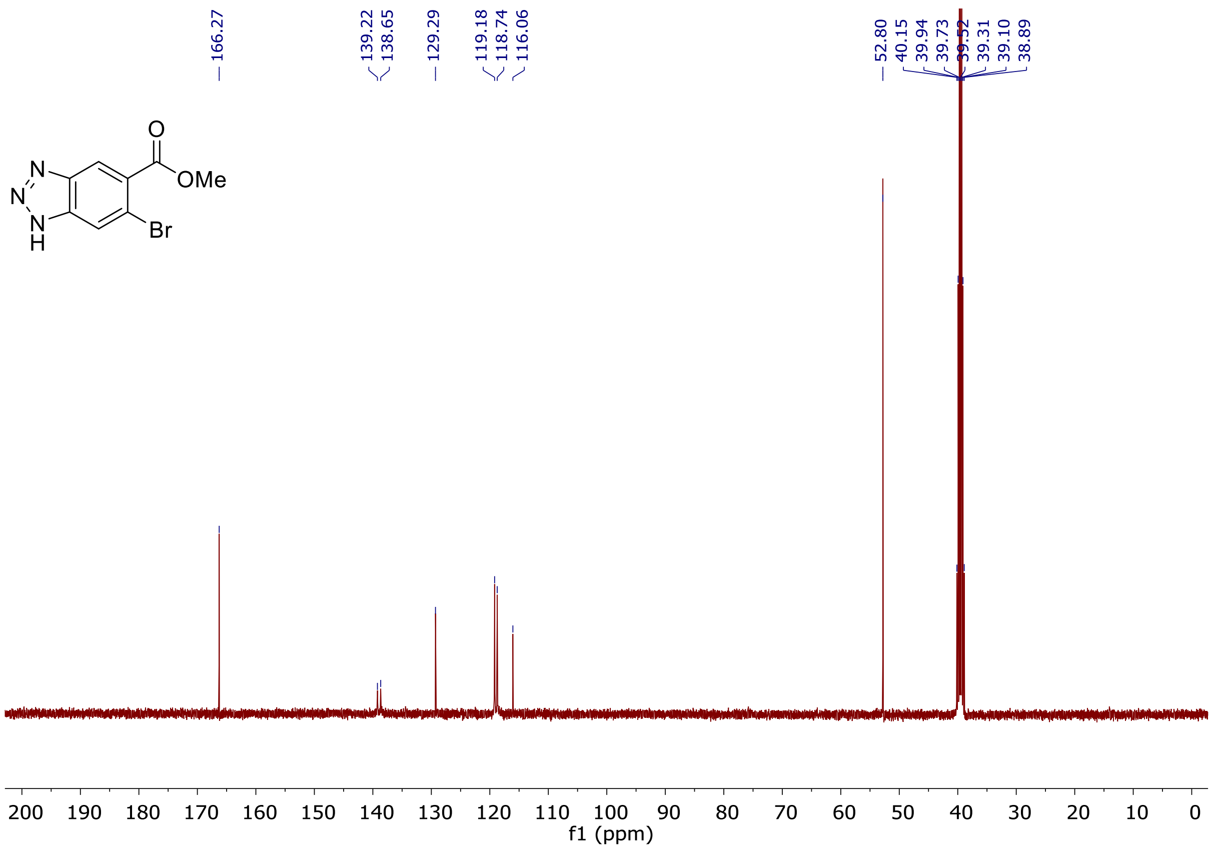

Methyl 1-acetyl-6-bromo-1H-benzo[d][1,2,3]triazole-5-carboxylate + Methyl 1-acetyl-5-bromo-1H-benzo[d][1,2,3]triazole-6-carboxylate (**148**)

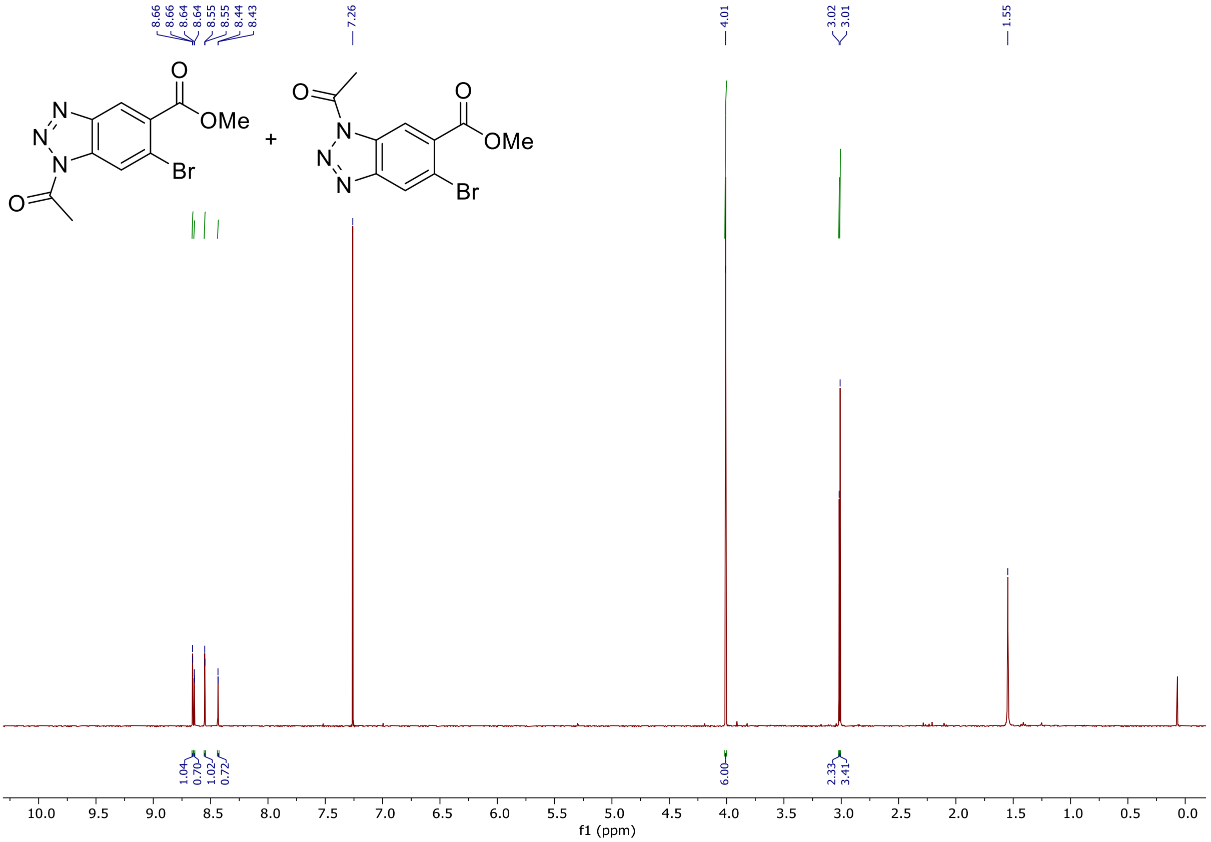

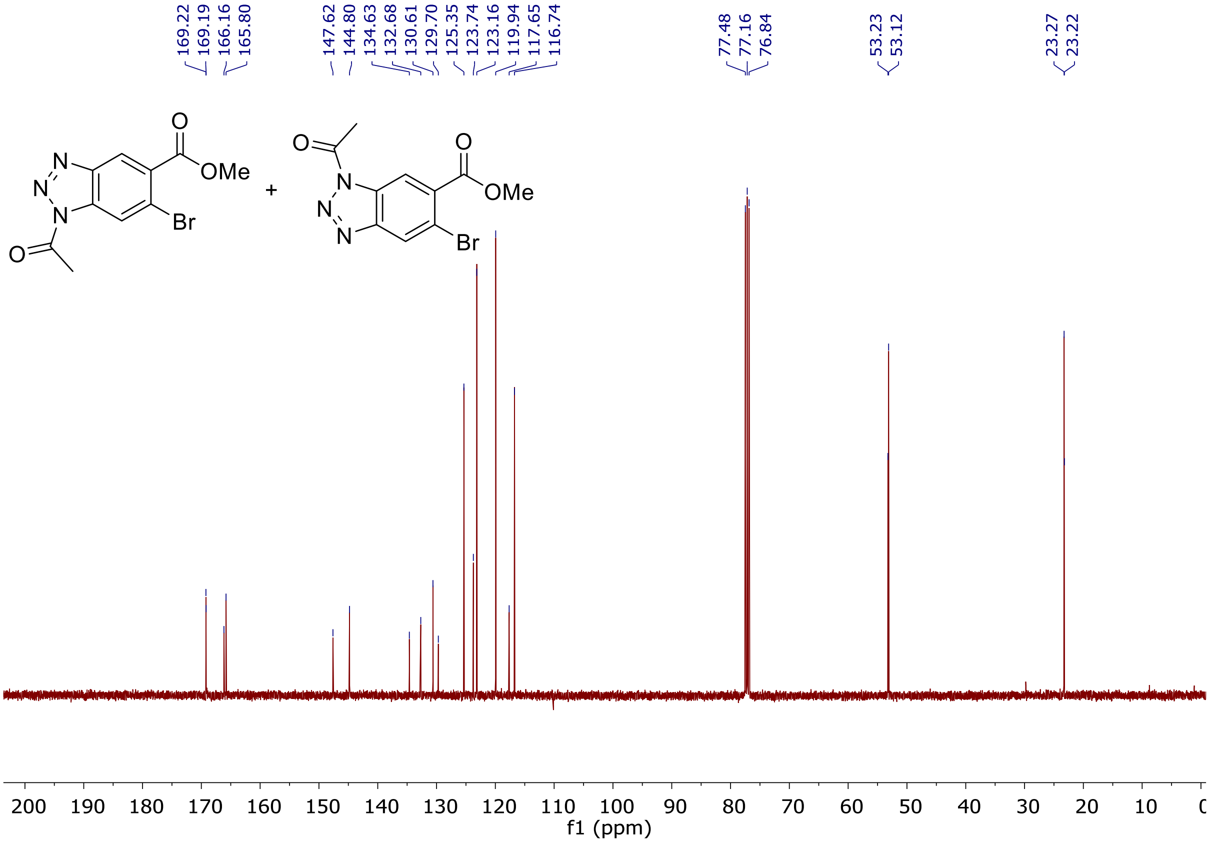

Methyl 6-(2,4-dimethoxyphenyl)-1H-benzo[d][1,2,3]triazole-5-carboxylate (**152**)

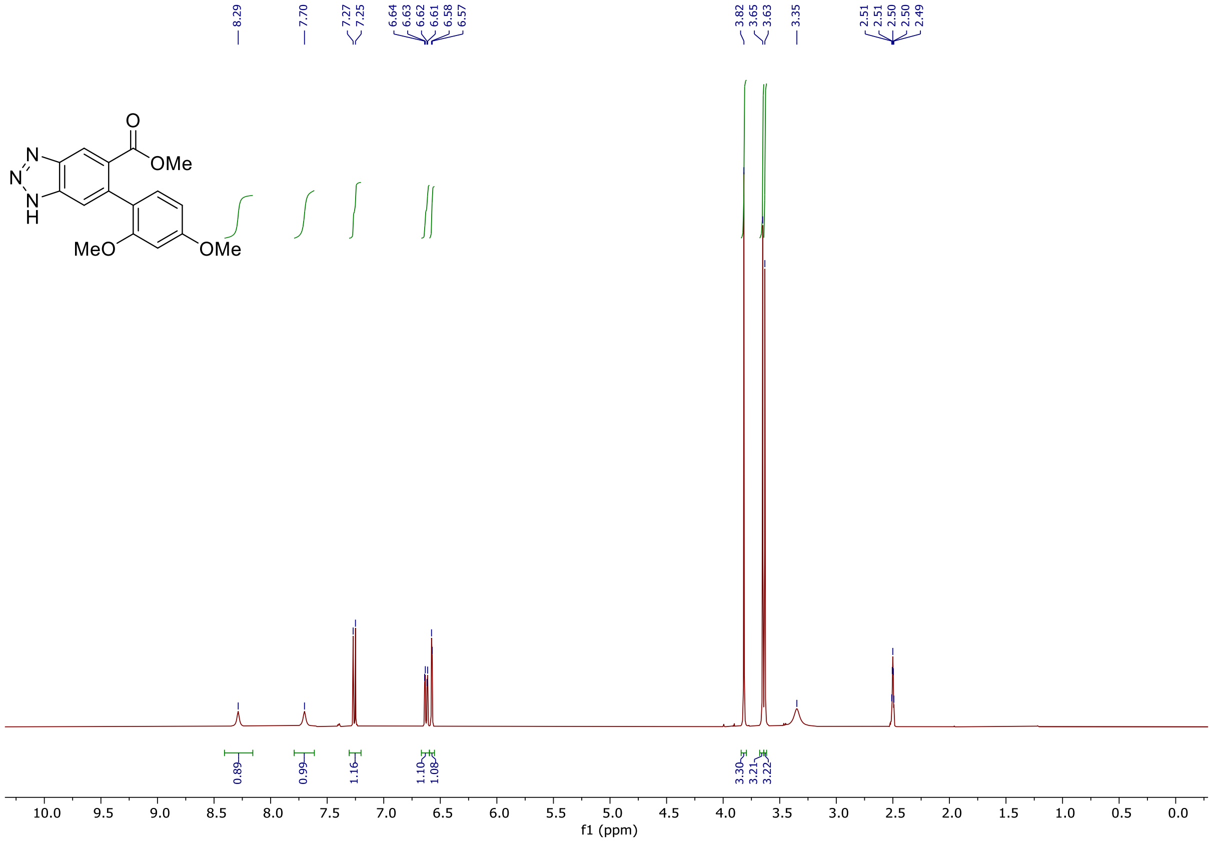

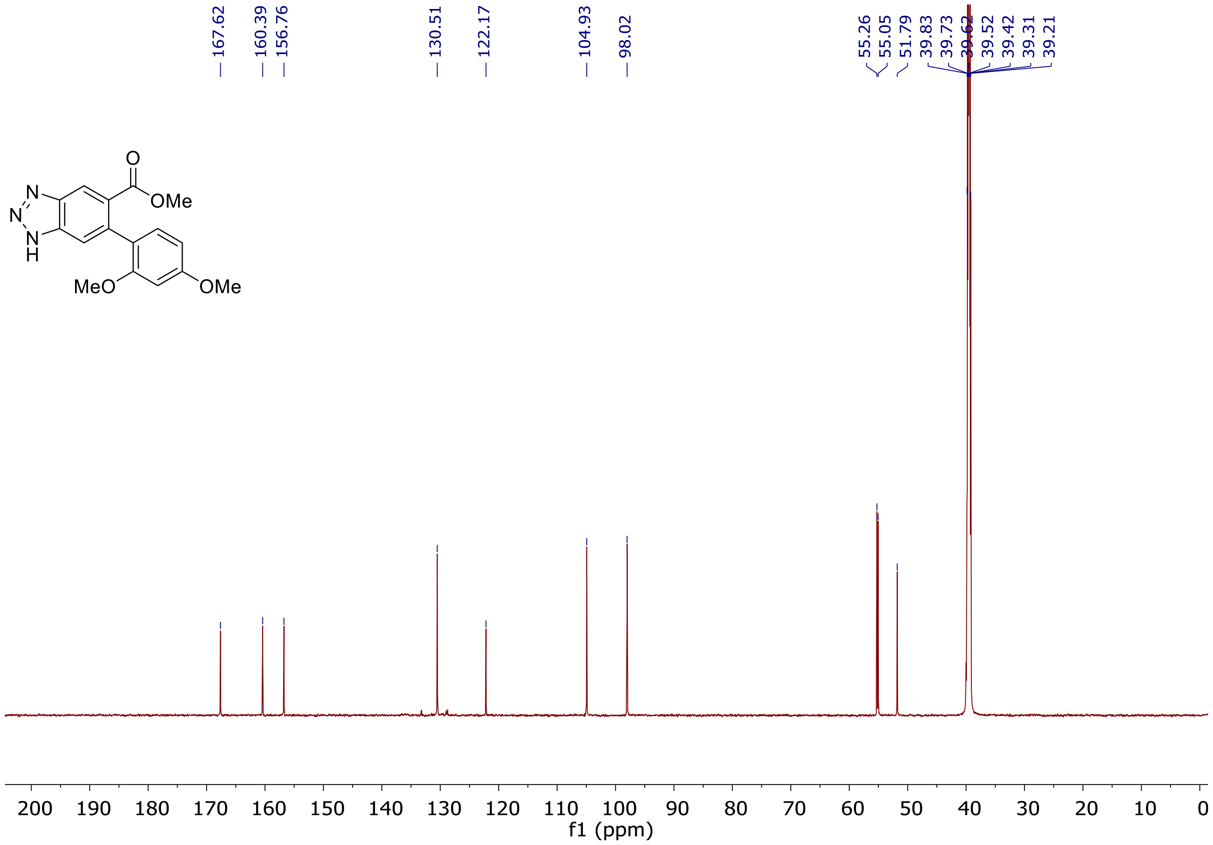

3-Hydroxybenzo[3,4]isochromeno[6,7-d][1,2,3]triazol-6(10H)-one (**154**)

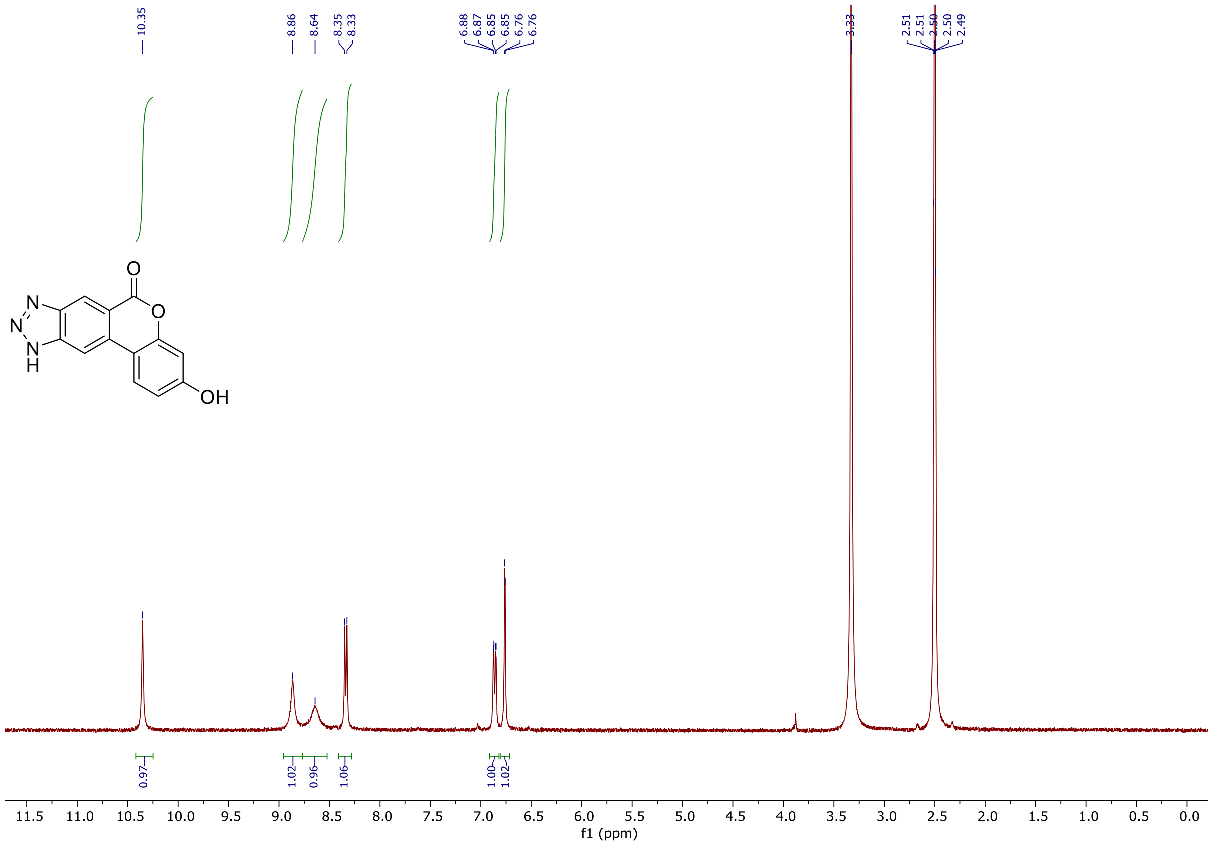

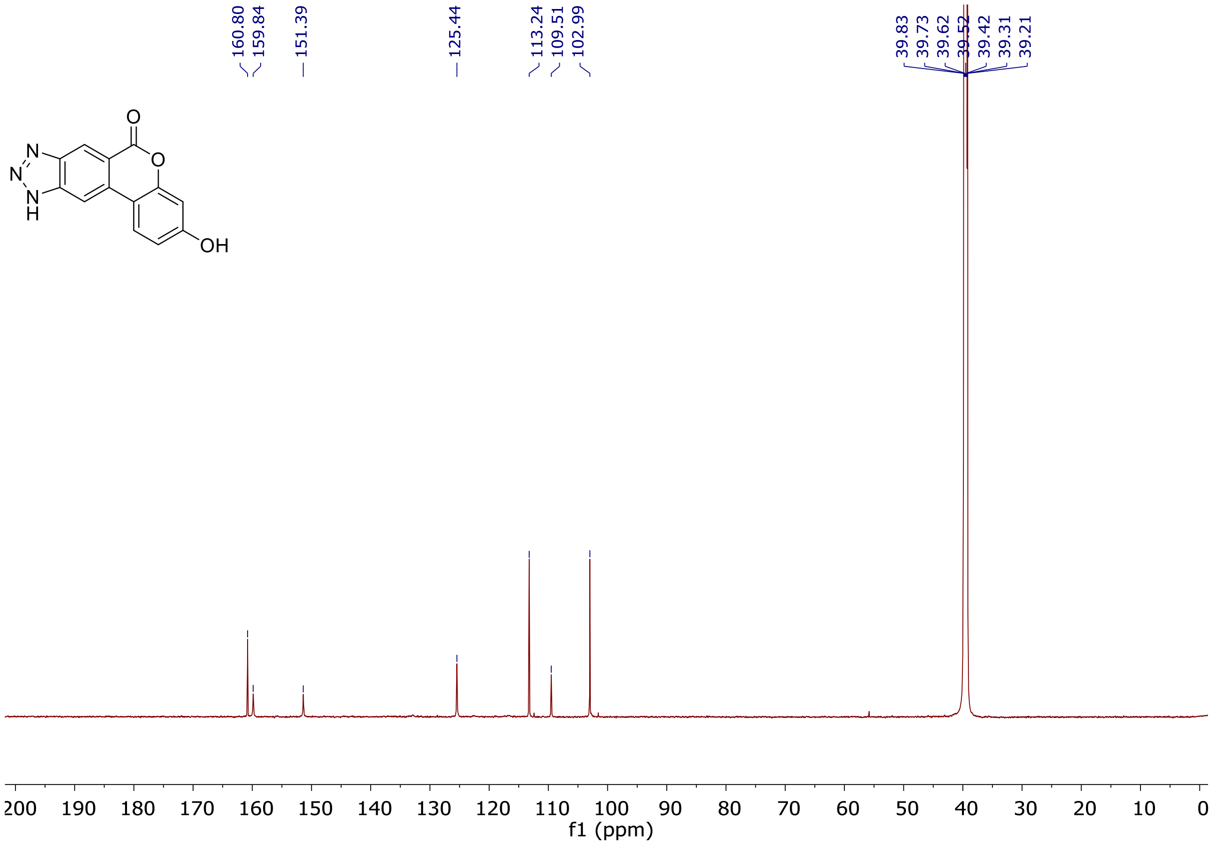

Methyl 2-bromo-5-methoxybenzoate (**156**)

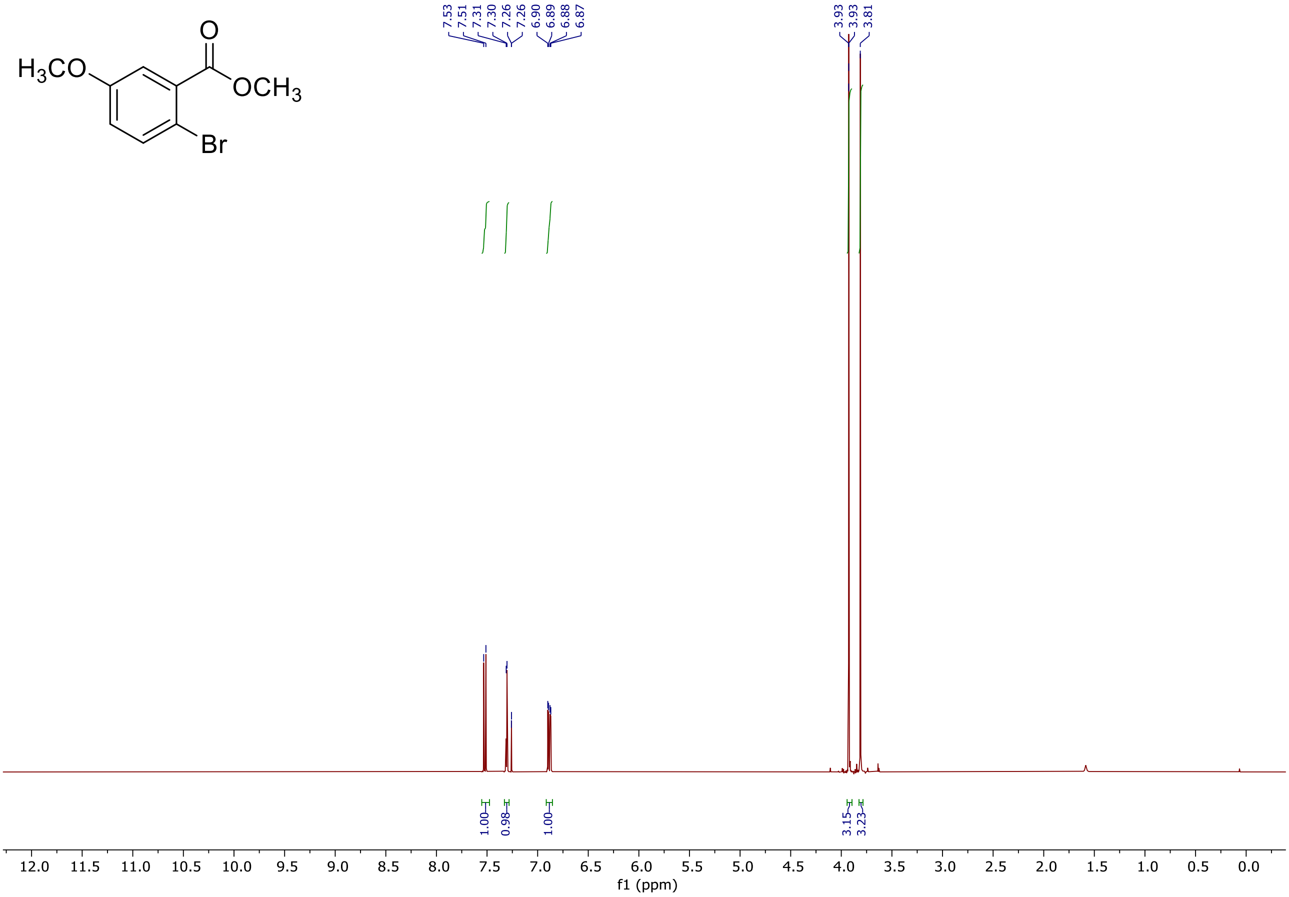

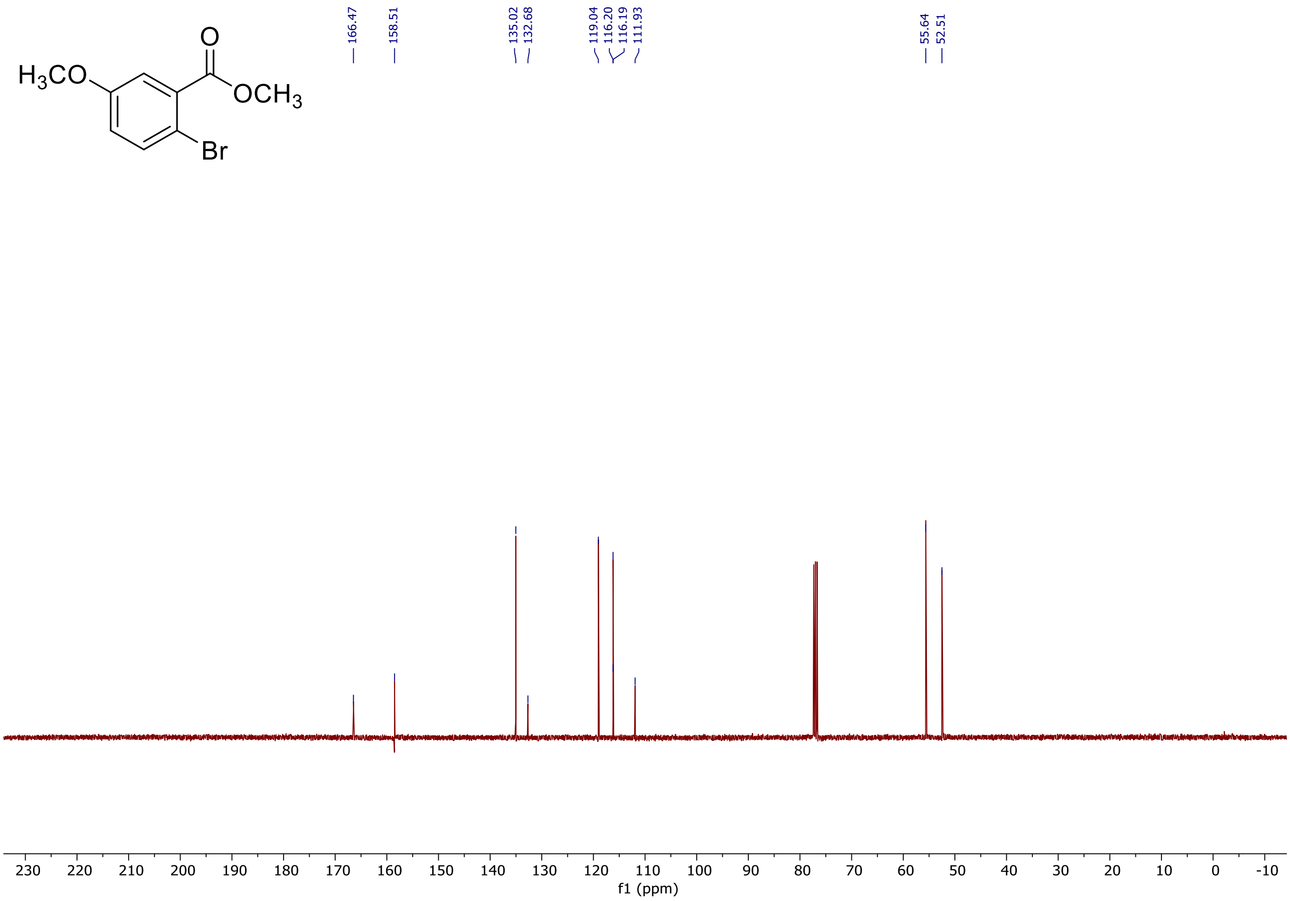

Methyl 3',4,4'-trimethoxy-[1,1'-biphenyl]-2-carboxylate (**158**)

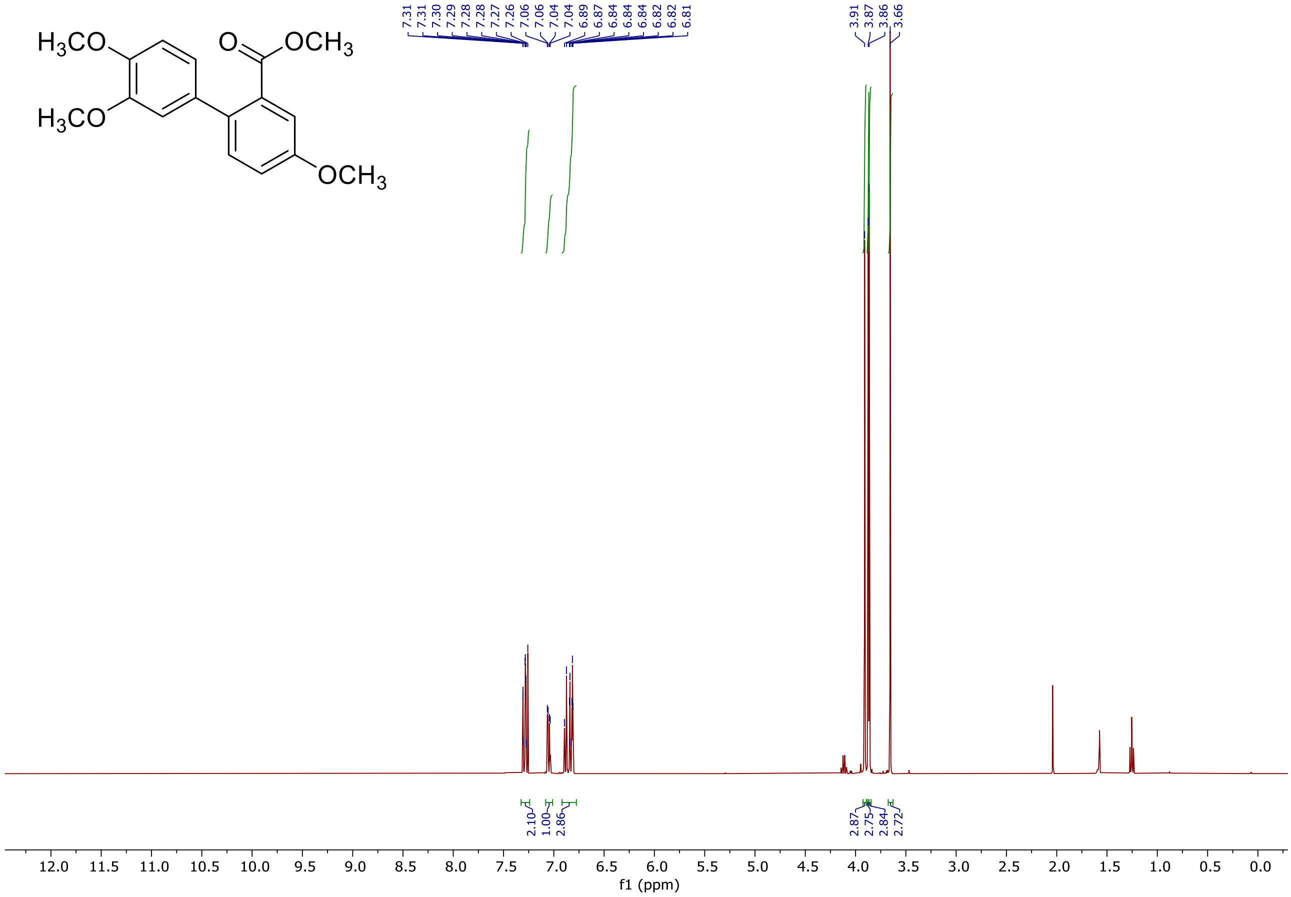

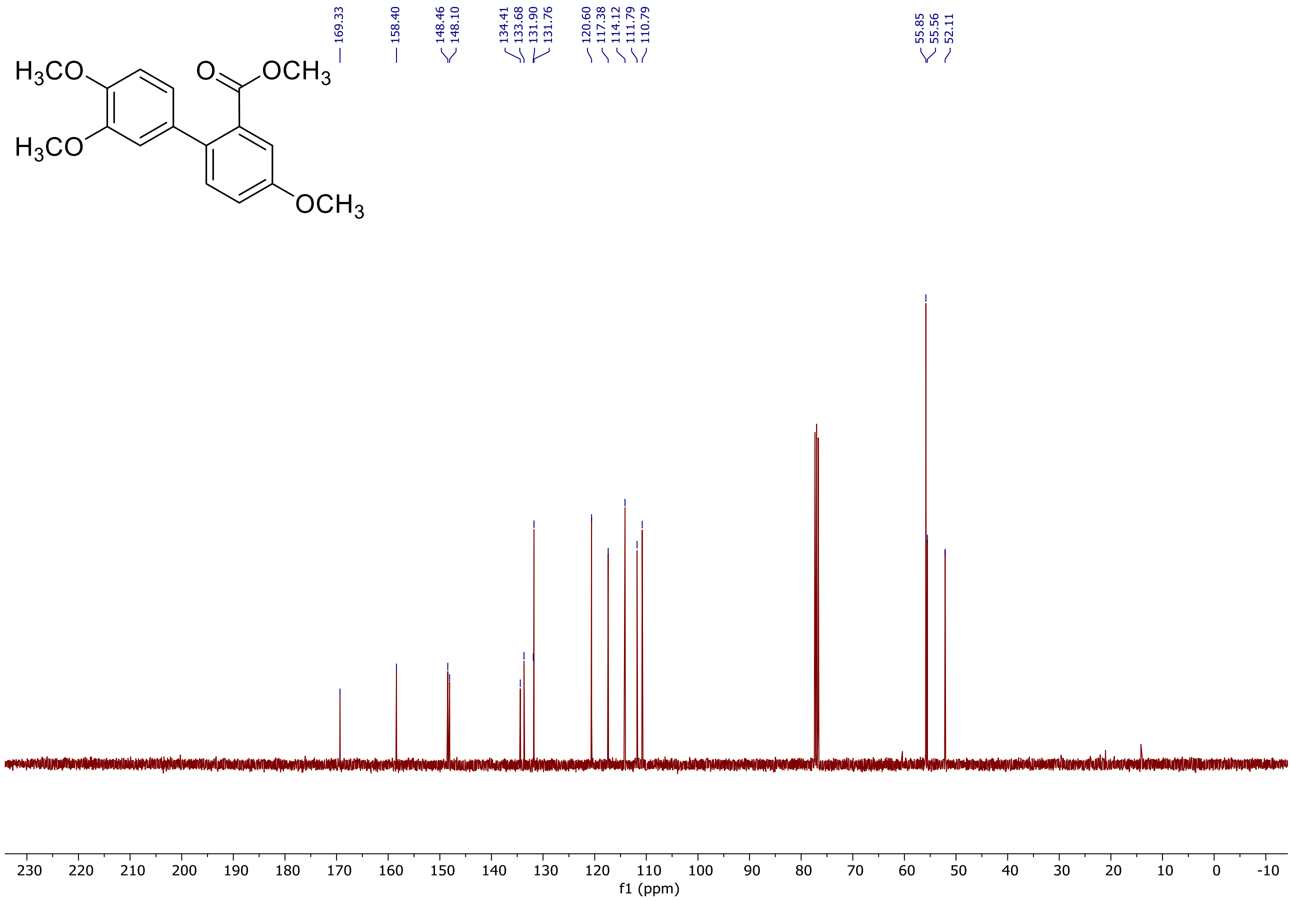

*3',4,4'-Trimethoxy-[1,1'-biphenyl]-2-carboxylic acid (**159**)*

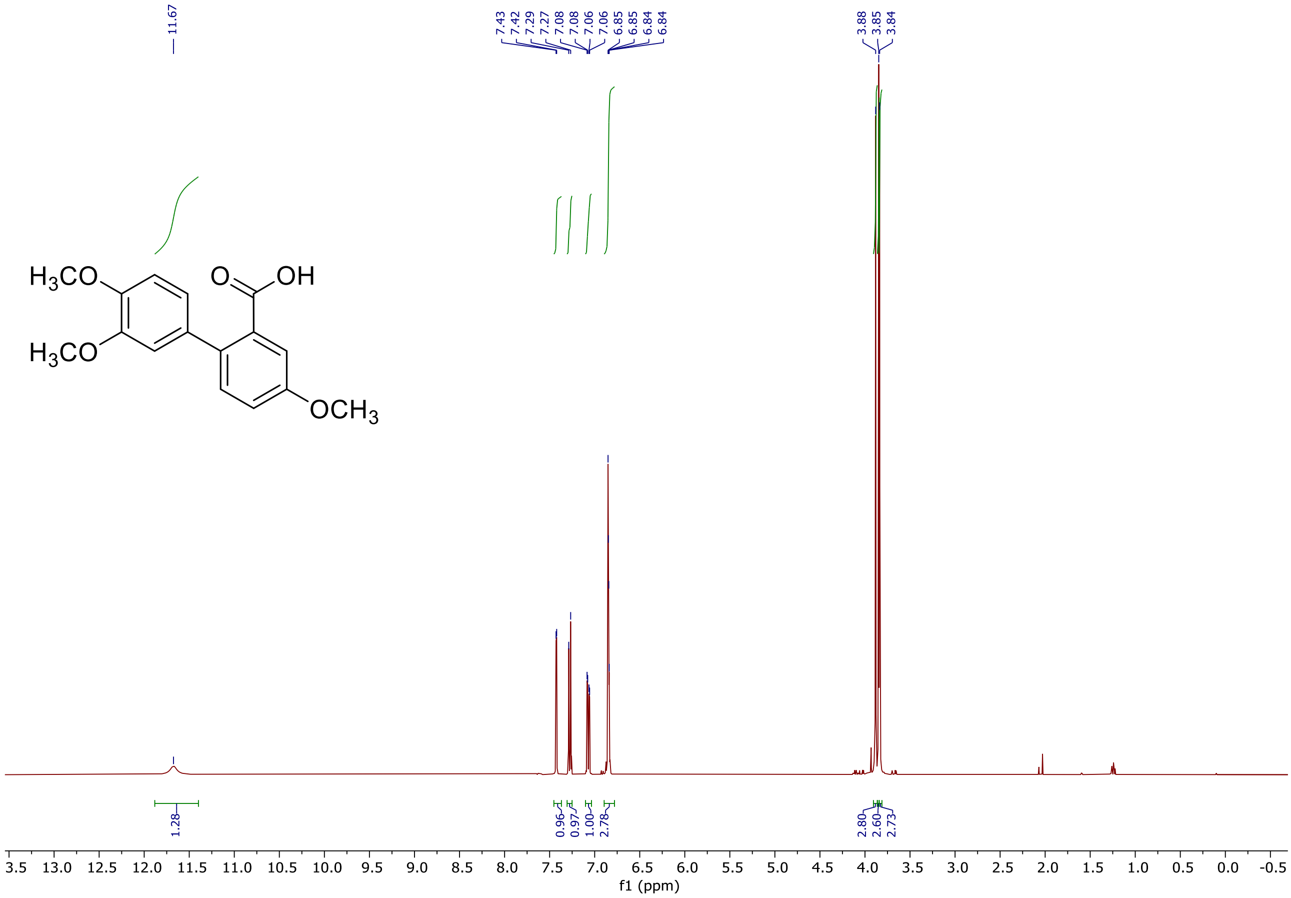

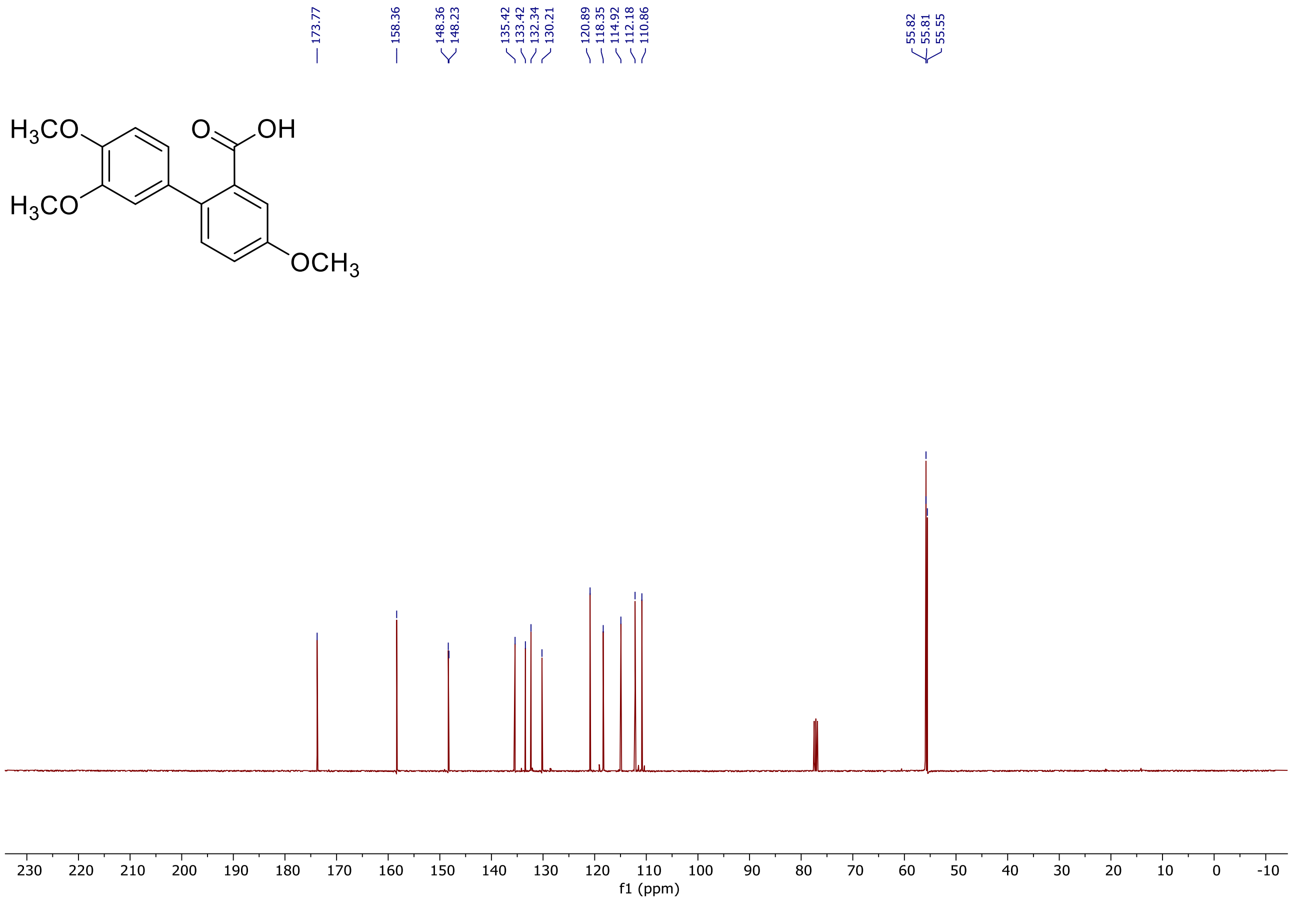

2,3,8-Trimethoxy-6H-benzo[c]chromen-6-one (**160**)

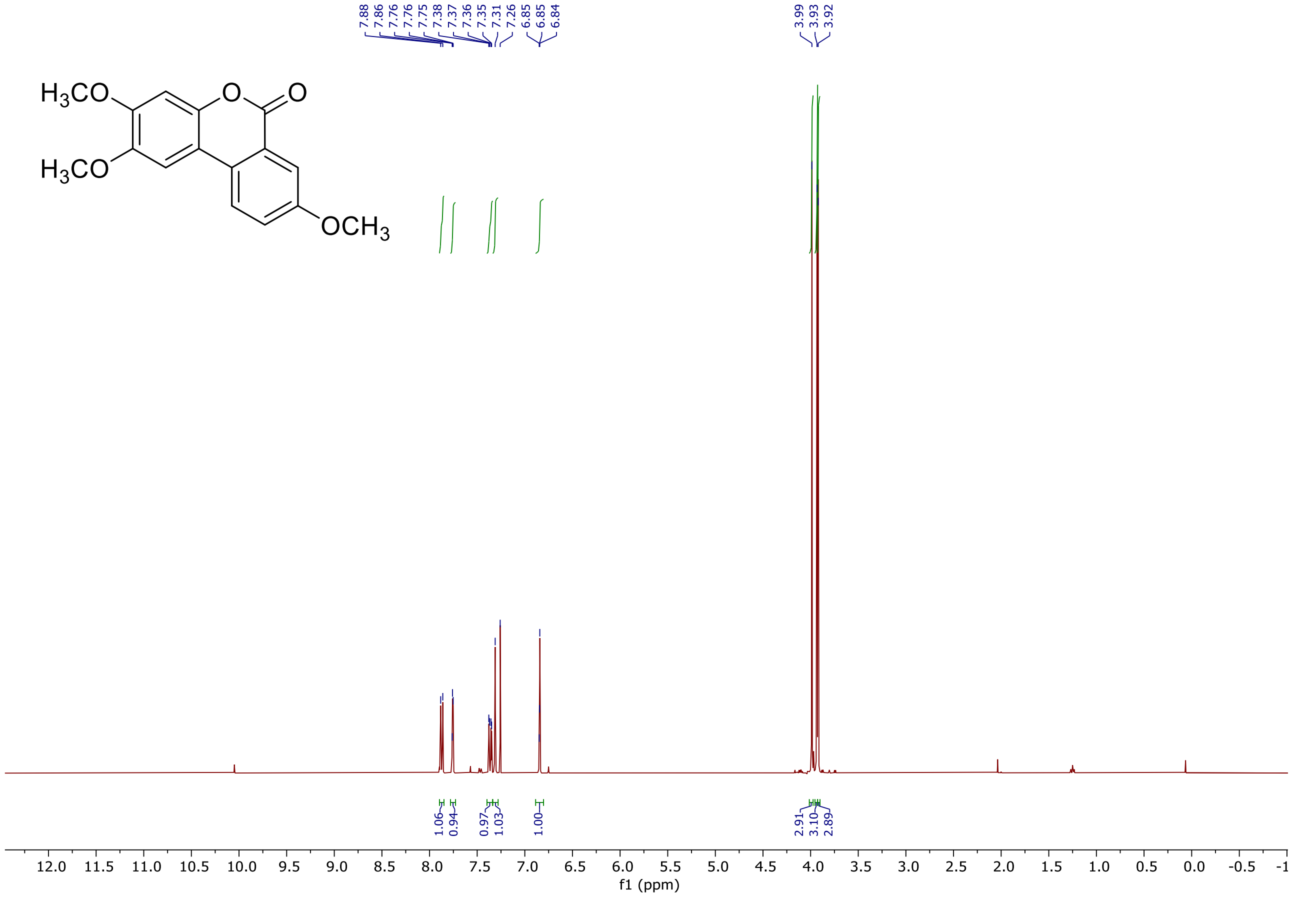

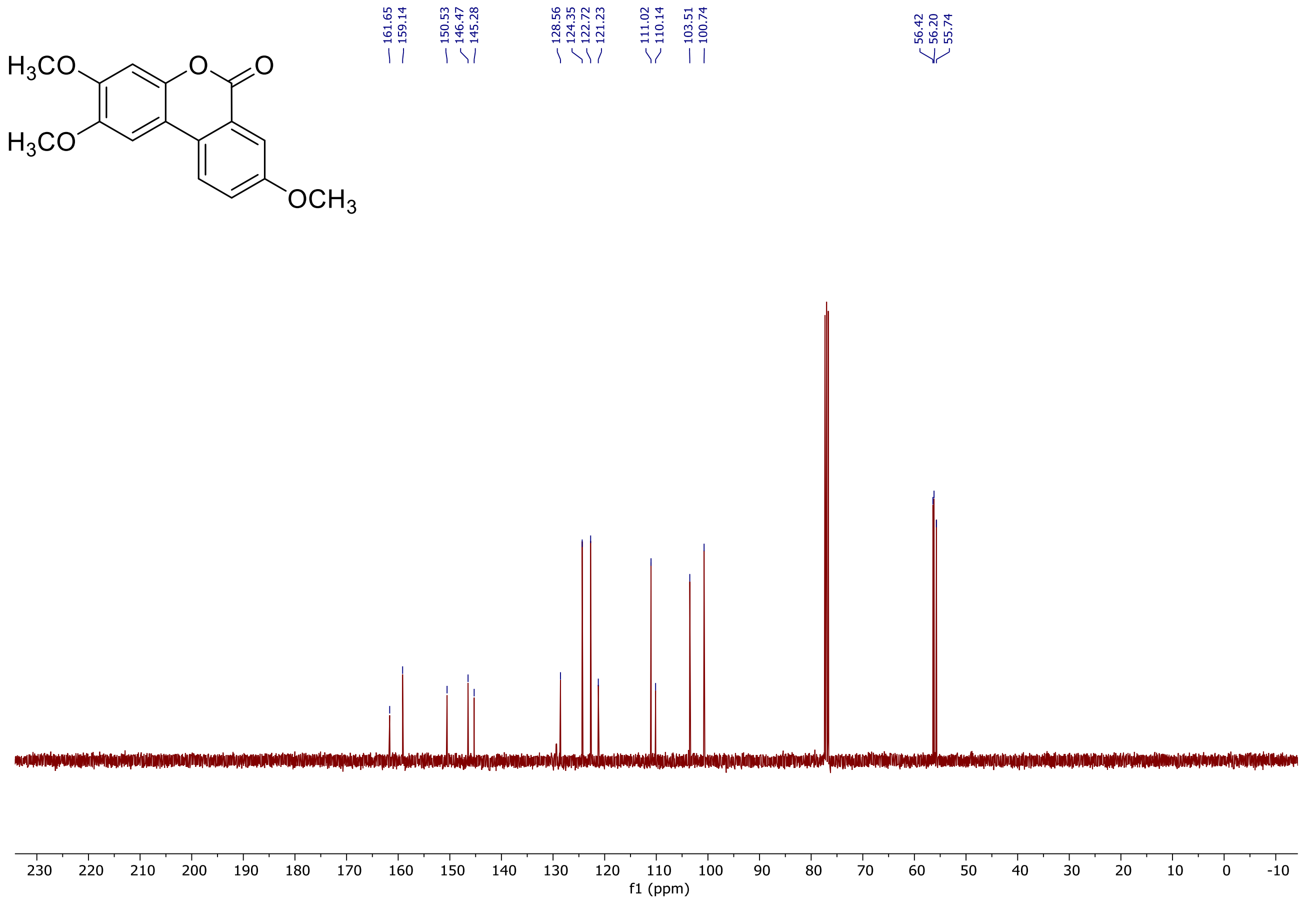

2,3,8-Trihydroxy-6H-benzo[c]chromen-6-one (**161**)

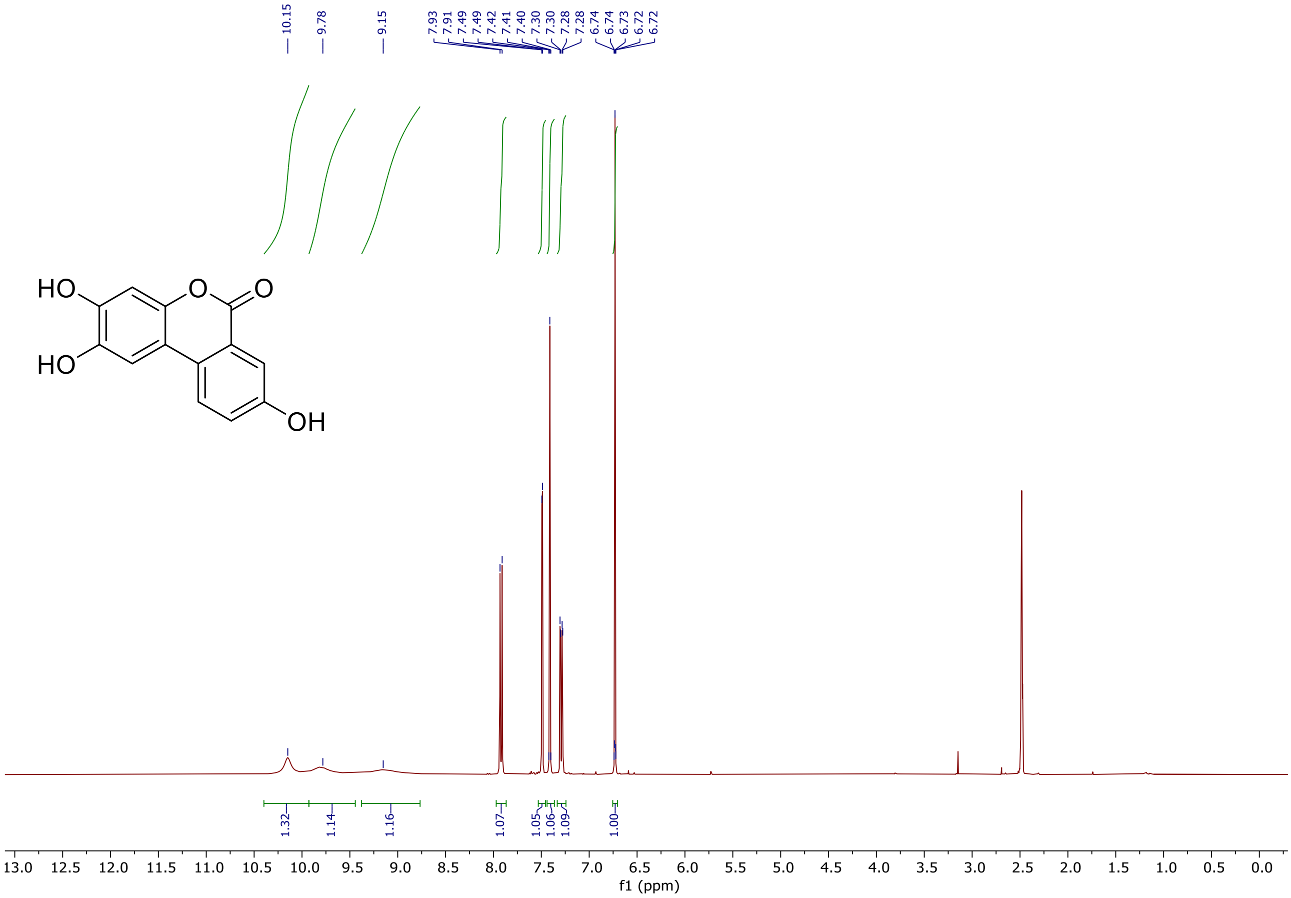

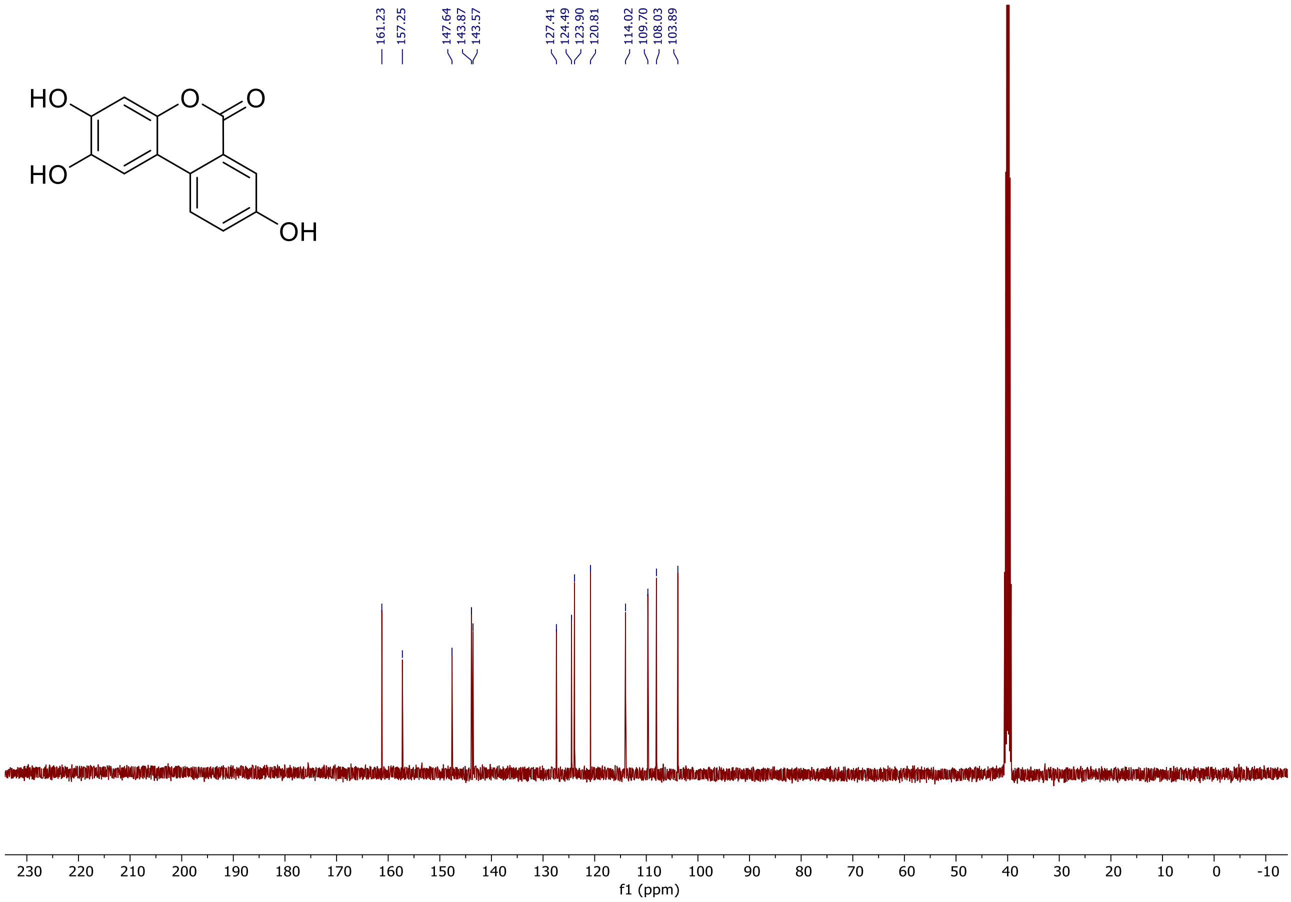

2-Bromo-4,5-dimethoxybenzamide (**162**)

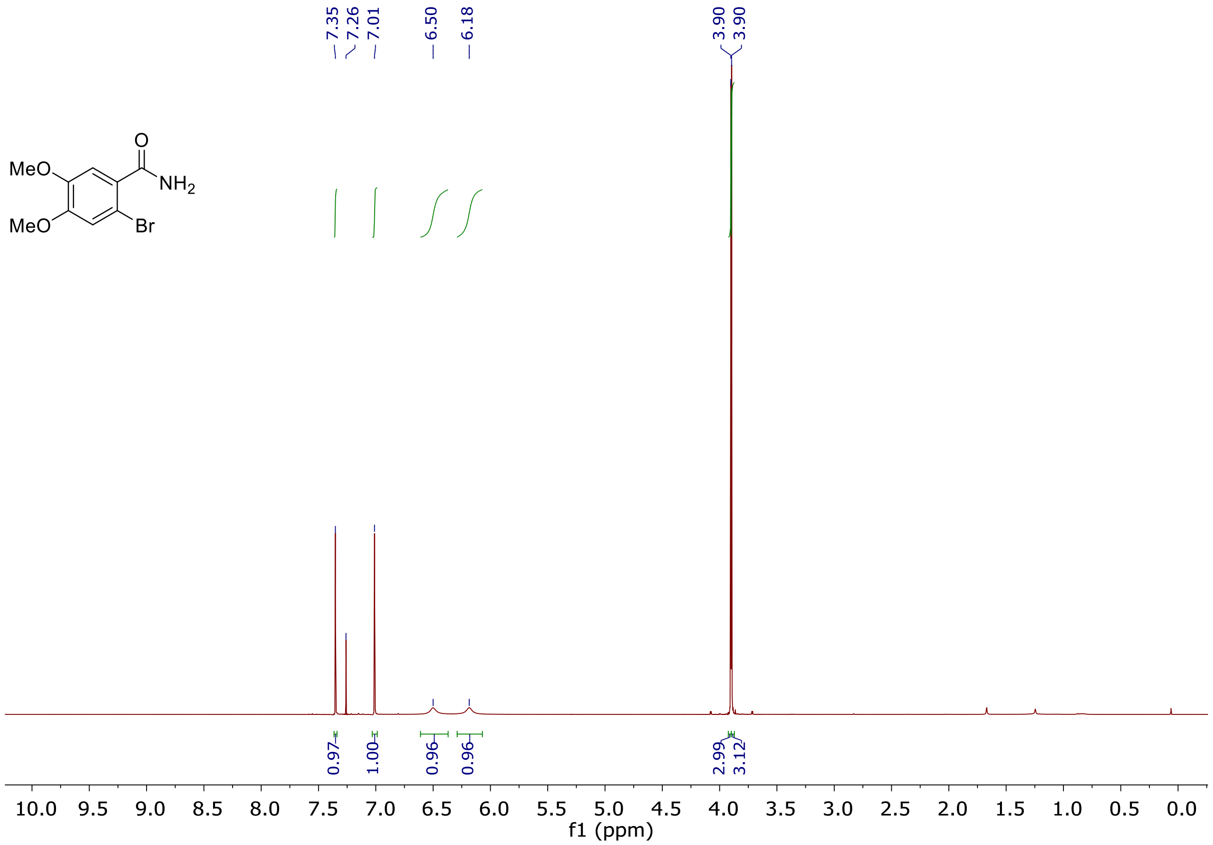

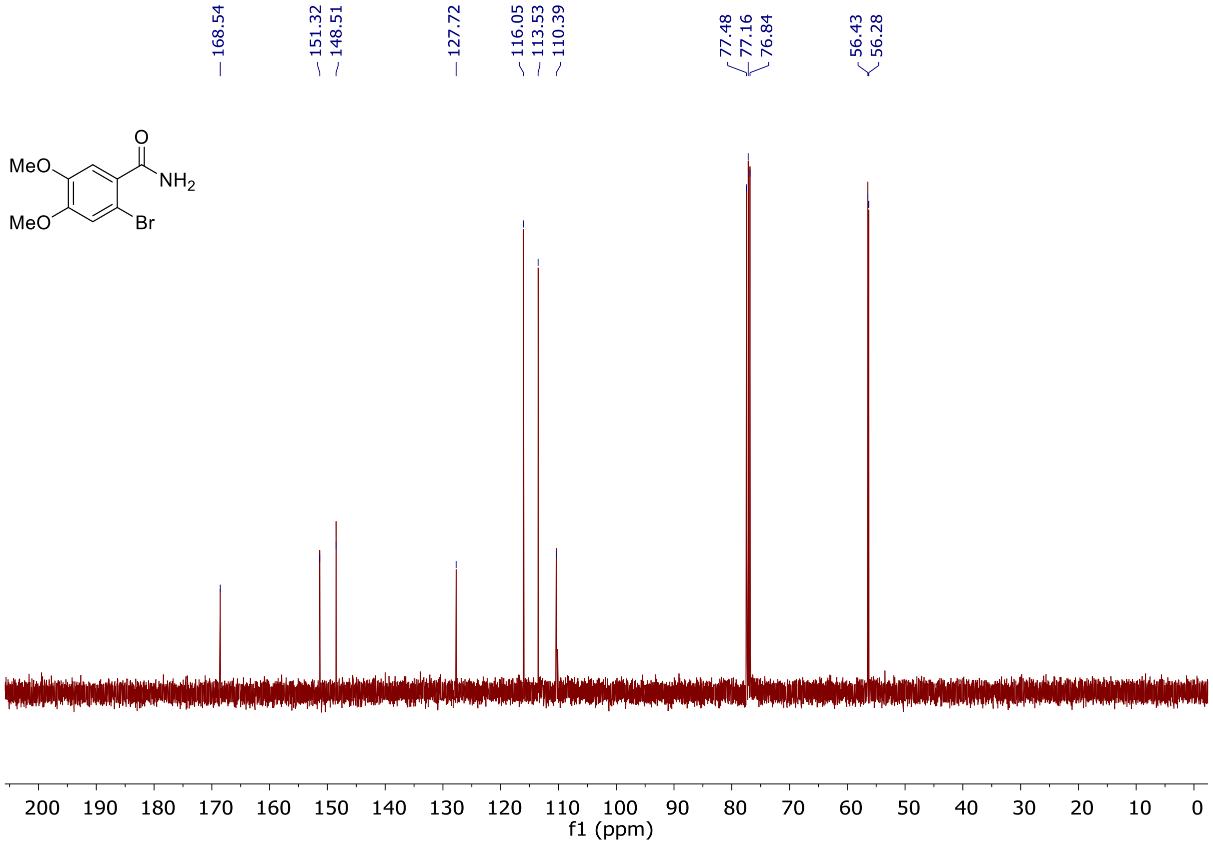

4,4',5-Trimethoxy-[1,1'-biphenyl]-2-carboxamide (**163**)

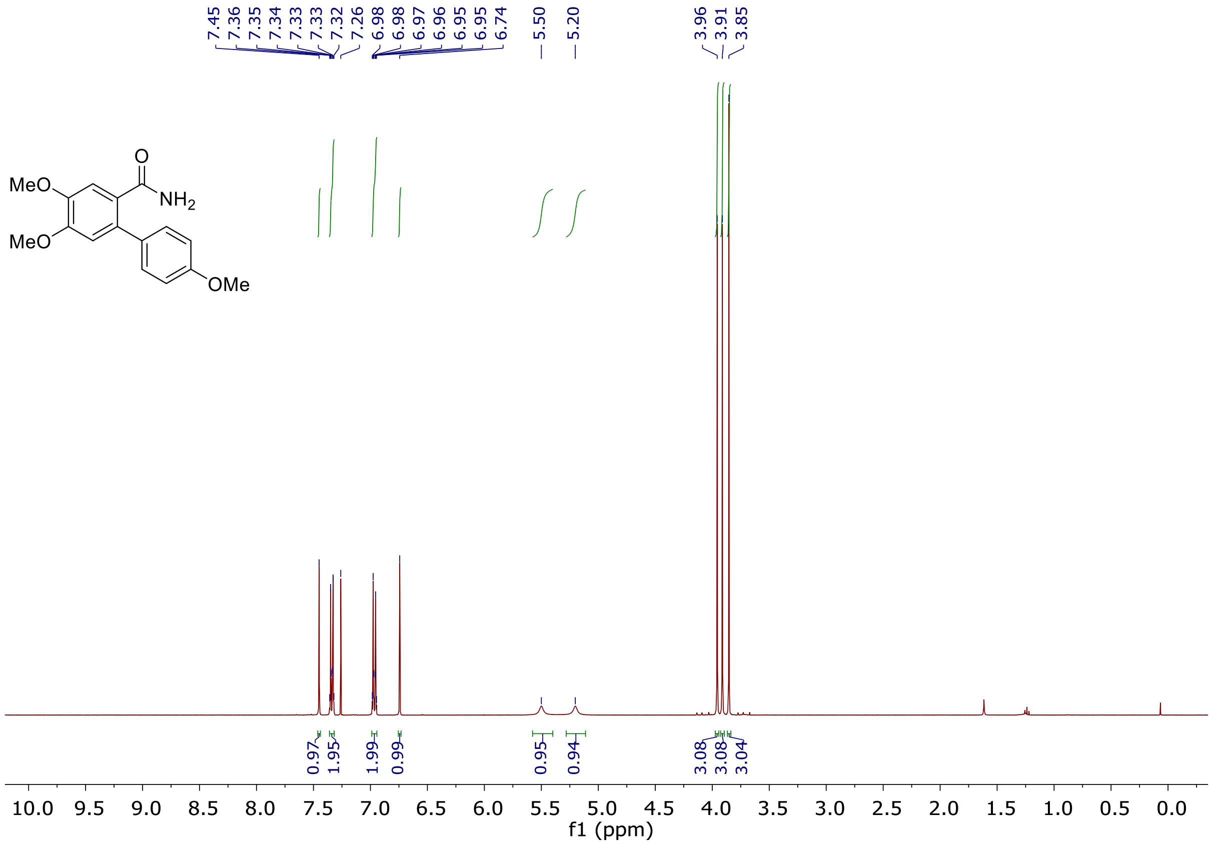

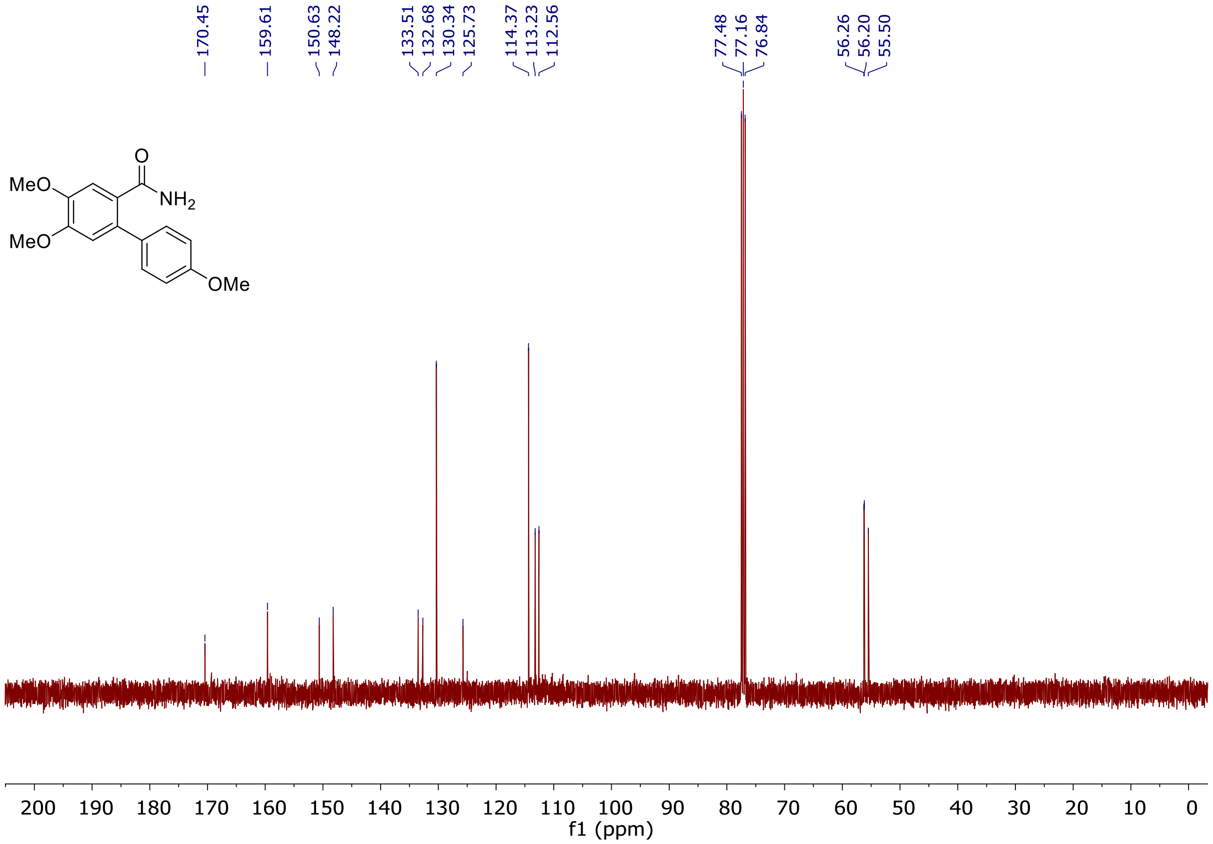

3,8,9-Trimethoxyphenanthridin-6(5H)-one (**164**)

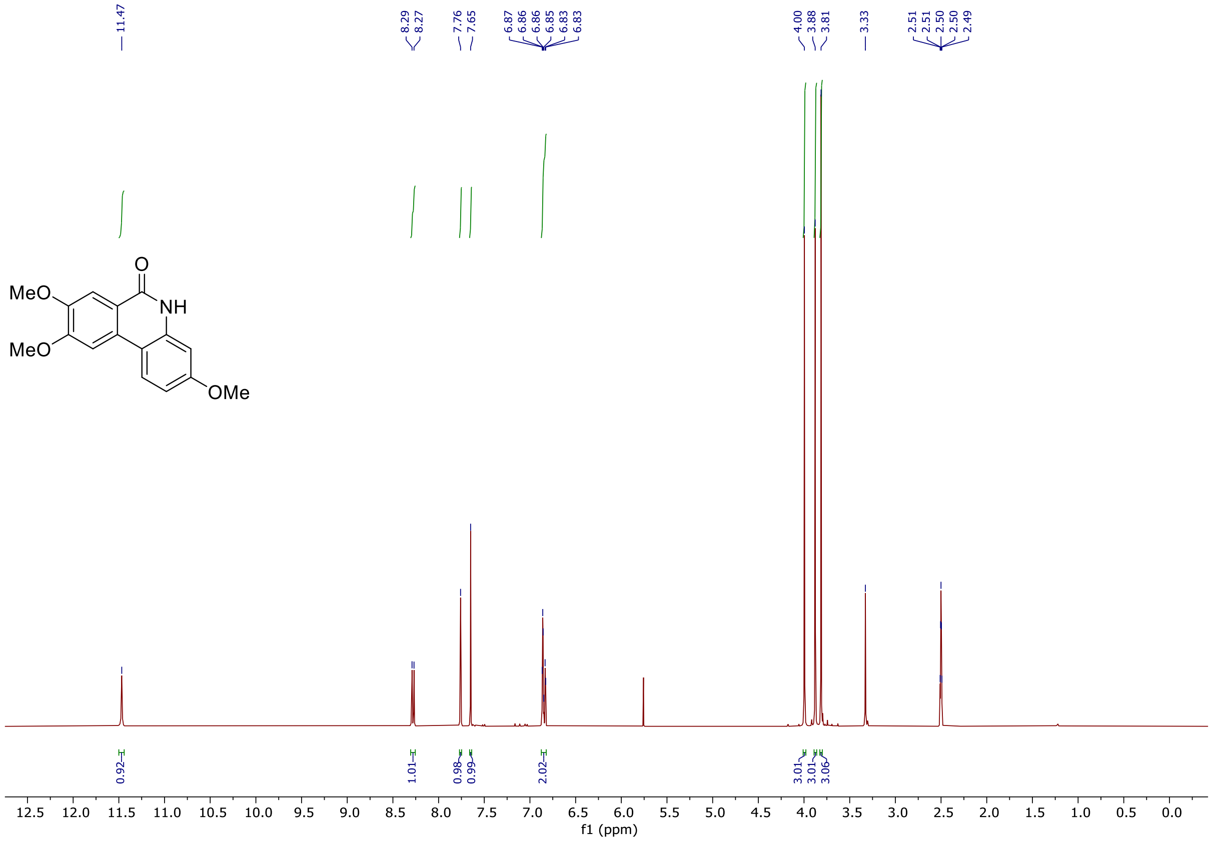

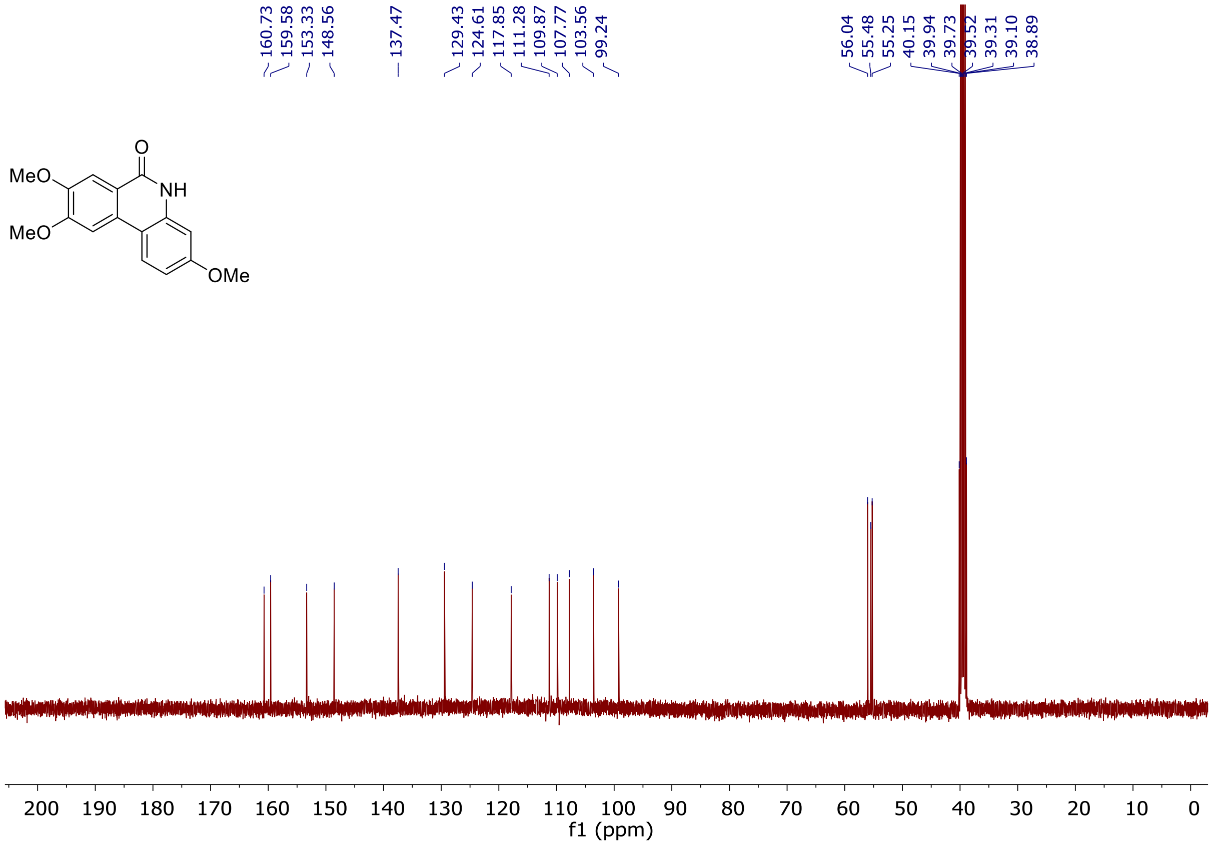

3,8,9-Trihydroxyphenanthridin-6(5H)-one (**165**)

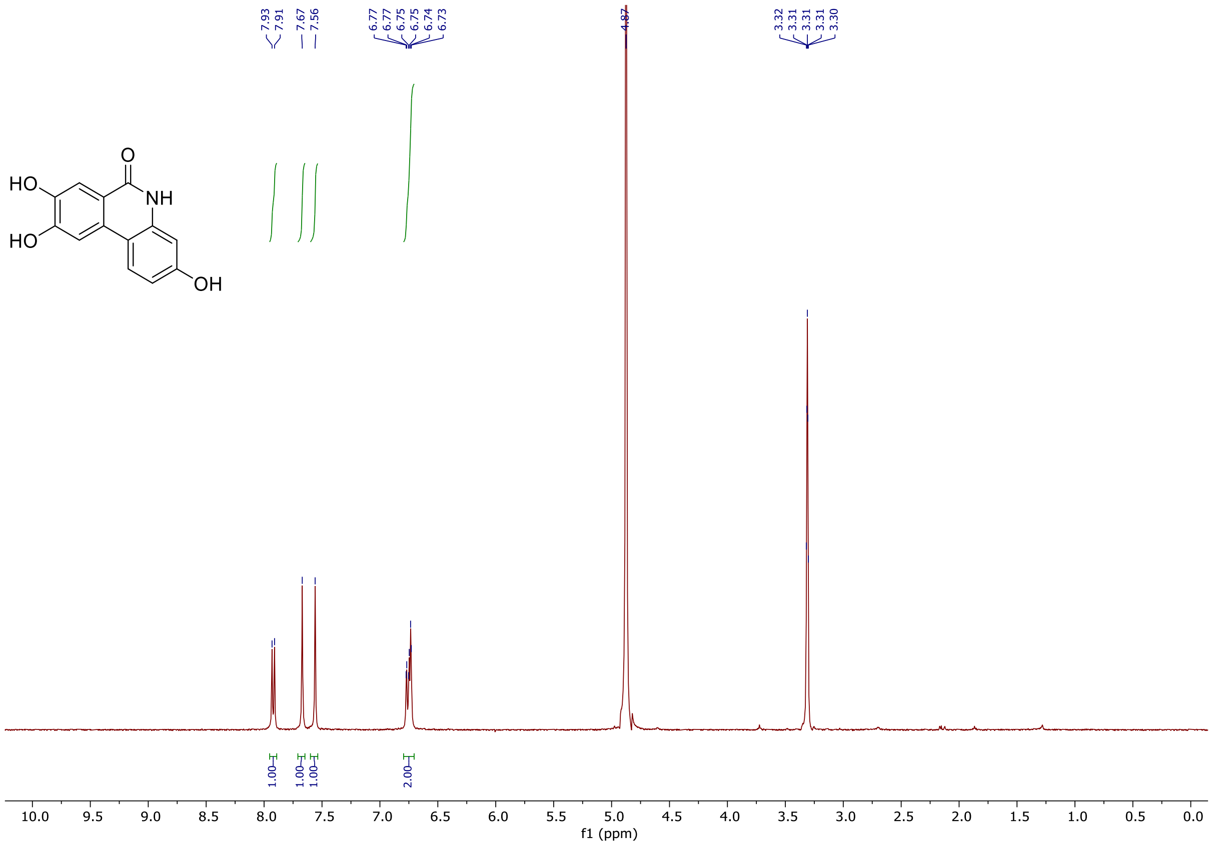

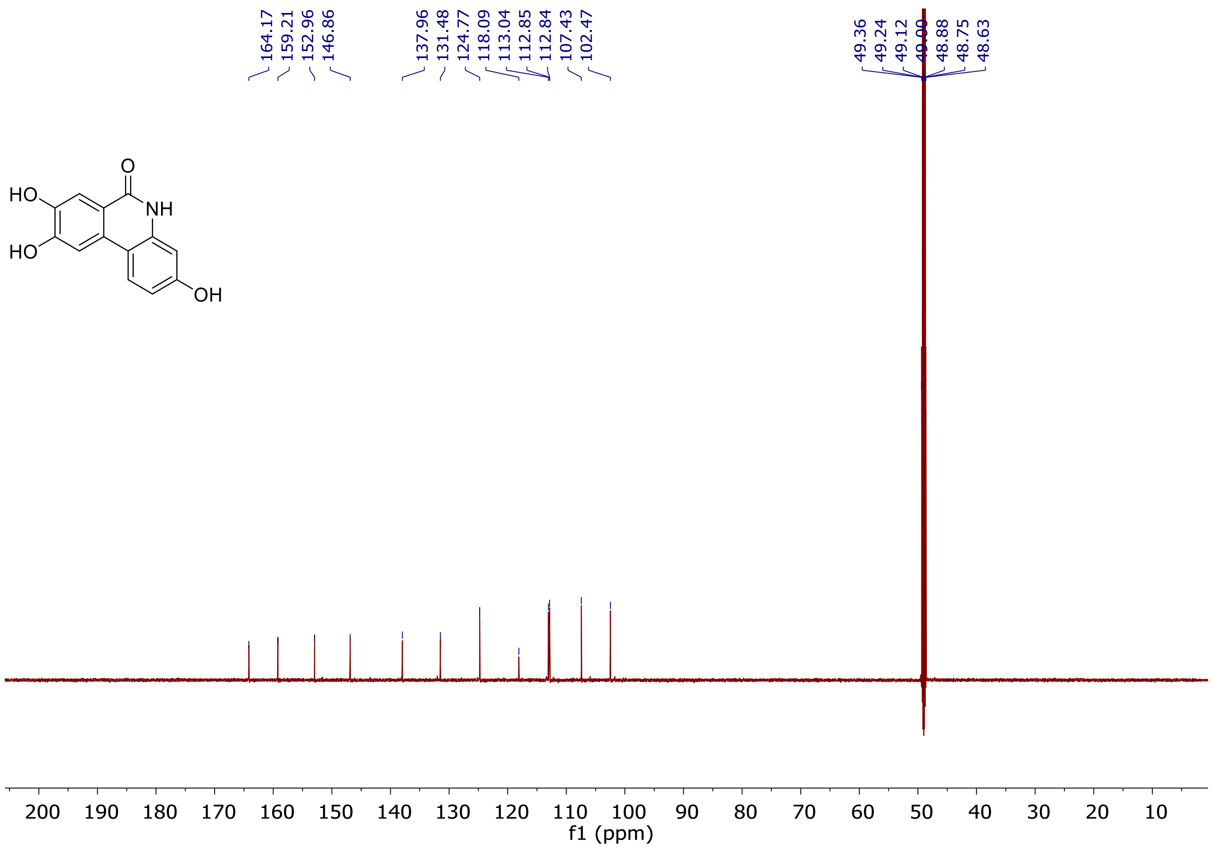

3,8,9-Trimethoxy-5-methylphenanthridin-6(5H)-one (**166**)

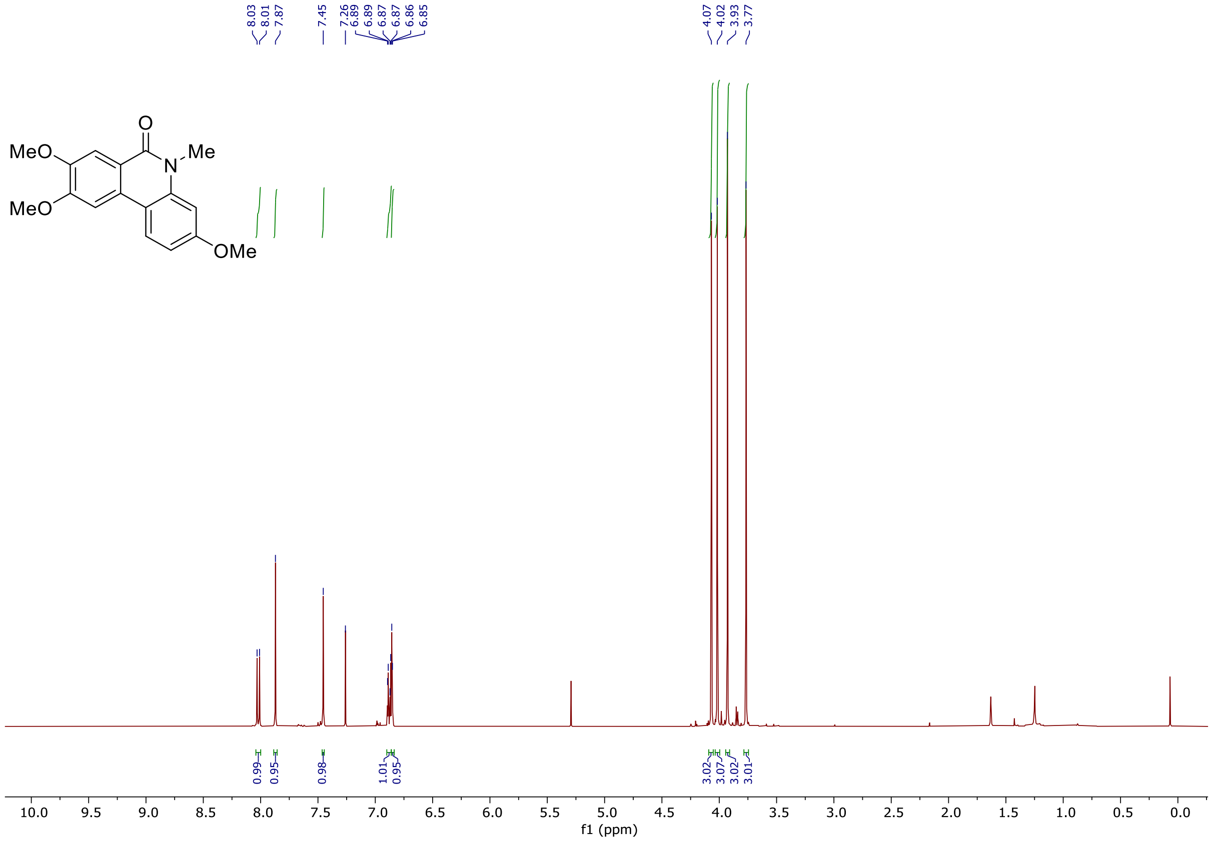

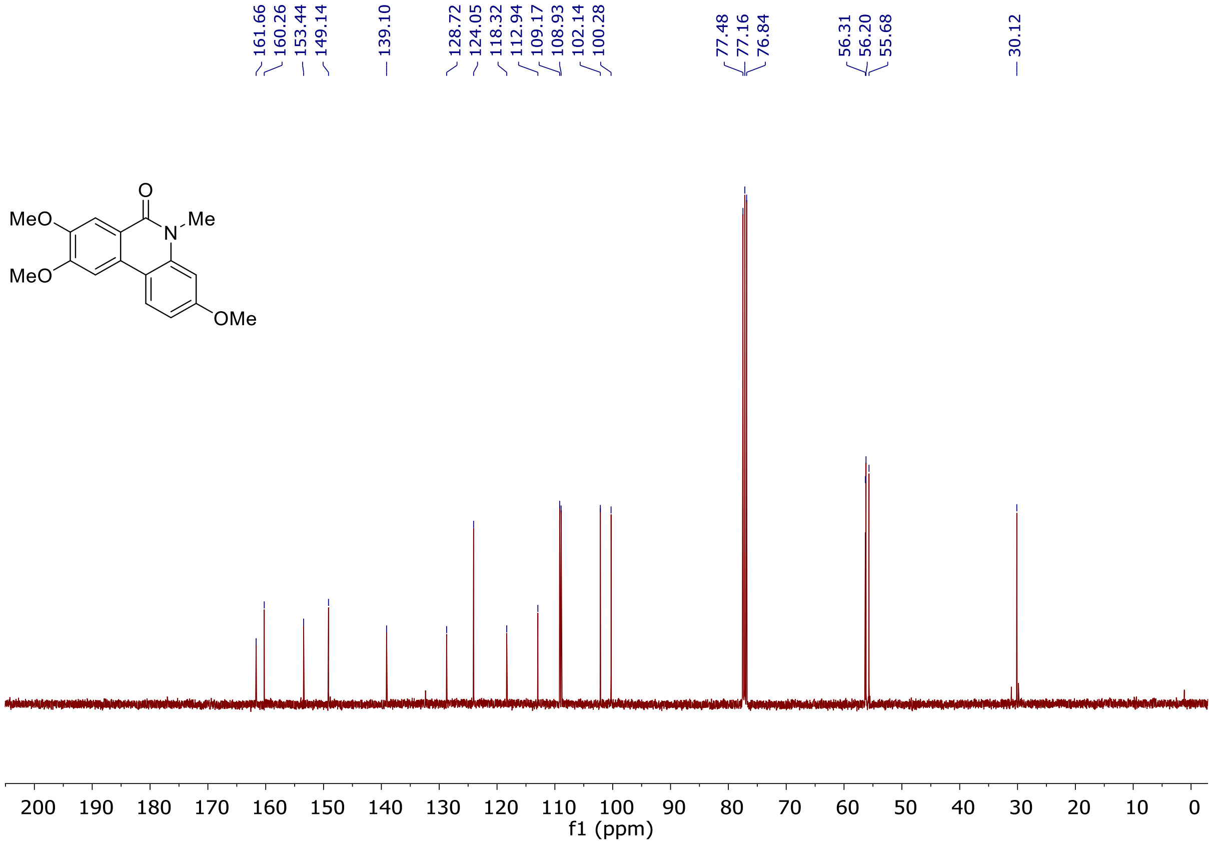

3,8,9-Trihydroxy-5-methylphenanthridin-6(5H)-one (**167**)

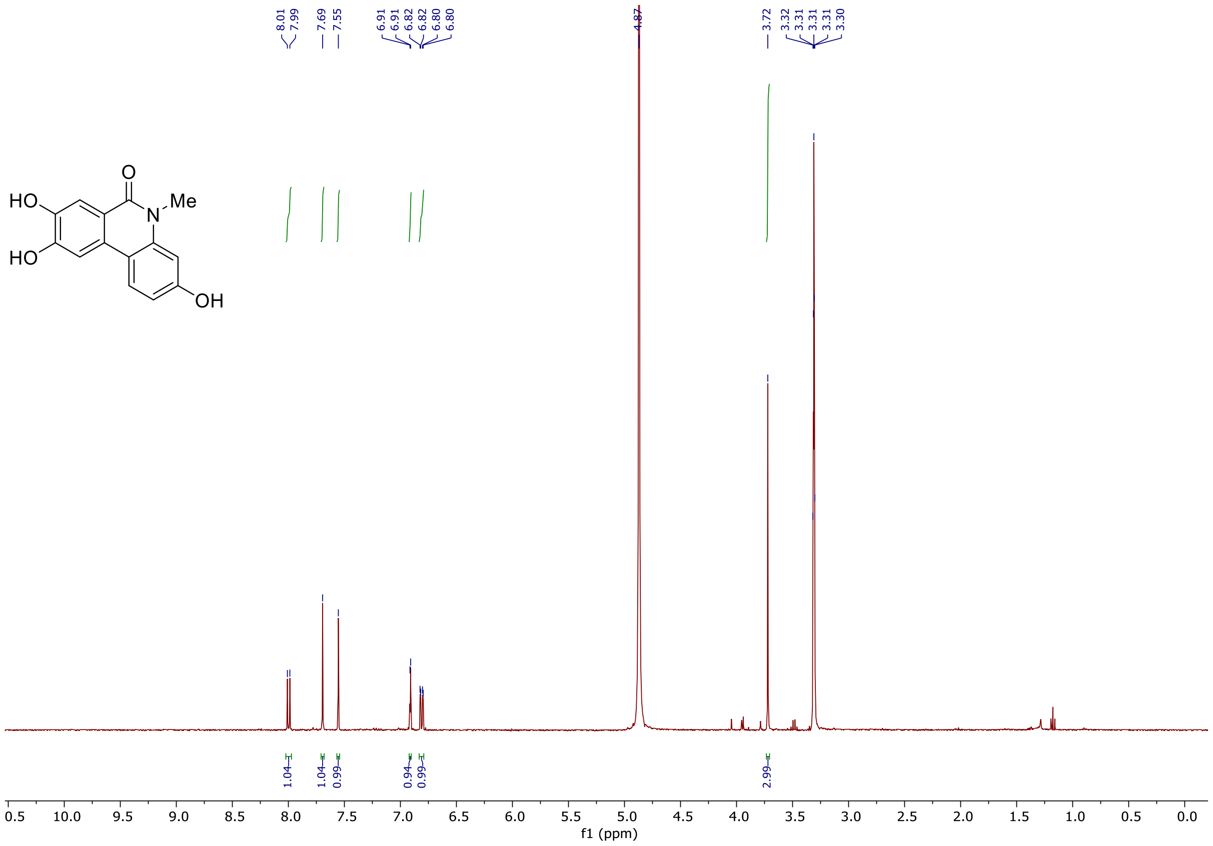

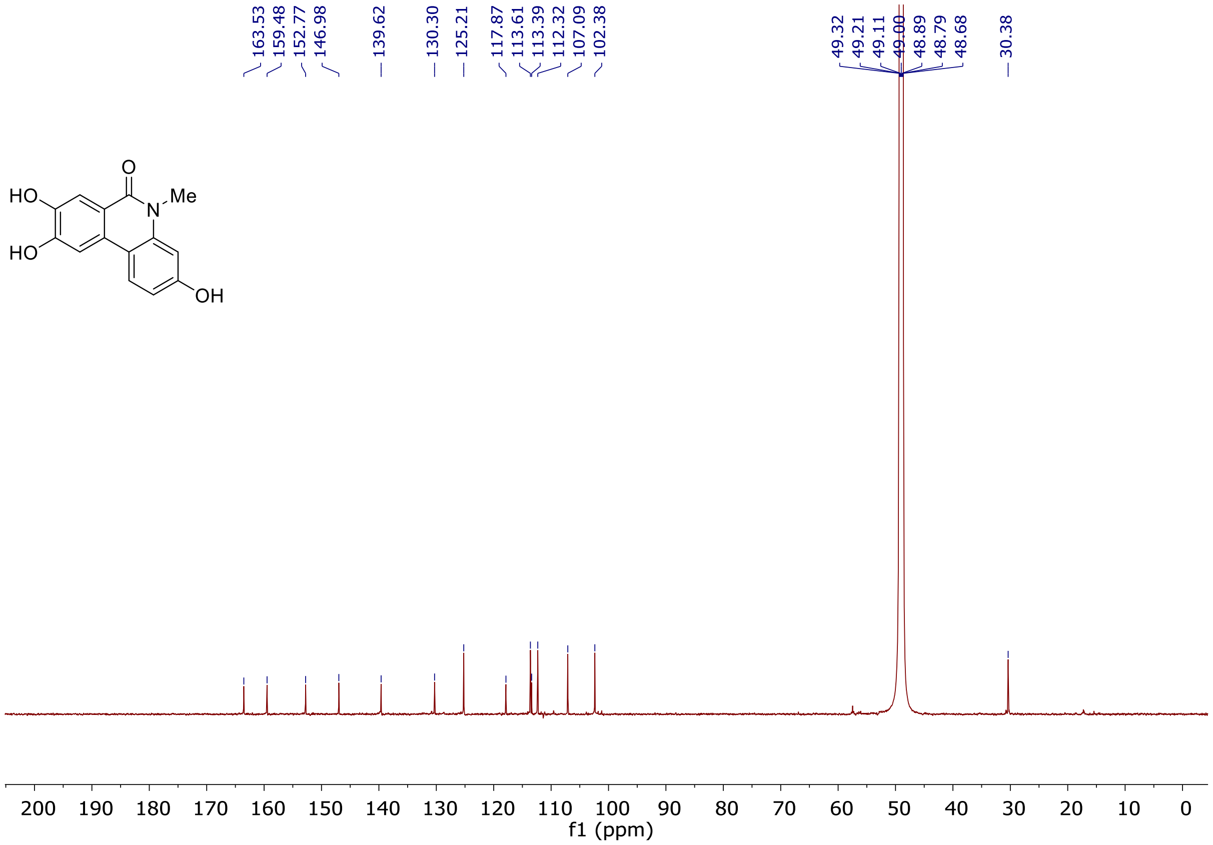

4,4',5-Trimethoxy-[1,1'-biphenyl]-2-carbaldehyde (**170**)

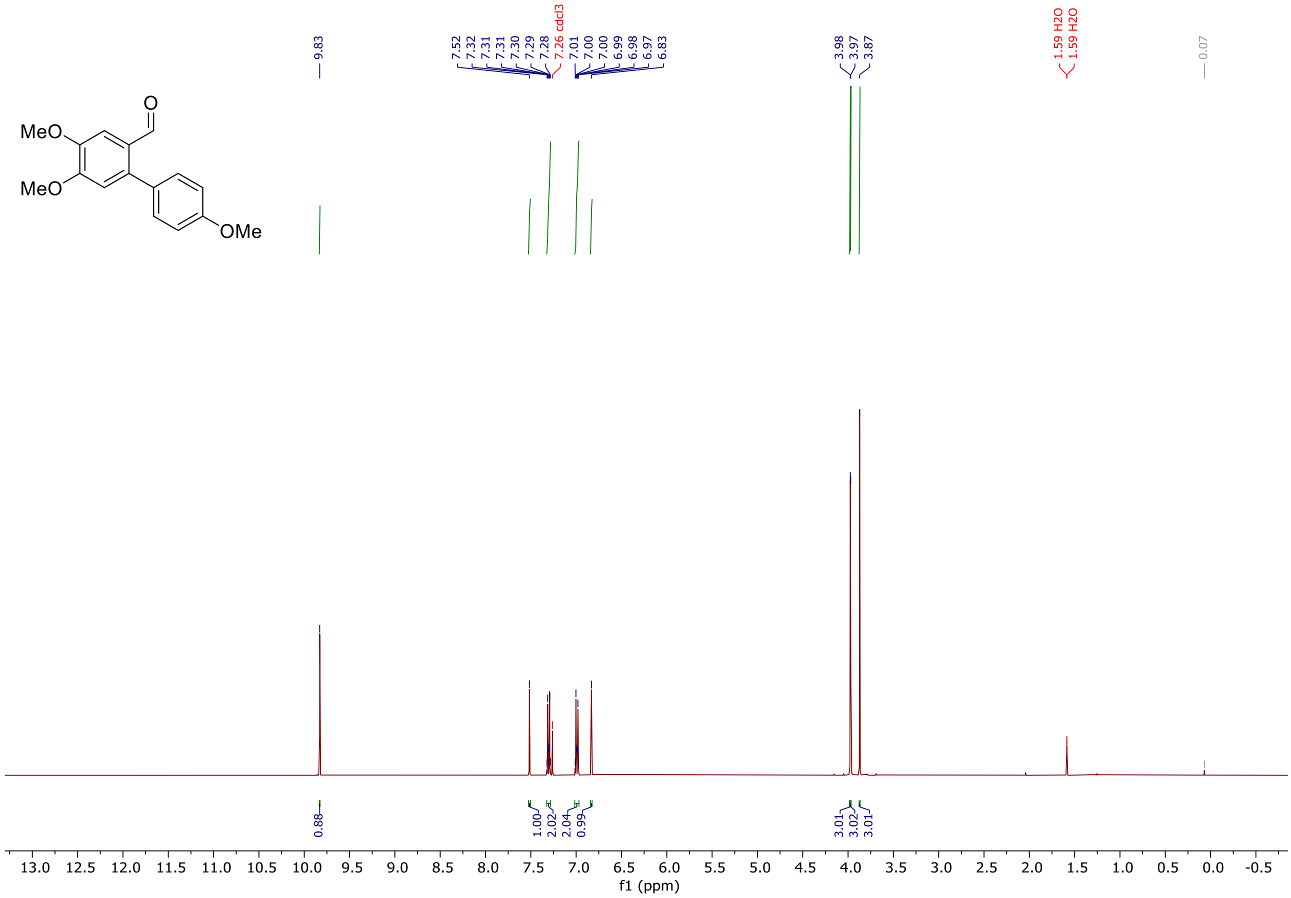

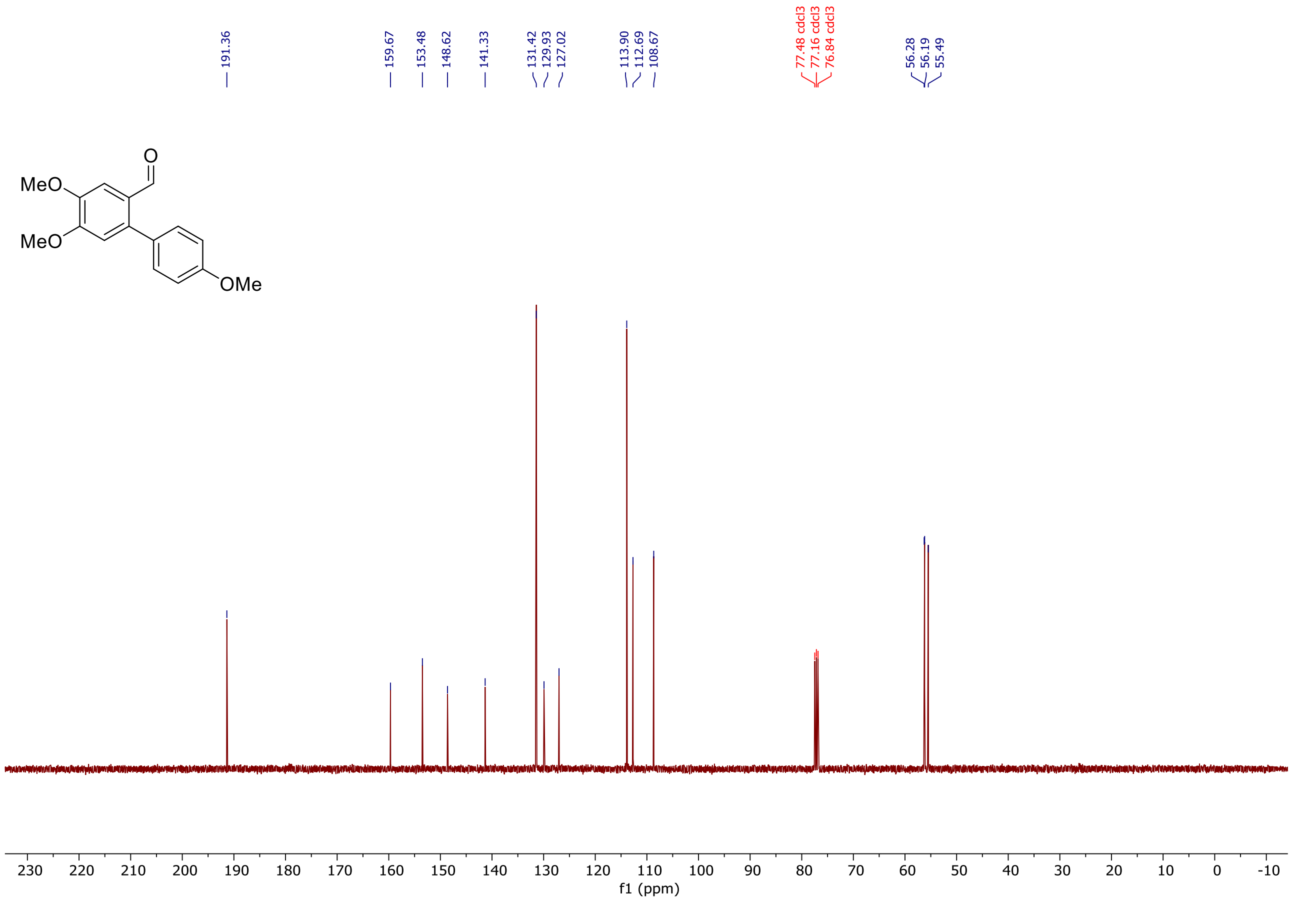

(E/Z)-4,4',5-Trimethoxy-2-(2-methoxyvinyl)-1,1'-biphenyl (**172**)

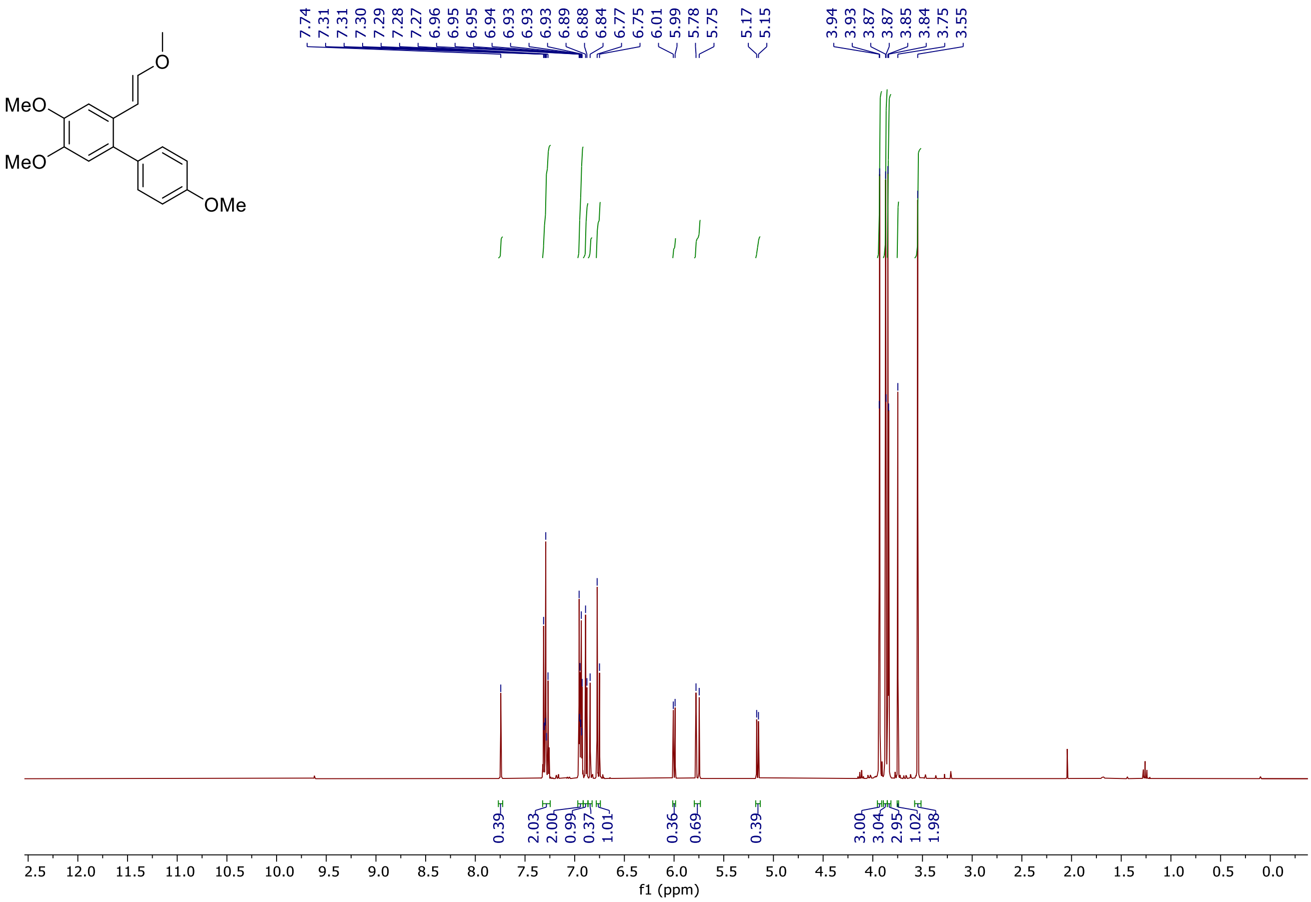

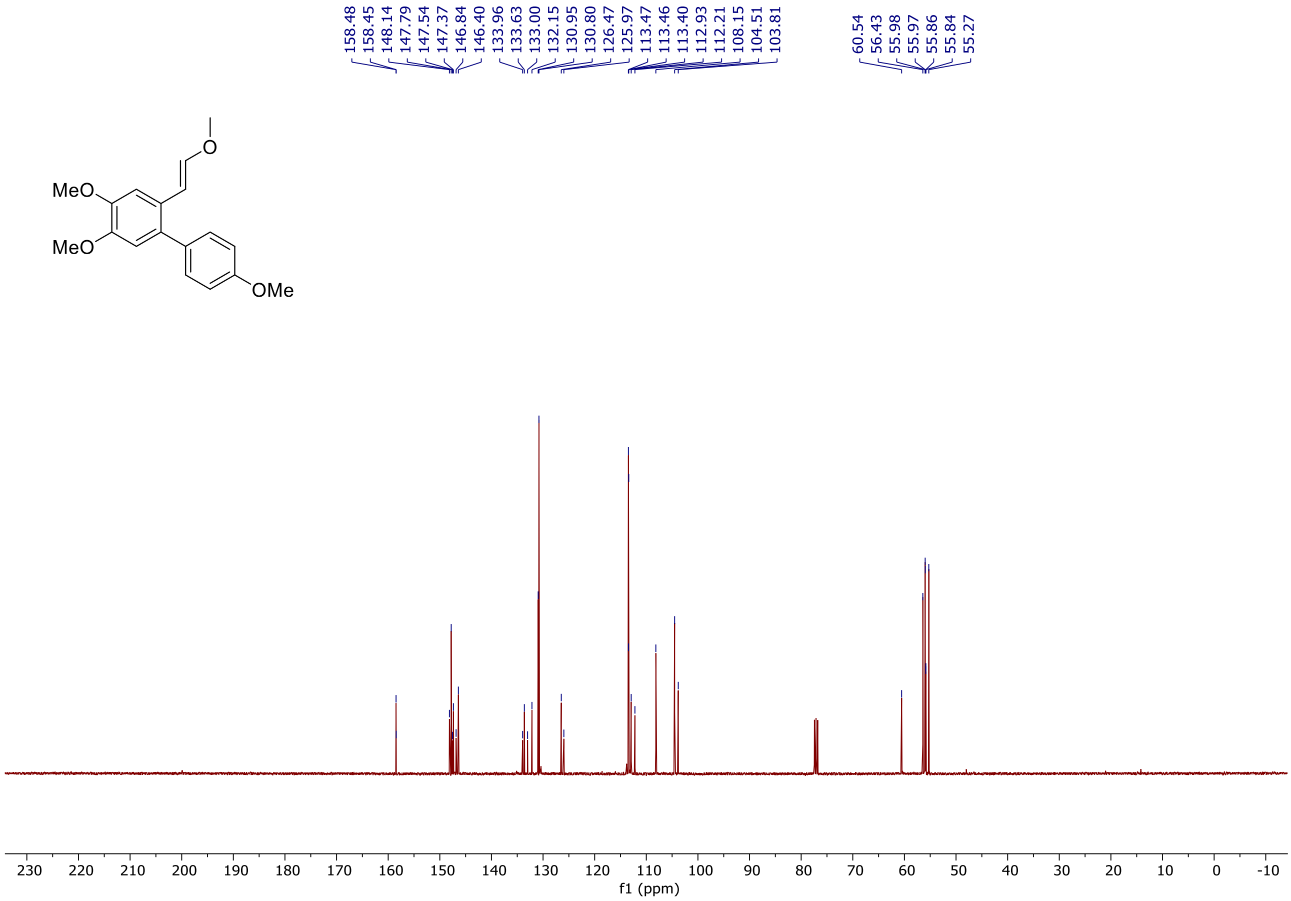

2,3,7-Trimethoxyphenanthrene (**175**)

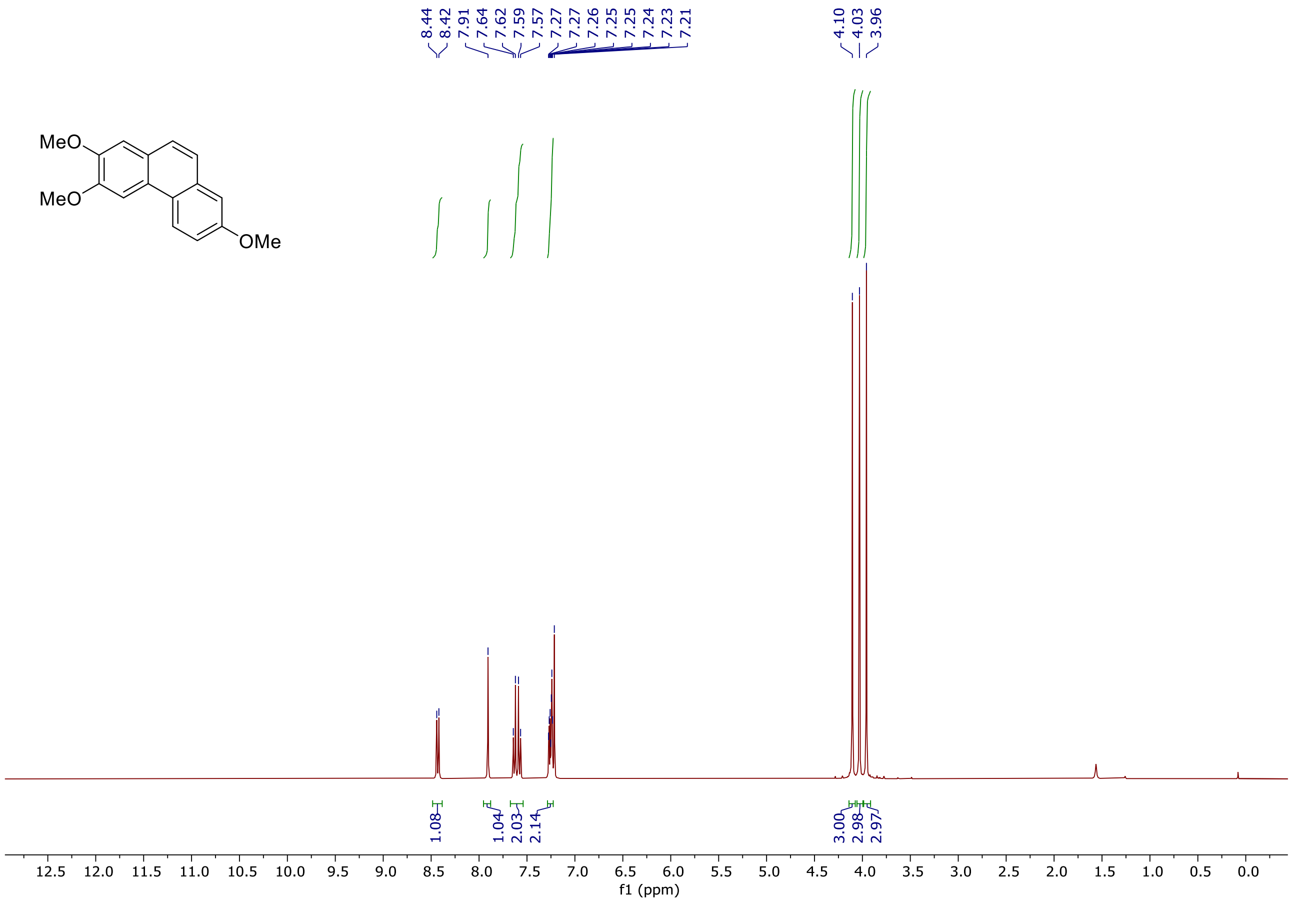

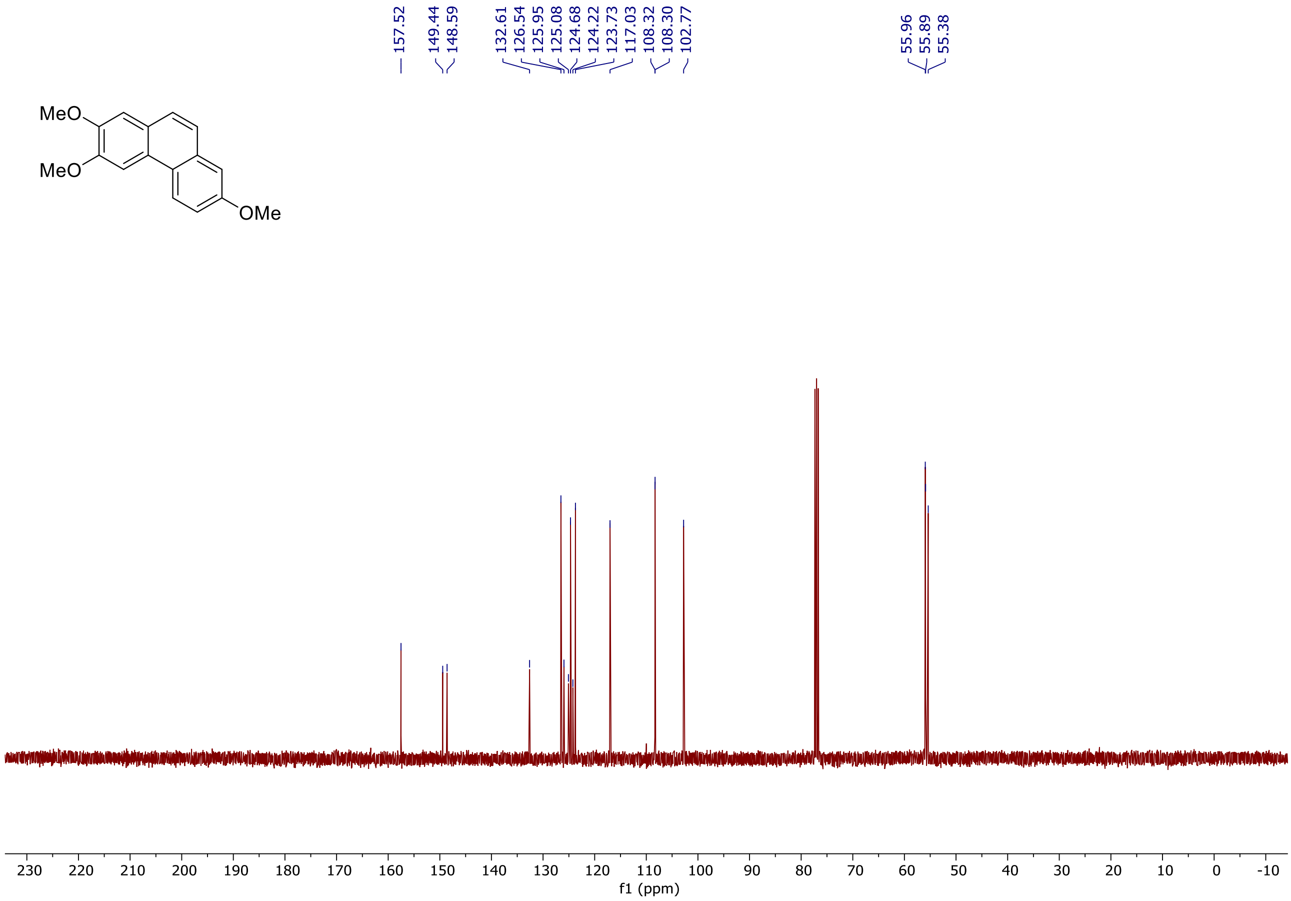

Phenanthrene-2,3,7-triol (**178**)

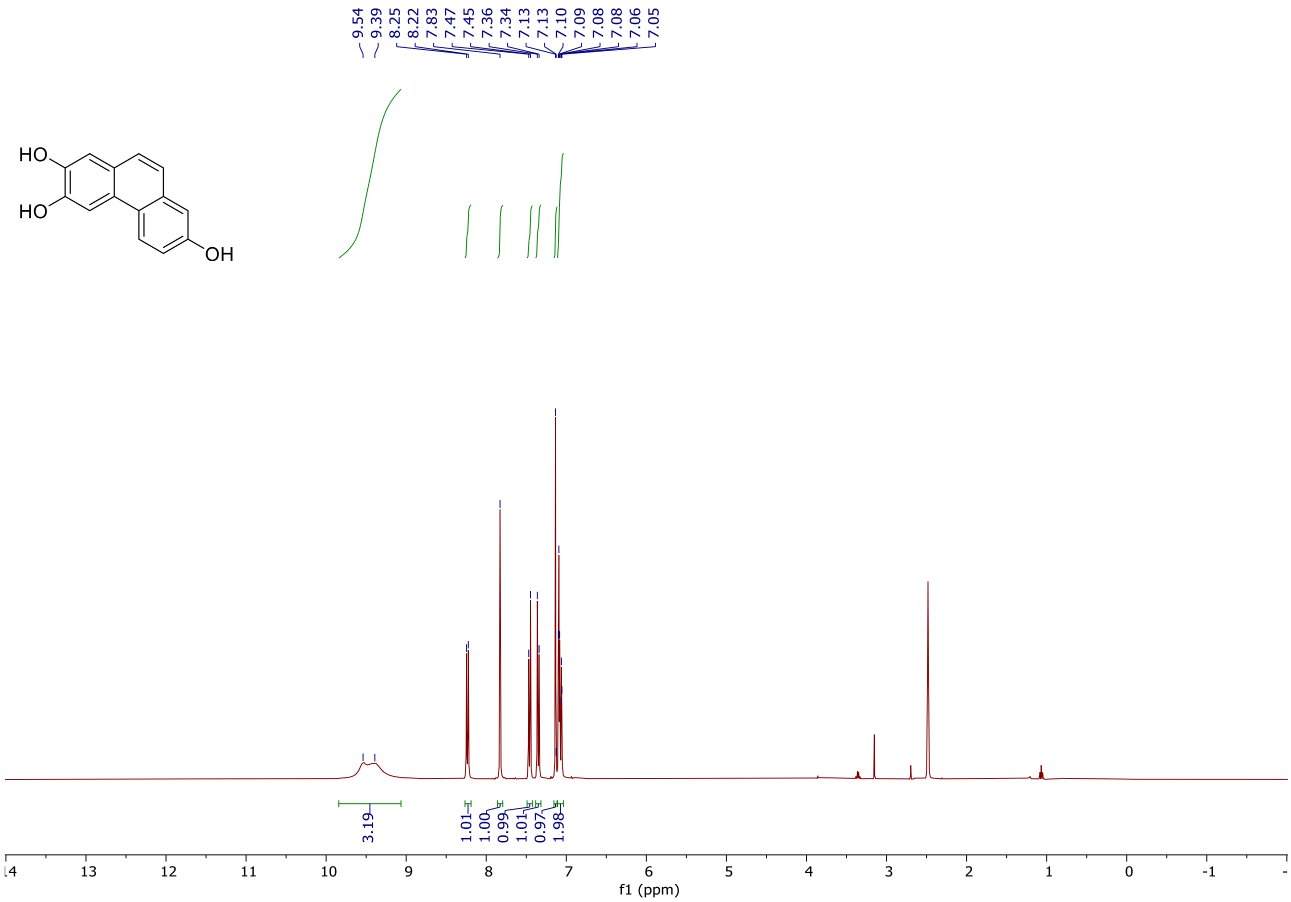

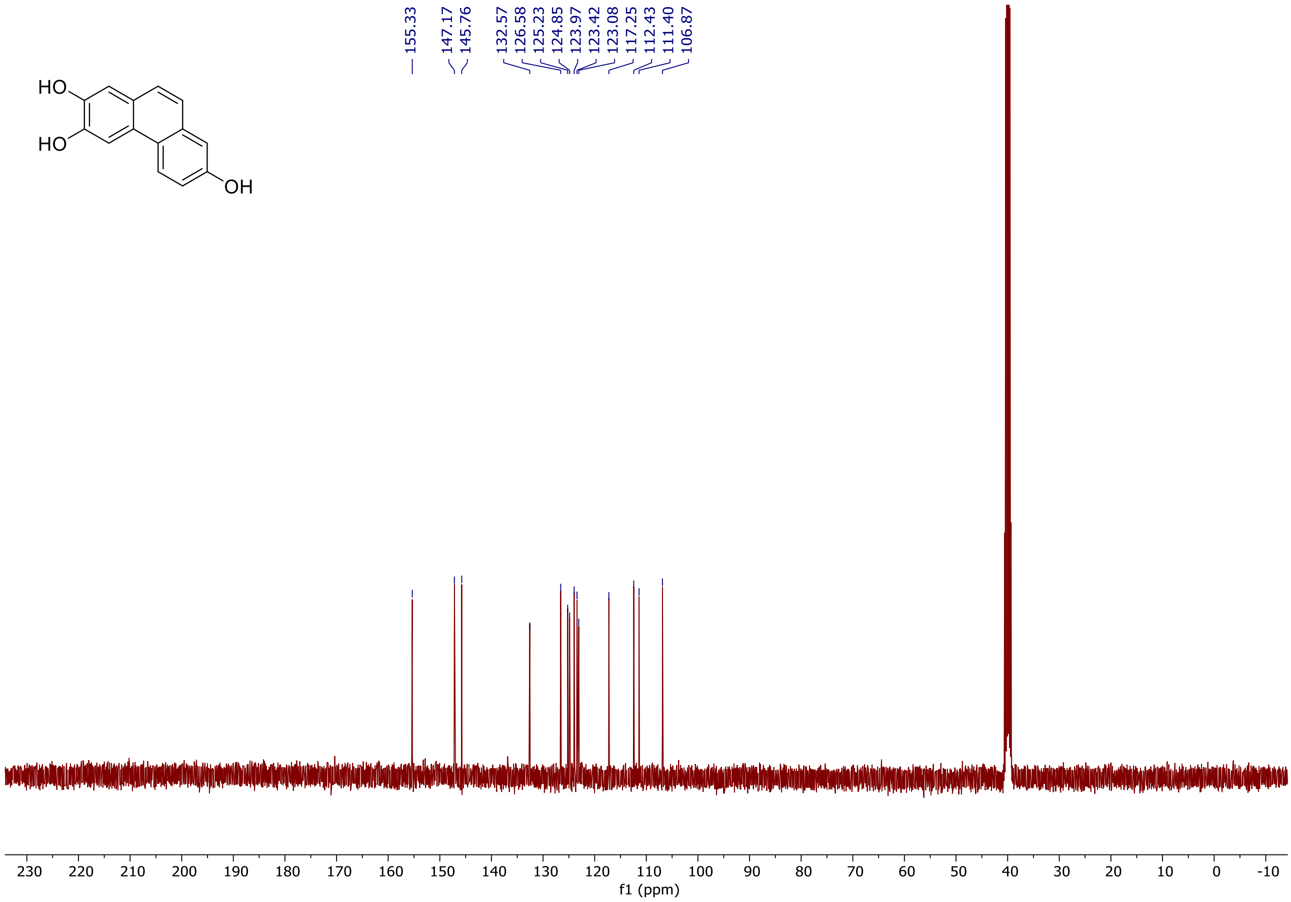

(E)-4,4',5-trimethoxy-[1,1'-biphenyl]-2-carbaldehyde O-acetyl oxime (**173**)

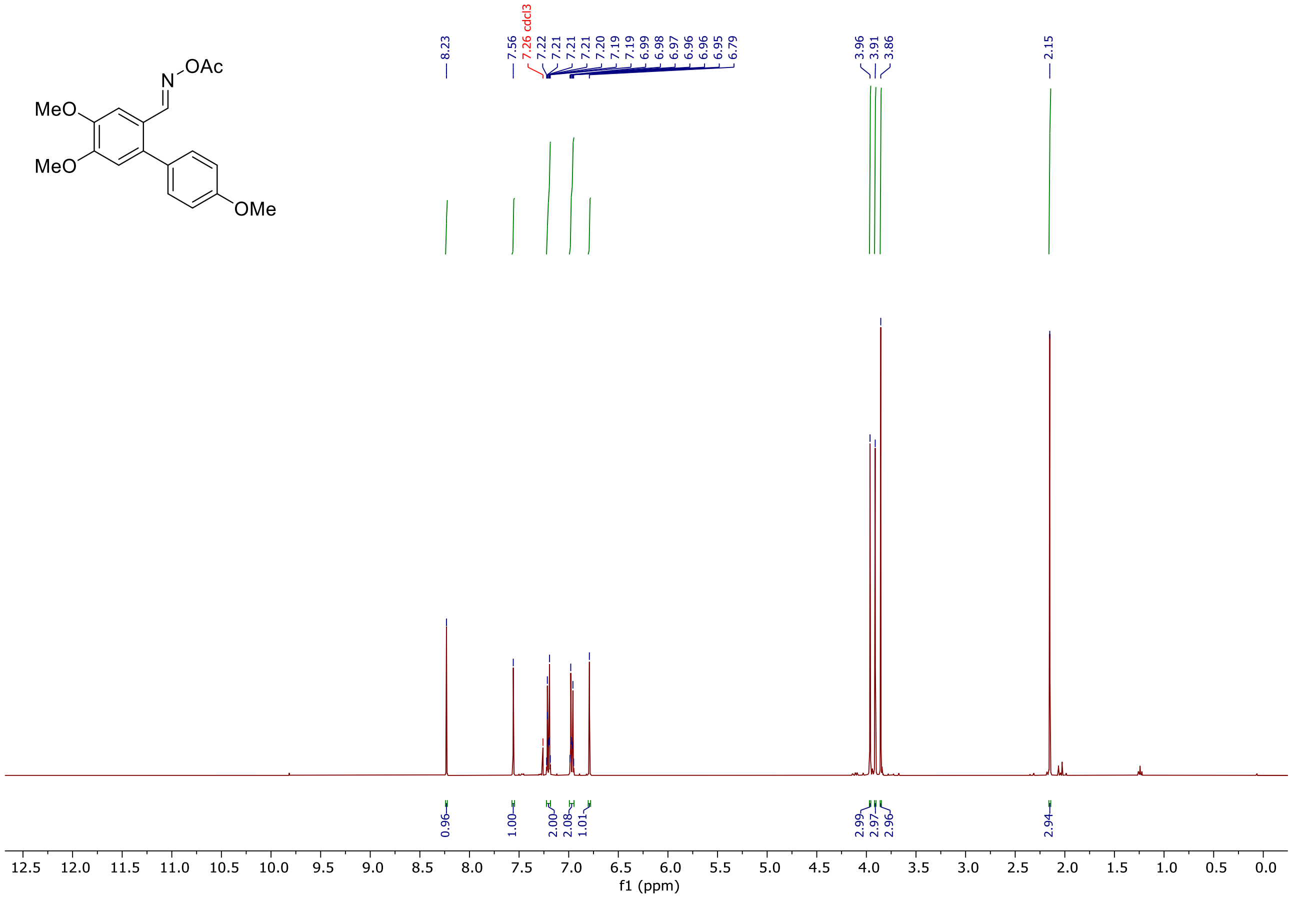

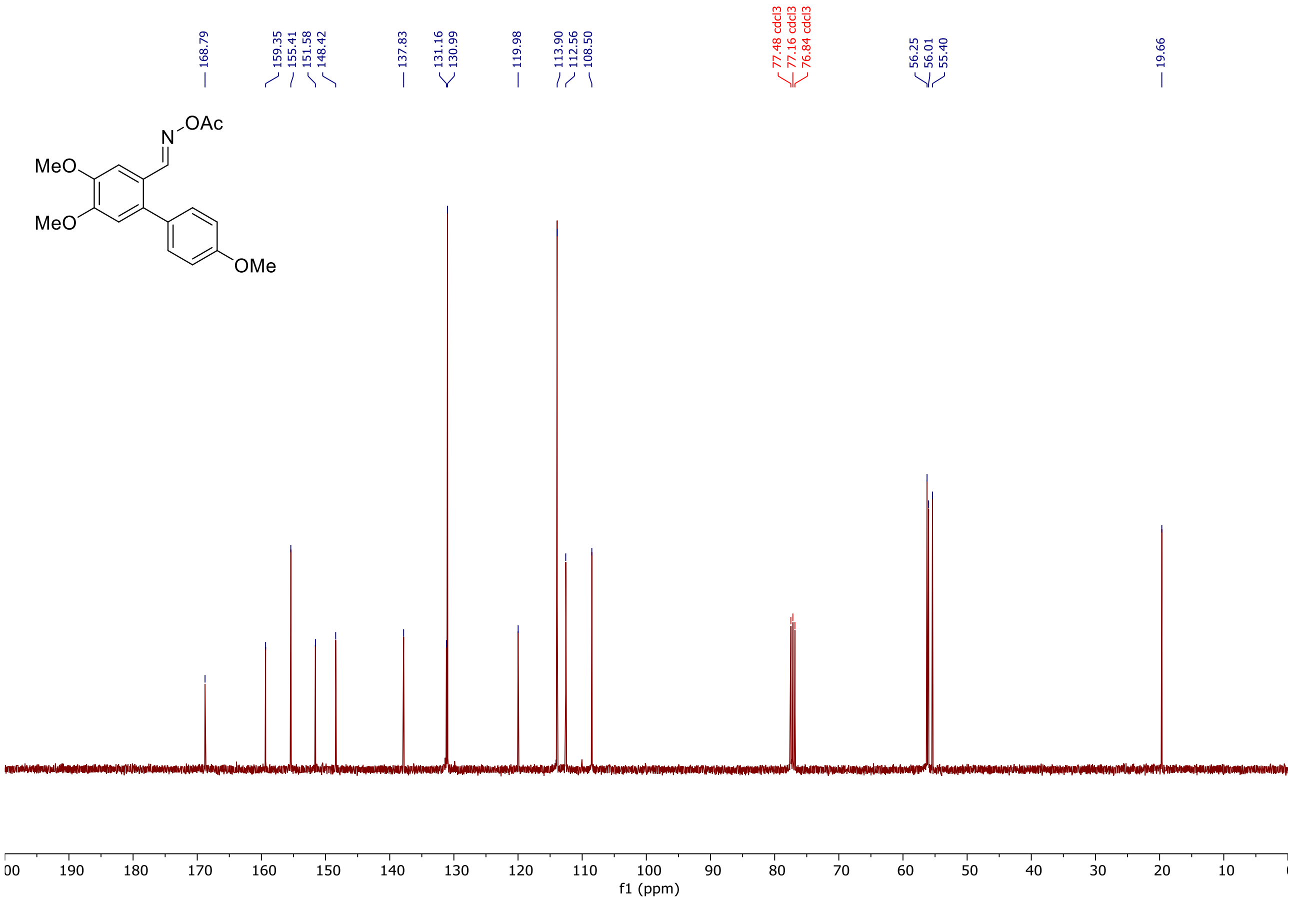

*3,8,9-Trimethoxyphenanthridine (**176**)*

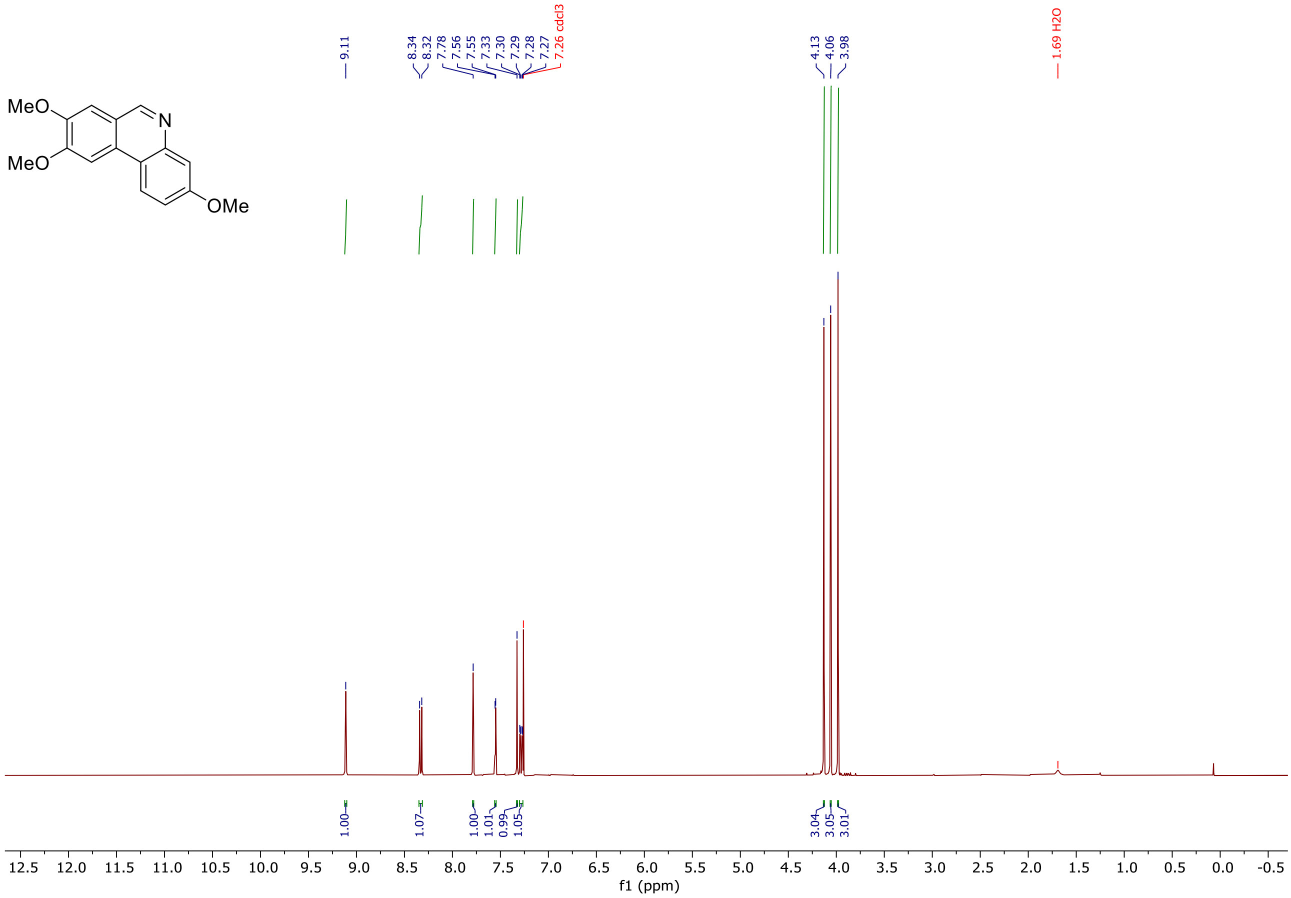

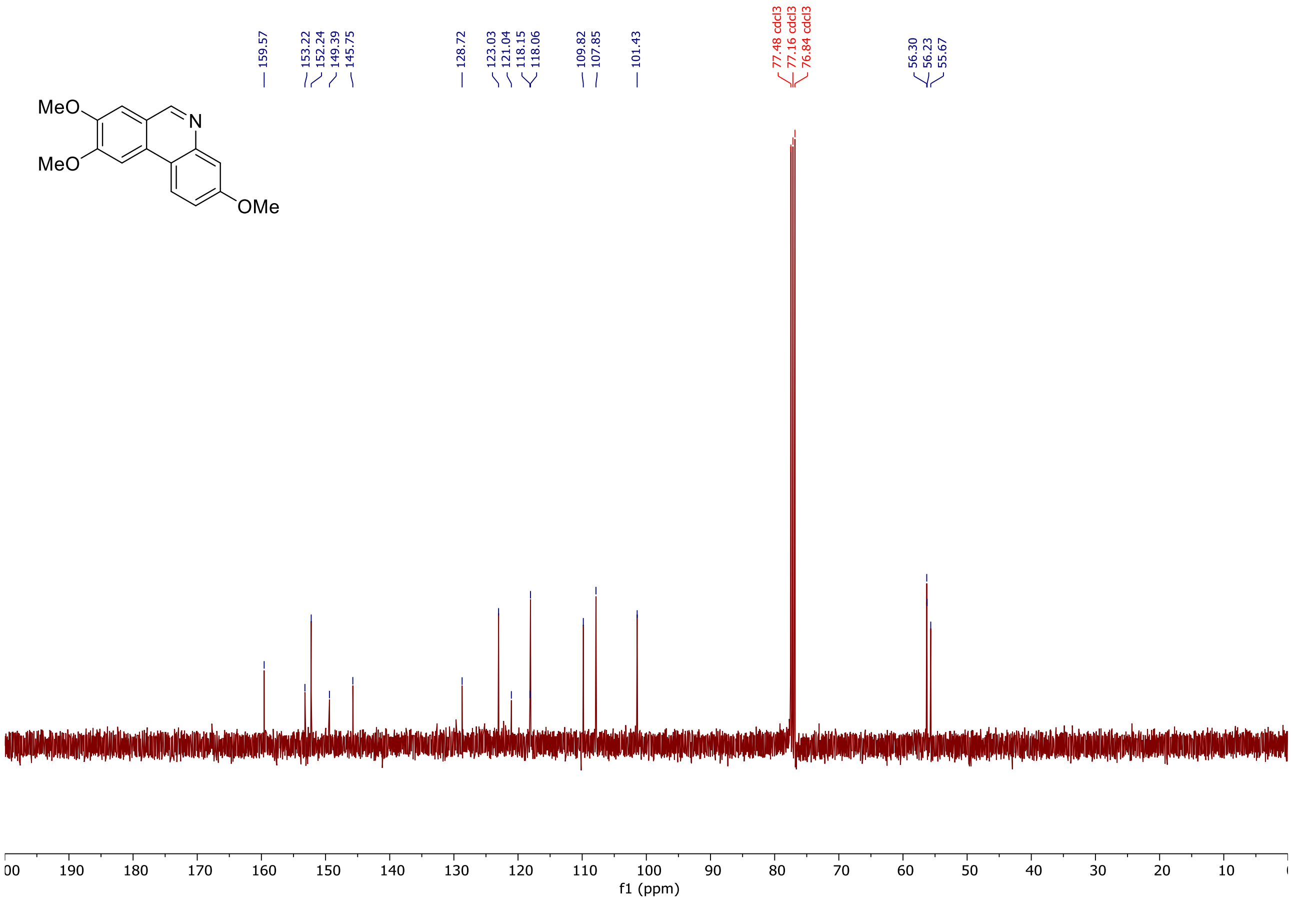

*Phenanthridine-3,8,9-triol (**179**)*

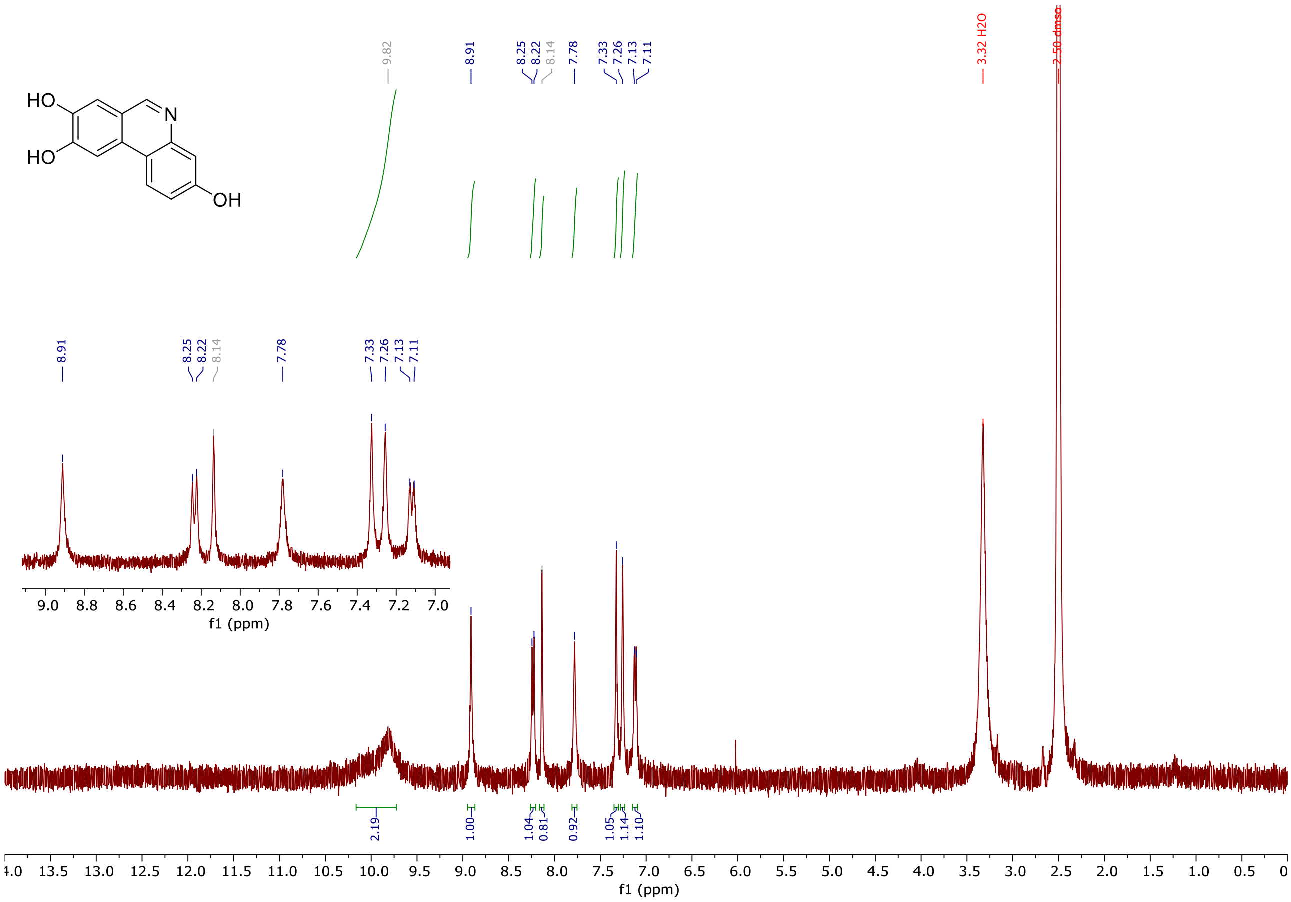

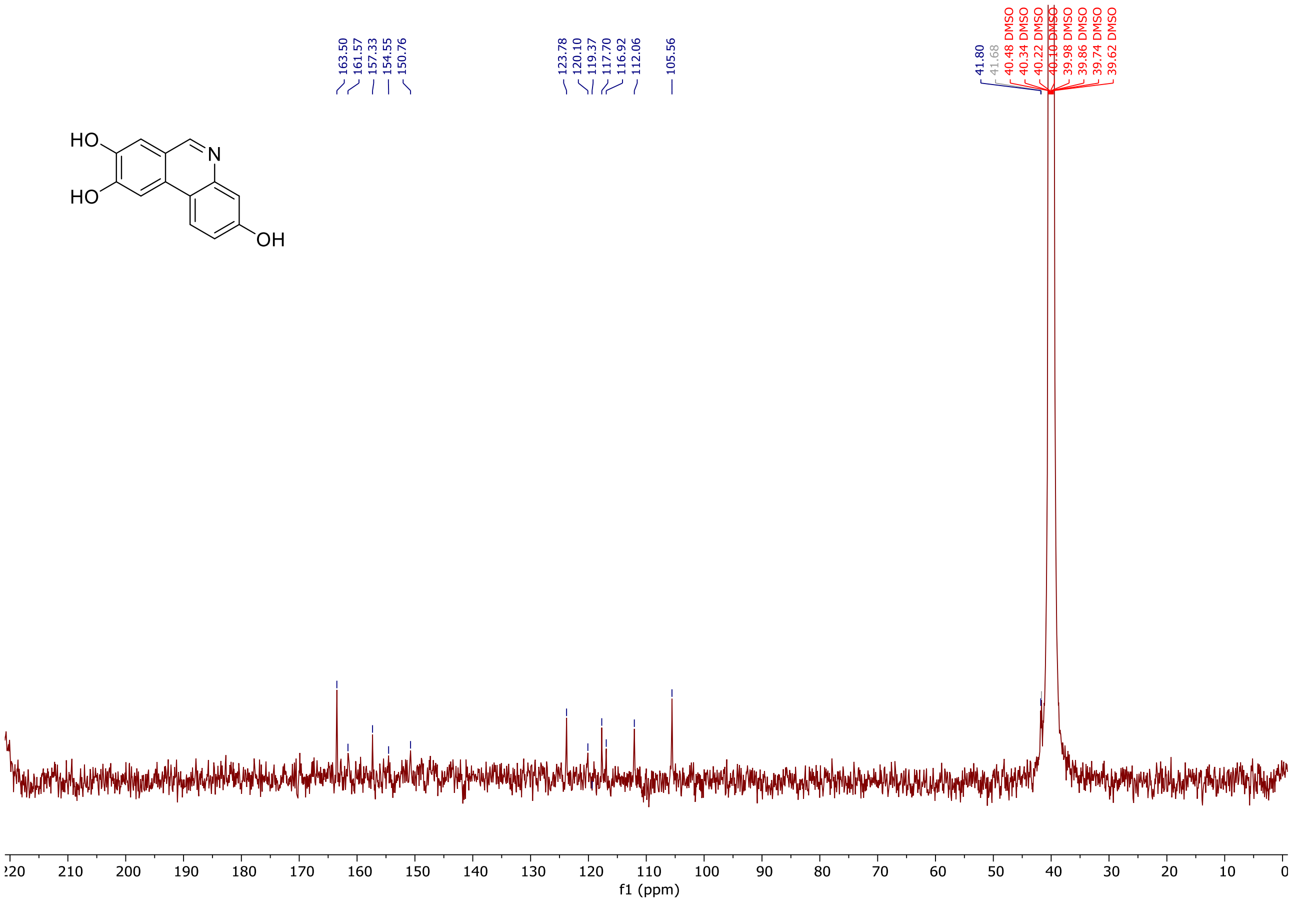

Bromo-5-methoxybenzaldehyde (**169**)

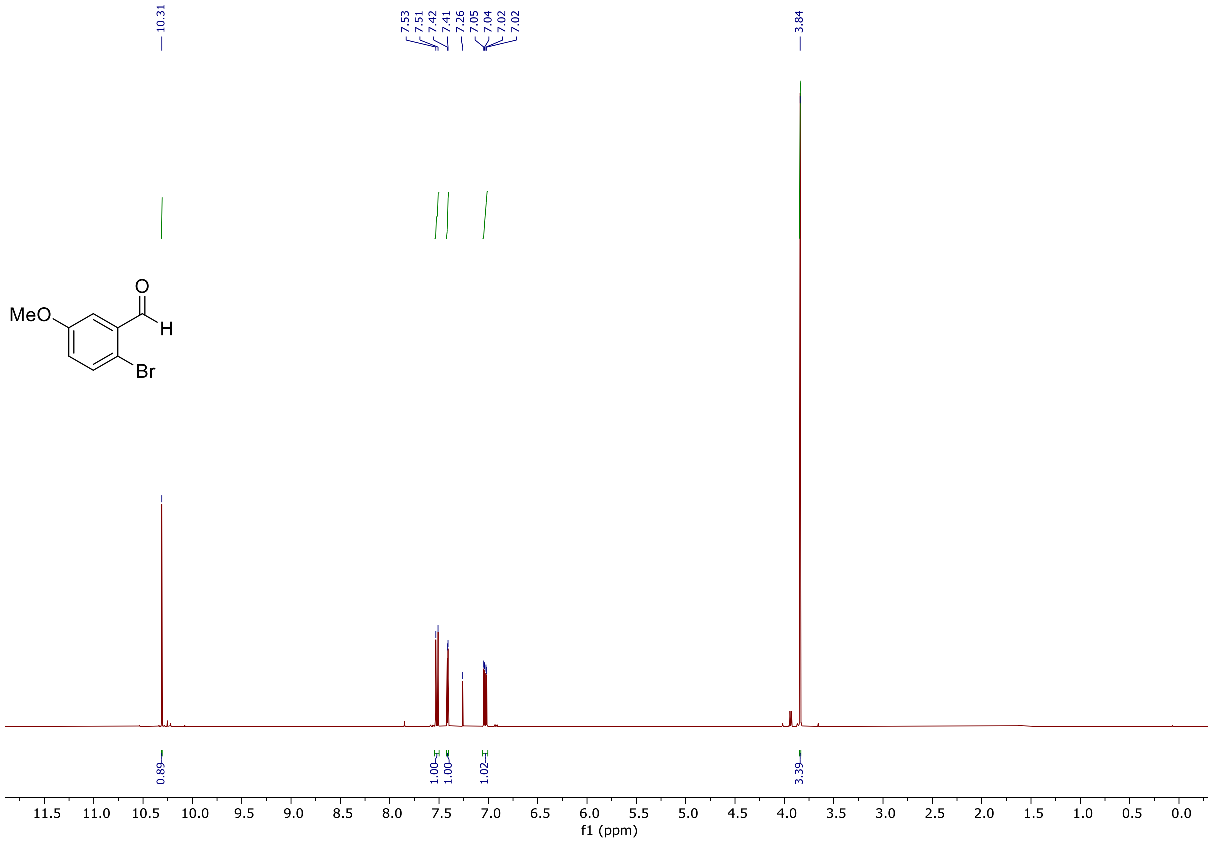

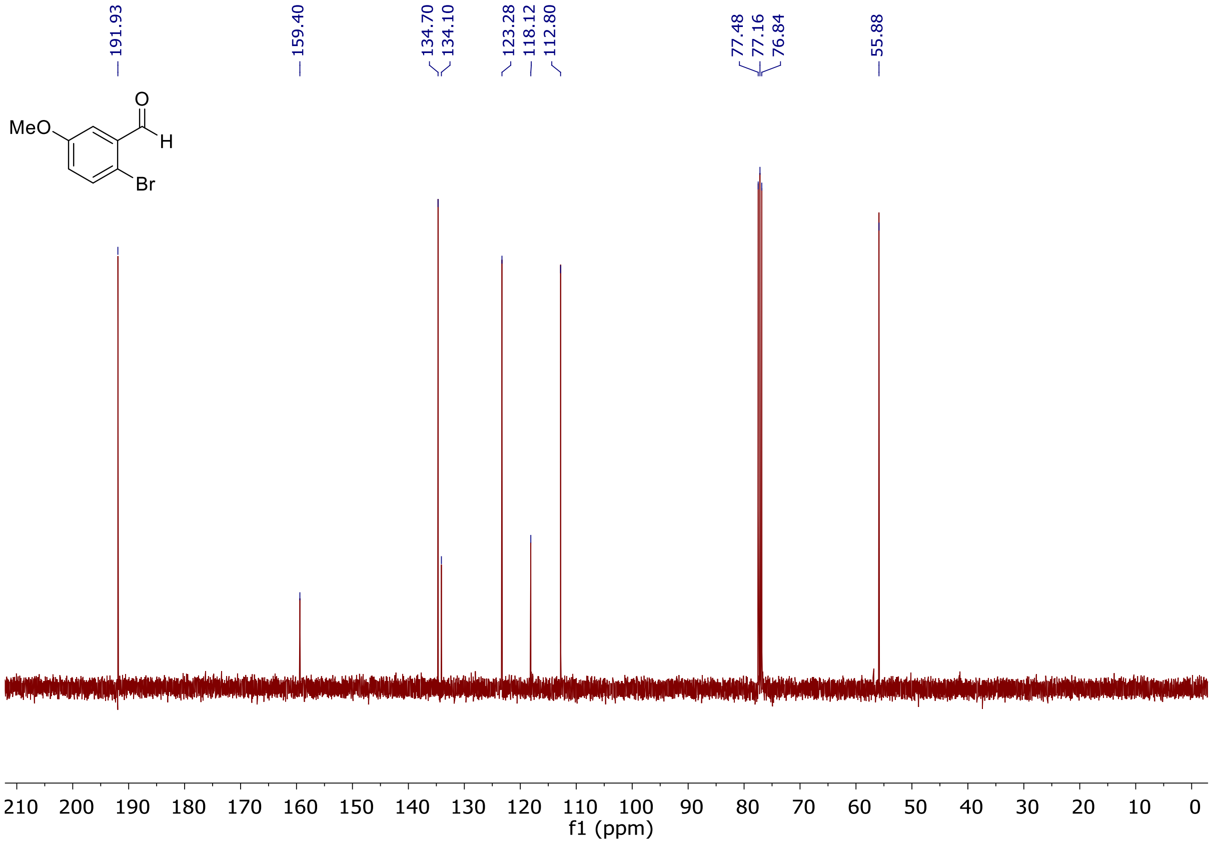

3',4,4'-Trimethoxy-[1,1'-biphenyl]-2-carbaldehyde (**171**)

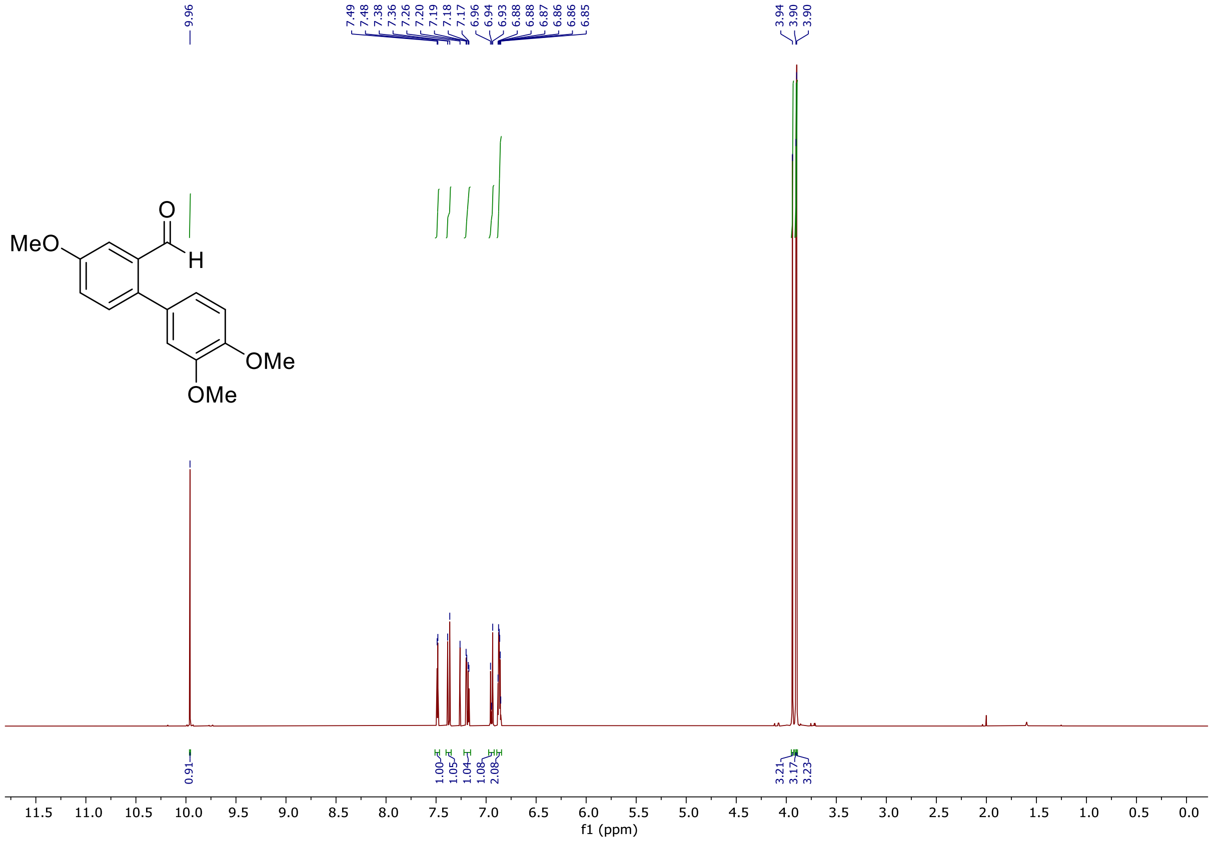

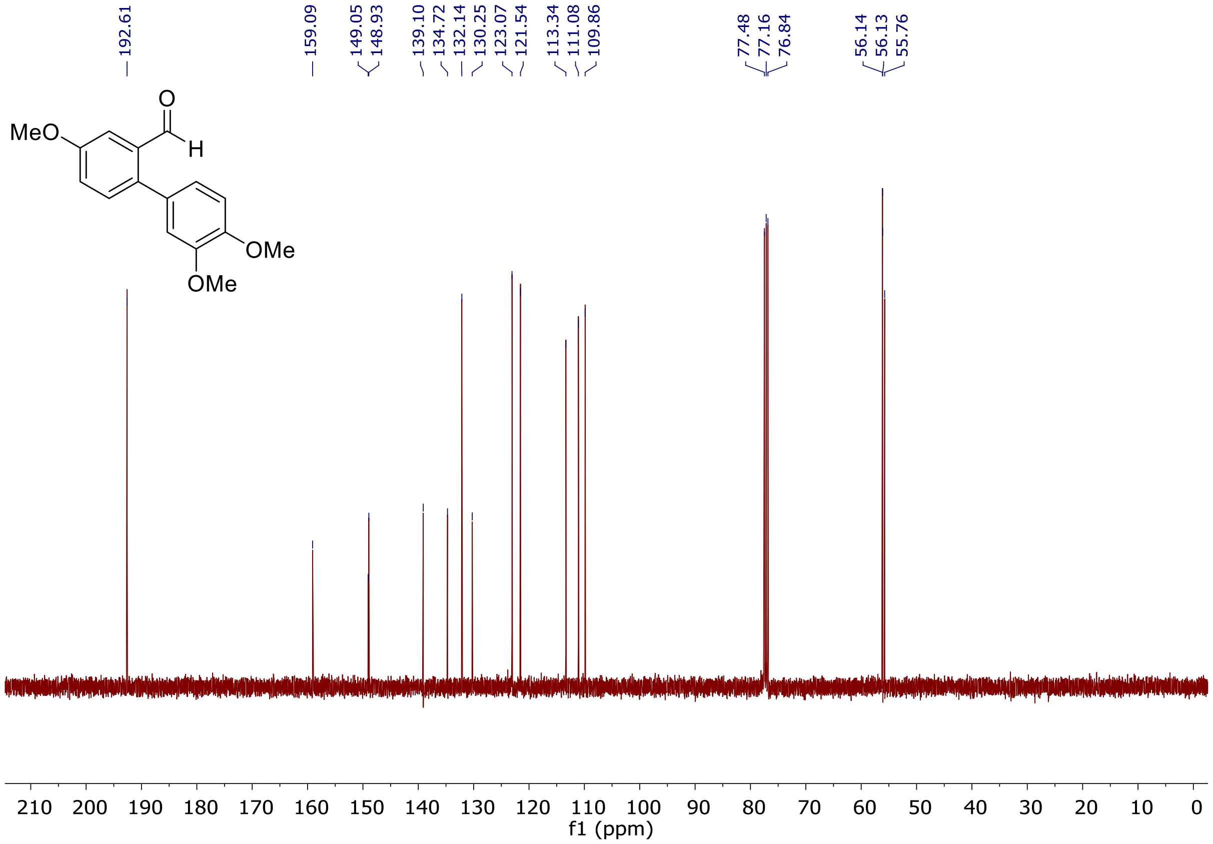

(E)-3',4,4'-trimethoxy-[1,1'-biphenyl]-2-carbaldehyde O-acetyl oxime (**174**)

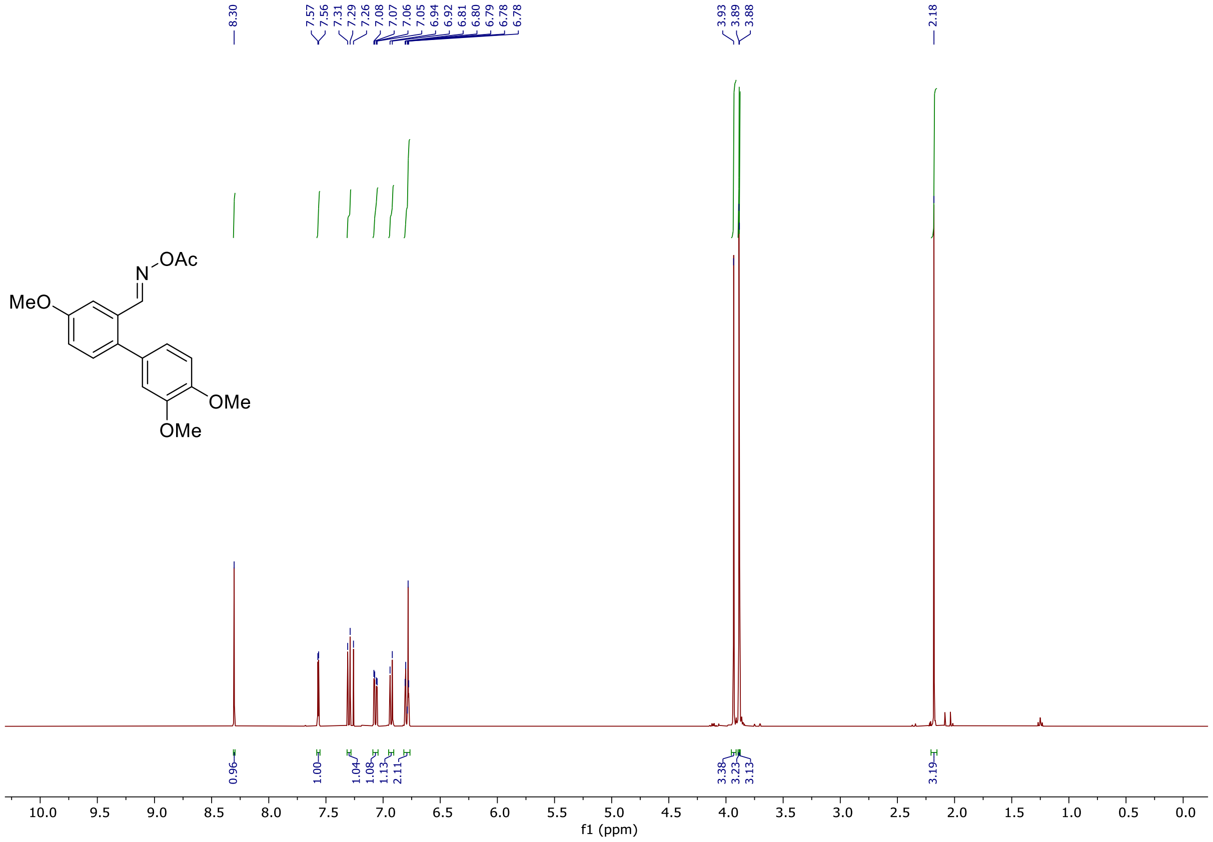

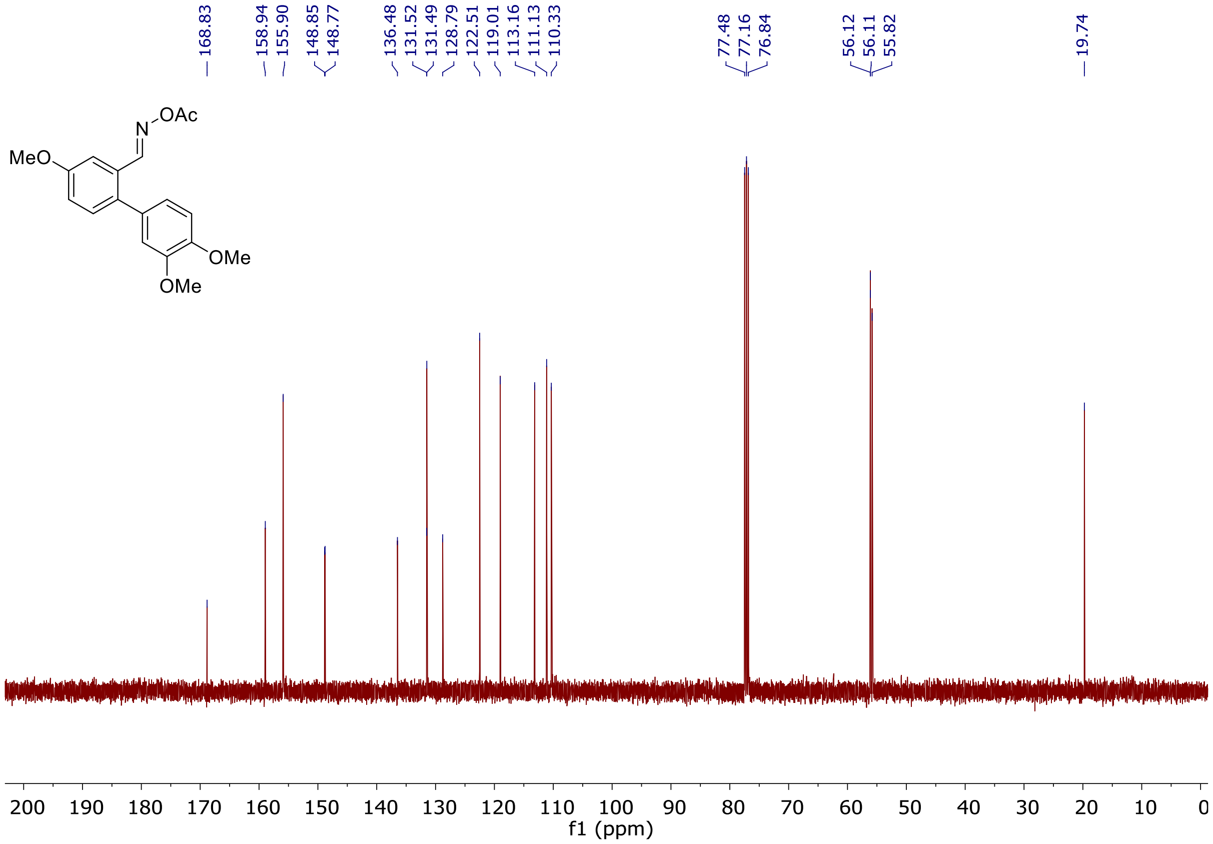

2,3,8-Trimethoxyphenanthridine (**177**)

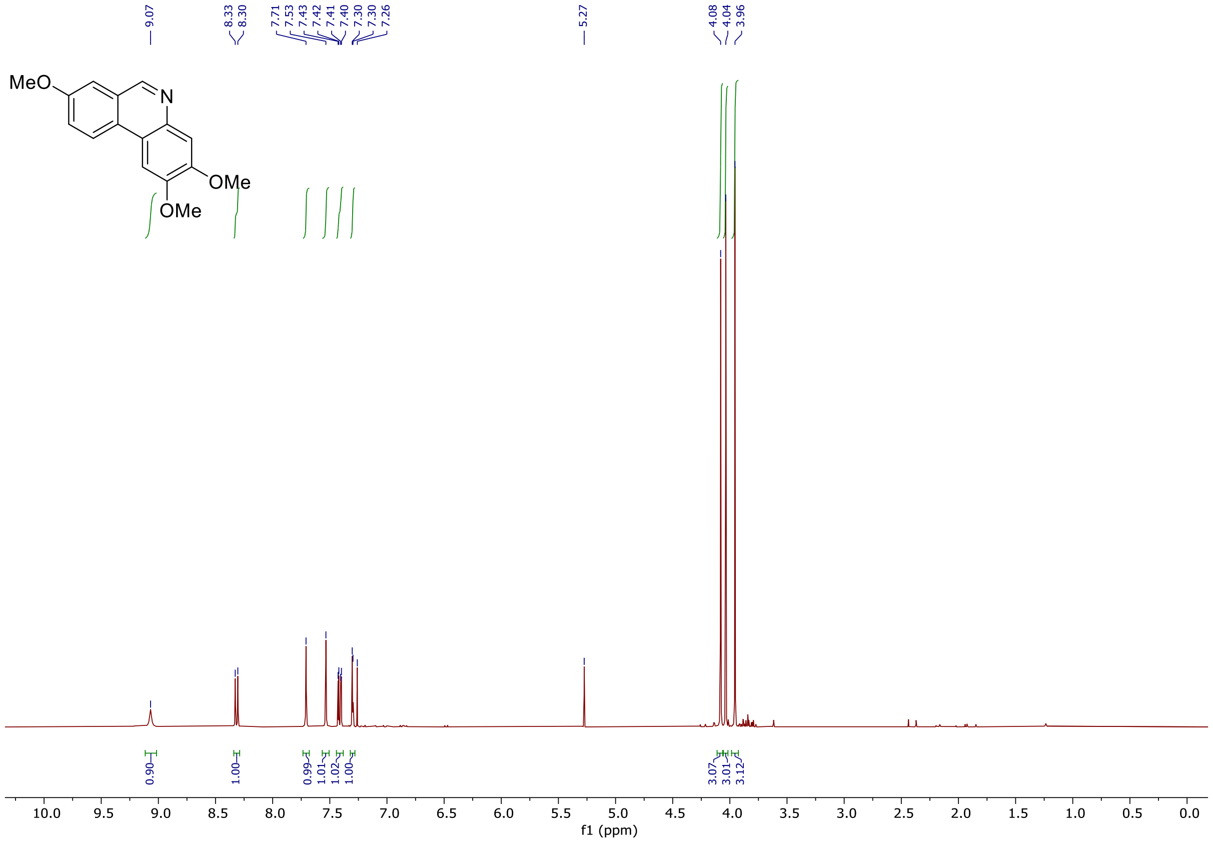

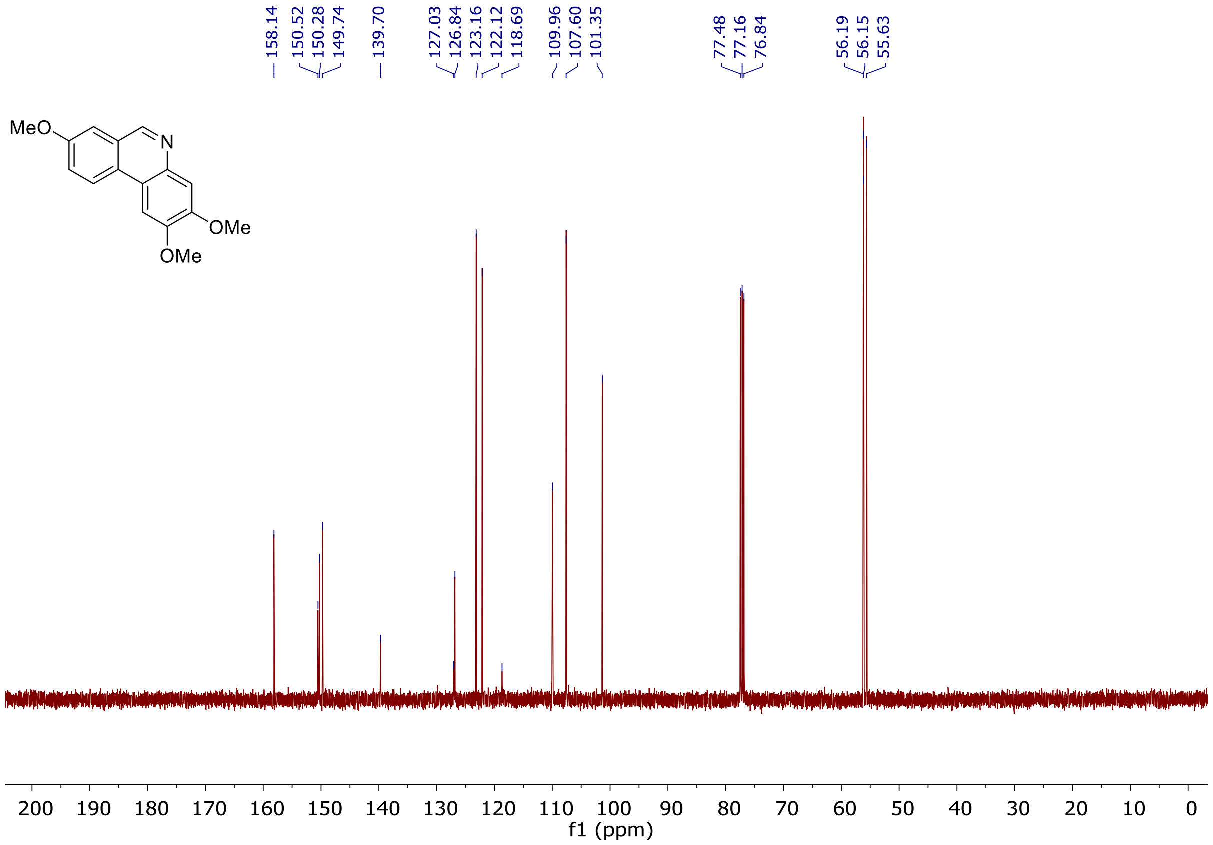

Phenanthridine-2,3,8-triol (**180**)

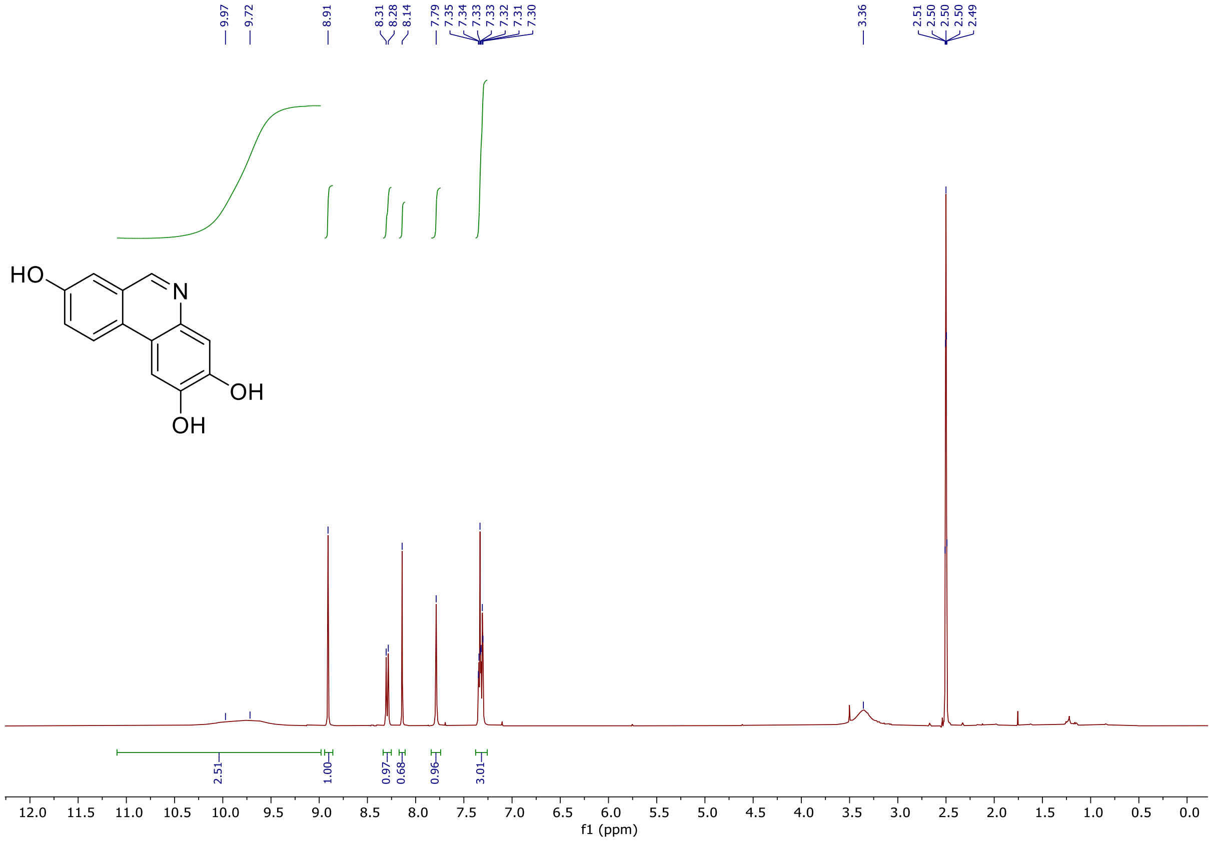

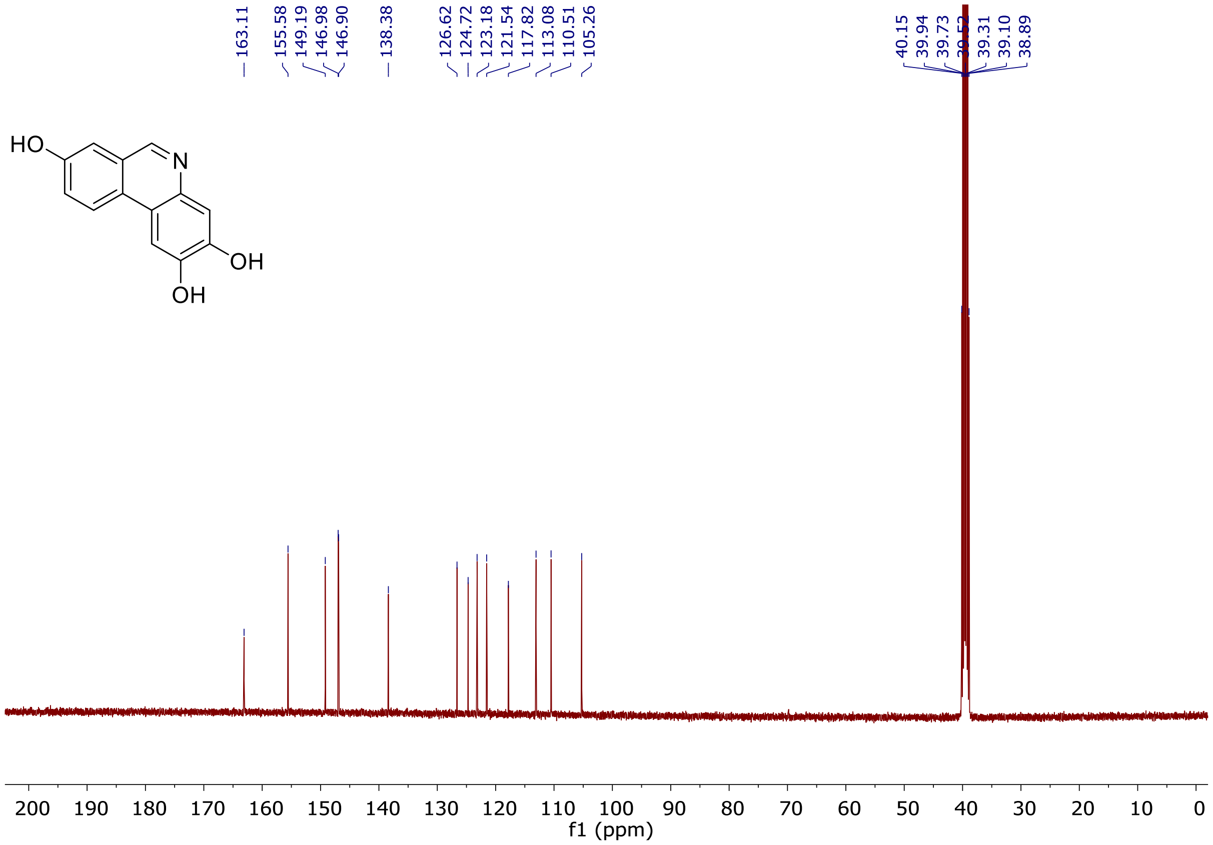

3,8,9-tris(benzyloxy)-6H-benzo[c]chromen-6-one (**181**)

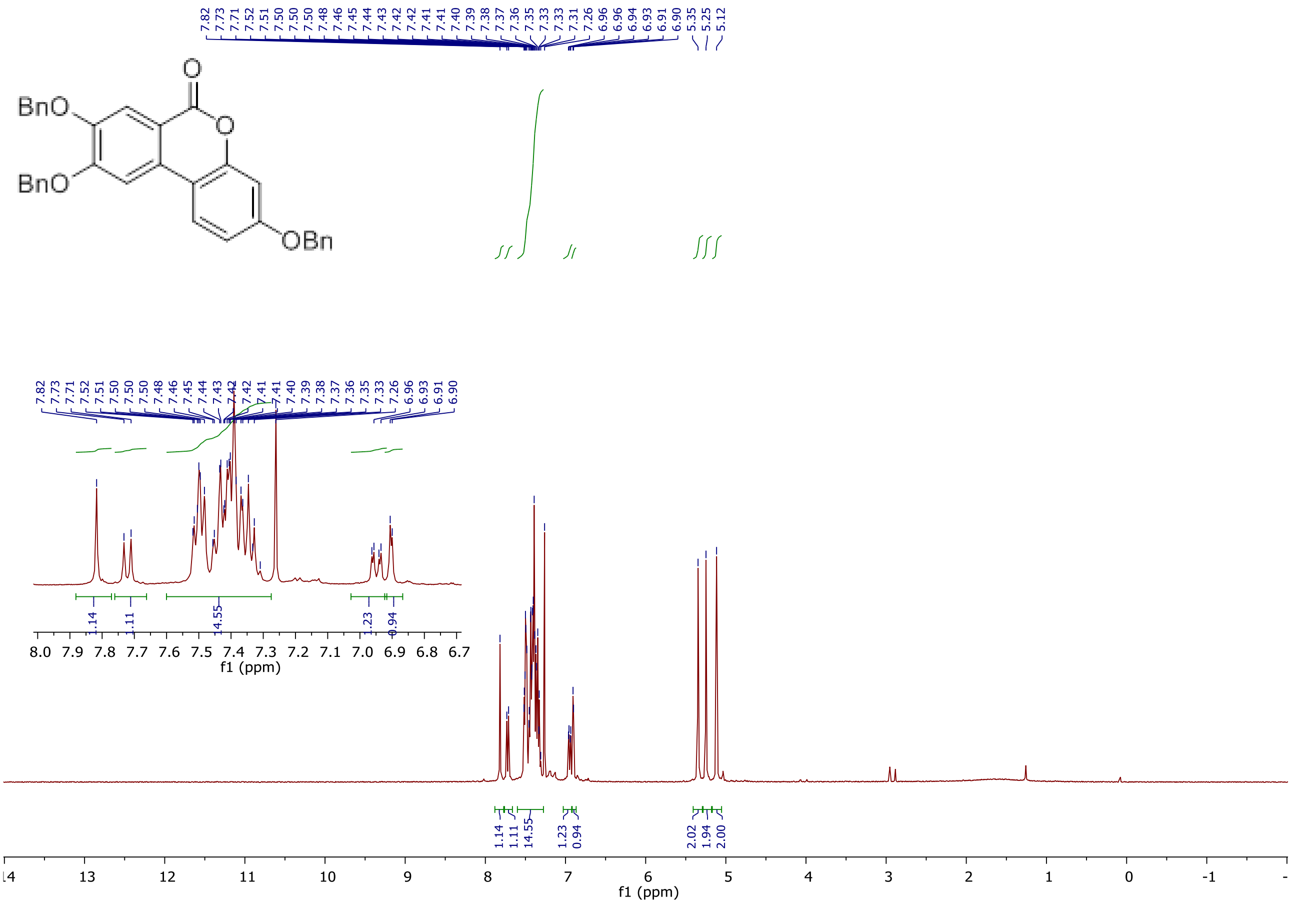

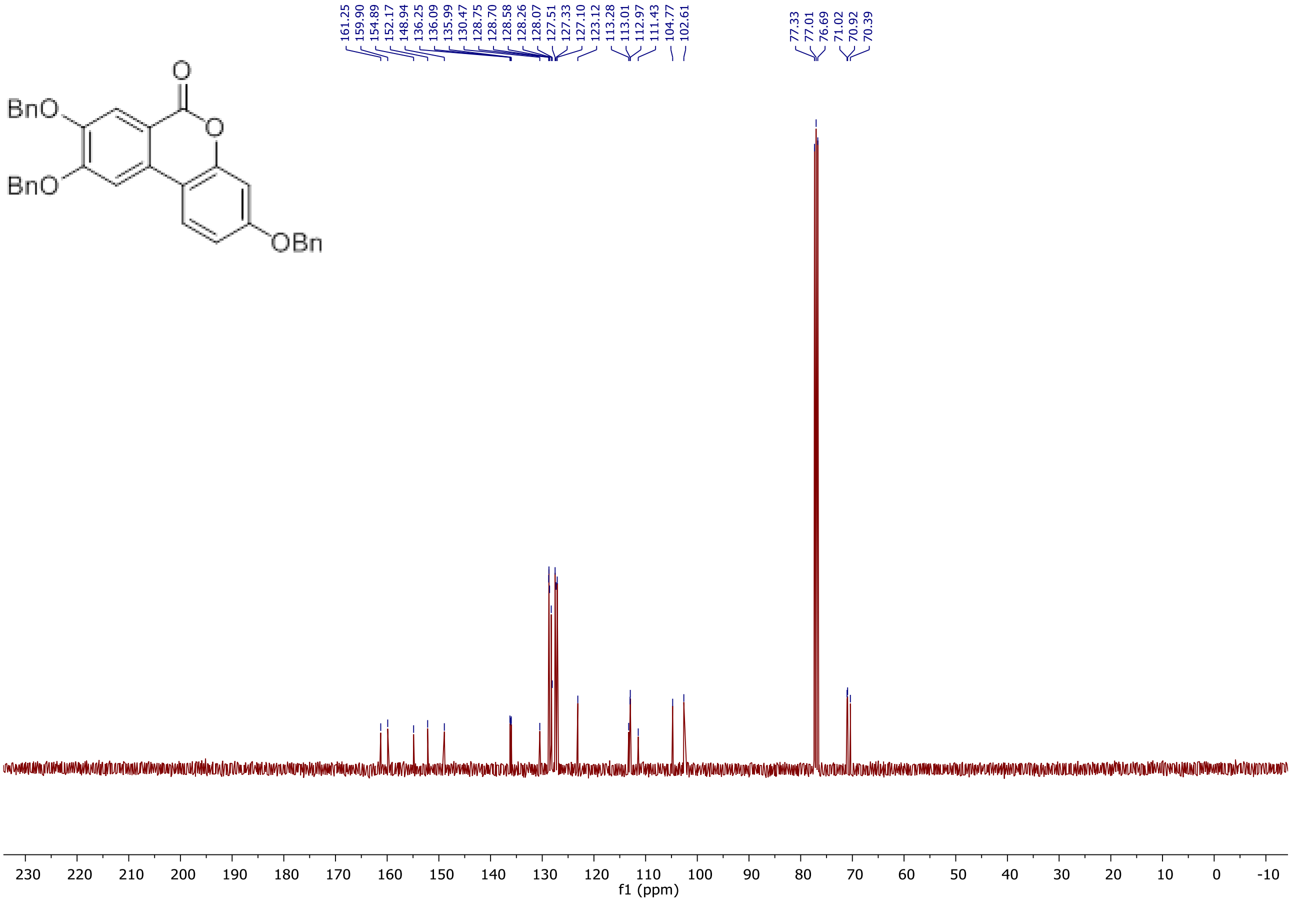

3,8,9-Tris(benzyloxy)-6,6-dimethyl-6H-benzo[c]chromene (**182**)

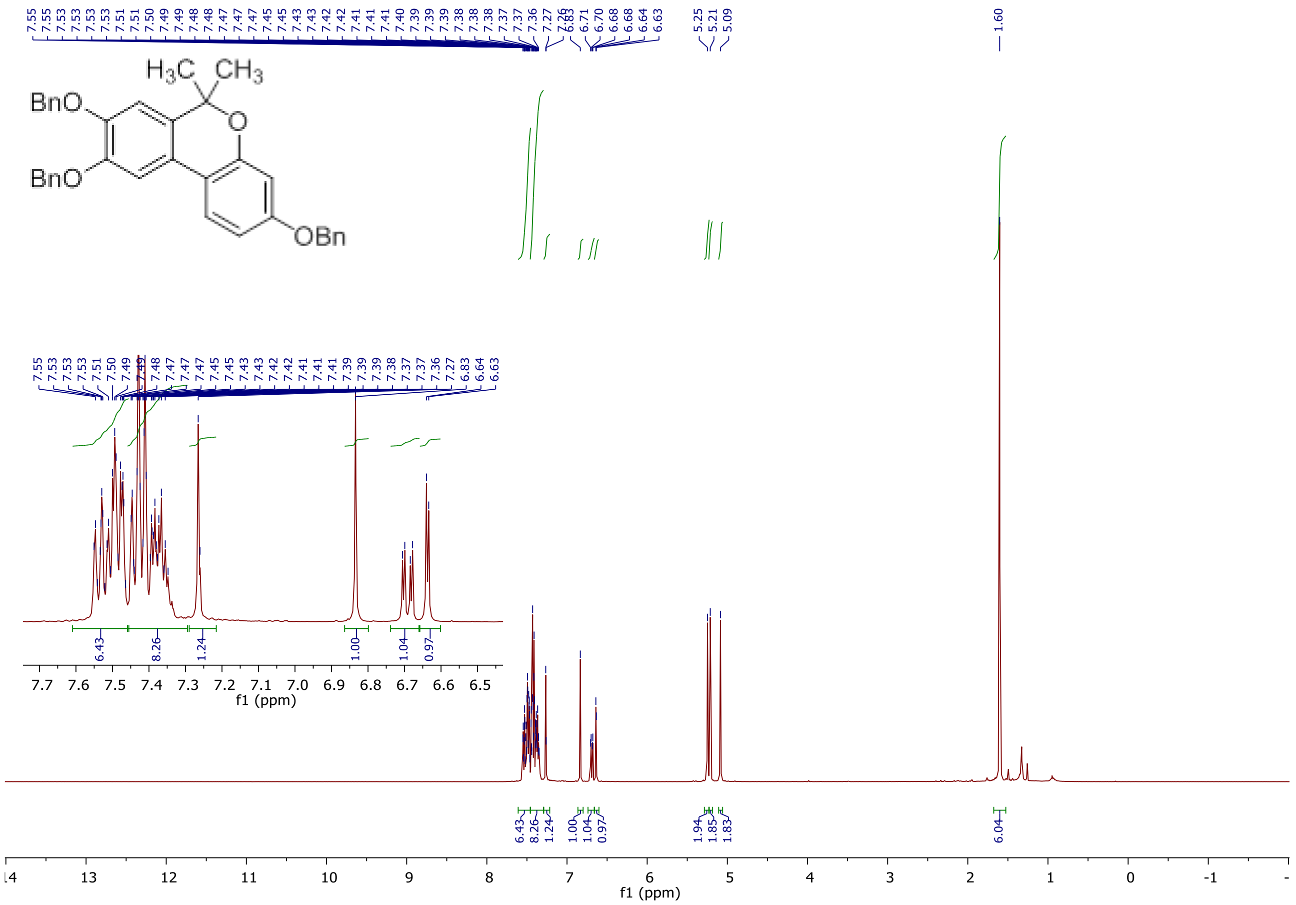

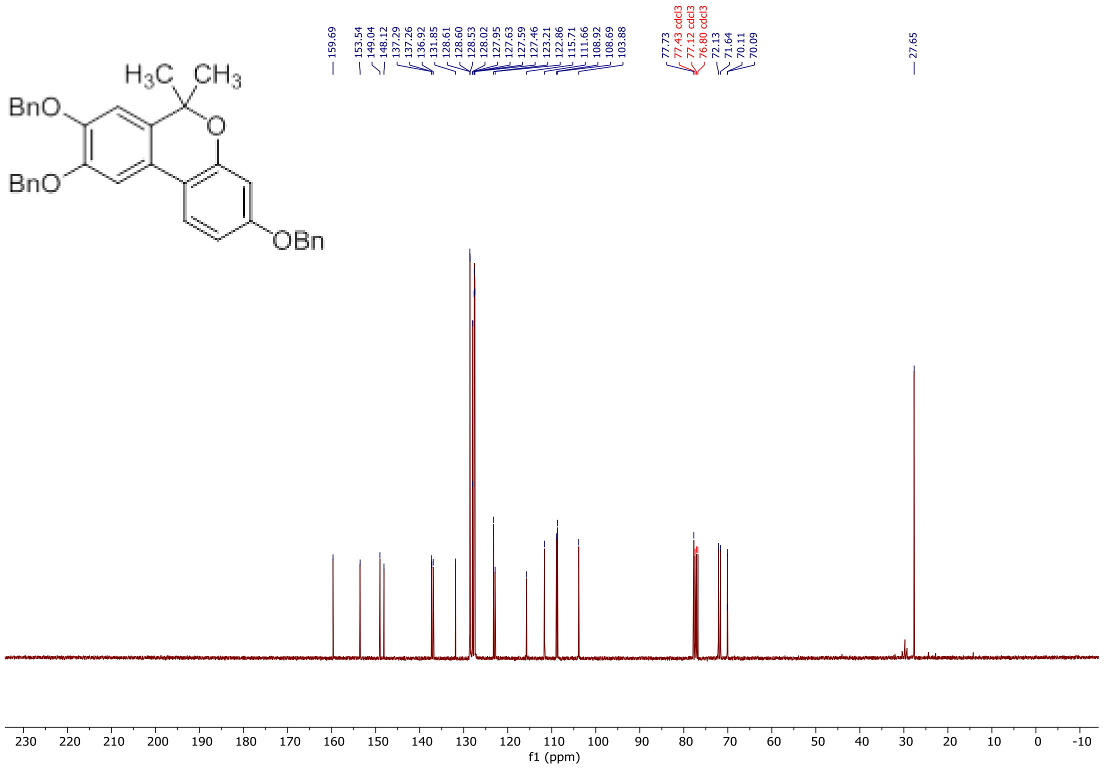

6,6-dimethyl-6H-benzo[c]chromene-3,8,9-triol (**183**)

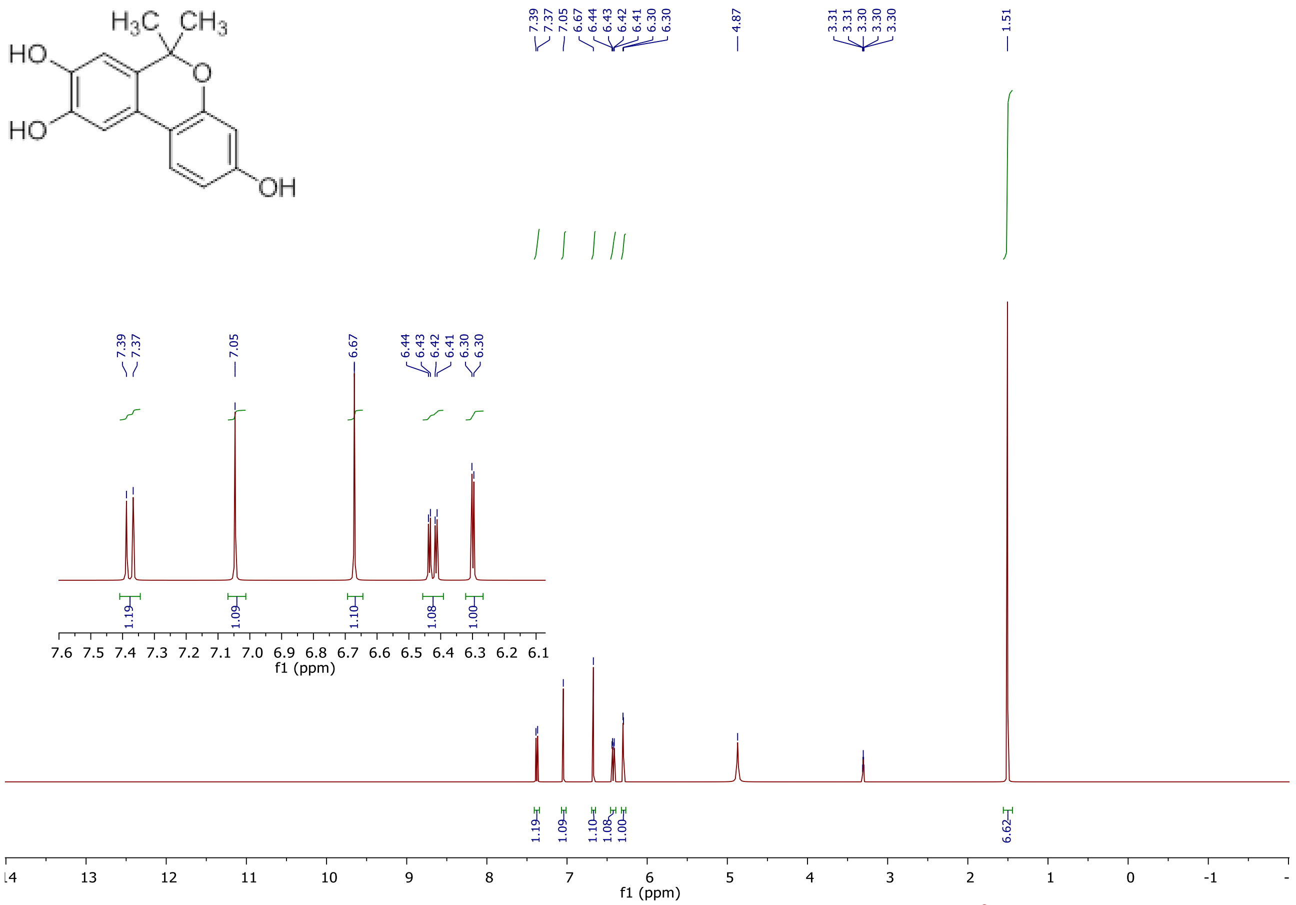

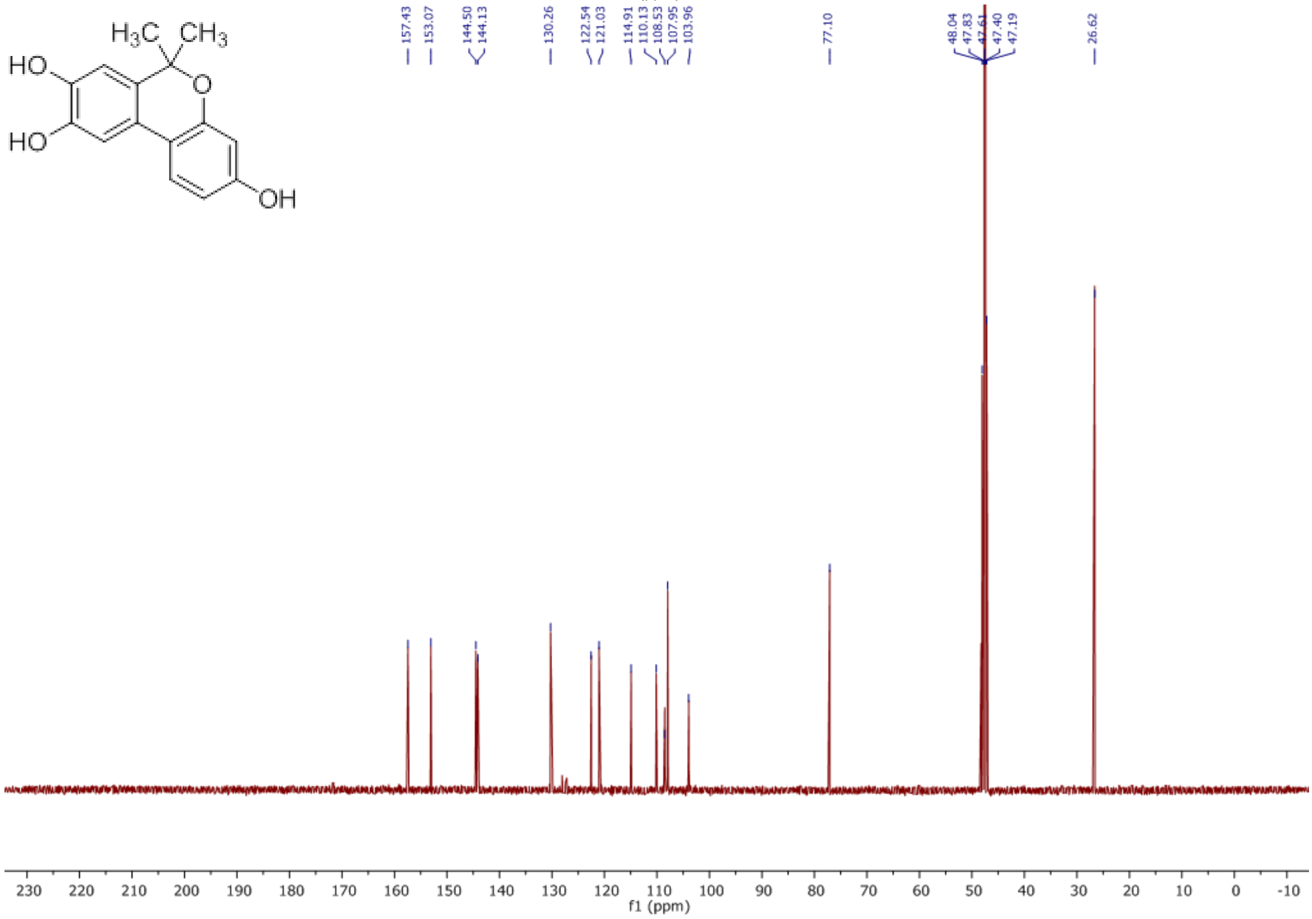

Methyl 4,4',5-trimethoxy-[1,1'-biphenyl]-2-carboxylate (**186**)

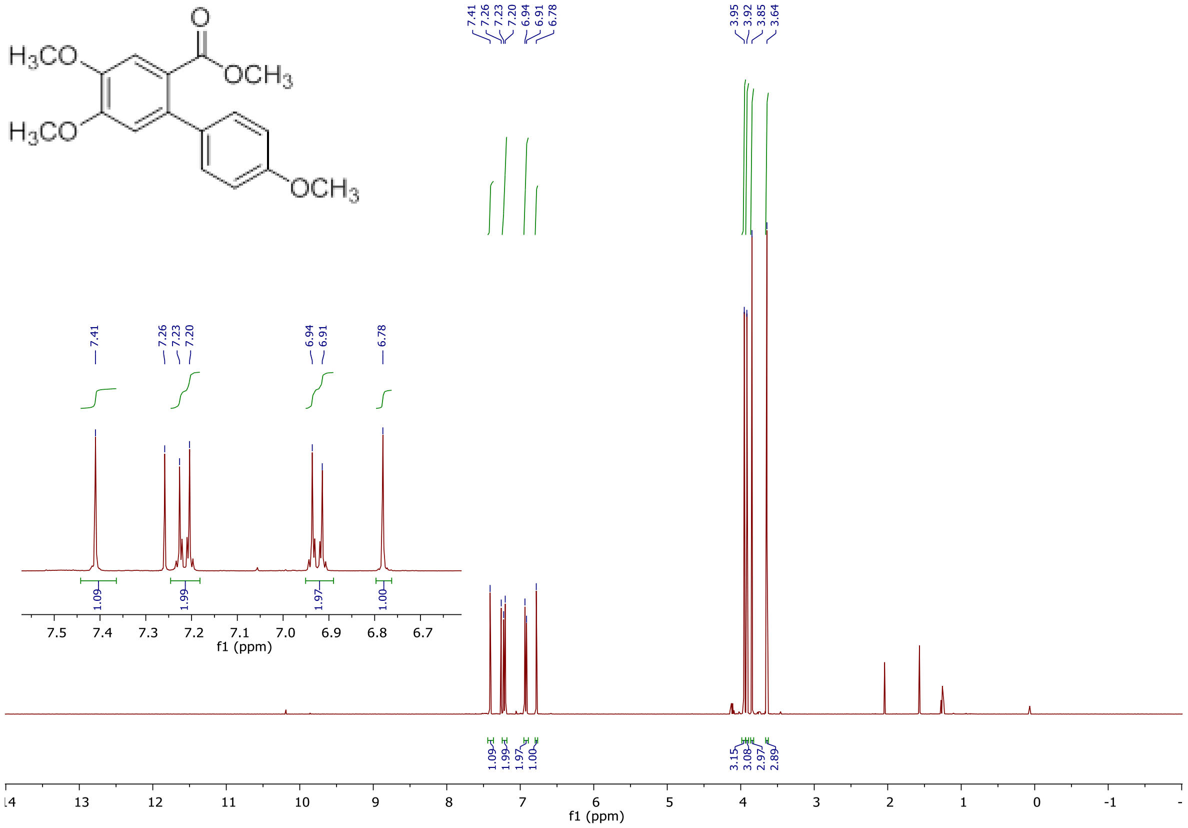

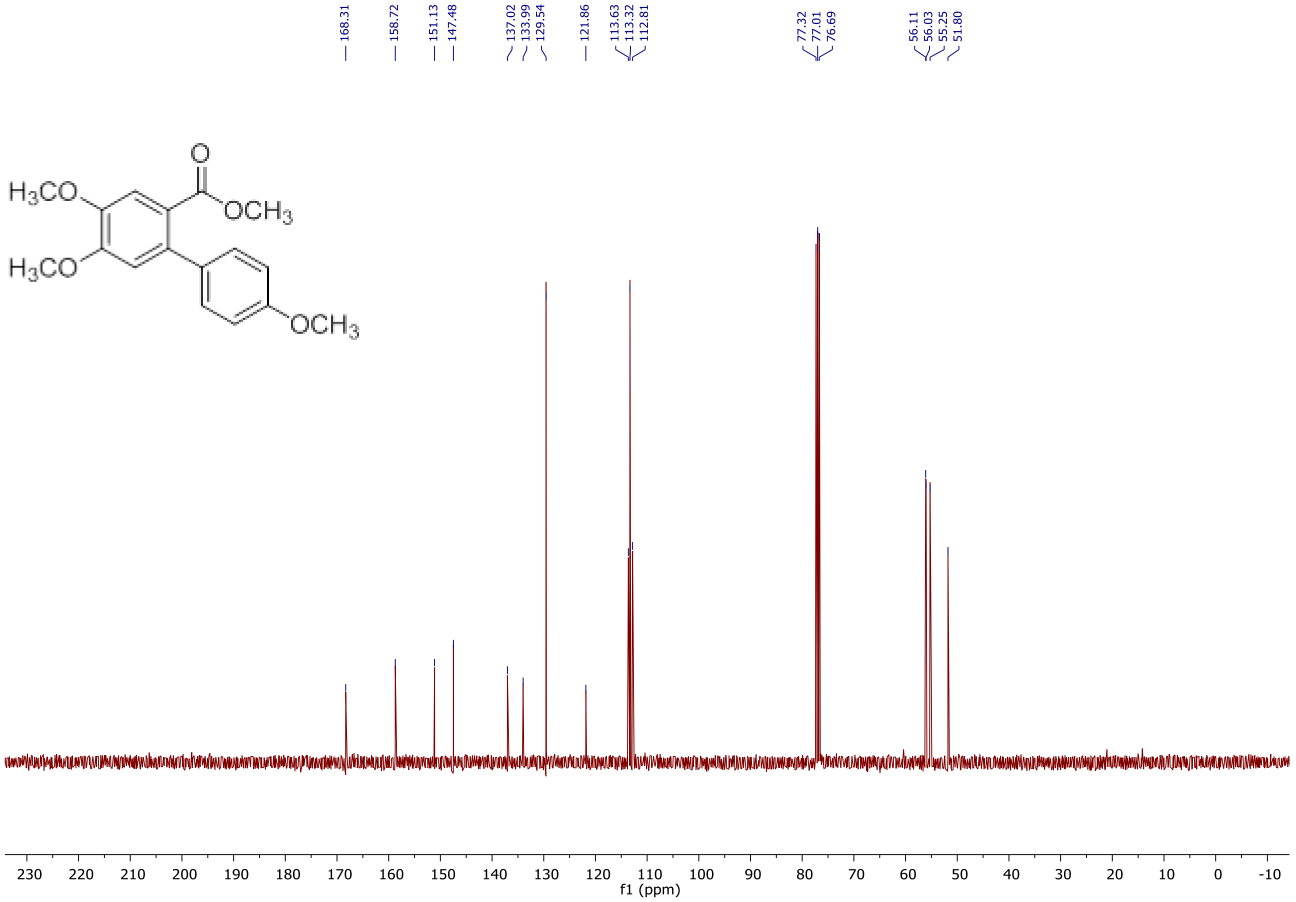

2,3,7-Trimethoxy-9H-fluoren-9-one (**191**)

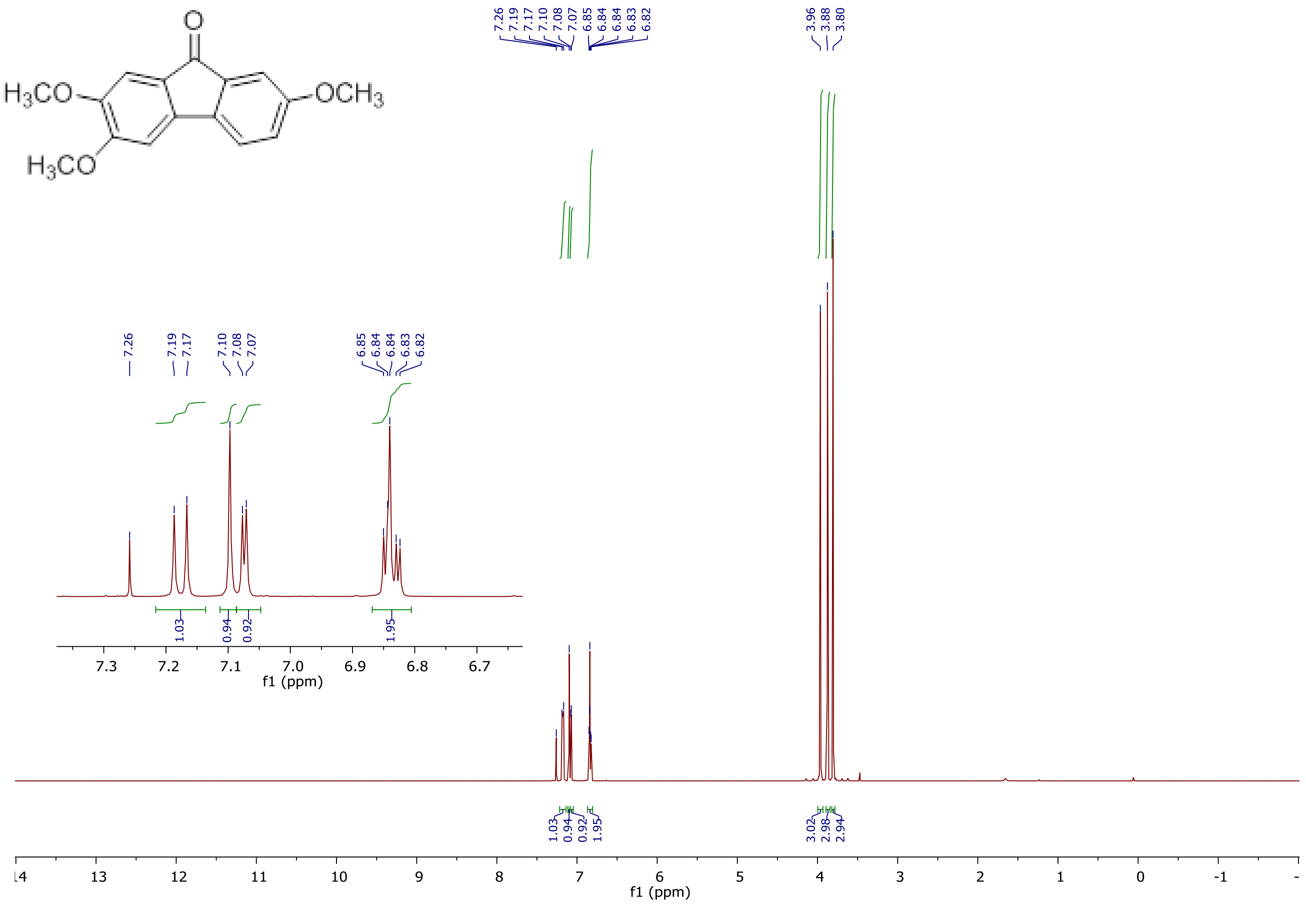

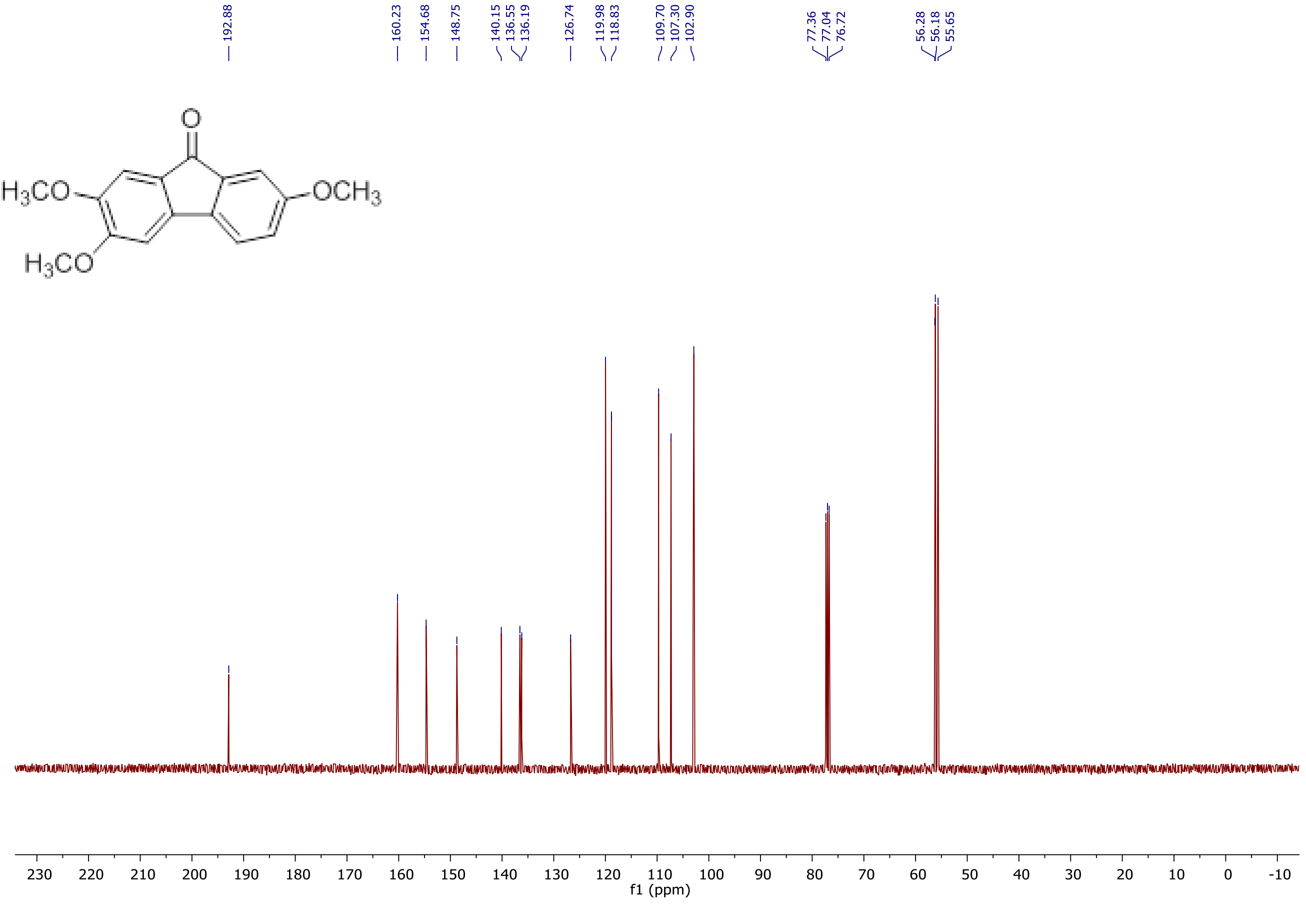

2,3,7-Trihydroxy-9H-fluoren-9-one (**192**)

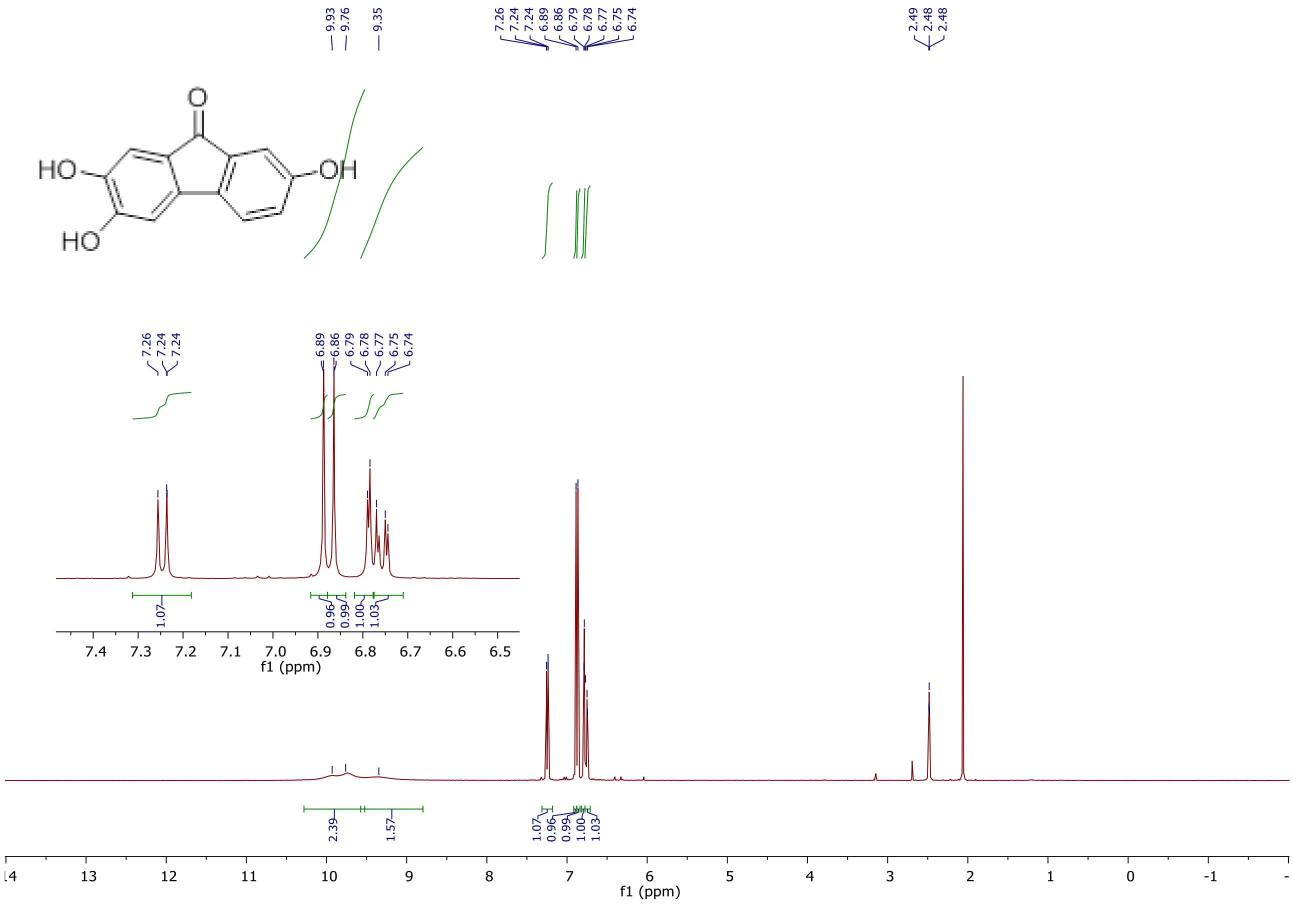

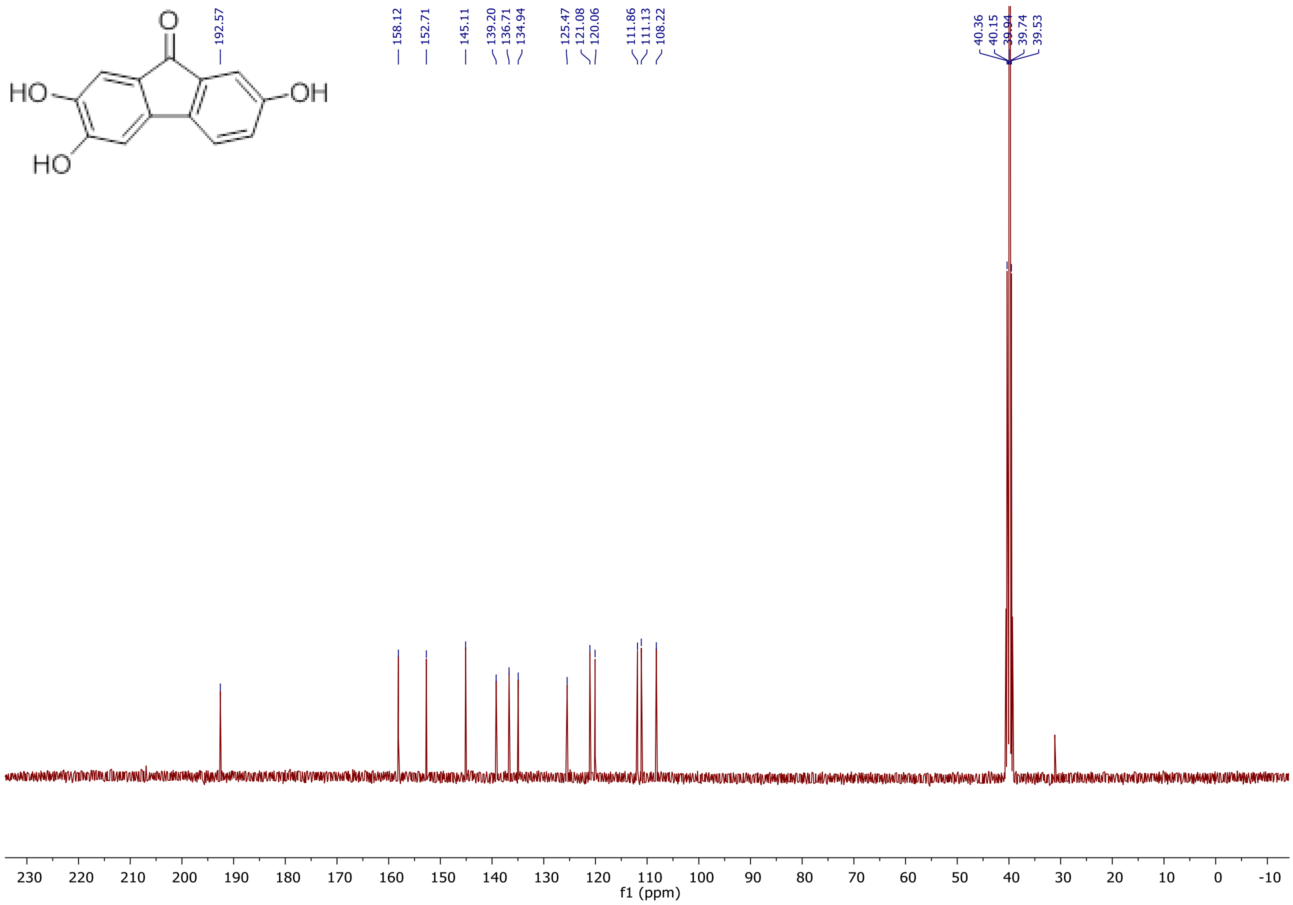

Methyl 3',4',5-trimethoxy-[1,1'-biphenyl]-2-carboxylate (**187**)

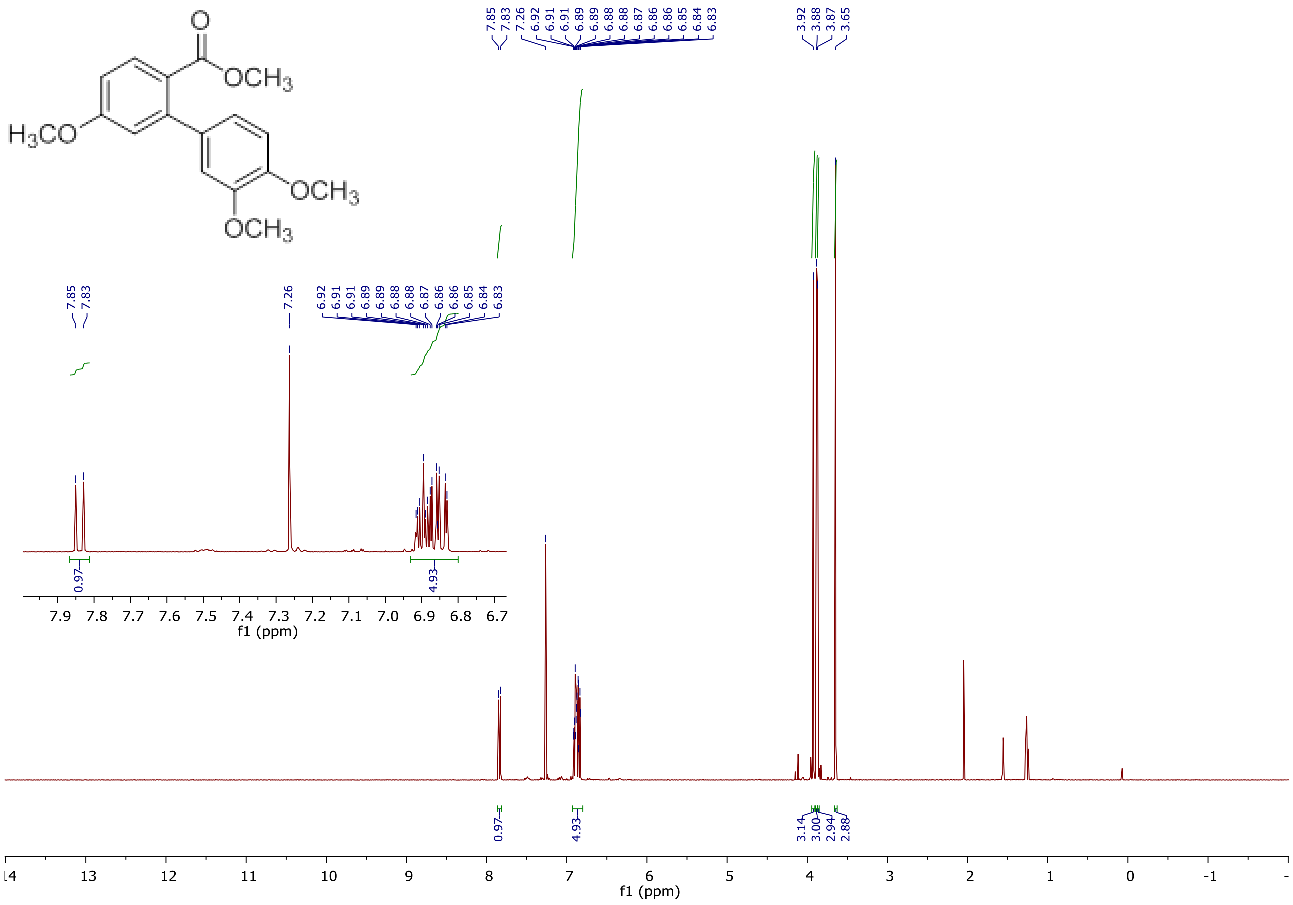

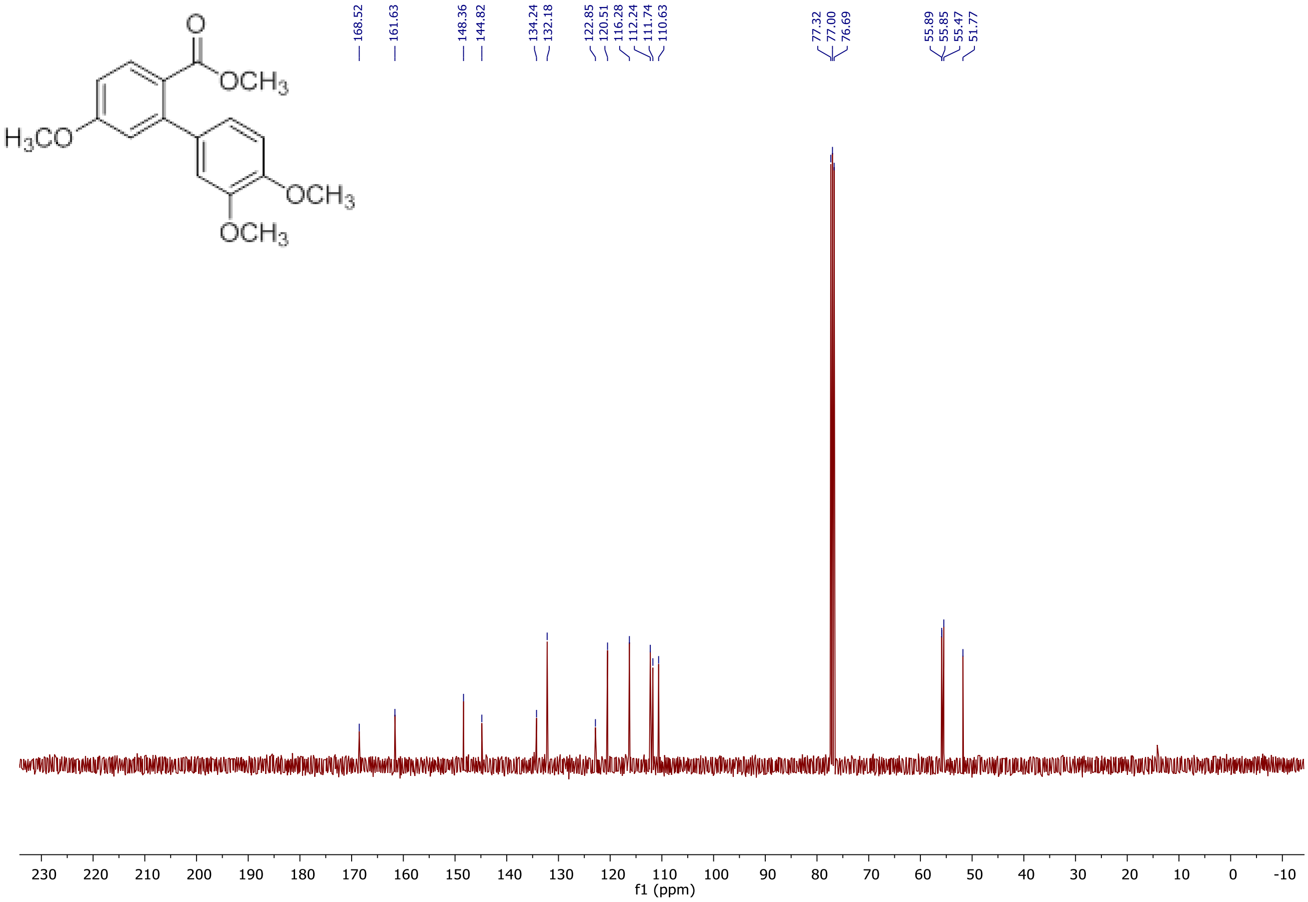

2,3,6-Trimethoxy-9H-fluoren-9-one (**193**)

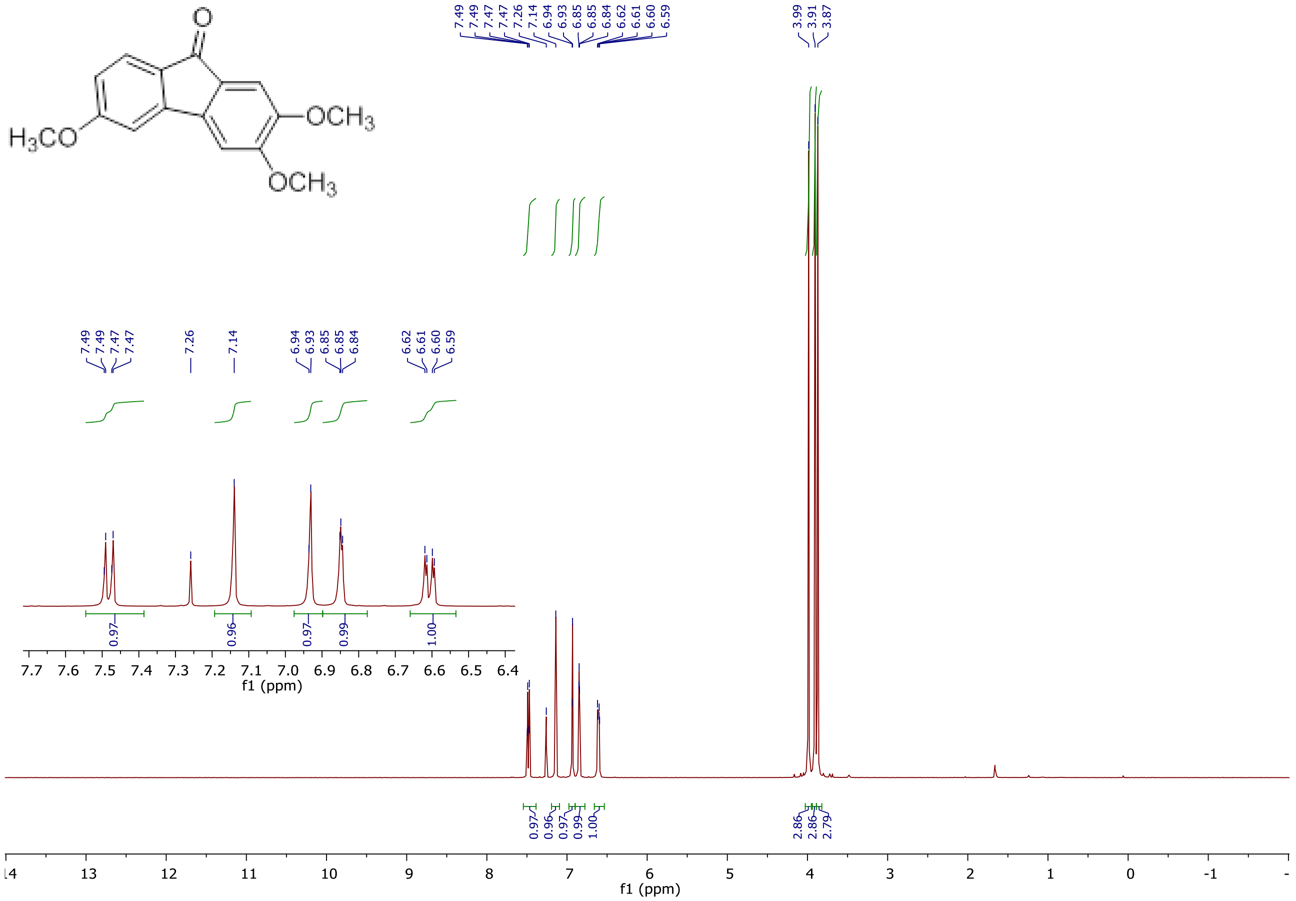

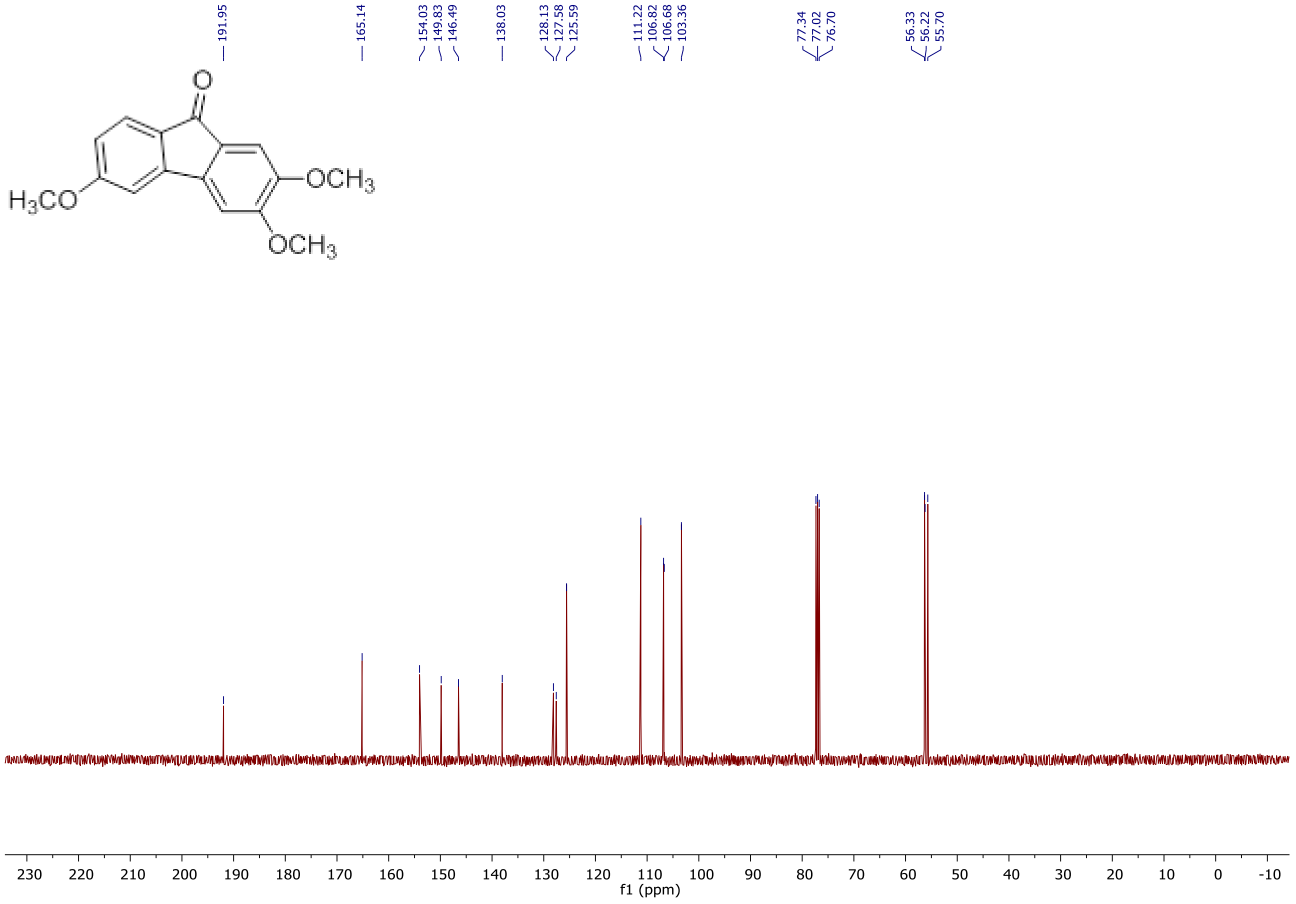

2,3,6-trihydroxy-9H-fluoren-9-one (**194**)

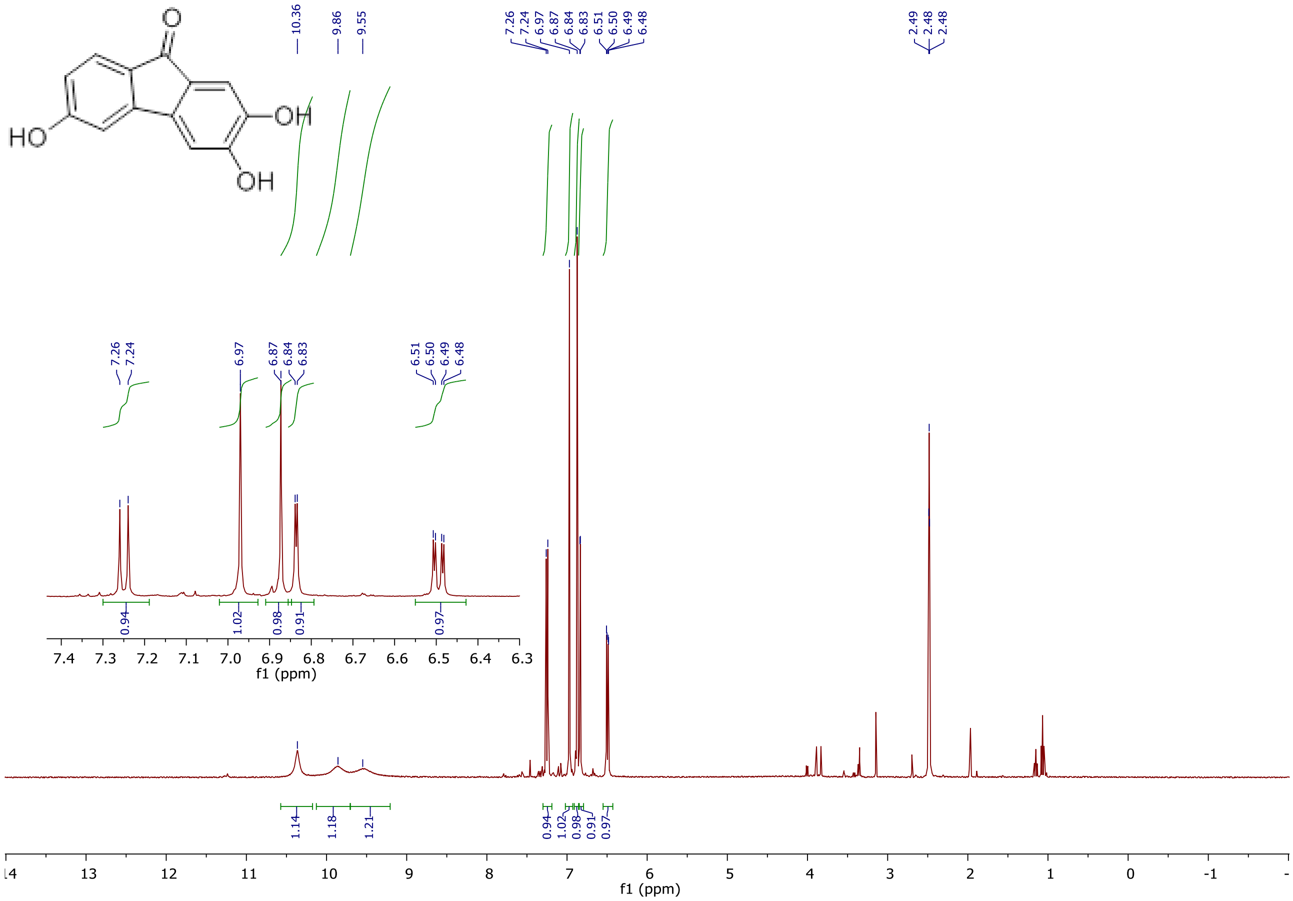

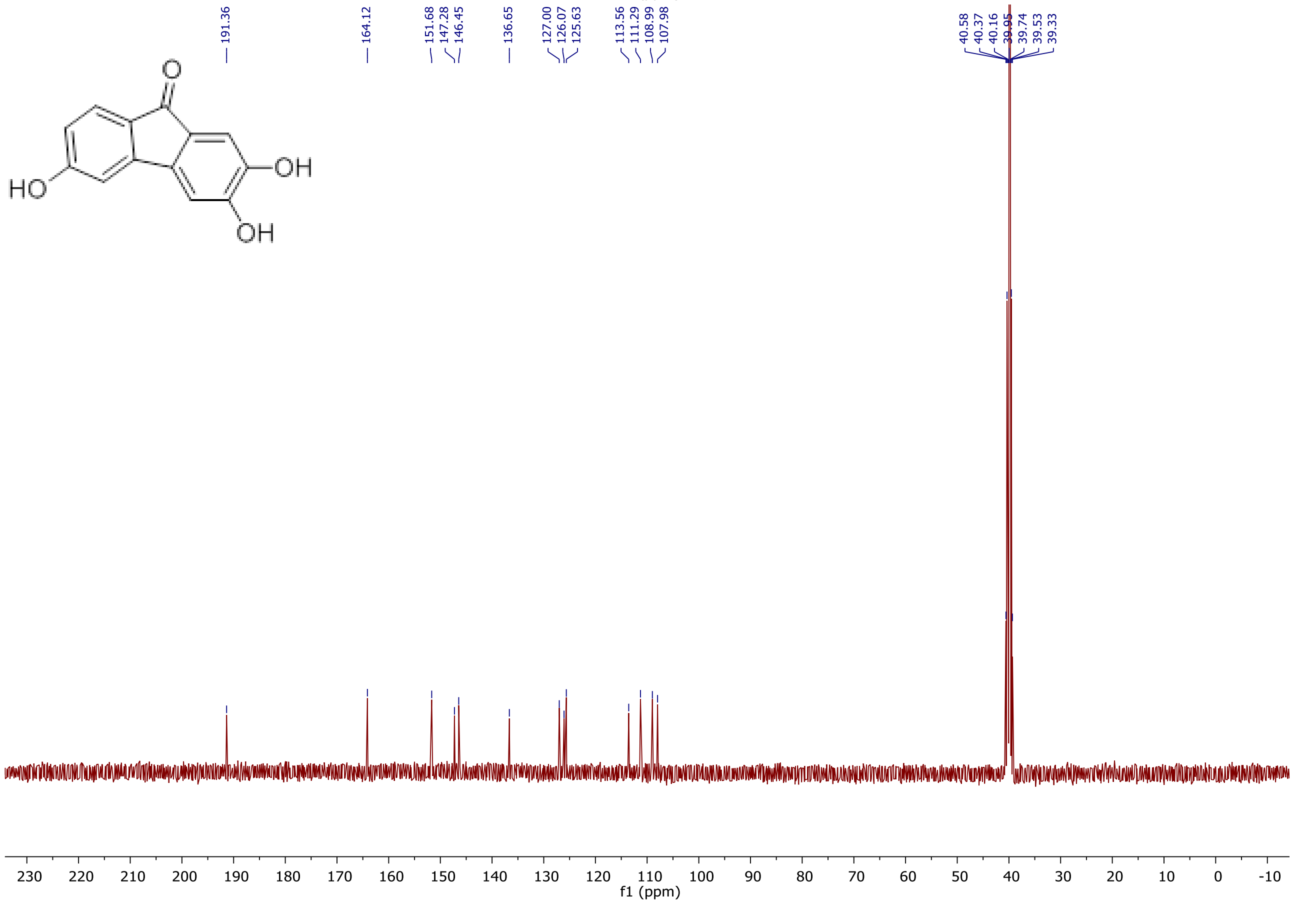

Methyl 3',4,4',5-tetramethoxy-[1,1'-biphenyl]-2-carboxylate (***188***)

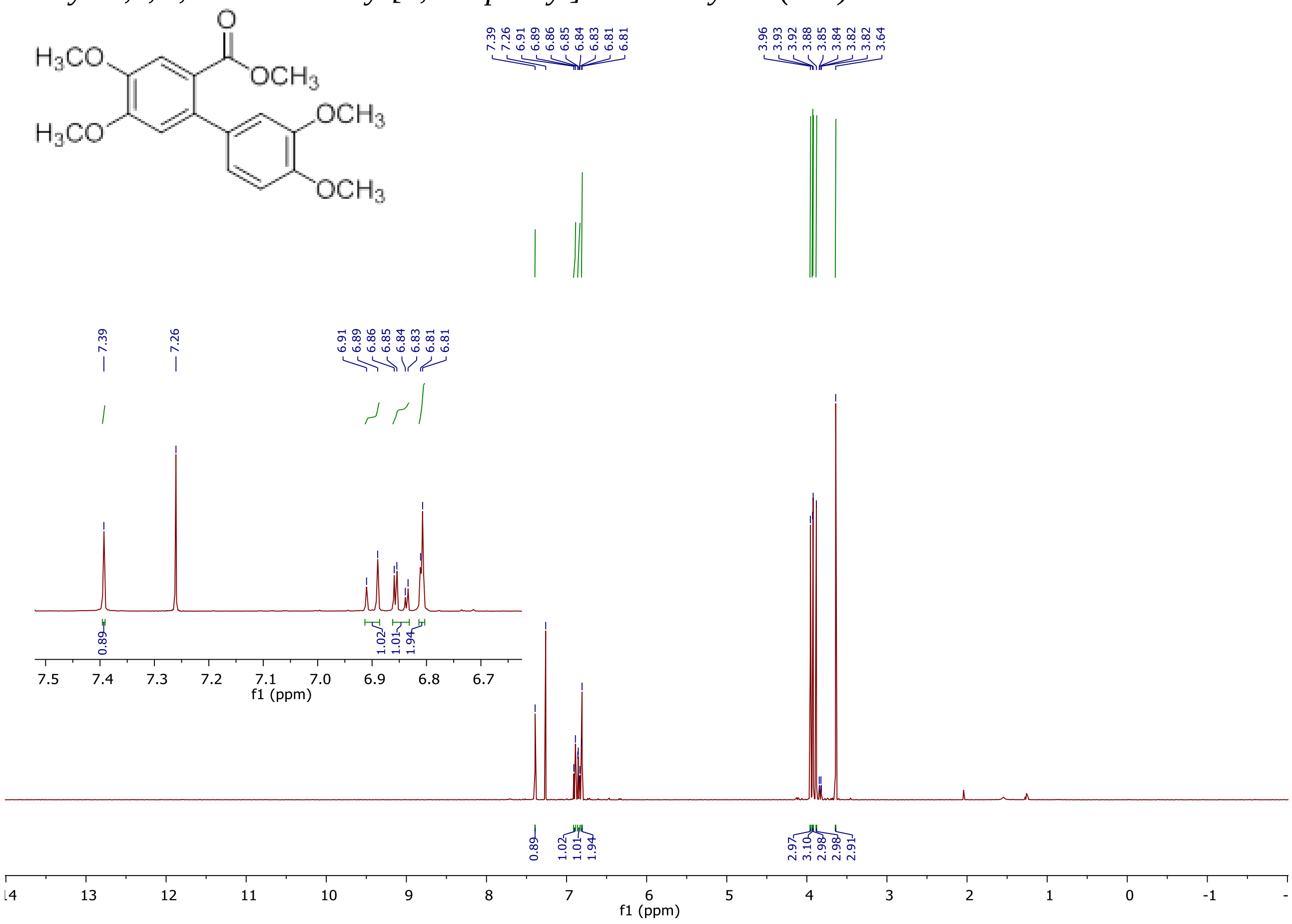

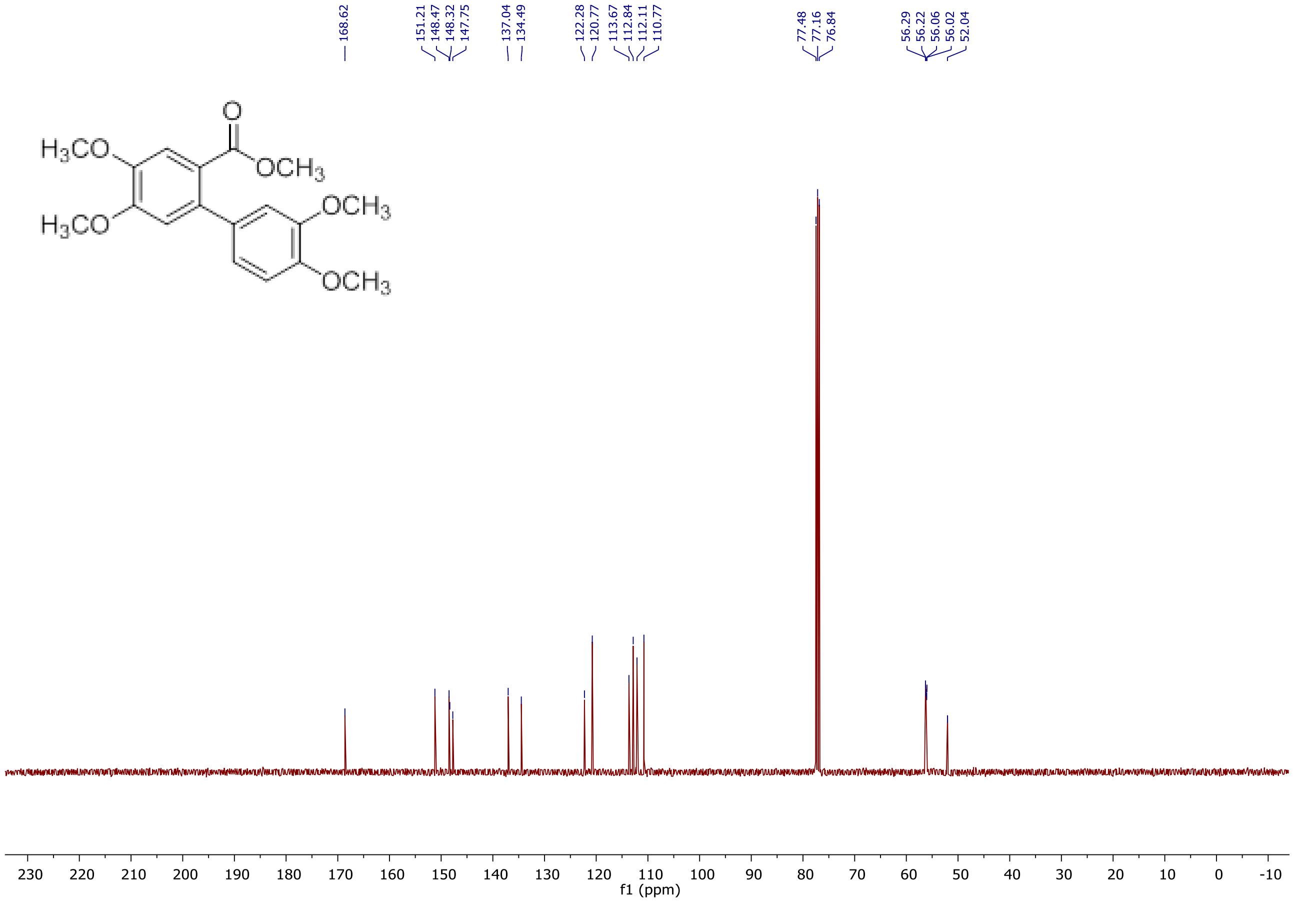

2,3,6,7-Tetramethoxy-9H-fluoren-9-one (**195**)

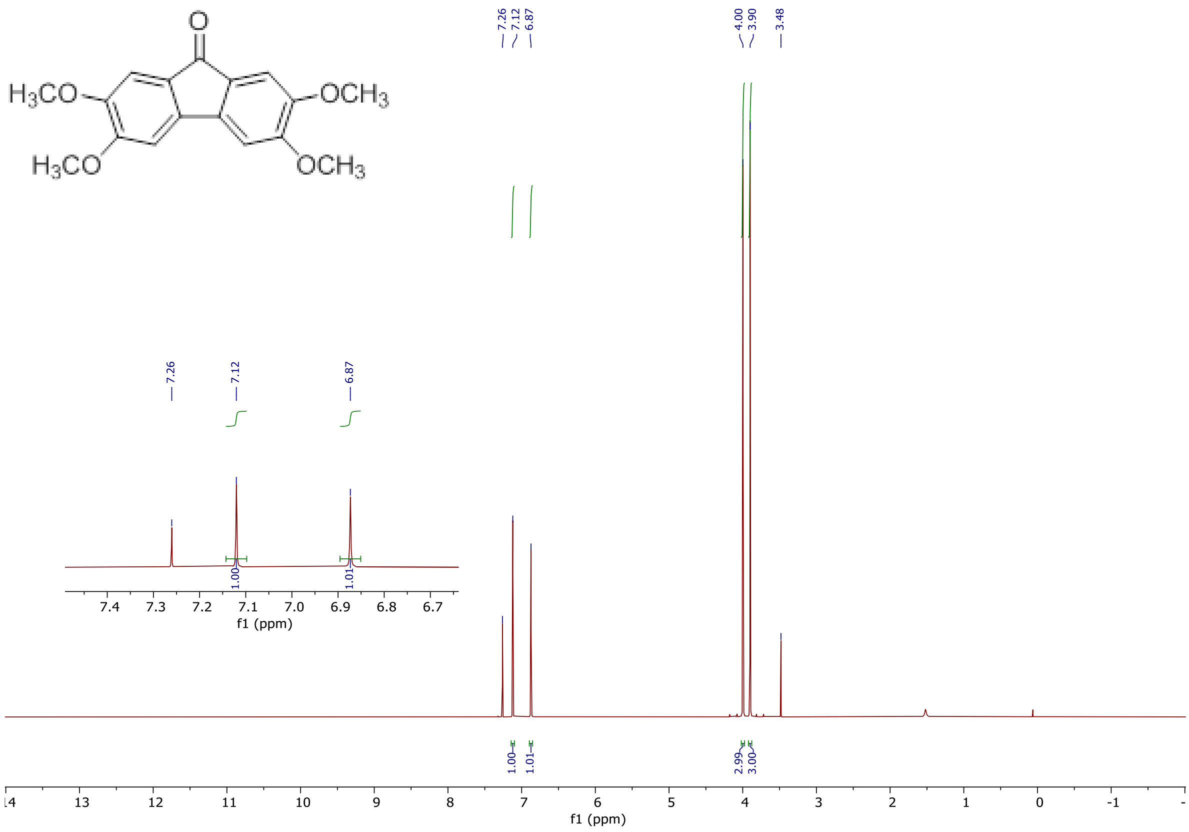

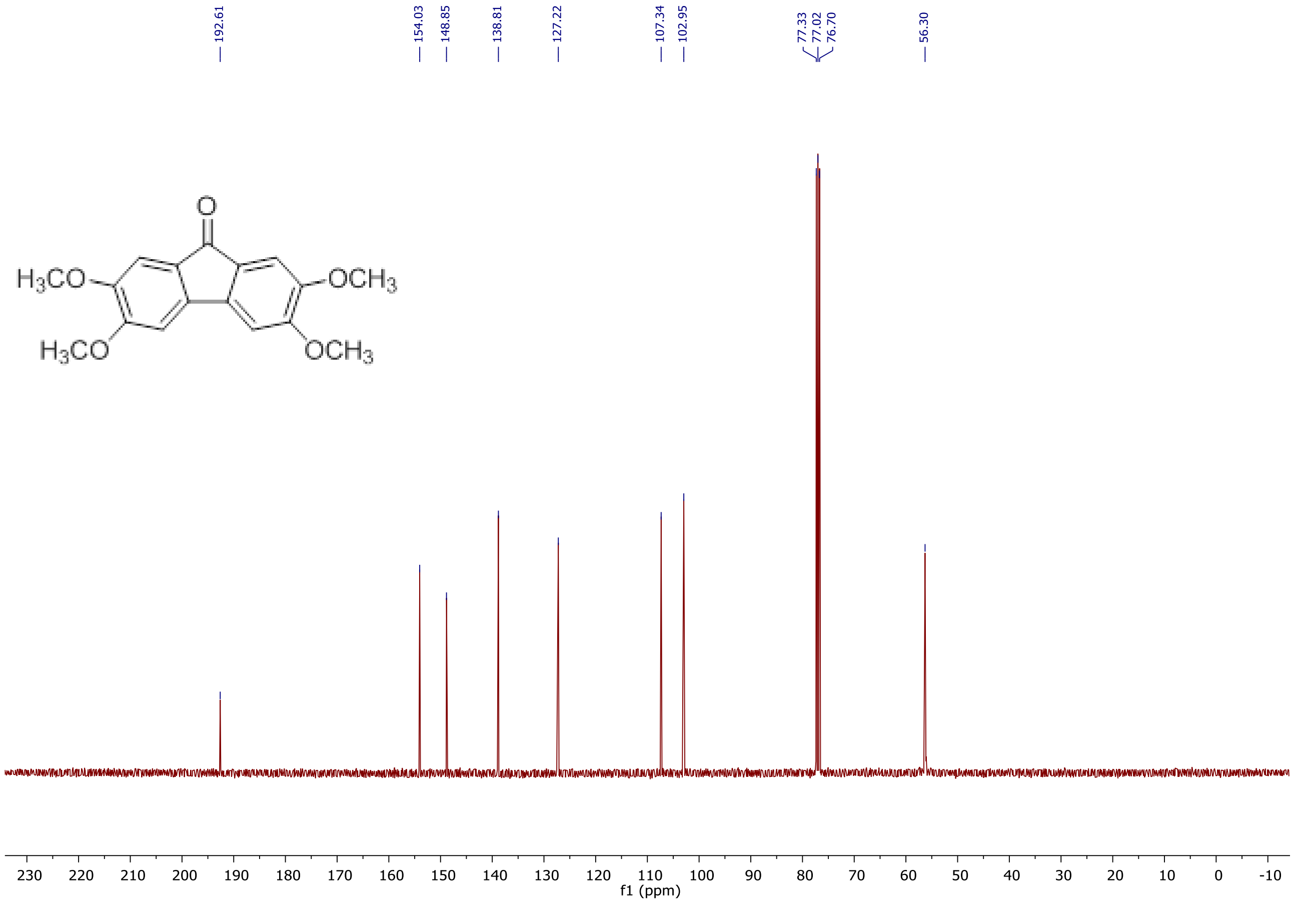

2,3,6,7-tetrahydroxy-9H-fluoren-9-one (**195**)

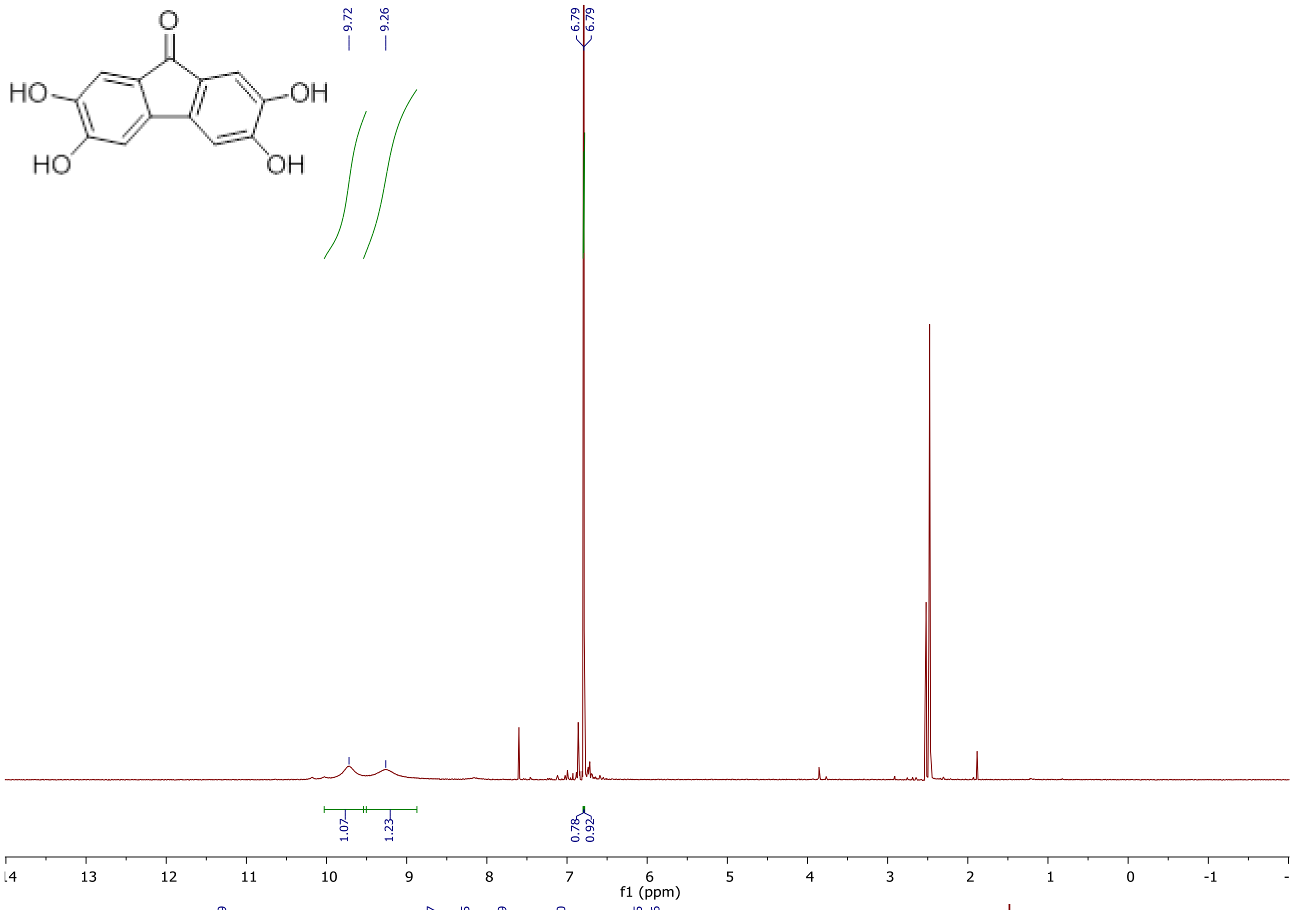

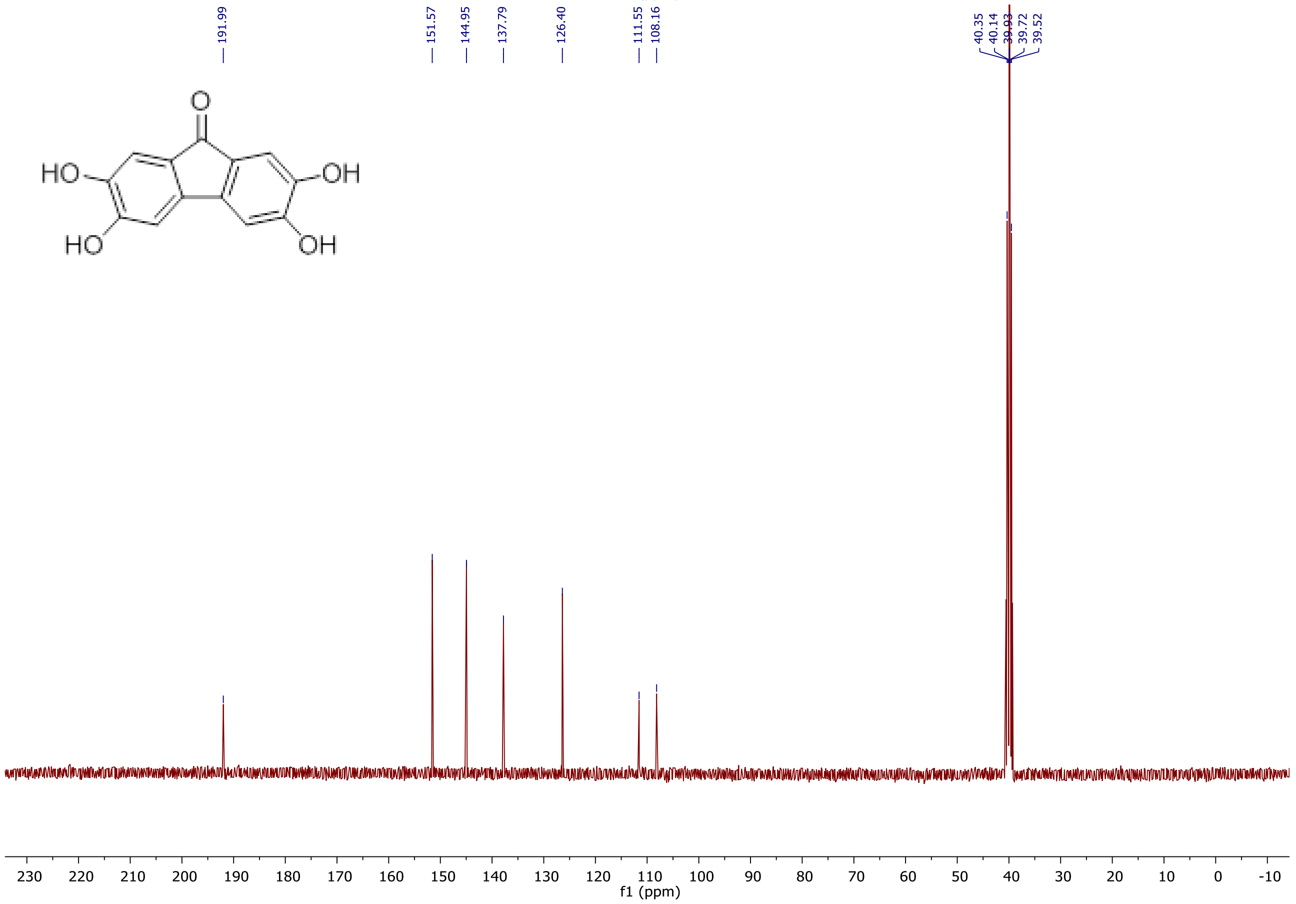

Methyl 4'-hydroxy-4,5-dimethoxy-[1,1'-biphenyl]-2-carboxylate (**189**)

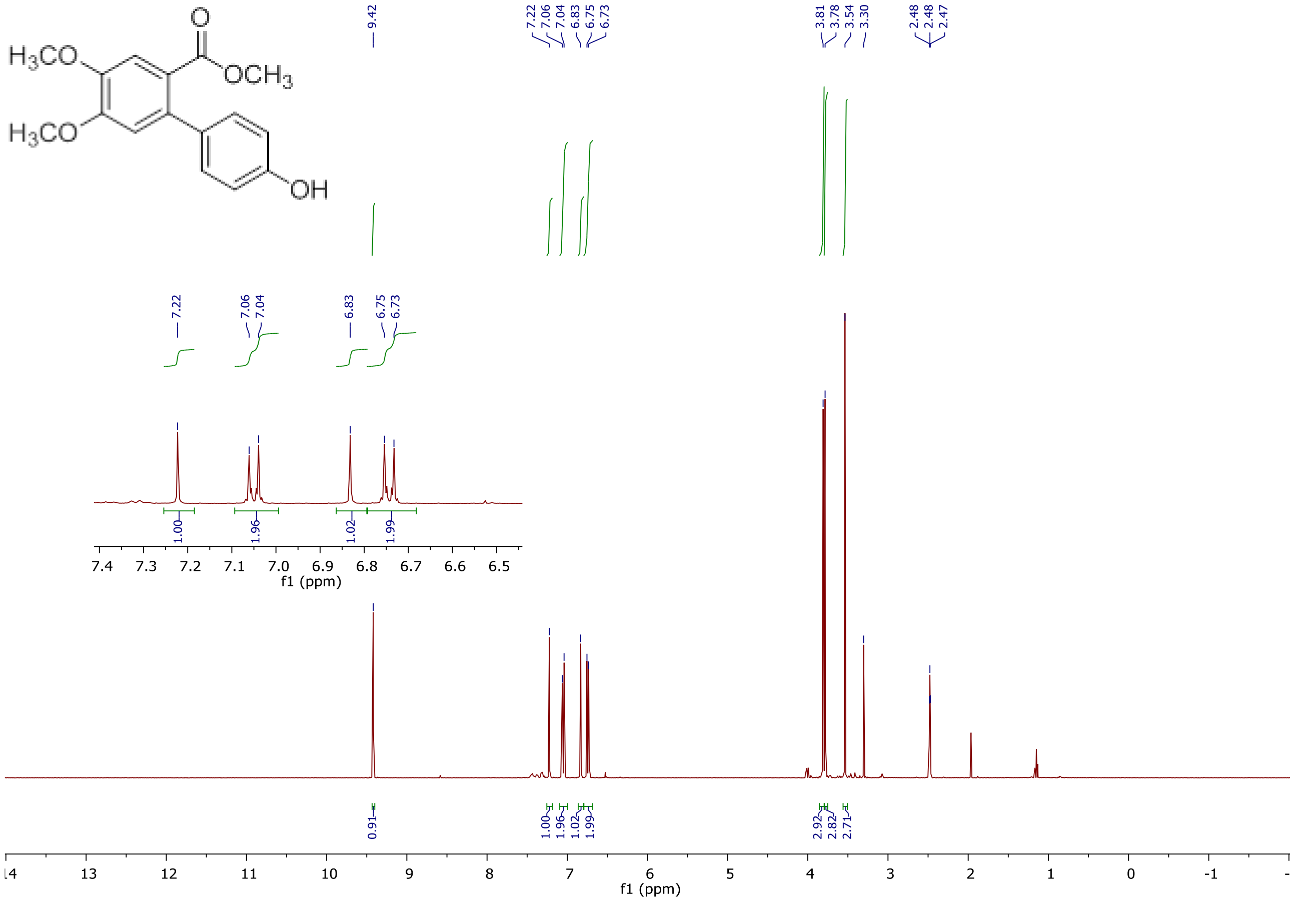

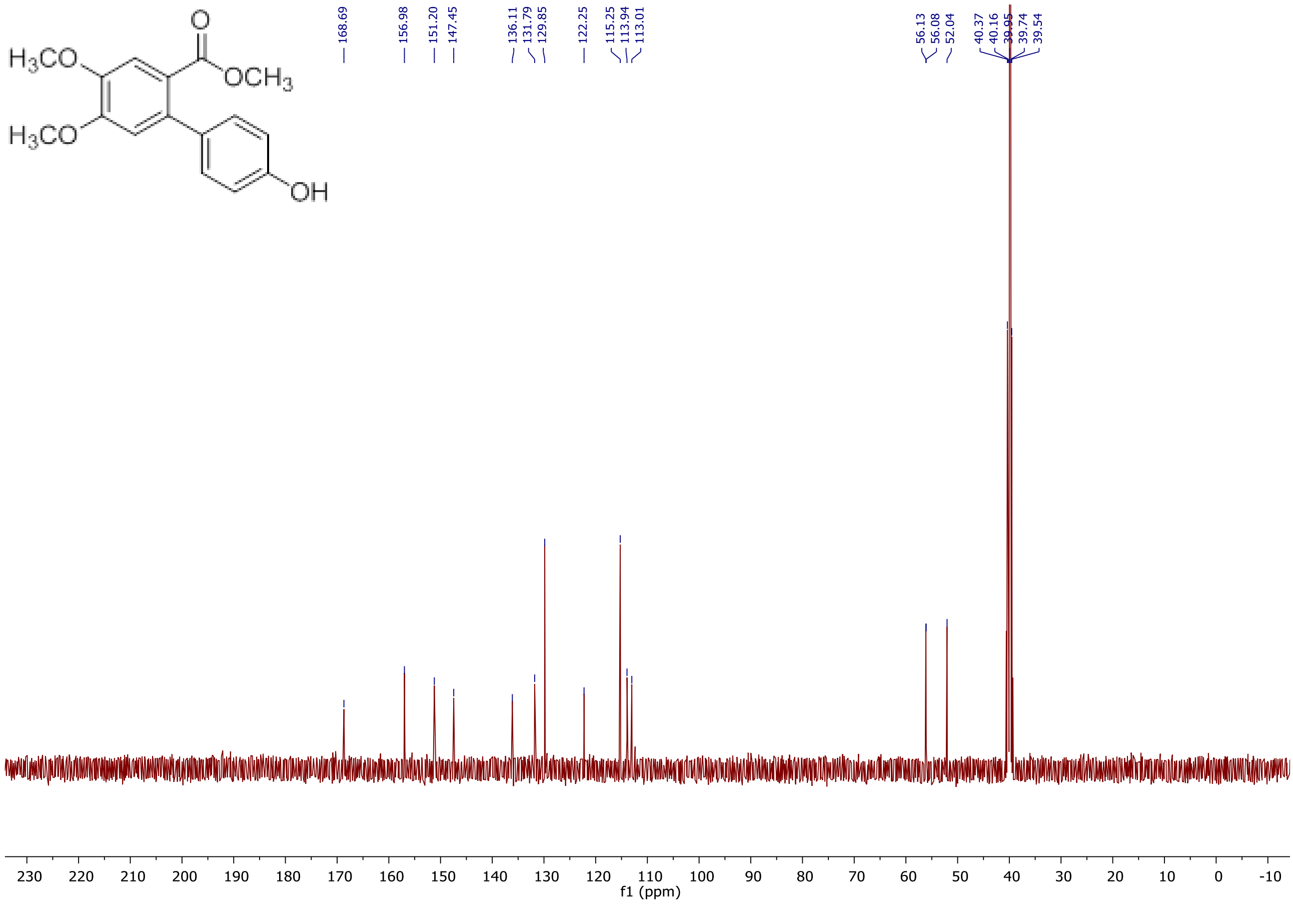

7-Hydroxy-2,3-dimethoxy-9H-fluoren-9-one (**197**)

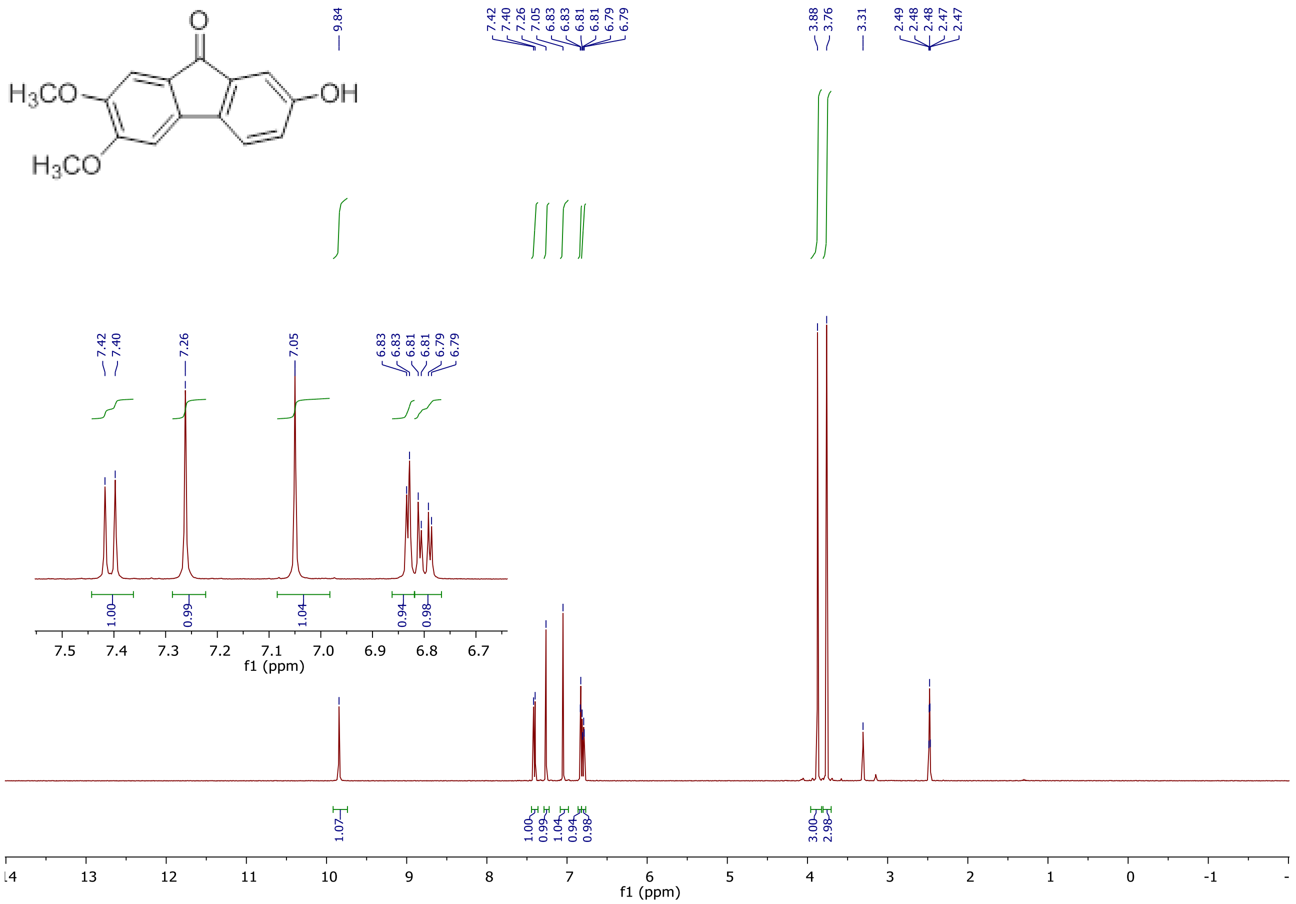

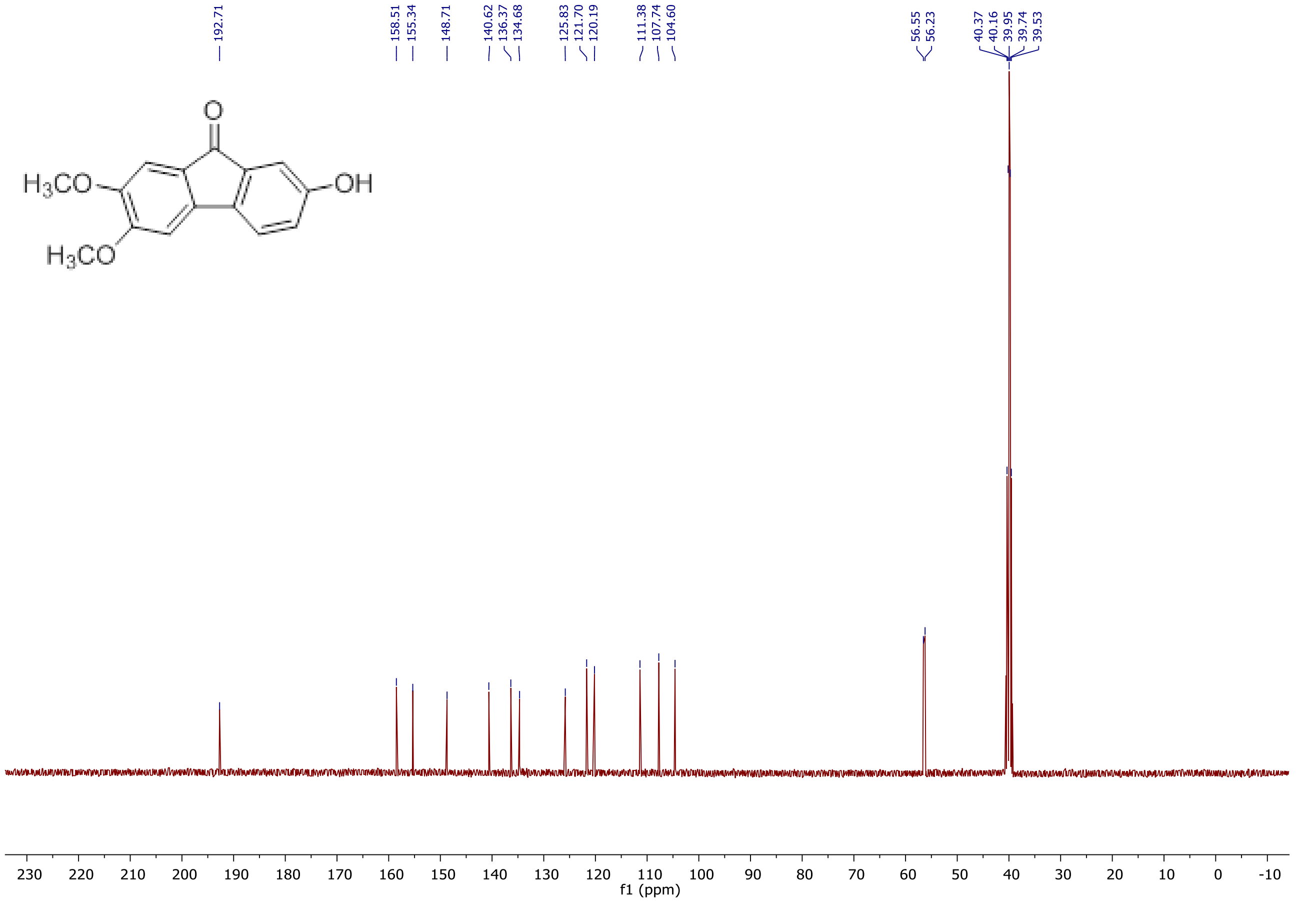

Methyl 6-(4-hydroxyphenyl)benzo[d][1,3]dioxole-5-carboxylate (**190**)

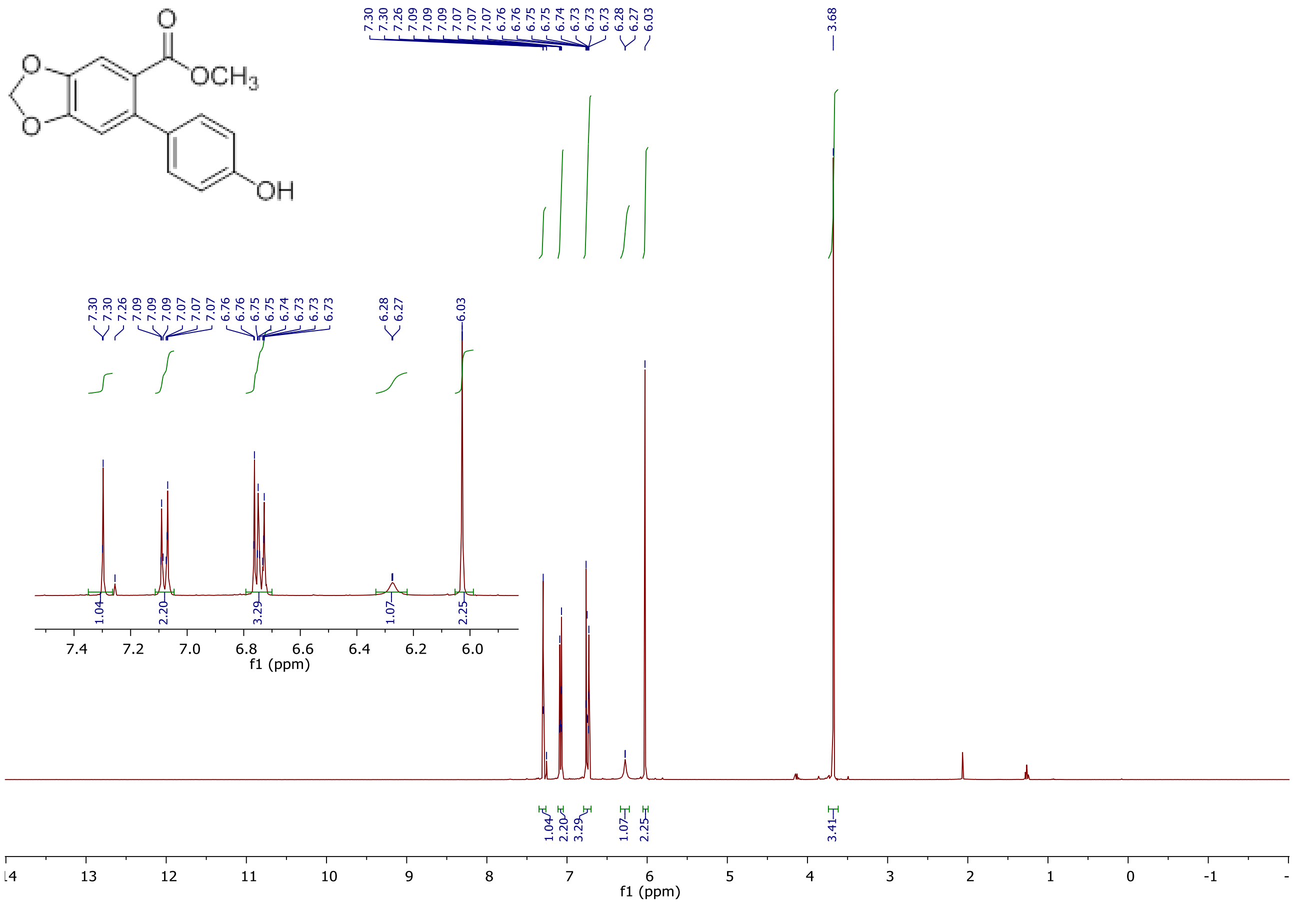

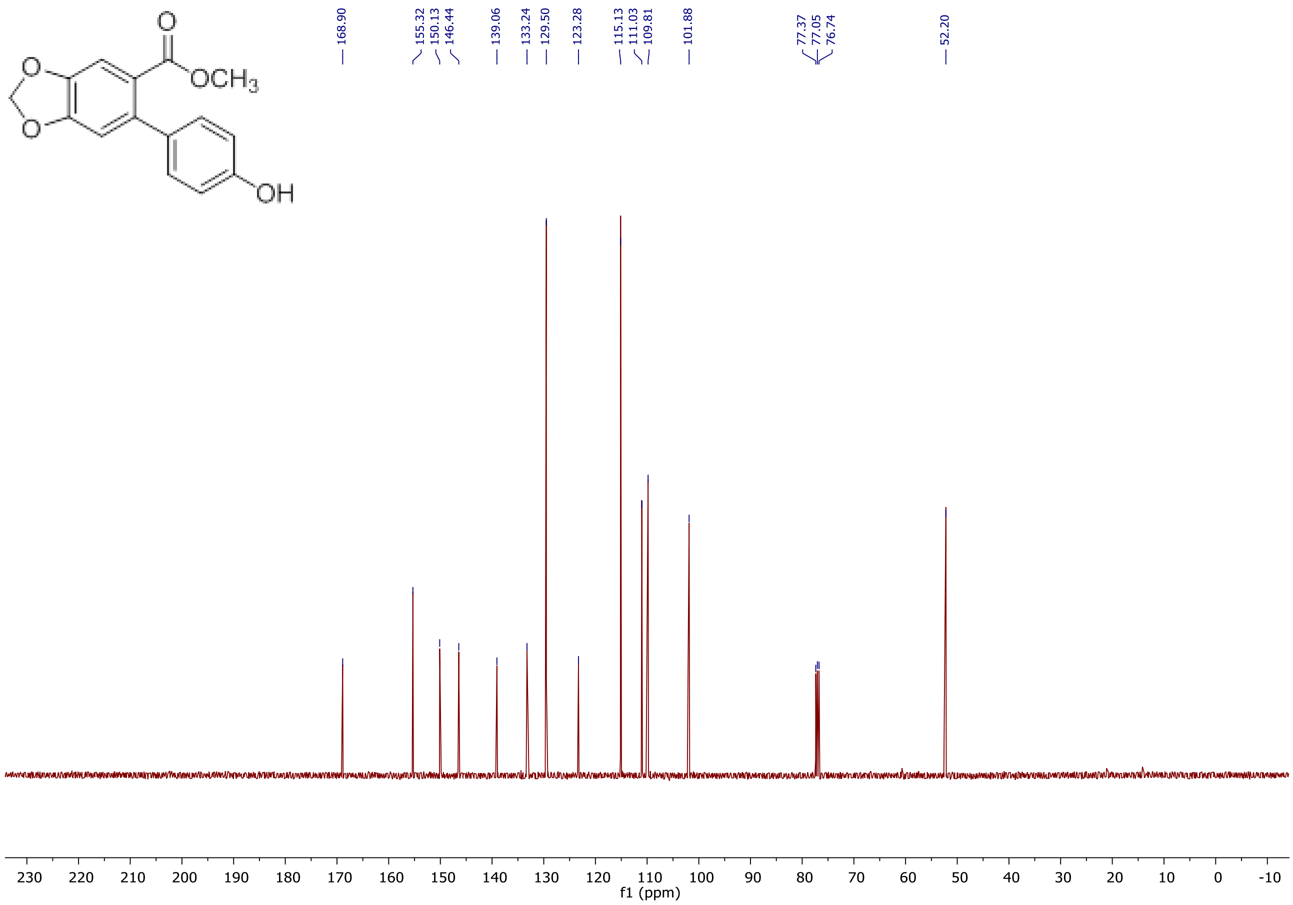

7-Hydroxy-9H-fluoreno[2,3-d][1,3]dioxol-9-one (**198**)

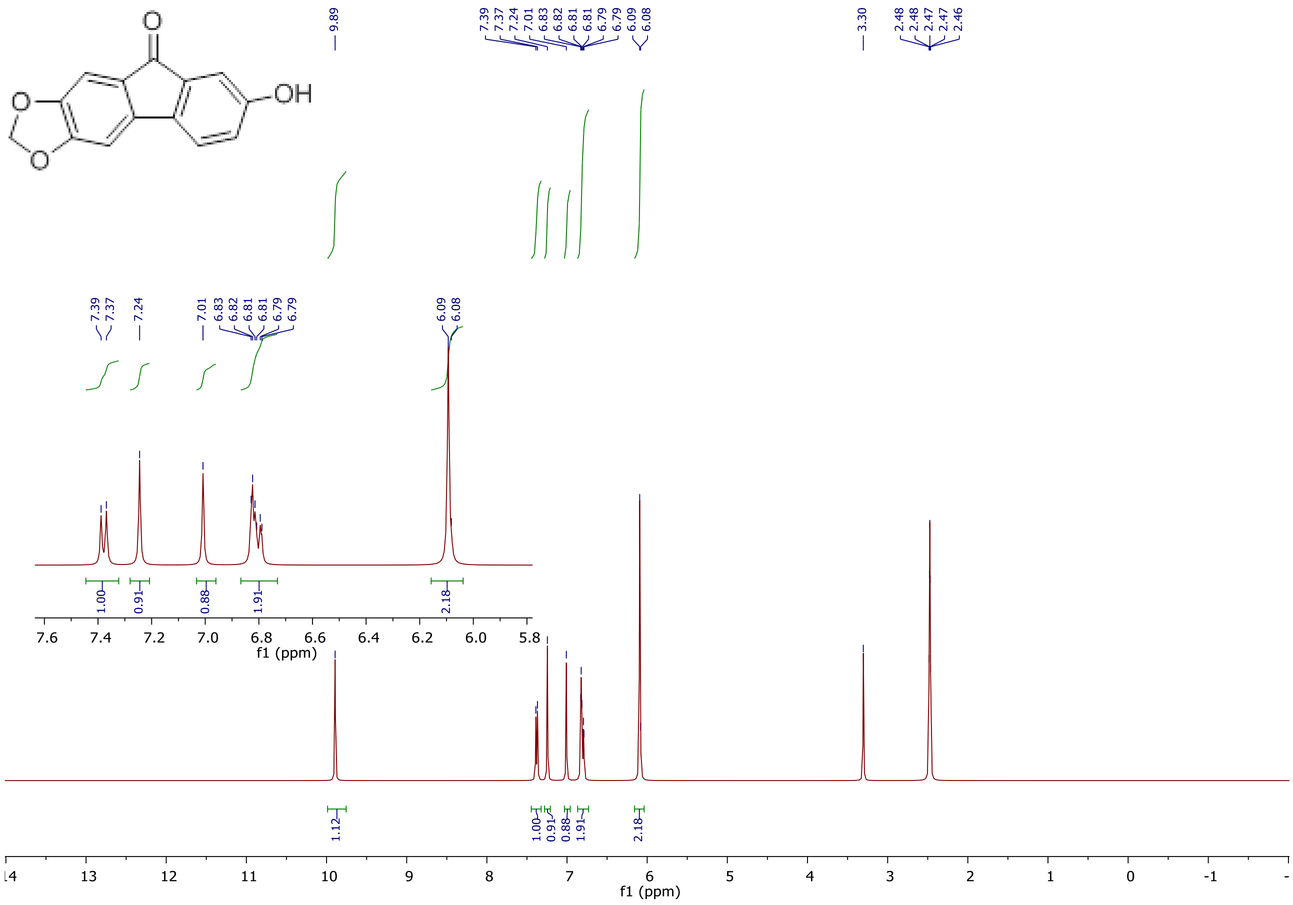

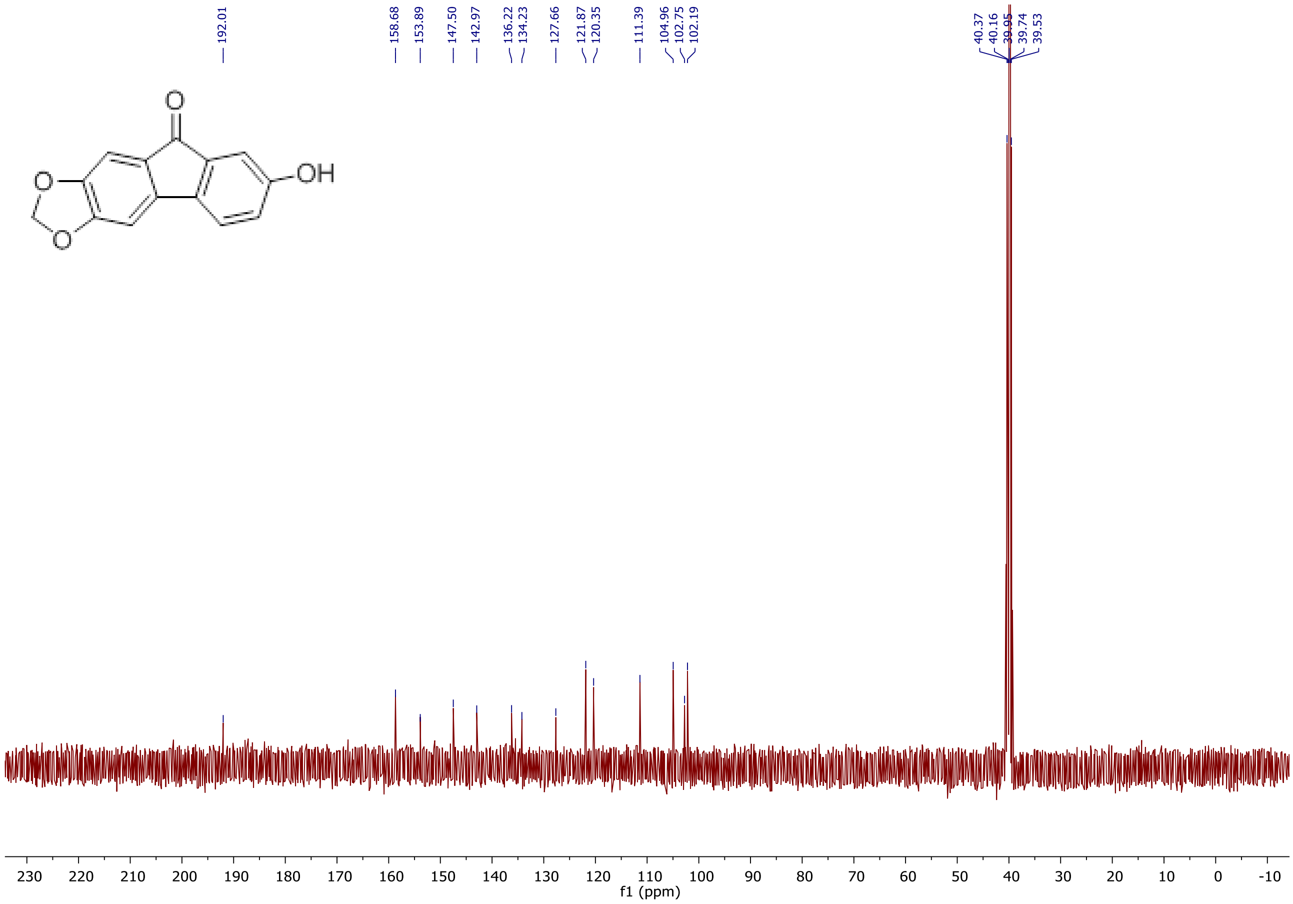

2,3,7-tris(benzyloxy)-9H-fluoren-9-one (**199**)

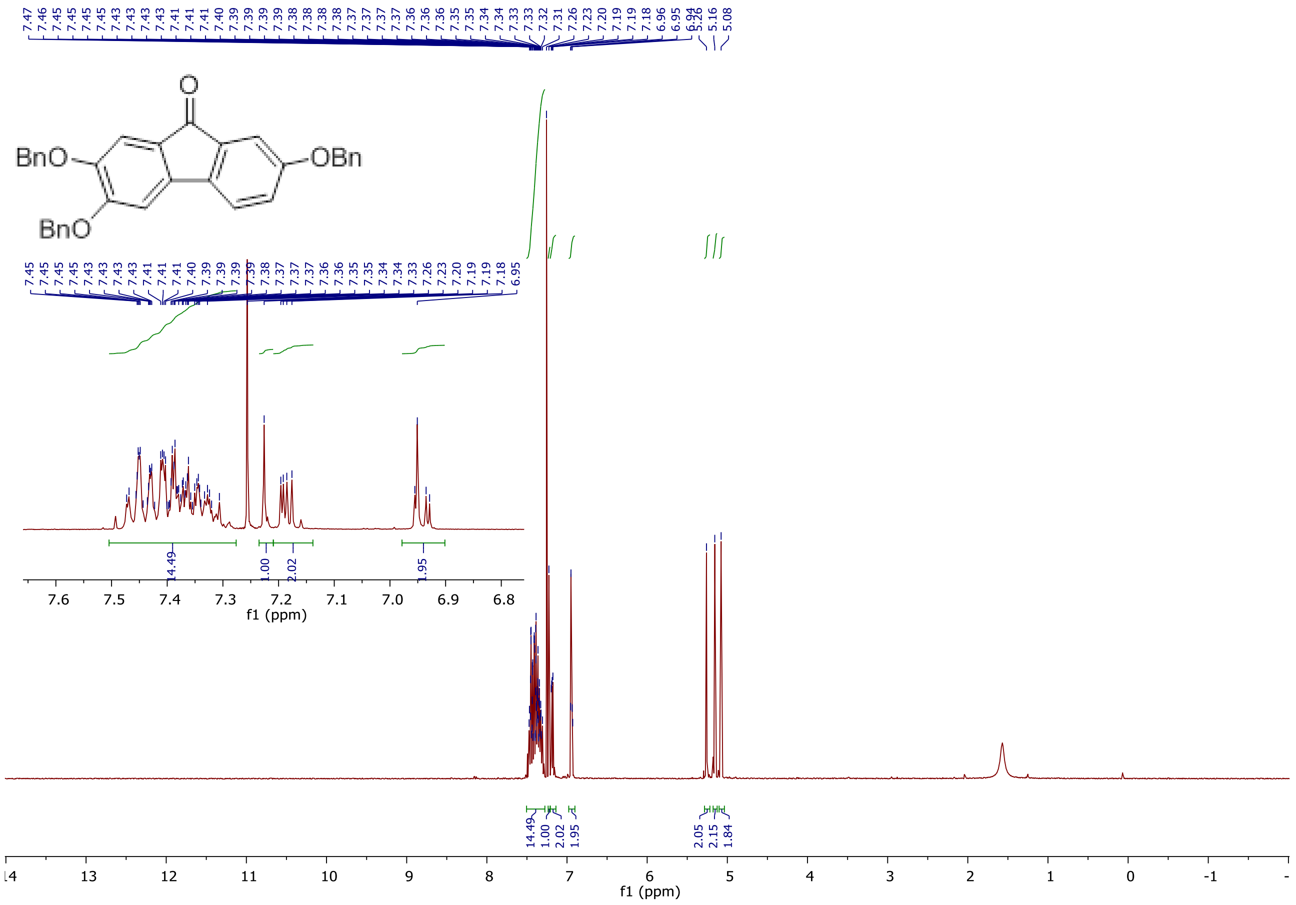

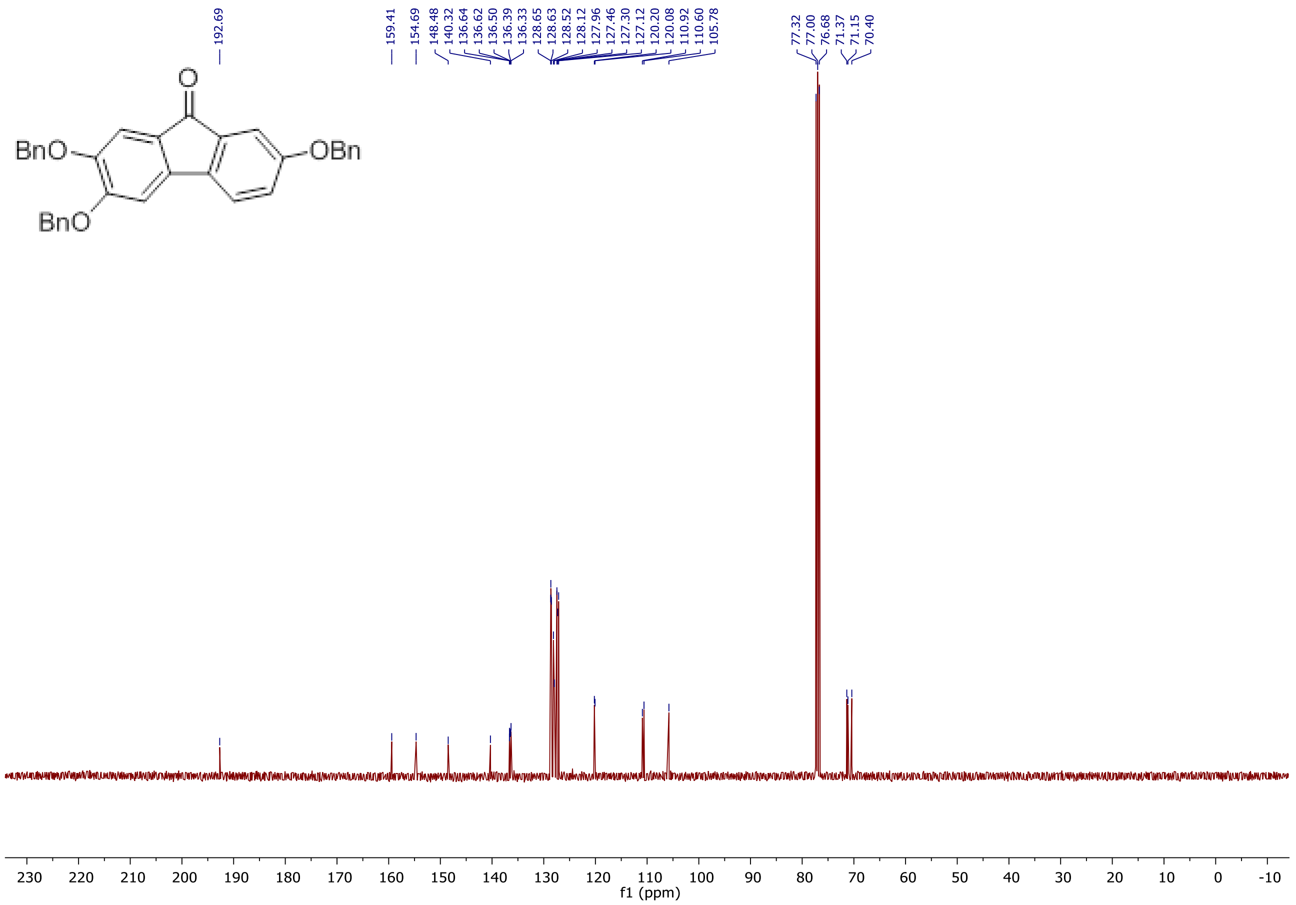

*2,3,7-Tris(benzyloxy)-9H-fluoren-9-ol (**200**)*

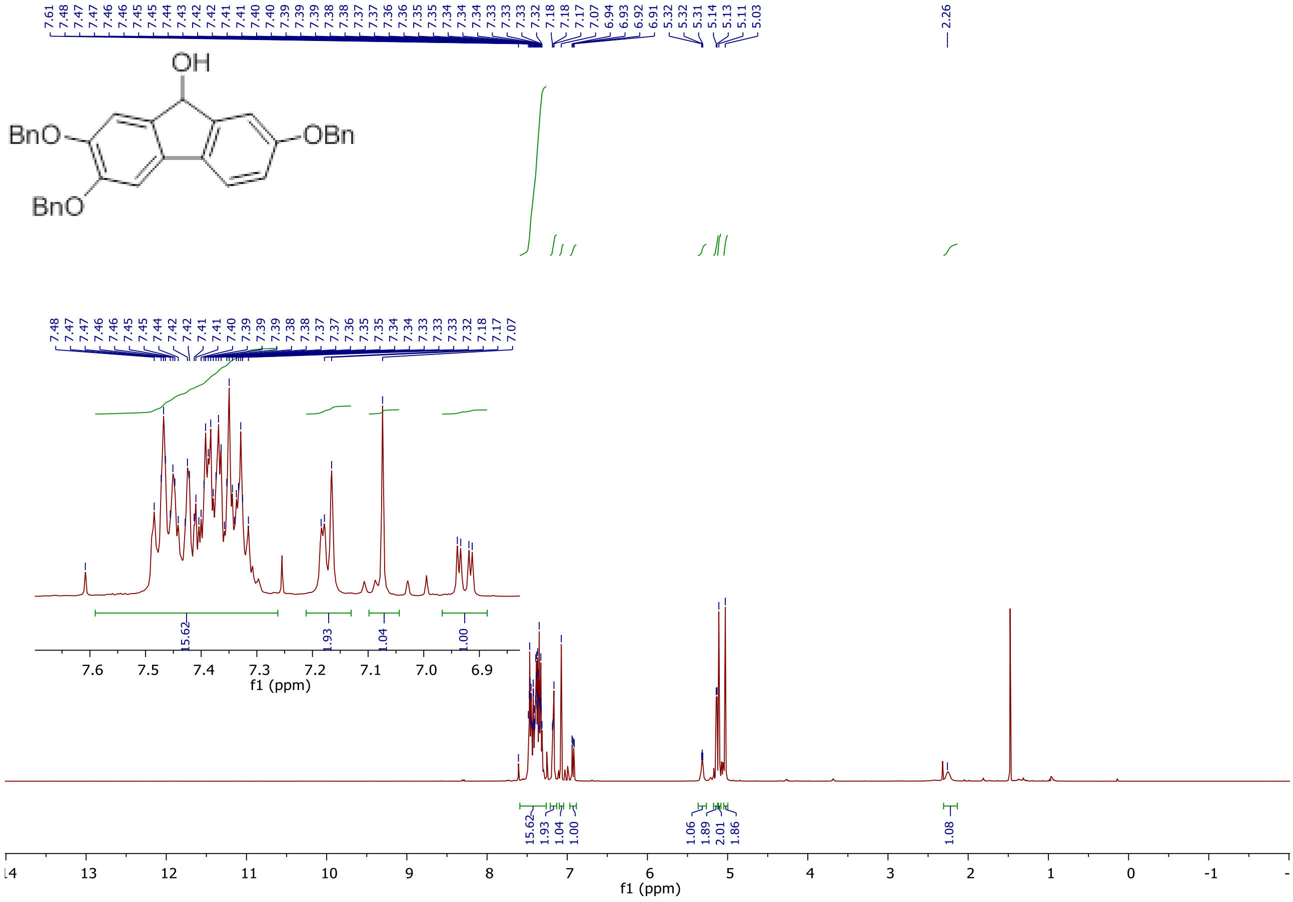

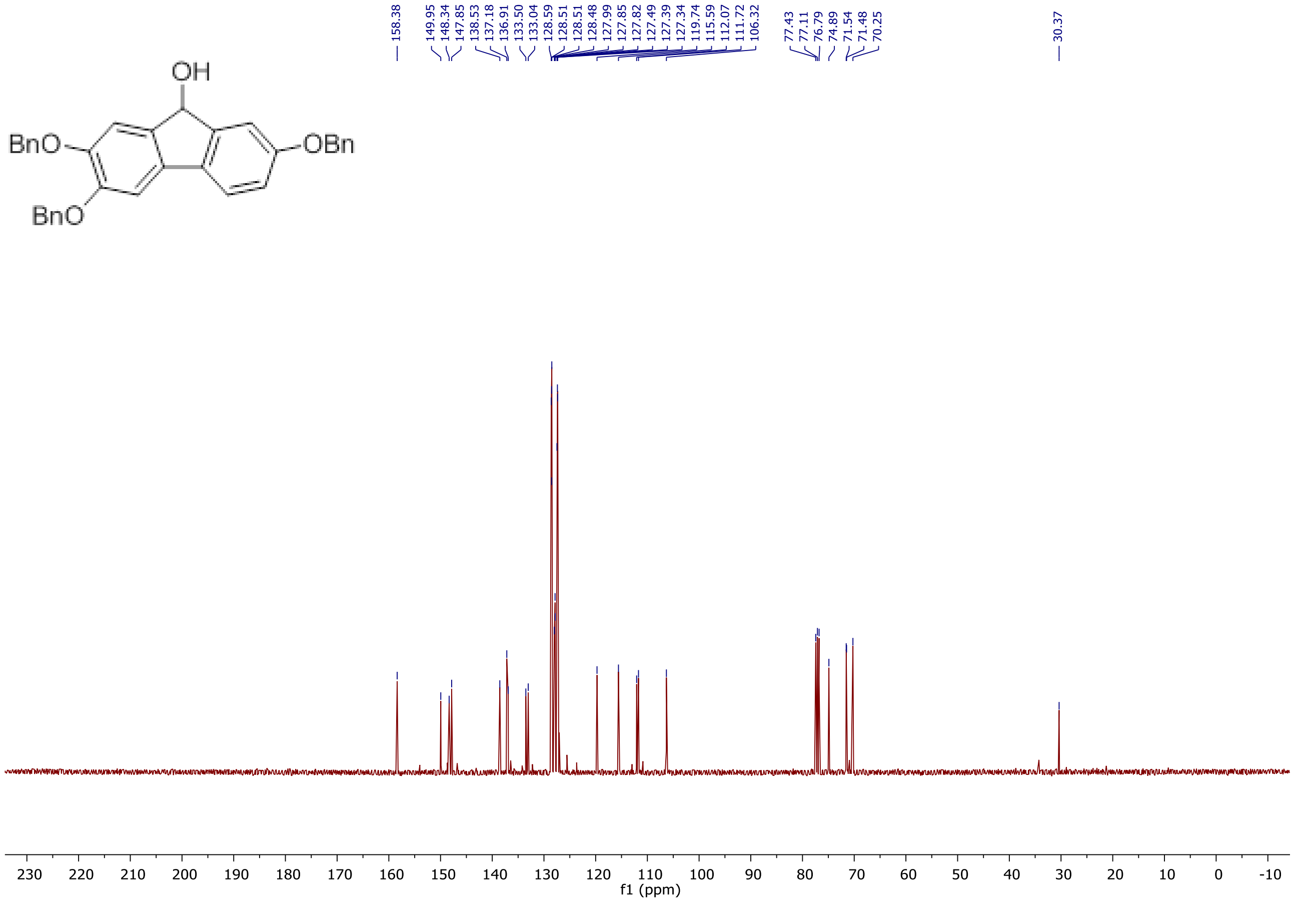

2,3,7-tris(benzyloxy)-9-methoxy-9H-fluorene (**201**)

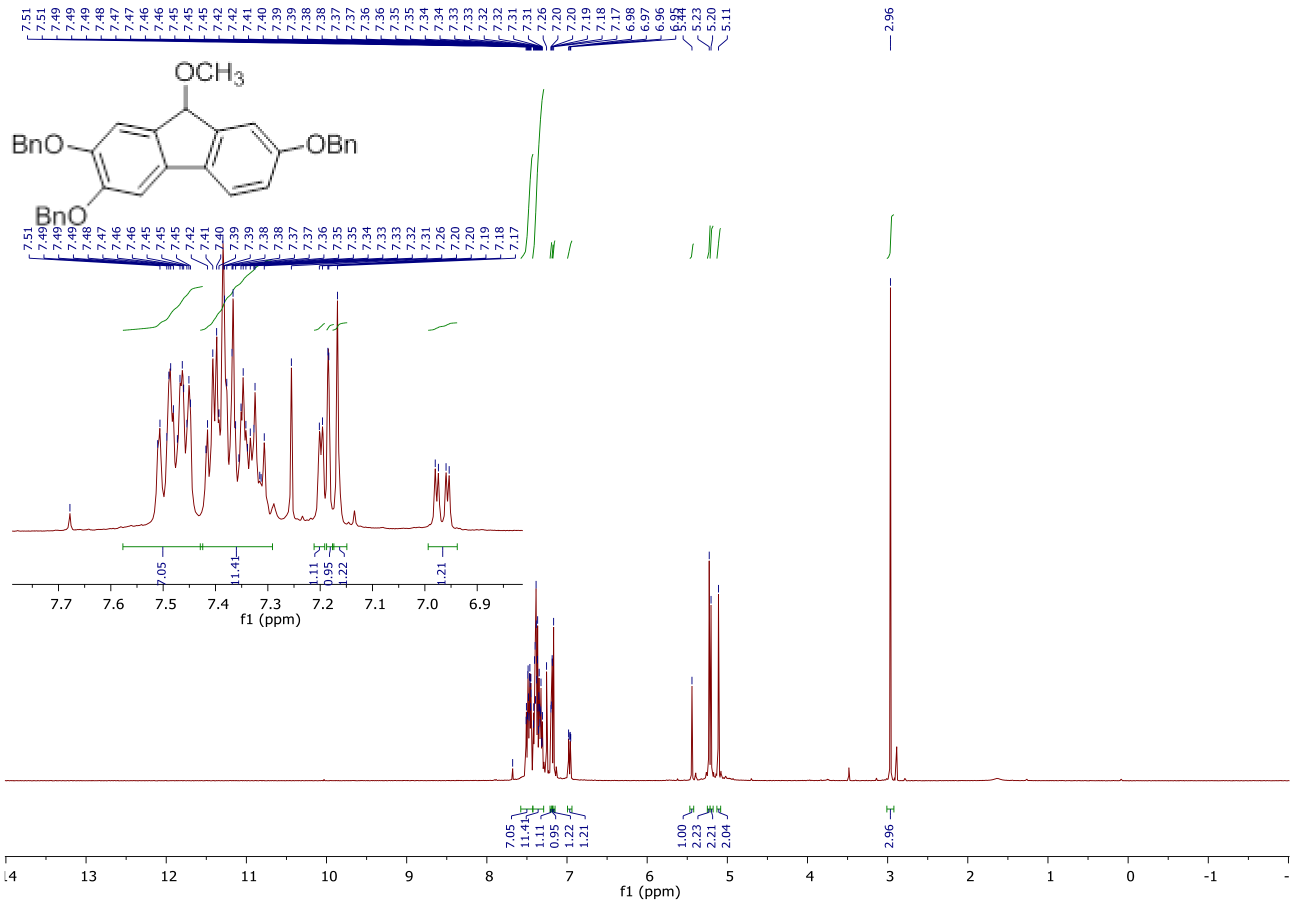

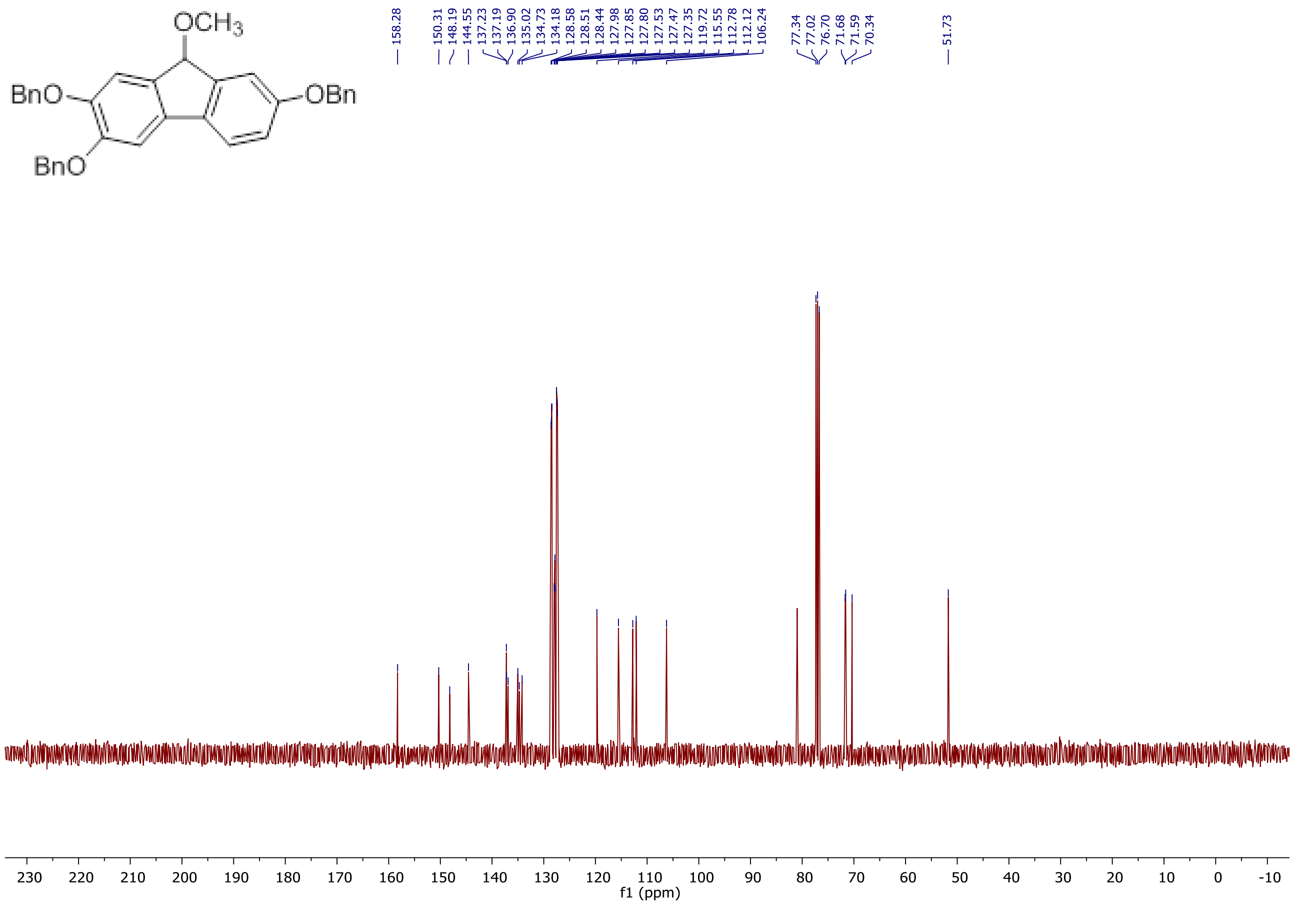

9H-Fluorene-2,3,7-triol (**202**)

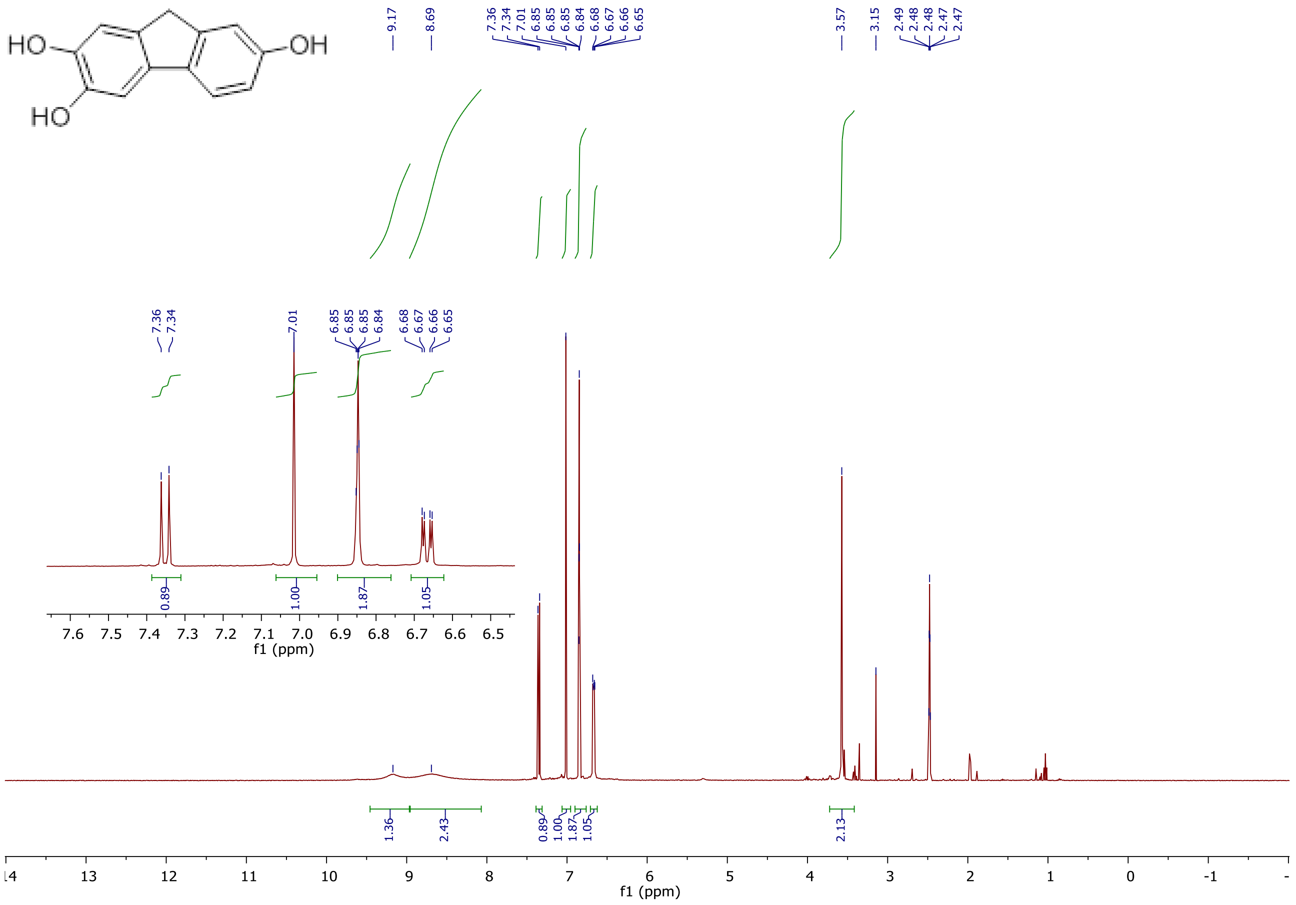

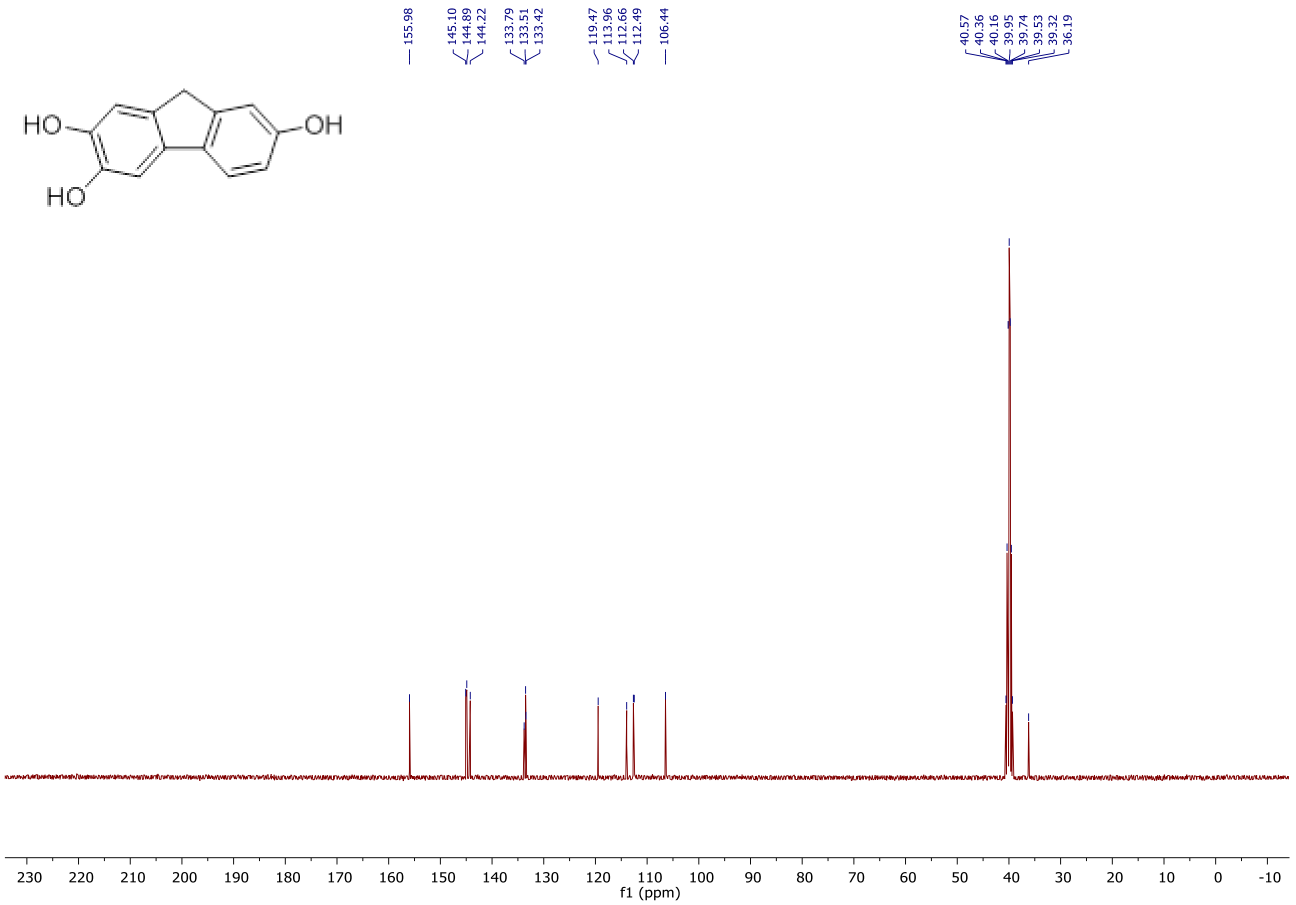

(3,4-Dimethoxyphenyl)(3-methoxyphenyl)methanone (**206**)

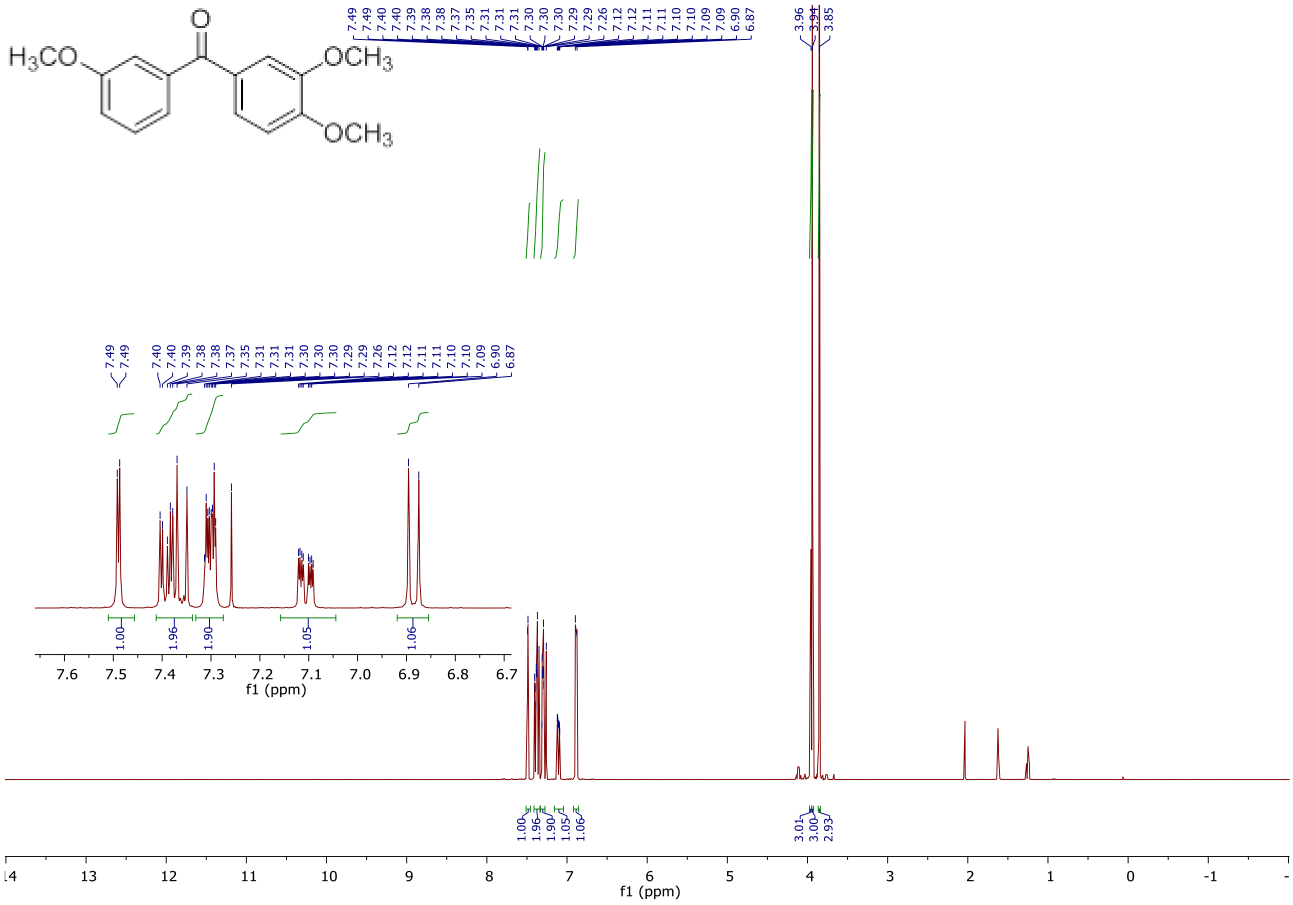

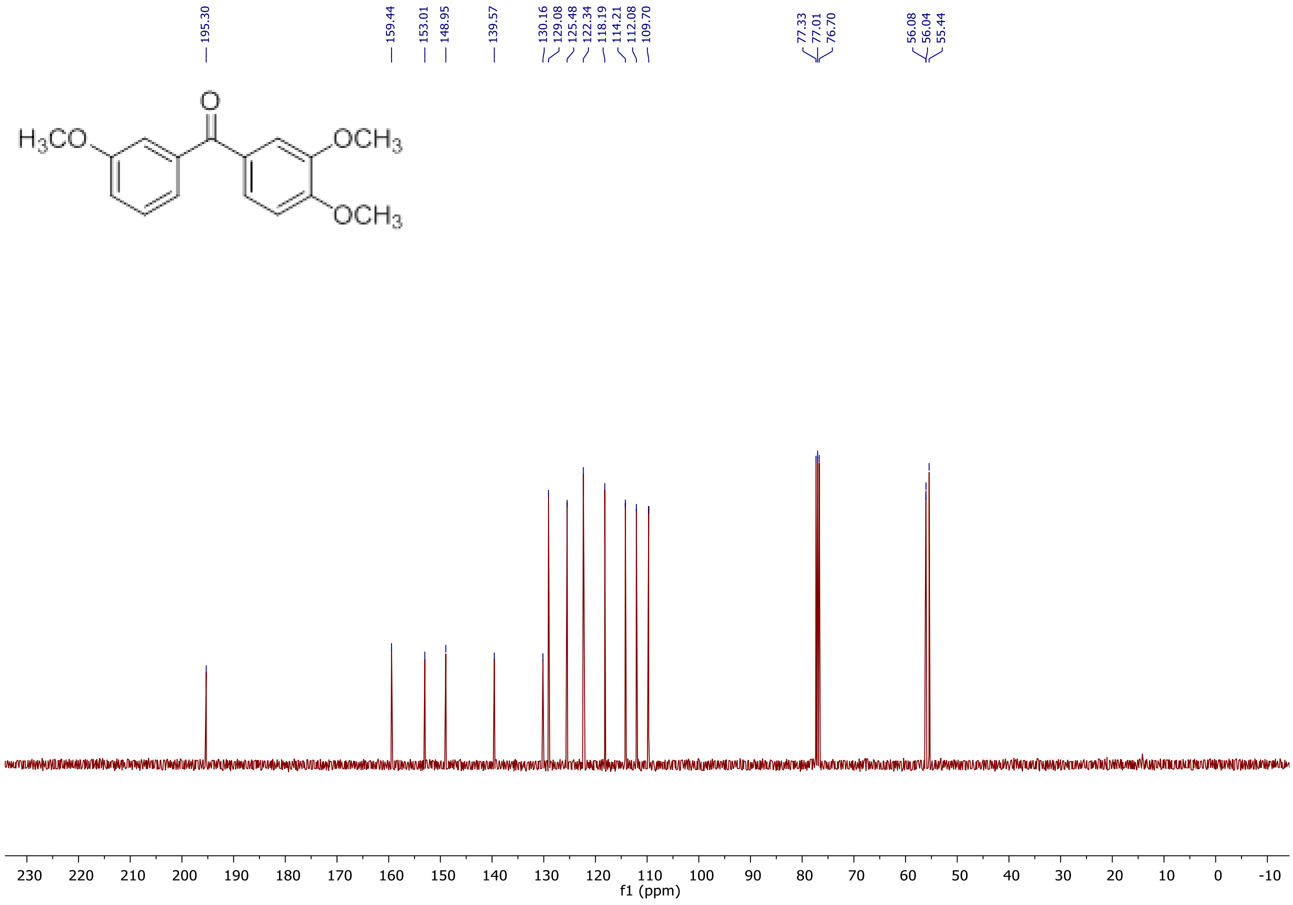

(3,4-Dihydroxyphenyl)(3-hydroxyphenyl)methanone **(209)**

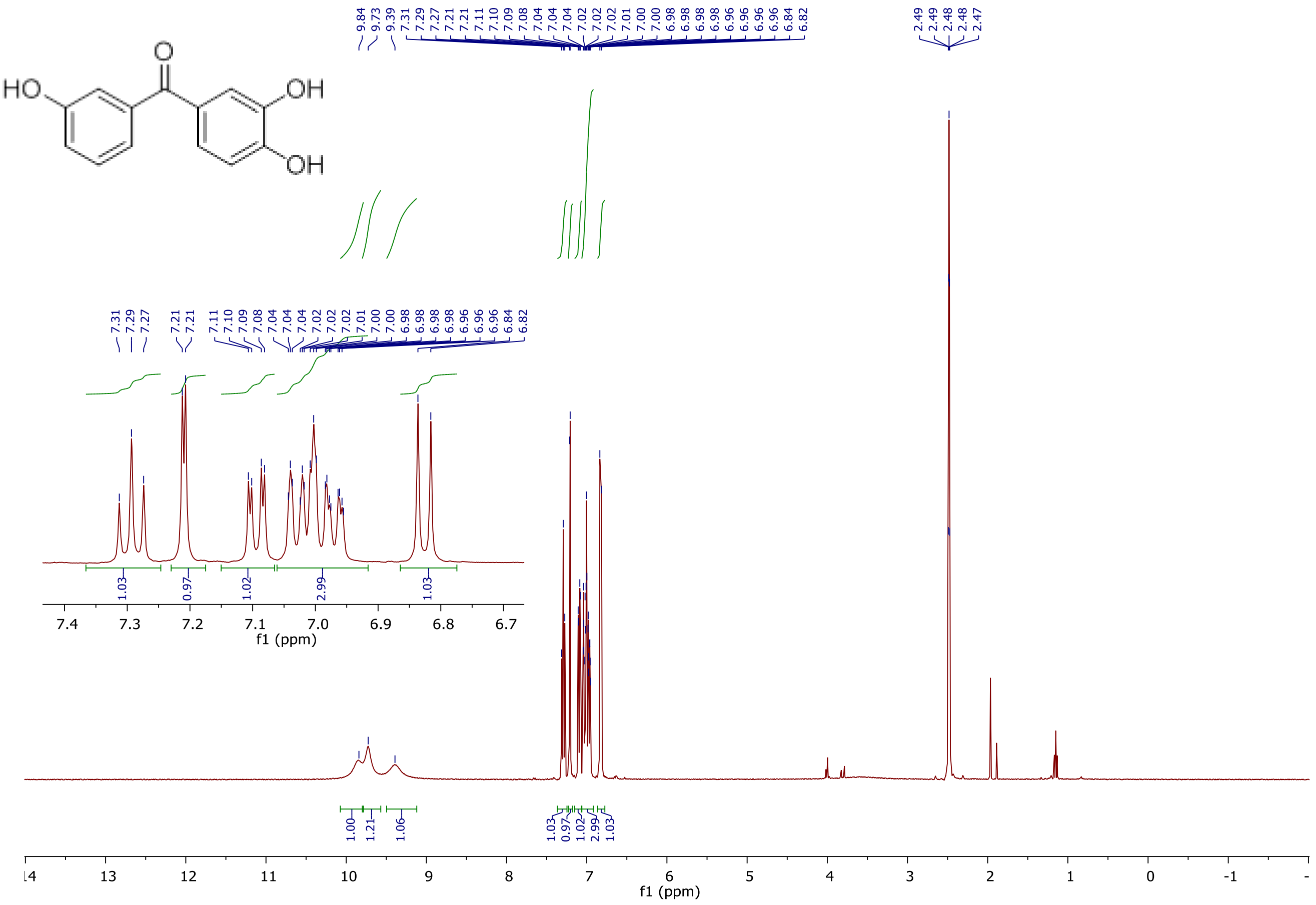

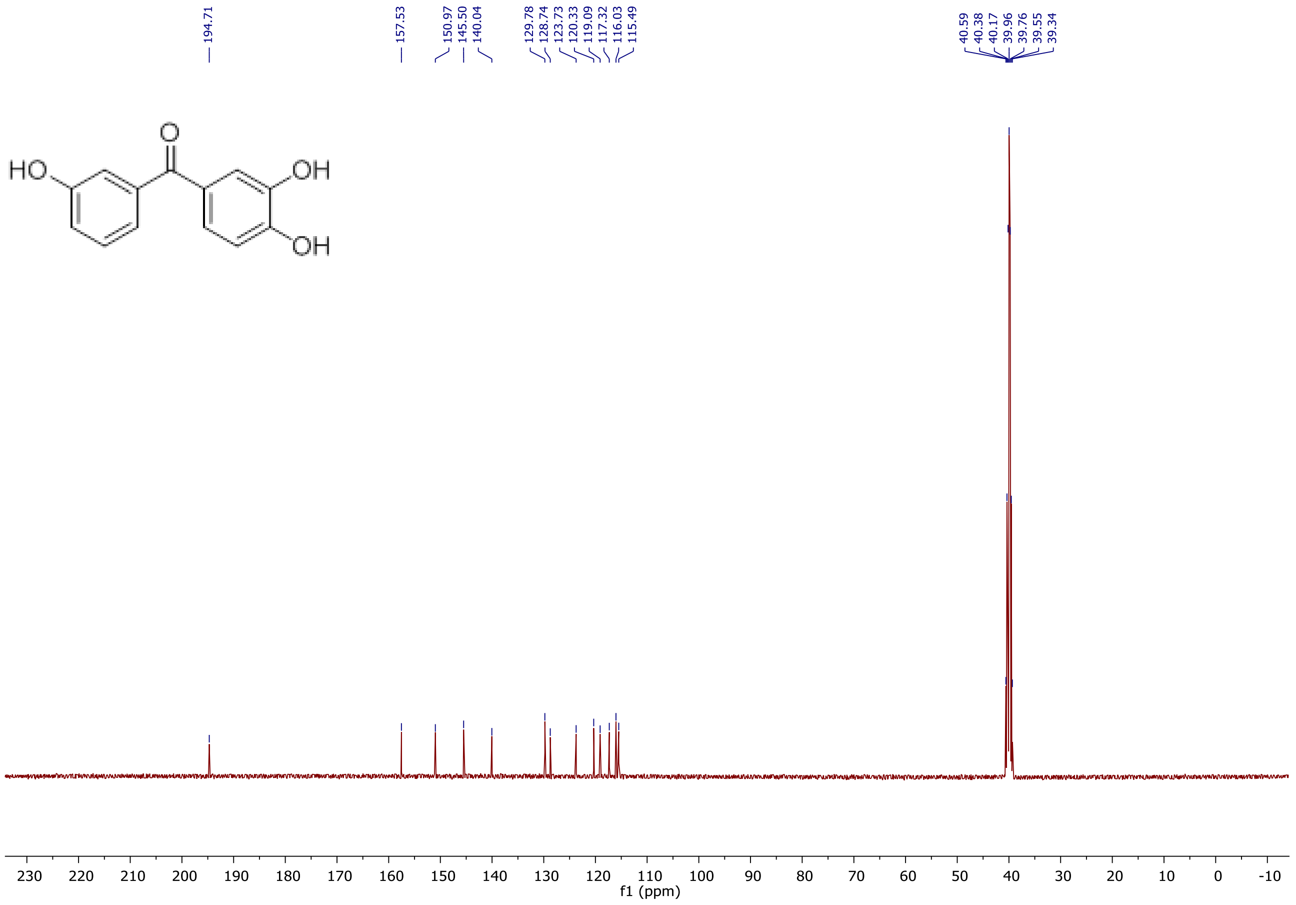

(3,4-Dimethoxyphenyl)(4-methoxyphenyl)methanone (**207**)

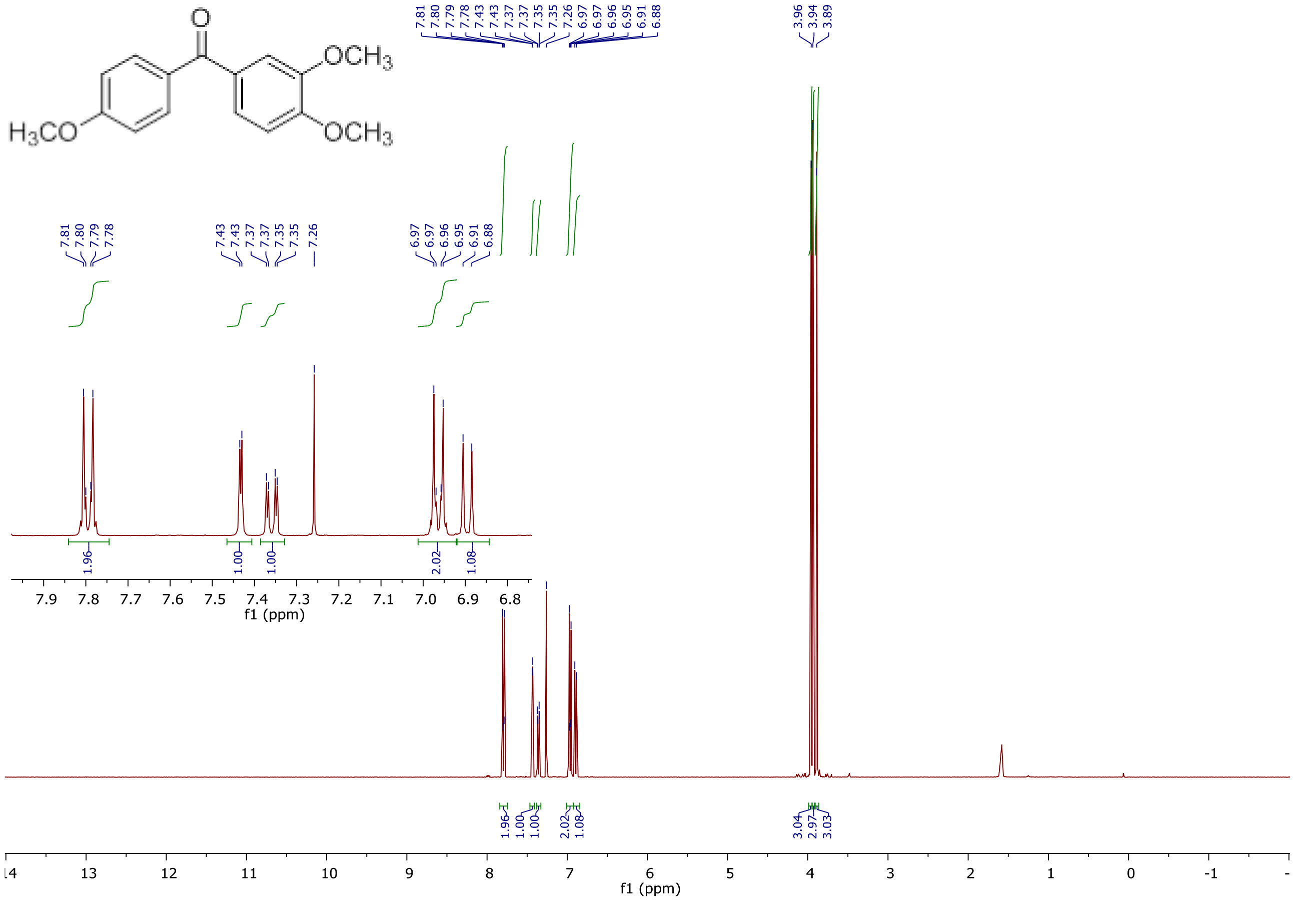

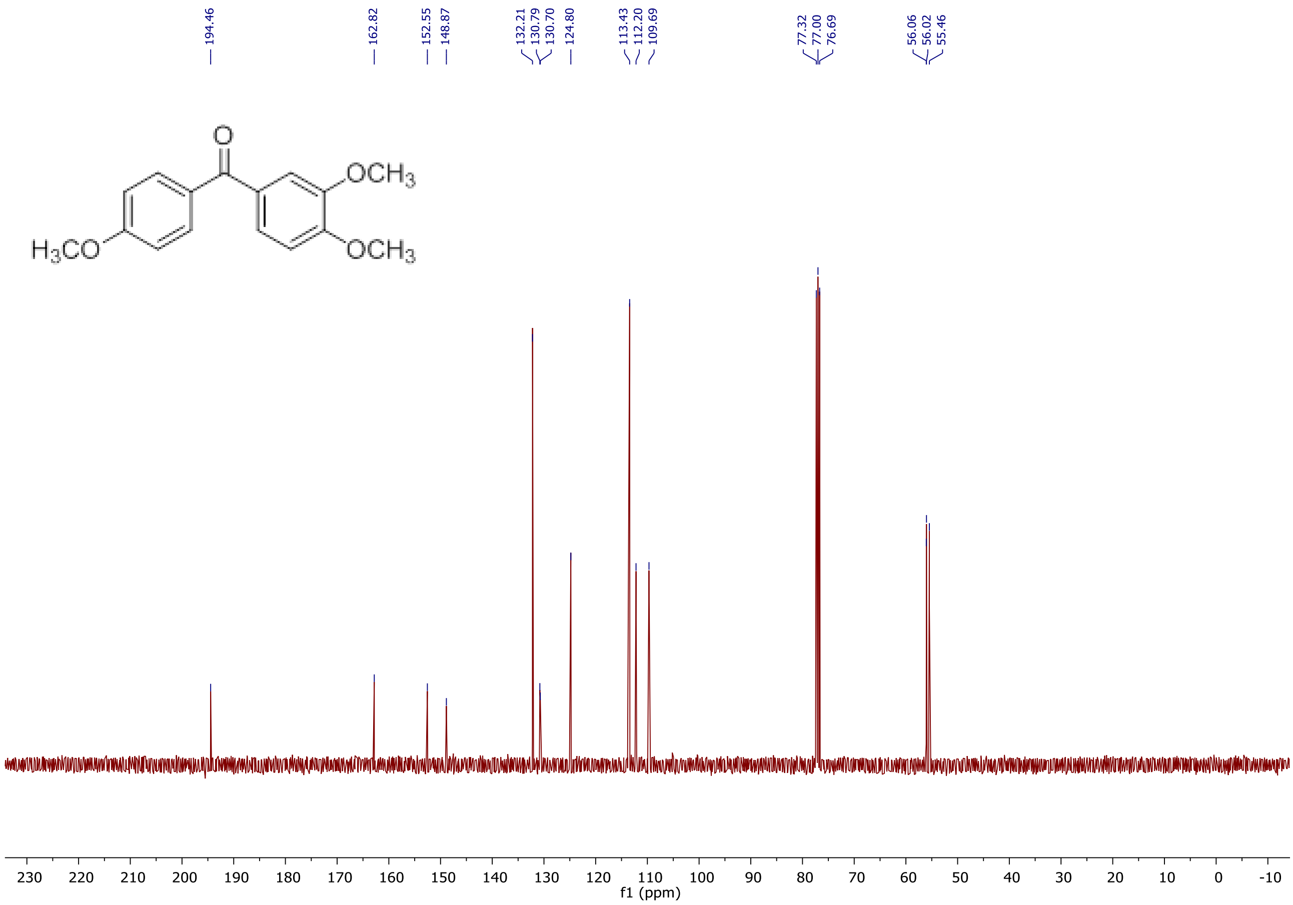

(3,4-Dihydroxyphenyl)(4-hydroxyphenyl)methanone (**210**)

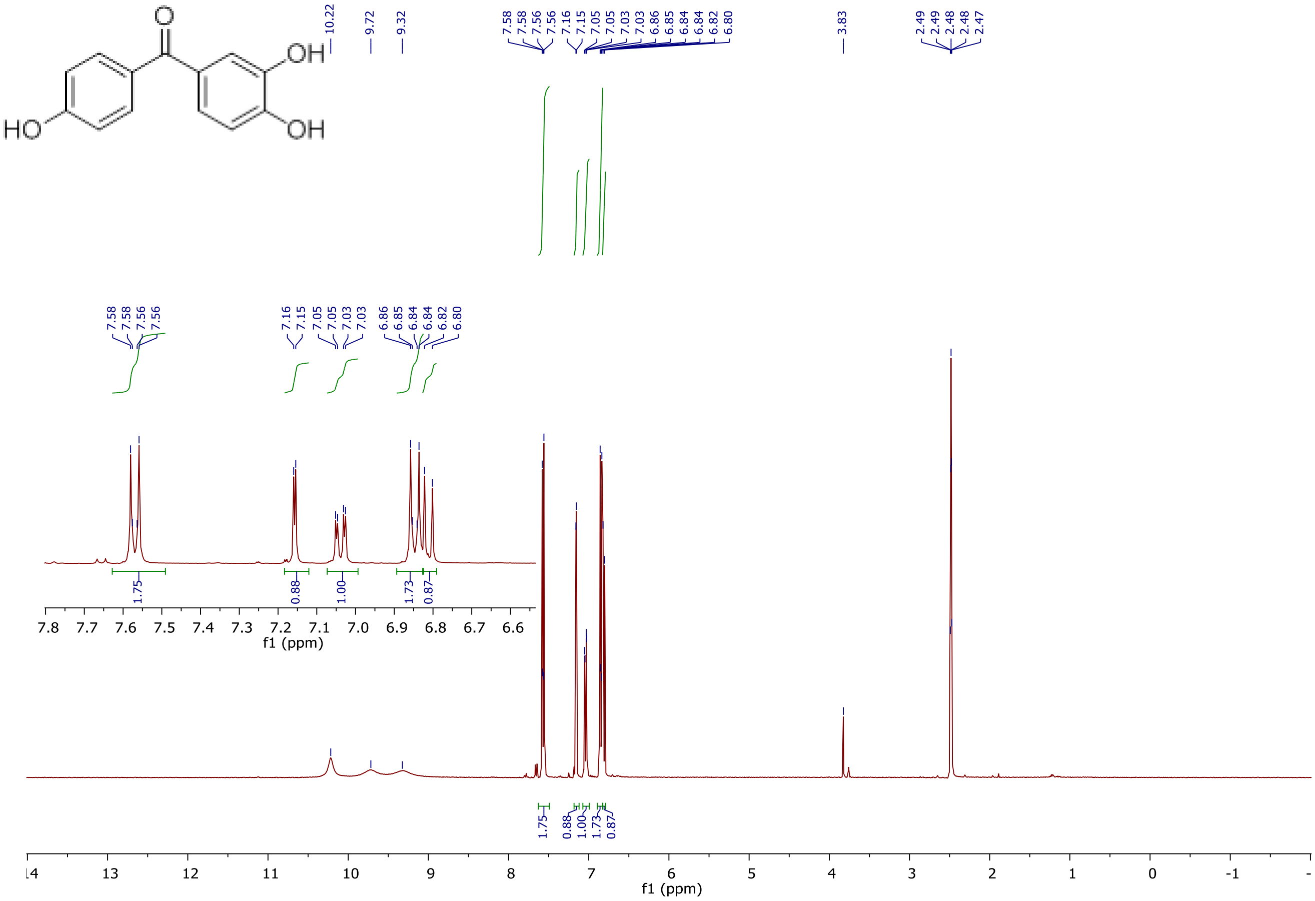

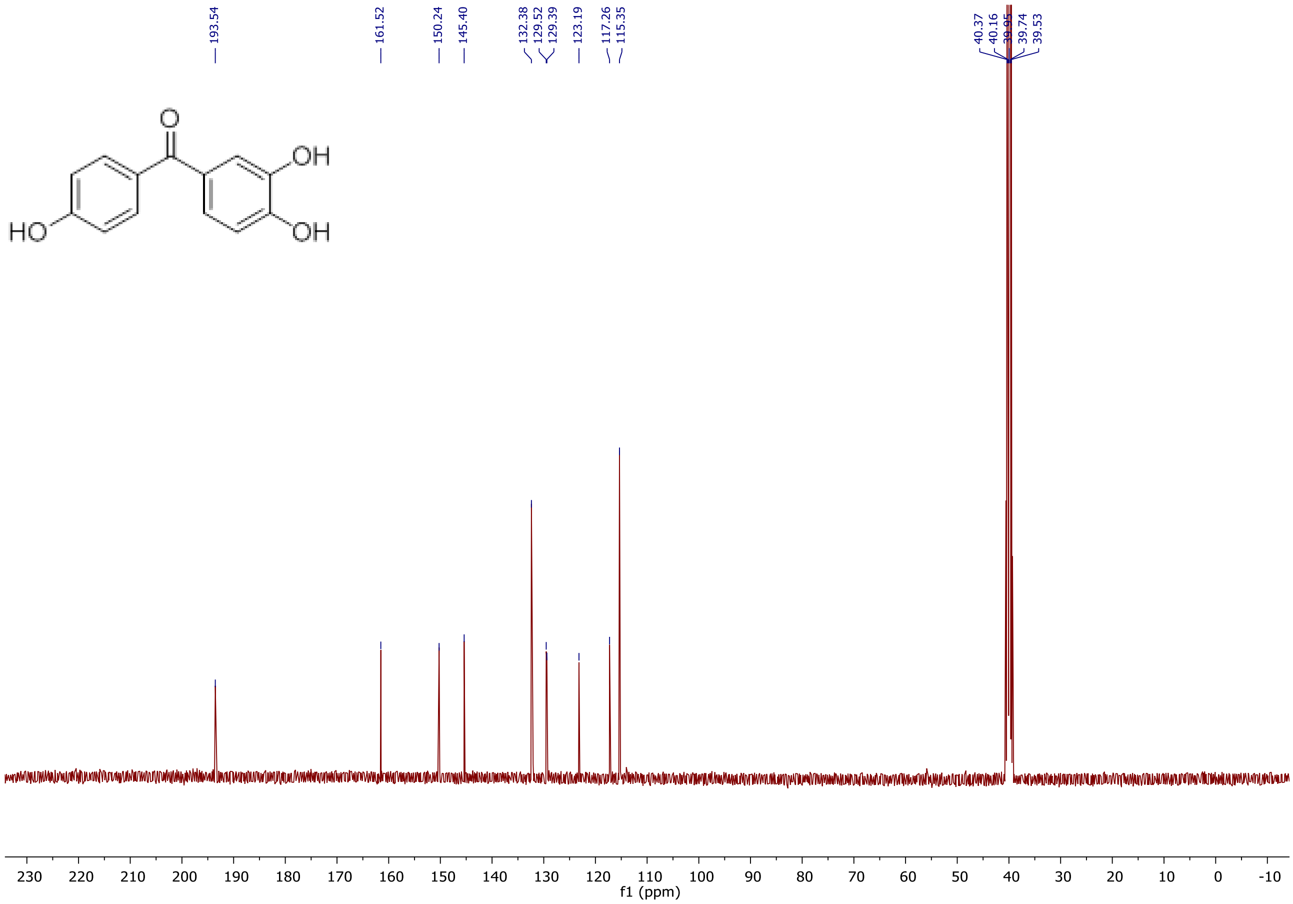

Bis(3,4-dimethoxyphenyl)methanone (**208**)

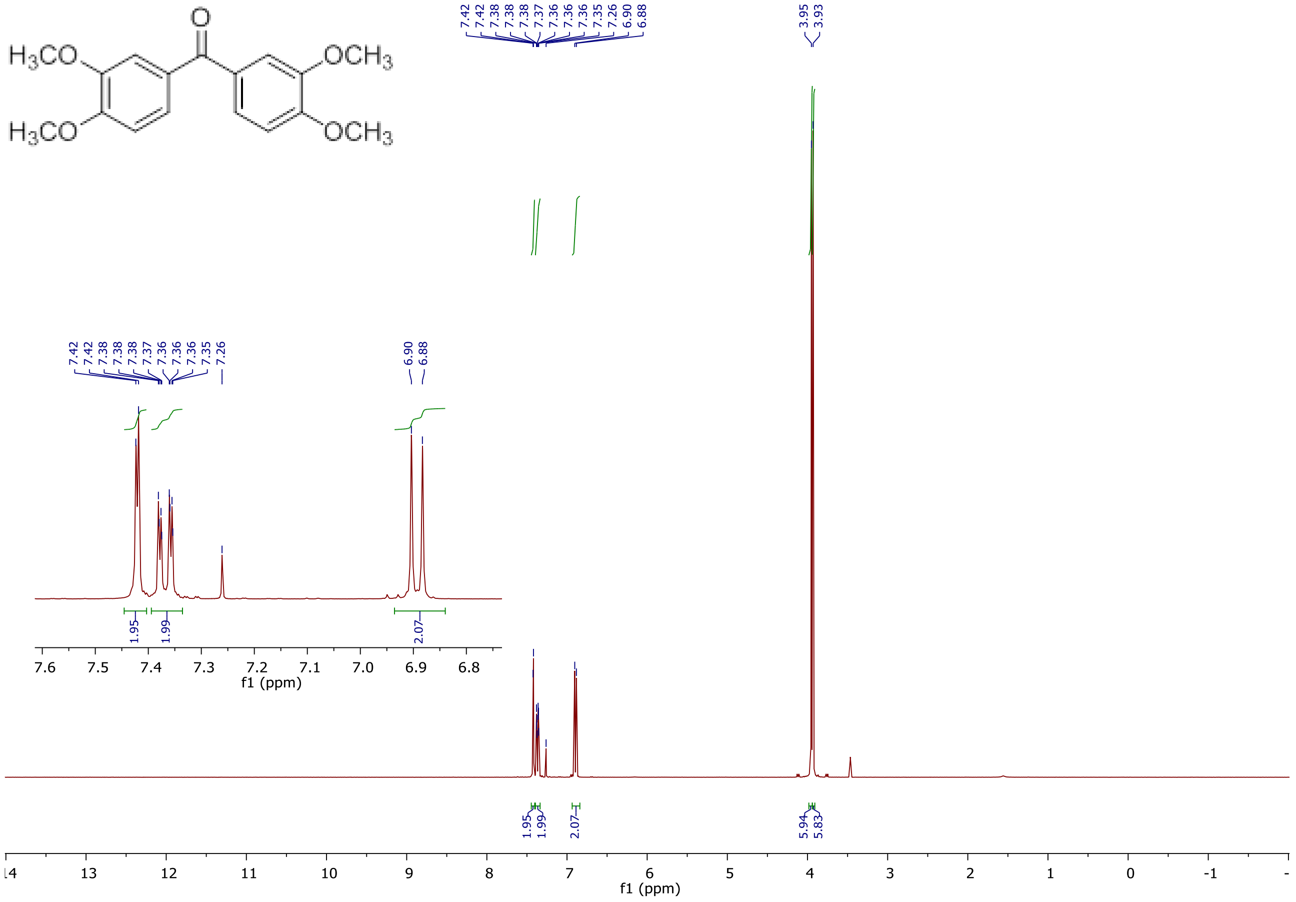

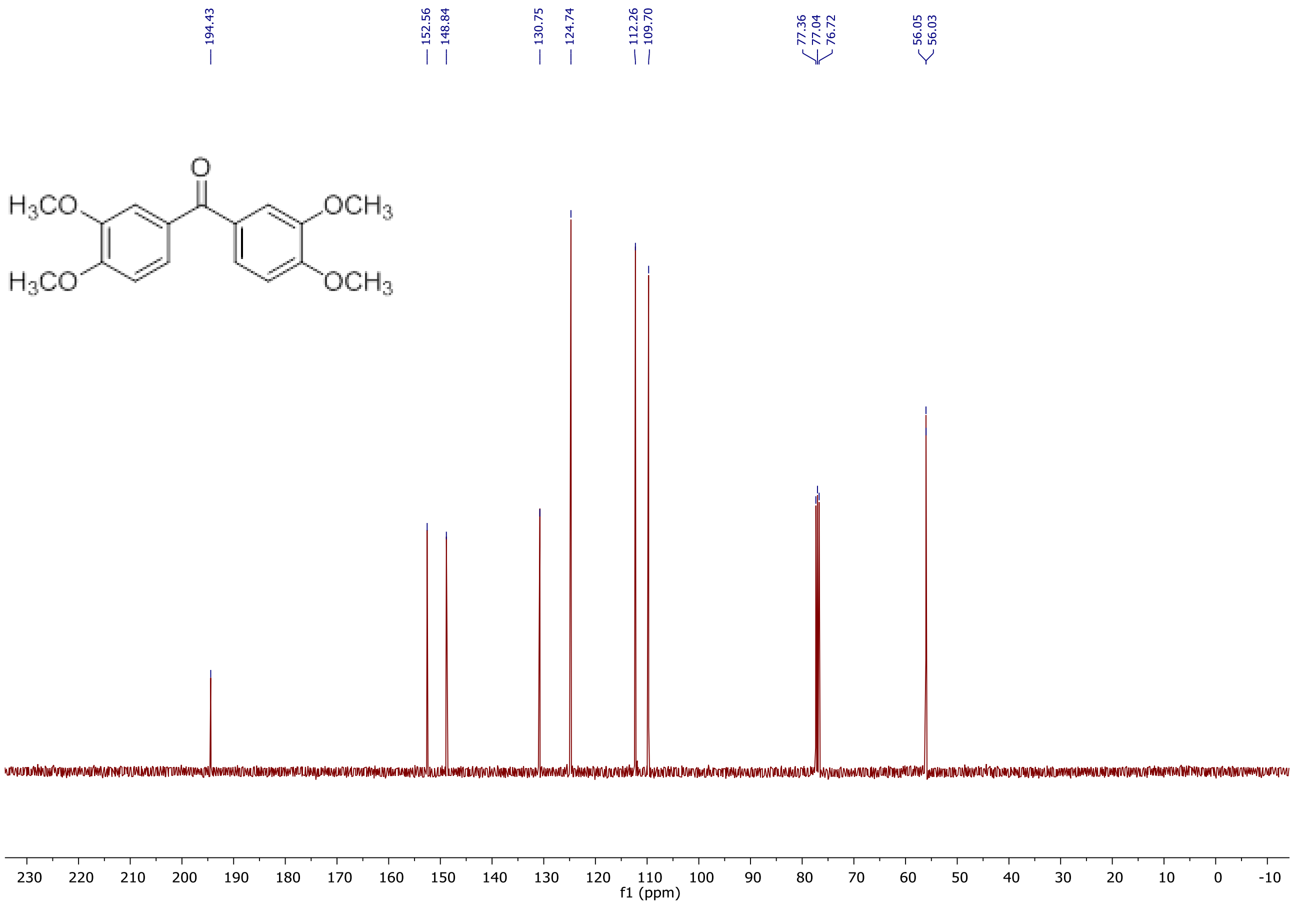

Bis(3,4-dihydroxyphenyl)methanone (**211**)

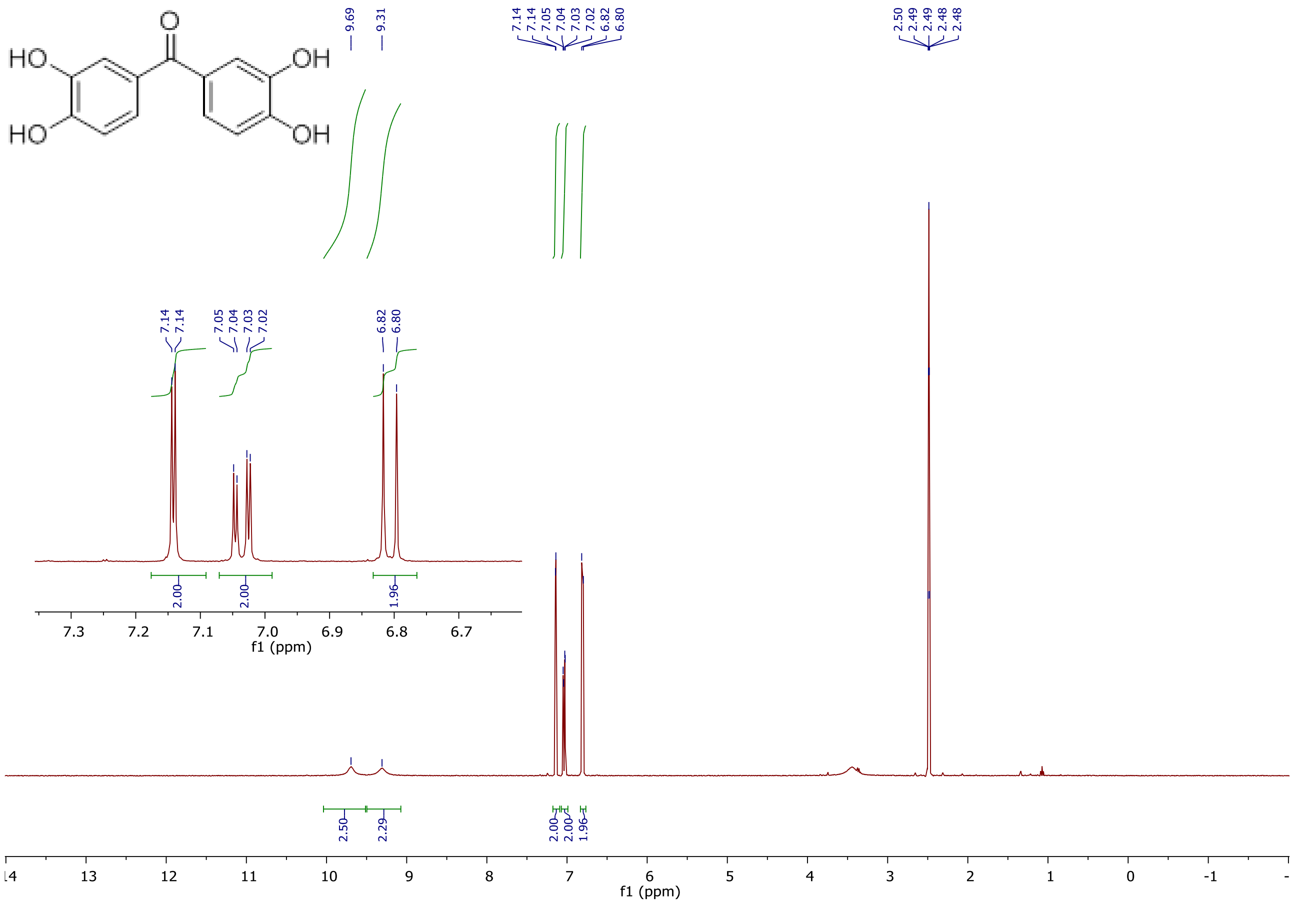

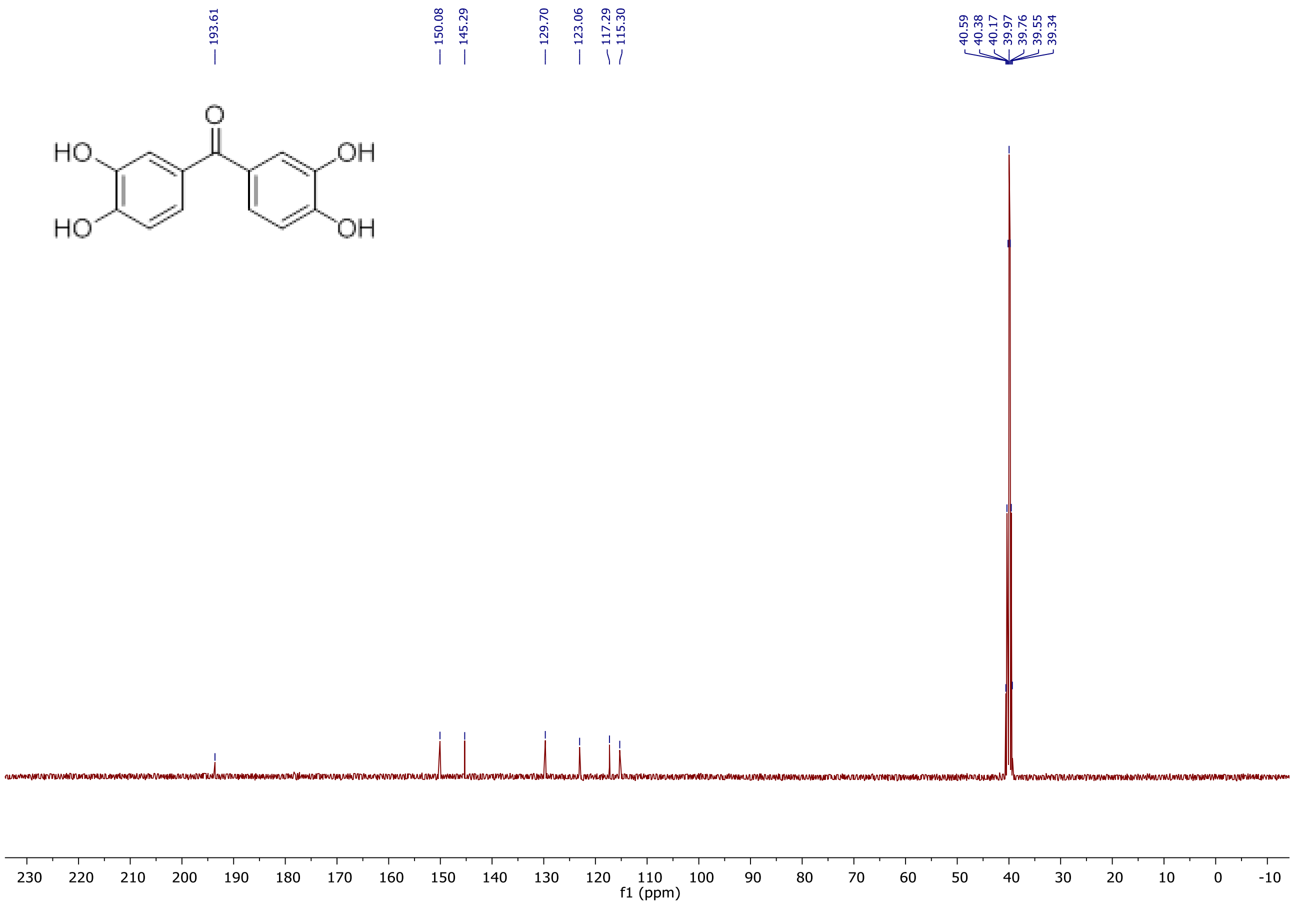

Supplement: Supplementary file 1 [file pharmaceuticals-16-00668-s001.zip › pharmaceuticals-2352161-supplementary.pdf]
